# Review of lattice results concerning low-energy particle physics: Flavour Lattice Averaging Group (FLAG)

**DOI:** 10.1140/epjc/s10052-016-4509-7

**Published:** 2017-02-17

**Authors:** S. Aoki, Y. Aoki, D. Bečirević, C. Bernard, T. Blum, G. Colangelo, M. Della Morte, P. Dimopoulos, S. Dürr, H. Fukaya, M. Golterman, Steven Gottlieb, S. Hashimoto, U. M. Heller, R. Horsley, A. Jüttner, T. Kaneko, L. Lellouch, H. Leutwyler, C.-J. D. Lin, V. Lubicz, E. Lunghi, R. Mawhinney, T. Onogi, C. Pena, C. T. Sachrajda, S. R. Sharpe, S. Simula, R. Sommer, A. Vladikas, U. Wenger, H. Wittig

**Affiliations:** 1 Center for Gravitational Physics, Yukawa Institute for Theoretical Physics, Kyoto University, Kitashirakawa Oiwakecho, Sakyo-ku, Kyoto, 606-8502 Japan; 2 Kobayashi-Maskawa Institute for the Origin of Particles and the Universe (KMI), Nagoya University, Nagoya, 464-8602 Japan; 3 Brookhaven National Laboratory, RIKEN BNL Research Center, Upton, NY 11973 USA; 4 Laboratoire de Physique Théorique (UMR8627), CNRS, Université Paris-Sud, Université Paris-Saclay, 91405 Orsay, France; 5 Department of Physics, Washington University, Saint Louis, MO 63130 USA; 6 Physics Department, University of Connecticut, Storrs, CT 06269-3046 USA; 7 Albert Einstein Center for Fundamental Physics, Institut für Theoretische Physik, Universität Bern, Sidlerstr. 5, 3012 Bern, Switzerland; 8 CP3-Origins and Danish IAS, University of Southern Denmark, Campusvej 55, 5230 Odense M, Denmark; 9 IFIC (CSIC), c/ Catedrático José Beltrán, 2, 46980 Paterna, Spain; 10 Centro Fermi-Museo Storico della Fisica e Centro Studi e Ricerche Enrico Fermi Compendio del Viminale, Piazza del Viminiale 1, 00184 Rome, Italy; 11 c/o Dipartimento di Fisica, Università di Roma Tor Vergata, Via della Ricerca Scientifica 1, 00133 Rome, Italy; 12 University of Wuppertal, Gaußstraße 20, 42119 Wuppertal, Germany; 13 Jülich Supercomputing Center, Forschungszentrum Jülich, 52425 Jülich, Germany; 14 Department of Physics, Osaka University, Toyonaka, Osaka 560-0043 Japan; 15 Department of Physics and Astronomy, San Francisco State University, San Francisco, CA 94132 USA; 16 Department of Physics, Indiana University, Bloomington, IN 47405 USA; 17 High Energy Accelerator Research Organization (KEK), Tsukuba, 305-0801 Japan; 18 School of High Energy Accelerator Science, The Graduate University for Advanced Studies (Sokendai), Tsukuba, 305-0801 Japan; 19 American Physical Society (APS), One Research Road, Ridge, NY 11961 USA; 20 Higgs Centre for Theoretical Physics, School of Physics and Astronomy, University of Edinburgh, Edinburgh, EH9 3FD UK; 21 School of Physics and Astronomy, University of Southampton, Southampton, SO17 1BJ UK; 22 Centre de Physique Théorique, UMR 7332, CNRS, Aix-Marseille Université, Université de Toulon, 13288 Marseille, France; 23 Institute of Physics, National Chiao-Tung University, Hsinchu, 30010 Taiwan; 24 Dipartimento di Matematica e Fisica, Università Roma Tre, Via della Vasca Navale 84, 00146 Rome, Italy; 25 Sezione di Roma Tre, INFN, Via della Vasca Navale 84, 00146 Rome, Italy; 26 Physics Department, Columbia University, New York, NY 10027 USA; 27 Departamento de Física Teórica, Instituto de Física Teórica UAM/CSIC, Universidad Autónoma de Madrid, Cantoblanco, 28049 Madrid, Spain; 28 Physics Department, University of Washington, Seattle, WA 98195-1560 USA; 29 John von Neumann Institute for Computing (NIC), DESY, Platanenallee 6, 15738 Zeuthen, Germany; 30 Sezione di Tor Vergata, INFN, c/o Dipartimento di Fisica, Università di Roma Tor Vergata, Via della Ricerca Scientifica 1, 00133 Rome, Italy; 31 PRISMA Cluster of Excellence, Institut für Kernphysik and Helmholtz Institute Mainz, University of Mainz, 55099 Mainz, Germany

## Abstract

We review lattice results related to pion, kaon, *D*- and *B*-meson physics with the aim of making them easily accessible to the particle-physics community. More specifically, we report on the determination of the light-quark masses, the form factor $f_+(0)$, arising in the semileptonic $K \rightarrow \pi$ transition at zero momentum transfer, as well as the decay constant ratio $f_K/f_\pi$ and its consequences for the CKM matrix elements $V_{us}$ and $V_{ud}$. Furthermore, we describe the results obtained on the lattice for some of the low-energy constants of $SU(2)_L\times SU(2)_R$ and $SU(3)_L\times SU(3)_R$ Chiral Perturbation Theory. We review the determination of the $B_K$ parameter of neutral kaon mixing as well as the additional four *B* parameters that arise in theories of physics beyond the Standard Model. The latter quantities are an addition compared to the previous review. For the heavy-quark sector, we provide results for $m_c$ and $m_b$ (also new compared to the previous review), as well as those for *D*- and *B*-meson-decay constants, form factors, and mixing parameters. These are the heavy-quark quantities most relevant for the determination of CKM matrix elements and the global CKM unitarity-triangle fit. Finally, we review the status of lattice determinations of the strong coupling constant $\alpha _s$.

## Introduction

**Table 1:** Summary of the main results of this review, grouped in terms of $N_{ f}$, the number of dynamical quark flavours in lattice simulations. Quark masses and the quark condensate are given in the ${\overline{\text {MS}}}$ scheme at running scale $\mu =2~\mathrm{GeV}$ or as indicated; the other quantities listed are specified in the quoted sections. For each result we list the references that entered the FLAG average or estimate. From the entries in this column one can also read off the number of results that enter our averages for each quantity. We emphasize that these numbers only give a very rough indication of how thoroughly the quantity in question has been explored on the lattice and recommend to consult the detailed tables and figures in the relevant section for more significant information and for explanations on the source of the quoted errors

Quantity	Sects.	$N_f=2+1+1$	Refs.	$N_f=2+1$	Refs.	$N_f=2$	Refs.
$$ m_s$$ [MeV]	3.1.3	93.9(1.1)	[4, 5]	92.0(2.1)	[6–10]	101(3)	[11, 12]
$$ m_{ud}$$ [MeV]	3.1.3	3.70(17)	[4]	3.373(80)	[7–10, 13]	3.6(2)	[11]
$m_s / m_{ud}$	3.1.4	27.30(34)	[4, 14]	27.43(31)	[6–8, 10]	27.3(9)	[11]
$m_u$ [MeV]	3.1.5	2.36(24)	[4]	2.16(9)(7)	${}^{\mathrm{a}}$	2.40(23)	[16]
$m_d$ [MeV]	3.1.5	5.03(26)	[4]	4.68(14)(7)	${}^{\mathrm{a}}$	4.80(23)	[16]
${m_u}/{m_d}$	3.1.5	0.470(56)	[4]	0.46(2)(2)	${}^{\mathrm{a}}$	0.50(4)	[16]
$\overline{m}_c(3~\hbox {GeV})$ [GeV]	3.2	0.996(25)	[4, 5]	0.987(6)	[9, 17]	1.03(4)	[11]
$m_c / m_s$	3.2.4	11.70(6)	[4, 5, 14]	11.82(16)	[17, 18]	11.74(35)	[11, 132]
$\overline{m}_b(\overline{m}_b)$ [GeV]	3.3.4	4.190(21)	[5, 19]	4.164(23)	[9]	4.256(81)	[20, 21]
$f_+(0)$	4.3	0.9704(24)(22)	[22]	0.9677(27)	[23, 24]	0.9560(57)(62)	[25]
$f_{K^\pm } / f_{\pi ^\pm }$	4.3	1.193(3)	[14, 26, 27]	1.192(5)	[28–31]	1.205(6)(17)	[32]
$f_{\pi ^\pm }$ [MeV]	4.6			130.2(1.4)	[28, 29, 31]		
$f_{K^\pm }$ [MeV]	4.6	155.6(4)	[14, 26, 27]	155.9(9)	[28, 29, 31]	157.5(2.4)	[32]
$\Sigma ^{1/3}$ [MeV]	5.2.1	280(8)(15)	[33]	274(3)	[10, 13, 34, 35]	266(10)	[33, 36–38]
${F_\pi }/{F}$	5.2.1	1.076(2)(2)	[39]	1.064(7)	[10, 29, 34, 35, 40]	1.073(15)	[36–38, 41]
$\bar{\ell }_3$	5.2.2	3.70(7)(26)	[39]	2.81(64)	[10, 29, 34, 35, 40]	3.41(82)	[36, 37, 41]
$\bar{\ell }_4$	5.2.2	4.67(3)(10)	[39]	4.10(45)	[10, 29, 34, 35, 40]	4.51(26)	[36, 37, 41]
$\bar{\ell }_6$	5.2.2					15.1(1.2)	[37, 41]
$\hat{B}_\mathrm{{K}}$	6.1	0.717(18)(16)	[42]	0.7625(97)	[10, 43–45]	0.727(22)(12)	[46]

$^{\mathrm{a}}$ This is a FLAG estimate, based on $\chi$PT and the isospin averaged up- and down-quark mass $m_{ud}$ [7–10, 13]

Flavour physics provides an important opportunity for exploring the limits of the Standard Model of particle physics and for constraining possible extensions that go beyond it. As the LHC explores a new energy frontier and as experiments continue to extend the precision frontier, the importance of flavour physics will grow, both in terms of searches for signatures of new physics through precision measurements and in terms of attempts to construct the theoretical framework behind direct discoveries of new particles. A major theoretical limitation consists in the precision with which strong-interaction effects can be quantified. Large-scale numerical simulations of lattice QCD allow for the computation of these effects from first principles. The scope of the Flavour Lattice Averaging Group (FLAG) is to review the current status of lattice results for a variety of physical quantities in low-energy physics. Set up in November 2007 it comprises experts in Lattice Field Theory, Chiral Perturbation Theory and Standard Model phenomenology. Our aim is to provide an answer to the frequently posed question “What is currently the best lattice value for a particular quantity?” in a way that is readily accessible to nonlattice-experts. This is generally not an easy question to answer; different collaborations use different lattice actions (discretizations of QCD) with a variety of lattice spacings and volumes, and with a range of masses for the *u*- and *d*-quarks. Not only are the systematic errors different, but also the methodology used to estimate these uncertainties varies between collaborations. In the present work we summarize the main features of each of the calculations and provide a framework for judging and combining the different results. Sometimes it is a single result that provides the “best” value; more often it is a combination of results from different collaborations. Indeed, the consistency of values obtained using different formulations adds significantly to our confidence in the results.

The first two editions of the FLAG review were published in 2011 [1] and 2014 [2]. The second edition reviewed results related to both light (*u*-, *d*- and *s*-), and heavy (*c*- and *b*-) flavours. The quantities related to pion and kaon physics were light-quark masses, the form factor $f_+(0)$ arising in semileptonic $K \rightarrow \pi$ transitions (evaluated at zero momentum transfer), the decay constants $f_K$ and $f_\pi$, and the $B_\mathrm{K}$ parameter from neutral kaon mixing. Their implications for the CKM matrix elements $V_{us}$ and $V_{ud}$ were also discussed. Furthermore, results were reported for some of the low-energy constants of $SU(2)_L \times SU(2)_R$ and $SU(3)_L \times SU(3)_R$ Chiral Perturbation Theory. The quantities related to *D*- and *B*-meson physics that were reviewed were the *B*- and *D*-meson-decay constants, form factors, and mixing parameters. These are the heavy–light quantities most relevant to the determination of CKM matrix elements and the global CKM unitarity-triangle fit. Last but not least, the current status of lattice results on the QCD coupling $\alpha _s$ was reviewed.

In the present paper we provide updated results for all the above-mentioned quantities, but also extend the scope of the review in two ways. First, we now present results for the charm and bottom quark masses, in addition to those of the three lightest quarks. Second, we review results obtained for the kaon mixing matrix elements of new operators that arise in theories of physics beyond the Standard Model. Our main results are collected in Tables 1 and 2.

Our plan is to continue providing FLAG updates, in the form of a peer reviewed paper, roughly on a biennial basis. This effort is supplemented by our more frequently updated website http://itpwiki.unibe.ch/flag [3], where figures as well as pdf-files for the individual sections can be downloaded. The papers reviewed in the present edition have appeared before the closing date **30 November 2015**.

**Table 2:** Summary of the main results of this review, grouped in terms of $N_{ f}$, the number of dynamical quark flavours in lattice simulations. The quantities listed are specified in the quoted sections. For each result we list the references that entered the FLAG average or estimate. From the entries in this column one can also read off the number of results that enter our averages for each quantity. We emphasize that these numbers only give a very rough indication of how thoroughly the quantity in question has been explored on the lattice and recommend to consult the detailed tables and figures in the relevant section for more significant information and for explanations on the source of the quoted errors

Quantity	Sects.	$N_f=2+1+1$	Refs.	$N_f=2+1$	Refs.	$N_f=2$	Refs.
$$ f_D$$ [MeV]	7.1	212.15(1.45)	[14, 27]	209.2(3.3)	[47, 48]	208(7)	[20]
$$ f_{D_s}$$ [MeV]	7.1	248.83(1.27)	[14, 27]	249.8(2.3)	[17, 48, 49]	250(7)	[20]
$$ {{f_{D_s}}/{f_D}}$$	7.1	1.1716(32)	[14, 27]	1.187(12)	[47, 48]	1.20(2)	[20]
$f_+^{D\pi }(0)$	7.2			0.666(29)	[50]		
$f_+^{DK}(0)$	7.2			0.747(19)	[51]		
$$ f_B$$ [MeV]	8.1	186(4)	[52]	192.0(4.3)	[48, 53–56]	188(7)	[20, 57, 58]
$$ f_{B_s}$$ [MeV]	8.1	224(5)	[52]	228.4(3.7)	[48, 53–56]	227(7)	[20, 57, 58]
$$ {{f_{B_s}}/{f_B}}$$	8.1	1.205(7)	[52]	1.201(16)	[48, 53–55]	1.206(23)	[20, 57, 58]
$f_{B_d}\sqrt{\hat{B}_{B_d}}$ [MeV]	8.2			219(14)	[54, 59]	216(10)	[20]
$f_{B_s}\sqrt{\hat{B}_{B_s}}$ [MeV]	8.2			270(16)	[54, 59]	262(10)	[20]
$\hat{B}_{B_d}$	8.2			1.26(9)	[54, 59]	1.30(6)	[20]
$\hat{B}_{B_s}$	8.2			1.32(6)	[54, 59]	1.32(5)	[20]
$\xi$	8.2			1.239(46)	[54, 60]	1.225(31)	[20]
$B_{B_s}/B_{B_d}$	8.2			1.039(63)	[54, 60]	1.007(21)	[20]

Quantity	Sects.	$N_f=2+1$ and $N_f=2+1+1$			Refs.		
$\alpha _{\overline{\mathrm{MS}}}^{(5)}(M_Z)$	9.9	0.1182(12)			[5, 9, 61–63]		
$\Lambda _{\overline{\mathrm{MS}}}^{(5)}$ [MeV]	9.9	211(14)			[5, 9, 61–63]		

This review is organized as follows. In the remainder of Sect. 1 we summarize the composition and rules of FLAG and discuss general issues that arise in modern lattice calculations. In Sect. 2 we explain our general methodology for evaluating the robustness of lattice results. We also describe the procedures followed for combining results from different collaborations in a single average or estimate (see Sect. 2.2 for our definition of these terms). The rest of the paper consists of sections, each dedicated to a single (or groups of closely connected) physical quantity(ies). Each of these sections is accompanied by an Appendix with explicatory notes.

### FLAG composition, guidelines and rules

FLAG strives to be representative of the lattice community, both in terms of the geographical location of its members and the lattice collaborations to which they belong. We aspire to provide the particle-physics community with a single source of reliable information on lattice results.

In order to work reliably and efficiently, we have adopted a formal structure and a set of rules by which all FLAG members abide. The collaboration presently consists of an Advisory Board (AB), an Editorial Board (EB), and seven Working Groups (WG). The rôle of the Advisory Board is that of general supervision and consultation. Its members may interfere at any point in the process of drafting the paper, expressing their opinion and offering advice. They also give their approval of the final version of the preprint before it is rendered public. The Editorial Board coordinates the activities of FLAG, sets priorities and intermediate deadlines, and takes care of the editorial work needed to amalgamate the sections written by the individual working groups into a uniform and coherent review. The working groups concentrate on writing up the review of the physical quantities for which they are responsible, which is subsequently circulated to the whole collaboration for critical evaluation.

The current list of FLAG members and their Working Group assignments is:

- Advisory Board (AB):    S. Aoki, C. Bernard, M. Golterman, H. Leutwyler, and C. Sachrajda
- Editorial Board (EB):   G. Colangelo, A. Jüttner, S. Hashimoto, S. Sharpe, A. Vladikas, and U. Wenger
- Working Groups (coordinator listed first):
  - Quark masses    L. Lellouch, T. Blum, and V. Lubicz
  - $V_{us},V_{ud}$    S. Simula, P. Boyle,^1^ and T. Kaneko
  - LEC    S. Dürr, H. Fukaya, and U.M. Heller
  - $B_K$    H. Wittig, P. Dimopoulos, and R. Mawhinney
  - $f_{B_{(s)}}$, $f_{D_{(s)}}$, $B_B$    M. Della Morte, Y. Aoki, and D. Lin
  - $B_{(s)}$, *D* semileptonic and radiative decays E. Lunghi, D. Becirevic, S. Gottlieb, and C. Pena
  - $\alpha _s$    R. Sommer, R. Horsley, and T. Onogi

As some members of the WG on quark masses were faced with unexpected hindrances, S. Simula has kindly assisted in the completion of the relevant section during the final phases of its composition.

The most important FLAG guidelines and rules are the following:

- the composition of the AB reflects the main geographical areas in which lattice collaborations are active, with members from America, Asia/Oceania and Europe;
- the mandate of regular members is not limited in time, but we expect that a certain turnover will occur naturally;
- whenever a replacement becomes necessary this has to keep, and possibly improve, the balance in FLAG, so that different collaborations, from different geographical areas are represented;
- in all working groups the three members must belong to three different lattice collaborations;^2^
- a paper is in general not reviewed (nor colour-coded, as described in the next section) by any of its authors;
- lattice collaborations not represented in FLAG will be consulted on the colour coding of their calculation;
- there are also internal rules regulating our work, such as voting procedures.

### Citation policy

We draw attention to this particularly important point. As stated above, our aim is to make lattice QCD results easily accessible to nonlattice-experts and we are well aware that it is likely that some readers will only consult the present paper and not the original lattice literature. It is very important that this paper be not the only one cited when our results are quoted. We strongly suggest that readers also cite the original sources. In order to facilitate this, in Tables 1 and 2, besides summarizing the main results of the present review, we also cite the original references from which they have been obtained. In addition, for each figure we make a bibtex-file available on our webpage [3] which contains the bibtex-entries of all the calculations contributing to the FLAG average or estimate. The bibliography at the end of this paper should also make it easy to cite additional papers. Indeed we hope that the bibliography will be one of the most widely used elements of the whole paper.

### General issues

Several general issues concerning the present review are thoroughly discussed in Sect. 1.1 of our initial 2010 paper [1] and we encourage the reader to consult the relevant pages. In the remainder of the present subsection, we focus on a few important points. Though the discussion has been duly updated, it is essentially that of Sect. 1.2 of the 2013 review [2].

The present review aims to achieve two distinct goals: first, to provide a **description** of the work done on the lattice concerning low-energy particle physics; and, second, to draw **conclusions** on the basis of that work, summarizing the results obtained for the various quantities of physical interest.

The core of the information as regards the work done on the lattice is presented in the form of tables, which not only list the various results, but also describe the quality of the data that underlie them. We consider it important that this part of the review represents a generally accepted description of the work done. For this reason, we explicitly specify the quality requirements^3^ used and provide sufficient details in appendices so that the reader can verify the information given in the tables.

On the other hand, the conclusions drawn on the basis of the available lattice results are the responsibility of FLAG alone. Preferring to err on the side of caution, in several cases we draw conclusions that are more conservative than those resulting from a plain weighted average of the available lattice results. This cautious approach is usually adopted when the average is dominated by a single lattice result, or when only one lattice result is available for a given quantity. In such cases one does not have the same degree of confidence in results and errors as when there is agreement among several different calculations using different approaches. The reader should keep in mind that the degree of confidence cannot be quantified, and it is not reflected in the quoted errors.

Each discretization has its merits, but also its shortcomings. For most topics covered in this review we have an increasingly broad database, and for most quantities lattice calculations based on totally different discretizations are now available. This is illustrated by the dense population of the tables and figures in most parts of this review. Those calculations that do satisfy our quality criteria indeed lead to consistent results, confirming universality within the accuracy reached. In our opinion, the consistency between independent lattice results, obtained with different discretizations, methods, and simulation parameters, is an important test of lattice QCD, and observing such consistency also provides further evidence that systematic errors are fully under control.

In the sections dealing with heavy quarks and with $\alpha _s$, the situation is not the same. Since the *b*-quark mass cannot be resolved with current lattice spacings, all lattice methods for treating *b* quarks use effective field theory at some level. This introduces additional complications not present in the light-quark sector. An overview of the issues specific to heavy-quark quantities is given in the introduction of Sect. 8. For *B* and *D* meson leptonic decay constants, there already exist a good number of different independent calculations that use different heavy-quark methods, but there are only one or two independent calculations of semileptonic *B* and *D* meson form factors and *B* meson mixing parameters. For $\alpha _s$, most lattice methods involve a range of scales that need to be resolved and controlling the systematic error over a large range of scales is more demanding. The issues specific to determinations of the strong coupling are summarized in Sect. 9.

*Number of sea quarks in lattice simulations:*

Lattice QCD simulations currently involve two, three or four flavours of dynamical quarks. Most simulations set the masses of the two lightest quarks to be equal, while the strange and charm quarks, if present, are heavier (and tuned to lie close to their respective physical values). Our notation for these simulations indicates which quarks are nondegenerate, e.g. $N_{ f}=2+1$ if $m_u=m_d < m_s$ and $N_{ f}=2+1+1$ if $m_u=m_d< m_s < m_c$. Calculations with $N_{ f}=2$, i.e. two degenerate dynamical flavours, often include strange valence quarks interacting with gluons, so that bound states with the quantum numbers of the kaons can be studied, albeit neglecting strange sea-quark fluctuations. The quenched approximation ($N_f=0$), in which sea-quark contributions are omitted, has uncontrolled systematic errors and is no longer used in modern lattice simulations with relevance to phenomenology. Accordingly, we will review results obtained with $N_f=2$, $N_f=2+1$, and $N_f = 2+1+1$, but omit earlier results with $N_f=0$. The only exception concerns the QCD coupling constant $\alpha _s$. Since this observable does not require valence light quarks, it is theoretically well defined also in the $N_f=0$ theory, which is simply pure gluon-dynamics. The $N_f$-dependence of $\alpha _s$, or more precisely of the related quantity $r_0 \Lambda _{\overline{\text {MS}}}$, is a theoretical issue of considerable interest; here $r_0$ is a quantity with the dimension of length, which sets the physical scale, as discussed in Appendix A.2. We stress, however, that only results with $N_f \ge 3$ are used to determine the physical value of $\alpha _s$ at a high scale.

*Lattice actions, simulation parameters and scale setting:*

The remarkable progress in the precision of lattice calculations is due to improved algorithms, better computing resources and, last but not least, conceptual developments. Examples of the latter are improved actions that reduce lattice artefacts and actions that preserve chiral symmetry to very good approximation. A concise characterization of the various discretizations that underlie the results reported in the present review is given in Appendix A.1.

Physical quantities are computed in lattice simulations in units of the lattice spacing so that they are dimensionless. For example, the pion decay constant that is obtained from a simulation is $f_\pi a$, where *a* is the spacing between two neighbouring lattice sites. To convert these results to physical units requires knowledge of the lattice spacing *a* at the fixed values of the bare QCD parameters (quark masses and gauge coupling) used in the simulation. This is achieved by requiring agreement between the lattice calculation and experimental measurement of a known quantity, which thus “sets the scale” of a given simulation. A few details of this procedure are provided in Appendix A.2.

*Renormalization and scheme dependence:*

Several of the results covered by this review, such as quark masses, the gauge coupling, and *B*-parameters, are for quantities defined in a given renormalization scheme and at a specific renormalization scale. The schemes employed (e.g. regularization-independent MOM schemes) are often chosen because of their specific merits when combined with the lattice regularization. For a brief discussion of their properties, see Appendix A.3. The conversion of the results, obtained in these so-called intermediate schemes, to more familiar regularization schemes, such as the ${\overline{\text {MS}}}$-scheme, is done with the aid of perturbation theory. It must be stressed that the renormalization scales accessible in simulations are limited, because of the presence of an ultraviolet (UV) cutoff of ${\sim } \pi /a$. To safely match to ${\overline{\text {MS}}}$, a scheme defined in perturbation theory, Renormalization Group (RG) running to higher scales is performed, either perturbatively or nonperturbatively (the latter using finite-size scaling techniques).

*Extrapolations:*

Because of limited computing resources, lattice simulations are often performed at unphysically heavy pion masses, although results at the physical point have become increasingly common. Further, numerical simulations must be done at nonzero lattice spacing, and in a finite (four-dimensional) volume. In order to obtain physical results, lattice data are obtained at a sequence of pion masses and a sequence of lattice spacings, and then extrapolated to the physical-pion mass and to the continuum limit. In principle, an extrapolation to infinite volume is also required. However, for most quantities discussed in this review, finite-volume effects are exponentially small in the linear extent of the lattice in units of the pion mass and, in practice, one often verifies volume independence by comparing results obtained on a few different physical volumes, holding other parameters equal. To control the associated systematic uncertainties, these extrapolations are guided by effective theories. For light-quark actions, the lattice-spacing dependence is described by Symanzik’s effective theory [64, 65]; for heavy quarks, this can be extended and/or supplemented by other effective theories such as Heavy-Quark Effective Theory (HQET). The pion-mass dependence can be parameterized with Chiral Perturbation Theory ($\chi$PT), which takes into account the Nambu–Goldstone nature of the lowest excitations that occur in the presence of light quarks. Similarly, one can use Heavy-Light Meson Chiral Perturbation Theory (HM$\chi$PT) to extrapolate quantities involving mesons composed of one heavy (*b* or *c*) and one light quark. One can combine Symanzik’s effective theory with $\chi$PT to simultaneously extrapolate to the physical-pion mass and the continuum; in this case, the form of the effective theory depends on the discretization. See Appendix A.4 for a brief description of the different variants in use and some useful references. Finally, $\chi$PT can also be used to estimate the size of finite-volume effects measured in units of the inverse pion mass, thus providing information on the systematic error due to finite-volume effects in addition to that obtained by comparing simulations at different volumes.

*Critical slowing down:*

The lattice spacings reached in recent simulations go down to 0.05 fm or even smaller. In this regime, long autocorrelation times slow down the sampling of the configurations [66–75]. Many groups check for autocorrelations in a number of observables, including the topological charge, for which a rapid growth of the autocorrelation time is observed with decreasing lattice spacing. This is often referred to as topological freezing. A solution to the problem consists in using open boundary conditions in time, instead of the more common antiperiodic ones [76]. More recently two other approaches have been proposed, one based on a multiscale thermalization algorithm [77] and another based on defining QCD on a nonorientable manifold [78]. The problem is also touched upon in Sect. 9.2, where it is stressed that attention must be paid to this issue. While large-scale simulations with open boundary conditions are already far advanced [79], unfortunately so far no results reviewed here have been obtained with any of the above methods. It is usually *assumed* that the continuum limit can be reached by extrapolation from the existing simulations and that potential systematic errors due to the long autocorrelation times have been adequately controlled.

*Simulation algorithms and numerical errors:*

Most of the modern lattice-QCD simulations use exact algorithms such as those of Refs. [80, 81], which do not produce any systematic errors when exact arithmetic is available. In reality, one uses numerical calculations at double (or in some cases even single) precision, and some errors are unavoidable. More importantly, the inversion of the Dirac operator is carried out iteratively and it is truncated once some accuracy is reached, which is another source of potential systematic error. In most cases, these errors have been confirmed to be much less than the statistical errors. In the following we assume that this source of error is negligible. Some of the most recent simulations use an inexact algorithm in order to speed-up the computation, though it may produce systematic effects. Currently available tests indicate that errors from the use of inexact algorithms are under control.

## Quality criteria, averaging and error estimation

The essential characteristics of our approach to the problem of rating and averaging lattice quantities have been outlined in our first publication [1]. Our aim is to help the reader assess the reliability of a particular lattice result without necessarily studying the original article in depth. This is a delicate issue, since the ratings may make things appear simpler than they are. Nevertheless, it safeguards against the common practice of using lattice results, and drawing physics conclusions from them, without a critical assessment of the quality of the various calculations. We believe that, despite the risks, it is important to provide some compact information as regards the quality of a calculation. We stress, however, the importance of the accompanying detailed discussion of the results presented in the various sections of the present review.

### Systematic errors and colour code

The major sources of systematic error are common to most lattice calculations. These include, as discussed in detail below, the chiral, continuum and infinite-volume extrapolations. To each such source of error for which systematic improvement is possible we assign one of three coloured symbols: green star, unfilled green circle (which replaced in Ref. [2] the amber disk used in the original FLAG review [1]) or red square. These correspond to the following ratings:

the parameter values and ranges used to generate the datasets allow for a satisfactory control of the systematic uncertainties;
the parameter values and ranges used to generate the datasets allow for a reasonable attempt at estimating systematic uncertainties, which, however, could be improved;
the parameter values and ranges used to generate the datasets are unlikely to allow for a reasonable control of systematic uncertainties.

The appearance of a red tag, even in a single source of systematic error of a given lattice result, disqualifies it from inclusion in the global average.

The attentive reader will notice that these criteria differ from those used in Refs. [1, 2]. In the previous FLAG editions we used the three symbols in order to rate the reliability of the systematic errors attributed to a given result by the paper’s authors. This sometimes proved to be a daunting task, as the methods used by some collaborations for estimating their systematics are not always explained in full detail. Moreover, it is sometimes difficult to disentangle and rate different uncertainties, since they are interwoven in the error analysis. Thus, in the present edition we have opted for a different approach: the three symbols rate the quality of a particular simulation, based on the values and range of the chosen parameters, and its aptness to obtain well-controlled systematic uncertainties. They do not rate the quality of the analysis performed by the authors of the publication. The latter question is deferred to the relevant sections of the present review, which contain detailed discussions of the results contributing (or not) to each FLAG average or estimate. As a result of this different approach to the rating criteria, as well as changes of the criteria themselves, the colour coding of some papers in the current FLAG version differs from that of Ref. [2].

For most quantities the colour-coding system refers to the following sources of systematic errors: (i) chiral extrapolation; (ii) continuum extrapolation; (iii) finite volume. As we will see below, renormalization is another source of systematic uncertainties in several quantities. This we also classify using the three coloured symbols listed above, but now with a different rationale: they express how reliably these quantities are renormalized, from a field-theoretic point of view (namely nonperturbatively, or with two-loop or one-loop perturbation theory).

Given the sophisticated status that the field has attained, several aspects, besides those rated by the coloured symbols, need to be evaluated before one can conclude whether a particular analysis leads to results that should be included in an average or estimate. Some of these aspects are not so easily expressible in terms of an adjustable parameter such as the lattice spacing, the pion mass or the volume. As a result of such considerations, it sometimes occurs, albeit rarely, that a given result does not contribute to the FLAG average or estimate, despite not carrying any red tags. This happens, for instance, whenever aspects of the analysis appear to be incomplete (e.g. an incomplete error budget), so that the presence of inadequately controlled systematic effects cannot be excluded. This mostly refers to results with a statistical error only, or results in which the quoted error budget obviously fails to account for an important contribution.

Of course any colour coding has to be treated with caution; we emphasize that the criteria are subjective and evolving. Sometimes a single source of systematic error dominates the systematic uncertainty and it is more important to reduce this uncertainty than to aim for green stars for other sources of error. In spite of these caveats we hope that our attempt to introduce quality measures for lattice simulations will prove to be a useful guide. In addition we would like to stress that the agreement of lattice results obtained using different actions and procedures provides further validation.

#### Systematic effects and rating criteria

The precise criteria used in determining the colour coding are unavoidably time-dependent; as lattice calculations become more accurate, the standards against which they are measured become tighter. For this reason, some of the quality criteria related to the light-quark sector have been tightened up between the first [1] and second [2] editions of FLAG.

In the second edition we have also reviewed quantities related to heavy-quark physics [2]. The criteria used for light- and heavy-flavour quantities were not always the same. For the continuum limit, the difference was more a matter of choice: the light-flavour Working Groups defined the ratings using conditions involving specific values of the lattice spacing, whereas the heavy-flavour Working Groups preferred more data-driven criteria. Also, for finite-volume effects, the heavy-flavour groups slightly relaxed the boundary between    and , compared to the light-quark case, to account for the fact that heavy-quark quantities are less sensitive to the finiteness of the volume.

In the present edition we have opted for simplicity and adopted unified criteria for both light- and heavy-flavoured quantities.^4^ The colour code used in the tables is specified as follows:

- Chiral extrapolation:

$$M_{\pi ,\mathrm {min}}< 200$$ MeV
200 MeV $$\le M_{\pi ,{\mathrm {min}}} \le $$ 400 MeV
400 MeV $$ < M_{\pi ,\mathrm {min}}$$

It is assumed that the chiral extrapolation is performed with at least a 3-point analysis; otherwise this will be explicitly mentioned. This condition is unchanged from Ref. [2].

- Continuum extrapolation:

at least three lattice spacings and at least 2 points below 0.1 fm and a range of lattice spacings satisfying $[a_{\mathrm {max}}/a_{\mathrm {min}}]^2 \ge 2$
at least two lattice spacings and at least 1 point below 0.1 fm and a range of lattice spacings satisfying $[a_{\mathrm {max}}/a_{\mathrm {min}}]^2 \ge 1.4$
otherwise

It is assumed that the lattice action is $\mathcal {O}(a)$-improved (i.e. the discretization errors vanish quadratically with the lattice spacing); otherwise this will be explicitly mentioned. For unimproved actions an additional lattice spacing is required. This condition has been tightened compared to that of Ref. [2] by the requirements concerning the range of lattice spacings.

- Finite-volume effects:

$[M_{\pi ,\mathrm {min}} / M_{\pi ,\mathrm {fid}}]^2 \exp \{4-M_{\pi ,\mathrm {min}}[L(M_{\pi ,\mathrm {min}})]_{\mathrm {max}}\} < 1$, or at least 3 volumes
$[M_{\pi ,\mathrm {min}} / M_{\pi ,\mathrm {fid}}]^2 \exp \{3-M_{\pi ,\mathrm {min}}[L(M_{\pi ,\mathrm {min}})]_{\mathrm {max}}\} < 1$, or at least 2 volumes
otherwise

- It is assumed here that calculations are in the *p*-regime^5^ of chiral perturbation theory, and that all volumes used exceed 2 fm. Here we are using a more sophisticated condition than that of Ref. [2]. The new condition involves the quantity $[L(M_{\pi ,\mathrm {min}})]_{\mathrm {max}}$, which is the maximum box size used in the simulations performed at smallest pion mass $M_{\pi ,\mathrm {min}}$, as well as a fiducial pion mass $M_{\pi ,\mathrm {fid}}$, which we set to 200 MeV (the cutoff value for a green star in the chiral extrapolation).
  The rationale for this condition is as follows. Finite-volume effects contain the universal factor $\exp \{- L~M_\pi \}$, and if this were the only contribution a criterion based on the values of $M_{\pi ,\text {min}} L$ would be appropriate. This is what we used in Ref. [2] (with $M_{\pi ,\text {min}} L>4$ for    and $M_{\pi ,\text {min}} L>3$ for ). However, as pion masses decrease, one must also account for the weakening of the pion couplings. In particular, one-loop chiral perturbation theory [82] reveals a behaviour proportional to $M_\pi ^2 \exp \{- L~M_\pi \}$. Our new condition includes this weakening of the coupling and ensures, for example, that simulations with $M_{\pi ,\mathrm {min}} = 135~\mathrm{MeV}$ and $L~M_{\pi ,\mathrm {min}} = 3.2$ are rated equivalently to those with $M_{\pi ,\mathrm {min}} = 200~\mathrm{MeV}$ and $L~M_{\pi ,\mathrm {min}} = 4$.

- Renormalization (where applicable):

nonperturbative
one-loop perturbation theory or higher with a reasonable estimate of truncation errors
otherwise

- In Ref. [1], we assigned a red square to all results which were renormalized at one-loop in perturbation theory. In Ref. [2] we decided that this was too restrictive, since the error arising from renormalization constants, calculated in perturbation theory at one-loop, is often estimated conservatively and reliably.

- Renormalization Group (RG) running (where applicable):
  For scale-dependent quantities, such as quark masses or $B_K$, it is essential that contact with continuum perturbation theory can be established. Various different methods are used for this purpose (cf. Appendix A.3): Regularization-independent Momentum Subtraction (RI/MOM), the Schrödinger functional, and direct comparison with (resummed) perturbation theory. Irrespective of the particular method used, the uncertainty associated with the choice of intermediate renormalization scales in the construction of physical observables must be brought under control. This is best achieved by performing comparisons between nonperturbative and perturbative running over a reasonably broad range of scales. These comparisons were initially only made in the Schrödinger functional approach, but are now also being performed in RI/MOM schemes. We mark the data for which information as regards nonperturbative running checks is available and give some details, but do not attempt to translate this into a colour code.

The pion mass plays an important role in the criteria relevant for chiral extrapolation and finite volume. For some of the regularizations used, however, it is not a trivial matter to identify this mass.

In the case of twisted-mass fermions, discretization effects give rise to a mass difference between charged and neutral pions even when the up- and down-quark masses are equal: the charged pion is found to be the heavier of the two for twisted-mass Wilson fermions (cf. Ref. [83]). In early work, typically referring to $N_f=2$ simulations (e.g. Refs. [83] and [36]), chiral extrapolations are based on chiral perturbation theory formulae which do not take these regularization effects into account. After the importance of keeping the isospin breaking when doing chiral fits was shown in Ref. [84], later work, typically referring to $N_f=2+1+1$ simulations, has taken these effects into account [4]. We use $M_{\pi ^\pm }$ for $M_{\pi ,\mathrm {min}}$ in the chiral-extrapolation rating criterion. On the other hand, sea quarks (corresponding to both charged and neutral “sea pions“ in an effective-chiral-theory logic) as well as valence quarks are intertwined with finite-volume effects. Therefore, we identify $M_{\pi ,\mathrm {min}}$ with the root mean square (RMS) of $M_{\pi ^+}$, $M_{\pi ^-}$ and $M_{\pi ^0}$ in the finite-volume rating criterion.^6^

In the case of staggered fermions, discretization effects give rise to several light states with the quantum numbers of the pion.^7^ The mass splitting among these “taste” partners represents a discretization effect of $\mathcal {O}(a^2)$, which can be significant at large lattice spacings but shrinks as the spacing is reduced. In the discussion of the results obtained with staggered quarks given in the following sections, we assume that these artefacts are under control. We conservatively identify $M_{\pi ,\mathrm {min}}$ with the root mean square (RMS) average of the masses of all the taste partners, both for chiral-extrapolation and finite-volume criteria.^8^

The strong coupling $\alpha _s$ is computed in lattice QCD with methods differing substantially from those used in the calculations of the other quantities discussed in this review. Therefore we have established separate criteria for $\alpha _s$ results, which will be discussed in Sect. 9.2.

#### Heavy-quark actions

In most cases, and in particular for the *b* quark, the discretization of the heavy-quark action follows a very different approach to that used for light flavours. There are several different methods for treating heavy quarks on the lattice, each with their own issues and considerations. All of these methods use Effective Field Theory (EFT) at some point in the computation, either via direct simulation of the EFT, or by using EFT as a tool to estimate the size of cutoff errors, or by using EFT to extrapolate from the simulated lattice quark masses up to the physical *b*-quark mass. Because of the use of an EFT, truncation errors must be considered together with discretization errors.

The charm quark lies at an intermediate point between the heavy and light quarks. In our previous review, the bulk of the calculations involving charm quarks treated it using one of the approaches adopted for the *b* quark. Many recent calculations, however, simulate the charm quark using light-quark actions, in particular the $N_f=2+1+1$ calculations. This has become possible thanks to the increasing availability of dynamical gauge field ensembles with fine lattice spacings. But clearly, when charm quarks are treated relativistically, discretization errors are more severe than those of the corresponding light-quark quantities.

In order to address these complications, we add a new heavy-quark treatment category to the rating system. The purpose of this criterion is to provide a guideline for the level of action and operator improvement needed in each approach to make reliable calculations possible, in principle.

A description of the different approaches to treating heavy quarks on the lattice is given in Appendix A.1.3, including a discussion of the associated discretization, truncation, and matching errors. For truncation errors we use HQET power counting throughout, since this review is focussed on heavy-quark quantities involving *B* and *D* mesons rather than bottomonium or charmonium quantities. Here we describe the criteria for how each approach must be implemented in order to receive an acceptable () rating for both the heavy-quark actions and the weak operators. Heavy-quark implementations without the level of improvement described below are rated not acceptable (). The matching is evaluated together with renormalization, using the renormalization criteria described in Sect. 2.1.1. We emphasize that the heavy-quark implementations rated as acceptable and described below have been validated in a variety of ways, such as via phenomenological agreement with experimental measurements, consistency between independent lattice calculations, and numerical studies of truncation errors. These tests are summarized in Sect. 8.

*Relativistic heavy-quark actions:*

 at least tree-level $\mathcal {O}(a)$ improved action and weak operators.

This is similar to the requirements for light-quark actions. All current implementations of relativistic heavy-quark actions satisfy this criterion.

*NRQCD*

 tree-level matched through $\mathcal {O}(1/m_h)$ and improved through $\mathcal {O}(a^2)$.

The current implementations of NRQCD satisfy this criterion, and also include tree-level corrections of $\mathcal {O}(1/m_h^2)$ in the action.

*HQET*

 tree-level matched through $\mathcal {O}(1/m_h)$ with discretization errors starting at $\mathcal {O}(a^2)$.

The current implementation of HQET by the ALPHA Collaboration satisfies this criterion, since both action and weak operators are matched nonperturbatively through $\mathcal {O}(1/m_h)$. Calculations that exclusively use a static-limit action do not satisfy this criterion, since the static-limit action, by definition, does not include $1/m_h$ terms. We therefore consider static computations in our final estimates only if truncation errors (in $1/m_h$) are discussed and included in the systematic uncertainties.

*Light-quark actions for heavy quarks*

 discretization errors starting at $\mathcal {O}(a^2)$ or higher.

This applies to calculations that use the tmWilson action, a nonperturbatively improved Wilson action, or the HISQ action for charm-quark quantities. It also applies to calculations that use these light-quark actions in the charm region and above together with either the static limit or with an HQET inspired extrapolation to obtain results at the physical *b* quark mass. In these cases, the continuum extrapolation criteria described earlier must be applied to the entire range of heavy-quark masses used in the calculation.

#### Conventions for the figures

For a coherent assessment of the present situation, the quality of the data plays a key role, but the colour coding cannot be carried over to the figures. On the other hand, simply showing all data on equal footing would give the misleading impression that the overall consistency of the information available on the lattice is questionable. Therefore, in the figures we indicate the quality of the data in a rudimentary way, using the following symbols:

corresponds to results included in the average or estimate (i.e. results that contribute to the black square below);
corresponds to results that are not included in the average but pass all quality criteria;
corresponds to all other results;
corresponds to FLAG averages or estimates; they are also highlighted by a grey vertical band.

The reason for not including a given result in the average is not always the same: the result may fail one of the quality criteria; the paper may be unpublished; it may be superseded by newer results; or it may not offer a complete error budget.

Symbols other than squares are used to distinguish results with specific properties and are always explained in the caption.^9^

Often nonlattice data are also shown in the figures for comparison. For these we use the following symbols:

corresponds to nonlattice results;
corresponds to Particle Data Group (PDG) results.

### Averages and estimates

FLAG results of a given quantity are denoted either as *averages* or as *estimates*. Here we clarify this distinction. To start with, both *averages* and *estimates* are based on results without any red tags in their colour coding. For many observables there are enough independent lattice calculations of good quality, with all sources of error (not merely those related to the colour-coded criteria), as analysed in the original papers, appearing to be under control. In such cases it makes sense to average these results and propose such an *average* as the best current lattice number. The averaging procedure applied to this data and the way the error is obtained is explained in detail in Sect. 2.3. In those cases where only a sole result passes our rating criteria (colour coding), we refer to it as our FLAG *average*, provided it also displays adequate control of all other sources of systematic uncertainty.

On the other hand, there are some cases in which this procedure leads to a result that, in our opinion, does not cover all uncertainties. Systematic error estimates are by their nature often subjective and difficult to estimate, and may thus end up being underestimated in one or more results that receive green symbols for all explicitly tabulated criteria. Adopting a conservative policy, in these cases we opt for an *estimate* (or a range), which we consider as a fair assessment of the knowledge acquired on the lattice at present. This *estimate* is not obtained with a prescribed mathematical procedure, but reflects what we consider the best possible analysis of the available information. The hope is that this will encourage more detailed investigations by the lattice community.

There are two other important criteria that also play a role in this respect, but that cannot be colour coded, because a systematic improvement is not possible. These are: (i) the publication status, and (ii) the number of sea-quark flavours $N_{ f}$. As far as the former criterion is concerned, we adopt the following policy: we average only results that have been published in peer-reviewed journals, i.e. they have been endorsed by referee(s). The only exception to this rule consists in straightforward updates of previously published results, typically presented in conference proceedings. Such updates, which supersede the corresponding results in the published papers, are included in the averages. Note that updates of earlier results rely, at least partially, on the same gauge-field-configuration ensembles. For this reason, we do not average updates with earlier results. Nevertheless, all results are listed in the tables,^10^ and their publication status is identified by the following symbols:

- Publication status:
  A   published or plain update of published results
  P   preprint
  C   conference contribution.

In the present edition, the publication status on the **30th of November 2015** is relevant. If the paper appeared in print after that date, this is accounted for in the bibliography, but does not affect the averages.

As noted above, in this review we present results from simulations with $N_f=2$, $N_f=2+1$ and $N_f=2+1+1$ (except for $r_0 \Lambda _{\overline{\text {MS}}}$ where we also give the $N_f=0$ result). We are not aware of an a priori way to quantitatively estimate the difference between results produced in simulations with a different number of dynamical quarks. We therefore average results at fixed $N_{ f}$ separately; averages of calculations with different $N_{ f}$ will not be provided.

To date, no significant differences between results with different values of $N_f$ have been observed in the quantities listed in Tables 1 and 2. In the future, as the accuracy and the control over systematic effects in lattice calculations increases, it will hopefully be possible to see a difference between results from simulations with $N_{ f}= 2$ and $N_{ f}= 2 + 1$, and thus determine the size of the Zweig-rule violations related to strange-quark loops. This is a very interesting issue *per se*, and one which can be quantitatively addressed only with lattice calculations.

The question of differences between results with $N_{ f}=2+1$ and $N_{ f}=2+1+1$ is more subtle. The dominant effect of including the charm sea quark is to shift the lattice scale, an effect that is accounted for by fixing this scale nonperturbatively using physical quantities. For most of the quantities discussed in this review, it is expected that residual effects are small in the continuum limit, suppressed by $\alpha _s(m_c)$ and powers of $\Lambda ^2/m_c^2$. Here $\Lambda$ is a hadronic scale that can only be roughly estimated and depends on the process under consideration. Note that the $\Lambda ^2/m_c^2$ effects have been addressed in Ref. [90]. Assuming that such effects are small, it might be reasonable to average the results from $N_{ f}=2+1$ and $N_{ f}=2+1+1$ simulations. This is not yet a pressing issue in this review, since there are relatively few results with $N_{ f}=2+1+1$, but it will become a more important question in the future.

### Averaging procedure and error analysis

In the present report we repeatedly average results obtained by different collaborations and estimate the error on the resulting averages. We follow the procedure of the previous edition [2], which we describe here in full detail.

One of the problems arising when forming averages is that not all of the datasets are independent. In particular, the same gauge-field configurations, produced with a given fermion discretization, are often used by different research teams with different valence-quark lattice actions, obtaining results that are not really independent. Our averaging procedure takes such correlations into account.

Consider a given measurable quantity *Q*, measured by *M* distinct, not necessarily uncorrelated, numerical experiments (simulations). The result of each of these measurement is expressed as

1 $$\begin{aligned} Q_i = x_i \, \pm \, \sigma ^{(1)}_i \pm \, \sigma ^{(2)}_i \pm \cdots \pm \, \sigma ^{(E)}_i, \end{aligned}$$

where $x_i$ is the value obtained by the *i*th experiment ($i = 1, \ldots , M$) and $\sigma ^{(k)}_i$ (for $k = 1, \ldots , E$) are the various errors. Typically $\sigma ^{(1)}_i$ stands for the statistical error and $\sigma ^{(k)}_i$ ($k \ge 2$) are the different systematic errors from various sources. For each individual result, we estimate the total error $\sigma _i$ by adding statistical and systematic errors in quadrature:

2 $$\begin{aligned} Q_i= & {} x_i \pm \sigma _i,\nonumber \\ \sigma _i\equiv & {} \sqrt{\sum _{k=1}^E [\sigma ^{(k)}_i]^2}. \end{aligned}$$

With the weight factor of each total error estimated in standard fashion:

3 $$\begin{aligned} \omega _i =\dfrac{\sigma _i^{-2}}{\sum _{i=1}^M \sigma _i^{-2}}, \end{aligned}$$

the central value of the average over all simulations is given by

4 $$\begin{aligned} x_\mathrm{av} =\sum _{i=1}^M x_i\, \omega _i. \end{aligned}$$

The above central value corresponds to a $\chi _\mathrm{min}^2$ weighted average, evaluated by adding statistical and systematic errors in quadrature. If the fit is not of good quality ($\chi _\mathrm{min}^2/\hbox {d.o.f.} > 1$), the statistical and systematic error bars are stretched by a factor $S = \sqrt{\chi ^2/\hbox {d.o.f.}}$

Next we examine error budgets for individual calculations and look for potentially correlated uncertainties. Specific problems encountered in connection with correlations between different data sets are described in the text that accompanies the averaging. If there is reason to believe that a source of error is correlated between two calculations, a $100\%$ correlation is assumed. The correlation matrix $C_{ij}$ for the set of correlated lattice results is estimated by a prescription due to Schmelling [91]. This consists in defining

5 $$\begin{aligned} \sigma _{i;j} =\sqrt{\underset{(k)}{\displaystyle \sum \nolimits ^\prime }[ \sigma _i^{(k)}]^2}, \end{aligned}$$

with $\sum _{(k)}^\prime$ running only over those errors of $x_i$ that are correlated with the corresponding errors of measurement $x_j$. This expresses the part of the uncertainty in $x_i$ that is correlated with the uncertainty in $x_j$. If no such correlations are known to exist, then we take $\sigma _{i;j} =0$. The diagonal and off-diagonal elements of the correlation matrix are then taken to be

6 $$\begin{aligned} C_{ii}= & {} \sigma _i^2 \quad (i = 1, \ldots , M),\nonumber \\ C_{ij}= & {} \sigma _{i;j} \, \sigma _{j;i} \quad (i \ne j). \end{aligned}$$

Finally the error of the average is estimated by

7 $$\begin{aligned} \sigma ^2_\mathrm{av} =\sum _{i=1}^M \sum _{j=1}^M \omega _i \,\omega _j \,C_{ij}, \end{aligned}$$

and the FLAG average is

8 $$\begin{aligned} Q_\mathrm{av} =x_\mathrm{av} \pm \sigma _\mathrm{av}. \end{aligned}$$

## Quark masses

Quark masses are fundamental parameters of the Standard Model. An accurate determination of these parameters is important for both phenomenological and theoretical applications. The charm and bottom masses, for instance, enter the theoretical expressions of several cross sections and decay rates in heavy-quark expansions. The up-, down- and strange-quark masses govern the amount of explicit chiral symmetry breaking in QCD. From a theoretical point of view, the values of quark masses provide information as regards the flavour structure of physics beyond the Standard Model. The Review of Particle Physics of the Particle Data Group contains a review of quark masses [92], which covers light as well as heavy flavours. Here we also consider light- and heavy- quark masses, but focus on lattice results and discuss them in more detail. We do not discuss the top quark, however, because it decays weakly before it can hadronize, and the nonperturbative QCD dynamics described by present day lattice simulations is not relevant. The lattice determination of light- (up, down, strange), charm- and bottom-quark masses is considered in Sects. 3.1, 3.2, and 3.3, respectively.

Quark masses cannot be measured directly in experiment because quarks cannot be isolated, as they are confined inside hadrons. On the other hand, quark masses are free parameters of the theory and, as such, cannot be obtained on the basis of purely theoretical considerations. Their values can only be determined by comparing the theoretical prediction for an observable, which depends on the quark mass of interest, with the corresponding experimental value.

In the last edition of this review [2], quark-mass determinations came from two- and three-flavour QCD calculations. Moreover, these calculations were most often performed in the isospin limit, where the up- and down-quark masses (especially those in the sea) are set equal. In addition, some of the results retained in our light-quark mass averages were based on simulations performed at values of $m_{ud}$ which were still substantially larger than its physical value imposing a significant extrapolation to reach the physical up- and down-quark mass point. Among the calculations performed near physical $m_{ud}$ by PACS-CS [93–95], BMW [7, 8] and RBC/UKQCD [31], only the ones in Refs. [7, 8] did so while controlling all other sources of systematic error.

Today, however, the effects of the charm quark in the sea are more and more systematically considered and most of the new quark-mass results discussed below have been obtained in $N_f=2+1+1$ simulations by ETM [4], HPQCD [14] and FNAL/MILC [5]. In addition, RBC/UKQCD [10], HPQCD [14] and FNAL/MILC [5] are extending their calculations down to up-down-quark masses at or very close to their physical values while still controlling other sources of systematic error. Another aspect that is being increasingly addressed are electromagnetic and $(m_d-m_u)$, strong isospin-breaking effects. As we will see below these are particularly important for determining the individual up- and down-quark masses. But with the level of precision being reached in calculations, these effects are also becoming important for other quark masses.

Three-flavour QCD has four free parameters: the strong coupling, $\alpha _s$ (alternatively $\Lambda _\mathrm {QCD}$) and the up-, down- and strange-quark masses, $m_u$, $m_d$ and $m_s$. Four-flavour calculations have an additional parameter, the charm-quark mass $m_c$. When the calculations are performed in the isospin limit, up- and down-quark masses are replaced by a single parameter: the isospin-averaged up- and down-quark mass, $m_{ud}=\frac{1}{2}(m_u+m_d)$. A lattice determination of these parameters, and in particular of the quark masses, proceeds in two steps:

1. One computes as many experimentally measurable quantities as there are quark masses. These observables should obviously be sensitive to the masses of interest, preferably straightforward to compute and obtainable with high precision. They are usually computed for a variety of input values of the quark masses which are then adjusted to reproduce experiment. Another observable, such as the pion decay constant or the mass of a member of the baryon octet, must be used to fix the overall scale. Note that the mass of a quark, such as the *b*, which is not accounted for in the generation of gauge configurations, can still be determined. For that an additional valence-quark observable containing this quark must be computed and the mass of that quark must be tuned to reproduce experiment.
2. The input quark masses are bare parameters which depend on the lattice spacing and particulars of the lattice regularization used in the calculation. To compare their values at different lattice spacings and to allow a continuum extrapolation they must be renormalized. This renormalization is a short-distance calculation, which may be performed perturbatively. Experience shows that one-loop calculations are unreliable for the renormalization of quark masses: usually at least two loops are required to have trustworthy results. Therefore, it is best to perform the renormalizations nonperturbatively to avoid potentially large perturbative uncertainties due to neglected higher-order terms. Nevertheless we will include in our averages one-loop results if they carry a solid estimate of the systematic uncertainty due to the truncation of the series.

In the absence of electromagnetic corrections, the renormalization factors for all quark masses are the same at a given lattice spacing. Thus, uncertainties due to renormalization are absent in ratios of quark masses if the tuning of the masses to their physical values can be done lattice spacing by lattice spacing and significantly reduced otherwise.

We mention that lattice QCD calculations of the *b*-quark mass have an additional complication which is not present in the case of the charm- and light-quarks. At the lattice spacings currently used in numerical simulations the direct treatment of the *b* quark with the fermionic actions commonly used for light quarks will result in large cutoff effects, because the *b*-quark mass is of order one in lattice units. There are a few widely used approaches to treat the *b* quark on the lattice, which have been already discussed in the FLAG 13 review (see Section 8 of Ref. [2]). Those relevant for the determination of the *b*-quark mass will be briefly described in Sect. 3.3.

### Masses of the light quarks

Light-quark masses are particularly difficult to determine because they are very small (for the up and down quarks) or small (for the strange quark) compared to typical hadronic scales. Thus, their impact on typical hadronic observables is minute, and it is difficult to isolate their contribution accurately.

Fortunately, the spontaneous breaking of $SU(3)_L\times SU(3)_R$ chiral symmetry provides observables which are particularly sensitive to the light-quark masses: the masses of the resulting Nambu–Goldstone bosons (NGB), i.e. pions, kaons and etas. Indeed, the Gell-Mann–Oakes–Renner relation [96] predicts that the squared mass of a NGB is directly proportional to the sum of the masses of the quark and antiquark which compose it, up to higher-order mass corrections. Moreover, because these NGBs are light and are composed of only two valence particles, their masses have a particularly clean statistical signal in lattice-QCD calculations. In addition, the experimental uncertainties on these meson masses are negligible. Thus, in lattice calculations, light-quark masses are typically obtained by renormalizing the input quark mass and tuning them to reproduce NGB masses, as described above.

#### Contributions from the electromagnetic interaction

As mentioned in Sect. 2.1, the present review relies on the hypothesis that, at low energies, the Lagrangian $\mathcal{L}_{\mathrm{QCD}}+\mathcal{L}_{\mathrm{QED}}$ describes nature to a high degree of precision. However, most of the results presented below are obtained in pure QCD calculations, which do not include QED. Quite generally, when comparing QCD calculations with experiment, radiative corrections need to be applied. In pure QCD simulations, where the parameters are fixed in terms of the masses of some of the hadrons, the electromagnetic contributions to these masses must be accounted for. Of course, once QED is included in lattice calculations, the subtraction of e.m. contributions is no longer necessary.

The electromagnetic interaction plays a particularly important role in determinations of the ratio $m_u/m_d$, because the isospin-breaking effects generated by this interaction are comparable to those from $m_u\ne m_d$ (see Sect. 3.1.5). In determinations of the ratio $m_s/m_{ud}$, the electromagnetic interaction is less important, but at the accuracy reached, it cannot be neglected. The reason is that, in the determination of this ratio, the pion mass enters as an input parameter. Because $M_\pi$ represents a small symmetry-breaking effect, it is rather sensitive to the perturbations generated by QED.

The decomposition of the sum $\mathcal{L}_{\mathrm{QCD}}+\mathcal{L}_{\mathrm{QED}}$ into two parts is not unique and specifying the QCD part requires a convention. In order to give results for the quark masses in the Standard Model at scale $\mu =2\,\hbox {GeV}$, on the basis of a calculation done within QCD, it is convenient to match the parameters of the two theories at that scale. We use this convention throughout the present review.^11^

Such a convention allows us to distinguish the physical mass $M_P$, $P\in \{\pi ^+,$ $\pi ^0$, $K^+$, $K^0\}$, from the mass $\hat{M}_P$ within QCD. The e.m. self-energy is the difference between the two, $M_P^\gamma \equiv M_P-\hat{M}_P$. Because the self-energy of the Nambu–Goldstone bosons diverges in the chiral limit, it is convenient to replace it by the contribution of the e.m. interaction to the *square* of the mass,

9 $$\begin{aligned} \Delta _{P}^\gamma \equiv M_P^2-\hat{M}_P^2= 2\,M_P M_P^\gamma +\mathcal {O}(e^4). \end{aligned}$$

The main effect of the e.m. interaction is an increase in the mass of the charged particles, generated by the photon cloud that surrounds them. The self-energies of the neutral ones are comparatively small, particularly for the Nambu–Goldstone bosons, which do not have a magnetic moment. Dashen’s theorem [102] confirms this picture, as it states that, to leading order (LO) of the chiral expansion, the self-energies of the neutral NGBs vanish, while the charged ones obey $\Delta _{K^+}^\gamma = \Delta _{\pi ^+}^\gamma$. It is convenient to express the self-energies of the neutral particles as well as the mass difference between the charged and neutral pions within QCD in units of the observed mass difference, $\Delta _\pi \equiv M_{\pi ^+}^2-M_{\pi ^0}^2$:

10 $$\begin{aligned} \Delta _{\pi ^0}^\gamma \equiv \epsilon _{\pi ^0}\,\Delta _\pi ,\quad \Delta _{K^0}^\gamma \equiv \epsilon _{K^0}\,\Delta _\pi ,\quad \!\!\hat{M}_{\pi ^+}^2- \hat{M}_{\pi ^0}^2\equiv \epsilon _m\,\Delta _\pi .\!\!\!\!\!\nonumber \\ \end{aligned}$$

In this notation, the self-energies of the charged particles are given by

11 $$\begin{aligned}&\Delta _{\pi ^+}^\gamma =(1+\epsilon _{\pi ^0}-\epsilon _m)\,\Delta _\pi ,\nonumber \\&\Delta _{K^+}^\gamma =(1+\epsilon +\epsilon _{K^0}-\epsilon _m)\,\Delta _\pi ,\end{aligned}$$

where the dimensionless coefficient $\epsilon$ parameterizes the violation of Dashen’s theorem,^12^

12 $$\begin{aligned} \Delta _{K^+}^\gamma -\Delta _{K^0}^\gamma - \Delta _{\pi ^+}^\gamma +\Delta _{\pi ^0}^\gamma \equiv \epsilon \,\Delta _\pi .\end{aligned}$$

Any determination of the light-quark masses based on a calculation of the masses of $\pi ^+,K^+$ and $K^0$ within QCD requires an estimate for the coefficients $\epsilon$, $\epsilon _{\pi ^0}$, $\epsilon _{K^0}$ and $\epsilon _m$.

The first determination of the self-energies on the lattice was carried out by Duncan, Eichten and Thacker [104]. Using the quenched approximation, they arrived at $M_{K^+}^\gamma -M_{K^0}^\gamma = 1.9\,\hbox {MeV}$. Actually, the parameterization of the masses given in that paper yields an estimate for all but one of the coefficients introduced above (since the mass splitting between the charged and neutral pions in QCD is neglected, the parameterization amounts to setting $\epsilon _m=0$ ab initio). Evaluating the differences between the masses obtained at the physical value of the electromagnetic coupling constant and at $e=0$, we obtain $\epsilon = 0.50(8)$, $\epsilon _{\pi ^0} = 0.034(5)$ and $\epsilon _{K^0} = 0.23(3)$. The errors quoted are statistical only: an estimate of lattice systematic errors is not possible from the limited results of Ref. [104]. The result for $\epsilon$ indicates that the violation of Dashen’s theorem is sizeable: according to this calculation, the nonleading contributions to the self-energy difference of the kaons amount to 50% of the leading term. The result for the self-energy of the neutral pion cannot be taken at face value, because it is small, comparable to the neglected mass difference $\hat{M}_{\pi ^+}-\hat{M}_{\pi ^0}$. To illustrate this, we note that the numbers quoted above are obtained by matching the parameterization with the physical masses for $\pi ^0$, $K^+$ and $K^0$. This gives a mass for the charged pion that is too high by 0.32 MeV. Tuning the parameters instead such that $M_{\pi ^+}$ comes out correctly, the result for the self-energy of the neutral pion becomes larger: $\epsilon _{\pi ^0}=0.10(7)$ where, again, the error is statistical only.

In an update of this calculation by the RBC Collaboration [105] (RBC 07), the electromagnetic interaction is still treated in the quenched approximation, but the strong interaction is simulated with $N_{ f}=2$ dynamical quark flavours. The quark masses are fixed with the physical masses of $\pi ^0$, $K^+$ and $K^0$. The outcome for the difference in the electromagnetic self-energy of the kaons reads $M_{K^+}^\gamma -M_{K^0}^\gamma = 1.443(55)\,\hbox {MeV}$. This corresponds to a remarkably small violation of Dashen’s theorem. Indeed, a recent extension of this work to $N_{ f}=2+1$ dynamical flavours [103] leads to a significantly larger self-energy difference: $M_{K^+}^\gamma -M_{K^0}^\gamma = 1.87(10)\,\hbox {MeV}$, in good agreement with the estimate of Eichten et al. Expressed in terms of the coefficient $\epsilon$ that measures the size of the violation of Dashen’s theorem, it corresponds to $\epsilon =0.5(1)$.

The input for the electromagnetic corrections used by MILC is specified in Ref. [106]. In their analysis of the lattice data, $\epsilon _{\pi ^0}$, $\epsilon _{K^0}$ and $\epsilon _m$ are set equal to zero. For the remaining coefficient, which plays a crucial role in determinations of the ratio $m_u/m_d$, the very conservative range $\epsilon =1(1)$ was used in MILC 04 [107], while in MILC 09 [89] and MILC 09A [6] this input has been replaced by $\epsilon =1.2(5)$, as suggested by phenomenological estimates for the corrections to Dashen’s theorem [108, 109]. Results of an evaluation of the electromagnetic self-energies based on $N_{ f}=2+1$ dynamical quarks in the QCD sector and on the quenched approximation in the QED sector have also been reported by MILC in Refs. [110–112] and updated recently in Refs. [113, 114]. Their latest (preliminary) result is $\bar{\epsilon }= 0.84(5)(19)$, where the first error is statistical and the second systematic, coming from discretization and finite-volume uncertainties added in quadrature. With the estimate for $\epsilon _m$ given in Eq. (13), this result corresponds to $\epsilon = 0.81(5)(18)$.

Preliminary results have also been reported by the BMW Collaboration in conference proceedings [115–117], with the updated result being $\epsilon = 0.57(6)(6)$, where the first error is statistical and the second systematic.

The RM123 Collaboration employs a new technique to compute e.m. shifts in hadron masses in 2-flavour QCD: the effects are included at leading order in the electromagnetic coupling $\alpha$ through simple insertions of the fundamental electromagnetic interaction in quark lines of relevant Feynman graphs [16]. They find $\epsilon =0.79(18)(18)$, where the first error is statistical and the second is the total systematic error resulting from chiral, finite-volume, discretization, quenching and fitting errors all added in quadrature.

Recently [118] the QCDSF/UKQCD Collaboration has presented results for several pseudoscalar meson masses obtained from $N_f = 2+1$ dynamical simulations of QCD + QED (at a single lattice spacing $a \simeq 0.07$ fm). Using the experimental values of the $\pi ^0$, $K^0$ and $K^+$ mesons masses to fix the three light-quark masses, they find $\epsilon = 0.50 (6)$, where the error is statistical only.

The effective Lagrangian that governs the self-energies to next-to-leading order (NLO) of the chiral expansion was set up in Ref. [119]. The estimates made in Refs. [108, 109] are obtained by replacing QCD with a model, matching this model with the effective theory and assuming that the effective coupling constants obtained in this way represent a decent approximation to those of QCD. For alternative model estimates and a detailed discussion of the problems encountered in models based on saturation by resonances, see Refs. [120–122]. In the present review of the information obtained on the lattice, we avoid the use of models altogether.

There is an indirect phenomenological determination of $\epsilon$, which is based on the decay $\eta \rightarrow 3\pi$ and does not rely on models. The result for the quark-mass ratio *Q*, defined in Eq. (32) and obtained from a dispersive analysis of this decay, implies $\epsilon = 0.70(28)$ (see Sect. 3.1.5). While the values found in older lattice calculations [103–105] are a little less than one standard deviation lower, the most recent determinations [16, 110–116, 123], though still preliminary, are in excellent agreement with this result and have significantly smaller error bars. However, even in the more recent calculations, e.m. effects are treated in the quenched approximation. Thus, we choose to quote $\epsilon = 0.7(3)$, which is essentially the $\eta \rightarrow 3\pi$ result and covers the range of post-2010 lattice results. Note that this value has an uncertainty which is reduced by about 40% compared to the result quoted in the first edition of the FLAG review [1].

We add a few comments concerning the physics of the self-energies and then specify the estimates used as an input in our analysis of the data. The Cottingham formula [124] represents the self-energy of a particle as an integral over electron scattering cross sections; elastic as well as inelastic reactions contribute. For the charged pion, the term due to elastic scattering, which involves the square of the e.m. form factor, makes a substantial contribution. In the case of the $\pi ^0$, this term is absent, because the form factor vanishes on account of charge conjugation invariance. Indeed, the contribution from the form factor to the self-energy of the $\pi ^+$ roughly reproduces the observed mass difference between the two particles. Furthermore, the numbers given in Refs. [125–127] indicate that the inelastic contributions are significantly smaller than the elastic contributions to the self-energy of the $\pi ^+$. The low-energy theorem of Das, Guralnik, Mathur, Low and Young [128] ensures that, in the limit $m_u,m_d\rightarrow 0$, the e.m. self-energy of the $\pi ^0$ vanishes, while the one of the $\pi ^+$ is given by an integral over the difference between the vector and axial-vector spectral functions. The estimates for $\epsilon _{\pi ^0}$ obtained in Ref. [104] and more recently in Ref. [118] are consistent with the suppression of the self-energy of the $\pi ^0$ implied by chiral $SU(2)\times SU(2)$. In our opinion, as already done in the FLAG 13 review [2], the value $\epsilon _{\pi ^0}=0.07(7)$ still represents a quite conservative estimate for this coefficient. The self-energy of the $K^0$ is suppressed less strongly, because it remains different from zero if $m_u$ and $m_d$ are taken massless and only disappears if $m_s$ is turned off as well. Note also that, since the e.m. form factor of the $K^0$ is different from zero, the self-energy of the $K^0$ does pick up an elastic contribution. The recent lattice result $\epsilon _{K^0} = 0.2(1)$ obtained in Ref. [118] indicates that the violation of Dashen’s theorem is smaller than in the case of $\epsilon$. Following the FLAG 13 review [2] we confirm the choice of the conservative value $\epsilon _{K^0} = 0.3(3)$.

Finally, we consider the mass splitting between the charged and neutral pions in QCD. This effect is known to be very small, because it is of second order in $m_u-m_d$. There is a parameter-free prediction, which expresses the difference $\hat{M}_{\pi ^+}^2-\hat{M}_{\pi ^0}^2$ in terms of the physical masses of the pseudoscalar octet and is valid to NLO of the chiral perturbation series. Numerically, the relation yields $\epsilon _m=0.04$ [129], indicating that this contribution does not play a significant role at the present level of accuracy. We attach a conservative error also to this coefficient: $\epsilon _m=0.04(2)$. The lattice result for the self-energy difference of the pions, reported in Ref. [103], $M_{\pi ^+}^\gamma -M_{\pi ^0}^\gamma = 4.50(23)\,\hbox {MeV}$, agrees with this estimate: expressed in terms of the coefficient $\epsilon _m$ that measures the pion-mass splitting in QCD, the result corresponds to $\epsilon _m=0.04(5)$. The corrections of next-to-next-to-leading order (NNLO) have been worked out in Ref. [130], but the numerical evaluation of the formulae again meets with the problem that the relevant effective coupling constants are not reliably known.

In summary, we use the following estimates for the e.m. corrections:

13 $$\begin{aligned}&\epsilon ={0.7(3)},\quad \epsilon _{\pi ^0}=0.07(7),\quad \epsilon _{K^0}=0.3(3),\nonumber \\&\quad \epsilon _m=0.04(2).\end{aligned}$$

While the range used for the coefficient $\epsilon$ affects our analysis in a significant way, the numerical values of the other coefficients only serve to set the scale of these contributions. The range given for $\epsilon _{\pi ^0}$ and $\epsilon _{K^0}$ may be overly generous, but because of the exploratory nature of the lattice determinations, we consider it advisable to use a conservative estimate.

Treating the uncertainties in the four coefficients as statistically independent and adding errors in quadrature, the numbers in Eq. (13) yield the following estimates for the e.m. self-energies,

14 $$\begin{aligned}&M_{\pi ^+}^\gamma = 4.7(3)~\hbox {MeV},\quad M_{\pi ^0}^\gamma = 0.3(3)~\hbox {MeV},\nonumber \\&\quad M_{\pi ^+}^\gamma -M_{\pi ^0}^\gamma =4.4(1)~\hbox {MeV},\nonumber \\&M_{K^+}^\gamma = 2.5(5)~\hbox {MeV},\quad M_{K^0}^\gamma =0.4(4)\,\hbox {MeV},\nonumber \\&\quad M_{K^+}^\gamma -M_{K^0}^\gamma = 2.1(4)~\hbox {MeV}, \end{aligned}$$

and for the pion and kaon masses occurring in the QCD sector of the Standard Model,

15 $$\begin{aligned}&\hat{M}_{\pi ^+}= 134.8(3)~\hbox {MeV},\quad \hat{M}_{\pi ^0} = 134.6(3)~\hbox {MeV} ,\nonumber \\&\quad \hat{M}_{\pi ^+}-\hat{M}_{\pi ^0}=0.2(1)~\hbox {MeV},\nonumber \\&\hat{M}_{K^+}=491.2(5)~\hbox {MeV},\quad \hat{M}_{K^0} =497.2(4)~\hbox {MeV},\nonumber \\&\quad \hat{M}_{K^+}-\hat{M}_{K^0}=-6.1(4)~\hbox {MeV}.\end{aligned}$$

The self-energy difference between the charged and neutral pion involves the same coefficient $\epsilon _m$ that describes the mass difference in QCD – this is why the estimate for $M_{\pi ^+}^\gamma -M_{\pi ^0}^\gamma$ is so precise.

#### Pion and kaon masses in the isospin limit

As mentioned above, most of the lattice calculations concerning the properties of the light mesons are performed in the isospin limit of QCD ($m_u-m_d\rightarrow 0$ at fixed $m_u+m_d$). We denote the pion and kaon masses in that limit by $\overline{M}_{\pi }$ and $\overline{M}_{K}$, respectively. Their numerical values can be estimated as follows. Since the operation $u\leftrightarrow d$ interchanges $\pi ^+$ with $\pi ^-$ and $K^+$ with $K^0$, the expansion of the quantities $\hat{M}_{\pi ^+}^2$ and $\frac{1}{2}(\hat{M}_{K^+}^2+\hat{M}_{K^0}^2)$ in powers of $m_u-m_d$ only contains even powers. As shown in Ref. [131], the effects generated by $m_u-m_d$ in the mass of the charged pion are strongly suppressed: the difference $\hat{M}_{\pi ^+}^2-\overline{M}_{\pi }^{\,2}$ represents a quantity of $\mathcal {O}[(m_u-m_d)^2(m_u+m_d)]$ and is therefore small compared to the difference $\hat{M}_{\pi ^+}^2-\hat{M}_{\pi ^0}^2$, for which an estimate was given above. In the case of $\frac{1}{2}(\hat{M}_{K^+}^2+\hat{M}_{K^0}^2)-\overline{M}_{K}^{\,2}$, the expansion does contain a contribution at NLO, determined by the combination $2L_8{-}L_5$ of low-energy constants, but the lattice results for that combination show that this contribution is very small, too. Numerically, the effects generated by $m_u-m_d$ in $\hat{M}_{\pi ^+}^2$ and in $\frac{1}{2}(\hat{M}_{K^+}^2+\hat{M}_{K^0}^2)$ are negligible compared to the uncertainties in the electromagnetic self-energies. The estimates for these given in Eq. (15) thus imply

16 $$\begin{aligned}&\overline{M}_{\pi }= \hat{M}_{\pi ^+}=134.8(3)\,\mathrm{MeV},\nonumber \\&\overline{M}_{K}= \sqrt{\frac{1}{2}(\hat{M}_{K^+}^2+\hat{M}_{K^0}^2)}= 494.2(3)\,\mathrm{MeV}. \end{aligned}$$

This shows that, for the convention used above to specify the QCD sector of the Standard Model, and within the accuracy to which this convention can currently be implemented, the mass of the pion in the isospin limit agrees with the physical mass of the neutral pion: $\overline{M}_{\pi }-M_{\pi ^0}=-0.2(3)$ MeV.

#### Lattice determination of $m_s$ and $m_{ud}$

We now turn to a review of the lattice calculations of the light-quark masses and begin with $m_s$, the isospin-averaged up- and down-quark mass, $m_{ud}$, and their ratio. Most groups quote only $m_{ud}$, not the individual up- and down-quark masses. We then discuss the ratio $m_u/m_d$ and the individual determination of $m_u$ and $m_d$.

Quark masses have been calculated on the lattice since the mid-1990s. However, early calculations were performed in the quenched approximation, leading to unquantifiable systematics. Thus in the following, we only review modern, unquenched calculations, which include the effects of light sea quarks.

Tables 3, 4 and 5 list the results of $N_{ f}=2$, $N_{ f}=2+1$ and $N_{ f}=2+1+1$ lattice calculations of $m_s$ and $m_{ud}$. These results are given in the ${\overline{\text {MS}}}$ scheme at $2\,\mathrm{GeV}$, which is standard nowadays, though some groups are starting to quote results at higher scales (e.g. Ref. [31]). The tables also show the colour coding of the calculations leading to these results. As indicated earlier in this review, we treat calculations with different numbers, $N_f$, of dynamical quarks separately.

$N_{ f}=2$ *lattice calculations* For $N_{ f}=2$, no new calculations have been performed since the previous edition of the FLAG review [2]. A quick inspection of Table 3 indicates that only the more recent calculations, ALPHA 12 [12] and ETM 10B [11], control all systematic effects – the special case of Dürr 11 [132] is discussed below. Only ALPHA 12 [12], ETM 10B [11] and ETM 07 [133] really enter the chiral regime, with pion masses down to about 270 MeV for ALPHA and ETM. Because this pion mass is still quite far from the physical-pion mass, ALPHA 12 refrain from determining $m_{ud}$ and give only $m_s$. All the other calculations have significantly more massive pions, the lightest being about 430 MeV, in the calculation by CP-PACS 01 [134]. Moreover, the latter calculation is performed on very coarse lattices, with lattice spacings $a\ge 0.11\,\mathrm{fm}$ and only one-loop perturbation theory is used to renormalize the results.

ETM 10B’s [11] calculation of $m_{ud}$ and $m_s$ is an update of the earlier twisted-mass determination of ETM 07 [133]. In particular, they have added ensembles with a larger volume and three new lattice spacings, $a = 0.054, 0.067$ and $0.098\,\mathrm{fm}$, allowing for a continuum extrapolation. In addition, it features analyses performed in *SU*(2) and *SU*(3) $\chi$PT.

The ALPHA 12 [12] calculation of $m_s$ is an update of ALPHA 05 [135], which pushes computations to finer lattices and much lighter pion masses. It also importantly includes a determination of the lattice spacing with the decay constant $F_K$, whereas ALPHA 05 converted results to physical units using the scale parameter $r_0$ [136], defined via the force between static quarks. In particular, the conversion relied on measurements of $r_0/a$ by QCDSF/UKQCD 04 [137] which differ significantly from the new determination by ALPHA 12. As in ALPHA 05, in ALPHA 12 both nonperturbative running and nonperturbative renormalization are performed in a controlled fashion, using Schrödinger functional methods.

**Table 3:** $N_{ f}=2$ lattice results for the masses $m_{ud}$ and $m_s$ (MeV, running masses in the ${\overline{\text {MS}}}$ scheme at scale 2 GeV). The significance of the colours is explained in Sect. 2. If information as regards nonperturbative running is available, this is indicated in the column “running”, with details given at the bottom of the table

Collaboration	Refs.	Publication status	Chiral extrapolation	Continuum extrapolation	Finite volume	Renormalization	Running	$$m_{ud} $$	$$m_s $$
ALPHA 12	[12]	A					$\,a,b$		102(3)(1)
Dürr 11$^{\mathrm{a}}$	[132]	A				−	−	3.52(10)(9)	97.0(2.6)(2.5)
ETM 10B	[11]	A					$\,c$	3.6(1)(2)	95(2)(6)
JLQCD/TWQCD 08A	[138]	A					−	4.452(81)(38)$$\left( {\begin{array}{c}+0\\ -227\end{array}}\right) $$	–
RBC 07$^\mathrm{b}$	[105]	A					−	4.25(23)(26)	119.5(5.6)(7.4)
ETM 07	[133]	A					−	3.85(12)(40)	105(3)(9)
QCDSF/UKQCD 06	[139]	A					−	4.08(23)(19)(23)	111(6)(4)(6)
SPQcdR 05	[140]	A					−	$4.3(4)(^{+1.1}_{-0.0})$	$101(8)(^{+25}_{-0})$
ALPHA 05	[135]	A					$\,a$		97(4)(18)$^{\mathrm{c}}$
QCDSF/UKQCD 04	[137]	A					−	4.7(2)(3)	119(5)(8)
JLQCD 02	[141]	A					−	$3.223(^{+46}_{-69})$	$84.5(^{+12.0}_{-1.7})$
CP-PACS 01	[134]	A					−	$3.45(10)(^{+11}_{-18})$	$89(2)(^{+2}_{-6})^{\mathrm{d}}$

*a* The masses are renormalized and run nonperturbatively up to a scale of $100\,\mathrm{GeV}$ in the $N_f=2$ SF scheme. In this scheme, nonperturbative and NLO running for the quark masses are shown to agree well from 100 GeV all the way down to 2 GeV [135]

*b* The running and renormalization results of Ref. [135] are improved in Ref. [12] with higher statistical and systematic accuracy

*c* The masses are renormalized nonperturbatively at scales $1/a\sim 2\div 3\,\mathrm{GeV}$ in the $N_f=2$ RI/MOM scheme. In this scheme, nonperturbative and N$^3$LO running for the quark masses are shown to agree from 4 GeV down to 2 GeV to better than 3% [142]

$^{\mathrm{a}}$ What is calculated is $m_c/m_s=11.27(30)(26)$. $m_s$ is then obtained using lattice and phenomenological determinations of $m_c$ which rely on perturbation theory. Finally, $m_{ud}$ is determined from $m_s$ using BMW 10A, 10B’s $N_f=2+1$ result for $m_s/m_{ud}$ [7, 8]. Since $m_c/m_s$ is renormalization group invariant in QCD, the renormalization and running of the quark masses enter indirectly through that of $m_c$, a mass that we do not review here

$^\mathrm{b}$ The calculation includes quenched e.m. effects

$^{\mathrm{c}}$ The data used to obtain the bare value of $m_s$ are from UKQCD/QCDSF 04 [137]

$^{\mathrm{d}}$ This value of $m_s$ was obtained using the kaon mass as input. If the $\phi$-meson mass is used instead, the authors find $m_s =90(^{+5}_{-11})$

**Table 4:** $N_{ f}=2+1$ lattice results for the masses $m_{ud}$ and $m_s$ (see Table 3 for notation)

Collaboration	Refs.	Publication status	Chiral extrapolation	Continuum extrapolation	Finite volume	Renormalization	Running	$$m_{ud} $$	$$m_s $$
RBC/UKQCD 14B$^{\mathrm{a}}$	[10]	P					*d*	3.31(4)(4)	90.3(0.9)(1.0)
RBC/UKQCD 12$^{\mathrm{a}}$	[31]	A					*d*	3.37(9)(7)(1)(2)	92.3(1.9)(0.9)(0.4)(0.8)
PACS-CS 12$^{\mathrm{b}}$	[143]	A					$\,b$	3.12(24)(8)	83.60(0.58)(2.23)
Laiho 11	[44]	C					−	3.31(7)(20)(17)	94.2(1.4)(3.2)(4.7)
BMW 10A, 10B$^{\mathrm{c}}$	[7, 8]	A					$\,c$	3.469(47)(48)	95.5(1.1)(1.5)
PACS-CS 10	[95]	A					$\,b$	2.78(27)	86.7(2.3)
MILC 10A	[13]	C					−	3.19(4)(5)(16)	–
HPQCD 10$^{\mathrm{d}}$	[9]	A				−	−	3.39(6)	92.2(1.3)
RBC/UKQCD 10A	[144]	A					$\,a$	3.59(13)(14)(8)	96.2(1.6)(0.2)(2.1)
Blum 10$^{\mathrm{e}}$	[103]	A					−	3.44(12)(22)	97.6(2.9)(5.5)
PACS-CS 09	[94]	A					$\,b$	2.97(28)(3)	92.75(58)(95)
HPQCD 09A$^{\mathrm{f}}$	[18]	A				−	−	3.40(7)	92.4(1.5)
MILC 09A	[6]	C					−	3.25 (1)(7)(16)(0)	89.0(0.2)(1.6)(4.5)(0.1)
MILC 09	[89]	A					−	3.2(0)(1)(2)(0)	88(0)(3)(4)(0)
PACS-CS 08	[93]	A					−	2.527(47)	72.72(78)
RBC/UKQCD 08	[145]	A					−	3.72(16)(33)(18)	107.3(4.4)(9.7)(4.9)
CP-PACS/JLQCD 07	[146]	A					−	$3.55(19)(^{+56}_{-20})$	$90.1(4.3)(^{+16.7}_{-4.3})$
HPQCD 05	[147]	A					−	$3.2(0)(2)(2)(0)^{\mathrm{g}}$	$87(0)(4)(4)(0)^{\mathrm{g}}$
MILC 04, HPQCD/MILC/UKQCD 04	[107, 148]	A					−	2.8(0)(1)(3)(0)	76(0)(3)(7)(0)

*a* The masses are renormalized nonperturbatively at a scale of 2 GeV in a couple of $N_f=3$ RI/SMOM schemes. A careful study of perturbative matching uncertainties has been performed by comparing results in the two schemes in the region of 2 GeV to 3 GeV [144]

*b* The masses are renormalized and run nonperturbatively up to a scale of $40\,\mathrm{GeV}$ in the $N_f=3$ SF scheme. In this scheme, nonperturbative and NLO running for the quark masses are shown to agree well from 40 GeV all the way down to 3 GeV [95]

*c* The masses are renormalized and run nonperturbatively up to a scale of 4 GeV in the $N_f=3$ RI/MOM scheme. In this scheme, nonperturbative and N$^3$LO running for the quark masses are shown to agree from 6 GeV down to 3 GeV to better than 1% [8]

*d* All required running is performed nonperturbatively

$^{\mathrm{a}}$ The results are given in the ${\overline{\text {MS}}}$ scheme at 3 instead of 2 GeV. We run them down to 2 GeV using numerically integrated 4-loop running [149, 150] with $N_f=3$ and with the values of $\alpha _s(M_Z)$, $m_b$ and $m_c$ taken from Ref. [151]. The running factor is 1.106. At three loops it is only 0.2% smaller, indicating that running uncertainties are small. We neglect them here

$^{\mathrm{b}}$ The calculation includes e.m. and $m_u\ne m_d$ effects through reweighting

$^{\mathrm{c}}$ The fermion action used is tree-level improved

$^{\mathrm{d}}$ What is calculated is then obtained by combining this result with HPQCD 09A’s $m_c/m_s=11.85(16)$ [18]. Finally, $m_{ud}$ is determined from $m_s$ with the MILC 09 result for $m_s/m_{ud}$. Since $m_c/m_s$ is renormalization group invariant in QCD, the renormalization and running of the quark masses enter indirectly through that of $m_c$ (see below)

$^{\mathrm{e}}$ The calculation includes quenched e.m. effects

$^{\mathrm{f}}$ What is calculated is $m_c/m_s=11.85(16)$. $m_s$ is then obtained by combing this result with the determination $m_c(m_c) = 1.268(9)$ GeV from Ref. [152]. Finally, $m_{ud}$ is determined from $m_s$ with the MILC 09 result for $m_s/m_{ud}$

$^{\mathrm{g}}$ The bare numbers are those of MILC 04. The masses are simply rescaled, using the ratio of the two-loop to one-loop renormalization factors

**Table 5:** $N_{ f}=2+1+1$ lattice results for the masses $m_{ud}$ and $m_s$ (see Table 3 for notation)

Collaboration	Refs.	Publication status	Chiral extrapolation	Continuum extrapolation	Finite volume	Renormalization	Running	$$m_{ud} $$	$$m_s $$
HPQCD 14A$^{\mathrm{a}}$	[5]	A				−	−		93.7(8)
ETM 14$^{\mathrm{a}}$	[4]	A					−	3.70(13)(11)	99.6(3.6)(2.3)

$^{\mathrm{a}}$ As explained in the text, $m_s$ is obtained by combining the results $m_c(5~\mathrm{GeV};N_f=4)=0.8905(56)$ GeV and $(m_c/m_s)(N_f=4)=11.652(65)$, determined on the same dataset. A subsequent scale and scheme conversion, performed by the authors leads, to the value 93.6(8). In the table we have converted this to $m_s(2~\mathrm{GeV};N_f=4)$, which makes a very small change

The conclusion of our analysis of $N_{ f}=2$ calculations is that the results of ALPHA 12 [12] and ETM 10B [11] (which update and extend ALPHA 05 [135] and ETM 07 [133], respectively), are the only ones to date which satisfy our selection criteria. Thus we average those two results for $m_s$, obtaining 101(3) MeV. Regarding $m_{ud}$, for which only ETM 10B [11] gives a value, we do not offer an average but simply quote ETM’s number. Thus, we quote as our estimates:

17 $$\begin{aligned}&m_s&= 101(3)~\hbox {MeV}&\,\mathrm {Refs.}~ [11, 12],\nonumber \\&N_{ f}=2 :&\\&m_{ud}&= 3.6(2) ~\hbox {MeV}&\,\mathrm {Ref.}~[11].\nonumber \end{aligned}$$

The errors on these results are 3 and 6%, respectively. However, these errors do not account for the fact that sea strange-quark mass effects are absent from the calculation, a truncation of the theory whose systematic effects cannot be estimated a priori. Thus, these results carry an additional unknown systematic error. It is worth remarking that the difference between ALPHA 12’s [12] central value for $m_s$ and that of ETM 10B [11] is 7(7) MeV.

We have not included the results of Dürr 11 [132] in the averages of Eq. (17), despite the fact that they satisfy our selection criteria. The reason for this is that the observable which they actually compute on the lattice is $m_c/m_s=11.27(30)(26)$, reviewed in Sect. 3.2.4. They obtain $m_s$ by combining that value of $m_c/m_s$ with already existing phenomenological calculations of $m_c$. Subsequently they obtain $m_{ud}$ by combining this result for $m_s$ with the $N_f=2+1$ calculation of $m_s/m_{ud}$ of BMW 10A, 10B [7, 8] discussed below. Thus, their results for $m_s$ and $m_{ud}$ are not per se lattice results, nor do they correspond to $N_f=2$. The value of the charm-quark mass which they use is an average of phenomenological determinations, which they estimate to be $m_c(2\,\mathrm{GeV})=1.093(13)\,\mathrm{GeV}$, with a 1.2% total uncertainty. This value for $m_c$ leads to the results for $m_s$ and $m_{ud}$ in Table 3 which are compatible with the averages given in Eq. (17) and have similar uncertainties. Note, however, that their determination of $m_c/m_s$ is about 1.5 combined standard deviations below the only other $N_{ f}=2$ result which satisfies our selection criteria, ETM 10B’s [11] result, as discussed in Sect. 3.2.4.

$N_{ f}=2+1$ *lattice calculations* We turn now to $N_{ f}=2+1$ calculations. These and the corresponding results for $m_{ud}$ and $m_s$ are summarized in Table 4. Given the very high precision of a number of the results, with total errors on the order of 1%, it is important to consider the effects neglected in these calculations. Since isospin-breaking and e.m. effects are small on $m_{ud}$ and $m_s$, and have been approximately accounted for in the calculations that will be retained for our averages, the largest potential source of uncontrolled systematic error is that due to the omission of the charm quark in the sea. Beyond the small perturbative corrections that come from matching the $N_{ f}=3$ to the $N_{ f}=4$ ${\overline{\text {MS}}}$ scheme at $m_c$ (${\sim } -0.2\%$), the charm sea-quarks affect the determination of the light-quark masses through contributions of order $1/m_c^2$. As these are further suppressed by the Okubo–Zweig–Iizuka rule, they are also expected to be small, but are difficult to quantify a priori. Fortunately, as we will see below, $m_s$ has been directly computed with $N_f=2+1+1$ simulations. In particular, HPQCD 14 [5] has computed $m_s$ in QCD$_4$ with very much the same approach as it had used to obtain the QCD$_3$ result of HPQCD 10 [9]. Their results for $m_s(N_f=3, 2~\mathrm{GeV})$ are $93.8(8)\,\mathrm{MeV}$ [5] and $92.2(1.3)\,\mathrm{MeV}$ [9], where the $N_f=4$ result has been converted perturbatively to $N_f=3$ in Ref. [5]. This leads to a relative difference of $1.7(1.6)\%$. While the two results are compatible within one combined standard deviation, a ${\sim } 2\%$ effect cannot be excluded. Thus, we will retain this 2% uncertainty and add it to the averages for $m_s$ and $m_{ud}$ given below.

The only new calculation since the last FLAG report [2] is that of RBC/UKQCD 14 [10]. It significantly improves on their RBC/UKQCD 12 [31] work by adding three new domain-wall fermion simulations to three used previously. Two of the new simulations are performed at essentially physical-pion masses ($M_\pi \simeq 139\,\mathrm{MeV}$) on lattices of about $5.4\,\mathrm{fm}$ in size and with lattice spacings of $0.114\,\mathrm{fm}$ and $0.084\,\mathrm{fm}$. It is complemented by a third simulation with $M_\pi \simeq 371\,\mathrm{MeV}$, $a\simeq 0.063$ and a rather small $L\simeq 2.0\,\mathrm{fm}$. Altogether, this gives them six simulations with six unitary $M_\pi$’s in the range of 139 to $371\,\mathrm{MeV}$ and effectively three lattice spacings from 0.063 to $0.114\,\mathrm{fm}$. They perform a combined global continuum and chiral fit to all of their results for the $\pi$ and *K* masses and decay constants, the $\Omega$ baryon mass and two Wilson-flow parameters. Quark masses in these fits are renormalized and run nonperturbatively in the RI/SMOM scheme. This is done by computing the relevant renormalization constant for a reference ensemble and determining those for other simulations relative to it by adding appropriate parameters in the global fit. This new calculation passes all of our selection criteria. Its results will replace the older RBC/UKQCD 12 results in our averages.

$N_{ f}=2+1$ MILC results for light-quark masses go back to 2004 [107, 148]. They use rooted staggered fermions. By 2009 their simulations covered an impressive range of parameter space, with lattice spacings which go down to 0.045 fm and valence-pion masses down to approximately 180 MeV [6]. The most recent MILC $N_{ f}=2+1$ results, i.e. MILC 10A [13] and MILC 09A [6], feature large statistics and two-loop renormalization. Since these datasets subsume those of their previous calculations, these latest results are the only ones that must be kept in any world average.

The PACS-CS 12 [143] calculation represents an important extension of the collaboration’s earlier 2010 computation [95], which already probed pion masses down to $M_\pi \simeq 135\,\mathrm{MeV}$, i.e. down to the physical-mass point. This was achieved by reweighting the simulations performed in PACS-CS 08 [93] at $M_\pi \simeq 160\,\mathrm{MeV}$. If adequately controlled, this procedure eliminates the need to extrapolate to the physical-mass point and, hence, the corresponding systematic error. The new calculation now applies similar reweighting techniques to include electromagnetic and $m_u\ne m_d$ isospin-breaking effects directly at the physical-pion mass. Further, as in PACS-CS 10 [95], renormalization of quark masses is implemented nonperturbatively, through the Schrödinger functional method [153]. As it stands, the main drawback of the calculation, which makes the inclusion of its results in a world average of lattice results inappropriate at this stage, is that for the lightest quark mass the volume is very small, corresponding to $LM_\pi \simeq 2.0$, a value for which finite-volume effects will be difficult to control. Another problem is that the calculation was performed at a single lattice spacing, forbidding a continuum extrapolation. Further, it is unclear at this point what might be the systematic errors associated with the reweighting procedure.

The BMW 10A, 10B [7, 8] calculation still satisfies our stricter selection criteria. They reach the physical up- and down-quark mass by *interpolation* instead of by extrapolation. Moreover, their calculation was performed at five lattice spacings ranging from 0.054 to 0.116 fm, with full nonperturbative renormalization and running and in volumes of up to (6 fm)$^3$ guaranteeing that the continuum limit, renormalization and infinite-volume extrapolation are controlled. It does neglect, however, isospin-breaking effects, which are small on the scale of their error bars.

Finally we come to another calculation which satisfies our selection criteria, HPQCD 10 [9]. It updates the staggered fermions calculation of HPQCD 09A [18]. In these papers the renormalized mass of the strange quark is obtained by combining the result of a precise calculation of the renormalized charm-quark mass, $m_c$, with the result of a calculation of the quark-mass ratio, $m_c/m_s$. As described in Ref. [152] and in Sect. 3.2, HPQCD determines $m_c$ by fitting Euclidean-time moments of the $\bar{c}c$ pseudoscalar density 2-point functions, obtained numerically in lattice QCD, to fourth-order, continuum perturbative expressions. These moments are normalized and chosen so as to require no renormalization with staggered fermions. Since $m_c/m_s$ requires no renormalization either, HPQCD’s approach displaces the problem of lattice renormalization in the computation of $m_s$ to one of computing continuum perturbative expressions for the moments. To calculate $m_{ud}$ HPQCD 10 [9] use the MILC 09 determination of the quark-mass ratio $m_s/m_{ud}$ [89].

HPQCD 09A [18] obtains $m_c/m_s=11.85(16)$ [18] fully nonperturbatively, with a precision slightly larger than 1%. HPQCD 10’s determination of the charm-quark mass, $m_c(m_c)=1.268(6)$,^13^ is even more precise, achieving an accuracy better than 0.5%. While these errors are, perhaps, surprisingly small, we take them at face value as we do those of RBC/UKQCD 14, since we will add a 2% error due to the quenching of the charm on the final result.

This discussion leaves us with four results for our final average for $m_s$: MILC 09A [6], BMW 10A, 10B [7, 8], HPQCD 10 [9] and RBC/UKQCD 14 [10]. Assuming that the result from HPQCD 10 is 100% correlated with that of MILC 09A, as it is based on a subset of the MILC 09A configurations, we find $m_s\!=\!92.0(1.1)\,\mathrm{MeV}$ with a $\chi ^2/\hbox {d.o.f.}\! =\! 1.8.$

For the light-quark mass $m_{ud}$, the results satisfying our criteria are RBC/UKQCD 14B, BMW 10A, 10B, HPQCD 10, and MILC 10A. For the error, we include the same 100% correlation between statistical errors for the latter two as for the strange case, resulting in $m_{ud}=3.373(43)$ at 2 GeV in the $\overline{\mathrm{MS}}$ scheme $(\chi ^2/\hbox {d.o.f.}=1.5).$ Adding the 2% estimate for the missing charm contribution, our final estimates for the light-quark masses are

18 $$\begin{aligned}&m_{ud}&= 3.373 (80)\;\mathrm{MeV}&\,\mathrm {Refs.}~ [7{-}10, 13],\nonumber \\&N_{ f}=2+1 :&\nonumber \\&m_s&=92.0(2.1)\;\;\mathrm{MeV}&\,\mathrm {Refs.}~ [6{-}10]. \nonumber \\ \end{aligned}$$

$N_{ f}=2+1+1$ *lattice calculations* One of the novelties since the last edition of this review [2] is the fact that $N_f=2+1+1$ results for the light-quark masses have been published. These and the features of the corresponding calculations are summarized in Table 5. Note that the results of Ref. [5] are reported as $m_s(2\,\mathrm{GeV};N_f=3)$ and those of Ref. [4] as $m_{ud(s)}(2\,\mathrm{GeV};N_f=4)$. We convert the former to $N_f=4$ and obtain $m_s(2\,\mathrm{GeV};N_f=4)=93.7(8)~\mathrm{MeV}$. The average of ETM 14 and HPQCD 14A is 93.9(1.1) $\mathrm{MeV}$ with $\chi ^2/\hbox {d.o.f.}=1.8.$ For the light0quark average we use the sole available value from ETM 14A. Our averages are

19 $$\begin{aligned}&m_{ud}&= 3.70 (17)\;\mathrm{MeV}&\,\mathrm {Ref.}~[4],\nonumber \\&N_{ f}=2+1+1{:}&\nonumber \\&m_s&=93.9(1.1)\; \mathrm{MeV}&\,\mathrm {Refs.}~ [4,5].\nonumber \\ \end{aligned}$$

**Fig. 1:**
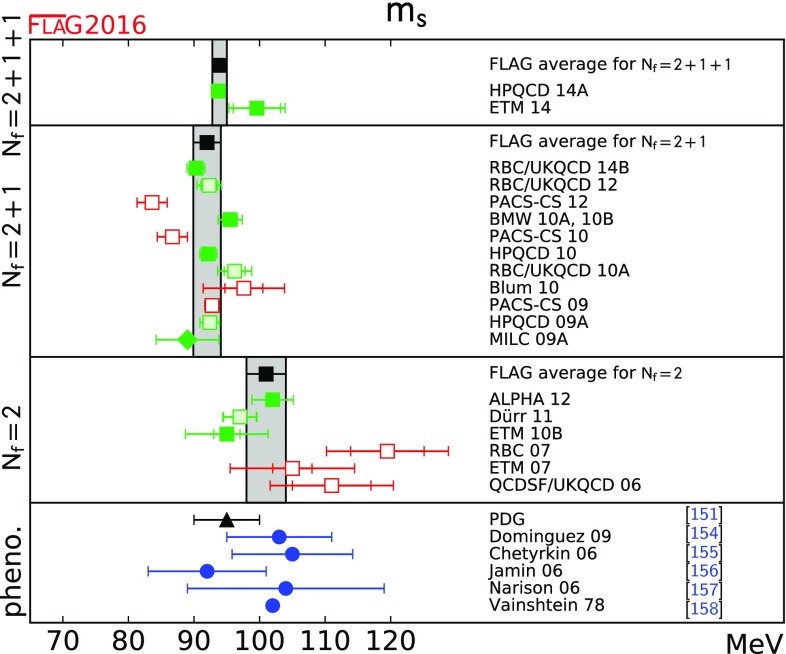
${\overline{\text {MS}}}$ mass of the strange quark (at 2 GeV scale) in MeV. The *upper three panels* show the lattice results listed in Tables 3, 4 and 5, while the bottom panel collects a few sum rule results and also indicates the current PDG estimate. *Diamonds and squares* represent results based on perturbative and nonperturbative renormalization, respectively. The *black squares* and the *grey bands* represent our estimates (17), (18) and (19). The significance of the colours is explained in Sect. 2

**Fig. 2:**
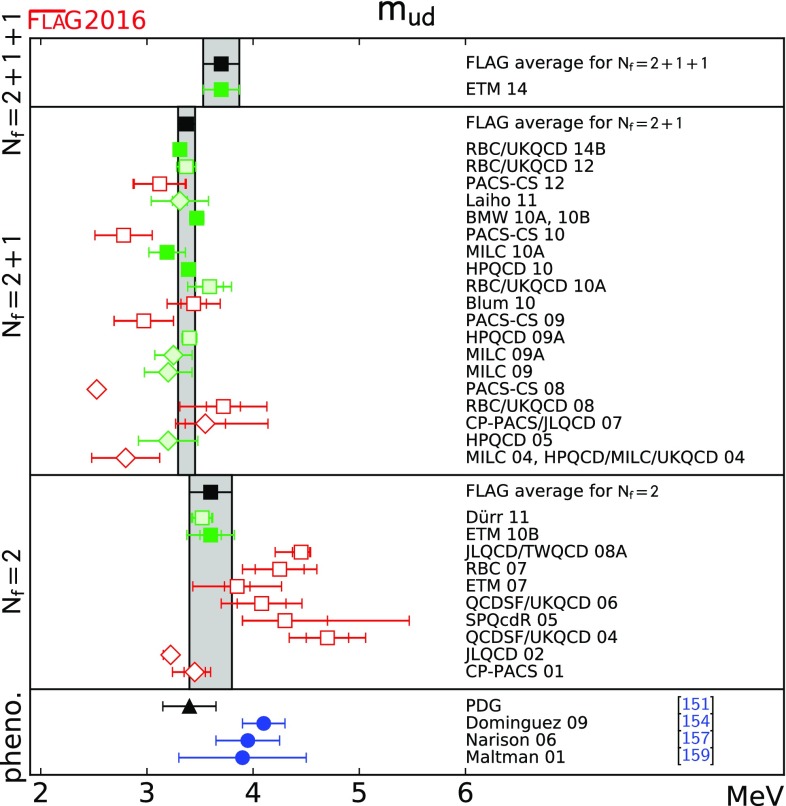
Mean mass of the two lightest quarks, $m_{ud}=\frac{1}{2}(m_u+m_d)$ (for details see Fig. 1)

In Figs. 1 and 2 the lattice results listed in Tables 3, 4 and 5 and the FLAG averages obtained at each value of $N_f$ are presented and compared with various phenomenological results.

#### Lattice determinations of $m_s/m_{ud}$

**Table 6:** Lattice results for the ratio $m_s/m_{ud}$

Collaboration	Refs.	$N_{ f}$	Publication status	Chiral extrapolation	Continuum extrapolation	Finite volume	$m_s/m_{ud}$
FNAL/MILC 14A	[14]	$2+1+1$	A				$27.35(5)^{+10}_{-7}$
ETM 14	[4]	$2+1+1$	A				26.66(32)(2)
RBC/UKQCD 14B	[10]	$2+1$	P				27.34(21)
RBC/UKQCD 12$^{\mathrm{a}}$	[31]	$2+1$	A				27.36(39)(31)(22)
PACS-CS 12$^{\mathrm{b}}$	[143]	$2+1$	A				26.8(2.0)
Laiho 11	[44]	$2+1$	C				28.4(0.5)(1.3)
BMW 10A, 10B$^{\mathrm{c}}$	[7, 8]	$2+1$	A				27.53(20)(8)
RBC/UKQCD 10A	[144]	$2+1$	A				26.8(0.8)(1.1)
Blum 10$^{\mathrm{d}}$	[103]	$2+1$	A				28.31(0.29)(1.77)
PACS-CS 09	[94]	$2+1$	A				31.2(2.7)
MILC 09A	[6]	$2+1$	C				27.41(5)(22)(0)(4)
MILC 09	[89]	$2+1$	A				27.2(1)(3)(0)(0)
PACS-CS 08	[93]	$2+1$	A				28.8(4)
RBC/UKQCD 08	[145]	$2+1$	A				28.8(0.4)(1.6)
MILC 04, HPQCD/MILC/UKQCD 04	[107, 148]	$2+1$	A				27.4(1)(4)(0)(1)
ETM 14D	[160]	2	C				27.63(13)
ETM 10B	[11]	2	A				27.3(5)(7)
RBC 07$^{\mathrm{d}}$	[105]	2	A				28.10(38)
ETM 07	[133]	2	A				27.3(0.3)(1.2)
QCDSF/UKQCD 06	[139]	2	A				27.2(3.2)

$^{\mathrm{a}}$ The errors are statistical, chiral and finite volume

$^{\mathrm{b}}$ The calculation includes e.m. and $m_u\ne m_d$ effects through reweighting

$^{\mathrm{c}}$ The fermion action used is tree-level improved

$^{\mathrm{d}}$ The calculation includes quenched e.m. effects

The lattice results for $m_s/m_{ud}$ are summarized in Table 6. In the ratio $m_s/m_{ud}$, one of the sources of systematic error – the uncertainties in the renormalization factors – drops out. Also, we can compare the lattice results with the leading-order formula of $\chi$PT,

20 $$\begin{aligned} \frac{m_s}{m_{ud}}\mathop {=}\limits ^{{\mathrm{LO}}}\frac{\hat{M}_{K^+}^2+ \hat{M}_{K^0}^2-\hat{M}_{\pi ^+}^2}{\hat{M}_{\pi ^+}^2},\end{aligned}$$

which relates the quantity $m_s/m_{ud}$ to a ratio of meson masses in QCD. Expressing these in terms of the physical masses and the four coefficients introduced in Eqs. (10)–(12), linearizing the result with respect to the corrections and inserting the observed mass values, we obtain

21 $$\begin{aligned} \frac{m_s}{m_{ud}} \mathop {=}\limits ^{{\mathrm{LO}}}25.9 - 0.1\, \epsilon + 1.9\, \epsilon _{\pi ^0} - 0.1\, \epsilon _{K^0} -1.8 \,\epsilon _m.\end{aligned}$$

If the coefficients $\epsilon$, $\epsilon _{\pi ^0}$, $\epsilon _{K^0}$ and $\epsilon _m$ are set equal to zero, the right hand side reduces to the value $m_s/m_{ud}=25.9$, which follows from Weinberg’s leading-order formulae for $m_u/m_d$ and $m_s/m_d$ [161], in accordance with the fact that these do account for the e.m. interaction at leading chiral order, and neglect the mass difference between the charged and neutral pions in QCD. Inserting the estimates (13) gives the effect of chiral corrections to the e.m. self-energies and of the mass difference between the charged and neutral pions in QCD. With these, the LO prediction in QCD becomes

22 $$\begin{aligned} \frac{m_s}{m_{ud}}\mathop {=}\limits ^{{\mathrm{LO}}}25.9(1), \end{aligned}$$

leaving the central value unchanged at 25.9. The corrections parameterized by the coefficients of Eq. (13) are small for this quantity. Note that the quoted uncertainty does not include an estimate of higher-order chiral contributions to this LO QCD formula, but only accounts for the error bars in the coefficients. However, even this small uncertainty is no longer irrelevant given the high precision reached in lattice determinations of the ratio $m_s/m_{ud}$.

The lattice results in Table 6, which satisfy our selection criteria, indicate that the corrections generated by the nonleading terms of the chiral perturbation series are remarkably small, in the range 3–10%. Despite the fact that the *SU*(3)-flavour-symmetry-breaking effects in the Nambu–Goldstone boson masses are very large ($M_K^2\simeq 13\, M_\pi ^2$), the mass spectrum of the pseudoscalar octet obeys the $SU(3)\times SU(3)$ Eq. (20) very well.

$N_{ f}=2$ *lattice calculations* With respect to the FLAG 13 review [2] there is only one new result, ETM 14D [160], based on recent ETM gauge ensembles generated close to the physical point with the addition of a clover term to the tmQCD action. The new simulations are performed at a single lattice spacing of ${\simeq } 0.09$ fm and at a single box size $L \simeq 4$ fm and therefore their calculations do not pass our criteria for the continuum extrapolation and finite-volume effects.

Therefore the FLAG average at $N_f = 2$ is still obtained by considering only the ETM 10B result (described already in the previous section), namely

23 $$\begin{aligned} N_f = 2:\quad m_s / m_{ud} = 27.3 ~ (9)\quad \,\mathrm {Ref.}~[11], \end{aligned}$$

with an overall uncertainty equal to 3.3%.

$N_{ f}=2+1$ *lattice calculations* For $N_f = 2+1$ our average of $m_s/m_{ud}$ is based on the new result RBC/UKQCD 14B, which replaces RBC/UKQCD 12 (see Sect. 3.1.3), and on the results MILC 09A and BMW 10A, 10B. The value quoted by HPQCD 10 does not represent independent information as it relies on the result for $m_s/m_{ud}$ obtained by the MILC Collaboration. Averaging these results according to the prescriptions of Sect. 2.3 gives $m_s / m_{ud} = 27.43(13)$ with $\chi ^2/\hbox {d.o.f.} \simeq 0.2$. Since the errors associated with renormalization drop out in the ratio, the uncertainties are even smaller than in the case of the quark masses themselves: the above number for $m_s/m_{ud}$ amounts to an accuracy of 0.5%.

At this level of precision, the uncertainties in the electromagnetic and strong isospin-breaking corrections are not completely negligible. The error estimate in the LO result (22) indicates the expected order of magnitude. In view of this, we ascribe conservatively a 1.0% uncertainty to this source of error. Thus, our final conservative estimate is

24 $$\begin{aligned}&N_f = 2+1 : \quad \nonumber \\&{m_s}/{m_{ud}} = 27.43 ~ (13) ~ (27) = 27.43 ~ (31) \,\mathrm {Refs.}~[6{-}8, 10],\quad \end{aligned}$$

with a total 1.1% uncertainty. It is also fully consistent with the ratio computed from our individual quark masses in Eq. (18), $m_s / m_{ud} = 27.6(6)$, which has a larger 2.2% uncertainty. In Eq. (24) the first error comes from the averaging of the lattice results, and the second is the one that we add to account for the neglect of isospin-breaking effects.

$N_{ f}=2+1+1$ *lattice calculations* For $N_f = 2+1+1$ there are two results, ETM 14 [4] and FNAL/MILC 14A [14], both of which satisfy our selection criteria.

ETM 14 uses 15 twisted-mass gauge ensembles at three lattice spacings ranging from 0.062 to 0.089 fm (using $f_\pi$ as input), in boxes of size ranging from 2.0 to 3.0 fm and pion masses from 210 to 440 MeV (explaining the tag  in the chiral extrapolation and the tag  for the continuum extrapolation). The value of $M_\pi L$ at their smallest pion mass is 3.2 with more than two volumes (explaining the tag  in the finite-volume effects). They fix the strange mass with the kaon mass.

FNAL/MILC 14A employs HISQ staggered fermions. Their result is based on 21 ensembles at 4 values of the coupling $\beta$ corresponding to lattice spacings in the range from 0.057 to 0.153 fm, in boxes of sizes up to 5.8 fm and with taste-Goldstone pion masses down to 130 MeV and RMS pion masses down to 143 MeV. They fix the strange mass with $M_{\bar{s}s}$, corrected for e.m. effects with $\bar{\epsilon }= 0.84(20)$ [113]. All of our selection criteria are satisfied with the tag  . Thus our average is given by $m_s / m_{ud} = 27.30 ~ (20)$, where the error includes a large stretching factor equal to $\sqrt{\chi ^2/\hbox {d.o.f.}} \simeq 2.1$, coming from our rules for the averages discussed in Sect. 2.2. Nevertheless the above number amounts still to an accuracy of 0.7%. As in the case of our average for $N_f = 2+1$, we add a 1.0% uncertainty related to the neglect of isospin-breaking effects, leading to

25 $$\begin{aligned} N_f= & {} 2+1+1 :\quad m_s / m_{ud} = 27.30 ~ (20) ~ (27) \nonumber \\= & {} 27.30 ~ (34)\,\mathrm {Refs.}~[4, 14], \end{aligned}$$

which corresponds to an overall uncertainty equal to 1.3%.

**Fig. 3:**
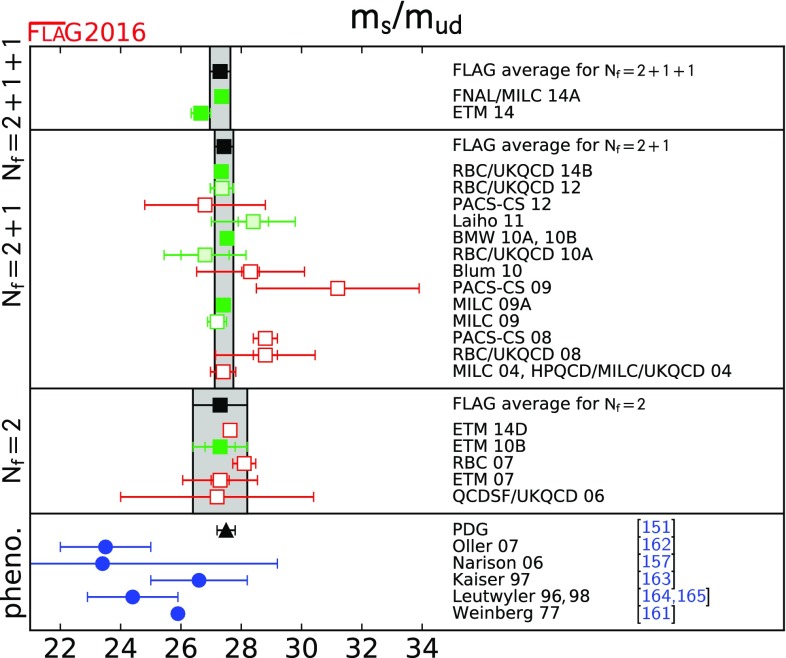
Results for the ratio $m_s/m_{ud}$. The *upper part* indicates the lattice results listed in Table 6 together with the FLAG averages for each value of $N_f$. The *lower part* shows results obtained from $\chi$PT and sum rules, together with the current PDG estimate

All the lattice results listed in Table 6 as well as the FLAG averages for each value of $N_f$ are reported in Fig. 3 and compared with $\chi$PT, sum rules and the updated PDG estimate $m_s / m_{ud} = 27.5(3)$ [151].

Note that our averages (23), (24) and (25), obtained for $N_f = 2$, $2+1$ and $2+1+1$, respectively, agree very well within the quoted errors. They also show that the LO prediction of $\chi$PT in Eq. (22) receives only small corrections from higher orders of the chiral expansion: according to Eqs. (24) and (25), these generate shifts of 5.9(1.1) and $5.4(1.2) \%$ relative to Eq. (22), respectively.

The ratio $m_s/m_{ud}$ can also be extracted from the masses of the neutral Nambu–Goldstone bosons: neglecting effects of order $(m_u-m_d)^2$ also here, the leading-order formula reads $m_s / m_{ud} \mathop {=}\limits ^{{\mathrm{LO}}}\frac{3}{2} \hat{M}_\eta ^2 / \hat{M}_\pi ^2 - \frac{1}{2}$. Numerically, this gives $m_s / m_{ud} \mathop {=}\limits ^{{\mathrm{LO}}}24.2$. The relation has the advantage that the e.m. corrections are expected to be much smaller here, but it is more difficult to calculate the $\eta$-mass on the lattice. The comparison with Eqs. (24) and (25) shows that, in this case, the NLO contributions are somewhat larger: 11.9(9) and $11.4( 1.1) \%$.

#### Lattice determination of $m_u$ and $m_d$

Since FLAG 13, two new results have been reported for nondegenerate light-quark masses, ETM 14 [4], and QCDSF/UKQCD 15 [166], for $N_f=2+1+1$, and 3 flavours respectively. The former uses simulations in pure QCD, but determines $m_u-m_d$ from the slope of the square of the kaon mass and the neutral-charged mass-squares difference, evaluated at the isospin-symmetric point. The latter uses QCD+QED dynamical simulations performed at the *SU*(3)-flavour-symmetric point, but at a single lattice spacing, so they do not enter our average. While QCDSF/UKQCD 15 use three volumes, the smallest has linear size roughly 1.7 fm, and the smallest partially quenched pion mass is greater than 200 MeV, so our finite-volume and chiral-extrapolation criteria require  ratings. In Ref. [166] results for $\epsilon$ and $m_{u}/m_{d}$ are computed in the so-called Dashen scheme. A subsequent paper [118] gives formulae to convert the $\epsilon$ parameters to the $\overline{\mathrm{MS}}$ scheme.

As the above implies, the determination of $m_u$ and $m_d$ separately requires additional input. MILC 09A [6] uses the mass difference between $K^0$ and $K^+$, from which they subtract electromagnetic effects using Dashen’s theorem with corrections, as discussed in Sect. 3.1.1. The up and down sea quarks remain degenerate in their calculation, fixed to the value of $m_{ud}$ obtained from $M_{\pi ^0}$.

To determine $m_u/m_d$, BMW 10A, 10B [7, 8] follow a slightly different strategy. They obtain this ratio from their result for $m_s/m_{ud}$ combined with a phenomenological determination of the isospin-breaking quark-mass ratio $Q=22.3(8)$, defined below in Eq. (32), from $\eta \rightarrow 3\pi$ decays [101] (the decay $\eta \rightarrow 3\pi$ is very sensitive to QCD isospin breaking but fairly insensitive to QED isospin breaking). As discussed in Sect. 3.1.6, the central value of the e.m. parameter $\epsilon$ in Eq. (13) is taken from the same source.

RM123 11 [167] actually uses the e.m. parameter $\epsilon =0.7(5)$ from the first edition of the FLAG review [1]. However, they estimate the effects of strong isospin breaking at first nontrivial order, by inserting the operator $\frac{1}{2}(m_u-m_d)\int (\bar{u}u-\bar{d}d)$ into correlation functions, while performing the gauge averages in the isospin limit. Applying these techniques, they obtain $(\hat{M}_{K^0}^2-\hat{M}_{K^+}^2)/(m_d-m_u)=2.57(8)\,\mathrm{MeV}$. Combining this result with the phenomenological $(\hat{M}_{K^0}^2-\hat{M}_{K^+}^2)=6.05(63)\times 10^3$ determined with the above value of $\epsilon$, they get $(m_d-m_u)=2.35(8)(24)\,\mathrm{MeV}$, where the first error corresponds to the lattice statistical and systematic uncertainties combined in quadrature, while the second arises from the uncertainty on $\epsilon$. Note that below we quote results from RM123 11 for $m_u$, $m_d$ and $m_u/m_d$. As described in Table 7, we obtain them by combining RM123 11’s result for $(m_d-m_u)$ with ETM 10B’s result for $m_{ud}$.

**Table 7:** Lattice results for $$m_u$$, $$m_d$$ (MeV) and for the ratio $$m_u/m_d$$. The values refer to the $${\overline{\text {MS}}}$$ scheme at scale 2 GeV. The top part of the table lists the result obtained with $$N_{ f}=2+1+1$$, while the middle and lower part presents calculations with $$N_f = 2+1 $$ and $$N_f = 2$$, respectively

Collaboration	Refs.	Publication status	Chiral extrapolation	Continuum extrapolation	Finite volume	Renormalization	Running	$m_u$	$m_d$	$m_u/m_d$
MILC 14	[113]	C				−	−			$0.4482(48)({}^{+\phantom {0}21}_{-115})(1)(165)$
ETM 14	[4]	A					$\,b$	2.36(24)	5.03(26)	0.470(56)
QCDSF/UKQCD 15$^{\mathrm{a}}$	[166]	P				−	−			0.52(5)
PACS-CS 12$^{\mathrm{b}}$	[143]	A					$\,a$	2.57(26)(7)	3.68(29)(10)	0.698(51)
Laiho 11	[44]	C					−	1.90(8)(21)(10)	4.73(9)(27)(24)	0.401(13)(45)
HPQCD 10$^{\mathrm{c}}$	[9]	A					−	2.01(14)	4.77(15)	
BMW 10A, 10B$^{\mathrm{d}}$	[7, 8]	A					$\,b$	2.15(03)(10)	4.79(07)(12)	0.448(06)(29)
Blum 10$^{\mathrm{g}}$	[103]	A					−	2.24(10)(34)	4.65(15)(32)	0.4818(96)(860)
MILC 09A	[6]	C					−	1.96(0)(6)(10)(12)	4.53(1)(8)(23)(12)	0.432(1)(9)(0)(39)
MILC 09	[89]	A					−	1.9(0)(1)(1)(1)	4.6(0)(2)(2)(1)	0.42(0)(1)(0)(4)
MILC 04, HPQCD/ MILC/UKQCD 04	[107] [148]	A					−	1.7(0)(1)(2)(2)	3.9(0)(1)(4)(2)	0.43(0)(1)(0)(8)
RM123 13	[16]	A					$\,c$	2.40(15)(17)	4.80 (15)(17)	0.50(2)(3)
RM123 11$^{\mathrm{f}}$	[167]	A					$\,c$	*2.43(11)(23)*	*4.78(11)(23)*	*0.51(2)(4)*
Dürr 11$^{\mathrm{e}}$	[132]	A				−	−	2.18(6)(11)	4.87(14)(16)	
RBC 07$^{\mathrm{g}}$	[105]	A					−	3.02(27)(19)	5.49(20)(34)	0.550(31)

*a* The masses are renormalized and run nonperturbatively up to a scale of $100~\mathrm{GeV}$ in the $N_f=2$ SF scheme. In this scheme, nonperturbative and NLO running for the quark masses are shown to agree well from 100 GeV all the way down to 2 GeV [135]

*b* The masses are renormalized and run nonperturbatively up to a scale of 4 GeV in the $N_f=3$ RI/MOM scheme. In this scheme, nonperturbative and N$^3$LO running for the quark masses are shown to agree from 6 GeV down to 3 GeV to better than 1% [8]

*c* The masses are renormalized nonperturbatively at scales $1/a\sim 2\div 3~\mathrm{GeV}$ in the $N_f=2$ RI/MOM scheme. In this scheme, nonperturbative and N$^3$LO running for the quark masses are shown to agree from 4 GeV down 2 GeV to better than 3% [142]

$^{{\mathrm{a}}}$ Results are computed in QCD $+$ QED and quoted in an unconventional “Dashen scheme”

$^{\mathrm{b}}$ The calculation includes e.m. and $m_u\ne m_d$ effects through reweighting

$^{\mathrm{c}}$ Values obtained by combining the HPQCD 10 result for $m_s$ with the MILC 09 results for $m_s/m_{ud}$ and $m_u/m_d$

$^{\mathrm{d}}$ The fermion action used is tree-level improved

$^{\mathrm{e}}$ Values obtained by combining the Dürr 11 result for $m_s$ with the BMW 10A, 10B results for $m_s/m_{ud}$ and $m_u/m_d$

$^{\mathrm{f}}$  The results presented on this line are in italics because they do not appear in the quoted paper. Rather, the values for $m_u$, $m_d$ and $m_u/m_d$ are obtained by combining the result of RM123 11 for $(m_d-m_u)$ [167] with $m_{ud}=3.6(2)\,\mathrm{MeV}$ from ETM 10B. $(m_d-m_u)=2.35(8)(24)\,\mathrm{MeV}$ in Ref. [167] was obtained assuming $\epsilon = 0.7(5)$ [1] and $\epsilon _m=\epsilon _{\pi ^0}=\epsilon _{K^0}=0$. In the quoted results, the first error corresponds to the lattice statistical and systematic errors combined in quadrature, while the second arises from the uncertainties associated with $\epsilon$

$^{\mathrm{g}}$ The calculation includes quenched e.m. effects

Instead of subtracting electromagnetic effects using phenomenology, RBC 07 [105] and Blum 10 [103] actually include a quenched electromagnetic field in their calculation. This means that their results include corrections to Dashen’s theorem, albeit only in the presence of quenched electromagnetism. Since the up and down quarks in the sea are treated as degenerate, very small isospin corrections are neglected, as in MILC’s calculation.

PACS-CS 12 [143] takes the inclusion of isospin-breaking effects one step further. Using reweighting techniques, it also includes electromagnetic and $m_u-m_d$ effects in the sea.

Lattice results for $m_u$, $m_d$ and $m_u/m_d$ are summarized in Table 7. In order to discuss them, we consider the LO formula

26 $$\begin{aligned} \frac{m_u}{m_d}\mathop {=}\limits ^{{\mathrm{LO}}}\frac{\hat{M}_{K^+}^2-\hat{M}_{K^0}^2+\hat{M}_{\pi ^+}^2}{\hat{M}_{K^0}^2-\hat{M}_{K^+}^2+\hat{M}_{\pi ^+}^2} .\end{aligned}$$

Using Eqs. (10)–(12) to express the meson masses in QCD in terms of the physical ones and linearizing in the corrections, this relation takes the form

27 $$\begin{aligned} \frac{m_u}{m_d}\mathop {=}\limits ^{{\mathrm{LO}}}0.558 - 0.084\, \epsilon - 0.02\, \epsilon _{\pi ^0} + 0.11\, \epsilon _m .\end{aligned}$$

Inserting the estimates (13) and adding errors in quadrature, the LO prediction becomes

28 $$\begin{aligned} \frac{m_u}{m_d}\mathop {=}\limits ^{{\mathrm{LO}}}0.50(3).\end{aligned}$$

Again, the quoted error exclusively accounts for the errors attached to the estimates (13) for the epsilons – contributions of nonleading order are ignored. The uncertainty in the leading-order prediction is dominated by the one in the coefficient $\epsilon$, which specifies the difference between the meson squared-mass splittings generated by the e.m. interaction in the kaon and pion multiplets. The reduction in the error on this coefficient since the previous review [1] results in a reduction of a factor of a little less than 2 in the uncertainty on the LO value of $m_u/m_d$ given in Eq. (28).

It is interesting to compare the assumptions made or results obtained by the different collaborations for the violation of Dashen’s theorem. The input used in MILC 09A is $\epsilon =1.2(5)$ [6], while the $N_f=2$ computation of RM123 13 finds $\epsilon =0.79(18)(18)$ [16]. As discussed in Sect. 3.1.6, the value of *Q* used by BMW 10A, 10B [7, 8] gives $\epsilon =0.70(28)$ at NLO (see Eq. (40)). On the other hand, RBC 07 [105] and Blum 10 [103] obtain the results $\epsilon =0.13(4)$ and $\epsilon =0.5(1)$. The new results from QCDSF/UKQCD 15 give $\epsilon =0.50(6)$ [118]. Note that PACS-CS 12 [143] do not provide results which allow us to determine $\epsilon$ directly. However, using their result for $m_u/m_d$, together with Eq. (27), and neglecting NLO terms, one finds $\epsilon =-1.6(6)$, which is difficult to reconcile with what is known from phenomenology (see Sects. 3.1.1 and 3.1.6). Since the values assumed or obtained for $\epsilon$ differ, it does not come as a surprise that the determinations of $m_u/m_d$ are different.

These values of $\epsilon$ are also interesting because they allow us to estimate the chiral corrections to the LO prediction (28) for $m_u/m_d$. Indeed, evaluating the relation (27) for the values of $\epsilon$ given above, and neglecting all other corrections in this equation, yields the LO values $(m_u/m_d)^\mathrm {LO}=0.46(4)$, 0.547(3), 0.52(1), 0.50(2), 0.49(2) and 0.51(1) for MILC 09A, RBC 07, Blum 10, BMW 10A, 10B, RM123 13, and QCDSF/UKQCD 15, respectively. However, in comparing these numbers to the nonperturbative results of Table 7 one must be careful not to double count the uncertainty arising from $\epsilon$. One way to obtain a sharp comparison is to consider the ratio of the results of Table 7 to the LO values $(m_u/m_d)^\mathrm{LO}$, in which the uncertainty from $\epsilon$ cancels to good accuracy. Here we will assume for simplicity that they cancel completely and will drop all uncertainties related to $\epsilon$. For $N_f = 2$ we consider RM123 13 [16], which updates RM123 11 and has no red dots. Since the uncertainties common to $\epsilon$ and $m_u/m_d$ are not explicitly given in Ref. [16], we have to estimate them. For that we use the leading-order result for $m_u/m_d$, computed with RM123 13’s value for $\epsilon$. Its error bar is the contribution of the uncertainty on $\epsilon$ to $(m_u/m_d)^\mathrm{LO}$. To good approximation this contribution will be the same for the value of $m_u/m_d$ computed in Ref. [16]. Thus, we subtract it in quadrature from RM123 13’s result in Table 7 and compute $(m_u/m_d)/(m_u/m_d)^\mathrm{LO}$, dropping uncertainties related to $\epsilon$. We find $(m_u/m_d)/(m_u/m_d)^\mathrm{LO} = 1.02(6)$. This result suggests that chiral corrections in the case of $N_{ f}=2$ are negligible. For the two most accurate $N_{ f}=2+1$ calculations, those of MILC 09A and BMW 10A, 10B, this ratio of ratios is 0.94(2) and 0.90(1), respectively. Though these two numbers are not fully consistent within our rough estimate of the errors, they indicate that higher-order corrections to Eq. (28) are negative and about 8% when $N_{ f}=2+1$. In the following, we will take them to be -8(4)%. The fact that these corrections are seemingly larger and of opposite sign than in the $N_{ f}=2$ case is not understood at this point. It could be an effect associated with the quenching of the strange quark. It could also be due to the fact that the RM123 13 calculation does not probe deeply enough into the chiral regime – it has $M_\pi \,{\mathop {\sim }\limits ^{{>}}}\,270\,\mathrm{MeV}$ – to pick up on important chiral corrections. Of course, being less than a two-standard-deviation effect, it may be that there is no problem at all and that differences from the LO result are actually small.

Given the exploratory nature of the RBC 07 calculation, its results do not allow us to draw solid conclusions about the e.m. contributions to $m_u/m_d$ for $N_{ f}=2$. As discussed in Sect. 3.1.3 and here, the $N_{ f}=2+1$ results of Blum 10, PACS-CS 12, and QCDSF/UKQCD 15 do not pass our selection criteria either. We therefore resort to the phenomenological estimates of the electromagnetic self-energies discussed in Sect. 3.1.1, which are validated by recent, preliminary lattice results.

Since RM123 13 [16] includes a lattice estimate of e.m. corrections, for the $N_{ f}=2$ final results we simply quote the values of $m_u$, $m_d$, and $m_{u}/m_{d}$ from RM123 13 given in Table 7:29with errors of roughly 10, 5 and 8%, respectively. In these results, the errors are obtained by combining the lattice statistical and systematic errors in quadrature.

For $N_{ f}=2+1$ there is to date no final, published computation of e.m. corrections. Thus, we take the LO estimate for $m_u/m_d$ of Eq. (28) and use the −8(4)% obtained above as an estimate of the size of the corrections from higher orders in the chiral expansion. This gives $m_u/m_d=0.46(3)$. The two individual masses can then be worked out from the estimate (18) for their mean. Therefore, for $N_{ f}=2+1$ we obtain30In these results, the first error represents the lattice statistical and systematic errors, combined in quadrature, while the second arises from the uncertainties associated with e.m. corrections of Eq. (13). The estimates in Eq. (30) have uncertainties of order 5, 3 and 7%, respectively.

Finally, for four flavours we simply adopt the results of ETM 14A which meet all of our criteria.31Naively propagating errors to the end, we obtain $(m_u/m_d)_{N_f=2}/(m_u/m_d)_{N_f=2+1}=1.09(10)$. If instead of Eq. (29) we use the results from RM123 11, modified by the e.m. corrections in Eq. (13), as was done in our previous review, we obtain $(m_u/m_d)_{N_f=2}/(m_u/m_d)_{N_f=2+1}=1.11(7)(1)$, confirming again the strong cancellation of e.m. uncertainties in the ratio. The $N_f=2$ and $2+1$ results are compatible at the 1 to 1.5 $\sigma$ level. Clearly the difference between three and four flavours is even smaller, and completely covered by the quoted uncertainties.

It is interesting to note that in the results above, the errors are no longer dominated by the uncertainties in the input used for the electromagnetic corrections, though these are still significant at the level of precision reached in the $N_f=2+1$ results. This is due to the reduction in the error on $\epsilon$ discussed in Sect. 3.1.1. Nevertheless, the comparison of Eqs. (28) and (30) indicates that more than half of the difference between the prediction $m_u/m_d=0.558$ obtained from Weinberg’s mass formulae [161] and the result for $m_u/m_d$ obtained on the lattice stems from electromagnetism, the higher orders in the chiral perturbation generating a comparable correction.

In view of the fact that a *massless up-quark* would solve the strong CP-problem, many authors have considered this an attractive possibility, but the results presented above exclude this possibility: the value of $m_u$ in Eq. (30) differs from zero by 20 standard deviations. We conclude that nature solves the strong CP-problem differently. This conclusion relies on lattice calculations of kaon masses and on the phenomenological estimates of the e.m. self-energies discussed in Sect. 3.1.1. The uncertainties therein currently represent the limiting factor in determinations of $m_u$ and $m_d$. As demonstrated in Refs. [16, 103–105, 110–116, 123], lattice methods can be used to calculate the e.m. self-energies. Further progress on the determination of the light-quark masses hinges on an improved understanding of the e.m. effects.

#### Estimates for *R* and *Q*

The quark-mass ratios

32 $$\begin{aligned} R\equiv \frac{m_s-m_{ud}}{m_d-m_u}\quad \hbox {and}\quad Q^2\equiv \frac{m_s^2-m_{ud}^2}{m_d^2-m_u^2} \end{aligned}$$

compare *SU*(3) breaking with isospin breaking. The quantity *Q* is of particular interest because of a low-energy theorem [168], which relates it to a ratio of meson masses,

33 $$\begin{aligned}&Q^2_M\equiv \frac{\hat{M}_K^2}{\hat{M}_\pi ^2}\cdot \frac{\hat{M}_K^2-\hat{M}_\pi ^2}{\hat{M}_{K^0}^2- \hat{M}_{K^+}^2},\quad \hat{M}^2_\pi \equiv \frac{1}{2}( \hat{M}^2_{\pi ^+}+ \hat{M}^2_{\pi ^0}) ,\nonumber \\&\quad \hat{M}^2_K\equiv \frac{1}{2}(\hat{M}^2_{K^+}+ \hat{M}^2_{K^0}).\end{aligned}$$

Chiral symmetry implies that the expansion of $Q_M^2$ in powers of the quark masses (i) starts with $Q^2$ and (ii) does not receive any contributions at NLO:

34 $$\begin{aligned} Q_M\mathop {=}\limits ^{{\mathrm{NLO}}}Q .\end{aligned}$$

Inserting the estimates for the mass ratios $m_s/m_{ud}$, and $m_u/m_d$ given for $N_{ f}=2$ in Eqs. (17) and (29) respectively, we obtain

35 $$\begin{aligned} R=40.7(3.7)(2.2),\quad Q=24.3(1.4)(0.6), \end{aligned}$$

where the errors have been propagated naively and the e.m. uncertainty has been separated out, as discussed in the third paragraph after Eq. (28). Thus, the meaning of the errors is the same as in Eq. (30). These numbers agree within errors with those reported in Ref. [16] where values for $m_s$ and $m_{ud}$ are taken from ETM 10B [11].

For $N_{ f}=2+1$, we use Eqs. (24) and (30) and obtain

36 $$\begin{aligned} R=35.7(1.9)(1.8),\quad Q=22.5(6)(6), \end{aligned}$$

where the meaning of the errors is the same as above. The $N_{ f}=2$ and $N_{ f}=2+1$ results are compatible within 2$\sigma$, even taking the correlations between e.m. effects into account.

Again, for $N_{ f}=2+1+1$, we simply take values from ETM 14A,

37 $$\begin{aligned} R=35.6(5.1),\quad Q=22.2(1.6), \end{aligned}$$

which are quite compatible with two and three flavour results.

It is interesting to use these results to study the size of chiral corrections in the relations of *R* and *Q* to their expressions in terms of meson masses. To investigate this issue, we use $\chi$PT to express the quark-mass ratios in terms of the pion and kaon masses in QCD and then again use Eqs. (10)–(12) to relate the QCD masses to the physical ones. Linearizing in the corrections, this leads to

38 $$\begin{aligned} R&\mathop {=}\limits ^{{\mathrm{LO}}}&R_M = 43.9 - 10.8\, \epsilon + 0.2\, \epsilon _{\pi ^0} - 0.2\, \epsilon _{K^0}- 10.7\, \epsilon _m,\nonumber \\ \end{aligned}$$

39 $$\begin{aligned} Q&\mathop {=}\limits ^{{\mathrm{NLO}}}&Q_M = 24.3 - 3.0\, \epsilon + 0.9\, \epsilon _{\pi ^0} - 0.1\, \epsilon _{K^0} + 2.6 \,\epsilon _m .\nonumber \\ \end{aligned}$$

While the first relation only holds to LO of the chiral perturbation series, the second remains valid at NLO, on account of the low-energy theorem mentioned above. The first terms on the right hand side represent the values of *R* and *Q* obtained with the Weinberg leading-order formulae for the quark-mass ratios [161]. Inserting the estimates (13), we find that the e.m. corrections lower the Weinberg values to $R_M= 36.7(3.3)$ and $Q_M= 22.3(9)$, respectively.

Comparison of $R_M$ and $Q_M$ with the full results quoted above gives a handle on higher-order terms in the chiral expansion. Indeed, the ratios $R_M/R$ and $Q_M/Q$ give NLO and NNLO (and higher)-corrections to the relations $R \mathop {=}\limits ^{{\mathrm{LO}}}R_M$ and $Q\mathop {=}\limits ^{{\mathrm{NLO}}}Q_M$, respectively. The uncertainties due to the use of the e.m. corrections of Eq. (13) are highly correlated in the numerators and denominators of these ratios, and we make the simplifying assumption that they cancel in the ratio. Thus, for $N_f=2$ we evaluate Eqs. (38) and (39) using $\epsilon =0.79(18)(18)$ from RM123 13 [16] and the other corrections from Eq. (13), dropping all uncertainties. We divide them by the results for *R* and *Q* in Eq. (35), omitting the uncertainties due to e.m. We obtain $R_M/R\simeq 0.88(8)$ and $Q_M/Q\simeq 0.91(5)$. We proceed analogously for $N_f=2+1$ and 2+1+1, using $\epsilon =0.70(3)$ from Eq. (13) and *R* and *Q* from Eqs. (36) and (37), and find $R_M/R\simeq 1.02(5)$ and 1.03(17), and $Q_M/Q\simeq 0.99(3)$ and 1.00(8). The chiral corrections appear to be small for three and four flavours, especially those in the relation of *Q* to $Q_M$. This is less true for $N_f=2$, where the NNLO and higher corrections to $Q=Q_M$ could be significant. However, as for other quantities which depend on $m_u/m_d$, this difference is not significant.

As mentioned in Sect. 3.1.1, there is a phenomenological determination of *Q* based on the decay $\eta \rightarrow 3\pi$ [169, 170]. The key point is that the transition $\eta \rightarrow 3\pi$ violates isospin conservation. The dominating contribution to the transition amplitude stems from the mass difference $m_u-m_d$. At NLO of $\chi$PT, the QCD part of the amplitude can be expressed in a parameter-free manner in terms of *Q*. It is well known that the electromagnetic contributions to the transition amplitude are suppressed (a thorough recent analysis is given in Ref. [171]). This implies that the result for *Q* is less sensitive to the electromagnetic uncertainties than the value obtained from the masses of the Nambu–Goldstone bosons. For a recent update of this determination and for further references to the literature, we refer to Ref. [172]. Using dispersion theory to pin down the momentum dependence of the amplitude, the observed decay rate implies $Q=22.3(8)$ (since the uncertainty quoted in Ref. [172] does not include an estimate for all sources of error, we have retained the error estimate given in Ref. [165], which is twice as large). The formulae for the corrections of NNLO are available also in this case [173] – the poor knowledge of the effective coupling constants, particularly of those that are relevant for the dependence on the quark masses, is currently the limiting factor encountered in the application of these formulae.

**Table 8:** Our estimates for the strange-quark and the average up-down-quark masses in the ${\overline{\text {MS}}}$ scheme at running scale $\mu =2~\mathrm{GeV}$. Numerical values are given in MeV. In the results presented here, the error is the one which we obtain by applying the averaging procedure of Sect. 2.3 to the relevant lattice results. We have added an uncertainty to the $N_f=2+1$ results, associated with the neglect of the charm sea-quark and isospin-breaking effects, as discussed around Eqs. (18) and (24). This uncertainty is not included in the $N_f=2$ results, as it should be smaller than the uncontrolled systematic associated with the neglect of strange sea-quark effects

$N_{ f}$	$m_{ud}$	$m_s$	$m_s/m_{ud}$
$2+1+1$	3.70(17)	93.9(1.1)	27.30(34)
$2+1$	3.373(80)	92.0(2.1)	27.43(31)
2	3.6(2)	101(3)	27.3(9)

**Table 9:** Our estimates for the masses of the two lightest quarks and related, strong isospin-breaking ratios. Again, the masses refer to the ${\overline{\text {MS}}}$ scheme at running scale $\mu =2\,\mathrm{GeV}$. Numerical values are given in MeV. In the results presented here, the first error is the one that comes from lattice computations, while the second for $N_f=2+1$ is associated with the phenomenological estimate of e.m. contributions, as discussed after Eq. (30). The second error on the $N_f=2$ results for *R* and *Q* is also an estimate of the e.m. uncertainty, this time associated with the lattice computation of Ref. [16], as explained after Eq. (35). We present these results in a separate table, because they are less firmly established than those in Table 8. For $N_f=2+1$ and $2+1+1$ they still include information coming from phenomenology, in particular on e.m. corrections, and for $N_f=2$ the e.m. contributions are computed neglecting the feedback of sea quarks on the photon field

$$N_{ f}$$	$$m_u $$	$$m_d $$	$$m_u/m_d$$	*R*	*Q*
$2+1+1$	2.36(24)	5.03(26)	0.470(56)	35.6(5.1)	22.2 (1.6)
$2+1$	2.16(9)(7)	4.68(14)(7)	0.46(2)(2)	35.0(1.9)(1.8)	22.5(6)(6)
2	2.40(23)	4.80(23)	0.50(4)	40.7(3.7)(2.2)	24.3(1.4)(0.6)

As was to be expected, the central value of *Q* obtained from $\eta$-decay agrees exactly with the central value obtained from the low-energy theorem: we have used that theorem to estimate the coefficient $\epsilon$, which dominates the e.m. corrections. Using the numbers for $\epsilon _m$, $\epsilon _{\pi ^0}$ and $\epsilon _{K^0}$ in Eq. (13) and adding the corresponding uncertainties in quadrature to those in the phenomenological result for *Q*, we obtain

40 $$\begin{aligned} \epsilon \mathop {=}\limits ^{{\mathrm{NLO}}}0.70(28).\end{aligned}$$

The estimate (13) for the size of the coefficient $\epsilon$ is taken from this, as is confirmed by the most recent, preliminary lattice determinations [16, 110–112, 115, 116].

Our final results for the masses $m_u$, $m_d$, $m_{ud}$, $m_s$ and the mass ratios $m_u/m_d$, $m_s/m_{ud}$, *R*, *Q* are collected in Tables 8 and 9. We separate $m_u$, $m_d$, $m_u/m_d$, *R* and *Q* from $m_{ud}$, $m_s$ and $m_s/m_{ud}$, because the latter are completely dominated by lattice results while the former still include some phenomenological input.

### Charm-quark mass

In the present review we collect and discuss for the first time the lattice determinations of the $\overline{\mathrm{MS}}$ charm-quark mass $\overline{m}_c$. Most of the results have been obtained by analyzing the lattice-QCD simulations of 2-point heavy–light- or heavy–heavy-meson correlation functions, using as input the experimental values of the *D*, $D_s$ and charmonium mesons. The exceptions are represented by the HPQCD 14A [5] result at $N_f = 2+1+1$, the HPQCD 08B [152], HPQCD 10 [9] and JLQCD 15B [174] results at $N_f = 2 +1$, and the ETM 11F [175] result at $N_f = 2$, where the moments method has been employed. The latter is based on the lattice calculation of the Euclidean time moments of pseudoscalar-pseudoscalar correlators for heavy-quark currents followed by an OPE expansion dominated by perturbative QCD effects, which provides the determination of both the heavy-quark mass and the strong coupling constant $\alpha _s$.

The heavy-quark actions adopted by the various lattice collaborations have been reviewed already in the FLAG 13 review [2], and their descriptions can be found in Sect. A.1.3. While the charm mass determined with the moments method does not need any lattice evaluation of the mass renormalization constant $Z_m$, the extraction of $\overline{m}_c$ from 2-point heavy-meson correlators does require the nonperturbative calculation of $Z_m$. The lattice scale at which $Z_m$ is obtained, is usually at least of the order $2{-}3$ GeV, and therefore it is natural in this review to provide the values of $\overline{m}_c(\mu )$ at the renormalization scale $\mu = 3~\mathrm{GeV}$. Since the choice of a renormalization scale equal to $\overline{m}_c$ is still commonly adopted (as by PDG [151]), we have collected in Table 10 the lattice results for both $\overline{m}_c(\overline{m}_c)$ and $\overline{m}_c(\hbox {3 GeV})$, obtained at $N_f = 2$, $2+1$ and $2+1+1$. When not directly available in the publications, we apply a conversion factor equal either to 0.900 between the scales $\mu = 2$ GeV and $\mu = 3$ GeV or to 0.766 between the scales $\mu = \overline{m}_c$ and $\mu = 3$ GeV, obtained using perturbative QCD evolution at four loops assuming $\Lambda _{QCD} = 300$ MeV for $N_f = 4$.

**Table 10:** Lattice results for the ${\overline{\text {MS}}}$-charm-quark mass $\overline{m}_c(\overline{m}_c)$ and $\overline{m}_c(3~\hbox {GeV})$ in GeV, together with the colour coding of the calculations used to obtain these. When not directly available in the publications, a conversion factor equal to 0.900 between the scales $\mu = 2$ GeV and $\mu = 3$ GeV (or equal to 0.766 between the scales $\mu = \overline{m}_c$ and $\mu = 3$ GeV) has been considered

Collaboration	Refs.	$N_f$	Publication status	Chiral extrapolation	Continuum extrapolation	Finite volume	Renormalization	$\overline{m}_c(\overline{m}_c)$	$\overline{m}_c(3~\hbox {GeV})$
HPQCD 14A	[5]	$2+1+1$	A				−	1.2715(95)	0.9851(63)
ETM 14A	[176]	$2+1+1$	A					1.3478(27)(195)	1.0557(22)(153)
ETM 14	[4]	$2+1+1$	A					1.348(46)	1.058(35)
JLQCD 15B	[174]	$2+1$	C				−	1.2769(21)(89)	0.9948(16)(69)
$\chi$QCD 14	[17]	$2+1$	A					1.304(5)(20)	1.006(5)(22)
HPQCD 10	[9]	$2+1$	A				−	1.273(6)	0.986(6)
HPQCD 08B	[152]	$2+1$	A				−	1.268(9)	0.986(10)
ALPHA 13B	[177]	2	C					1.274(36)	0.976(28)
ETM 11F	[175]	2	C				−	1.279(12)/1.296(18)$^{\mathrm{a}}$	0.979(09)/0.998(14)$^{\mathrm{a}}$
ETM 10B	[11]	2	A					1.28(4)	1.03(4)
PDG	[151]							1.275(25)	

$^{\mathrm{a}}$ Two results are quoted

In the next subsections we review separately the results of $\overline{m}_c(\overline{m}_c)$ for the various values of $N_f$.

#### $N_f = 2+1+1$ results

There are three recent results employing four dynamical quarks in the sea. ETM 14 [4] uses 15 twisted-mass gauge ensembles at three lattice spacings ranging from 0.062 to 0.089 fm (using $f_\pi$ as input), in boxes of size ranging from 2.0 to 3.0 fm and pion masses from 210 to 440 MeV (explaining the tag  in the chiral extrapolation and the tag  for the continuum extrapolation). The value of $M_\pi L$ at their smallest pion mass is 3.2 with more than two volumes (explaining the tag  in the finite-volume effects). They fix the strange mass with the kaon mass and the charm one with that of the $D_s$ and *D* mesons.

ETM 14A [176] uses 10 out of the 15 gauge ensembles adopted in ETM 14 spanning the same range of values for the pion mass and the lattice spacing, but the latter is fixed using the nucleon mass. Two lattice volumes with size larger than 2.0 fm are employed. The physical strange and the charm mass are obtained using the masses of the $\Omega ^-$ and $\Lambda _c^+$ baryons, respectively.

HPQCD 14A [5] works with the moments method adopting HISQ staggered fermions. Their results are based on 9 out of the 21 ensembles carried out by the MILC Collaboration [14] at 4 values of the coupling $\beta$ corresponding to lattice spacings in the range from 0.057 to 0.153 fm, in boxes of sizes up to 5.8 fm and with taste-Goldstone-pion masses down to 130 MeV and RMS-pion masses down to 173 MeV. The strange- and charm-quark masses are fixed using as input the lattice result $M_{\bar{s}s} = 688.5 (2.2)~\mathrm{MeV}$, calculated without including $\bar{s}s$ annihilation effects, and $M_{\eta _c} = 2.9863(27)~\mathrm{GeV}$, obtained from the experimental $\eta _c$ mass after correcting for $\bar{c}c$ annihilation and e.m. effects. All of the selection criteria of Sect. 2.1.1 are satisfied with the tag .^14^

According to our rules on the publication status all the three results can enter the FLAG average at $N_f = 2+1+1$. The determinations of $\overline{m}_c$ obtained by ETM 14 and 14A agree quite well with each other, but they are not compatible with the HPQCD 14A result. Therefore we first combine the two ETM results with a 100$\%$ correlation in the statistical error, yielding $\overline{m}_c(\overline{m}_c) = 1.348 (20)~ \mathrm{GeV}$. Then we perform the average with the HPQCD 14A result, obtaining the final FLAG averages,

41 $$\begin{aligned}& \overline{m}_c(\overline{m}_c) = 1.286 ~ (30) ~ \mathrm{GeV}&\,\mathrm {Refs.}~[4,5], \end{aligned}$$

42 $$\begin{aligned}&N_f = 2+1+1:&\nonumber \\& \overline{m}_c(\hbox {3 GeV}) = 0.996 ~ (25)~ \mathrm{GeV}&\,\mathrm {Refs.}~[4,5], \end{aligned}$$

where the errors include a quite large value (3.5 and 4.4, respectively) for the stretching factor $\sqrt{\chi ^2/\hbox {d.o.f.}}$ coming from our rules for the averages discussed in Sect. 2.2.

#### $N_f = 2+1$ results

The HPQCD 10 [9] result is based on the moments method adopting a subset of $N_f = 2+1$ Asqtad-staggered-fermion ensembles from MILC [89], on which HISQ valence fermions are studied. The charm mass is fixed from that of the $\eta _c$ meson, $M_{\eta _c} = 2.9852 (34) ~ \mathrm{GeV}$ corrected for $\bar{c}c$ annihilation and e.m. effects. HPQCD 10 replaces the result HPQCD 08B [152], in which Asqtad staggered fermions have been used also for the valence quarks.

$\chi$QCD 14 [17] uses a mixed-action approach based on overlap fermions for the valence quarks and on domain-wall fermions for the sea quarks. They adopt six of the gauge ensembles generated by the RBC/UKQCD Collaboration [144] at two values of the lattice spacing (0.087 and 0.11 fm) with unitary pion masses in the range from 290 to 420 MeV. For the valence quarks no light-quark masses are simulated. At the lightest pion mass $M_\pi \simeq$ 290 MeV, the value of $M_\pi L$ is 4.1, which satisfies the tag  for the finite-volume effects. The strange- and charm-quark masses are fixed together with the lattice scale by using the experimental values of the $D_s$, $D_s^*$ and $J/\psi$ meson masses.

JLQCD 15B [174] determines the charm mass through the moments method using Möbius domain-wall fermions at three values of the lattice spacing, ranging from 0.044 to 0.083 fm. The lightest pion mass is ${\simeq } 230$ MeV and the corresponding value of $M_\pi L$ is ${\simeq } 4.4$.

Thus, according to our rules on the publication status, the FLAG average for the charm-quark mass at $N_f = 2+1$ is obtained by combining the two results HPQCD 10 and $\chi$QCD 14, leading to

43 $$\begin{aligned}&\overline{m}_c(\overline{m}_c) = 1.275 ~ (8) ~ \mathrm{GeV}&\,\mathrm {Refs.}~[9,17], \end{aligned}$$

44 $$\begin{aligned}&N_f = 2+1:&\nonumber \\&\overline{m}_c(\hbox {3 GeV}) = 0.987 ~ (6)~ \mathrm{GeV}&\,\mathrm {Refs.}~[9,17], \end{aligned}$$

where the error on $\overline{m}_c(\overline{m}_c)$ includes a stretching factor $\sqrt{\chi ^2/\hbox {d.o.f.}} \simeq 1.4$ as discussed in Sect. 2.2.

#### $N_f = 2$ results

We turn now to the three results at $N_f = 2$.

ETM 10B [11] is based on tmQCD simulations at four values of the lattice spacing in the range from 0.05 fm to 0.1 fm, with pion masses as low as 270 MeV at two lattice volumes. They fix the strange-quark mass with either $M_K$ or $M_{\bar{s}s}$ and the charm mass using alternatively the *D*, $D_s$ and $\eta _c$ masses.

ETM 11F [175] is based on the same gauge ensemble as ETM 10B, but the moments method is adopted.

ALPHA 13B uses a subset of the CLS gauge ensembles with $\mathcal {O}(a)$-improved Wilson fermions generated at two values of the lattice spacing (0.048 fm and 0.065 fm), using the kaon decay constant to fix the scale. The pion masses are as low as 190 MeV with the value of $M_\pi L$ equal to $\simeq 4$ at the lightest pion mass (explaining the tag  for finite-volume effects).

According to our rules on the publication status ETM 10B becomes the FLAG average at $N_f = 2$, namely

45 $$\begin{aligned}&\overline{m}_c(\overline{m}_c) = 1.28~(4) ~ \mathrm{GeV}&\,\mathrm {Ref.}~[11], \end{aligned}$$

46 $$\begin{aligned}&N_f = 2:&\nonumber \\&\overline{m}_c(\hbox {3 GeV}) = 1.03~(4)~ \mathrm{GeV}&\,\mathrm {Ref.}~[11]. \end{aligned}$$

**Fig. 4:**
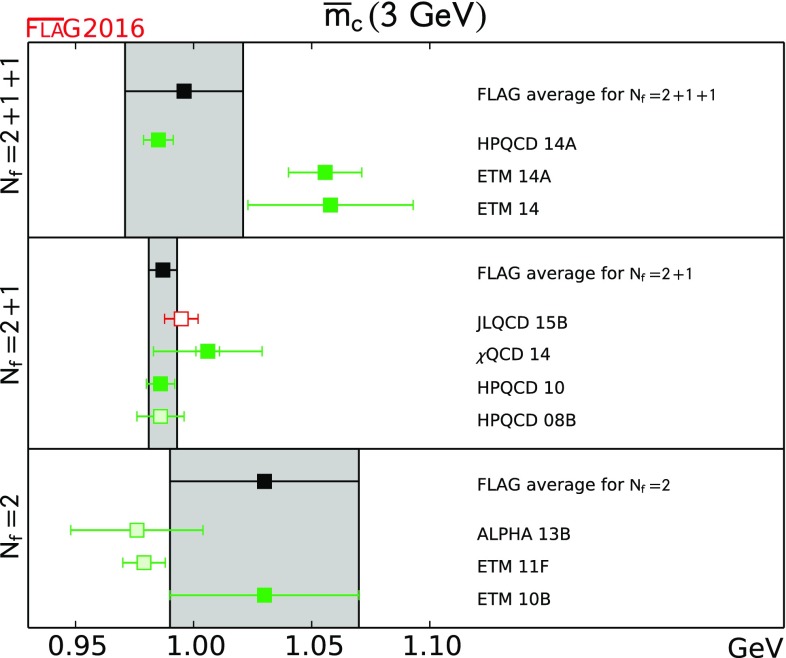
Lattice results and FLAG averages at $N_f = 2$, $2+1$, and $2+1+1$ for the charm-quark mass $\overline{m}_c(3~\mathrm{GeV})$

In Fig. 4 the lattice results of Table 10 and the FLAG averages obtained at $N_f = 2$, $2+1$ and $2+1+1$ are presented.

#### Lattice determinations of the ratio $m_c/m_s$

Because some of the results for the light-quark masses given in this review are obtained via the quark-mass ratio $m_c/m_s$, we now review also these lattice calculations, which are listed in Table 11.

**Table 11:** Lattice results for the quark-mass ratio $m_c/m_s$, together with the colour coding of the calculations used to obtain these

Collaboration	Refs.	$N_{ f}$	Publication status	Chiral extrapolation	Continuum extrapolation	Finite volume	$m_c/m_s$
HPQCD 14A	[5]	$2+1+1$	A				11.652(35)(55)
FNAL/MILC 14A	[14]	$2+1+1$	A				11.747(19)($^{+59}_{-43}$)
ETM 14	[4]	$2+1+1$	A				11.62(16)
$\chi$QCD 14	[17]	$2+1$	A				11.1(8)
HPQCD 09A	[18]	$2+1$	A				11.85(16)
ETM 14D	[160]	2	C				12.29(10)
Dürr 11	[132]	2	A				11.27(30)(26)
ETM 10B	[11]	2	A				12.0(3)

We begin with the $N_f = 2$ results. Besides the result ETM 10B, already discussed in Sect. 3.2.3, there are two more results, Dürr 11 [132] and ETM 14D [160]. Dürr 11 [132] is based on QCDSF $N_f = 2$ $\mathcal {O}(a)$-improved Wilson-fermion simulations [139, 178] on which valence, Brillouin-improved Wilson quarks [179] are considered. It features only 2 ensembles with $M_\pi < 400~\mathrm{MeV}$. The bare axial-Ward-identity (AWI) masses for $m_s$ and $m_c$ are tuned to simultaneously reproduce the physical values of $M_{\bar{s}s}^2/(M_{D_s^*}^2-M_{D_s}^2)$ and $(2M_{D_s^*}^2-M_{\bar{s}s}^2)/(M_{D_s^*}^2-M_{D_s}^2)$, where $M_{\bar{s}s}^2 = 685.8 (8)$ MeV is the quark-connected-$\bar{s}s$ pseudoscalar mass.

The ETM 14D result [160] is based on recent ETM gauge ensembles generated close to the physical point with the addition of a clover term to the tmQCD action. The new simulations are performed at a single lattice spacing of ${\simeq } 0.09$ fm and at a single box size $L \simeq 4$ fm and therefore their calculations do not pass our criteria for the continuum extrapolation and finite-volume effects. The FLAG average at $N_f = 2$ can be therefore obtained by averaging ETM 10B and Dürr 11, obtaining

47 $$\begin{aligned} N_f = 2: \quad m_c / m_s = 11.74 ~ (35)\quad \,\mathrm {Refs.}~[11,132], \end{aligned}$$

where the error includes the stretching factor $\sqrt{\chi ^2/\hbox {d.o.f.}} \simeq 1.5$.

The situation is similar also for the $N_f = 2+1$ results, as besides $\chi$QCD 14 there is only the result HPQCD 09A [18]. The latter is based on a subset of $N_f = 2+1$ Asqtad-staggered-fermion simulations from MILC, on which HISQ-valence fermions are studied. The strange mass is fixed with $M_{\bar{s}s} = 685.8(4.0),\mathrm{MeV}$ and the charm’s from that of the $\eta _c$, $M_{\eta _c} = 2.9852(34)~\mathrm{GeV}$ corrected for $\bar{c}c$ annihilation and e.m. effects. By combing the results $\chi$QCD 14 and HPQCD 09A we obtain

48 $$\begin{aligned} N_f = 2+1: \quad m_c / m_s = 11.82 ~ (16)\quad \,\mathrm {Refs.}~[17,18], \end{aligned}$$

with a $$\chi ^2/\hbox {d.o.f.} \simeq 0.85$$.

Turning now to the $N_f = 2+1+1$ results, in addition to the HPQCD 14A and ETM 14 calculations, already described in Sect. 3.2.1, we consider the recent FNAL/MILC 14 result [14], where HISQ staggered fermions are employed. Their result is based on the use of 21 gauge ensembles at 4 values of the coupling $\beta$ corresponding to lattice spacings in the range from 0.057 to 0.153 fm, in boxes of sizes up to 5.8 fm and with taste-Goldstone-pion masses down to 130 MeV and RMS-pion masses down to 143 MeV. They fix the strange mass with $M_{\bar{s}s}$, corrected for e.m. effects with $\bar{\epsilon }= 0.84(20)$ [113]. The charm mass is fixed with the mass of the $D_s$ meson. As for the HPQCD 14A result, all of our selection criteria are satisfied with the tag . However, a slight tension exists between the two results. Indeed by combining HPQCD 14A and FNAL/MILC 14 results, assuming a 100 $\%$ correlation between the statistical errors (since the two results share the same gauge configurations), we obtain $m_c / m_s = 11.71 (6)$, where the error includes the stretching factor $\sqrt{\chi ^2/\hbox {d.o.f.}} \simeq 1.35$. A further average with the ETM 14A result leads to our final average

49 $$\begin{aligned} N_f = 2+1+1: \quad m_c / m_s = 11.70 ~ (6)\quad \,\mathrm {Refs.}~[4,5,14],\nonumber \\ \end{aligned}$$

which has a remarkable overall precision of 0.5$\%$.

**Fig. 5:**
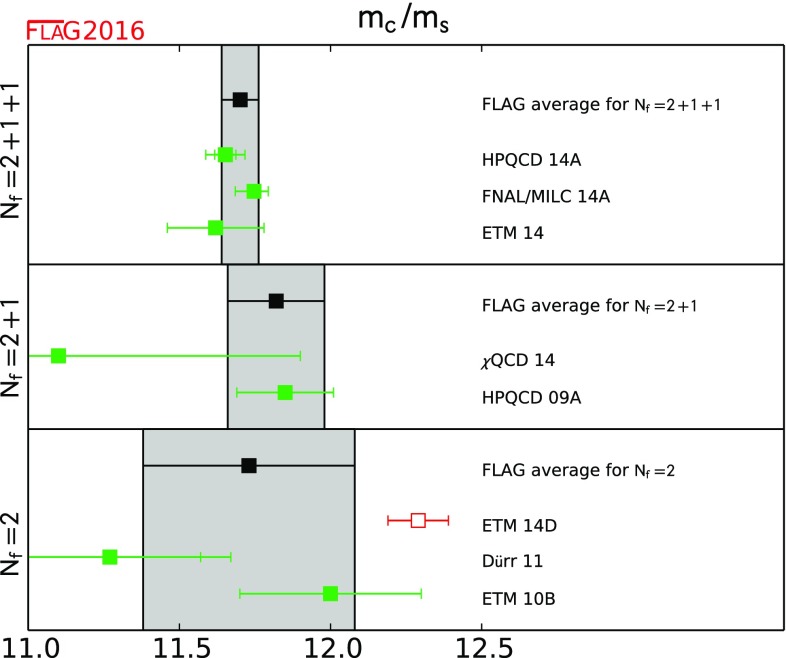
Lattice results for the ratio $m_c / m_s$ listed in Table 11 and the FLAG averages corresponding to $N_f =2$, $2+1$ and $2+1+1$

All of the results for $m_c/m_s$ discussed above are shown in Fig. 5 together with the FLAG averages corresponding to $N_f =2$, $2+1$ and $2+1+1$.

### Bottom-quark mass

We now give the lattice results for the $\overline{\mathrm{MS}}$-bottom-quark mass $\overline{m}_b$ for the first time as part of this review. Related heavy-quark actions and observables have been discussed in the FLAG 13 review [2], and descriptions can be found in Sect. A.1.3. In Table 12 we have collected the lattice results for $\overline{m}_b(\overline{m}_b)$ obtained at $N_f = 2$, $2+1$ and $2+1+1$, which in the following we review separately. Available results for the quark-mass ratio $m_b / m_c$ are also reported. Afterwards we evaluate the corresponding FLAG averages.

**Table 12:** Lattice results for the ${\overline{\text {MS}}}$-bottom-quark mass $\overline{m}_b(\overline{m}_b)$ in GeV, together with the systematic error ratings for each. Available results for the quark mass ratio $m_b / m_c$ are also reported

Collaboration	Refs.	$N_f$	Publication status	Chiral extrapolation	Continuum extrapolation	Finite volume	Renormalization	Heavy-quark treatment	$\overline{m}_b(\overline{m}_b)$	$m_b / m_c$
HPQCD 14B	[19]	$2+1+1$	A						4.196(23)$^{\mathrm{a}}$	
ETM 14B	[180]	$2+1+1$	C						4.26(7)(14)	4.40(6)(5)
HPQCD 14A	[5]	$2+1+1$	A				−		4.162(48)	4.528(14)(52)
HPQCD 13B	[181]	$2+1$	A			−	−		4.166(43)	
HPQCD 10	[9]	$2+1$	A				−		4.164(23)$^{\mathrm{b}}$	4.51(4)
ETM 13B	[20]	2	A						4.31(9)(8)	
ALPHA 13C	[21]	2	A						4.21(11)	
ETM 11A	[182]	2	A						4.29(14)	
PDG	[151]								4.18(3)	

$^{\mathrm{a}}$ Warning: only two pion points are used for chiral extrapolation

$^{\mathrm{b}}$ The number that is given is $m_b(10~\mathrm{GeV}, N_f = 5) = 3.617(25)~\mathrm{GeV}$

#### $N_f=2+1+1$

Results have been published by HPQCD using NRQCD and HISQ-quark actions (HPQCD 14B  [19] and HPQCD 14A [5], respectively). In both works the *b*-quark mass is computed with the moments method, that is, from Euclidean-time moments of 2-point, heavy–heavy meson correlation functions (see Sect. 9.7 for a description of the method).

In HPQCD 14B the *b*-quark mass is computed from ratios of the moments $R_n$ of heavy current–current correlation functions, namely

50 $$\begin{aligned} \left[ \frac{R_n r_{n-2}}{R_{n-2}r_n}\right] ^{1/2} \frac{\bar{M}_\mathrm{kin}}{2 m_b} = \frac{\bar{M}_{\Upsilon ,\eta _b}}{2 \bar{m}_b(\mu )}, \end{aligned}$$

where $r_n$ are the perturbative moments calculated at N$^3$LO, $\bar{M}_\mathrm{kin}$ is the spin-averaged kinetic mass of the heavy-heavy vector and pseudoscalar mesons and $\bar{M}_{\Upsilon ,\eta _b}$ is the experimental spin average of the $\Upsilon$ and $\eta _b$ masses. The kinetic mass $\bar{M}_\mathrm{kin}$ is chosen since in the lattice calculation the splitting of the $\Upsilon$ and $\eta _b$ states is inverted. In Eq. (50) the bare mass $m_b$ appearing on the left hand side is tuned so that the spin-averaged mass agrees with experiment, while the mass $\overline{m}_b$ at the fixed scale $\mu = 4.18$ GeV is extrapolated to the continuum limit using three HISC (MILC) ensembles with $a \approx 0.15, 0.12$ and 0.09 fm and two pion masses, one of which is the physical one. Therefore according to our rules on the chiral extrapolation a warning must be given. Their final result is $\overline{m}_b(\mu = 4.18~\mathrm{GeV}) = 4.207(26)$ GeV, where the error is from adding systematic uncertainties in quadrature only (statistical errors are smaller than $0.1 \%$ and ignored). The errors arise from renormalization, perturbation theory, lattice spacing, and NRQCD systematics. The finite-volume uncertainty is not estimated, but at the lowest pion mass they have $m_\pi L \simeq 4$, which leads to the tag .

In HPQCD 14A the quark mass is computed using a similar strategy as above but with HISQ heavy quarks instead of NRQCD. The gauge-field ensembles are the same as in HPQCD 14B above plus the one with $a = 0.06$ fm (four lattice spacings in all). Bare heavy-quark masses are tuned to their physical values using the $\eta _h$ mesons, and ratios of ratios yield $m_h/m_c$. The $\overline{\mathrm{MS}}$-charm-quark mass determined as described in Sect. 3.2 then gives $m_b$. The moment ratios are expanded using the OPE, and the quark masses and $\alpha _S$ are determined from fits of the lattice ratios to this expansion. The fits are complicated: HPQCD uses cubic splines for valence- and sea-mass dependence, with several knots, and many priors for 21 ratios to fit 29 data points. Taking this fit at face value results in a  rating for the continuum limit since they use four lattice spacings down to 0.06 fm. See, however, the detailed discussion of the continuum limit given in Sect. 9.7 on $\alpha _S$.

The third four-flavour result is from the ETM Collaboration and appears in a conference proceedings, so it is not included in our final average. The calculation is performed on a set of configurations generated with twisted Wilson fermions with three lattice spacings in the range 0.06 to 0.09 fm and with pion masses in the range 210 to 440 MeV. The *b*-quark mass is determined from a ratio of heavy–light pseudoscalar meson masses designed to yield the quark pole mass in the static limit. The pole mass is related to the $\overline{\mathrm{MS}}$ mass through perturbation theory at N$^3$LO. The key idea is that by taking ratios of ratios, the *b*-quark mass is accessible through fits to heavy–light(strange)-meson correlation functions computed on the lattice in the range ${\sim }1{-}2\times m_c$ and the static limit, the latter being exactly 1. By simulating below $\overline{m}_b$, taking the continuum limit is easier. They find $\overline{m}_b(\overline{m}_b) = 4.26(7)(14)$ GeV, where the first error is statistical and the second systematic. The dominant errors come from setting the lattice scale and fit systematics.

#### $N_f=2+1$

HPQCD 13B  [181] extracts $\overline{m}_b$ from a lattice determination of the $\Upsilon$ energy in NRQCD and the experimental value of the meson mass. The latter quantities yield the pole mass which is related to the $\overline{\mathrm{MS}}$ mass in 3-loop perturbation theory. The MILC coarse (0.12 fm) and fine (0.09 fm) Asqtad-$2+1$-flavour ensembles are employed in the calculation. The bare light-(sea)-quark masses correspond to a single, relatively heavy, pion mass of about 300 MeV. No estimate of the finite-volume error is given.

The value of $\overline{m}_b(\overline{m}_b)$ reported in HPQCD 10 [9] is computed in a very similar fashion to the one in HPQCD 14A described in the last section, except that MILC $2+1$-flavour-Asqtad ensembles are used under HISQ-heavy-valence quarks. The lattice spacings of the ensembles range from 0.18 to 0.045 fm and pion masses down to about 165 MeV. In all, 22 ensembles were fit simultaneously. An estimate of the finite-volume error based on leading-order perturbation theory for the moment ratio is also provided. Details of perturbation theory and renormalization systematics are given in Sect. 9.7.

#### $N_f=2$

The ETM Collaboration computes $\overline{m}_b(\overline{m}_b)$ using the ratio method described above on two-flavour twisted-mass gauge ensembles with four values of the lattice spacing in the range 0.10 to 0.05 fm and pion masses between 280 and 500 MeV (ETM 13B updates ETM 11). The heavy-quark masses cover a range from charm to a little more than three GeV, plus the exact static-limit point. They find $\overline{m}_b(\overline{m}_b) = 4.31(9)(8)$ GeV for two-flavour running, while $\overline{m}_b(\overline{m}_b) = 4.27(9)(8)$ using four-flavour running, from the 3 GeV scale used in the N$^3$LO perturbative matching calculation from the pole mass to the $\overline{\mathrm{MS}}$ mass. The latter are computed nonperturbatively in the RI-MOM scheme at 3 GeV and matched to $\overline{\mathrm{MS}}$. The dominant errors are combined statistical $+$ fit(continuum $+$ chiral limits) and the uncertainty in setting the lattice scale. ETM quotes the average of two- and five-flavour results, $\overline{m}_b(\overline{m}_b) = 4.29(9)(8)(2)$ where the last error is one-half the difference between the two. In our average (see below), we use the two-flavour result.

The Alpha Collaboration uses HQET for heavy–light mesons to obtain $m_b$ [21] (ALPHA 13C). They employ CLS, nonperturbatively improved, Wilson gauge field ensembles with three lattice spacings (0.075–0.048 fm), pion masses from 190 to 440 MeV, and three or four volumes at each lattice spacing, with $m_\pi L > 4.0$. The bare-quark mass is related to the RGI-scheme mass using the Schrödinger Functional technique with conversion to $\overline{\mathrm{MS}}$ through four-loop anomalous dimensions for the mass. The final result, extrapolated to the continuum and chiral limits, is $\overline{m}_b(\overline{m}_b) = 4.21(11)$ with two-flavour running, where the error combines statistical and systematic uncertainties. The value includes all corrections in HQET through $\Lambda ^2/m_b$, but repeating the calculation in the static limit yields the identical result, indicating the HQET expansion is under very good control.

#### Averages for $\overline{m}_b(\overline{m}_b)$

Taking the results that meet our rating criteria, , or better, we compute the averages from HPQCD 14A and 14B for $N_f = 2+1+1$, ETM 13B and ALPHA 13C for $N_f = 2$, and we take HPQCD 10 as estimate for $N_f = 2+1$, obtaining

51 $$\begin{aligned}&N_f= 2+1+1 :&\overline{m}_b(\overline{m}_b)&= 4.190 (21)&\,\mathrm {Refs.}~[5,19],\end{aligned}$$

52 $$\begin{aligned}&N_f= 2+1 :&\overline{m}_b(\overline{m}_b)&= 4.164 (23)&\,\mathrm {Ref.}~[9], \end{aligned}$$

53 $$\begin{aligned}&N_f= 2 :&\overline{m}_b(\overline{m}_b)&= 4.256 (81)&\,\mathrm {Refs.}~[20,21]. \end{aligned}$$

Since HPQCD quotes $$\overline{m}_b(\overline{m}_b)$$ values using $$N_f = 5$$ running, we used those values directly in these $$N_f=2+1+1$$ and $$2+1$$ averages. The results ETM 13B and ALPHA 13C, entering the average at $$N_f = 2$$, correspond to the $$N_f =2 $$ running.

**Fig. 6:**
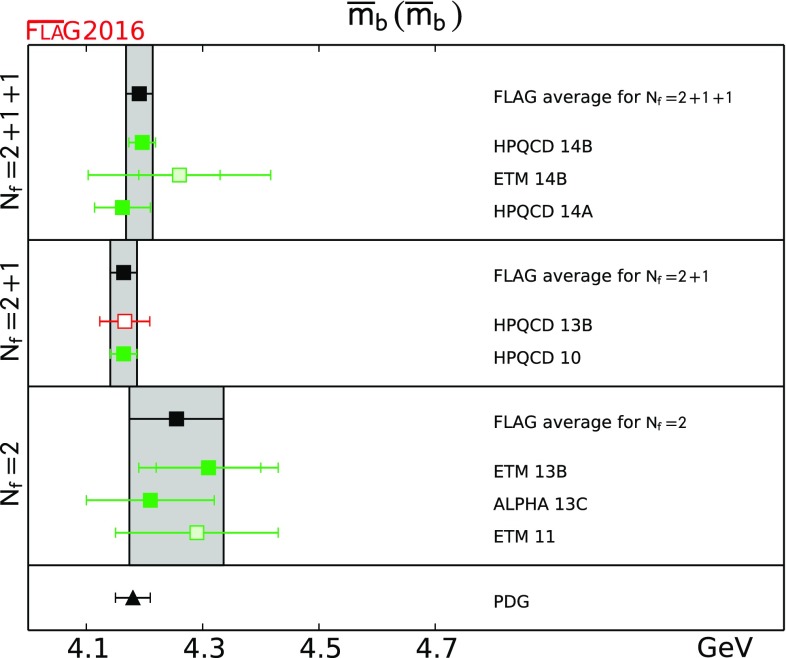
Lattice results and FLAG averages at $N_f = 2$, $2+1$, and $2+1+1$ for the *b*-quark mass $\overline{m}_b(\overline{m}_b)$. The updated PDG value from Ref. [151] is reported for comparison

All the results for $\overline{m}_b(\overline{m}_b)$ discussed above are shown in Fig. 6 together with the FLAG averages corresponding to $N_f = 2$, $2+1$ and $2+1+1$.

## Leptonic and semileptonic kaon and pion decay and $|V_{ud}|$ and $|V_{us}|$

This section summarizes state-of-the-art lattice calculations of the leptonic kaon and pion decay constants and the kaon semileptonic-decay form factor and provides an analysis in view of the Standard Model. With respect to the previous edition of the FLAG review [2] the data in this section has been updated. As in Ref. [2], when combining lattice data with experimental results, we take into account the strong *SU*(2) isospin correction, either obtained in lattice calculations or estimated by using chiral perturbation theory, both for the kaon leptonic decay constant $f_{K^\pm }$ and for the ratio $f_{K^\pm } / f_{\pi ^\pm }$.

### Experimental information concerning $|V_{ud}|$, $|V_{us}|$, $f_+(0)$ and ${f_{K^\pm }}/{f_{\pi ^\pm }}$

The following review relies on the fact that precision experimental data on kaon decays very accurately determine the product $|V_{us}|f_+(0)$ [183] and the ratio $|V_{us}/V_{ud}|f_{K^\pm }/f_{\pi ^\pm }$ [183, 184]:

54 $$\begin{aligned} |V_{us}| f_+(0) = 0.2165(4),\quad \left| \frac{V_{us}}{V_{ud}}\right| \frac{ f_{K^\pm }}{ f_{\pi ^\pm }} \; =0.2760(4).\end{aligned}$$

Here and in the following $f_{K^\pm }$ and $f_{\pi ^\pm }$ are the isospin-broken decay constants, respectively, in QCD (the electromagnetic effects have already been subtracted in the experimental analysis using chiral perturbation theory). We will refer to the decay constants in the *SU*(2) isospin-symmetric limit as $f_K$ and $f_\pi$ (the latter at leading order in the mass difference ($m_u - m_d$) coincides with $f_{\pi ^\pm }$). $|V_{ud}|$ and $|V_{us}|$ are elements of the Cabibbo–Kobayashi–Maskawa matrix and $f_+(t)$ represents one of the form factors relevant for the semileptonic decay $K^0\rightarrow \pi ^-\ell \,\nu$, which depends on the momentum transfer *t* between the two mesons. What matters here is the value at $t=0$: $f_+(0)\equiv f_+^{K^0\pi ^-}(t)\,\Big |_{\;t\rightarrow 0}$. The pion and kaon decay constants are defined by^15^

$$\begin{aligned}&\langle 0|\,\,\overline{d}\gamma _\mu \gamma _5 u|\pi ^+(p)\rangle =i p_\mu f_{\pi ^+},\\&\quad \langle 0|\,\,\overline{s}\gamma _\mu \gamma _5 u|K^+(p)\rangle =i p_\mu f_{K^+}.\end{aligned}$$

In this normalization, $f_{\pi ^\pm } \simeq 130$ MeV, $f_{K^\pm }\simeq 155$ MeV.

The measurement of $|V_{ud}|$ based on superallowed nuclear $\beta$ transitions has now become remarkably precise. The result of the update of Hardy and Towner [186], which is based on 20 different superallowed transitions, reads^16^

55 $$\begin{aligned} |V_{ud}| = 0.97417(21).\end{aligned}$$

**Table 13:** Colour code for the data on $f_+(0)$

Collaboration	Refs.	$N_{ f}$	Publication status	Chiral extrapolation	Continuum extrapolation	Finite-volume errors	$f_+(0)$
ETM 15C	[208]	$2+1+1$	C				0.9709(45)(9)
FNAL/MILC 13E	[22]	$2+1+1$	A				0.9704(24)(22)
FNAL/MILC 13C	[209]	$2+1+1$	C				0.9704(24)(32)
RBC/UKQCD 15A	[24]	$2+1$	A				0.9685(34)(14)
RBC/UKQCD 13	[210]	$2+1$	A				0.9670(20)($^{+18}_{-46}$)
FNAL/MILC 12I	[23]	$2+1$	A				0.9667(23)(33)
JLQCD 12	[211]	$2+1$	C				0.959(6)(5)
JLQCD 11	[212]	$2+1$	C				0.964(6)
RBC/UKQCD 10	[213]	$2+1$	A				0.9599(34)($^{+31}_{-47}$)(14)
RBC/UKQCD 07	[214]	$2+1$	A				0.9644(33)(34)(14)
ETM 10D	[215]	2	C				0.9544(68)$_{\mathrm{stat}}$
ETM 09A	[25]	2	A				0.9560(57)(62)
QCDSF 07	[216]	2	C				0.9647(15)$_{\mathrm{stat}}$
RBC 06	[217]	2	A				0.968(9)(6)
JLQCD 05	[218]	2	C				0.967(6), 0.952(6)

The matrix element $|V_{us}|$ can be determined from semiinclusive $\tau$ decays [193–196]. Separating the inclusive decay $\tau \rightarrow \hbox {hadrons}+\nu$ into nonstrange and strange final states, e.g. HFAG 14 [197] obtain

56 $$\begin{aligned} |V_{us}|=0.2176(21) .\end{aligned}$$

Maltman et al. [195, 198, 199] and Gamiz et al. [200, 201] arrive at very similar values.

Inclusive hadronic $\tau$ decay offers an interesting way to measure $|V_{us}|$, but a number of open issues yet remain to be clarified. In particular, the value of $|V_{us}|$ as determined from $\tau$ decays differs from the result one obtains from assuming three-flavour SM-unitarity by more than three standard deviations [197]. It is important to understand this apparent tension better. A possibility is that at the current level of precision the treatment of higher orders in the operator product expansion and violations of quark-hadron duality may play a role. Very recently [202] a new implementation of the relevant sum rules has been elaborated suggesting a much larger value of $|V_{us}|$ with respect to the result (56), namely $|V_{us}| = 0.2228 (23)$, which is in much better agreement with CKM unitarity. Another possibility is that $\tau$ decay involves new physics, but more work both on the theoretical and experimental side is required.

The experimental results in Eq. (54) are for the semileptonic decay of a neutral kaon into a negatively charged pion and the charged pion and kaon leptonic decays, respectively, in QCD. In the case of the semileptonic decays the corrections for strong and electromagnetic isospin breaking in chiral perturbation theory at NLO have allowed for averaging the different experimentally measured isospin channels [203]. This is quite a convenient procedure as long as lattice QCD does not include strong or QED isospin-breaking effects. Lattice results for $f_K/f_\pi$ are typically quoted for QCD with (squared) pion and kaon masses of $M_\pi ^2=M_{\pi ^0}^2$ and $M_K^2=\frac{1}{2} (M_{K^\pm }^2+M_{K^0}^2-M_{\pi ^\pm }^2+M_{\pi ^0}^2)$ for which the leading strong and electromagnetic isospin violations cancel. While progress is being made for including strong and electromagnetic isospin breaking in the simulations (e.g. Refs. [16, 93, 167, 204–207]), for now contact to experimental results is made by correcting leading *SU*(2) isospin breaking guided either by chiral perturbation theory or by lattice calculations.

**Table 14:** Colour code for the data on the ratio of decay constants: $f_K/f_\pi$ is the pure QCD *SU*(2)-symmetric ratio, while $f_{K^\pm }/f_{\pi ^\pm }$ is in pure QCD including the *SU*(2) isospin-breaking correction

Collaboration	Refs.	$N_{ f}$	Publication status	Chiral extrapolation	Continuum extrapolation	Finite-volume errors	$f_K/f_\pi$	$f_{K^\pm }/f_{\pi ^\pm }$
ETM 14E	[27]	$2+1+1$	A				1.188(11)(11)	1.184(12)(11)
FNAL/MILC 14A	[14]	$2+1+1$	A					1.1956(10)($_{-18}^{+26}$)
ETM 13F	[230]	$2+1+1$	C				1.193(13)(10)	1.183(14)(10)
HPQCD 13A	[26]	$2+1+1$	A				1.1948(15)(18)	1.1916(15)(16)
MILC 13A	[231]	$2+1+1$	A					1.1947(26)(37)
MILC 11	[232]	$2+1+1$	C					1.1872(42)$_\mathrm{stat.}{}^{\mathrm{a}}$
ETM 10E	[233]	$2+1+1$	C				1.224(13)$_\mathrm{stat}$	
RBC/UKQCD 14B	[10]	$2+1$	A				1.1945(45)	
RBC/UKQCD 12	[31]	$2+1$	A				1.199(12)(14)	
Laiho 11	[44]	$2+1$	C					$1.202(11)(9)(2)(5)^{\mathrm{b}}$
MILC 10	[29]	$2+1$	C					1.197(2)($^{+3}_{-7}$)
JLQCD/TWQCD 10	[234]	$2+1$	C				1.230(19)	
RBC/UKQCD 10A	[144]	$2+1$	A				1.204(7)(25)	
PACS-CS 09	[94]	$2+1$	A				1.333(72)	
BMW 10	[30]	$2+1$	A				1.192(7)(6)	
JLQCD/TWQCD 09A	[235]	$2+1$	C				$1.210(12)_\mathrm{stat}$	
MILC 09A	[6]	$2+1$	C					1.198(2)($^{+6}_{-8}$)
MILC 09	[89]	$2+1$	A					1.197(3)($^{\;+6}_{-13}$)
Aubin 08	[236]	$2+1$	C					1.191(16)(17)
PACS-CS 08, 08A	[93, 237]	$2+1$	A				1.189(20)	
RBC/UKQCD 08	[145]	$2+1$	A				1.205(18)(62)	
HPQCD/UKQCD 07	[28]	$2+1$	A				1.189(2)(7)	
NPLQCD 06	[238]	$2+1$	A				1.218(2)($^{+11}_{-24}$)	
MILC 04	[107]	$2+1$	A					1.210(4)(13)
ETM 14D	[160]	2	C				1.203(5)$_\mathrm{stat}$	
ALPHA 13A	[239]	2	C				1.1874(57)(30)	
BGR 11	[240]	2	A				1.215(41)	
ETM 10D	[215]	2	C				1.190(8)$_\mathrm{stat}$	
ETM 09	[32]	2	A				1.210(6)(15)(9)	
QCDSF/UKQCD 07	[241]	2	C				1.21(3)	

$^{\mathrm{a}}$ Result with statistical error only from polynomial interpolation to the physical point

$^{\mathrm{b}}$ This work is the continuation of Aubin 08

### Lattice results for $f_+(0)$ and $f_{K^\pm }/f_{\pi ^\pm }$

The traditional way of determining $|V_{us}|$ relies on using estimates for the value of $f_+(0)$, invoking the Ademollo–Gatto theorem [219]. Since this theorem only holds to leading order of the expansion in powers of $m_u$, $m_d$ and $m_s$, theoretical models are used to estimate the corrections. Lattice methods have now reached the stage where quantities like $f_+(0)$ or $f_K/f_\pi$ can be determined to good accuracy. As a consequence, the uncertainties inherent in the theoretical estimates for the higher-order effects in the value of $f_+(0)$ do not represent a limiting factor any more and we shall therefore not invoke those estimates. Also, we will use the experimental results based on nuclear $\beta$ decay and $\tau$ decay exclusively for comparison – the main aim of the present review is to assess the information gathered with lattice methods and to use it for testing the consistency of the SM and its potential to provide constraints for its extensions.

**Fig. 7:**
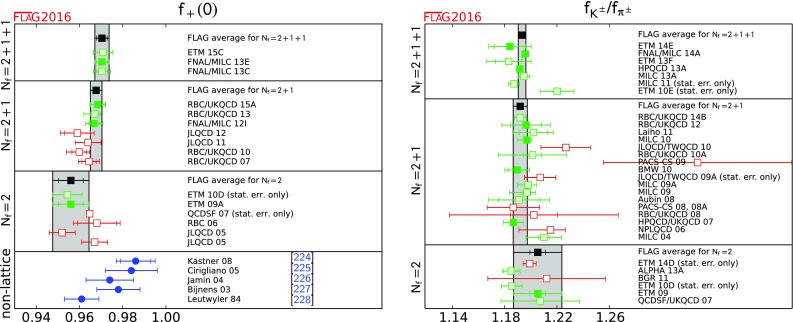
Comparison of lattice results (*squares*) for $f_+(0)$ and $f_{K^\pm }/ f_{\pi ^\pm }$ with various model estimates based on $\chi$PT (*blue circles*). The ratio $f_{K^\pm }/f_{\pi ^\pm }$ is obtained in pure QCD including the *SU*(2) isospin-breaking correction (see Sect. 4.3). The *black squares* and *grey bands* indicate our estimates. The *significance of the colours* is explained in Sect. 2

The database underlying the present review of the semileptonic form factor and the ratio of decay constants is listed in Tables 13 and 14. The properties of the lattice data play a crucial role for the conclusions to be drawn from these results: range of $M_\pi$, size of $L M_\pi$, continuum extrapolation, extrapolation in the quark masses, finite-size effects, etc. The key features of the various datasets are characterized by means of the colour code specified in Sect. 2.1. More detailed information on individual computations are compiled in Appendix B.2.

The quantity $f_+(0)$ represents a matrix element of a strangeness-changing null-plane charge, $f_+(0)=\langle K|Q^{us}|\pi \rangle$. The vector charges obey the commutation relations of the Lie algebra of *SU*(3), in particular $[Q^{us},Q^{su}]=Q^{uu-ss}$. This relation implies the sum rule $\sum _n |\langle K|Q^{us}|n \rangle |^2-\sum _n |\langle K|Q^{su}|n \rangle |^2=1$. Since the contribution from the one-pion intermediate state to the first sum is given by $f_+(0)^2$, the relation amounts to an exact representation for this quantity [220]:

57 $$\begin{aligned} f_+(0)^2=1-\sum _{n\ne \pi } |\langle K|Q^{us}|n \rangle |^2+\sum _n |\langle K |Q^{su}|n \rangle |^2.\end{aligned}$$

While the first sum on the right extends over nonstrange intermediate states, the second runs over exotic states with strangeness $\pm 2$ and is expected to be small compared to the first.

The expansion of $f_+(0)$ in *SU*(3) chiral perturbation theory in powers of $m_u$, $m_d$ and $m_s$ starts with $f_+(0)=1+f_2+f_4+\cdots$ [129]. Since all of the low-energy constants occurring in $f_2$ can be expressed in terms of $M_\pi$, $M_K$, $M_\eta$ and $f_\pi$ [221], the NLO correction is known. In the language of the sum rule (57), $f_2$ stems from nonstrange intermediate states with three mesons. Like all other nonexotic intermediate states, it lowers the value of $f_+(0)$: $f_2=-0.023$ when using the experimental value of $f_\pi$ as input. The corresponding expressions have also been derived in quenched or partially quenched (staggered) chiral perturbation theory [23, 222]. At the same order in the *SU*(2) expansion [223], $f_+(0)$ is parameterized in terms of $M_\pi$ and two a priori unknown parameters. The latter can be determined from the dependence of the lattice results on the masses of the quarks. Note that any calculation that relies on the $\chi$PT formula for $f_2$ is subject to the uncertainties inherent in NLO results: instead of using the physical value of the pion decay constant $f_\pi$, one may, for instance, work with the constant $f_0$ that occurs in the effective Lagrangian and represents the value of $f_\pi$ in the chiral limit. Although trading $f_\pi$ for $f_0$ in the expression for the NLO term affects the result only at NNLO, it may make a significant numerical difference in calculations where the latter are not explicitly accounted for (the lattice results concerning the value of the ratio $f_\pi /f_0$ are reviewed in Sect. 5.3).

The lattice results shown in the left panel of Fig. 7 indicate that the higher-order contributions $\Delta f\equiv f_+(0)-1-f_2$ are negative and thus amplify the effect generated by $f_2$. This confirms the expectation that the exotic contributions are small. The entries in the lower part of the left panel represent various model estimates for $f_4$. In Ref. [228] the symmetry-breaking effects are estimated in the framework of the quark model. The more recent calculations are more sophisticated, as they make use of the known explicit expression for the $K_{\ell 3}$ form factors to NNLO in $\chi$PT [227, 229]. The corresponding formula for $f_4$ accounts for the chiral logarithms occurring at NNLO and is not subject to the ambiguity mentioned above.^17^ The numerical result, however, depends on the model used to estimate the low-energy constants occurring in $f_4$ [224–227]. The figure indicates that the most recent numbers obtained in this way correspond to a positive or an almost vanishing rather than a negative value for $\Delta f$. We note that FNAL/MILC 12I [23] have made an attempt at determining a combination of some of the low-energy constants appearing in $f_4$ from lattice data.

### Direct determination of $f_+(0)$ and $f_{K^\pm }/f_{\pi ^\pm }$

All lattice results for the form factor $f_+(0)$ and many available results for the ratio of decay constants, which we summarize here in Tables 13 and 14, respectively, have been computed in isospin-symmetric QCD. The reason for this unphysical parameter choice is that there are only few simulations of *SU*(2) isospin-breaking effects in lattice QCD, which is ultimately the cleanest way for predicting these effects  [16, 103, 104, 110, 115, 167, 206, 207]. In the meantime one relies either on chiral perturbation theory [107, 129] to estimate the correction to the isospin limit or one calculates the breaking at leading order in $(m_u-m_d)$ in the valence quark sector by extrapolating the lattice data for the charged kaons to the physical value of the *up*(*down*)-quark mass (the result for the pion decay constant is always extrapolated to the value of the average light-quark mass $\hat{m}$). This defines the prediction for $f_{K^\pm }/f_{\pi ^\pm }$.

Since the majority of the collaborations present their newest results including the strong *SU*(2) isospin-breaking correction (as we will see this comprises the majority of results which qualify for inclusion into the FLAG average), we prefer to provide in Fig. 7 the overview of the world data of $f_{K^\pm }/f_{\pi ^\pm }$, at variance with the choice made in the previous edition of the FLAG review [2]. For all the results of Table 14 provided only in the isospin-symmetric limit we apply individually an isospin correction which will be described later on (see equations Eqs. (62)–(63)).

The plots in Fig. 7 illustrate our compilation of data for $f_+(0)$ and $f_{K^\pm }/f_{\pi ^\pm }$. The lattice data for the latter quantity are largely consistent even when comparing simulations with different $N_f$, while in the case of $f_+(0)$ a slight tendency to get higher values for increasing $N_f$ seems to be visible, even if it does not exceed one standard deviation. We now proceed to form the corresponding averages, separately for the data with $N_{ f}=2+1+1$, $N_{ f}=2+1$ and $N_{ f}=2$ dynamical flavours and in the following we will refer to these averages as the “direct” determinations.

For $f_+(0)$ there are currently two computational strategies: FNAL/MILC uses the Ward identity to relate the $K\rightarrow \pi$ form factor at zero momentum transfer to the matrix element $\langle \pi |S|K\rangle$ of the flavour-changing scalar current. Peculiarities of the staggered fermion discretization used by FNAL/MILC (see Ref. [23]) makes this the favoured choice. The other collaborations are instead computing the vector-current matrix element $\langle \pi |V_\mu |K\rangle$. Apart from FNAL/MILC 13C and the recent FNAL/MILC 13E all simulations in Table 13 involve unphysically heavy quarks and therefore the lattice data needs to be extrapolated to the physical-pion and -kaon masses corresponding to the $K^0\rightarrow \pi ^-$ channel. We note also that the recent computations of $f_+(0)$ obtained by the FNAL/MILC and RBC/UKQCD Collaborations make use of the partially twisted boundary conditions to determine the form-factor results directly at the relevant kinematical point $q^2=0$ [242, 243], avoiding in this way any uncertainty due to the momentum dependence of the vector and/or scalar form factors. The ETM Collaboration uses partially twisted boundary conditions to compare the momentum dependence of the scalar and vector form factors with the one of the experimental data [215], while keeping at the same time the advantage of the high-precision determination of the scalar form factor at the kinematical end-point $q_{\mathrm{max}}^2 = (M_K - M_\pi )^2$ [25, 244] for the interpolation at $q^2 = 0$.

According to the colour codes reported in Table 13 and to the FLAG rules of Sect. 2.2, only the result ETM 09A with $N_{ f}=2$, the results FNAL/MILC 12I and RBC/UKQCD 15A with $N_{ f}=2+1$ and the result FNAL/MILC 13E with $N_{ f}=2+1+1$ dynamical flavours of fermions, respectively, can enter the FLAG averages.

At $N_{ f}=2+1+1$ the new result from the FNAL/MILC Collaboration, $f_+(0) = 0.9704 (24) (22)$ (FNAL/MILC 13E), is based on the use of the Highly Improved Staggered Quark (HISQ) action (for both valence and sea quarks), which has been taylored to reduce staggered taste-breaking effects, and includes simulations with three lattice spacings and physical light-quark masses. These features allow one to keep the uncertainties due to the chiral extrapolation and to the discretization artefacts well below the statistical error. The remaining largest systematic uncertainty comes from finite-size effects.

At $N_{ f}=2+1$ there is a new result from the RBC/UKQCD Collaboration, $f_+(0) = 0.9685 (34) (14)$ [24] (RBC/UKQCD 15A), which satisfies all FLAG criteria for entering the average. RBC/UKQCD 15A superseeds RBC/UKQCD 13 thanks to two new simulations at the physical point. The other result eligible to enter the FLAG average at $N_{ f}=2+1$ is the one from FNAL/MILC 12I, $f_+(0)=0.9667(23)(33)$. The two results, based on different fermion discretizations (staggered fermions in the case of FNAL/MILC and domain-wall fermions in the case of RBC/UKQCD) are in nice agreement. Moreover, in the case of FNAL/MILC the form factor has been determined from the scalar current matrix element, while in the case of RBC/UKQCD it has been determined including also the matrix element of the vector current. To a certain extent both simulations are expected to be affected by different systematic effects.

RBC/UKQCD 15A has analysed results on ensembles with pion masses down to 140 MeV, mapping out the complete range from the *SU*(3)-symmetric limit to the physical point. No significant cutoff effects (results for two lattice spacings) were observed in the simulation results. Ensembles with unphysical light-quark masses are weighted to work as a guide for small corrections toward the physical point, reducing in this way the model dependence in the fitting ansatz. The systematic uncertainty turns out to be dominated by finite-volume effects, for which an estimate based on effective-theory arguments is provided.

The result FNAL/MILC 12I is from simulations reaching down to a lightest RMS pion mass of about 380 MeV (the lightest valence pion mass for one of their ensembles is about 260 MeV). Their combined chiral and continuum extrapolation (results for two lattice spacings) is based on NLO staggered chiral perturbation theory supplemented by the continuum NNLO expression [227] and a phenomenological parameterization of the breaking of the Ademollo–Gatto theorem at finite-lattice spacing inherent in their approach. The $p^4$ low-energy constants entering the NNLO expression have been fixed in terms of external input [130].

The ETM Collaboration uses the twisted-mass discretization and provides at $N_{ f}=2$ a comprehensive study of the systematics [25, 215], by presenting results for four lattice spacings and by simulating at light pion masses (down to $M_\pi = 260$ MeV). This makes it possible to constrain the chiral extrapolation, using both *SU*(3) [221] and *SU*(2) [223] chiral perturbation theory. Moreover, a rough estimate for the size of the effects due to quenching the strange quark is given, based on the comparison of the result for $N_{ f}=2$ dynamical quark flavours [32] with the one in the quenched approximation, obtained earlier by the SPQcdR Collaboration [244].

We now compute the $N_f =2+1$ FLAG average for $f_+(0)$ based on FNAL/MILC 12I and RBC/UKQCD 15A, which we consider uncorrelated, while for $N_f = 2+1+1$ and $N_f = 2$ we consider directly the FNAL/MILC 13E and ETM 09A results, respectively:

58 $$\begin{aligned}&\hbox {direct},\,N_{ f}=2+1+1:&f_+(0)&= 0.9704(24)(22)\quad \,\mathrm {Ref.}~[22],\end{aligned}$$

59 $$\begin{aligned}&\hbox {direct},\,N_{ f}=2+1:&f_+(0)&= 0.9677(27) \quad \,\mathrm {Refs.}~[23,24], \end{aligned}$$

60 $$\begin{aligned}&\hbox {direct},\,N_{ f}=2:&f_+(0)&= 0.9560(57)(62)\quad \,\mathrm {Ref.}~[25], \end{aligned}$$

where the brackets in the first and third lines indicate the statistical and systematic errors, respectively. We stress that the results (58) and (59), corresponding to $N_f = 2+1+1$ and $N_f = 2+1$ respectively, include already simulations with physical light-quark masses.

In the case of the ratio of decay constants the datasets that meet the criteria formulated in the introduction are HPQCD 13A [26], FNAL/MILC 14A [14] (which updates MILC 13A [231]) and ETM 14E [27] with $N_f=2+1+1$, MILC 10 [29], BMW 10 [30], HPQCD/UKQCD 07 [28] and RBC/UKQCD 12 [31] (which is an update of RBC/UKQCD 10A [144]) with $N_{ f}=2+1$ and ETM 09 [32] with $N_{ f}=2$ dynamical flavours.

ETM 14E uses the twisted-mass discretization and provides a comprehensive study of the systematics by presenting results for three lattice spacings in the range 0.06–0.09 fm and for pion masses in the range 210–450 MeV. This makes it possible to constrain the chiral extrapolation, using both *SU*(2) [223] chiral perturbation theory and polynomial fits. The ETM Collaboration always includes the spread in the central values obtained from different ansätze into the systematic errors. The final result of their analysis is ${f_{K^\pm }}/{f_{\pi ^\pm }}= 1.184(12)_\mathrm{stat+fit}(3)_\mathrm{Chiral}(9)_\mathrm{a^2}(1)_{Z_P}(3)_{FV}(3)_{IB}$ where the errors are (statistical + the error due to the fitting procedure), due to the chiral extrapolation, the continuum extrapolation, the mass-renormalization constant, the finite-volume and (strong) isospin-breaking effects.

FNAL/MILC 14A has determined the ratio of the decay constants from a comprehensive set of HISQ ensembles with $N_f = 2+1+1$ dynamical flavours. They have generated ensembles for four values of the lattice spacing ($0.06{-}0.15$ fm, scale set with $f_{\pi ^+}$) and with both physical and unphysical values of the light sea-quark masses, controlling in this way the systematic uncertainties due to chiral and continuum extrapolations. With respect to MILC 13A they have increased the statistics and added an important ensemble at the finest lattice spacing and for physical values of the light-quark mass. The final result of their analysis is ${f_{K^\pm }}/{f_{\pi ^\pm }}=1.1956(10)_\mathrm{stat}(_{-14}^{+23})_\mathrm{a^2} (10)_{FV} (5)_{EM}$ where the errors are statistical, due to the continuum extrapolation, finite-volume and electromagnetic effects. With respect to MILC 13A a factor of ${\simeq } 2.6,~ 1.8$ and $\simeq 1.7$ has been gained for the statistical, the discretization and the finite-volume errors.

HPQCD 13A analyses ensembles generated by MILC and therefore its study of ${f_{K^\pm }}/{f_{\pi ^\pm }}$ is based on the same set of ensembles bar the one for the finest lattice spacing ($a = 0.09{-}0.15$ fm, scale set with $f_{\pi ^+}$ and relative scale set with the Wilson flow [245, 246]) supplemented by some simulation points with heavier quark masses. HPQCD employs a global fit based on continuum NLO *SU*(3) chiral perturbation theory for the decay constants supplemented by a model for higher-order terms including discretization and finite-volume effects (61 parameters for 39 data points supplemented by Bayesian priors). Their final result is $f_{K^\pm }/f_{\pi ^\pm }=1.1916(15)_\mathrm{stat}(12)_\mathrm{a^2}(1)_{FV}(10)$, where the errors are statistical, due to the continuum extrapolation, due to finite-volume effects and the last error contains the combined uncertainties from the chiral extrapolation, the scale-setting uncertainty, the experimental input in terms of $f_{\pi ^+}$ and from the uncertainty in $m_u/m_d$.

In the previous edition of the FLAG review [2] the error budget of HPQCD 13A was compared with the one of MILC 13A and discussed in detail. It was pointed out that, despite the large overlap in primary lattice data, both collaborations arrive at surprisingly different error budgets. The same still holds when the comparison is made between HPQCD 13A and FNAL/MILC 14A.

Concerning the cutoff dependence, the finest lattice included into MILC’s analysis is $a = 0.06$ fm while the finest lattice in HPQCD’s case is $a = 0.09$ fm and both collaborations allow for taste-breaking terms in their analyses. MILC estimates the residual systematic after extrapolating to the continuum limit by taking the split between the result of an extrapolation with up to quartic and only up to quadratic terms in *a* as their systematic. HPQCD on the other hand models cutoff effects within their global fit ansatz up to including terms in $a^8$, using priors for the unknown coefficients and without including the spread in the central values obtained from different ansätze into the systematic errors. In this way HPQCD arrives at a systematic error due to the continuum limit which is smaller than MILC’s estimate by about a factor ${\simeq } 1.8$.

Turning to finite-volume effects, NLO staggered chiral perturbation theory (MILC) or continuum chiral perturbation theory (HPQCD) was used for correcting the lattice data towards the infinite-volume limit. MILC then compared the finite-volume correction to the one obtained by the NNLO expression and took the difference as their estimate for the residual finite-volume error. In addition they checked the compatibility of the effective-theory predictions (NLO continuum, staggered and NNLO continuum chiral perturbation theory) against lattice data of different spacial extent. The final verdict is that the related residual systematic uncertainty on ${f_{K^\pm }}/{f_{\pi ^\pm }}$ made by MILC is larger by an order of magnitude than the one made by HPQCD.

Adding in quadrature all the uncertainties one gets $f_{K^\pm }/f_{\pi ^\pm } = 1.1916(22)$ (HPQCD 13A) and ${f_{K^\pm }}/{f_{\pi ^\pm }}=1.1960(24)$ 18 (FNAL/MILC 14A). It can be seen that the total errors turn out to be very similar, but the central values seem to show a slight tension of about two standard deviations. While FLAG is looking forward to independent confirmations of the result for ${f_{K^\pm }}/{f_{\pi ^\pm }}$ at the same level of precision, we evaluate the FLAG average using a two-step procedure. First, the HPQCD 13A and FNAL/MILC 14A are averaged assuming a $100 \%$ statistical correlation, obtaining ${f_{K^\pm }}/{f_{\pi ^\pm }}=1.1936(29)$, where, following the prescription of Sect. 2.3, the error has been inflated by the factor $\sqrt{(\chi ^2/\mathrm{d.o.f.})} \simeq \sqrt{2.5}$ as a result of the tension between the two central values. Then, the above finding is averaged with the (uncorrelated) ETM 14E result, obtaining

61 $$\begin{aligned}&\hbox {direct},\,N_{ f}=2+1+1: \quad {f_{K^\pm }}/{f_{\pi ^\pm }}=1.1933(29)\nonumber \\&\quad \,\mathrm {Refs.}~ [14,26,27]. \end{aligned}$$

For both $N_f=2+1$ and $N_f=2$ no new result enters the corresponding FLAG averages with respect to the previous edition of the FLAG review [2] and before the closing date specified in Sect. 1. Here we limit ourselves to note that for $N_f=2+1$ MILC 10 and HPQCD/UKQCD 07 are based on staggered fermions, BMW 10 has used improved Wilson fermions and RBC/UKQCD 12’s result is based on the domain-wall formulation. Concerning simulations with $N_f=2$ the FLAG average remains the ETM 09 result, which has simulated twisted-mass fermions. In contrast to FNAL/MILC 14A all these simulations are for unphysical values of the light-quark masses (corresponding to smallest pion masses in the range $240{-}260$ MeV in the case of MILC 10, HPQCD/UKQCD 07 and ETM 09 and around 170 MeV for RBC/UKQCD 12) and therefore slightly more sophisticated extrapolations needed to be controlled. Various ansätze for the mass and cutoff dependence comprising *SU*(2) and *SU*(3) chiral perturbation theory or simply polynomials were used and compared in order to estimate the model dependence. While BMW 10 and RBC/UKQCD 12 are entirely independent computations, subsets of the MILC gauge ensembles used by MILC 10 and HPQCD/UKQCD 07 are the same. MILC 10 is certainly based on a larger and more advanced set of gauge configurations than HPQCD/UKQCD 07. This allows them for a more reliable estimation of systematic effects. In this situation we consider only their statistical but not their systematic uncertainties to be correlated.

Before determining the average for $f_{K^\pm }/f_{\pi ^\pm }$, which should be used for applications to Standard-Model phenomenology, we apply the isospin correction individually to all those results which have been published in the isospin-symmetric limit, i.e. BMW 10, HPQCD/UKQCD 07 and RBC/UKQCD 12 at $N_f = 2+1$ and ETM 09 at $N_f = 2$. To this end, as in the previous edition of the FLAG review [2], we make use of NLO *SU*(3) chiral perturbation theory [129, 247], which predicts

62 $$\begin{aligned} \frac{f_{K^\pm }}{f_{\pi ^\pm }}= \frac{f_K}{f_\pi } ~ \sqrt{1 + \delta _{SU(2)}}, \end{aligned}$$

where [247]

63 $$\begin{aligned} \delta _{SU(2)}\approx & {} \sqrt{3}\,\epsilon _{SU(2)} \left[ -\frac{4}{3} \left( f_K/f_\pi -1\right) +\frac{2}{3 (4\pi )^2 f_0^2} \right. \nonumber \\&\times \left. \left( M_K^2-M_\pi ^2-M_\pi ^2\ln \frac{M_K^2}{M_\pi ^2}\right) \right] . \end{aligned}$$

We use as input $\epsilon _{SU(2)}=\sqrt{3}/4/R$ with the FLAG result for *R* of Eq. (36), $F_0=f_0/\sqrt{2}=80(20)$ MeV, $M_\pi =135$ MeV and $M_K=495$ MeV (we decided to choose a conservative uncertainty on $f_0$ in order to reflect the magnitude of potential higher-order corrections). The results are reported in Table 15, where in the last column the first error is statistical and the second error is due to the isospin correction (the remaining errors are quoted in the same order as in the original data).

**Table 15:** Values of the *SU*(2) isospin-breaking correction $\delta _{SU(2)}$ applied to the lattice data for $f_K/f_\pi$, entering the FLAG average at $N_f=2+1$, for obtaining the corrected charged ratio $f_{K^\pm }/f_{\pi ^\pm }$

	$f_K/f_\pi$	$\delta _{SU(2)}$	$f_{K^\pm }/f_{\pi ^\pm }$
HPQCD/UKQCD 07	1.189(2)(7)	$-$0.0040(7)	1.187(2)(2)(7)
BMW 10	1.192(7)(6)	$-$0.0041(7)	1.190(7)(2)(6)
RBC/UKQCD 12	1.199(12)(14)	$-$0.0043(9)	1.196(12)(2)(14)

For $N_f=2$ a dedicated study of the strong-isospin correction in lattice QCD does exist. The (updated) result of the RM123 Collaboration [16] amounts to $\delta _{SU(2)}=-0.0080(4)$ and we use this result for the isospin correction of the ETM 09 result at $N_f=2$.

Note that the RM123 value for the strong-isospin correction is almost incompatible with the results based on *SU*(3) chiral perturbation theory, $\delta _{SU(2)}=-0.004(1)$ (see Table 15). Moreover, for $N_f=2+1+1$ HPQCD 13A [26] and ETM 14E [27] estimate a value for $\delta _{SU(2)}$ equal to $-0.0054(14)$ and $-0.0080(38)$, respectively. One would not expect the strange and heavier sea-quark contributions to be responsible for such a large effect. Whether higher-order effects in chiral perturbation theory or other sources are responsible still needs to be understood. More lattice QCD simulations of *SU*(2) isospin-breaking effects are therefore required. To remain on the conservative side we add a $100 \%$ error to the correction based on *SU*(3) chiral perturbation theory. For further analyses we add (in quadrature) such an uncertainty to the systematic error.

Using the results of Table 15 for $N_f = 2+1$ we obtain

64 $$\begin{aligned}&\hbox {direct},\,N_{ f}=2+1+1:f_{K^\pm } / f_{\pi ^\pm } = 1.193(3)\nonumber \\&\quad \,\mathrm {Refs.}~[14,26,27], \end{aligned}$$

65 $$\begin{aligned}&\hbox {direct},\,N_{ f}=2+1: f_{K^\pm } / f_{\pi ^\pm } = 1.192(5)\nonumber \\&\quad \,\mathrm {Refs.}~[28{-}31], \end{aligned}$$

66 $$\begin{aligned}&\hbox {direct},\,N_{ f}=2: f_{K^\pm } / f_{\pi ^\pm } = 1.205(6)(17)\nonumber \\&\quad \,\mathrm {Ref.}~[32], \end{aligned}$$

for QCD with broken isospin.

**Fig. 8:**
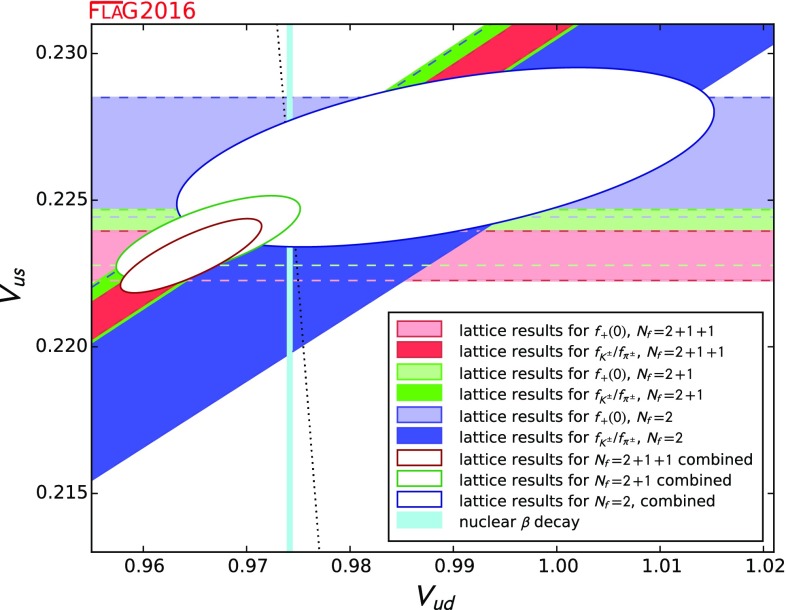
The plot compares the information for $|V_{ud}|$, $|V_{us}|$ obtained on the lattice with the experimental result extracted from nuclear $\beta$ transitions. The *dotted line* indicates the correlation between $|V_{ud}|$ and $|V_{us}|$ that follows if the CKM-matrix is unitary

It is instructive to convert the above results for $f_+(0)$ and ${f_{K^\pm }}/{f_{\pi ^\pm }}$ into a corresponding range for the CKM matrix elements $|V_{ud}|$ and $|V_{us}|$, using the relations (54). Consider first the results for $N_{ f}=2+1+1$. The range for $f_+(0)$ in Eq. (58) is mapped into the interval $|V_{us}|=0.2231(9)$, depicted as a horizontal red band in Fig. 8, while the one for ${f_{K^\pm }}/{f_{\pi ^\pm }}$ in Eq. (64) is converted into $|V_{us}|/|V_{ud}|= 0.2313(7)$, shown as a tilted red band. The red ellipse is the intersection of these two bands and represents the 68% likelihood contour,^19^ obtained by treating the above two results as independent measurements. Repeating the exercise for $N_{ f}=2+1$ and $N_{ f}=2$ leads to the green and blue ellipses, respectively. The plot indicates a slight tension between the $N_f=2+1+1$ and the nuclear $\beta$ decay results.

### Tests of the Standard Model

In the Standard Model, the CKM matrix is unitary. In particular, the elements of the first row obey

67 $$\begin{aligned} |V_u|^2\equiv |V_{ud}|^2 + |V_{us}|^2 + |V_{ub}|^2 = 1.\end{aligned}$$

The tiny contribution from $|V_{ub}|$ is known much better than needed in the present context: $|V_{ub}|= 4.13 (49) \times 10^{-3}$ [151]. In the following, we first discuss the evidence for the validity of the relation (67) and only then use it to analyse the lattice data within the Standard Model.

In Fig. 8, the correlation between $|V_{ud}|$ and $|V_{us}|$ imposed by the unitarity of the CKM matrix is indicated by a dotted line (more precisely, in view of the uncertainty in $|V_{ub}|$, the correlation corresponds to a band of finite width, but the effect is too small to be seen here). The plot shows that there is a slight tension with unitarity in the data for $N_f = 2 + 1 + 1$: Numerically, the outcome for the sum of the squares of the first row of the CKM matrix reads $|V_u|^2 = 0.980(9)$, which deviates from unity at the level of two standard deviations. Still, it is fair to say that at this level the Standard Model passes a nontrivial test that exclusively involves lattice data and well-established kaon decay branching ratios. Combining the lattice results for $f_+(0)$ and ${f_{K^\pm }}/{f_{\pi ^\pm }}$ in Eqs. (58) and (64) with the $\beta$ decay value of $|V_{ud}|$ quoted in Eq. (55), the test sharpens considerably: the lattice result for $f_+(0)$ leads to $|V_u|^2 = 0.9988(6)$, which highlights again a $2\sigma$-tension with unitarity, while the one for ${f_{K^\pm }}/{f_{\pi ^\pm }}$ implies $|V_u|^2 = 0.9998(5)$, confirming the first-row CKM unitarity below the permille level.

**Fig. 9:**
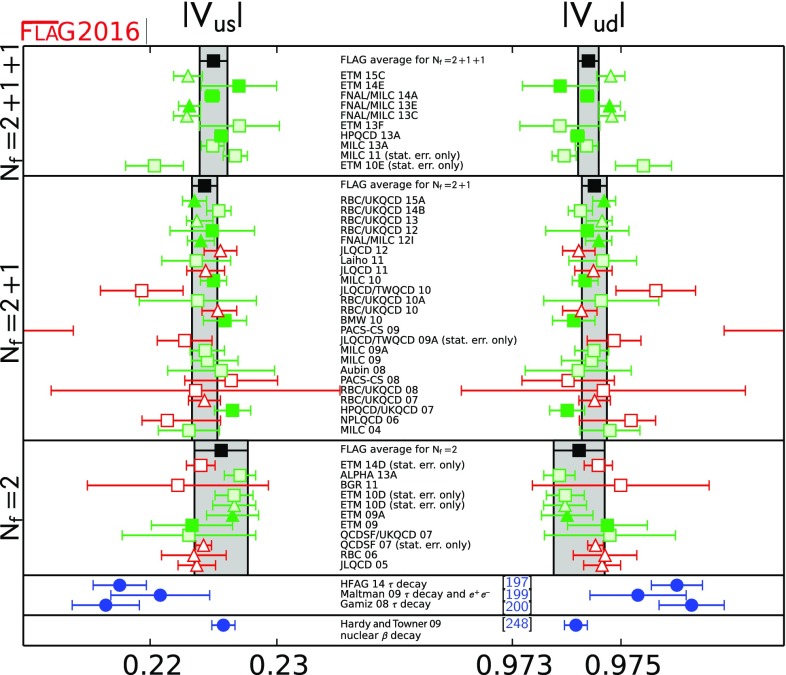
Results for $|V_{us}|$ and $|V_{ud}|$ that follow from the lattice data for $f_+(0)$ (*triangles*) and ${f_{K^\pm }}/{f_{\pi ^\pm }}$ (*squares*), on the basis of the assumption that the CKM matrix is unitary. The *black squares* and the *grey bands* represent our estimates, obtained by combining these two different ways of measuring $|V_{us}|$ and $|V_{ud}|$ on a lattice. For comparison, the figure also indicates the results obtained if the data on nuclear $\beta$ decay and $\tau$ decay are analysed within the Standard Model

The situation is similar for $N_{ f}=2+1$: $|V_u|^2 = 0.984(11)$ with the lattice data alone. Combining the lattice results for $f_+(0)$ and ${f_{K^\pm }}/{f_{\pi ^\pm }}$ in Eqs. (59) and (65) with the $\beta$ decay value of $|V_{ud}|$, the test sharpens again considerably: the lattice result for $f_+(0)$ leads to $|V_u|^2 = 0.9991(6)$, while the one for ${f_{K^\pm }}/{f_{\pi ^\pm }}$ implies $|V_u|^2 = 0.9999(6)$, thus confirming again CKM unitarity below the permille level.

Repeating the analysis for $N_f = 2$, we find $|V_u|^2 = 1.029(34)$ with the lattice data alone. This number is fully compatible with unity and perfectly consistent with the value of $|V_{ud}|$ found in nuclear $\beta$ decay: combining this value with the result (60) for $f_+(0)$ yields $|V_u|^2=1.0003(10)$, combining it with the data (66) on ${f_{K^\pm }}/{f_{\pi ^\pm }}$ gives $|V_u|^2= 0.9988(15)$.

**Table 16:** Values of $|V_{us}|$ and $|V_{ud}|$ obtained from the lattice determinations of either $f_+(0)$ or ${f_{K^\pm }}/{f_{\pi ^\pm }}$ assuming CKM unitarity. The first (second) number in brackets represents the statistical (systematic) error

Collaboration	Refs.	$N_{ f}$	From	$|V_{us}|$	$|V_{ud}|$
FNAL/MILC 13E	[22]	$2+1+1$	$f_+(0)$	0.2231(7)(5)	0.97479(16)(12)
ETM 14E	[27]	$2+1+1$	${f_{K^\pm }}/{f_{\pi ^\pm }}$	0.2270(22)(20)	0.97388(51)(47)
FNAL/MILC 14A	[14]	$2+1+1$	${f_{K^\pm }}/{f_{\pi ^\pm }}$	0.2249(4)(4)	0.97438(8)(9)
HPQCD 13A	[26]	$2+1+1$	${f_{K^\pm }}/{f_{\pi ^\pm }}$	0.2256(4)(3)	0.97420(10)(7)
RBC/UKQCD 15A	[24]	$2+1$	$f_+(0)$	0.2235(9)(3)	0.97469(20)(7)
FNAL/MILC 12I	[23]	$2+1$	$f_+(0)$	0.2240(7)(8)	0.97459(16)(18)
MILC 10	[29]	$2+1$	${f_{K^\pm }}/{f_{\pi ^\pm }}$	0.2250(5)(9)	0.97434(11)(21)
RBC/UKQCD 12	[144]	$2+1$	${f_{K^\pm }}/{f_{\pi ^\pm }}$	0.2249(22)(25)	0.97438(50)(58)
BMW 10	[30]	$2+1$	${f_{K^\pm }}/{f_{\pi ^\pm }}$	0.2259(13)(11)	0.97413(30)(25)
HPQCD/UKQCD 07	[28]	$2+1$	${f_{K^\pm }}/{f_{\pi ^\pm }}$	0.2265(6)(13)	0.97401(14)(29)
ETM 09A	[25]	2	$f_+(0)$	0.2265(14) (15)	0.97401(33)(34)
ETM 09	[32]	2	${f_{K^\pm }}/{f_{\pi ^\pm }}$	0.2233(11) (30)	0.97475(25)(69)

Note that the above tests also offer a check of the basic hypothesis that underlies our analysis: we are assuming that the weak interaction between the quarks and the leptons is governed by the same Fermi constant as the one that determines the strength of the weak interaction among the leptons and determines the lifetime of the muon. In certain modifications of the Standard Model, this is not the case. In those models it need not be true that the rates of the decays $\pi \rightarrow \ell \nu$, $K\rightarrow \ell \nu$ and $K\rightarrow \pi \ell \nu$ can be used to determine the matrix elements $|V_{ud}f_\pi |$, $|V_{us}f_K|$ and $|V_{us}f_+(0)|$, respectively and that $|V_{ud}|$ can be measured in nuclear $\beta$ decay. The fact that the lattice data are consistent with unitarity and with the value of $|V_{ud}|$ found in nuclear $\beta$ decay indirectly also checks the equality of the Fermi constants.

### Analysis within the Standard Model

The Standard Model implies that the CKM matrix is unitary. The precise experimental constraints quoted in (54) and the unitarity condition (67) then reduce the four quantities $|V_{ud}|,|V_{us}|,f_+(0), {f_{K^\pm }}/{f_{\pi ^\pm }}$ to a single unknown: any one of these determines the other three within narrow uncertainties.

Figure 9 shows that the results obtained for $|V_{us}|$ and $|V_{ud}|$ from the data on ${f_{K^\pm }}/{f_{\pi ^\pm }}$ (squares) are quite consistent with the determinations via $f_+(0)$ (triangles). In order to calculate the corresponding average values, we restrict ourselves to those determinations that we have considered best in Sect. 4.3. The corresponding results for $|V_{us}|$ are listed in Table 16 (the error in the experimental numbers used to convert the values of $f_+(0)$ and ${f_{K^\pm }}/{f_{\pi ^\pm }}$ into values for $|V_{us}|$ is included in the statistical error).

**Table 17:** The upper half of the table shows our final results for $|V_{us}|$, $|V_{ud}|$, $f_+(0)$ and ${f_{K^\pm }}/{f_{\pi ^\pm }}$, which are obtained by analysing the lattice data within the Standard Model. For comparison, the lower half lists the values that follow if the lattice results are replaced by the experimental results on nuclear $\beta$ decay and $\tau$ decay, respectively

	Refs.	$|V_{us}|$	$|V_{ud}|$	$f_+(0)$	${f_{K^\pm }}/{f_{\pi ^\pm }}$
$N_{ f}= 2+1+1$		0.2250(11)	0.97440(19)	0.9622(50)	1.195(5)
$N_{ f}= 2+1$		0.2243(10)	0.97451(23)	0.9652(47)	1.199(5)
$N_{ f}=2$		0.2256(21)	0.97423(47)	0.9597(91)	1.192(9)
$\beta$ Decay	[186]	0.2258(9)	0.97417(21)	0.9588(42)	1.191(4)
$\tau$ Decay	[200]	0.2165(26)	0.9763(6)	1.0000(122)	1.245(12)
$\tau$ Decay	[199]	0.2208(39)	0.9753(9)	0.9805(174)	1.219(18)

**Table 18:** Colour code for the lattice data on $f_{\pi ^\pm }$ and $f_{K^\pm }$ together with information on the way the lattice spacing was converted to physical units and on whether or not an isospin-breaking correction has been applied to the quoted result (see Sect. 4.3). The numerical values are listed in MeV units

Collaboration	Refs.	$N_{ f}$	Publication status	Chiral extrapolation	Continuum extrapolation	Finite-volume errors	Renormalization	Physical scale	*SU*(2) breaking	$f_{\pi ^\pm }$	$f_{K^\pm }$
ETM 14E	[27]	$2+1+1$	A				na	$f_\pi$		–	154.4(1.5)(1.3)
FNAL/MILC 14A	[14]	$2+1+1$	A				na	$f_\pi$		–	155.92(13)($_{-23}^{+34}$)
HPQCD 13A	[26]	$2+1+1$	A				na	$f_\pi$		–	155.37(20)(27)
MILC 13A	[231]	$2+1+1$	A				na	$f_\pi$		–	155.80(34)(54)
ETM 10E	[233]	$2+1+1$	C				na	$f_\pi$	$\checkmark$	–	159.6(2.0)
RBC/UKQCD 14B	[10]	$2+1$	A				NPR	$m_\Omega$	$\checkmark$	130.19(89)	155.18(89)
RBC/UKQCD 12	[31]	$2+1$	A				NPR	$m_\Omega$	$\checkmark$	127.1(2.7)(2.7)	152.1(3.0)(1.7)
Laiho 11	[44]	$2+1$	C				na	${}^{\mathrm{a}}$		130.53(87)(210)	156.8(1.0)(1.7)
MILC 10	[29]	$2+1$	C				na	${}^{\mathrm{a}}$		129.2(4)(14)	–
MILC 10	[29]	$2+1$	C				na	$f_\pi$		–	156.1(4)($_{-9}^{+6}$)
JLQCD/TWQCD 10	[234]	$2+1$	C				na	$m_\Omega$	$\checkmark$	118.5(3.6)$_\mathrm{stat}$	145.7(2.7)$_\mathrm{stat}$
RBC/UKQCD 10A	[144]	$2+1$	A				NPR	$m_\Omega$	$\checkmark$	124(2)(5)	148.8(2.0)(3.0)
PACS-CS 09	[94]	$2+1$	A				NPR	$m_\Omega$	$\checkmark$	124.1(8.5)(0.8)	165.0(3.4)(1.1)
JLQCD/TWQCD 09A	[235]	$2+1$	C				na	$f_\pi$	$\checkmark$	–	156.9(5.5)$_\mathrm{stat}$
MILC 09A	[6]	$2+1$	C				na	$\Delta M_\Upsilon$		128.0(0.3)(2.9)	153.8(0.3)(3.9)
MILC 09A	[6]	$2+1$	C				na	$f_\pi$		–	156.2(0.3)(1.1)
MILC 09	[89]	$2+1$	A				na	$\Delta M_\Upsilon$		128.3(0.5)($^{+2.4}_{-3.5}$)	154.3(0.4)($^{+2.1}_{-3.4}$)
MILC 09	[89]	$2+1$	A				na	$f_\pi$			156.5(0.4)($^{+1.0}_{-2.7}$)
Aubin 08	[236]	$2+1$	C				na	$\Delta M_\Upsilon$		129.1(1.9)(4.0)	153.9(1.7)(4.4)
PACS-CS 08, 08A	[93, 237]	$2+1$	A				1lp	$m_\Omega$	$\checkmark$	134.0(4.2)$_\mathrm{stat}$	159.0(3.1)$_\mathrm{stat}$
RBC/UKQCD 08	[145]	$2+1$	A				NPR	$m_\Omega$	$\checkmark$	124.1(3.6)(6.9)	149.4(3.6)(6.3)
HPQCD/UKQCD 07	[28]	$2+1$	A				na	$\Delta M_\Upsilon$	$\checkmark$	132(2)	156.7(0.7)(1.9)
MILC 04	[107]	$2+1$	A				na	$\Delta M_\Upsilon$		129.5(0.9)(3.5)	156.6(1.0)(3.6)
ETM 14D	[160]	2	C				na	$f_\pi$	$\checkmark$	–	153.3(7.5)$_\mathrm{stat}$
TWQCD 11	[249]	2	P				na	${r_0}^{\mathrm{c}}$		127.3(1.7)(2.0)$^{\mathrm{d}}$	–
ETM 09	[32]	2	A				na	$f_\pi$	$\checkmark$	–	157.5(0.8)(2.0)(1.1)$^{\mathrm{b}}$
JLQCD/TWQCD 08A	[138]	2	A				na	$r_0$		119.6(3.0)($^{+6.5}_{-1.0}$)$^{\mathrm{d}}$	–

The label ‘na’ indicates the lattice calculations which do not require the use of any renormalization constant for the axial current, while the label ‘NPR’ (‘1lp’) signals the use of a renormalization constant calculated nonperturbatively (at one-loop order in perturbation theory)

$^{\mathrm{a}}$ The ratios of lattice spacings within the ensembles were determined using the quantity $r_1$. The conversion to physical units was made on the basis of Ref. [250] and we note that such a determination depends on the experimental value of the pion decay constant

$^{\mathrm{b}}$ Errors are (stat $+$ chiral)($a\ne 0$)(finite size)

$^{\mathrm{c}}$ The ratio $f_\pi /M_\pi$ was used as experimental input to fix the light-quark mass

$^{\mathrm{d}}$ $L_\mathrm{min}<2$ fm in these simulations

For $N_{ f}=2+1+1$ we consider the data both for $f_+(0)$ and ${f_{K^\pm }}/{f_{\pi ^\pm }}$, treating FNAL/MILC 13E, FNAL/MILC 14A and HPQCD 13A as statistically correlated (according to the prescription of Sect. 2.3). We obtain $|V_{us}|=0.2250(11)$, where the error includes the inflation factor due the value of $\chi ^2/\mathrm{d.o.f.} \simeq 2.3$. This result is indicated on the left hand side of Fig. 9 by the narrow vertical band. In the case $N_f = 2+1$ we consider MILC 10, FNAL/MILC 12I and HPQCD/UKQCD 07 on the one hand and RBC/UKQCD 12 and RBC/UKQCD 15A on the other hand, as mutually statistically correlated, since the analysis in the two cases starts from partly the same set of gauge ensembles. In this way we arrive at $|V_{us}| = 0.2243(10)$ with $\chi ^2/\mathrm{d.o.f.} \simeq 1.0$. For $N_{ f}=2$ we consider ETM 09A and ETM 09 as statistically correlated, obtaining $|V_{us}|=0.2256(21)$ with $\chi ^2/\mathrm{d.o.f.} \simeq 0.7$. The figure shows that the result obtained for the data with $N_{ f}=2$, $N_{ f}=2+1$ and $N_{ f}=2+1+1$ are consistent with each other.

Alternatively, we can solve the relations for $|V_{ud}|$ instead of $|V_{us}|$. Again, the result $|V_{ud}|=0.97440(19)$, which follows from the lattice data with $N_{ f}=2+1+1$, is perfectly consistent with the values $|V_{ud}|=0.97451(23)$ and $|V_{ud}|=0.97423(47)$ obtained from the data with $N_{ f}=2+1$ and $N_{ f}=2$, respectively. The reduction of the uncertainties in the result for $|V_{ud}|$ due to CKM unitarity is to be expected from Fig. 8: the unitarity condition reduces the region allowed by the lattice results to a nearly vertical interval.

Next, we determine the values of $f_+(0)$ and ${f_{K^\pm }}/{f_{\pi ^\pm }}$ that follow from our determinations of $|V_{us}|$ and $|V_{ud}|$ obtained from the lattice data within the Standard Model. We find $f_+(0) = 0.9622(50)$ for $N_{ f}=2+1+1$, $f_+(0) = 0.9652(47)$ for $N_{ f}=2+1$, $f_+(0) = 0.9597(91)$ for $N_{ f}=2$ and ${f_{K^\pm }}/{f_{\pi ^\pm }}= 1.195(5)$ for $N_{ f}=2+1+1$, ${f_{K^\pm }}/{f_{\pi ^\pm }}= 1.199(5)$ for $N_{ f}=2+1$, ${f_{K^\pm }}/{f_{\pi ^\pm }}= 1.192(9)$ for $N_{ f}=2$, respectively. These results are collected in the upper half of Table 17. In the lower half of the table, we list the analogous results found by working out the consequences of the CKM unitarity using the values of $|V_{ud}|$ and $|V_{us}|$ obtained from nuclear $\beta$ decay and $\tau$ decay, respectively. The comparison shows that the lattice result for $|V_{ud}|$ not only agrees very well with the totally independent determination based on nuclear $\beta$ transitions, but it is also remarkably precise. On the other hand, the values of $|V_{ud}|$, $f_+(0)$ and ${f_{K^\pm }}/{f_{\pi ^\pm }}$ which follow from the $\tau$-decay data if the Standard Model is assumed to be valid, are not in good agreement with the lattice results for these quantities. The disagreement is reduced considerably if the analysis of the $\tau$ data is supplemented with experimental results on electroproduction [199]: the discrepancy then amounts to little more than one standard deviation.

### Direct determination of $f_{K^\pm }$ and $f_{\pi ^\pm }$

It is useful for flavour physics studies to provide not only the lattice average of $f_{K^\pm } / f_{\pi ^\pm }$, but also the average of the decay constant $f_{K^\pm }$. The case of the decay constant $f_{\pi ^\pm }$ is different, since the experimental value of this quantity is often used for setting the scale in lattice QCD (see Appendix A.2). However, the physical scale can be set in different ways, namely by using as input the mass of the $\Omega$-baryon ($m_\Omega$) or the $\Upsilon$-meson spectrum ($\Delta M_\Upsilon$), which are less sensitive to the uncertainties of the chiral extrapolation in the light-quark mass with respect to $f_{\pi ^\pm }$. In such cases the value of the decay constant $f_{\pi ^\pm }$ becomes a direct prediction of the lattice-QCD simulations. It is therefore interesting to provide also the average of the decay constant $f_{\pi ^\pm }$, obtained when the physical scale is set through another hadron observable, in order to check the consistency of different scale-setting procedures.

Our compilation of the values of $f_{\pi ^\pm }$ and $f_{K^\pm }$ with the corresponding colour code is presented in Table 18. With respect to the case of $f_{K^\pm } / f_{\pi ^\pm }$ we have added two columns indicating which quantity is used to set the physical scale and the possible use of a renormalization constant for the axial current. Indeed, for several lattice formulations the use of the nonsinglet axial-vector Ward identity allows one to avoid the use of any renormalization constant.

One can see that the determinations of $f_{\pi ^\pm }$ and $f_{K^\pm }$ suffer from larger uncertainties with respect to the ones of the ratio $f_{K^\pm } / f_{\pi ^\pm }$, which is less sensitive to various systematic effects (including the uncertainty of a possible renormalization constant) and, moreover, is not exposed to the uncertainties of the procedure used to set the physical scale.

According to the FLAG rules, for $N_f = 2 + 1 + 1$ three datasets can form the average of $f_{K^\pm }$ only: ETM 14E [27], FNAL/MILC 14A [14] and HPQCD 13A [26]. Following the same procedure already adopted in Sect. 4.3 in the case of the ratio of the decay constant we treat FNAL/MILC 14A and HPQCD 13A as statistically correlated. For $N_f = 2 + 1$ three datasets can form the average of $f_{\pi ^\pm }$ and $f_{K^\pm }$ : RBC/UKQCD 12 [31] (update of RBC/UKQCD 10A), HPQCD/UKQCD 07 [28] and MILC 10 [29], which is the latest update of the MILC program. We consider HPQCD/UKQCD 07 and MILC 10 as statistically correlated and use the prescription of Sect. 2.3 to form an average. For $N_f = 2$ the average cannot be formed for $f_{\pi ^\pm }$, and only one data set (ETM 09) satisfies the FLAG rules in the case of $f_{K^\pm }$.

Thus, our estimates read

68 $$\begin{aligned}&N_f = 2 + 1: f_{\pi ^\pm }= 130.2 ~ (1.4)~ \hbox {MeV}\nonumber \\&\quad \,\mathrm {Refs.}~[28,29,31],\end{aligned}$$

69 $$\begin{aligned}&N_f = 2 + 1 + 1: f_{K^\pm } = 155.6 ~ (0.4)~ \hbox {MeV}\nonumber \\&\quad \,\mathrm {Refs.}~[14,26,27] ,\nonumber \\&N_f = 2 + 1: f_{K^\pm } = 155.9 ~ (0.9)~ \hbox {MeV}\nonumber \\&\quad \,\mathrm {Refs.}~[28,29,31],\\&N_f = 2: f_{K^\pm } = 157.5 ~ (2.4)~ \hbox {MeV}\nonumber \\&\quad \,\mathrm {Ref.}~[32].\nonumber \end{aligned}$$

**Fig. 10:**
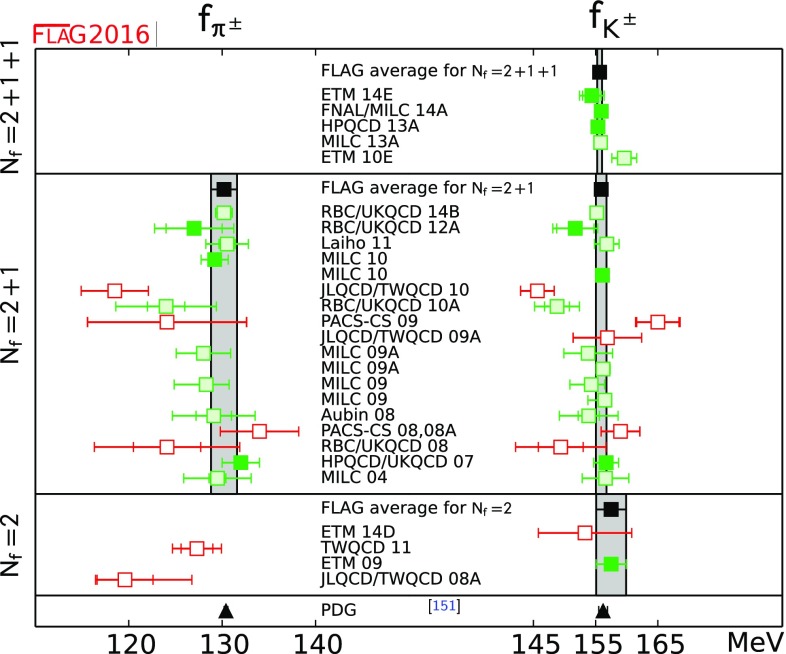
Values of $f_\pi$ and $f_K$. The *black squares* and *grey bands* indicate our estimates (68) and (69). The *black triangles* represent the experimental values quoted by the PDG; see Eq. (70)

The lattice results of Table 18 and our estimates (68)–(69) are reported in Fig. 10. The latter ones agree within the errors with the latest experimental determinations of $f_\pi$ and $f_K$ from the PDG [151]:

70 $$\begin{aligned}&f_{\pi ^\pm }^{(PDG)} = 130.41 ~ (0.20)~\hbox {MeV},\nonumber \\&\quad f_{K^\pm }^{(PDG)} = 156.2 ~ (0.7)~\hbox {MeV}. \end{aligned}$$

Moreover, the values of $f_{\pi ^\pm }$ and $f_{K^\pm }$ quoted by the PDG are obtained assuming Eq. (55) for the value of $|V_{ud}|$ and adopting the average of FNAL/MILC 12I and RBC-UKQCD 10 results for $f_+(0)$.

## Low-energy constants

In the study of the quark-mass dependence of QCD observables calculated on the lattice, it is common practice to invoke chiral perturbation theory ($\chi$PT). For a given quantity this framework predicts the nonanalytic quark-mass dependence and it provides symmetry relations among different observables. These relations are best expressed with the help of a set of linearly independent and universal (i.e. process-independent) low-energy constants (LECs), which appear as coefficients of the polynomial terms (in $m_q$ or $M_\pi ^2$) in different observables. When numerical simulations are done at heavier than physical (light) quark masses, $\chi$PT is usually invoked in the extrapolation to physical quark masses.

### Chiral perturbation theory

$\chi$PT is an effective field theory approach to the low-energy properties of QCD based on the spontaneous breaking of chiral symmetry, $SU(N_{ f})_L \times SU(N_{ f})_R \rightarrow SU(N_{ f})_{L+R}$, and its soft explicit breaking by quark-mass terms. In its original implementation, in infinite volume, it is an expansion in $m_q$ and $p^2$ with power counting $M_\pi ^2 \sim m_q \sim p^2$.

If one expands around the *SU*(2) chiral limit, there appear two LECs at order $p^2$ in the chiral effective Lagrangian,

71 $$\begin{aligned}&F\equiv F_\pi \,\Big |_{\;m_u,m_d\rightarrow 0} \quad \hbox {and} \quad B\equiv \frac{\Sigma }{F^2},\nonumber \\&\quad \hbox {where}\ \Sigma \equiv -\langle \overline{u}u\rangle \,\Big |_{\;m_u,m_d\rightarrow 0}, \end{aligned}$$

and seven at order $p^4$, indicated by $\bar{\ell }_i$ with $i=1,\ldots ,7$. In the analysis of the *SU*(3) chiral limit there are also just two LECs at order $p^2$,

72 $$\begin{aligned}&F_0\equiv F_\pi \,\Big |_{\;m_u,m_d,m_s\rightarrow 0} \quad \hbox {and} \quad B_0\equiv \frac{\Sigma _0}{F_0^2},\nonumber \\&\quad \hbox {where}\ \Sigma _0\equiv -\langle \overline{u}u\rangle \,\Big |_{\;m_u,m_d,m_s\rightarrow 0}, \end{aligned}$$

but ten at order $p^4$, indicated by the capital letter $L_i(\mu )$ with $i=1,\ldots ,10$. These constants are independent of the quark masses,^20^ but they become scale dependent after renormalization (sometimes a superscript *r* is added). The *SU*(2) constants $\bar{\ell }_i$ are scale independent, since they are defined at scale $\mu =M_\pi$ (as indicated by the bar). For the precise definition of these constants and their scale dependence we refer the reader to Refs. [129, 131].

If the box volume is finite but large compared to the Compton wavelength of the pion, $L \gg 1/M_\pi$, the power counting generalizes to $m_q \sim p^2 \sim 1/L^2$, as one would assume based on the fact that $p_\mathrm {min}=2\pi /L$ is the minimum momentum in a finite box. This is the so-called *p*-regime of $\chi$PT. It coincides with the setting that is used for standard phenomenologically oriented lattice-QCD computations, and we shall consider the *p*-regime the default in the following. However, if the pion mass is so small that the box-length *L* is no longer large compared to the Compton wavelength that the pion would have, at the given $m_q$, in infinite volume, then the chiral series must be reordered. Such finite-volume versions of $\chi$PT with correspondingly adjusted power-counting schemes, referred to as $\epsilon$- and $\delta$-regime, are described in Sects. 5.1.4 and 5.1.5, respectively.

Lattice calculations can be used to test if chiral symmetry is indeed spontaneously broken along the path $SU(N_{ f})_L \times SU(N_{ f})_R \rightarrow SU(N_{ f})_{L+R}$ by measuring nonzero chiral condensates and by verifying the validity of the GMOR relation $M_\pi ^2\propto m_q$ close to the chiral limit. If the chiral extrapolation of quantities calculated on the lattice is made with the help of fits to their $\chi$PT forms, apart from determining the observable at the physical value of the quark masses, one also obtains the relevant LECs. This is a very important by-product for two reasons:

1. All LECs up to order $p^4$ (with the exception of *B* and $B_0$, since only the product of these times the quark masses can be estimated from phenomenology) have either been determined by comparison to experiment or estimated theoretically, e.g. in large-$N_c$ QCD. A lattice determination of the better known LECs thus provides a test of the $\chi$PT approach.
2. The less well-known LECs are those which describe the quark-mass dependence of observables – these cannot be determined from experiment, and therefore the lattice, where quark masses can be varied, provides unique quantitative information. This information is essential for improving phenomenological $\chi$PT predictions in which these LECs play a role.

We stress that this program is based on the nonobvious assumption that $\chi$PT is valid in the region of masses and momenta used in the lattice simulations under consideration, something that can and should be checked. In the end one wants to compare lattice and phenomenological determinations of LECs, much in the spirit of Ref. [251]. An overview of many of the conceptual issues involved in matching lattice data to an effective field theory framework like $\chi$PT is given in Refs. [252–254].

The fact that, at large volume, the finite-size effects, which occur if a system undergoes spontaneous symmetry breakdown, are controlled by the Nambu–Goldstone modes, was first noted in solid state physics, in connection with magnetic systems [255, 256]. As pointed out in Ref. [257] in the context of QCD, the thermal properties of such systems can be studied in a systematic and model-independent manner by means of the corresponding effective field theory, provided the temperature is low enough. While finite volumes are not of physical interest in particle physics, lattice simulations are necessarily carried out in a finite box. As shown in Refs. [258–260], the ensuing finite-size effects can be studied on the basis of the effective theory – $\chi$PT in the case of QCD – provided the simulation is close enough to the continuum limit, the volume is sufficiently large and the explicit breaking of chiral symmetry generated by the quark masses is sufficiently small. Indeed, $\chi$PT represents a useful tool for the analysis of the finite-size effects in lattice simulations.

In the remainder of this subsection we collect the relevant $\chi$PT formulae that will be used in the two following subsections to extract *SU*(2) and *SU*(3) LECs from lattice data.

#### Quark-mass dependence of pseudoscalar masses and decay constants

A. *SU*(2) formulae

The expansions^21^ of $M_\pi ^2$ and $F_\pi$ in powers of the quark mass are known to next-to-next-to-leading order (NNLO) in the *SU*(2) chiral effective theory. In the isospin limit, $m_u=m_d=m$, the explicit expressions may be written in the form [261]

73 $$\begin{aligned} M_\pi ^2= & {} M^2\left\{ 1-\frac{1}{2}x\ln \frac{\Lambda _3^2}{M^2} +\frac{17}{8}x^2 \left( \ln \frac{\Lambda _M^2}{M^2} \right) ^2 \right. \nonumber \\&\quad \left. +x^2 k_M +\mathcal {O}(x^3) \right\} , \\ F_\pi= & {} F\left\{ 1+x\ln \frac{\Lambda _4^2}{M^2} -\frac{5}{4}x^2 \left( \ln \frac{\Lambda _F^2}{M^2} \right) ^2 \right. \nonumber \\&\quad \left. +x^2k_F +\mathcal {O}(x^3) \right\} . \nonumber \end{aligned}$$

Here the expansion parameter is given by

74 $$\begin{aligned} x=\frac{M^2}{(4\pi F)^2},\quad M^2=2Bm=\frac{2\Sigma m}{F^2}, \end{aligned}$$

but there is another option as discussed below. The scales $\Lambda _3,\Lambda _4$ are related to the effective coupling constants $\bar{\ell }_3,\bar{\ell }_4$ of the chiral Lagrangian at scale $M_\pi \equiv M_\pi ^\mathrm {phys}$ by

75 $$\begin{aligned} \bar{\ell }_n=\ln \frac{\Lambda _n^2}{M_\pi ^2},\quad n=1,\ldots ,7. \end{aligned}$$

Note that in Eq. (73) the logarithms are evaluated at $M^2$, not at $M_\pi ^2$. The coupling constants $k_M,k_F$ in Eq. (73) are mass-independent. The scales of the squared logarithms can be expressed in terms of the $\mathcal {O}(p^4)$ coupling constants as

76 $$\begin{aligned} \ln \frac{\Lambda _M^2}{M^2}= & {} \frac{1}{51}\left( 28\ln \frac{\Lambda _1^2}{M^2} +32\ln \frac{\Lambda _2^2}{M^2} -9 \ln \frac{\Lambda _3^2}{M^2}+49 \right) , \nonumber \\ \ln \frac{\Lambda _F^2}{M^2}= & {} \frac{1}{30}\left( 14\ln \frac{\Lambda _1^2}{M^2} +16\ln \frac{\Lambda _2^2}{M^2} \right. \nonumber \\&\quad \left. +6 \ln \frac{\Lambda _3^2}{M^2} - 6 \ln \frac{\Lambda _4^2}{M^2} +23 \right) . \end{aligned}$$

Hence by analysing the quark-mass dependence of $M_\pi ^2$ and $F_\pi$ with Eq. (73), possibly truncated at NLO, one can determine^22^ the $\mathcal {O}(p^2)$ LECs *B* and *F*, as well as the $\mathcal {O}(p^4)$ LECs $\bar{\ell }_3$ and $\bar{\ell }_4$. The quark condensate in the chiral limit is given by $\Sigma =F^2B$. With precise enough data at several small enough pion masses, one could in principle also determine $\Lambda _M$, $\Lambda _F$ and $k_M$, $k_F$. To date this is not yet possible. The results for the LO and NLO constants will be presented in Sect. 5.2.

Alternatively, one can invert Eq. (73) and express $M^2$ and *F* as an expansion in

77 $$\begin{aligned} \xi \equiv \frac{M_\pi ^2}{16 \pi ^2 F_\pi ^2}, \end{aligned}$$

and the corresponding expressions then take the form

78 $$\begin{aligned} M^2= & {} M_\pi ^2\,\left\{ 1+\frac{1}{2}\,\xi \,\ln \frac{\Lambda _3^2}{M_\pi ^2}- \frac{5}{8}\,\xi ^2 \left( \!\ln \frac{\Omega _M^2}{M_\pi ^2}\!\right) ^2\right. \nonumber \\&\left. + \xi ^2 c_{\scriptscriptstyle M}+\mathcal {O}(\xi ^3)\phantom {\left( \!\ln \frac{\Omega _M^2}{M_\pi ^2}\!\right) ^2}\right\} ,\\&F= F_\pi \,\left\{ 1-\xi \,\ln \frac{\Lambda _4^2}{M_\pi ^2}-\frac{1}{4}\,\xi ^2 \left( \!\ln \frac{\Omega _F^2}{M_\pi ^2}\!\right) ^2\right. \nonumber \\&\quad \left. +\xi ^2 c_{\scriptscriptstyle F}+\mathcal {O}(\xi ^3)\right\} .\nonumber \end{aligned}$$

The scales of the quadratic logarithms are determined by $\Lambda _1,\ldots ,\Lambda _4$ through79B. *SU*(3) formulae

While the formulae for the pseudoscalar masses and decay constants are known to NNLO for *SU*(3) as well [262], they are rather complicated and we restrict ourselves here to next-to-leading order (NLO). In the isospin limit, the relevant *SU*(3) formulae take the form [129]80where $m_{ud}$ is the common up and down quark mass (which may be different from the one in the real world), and $B_0=\Sigma _0/F_0^2$, $F_0$ denote the condensate parameter and the pseudoscalar decay constant in the *SU*(3) chiral limit, respectively. In addition, we use the notation

81 $$\begin{aligned} \mu _P=\frac{M_P^2}{32\pi ^2F_0^2} \ln \!\left( \frac{M_P^2}{\mu ^2}\right) . \end{aligned}$$

At the order of the chiral expansion used in these formulae, the quantities $\mu _\pi$, $\mu _K$, $\mu _\eta$ can equally well be evaluated with the leading-order expressions for the masses,

82 $$\begin{aligned}&M_\pi ^2\mathop {=}\limits ^{{\mathrm{LO}}}2B_0\,m_{ud},\quad M_K^2\mathop {=}\limits ^{{\mathrm{LO}}}B_0(m_s+m_{ud}),\nonumber \\&\quad M_\eta ^2\mathop {=}\limits ^{{\mathrm{LO}}}\tfrac{2}{3}B_0(2m_s+m_{ud}). \end{aligned}$$

Throughout, $L_i$ denotes the renormalized low-energy constant/coupling (LEC) at scale $\mu$, and we adopt the convention which is standard in phenomenology, $\mu =M_\rho =770\,\mathrm {MeV}$. The normalization used for the decay constants is specified in Footnote 21.

#### Pion form factors and charge radii

The scalar and vector form factors of the pion are defined by the matrix elements

83 $$\begin{aligned} \begin{aligned}&\langle \pi ^i(p_2) |\, \overline{q}\, q \, | \pi ^k(p_1) \rangle = \delta ^{ik} F_S^\pi (t) ,\\&\langle \pi ^i(p_2) | \,\overline{q}\, \tfrac{1}{2}\tau ^j \gamma ^\mu q\,| \pi ^k(p_1) \rangle = \mathrm {i} \,\epsilon ^{ijk} (p_1^\mu + p_2^\mu ) F_V^\pi (t) ,\end{aligned} \end{aligned}$$

where the operators contain only the lightest two quark flavours, i.e. $\tau ^1$, $\tau ^2$, $\tau ^3$ are the Pauli matrices, and $t\equiv (p_1-p_2)^2$ denotes the momentum transfer.

The vector form factor has been measured by several experiments for time-like as well as for space-like values of *t*. The scalar form factor is not directly measurable, but it can be evaluated theoretically from data on the $\pi \pi$ and $\pi K$ phase shifts [263] by means of analyticity and unitarity, i.e. in a model-independent way. Lattice calculations can be compared with data or model-independent theoretical evaluations at any given value of *t*. At present, however, most lattice studies concentrate on the region close to $t=0$ and on the evaluation of the slope and curvature which are defined as

84 $$\begin{aligned} F^\pi _V(t)= & {} 1+\tfrac{1}{6}\langle r^2 \rangle ^\pi _V t + c_V t^2+\cdots ,\\ F^\pi _S(t)= & {} F^\pi _S(0) \left[ 1+\tfrac{1}{6}\langle r^2 \rangle ^\pi _S t + c_S\, t^2+ \cdots \right] . \nonumber \end{aligned}$$

The slopes are related to the mean-square vector and scalar radii which are the quantities on which most experiments and lattice calculations concentrate.

In $\chi$PT, the form factors are known at NNLO for *SU*(2) [264]. The corresponding formulae are available in fully analytical form and are compact enough to be used for the chiral extrapolation of the data (as done, for example in Refs. [41, 265]). The expressions for the scalar and vector radii and for the $c_{S,V}$ coefficients at two-loop level read

85 $$\begin{aligned}&\langle r^2 \rangle ^\pi _S = \frac{1}{(4\pi F_\pi )^2} \left\{ 6 \ln \frac{\Lambda _4^2}{M_\pi ^2}-\frac{13}{2} -\frac{29}{3}\,\xi \left( \!\ln \frac{\Omega _{r_S}^2}{M_\pi ^2} \!\right) ^2 \right. \nonumber \\&\quad \quad \quad \quad \quad \left. +\, 6 \xi \, k_{r_S}+\mathcal {O}(\xi ^2)\phantom {\left( \!\ln \frac{\Omega _{r_S}^2}{M_\pi ^2} \!\right) ^2}\right\} ,\nonumber \\&\langle r^2 \rangle ^\pi _V = \frac{1}{(4\pi F_\pi )^2} \left\{ \ln \frac{\Lambda _6^2}{M_\pi ^2}-1 +2\,\xi \left( \!\ln \frac{\Omega _{r_V}^2}{M_\pi ^2} \!\right) ^2+6 \xi \,k_{r_V}\right. \nonumber \\&\left. \quad \quad \quad \quad \quad +\,\mathcal {O}(\xi ^2)\phantom {\left( \!\ln \frac{\Omega _{r_S}^2}{M_\pi ^2} \!\right) ^2}\right\} , \\&c_S =\frac{1}{(4\pi F_\pi M_\pi )^2} \left\{ \frac{19}{120} + \xi \left[ \frac{43}{36} \left( \! \ln \frac{\Omega _{c_S}^2}{M_\pi ^2} \!\right) ^2 + k_{c_S} \right] \right\} ,\nonumber \\&c_V =\frac{1}{(4\pi F_\pi M_\pi )^2} \left\{ \frac{1}{60}+\xi \left[ \frac{1}{72} \left( \! \ln \frac{\Omega _{c_V}^2}{M_\pi ^2} \!\right) ^2 + k_{c_V} \right] \right\} ,\nonumber \end{aligned}$$

where

86 $$\begin{aligned} \ln \frac{\Omega _{r_S}^2}{M_\pi ^2}= & {} \frac{1}{29}\,\left( 31\,\ln \frac{\Lambda _1^2}{M_\pi ^2}+34\,\ln \frac{\Lambda _2^2}{M_\pi ^2}-36\,\ln \frac{\Lambda _4^2}{M_\pi ^2}+\frac{145}{24}\right) ,\nonumber \\ \ln \frac{\Omega _{r_V}^2}{M_\pi ^2}= & {} \frac{1}{2}\,\left( \ln \frac{\Lambda _1^2}{M_\pi ^2}-\ln \frac{\Lambda _2^2}{M_\pi ^2}+\ln \frac{\Lambda _4^2}{M_\pi ^2}+\ln \frac{\Lambda _6^2}{M_\pi ^2}-\frac{31}{12}\right) ,\nonumber \\ \ln \frac{\Omega _{c_S}^2}{M_\pi ^2}= & {} \frac{43}{63}\,\left( 11\,\ln \frac{\Lambda _1^2}{M_\pi ^2}+14\,\ln \frac{\Lambda _2^2}{M_\pi ^2}+18\,\ln \frac{\Lambda _4^2}{M_\pi ^2}-\frac{6041}{120}\right) ,\nonumber \\ \ln \frac{\Omega _{c_V}^2}{M_\pi ^2}= & {} \frac{1}{72}\,\left( 2\ln \frac{\Lambda _1^2}{M_\pi ^2}-2\ln \frac{\Lambda _2^2}{M_\pi ^2}-\ln \frac{\Lambda _6^2}{M_\pi ^2}-\frac{26}{30}\right) ,\end{aligned}$$

and $k_{r_S},k_{r_V}$ and $k_{c_S},k_{c_V}$ are independent of the quark masses. Their expression in terms of the $\ell _i$ and of the $\mathcal {O}(p^6)$ constants $c_M,c_F$ is known but will not be reproduced here.

The *SU*(3) formula for the slope of the pion vector form factor reads, to NLO [221],

87 $$\begin{aligned} \langle r^2\rangle _V^\pi \;\mathop {=}\limits ^{{\mathrm{NLO}}}\;-\frac{1}{32\pi ^2F_0^2} \left\{ 3+2\ln \frac{M_\pi ^2}{\mu ^2}+\ln \frac{M_K^2}{\mu ^2}\right\} +\frac{12L_9}{F_0^2},\nonumber \\ \end{aligned}$$

while the expression $\langle r^2\rangle _S^\mathrm {oct}$ for the octet part of the scalar radius does not contain any NLO low-energy constant at one-loop order [221] – contrary to the situation in *SU*(2); see Eq. (85).

The difference between the quark-line connected and the full (i.e. containing the connected and the disconnected pieces) scalar pion form factor has been investigated by means of $\chi$PT in Ref. [266]. It is expected that the technique used can be applied to a large class of observables relevant in QCD phenomenology.

As a point of practical interest let us remark that there are no finite-volume correction formulae for the mean-square radii $\langle r^2\rangle _{V,S}$ and the curvatures $c_{V,S}$. The lattice data for $F_{V,S}(t)$ need to be corrected, point by point in *t*, for finite-volume effects. In fact, if a given *t* is realized through several inequivalent $p_1\!-\!p_2$ combinations, the level of agreement after the correction has been applied is indicative of how well higher-order effects are under control.

#### Partially quenched and mixed action formulations

The term “partially quenched QCD” is used in two ways. For heavy quarks (*c*, *b* and sometimes *s*) it usually means that these flavours are included in the valence sector, but not into the functional determinant, i.e. the sea sector. For the light quarks (*u*, *d* and sometimes *s*) it means that they are present in both the valence and the sea sector of the theory, but with different masses (e.g. a series of valence quark masses is evaluated on an ensemble with fixed sea-quark masses).

The program of extending the standard (unitary) *SU*(3) theory to the (second version of) “partially quenched QCD” has been completed at the two-loop (NNLO) level for masses and decay constants [267]. These formulae tend to be complicated, with the consequence that a state-of-the-art analysis with $\mathcal {O}(2000)$ bootstrap samples on $\mathcal {O}(20)$ ensembles with $\mathcal {O}(5)$ masses each [and hence $\mathcal {O}(200\,000)$ different fits] will require significant computational resources. For a summary of recent developments in $\chi$PT relevant to lattice QCD we refer to Ref. [268]. The *SU*(2) partially quenched formulae can be obtained from the *SU*(3) ones by “integrating out the strange quark.” At NLO, they can be found in Ref. [269] by setting the lattice-artefact terms from the staggered $\chi$PT form to zero.

The theoretical underpinning of how “partial quenching” is to be understood in the (properly extended) chiral framework is given in Ref. [270]. Specifically, for partially quenched QCD with staggered quarks it is shown that a transfer matrix can be constructed which is not Hermitian but bounded, and can thus be used to construct correlation functions in the usual way. The program of calculating all observables in the *p*-regime in finite volume to two loops, first completed in the unitary theory [271, 272], has been carried out for the partially quenched case, too [273].

A further extension of the $\chi$PT framework concerns the lattice effects that arise in partially quenched simulations where sea and valence quarks are implemented with different lattice fermion actions [222, 274–280].

#### Correlation functions in the $\epsilon$-regime

The finite-size effects encountered in lattice calculations can be used to determine some of the LECs of QCD. In order to illustrate this point, we focus on the two lightest quarks, take the isospin limit $m_u=m_d=m$ and consider a box of size $L_s$ in the three space directions and size $L_t$ in the time direction. If *m* is sent to zero at fixed box size, chiral symmetry is restored, and the zero-momentum mode of the pion field becomes nonperturbative. An intuitive way to understand the regime with $ML<1$ ($L=L_s\,\lesssim \,L_t$) starts from considering the pion propagator $G(p)=1/(p^2+M^2)$ in finite volume. For $ML\,\gtrsim \,1$ and $p\sim 1/L$, $G(p)\sim L^2$ for small momenta, including $p=0$. But when *M* becomes of order $1/L^2$, $G(0)\propto L^4\gg G(p\ne 0)\sim L^2$. The $p=0$ mode of the pion field becomes nonperturbative, and the integration over this mode restores chiral symmetry in the limit $m\rightarrow 0$.

The pion effective action for the zero-momentum field depends only on the combination $\mu =m\Sigma V$, the symmetry-restoration parameter, where $V=L_s^3 L_t$. In the $\epsilon$-regime, in which $m\sim 1/V$, all other terms in the effective action are sub-dominant in powers of $\epsilon \sim 1/L$, leading to a reordering of the usual chiral expansion, which assumes that $m\sim \epsilon ^2$ instead of $m\sim \epsilon ^4$. In the *p*-regime, with $m\sim \epsilon ^2$ or equivalently $ML\,\gtrsim \, 1$, finite-volume corrections are of order $\int d^4p\,e^{ipx}\,G(p)|_{x\sim L}\sim e^{-ML}$, while in the $\epsilon$-regime, the chiral expansion is an expansion in powers of $1/(\Lambda _\mathrm {QCD}L)\sim 1/(FL)$.

As an example, we consider the correlator of the axial charge carried by the two lightest quarks, $q(x)=\{u(x),d(x)\}$. The axial current and the pseudoscalar density are given by

88 $$\begin{aligned} A_\mu ^i(x)= \overline{q}(x)\tfrac{1}{2} \tau ^i\,\gamma _\mu \gamma _5\,q(x),\quad P^i(x) = \overline{q}(x)\frac{1}{2} \tau ^i\,\mathrm {i} \gamma _5\,q(x),\nonumber \\ \end{aligned}$$

where $\tau ^1, \tau ^2,\tau ^3$ are the Pauli matrices in flavour space. In Euclidean space, the correlators of the axial charge and of the space integral over the pseudoscalar density are given by

89 $$\begin{aligned} \delta ^{ik}C_{AA}(t)= & {} L_s^3\int \mathrm{d}^3\vec {x}\;\langle A_4^i(\vec {x},t) A_4^k(0)\rangle , \\ \delta ^{ik}C_{PP}(t)= & {} L_s^3\int \mathrm{d}^3\vec {x}\;\langle P^i(\vec {x},t) P^k(0)\rangle .\nonumber \end{aligned}$$

$\chi$PT yields explicit finite-size scaling formulae for these quantities [260, 281, 282]. In the $\epsilon$-regime, the expansion starts with

90 $$\begin{aligned} C_{AA}(t)= & {} \frac{F^2L_s^3}{L_t}\left[ a_A+ \frac{L_t}{F^2L_s^3}\,b_A\,h_1\left( \frac{t}{L_t} \right) +\mathcal {O}(\epsilon ^4)\right] , \nonumber \\ C_{PP}(t)= & {} \Sigma ^2L_s^6\left[ a_P+\frac{L_t}{F^2L_s^3}\,b_P\,h_1\left( \frac{t}{L_t} \right) +\mathcal {O}(\epsilon ^4)\right] ,\nonumber \\ \end{aligned}$$

where the coefficients $a_A$, $b_A$, $a_P$, $b_P$ stand for quantities of $\mathcal {O}(\epsilon ^0)$. They can be expressed in terms of the variables $L_s$, $L_t$ and *m* and involve only the two leading low-energy constants *F* and $\Sigma$. In fact, at leading order only the combination $\mu =m\,\Sigma \,L_s^3 L_t$ matters, the correlators are *t*-independent and the dependence on $\mu$ is fully determined by the structure of the groups involved in the pattern of spontaneous symmetry breaking. In the case of $SU(2)\times SU(2)$ $\rightarrow$ *SU*(2), relevant for QCD in the symmetry-restoration region with two light quarks, the coefficients can be expressed in terms of Bessel functions. The *t*-dependence of the correlators starts showing up at $\mathcal {O}(\epsilon ^2)$, in the form of a parabola, viz. $h_1(\tau )=\frac{1}{2}[(\tau -\frac{1}{2} )^2-\frac{1}{12} ]$. Explicit expressions for $a_A$, $b_A$, $a_P$, $b_P$ can be found in Refs. [260, 281, 282], where some of the correlation functions are worked out to NNLO. By matching the finite-size scaling of correlators computed on the lattice with these predictions one can extract *F* and $\Sigma$. A way to deal with the numerical challenges germane to the $\epsilon$-regime has been described [283].

The fact that the representation of the correlators to NLO is not “contaminated” by higher-order unknown LECs, makes the $\epsilon$-regime potentially convenient for a clean extraction of the LO couplings. The determination of these LECs is then affected by different systematic uncertainties with respect to the standard case; simulations in this regime yield complementary information which can serve as a valuable cross-check to get a comprehensive picture of the low-energy properties of QCD.

The effective theory can also be used to study the distribution of the topological charge in QCD [284] and the various quantities of interest may be defined for a fixed value of this charge. The expectation values and correlation functions then not only depend on the symmetry-restoration parameter $\mu$, but also on the topological charge $\nu$. The dependence on these two variables can explicitly be calculated. It turns out that the 2-point correlation functions considered above retain the form (90), but the coefficients $a_A$, $b_A$, $a_P$, $b_P$ now depend on the topological charge as well as on the symmetry-restoration parameter (see Refs. [285–287] for explicit expressions).

A specific issue with $\epsilon$-regime calculations is the scale setting. Ideally one would perform a *p*-regime study with the same bare parameters to measure a hadronic scale (e.g. the proton mass). In the literature, sometimes a gluonic scale (e.g. $r_0$) is used to avoid such expenses. Obviously the issues inherent in scale setting are aggravated if the $\epsilon$-regime simulation is restricted to a fixed sector of topological charge.

It is important to stress that in the $\epsilon$-expansion higher-order finite-volume corrections might be significant, and the physical box size (in fm) should still be large in order to keep these distortions under control. The criteria for the chiral extrapolation and finite-volume effects are obviously different from the *p*-regime. For these reasons we have to adjust the colour coding defined in Sect. 2.1 (see Sect. 5.2 for more details).

Recently, the effective theory has been extended to the “mixed regime” where some quarks are in the *p*-regime and some in the $\epsilon$-regime [288, 289]. In Ref. [290] a technique is proposed to smoothly connect the *p*- and $\epsilon$-regimes. In Ref. [291] the issue is reconsidered with a counting rule which is essentially the same as in the *p*-regime. In this new scheme, one can treat the IR fluctuations of the zero-mode nonperturbatively, while keeping the logarithmic quark mass dependence of the *p*-regime.

Also first steps towards calculating higher *n*-point functions in the $\epsilon$-regime have been taken. For instance the electromagnetic pion form factor in QCD has been calculated to NLO in the $\epsilon$-expansion, and a way to get rid of the pion zero-momentum part has been proposed [292].

#### Energy levels of the QCD Hamiltonian in a box and $\delta$-regime

At low temperature, the properties of the partition function are governed by the lowest eigenvalues of the Hamiltonian. In the case of QCD, the lowest levels are due to the Nambu–Goldstone bosons and can be worked out with $\chi$PT [293]. In the chiral limit the level pattern follows the one of a quantum-mechanical rotator, i.e. $E_\ell =\ell (\ell +1)/(2\,\Theta )$ with $\ell = 0, 1,2,\ldots$ For a cubic spatial box and to leading order in the expansion in inverse powers of the box size $L_s$, the moment of inertia is fixed by the value of the pion decay constant in the chiral limit, i.e. $\Theta =F^2L_s^3$.

In order to analyse the dependence of the levels on the quark masses and on the parameters that specify the size of the box, a reordering of the chiral series is required, the so-called $\delta$-expansion; the region where the properties of the system are controlled by this expansion is referred to as the $\delta$-regime. Evaluating the chiral series in this regime, one finds that the expansion of the partition function goes in even inverse powers of $FL_s$, that the rotator formula for the energy levels holds up to NNLO and the expression for the moment of inertia is now also known up to and including terms of order $(FL_s)^{-4}$ [294–296]. Since the level spectrum is governed by the value of the pion decay constant in the chiral limit, an evaluation of this spectrum on the lattice can be used to measure *F*. More generally, the evaluation of various observables in the $\delta$-regime offers an alternative method for a determination of some of the low-energy constants occurring in the effective Lagrangian. At present, however, the numerical results obtained in this way [178, 297] are not yet competitive with those found in the *p*- or $\epsilon$-regime.

#### Other methods for the extraction of the low-energy constants

An observable that can be used to extract LECs is the topological susceptibility

91 $$\begin{aligned} \chi _t=\int \mathrm{d}^4\!x\; \langle \omega (x) \omega (0)\rangle , \end{aligned}$$

where $\omega (x)$ is the topological charge density,

92 $$\begin{aligned} \omega (x)=\frac{1}{32\pi ^2} \epsilon ^{\mu \nu \rho \sigma }\mathrm{Tr}\left[ F_{\mu \nu }(x)F_{\rho \sigma }(x)\right] . \end{aligned}$$

At infinite volume, the expansion of $\chi _t$ in powers of the quark masses starts with [298]

93 $$\begin{aligned} \chi _t=\overline{m}\,\Sigma \,\{1+\mathcal {O}(m)\},\quad \overline{m}\equiv \left( \frac{1}{m_u}+\frac{1}{m_d}+\frac{1}{m_s}+\cdots \right) ^{-1}.\nonumber \\ \end{aligned}$$

The condensate $\Sigma$ can thus be extracted from the properties of the topological susceptibility close to the chiral limit. The behaviour at finite volume, in particular in the region where the symmetry is restored, is discussed in Ref. [282]. The dependence on the vacuum angle $\theta$ and the projection on sectors of fixed $\nu$ have been studied in Ref. [284]. For a discussion of the finite-size effects at NLO, including the dependence on $\theta$, we refer to Refs. [287, 299].

The role that the topological susceptibility plays in attempts to determine whether there is a large paramagnetic suppression when going from the $N_{ f}=2$ to the $N_{ f}=2+1$ theory has been highlighted in Ref. [300]. And the potential usefulness of higher moments of the topological charge distribution to determine LECs has been investigated in Ref. [301].

Another method for computing the quark condensate has been proposed in Ref. [302], where it is shown that starting from the Banks–Casher relation [303] one may extract the condensate from suitable (renormalizable) spectral observables, for instance the number of Dirac operator modes in a given interval. For those spectral observables higher-order corrections can be systematically computed in terms of the chiral effective theory. For recent implementations of this strategy, see Refs. [33, 38, 304]. As an aside let us remark that corrections to the Banks–Casher relation that come from a finite quark mass, a finite four-dimensional volume and (with Wilson-type fermions) a finite-lattice spacing can be parameterized in a properly extended version of the chiral framework [305, 306].

An alternative strategy is based on the fact that at LO in the $\epsilon$-expansion the partition function in a given topological sector $\nu$ is equivalent to the one of a chiral Random Matrix Theory (RMT) [307–310]. In RMT it is possible to extract the probability distributions of individual eigenvalues [311–313] in terms of two dimensionless variables $\zeta =\lambda \Sigma V$ and $\mu =m\Sigma V$, where $\lambda$ represents the eigenvalue of the massless Dirac operator and *m* is the sea-quark mass. More recently this approach has been extended to the Hermitian (Wilson) Dirac operator [314] which is easier to study in numerical simulations. Hence, if it is possible to match the QCD low-lying spectrum of the Dirac operator to the RMT predictions, then one may extract^23^ the chiral condensate $\Sigma$. One issue with this method is that for the distributions of individual eigenvalues higher-order corrections are still not known in the effective theory, and this may introduce systematic effects which are hard^24^ to control. Another open question is that, while it is clear how the spectral density is renormalized [318], this is not the case for the individual eigenvalues, and one relies on assumptions. There have been many lattice studies [319–323] which investigate the matching of the low-lying Dirac spectrum with RMT. In this review the results of the LECs obtained in this way^25^ are not included.

### Extraction of *SU*(2) low-energy constants

In this and the following subsections we summarize the lattice results for the *SU*(2) and *SU*(3) LECs, respectively. In either case we first discuss the $\mathcal {O}(p^2)$ constants and then proceed to their $\mathcal {O}(p^4)$ counterparts. The $\mathcal {O}(p^2)$ LECs are determined from the chiral extrapolation of masses and decay constants or, alternatively, from a finite-size study of correlators in the $\epsilon$-regime. At order $p^4$ some LECs affect 2-point functions while others appear only in three- or 4-point functions; the latter need to be determined from form factors or scattering amplitudes. The $\chi$PT analysis of the (nonlattice) phenomenological quantities is nowadays^26^ based on $\mathcal {O}(p^6)$ formulae. At this level the number of LECs explodes and we will not discuss any of these. We will, however, discuss how comparing different orders and different expansions (in particular the *x* versus $\xi$-expansion) can help to assess the theoretical uncertainties of the LECs determined on the lattice.

**Table 19:** Cubic root of the *SU*(2) quark condensate $\Sigma \equiv -\langle \overline{u}u\rangle |_{m_u,m_d\rightarrow 0}$ in $\,\mathrm {MeV}$ units, in the $\overline{\mathrm{MS}}$-scheme, at the renormalization scale $\mu =2$ GeV. All ETM values which were available only in $r_0$ units were converted on the basis of $r_0=0.48(2)~\mathrm{fm}$ [333, 350, 351], with this error being added in quadrature to any existing systematic error

Collaboration	Refs.	$N_{ f}$	Publication status	Chiral extrapolation	Continuum extrapolation	Finite volume	Renormalization	$\Sigma ^{1/3}$
ETM 13	[33]	$2+1+1$	A					280(8)(15)
RBC/UKQCD 15E	[335]	$2+1$	P					274.2(2.8)(4.0)
RBC/UKQCD 14B	[10]	$2+1$	A					275.9(1.9)(1.0)
BMW 13	[35]	$2+1$	A					271(4)(1)
Borsanyi 12	[34]	$2+1$	A					272.3(1.2)(1.4)
MILC 10A	[13]	$2+1$	C					281.5(3.4)$$\left( {\begin{array}{c}+2.0\\ -5.9\end{array}}\right) $$(4.0)
JLQCD/TWQCD 10A	[338]	$2+1$	A					234(4)(17)
RBC/UKQCD 10A	[144]	$2+1$	A					256(5)(2)(2)
JLQCD 09	[337]	$2+1$	A					242(4)$$\left( {\begin{array}{c}+19\\ -18\end{array}}\right) $$
MILC 09A, *SU*(3)-fit	[6]	$2+1$	C					279(1)(2)(4)
MILC 09A, *SU*(2)-fit	[6]	$2+1$	C					280(2)$$\left( {\begin{array}{c}+4\\ -8\end{array}}\right) $$(4)
MILC 09	[89]	$2+1$	A					278(1)$$\left( {\begin{array}{c}+2\\ -3\end{array}}\right) $$(5)
TWQCD 08	[340]	$2+1$	A					259(6)(9)
JLQCD/TWQCD 08B	[341]	$2+1$	C					249(4)(2)
PACS-CS 08, *SU*(3)-fit	[93]	$2+1$	A					312(10)
PACS-CS 08, *SU*(2)-fit	[93]	$2+1$	A					309(7)
RBC/UKQCD 08	[145]	$2+1$	A					255(8)(8)(13)
Engel 14	[38]	2	A					263(3)(4)
Brandt 13	[37]	2	A					261(13)(1)
ETM 13	[33]	2	A					283(7)(17)
ETM 12	[342]	2	A					299(26)(29)
Bernardoni 11	[343]	2	C					306(11)
TWQCD 11	[249]	2	A					230(4)(6)
TWQCD 11A	[344]	2	A					259(6)(7)
JLQCD/TWQCD 10A	[338]	2	A					242(5)(20)
Bernardoni 10	[345]	2	A					262$$\left( {\begin{array}{c}+33\\ -34\end{array}}\right) \left( {\begin{array}{c}+4\\ -5\end{array}}\right) $$
ETM 09C	[36]	2	A					270(5)$$\left( {\begin{array}{c}+3\\ -4\end{array}}\right) $$
ETM 09B	[346]	2	C					245(5)
ETM 08	[41]	2	A					264(3)(5)
CERN 08	[302]	2	A					276(3)(4)(5)
Hasenfratz 08	[347]	2	A					248(6)
JLQCD/TWQCD 08A	[138]	2	A					235.7(5.0)(2.0)$$\left( {\begin{array}{c}+12.7\\ -0.0\end{array}}\right) $$
JLQCD/TWQCD 07	[348]	2	A					239.8(4.0)
JLQCD/TWQCD 07A	[349]	2	A					252(5)(10)

**Table 20:** Results for the *SU*(2) low-energy constant *F* (in MeV) and for the ratio $F_\pi /F$. All ETM values which were available only in $r_0$ units were converted on the basis of $r_0=0.48(2)~\mathrm{fm}$ [333, 350, 351], with this error being added in quadrature to any existing systematic error. Numbers in slanted fonts have been calculated by us, based on $\sqrt{2}F_\pi ^\mathrm {phys}=130.41(20)~\,\mathrm {MeV}$ [151], with this error being added in quadrature to any existing systematic error

Collaboration	Refs.	$N_{ f}$	Publication status	Chiral extrapolation	Continuum extrapolation	Finite volume	*F*	$F_\pi /F$
ETM 11	[352]	$2+1+1$	C				85.60(4)	*1.077(1)*
ETM 10	[39]	$2+1+1$	A				85.66(6)(13)	1.076(2)(2)
RBC/UKQCD 15E	[335]	$2+1$	P				85.8(1.1)(1.5)	1.0641(21)(49)
RBC/UKQCD 14B	[10]	$2+1$	A				86.63(12)(13)	1.0645(15)(0)
BMW 13	[35]	$2+1$	A				88.0(1.3)(0.3)	1.055(7)(2)
Borsanyi 12	[34]	$2+1$	A				86.78(05)(25)	1.0627(06)(27)
NPLQCD 11	[40]	$2+1$	A				86.8(2.1)$$\left( {\begin{array}{c}+3.3\\ -3.4\end{array}}\right) $$	1.062(26)$$\left( {\begin{array}{c}+42\\ -40\end{array}}\right) $$
MILC 10	[29]	$2+1$	C				87.0(4)(5)	*1.060(5)(6)*
MILC 10A	[13]	$2+1$	C				87.5(1.0)$$\left( {\begin{array}{c}+0.7\\ -2.6\end{array}}\right) $$	1.054(12)$$\left( {\begin{array}{c}+31\\ -09\end{array}}\right) $$
MILC 09A, *SU*(3)-fit	[6]	$2+1$	C				86.8(2)(4)	1.062(1)(3)
MILC 09A, *SU*(2)-fit	[6]	$2+1$	C				87.4(0.6)$$\left( {\begin{array}{c}+0.9\\ -1.0\end{array}}\right) $$	1.054(7)$$\left( {\begin{array}{c}+12\\ -11\end{array}}\right) $$
MILC 09	[89]	$2+1$	A				87.66(17)$$\left( {\begin{array}{c}+28\\ -52\end{array}}\right) $$	1.052(2)$$\left( {\begin{array}{c}+6\\ -3\end{array}}\right) $$
PACS-CS 08, *SU*(3)-fit	[93]	$2+1$	A				90.3(3.6)	1.062(8)
PACS-CS 08, *SU*(2)-fit	[93]	$2+1$	A				89.4(3.3)	1.060(7)
RBC/UKQCD 08	[145]	$2+1$	A				81.2(2.9)(5.7)	1.080(8)
ETM 15A	[333]	2	P				86.3(2.8)	*1.069(35)*
Engel 14	[38]	2	A				85.8(0.7)(2.0)	*1.075(09)(25)*
Brandt 13	[37]	2	A				84(8)(2)	1.080(16)(6)
QCDSF 13	[353]	2	A				86(1)	*1.07(1)*
TWQCD 11	[249]	2	A				83.39(35)(38)	*1.106(5)(5)*
ETM 09C	[36]	2	A				85.91(07)$$\left( {\begin{array}{c}+78\\ -07\end{array}}\right) $$	1.0755(6)$$\left( {\begin{array}{c}+08\\ -94\end{array}}\right) $$
ETM 09B	[346]	2	C				92.1(4.9)	*1.00(5)*
ETM 08	[41]	2	A				86.6(7)(7)	1.067(9)(9)
Hasenfratz 08	[347]	2	A				90(4)	*1.02(5)*
JLQCD/TWQCD 08A	[138]	2	A				79.0(2.5)(0.7)$$\left( {\begin{array}{c}+4.2\\ -0.0\end{array}}\right) $$	1.167(37)(10)$$\left( {\begin{array}{c}+02\\ -62\end{array}}\right) $$
JLQCD/TWQCD 07	[348]	2	A				87.3(5.6)	*1.06(7)*
Colangelo 03	[354]						86.2(5)	1.0719(52)

**Table 21:** Results for the *SU*(2) NLO low-energy constants $\bar{\ell }_3$ and $\bar{\ell }_4$. For comparison, the last two lines show results from phenomenological analyses

Collaboration	Refs.	$N_{ f}$	Publication status	Chiral extrapolation	Continuum extrapolation	Finite volume	$\bar{\ell }_3$	$\bar{\ell }_4$
ETM 11	[352]	$2+1+1$	C				3.53(5)	4.73(2)
ETM 10	[39]	$2+1+1$	A				3.70(7)(26)	4.67(3)(10)
RBC/UKQCD 15E	[335]	$2+1$	P				2.81(19)(45)	4.02(8)(24)
RBC/UKQCD 14B	[10]	$2+1$	A				2.73(13)(0)	4.113(59)(0)
BMW 13	[35]	$2+1$	A				2.5(5)(4)	3.8(4)(2)
RBC/UKQCD 12	[31]	$2+1$	A				2.91(23)(07)	3.99(16)(09)
Borsanyi 12	[34]	$2+1$	A				3.16(10)(29)	4.03(03)(16)
NPLQCD 11	[40]	$2+1$	A				4.04(40)$$\left( {\begin{array}{c}+73\\ -55\end{array}}\right) $$	4.30(51)$$\left( {\begin{array}{c}+84\\ -60\end{array}}\right) $$
MILC 10	[29]	$2+1$	C				3.18(50)(89)	4.29(21)(82)
MILC 10A	[13]	$2+1$	C				2.85(81)$$\left( {\begin{array}{c}+37\\ -92\end{array}}\right) $$	3.98(32)$$\left( {\begin{array}{c}+51\\ -28\end{array}}\right) $$
RBC/UKQCD 10A	[144]	$2+1$	A				2.57(18)	3.83(9)
MILC 09A, *SU*(3)-fit	[6]	$2+1$	C				3.32(64)(45)	4.03(16)(17)
MILC 09A, *SU*(2)-fit	[6]	$2+1$	C				3.0(6)$$\left( {\begin{array}{c}+9\\ -6\end{array}}\right) $$	3.9(2)(3)
PACS-CS 08, *SU*(3)-fit	[93]	$2+1$	A				3.47(11)	4.21(11)
PACS-CS 08, *SU*(2)-fit	[93]	$2+1$	A				3.14(23)	4.04(19)
RBC/UKQCD 08	[145]	$2+1$	A				3.13(33)(24)	4.43(14)(77)
ETM 15A	[333]	2	P					3.3(4)
Gülpers 15	[355]	2	P					4.54(30)(0)
Gülpers 13	[356]	2	A					4.76(13)
Brandt 13	[37]	2	A				3.0(7)(5)	4.7(4)(1)
QCDSF 13	[353]	2	A					4.2(1)
Bernardoni 11	[343]	2	C				4.46(30)(14)	4.56(10)(4)
TWQCD 11	[249]	2	A				4.149(35)(14)	4.582(17)(20)
ETM 09C	[36]	2	A				3.50(9)$$\left( {\begin{array}{c}+09\\ -30\end{array}}\right) $$	4.66(4)$$\left( {\begin{array}{c}+04\\ -33\end{array}}\right) $$
JLQCD/TWQCD 09	[357]	2	A					4.09(50)(52)
ETM 08	[41]	2	A				3.2(8)(2)	4.4(2)(1)
JLQCD/TWQCD 08A	[138]	2	A				3.38(40)(24)$$\left( {\begin{array}{c}+31\\ -00\end{array}}\right) $$	4.12(35)(30)$$\left( {\begin{array}{c}+31\\ -00\end{array}}\right) $$
CERN-TOV 06	[358]	2	A				3.0(5)(1)	
Colangelo 01	[261]							4.4(2)
Gasser 84	[131]						2.9(2.4)	4.3(9)

**Table 22:** Top (vector form factor of the pion): Lattice results for the charge radius $\langle r^2\rangle _V^\pi$ (in $\hbox {fm}^2$), the curvature $c_V$ (in $\hbox {GeV}^{-4}$) and the effective coupling constant $\bar{\ell }_6$ are compared with the experimental value, as obtained by NA7, and some phenomenological estimates. Bottom (scalar form factor of the pion): Lattice results for the scalar radius $\langle r^2 \rangle _S^\pi$ (in $\hbox {fm}^2$) and the combination $\bar{\ell }_1-\bar{\ell }_2$ are compared with a dispersive calculation of these quantities

Collaboration	Refs.	$N_{ f}$	Publication status	Chiral extrapolation	Continuum extrapolation	Finite volume	$\langle r^2\rangle _V^\pi$	$c_V$	$\bar{\ell }_6$
HPQCD 15B	[336]	$2+1+1$	P				0.403(18)(6)		
JLQCD 15A , *SU*(2)-fit	[359]	$2+1$	P				0.395(26)(32)		13.49(89)(82)
JLQCD 14	[360]	$2+1$	A				0.49(4)(4)		7.5(1.3)(1.5)
PACS-CS 11A	[361]	$2+1$	A				0.441(46)		
RBC/UKQCD 08A	[339]	$2+1$	A				0.418(31)		12.2(9)
LHP 04	[362]	$2+1$	A				0.310(46)		
Brandt 13	[37]	2	A				0.481(33)(13)		15.5(1.7)(1.3)
JLQCD/TWQCD 09	[357]	2	A				0.409(23)(37)	3.22(17)(36)	11.9(0.7)(1.0)
ETM 08	[41]	2	A				0.456(30)(24)	3.37(31)(27)	14.9(1.2)(0.7)
QCDSF/UKQCD 06A	[363]	2	A				0.441(19)(63)		
Bijnens 98	[264]						0.437(16)	3.85(60)	16.0(0.5)(0.7)
NA7 86	[364]						0.439(8)		
Gasser 84	[131]								16.5(1.1)

Collaboration	Refs.	$N_{ f}$	Publication status	Chiral extrapolation	Continuum extrapolation	Finite volume	$\langle r^2\rangle _S^\pi$	$\bar{\ell }_1-\bar{\ell }_2$	
HPQCD 15B	[336]	$2+1+1$	P				0.481(37)(50)		
RBC/UKQCD 15E	[335]	$2+1$	P					$-$9.2(4.9)(6.5)	
Gülpers 15	[355]	2	P				0.600(52)(0)		
Gülpers 13	[356]	2	A				0.637(23)		
JLQCD/TWQCD 09	[357]	2	A				0.617(79)(66)	$-$2.9(0.9)(1.3)	
Colangelo 01	[261]						0.61(4)	$-$4.7(6)	

**Fig. 11:**
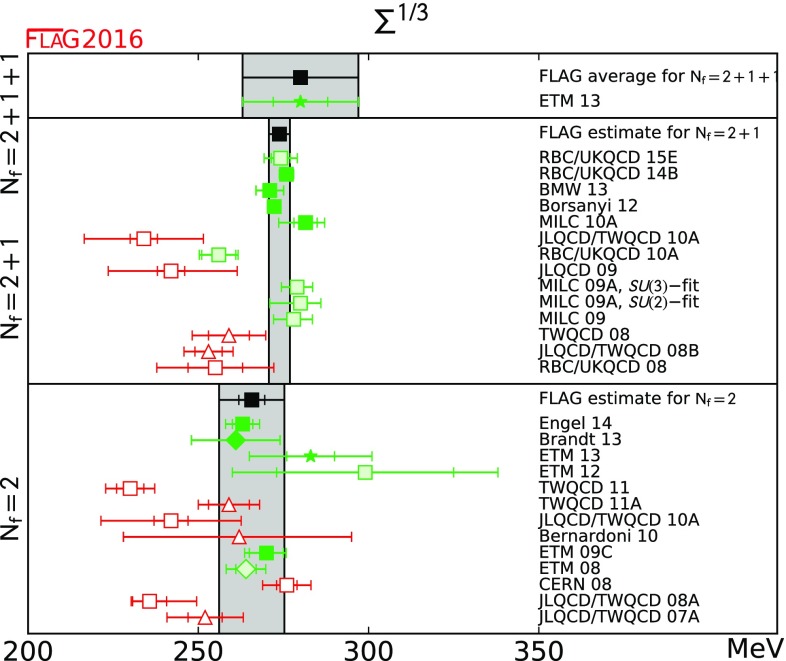
Cubic root of the *SU*(2) quark condensate $\Sigma \equiv -\langle \overline{u}u\rangle |_{m_u,m_d\rightarrow 0}$ in the $\overline{\mathrm{MS}}$-scheme, at the renormalization scale $\mu =2$ GeV. *Squares* indicate determinations from correlators in the *p*-regime. *Up triangles* refer to extractions from the topological susceptibility, *diamonds* to determinations from the pion form factor, and *star symbols* refer to the spectral density method

**Fig. 12:**
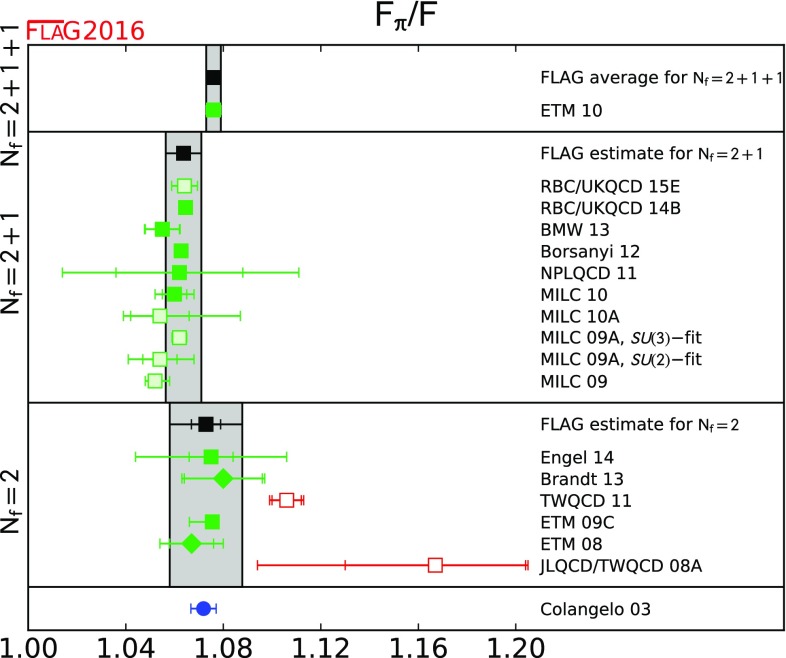
Comparison of the results for the ratio of the physical-pion decay constant $F_\pi$ and the leading-order *SU*(2) low-energy constant *F*. The meaning of the symbols is the same as in Fig. 11

**Fig. 13:**
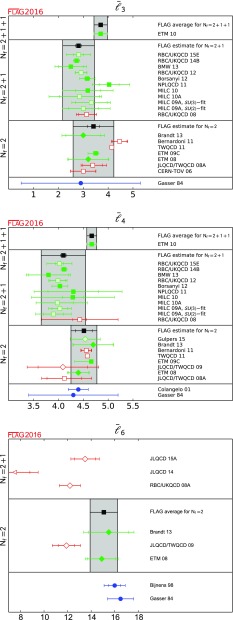
Effective coupling constants $\bar{\ell }_3$, $\bar{\ell }_4$ and $\bar{\ell }_6$. *Squares* indicate determinations from correlators in the *p*-regime, *diamonds* refer to determinations from the pion form factor

The lattice results for the *SU*(2) LECs are summarized in Tables 19, 20, 21 and 22 and Figs. 11, 12 and 13. The tables present our usual colour coding which summarizes the main aspects related to the treatment of the systematic errors of the various calculations.

A delicate issue in the lattice determination of chiral LECs (in particular at NLO) which cannot be reflected by our colour coding is a reliable assessment of the theoretical error that comes from the chiral expansion. We add a few remarks on this point:

1. Using *both* the *x* and the $\xi$ expansion is a good way to test how the ambiguity of the chiral expansion (at a given order) affects the numerical values of the LECs that are determined from a particular set of data [35, 138]. For instance, to determine $\bar{\ell }_4$ (or $\Lambda _4$) from lattice data for $F_\pi$ as a function of the quark mass, one may compare the fits based on the parameterization $F_\pi =F\{1+x\ln (\Lambda _4^2/M^2)\}$ [see Eq. (73)] with those obtained from $F_\pi =F/\{1-\xi \ln (\Lambda _4^2/M_\pi ^2)\}$ [see Eq. (78)]. The difference between the two results provides an estimate of the uncertainty due to the truncation of the chiral series. Which central value one chooses is in principle arbitrary, but we find it advisable to use the one obtained with the $\xi$ expansion,^27^ in particular because it makes the comparison with phenomenological determinations (where it is standard practice to use the $\xi$ expansion) more meaningful.
2. Alternatively one could try to estimate the influence of higher chiral orders by reshuffling irrelevant higher-order terms. For instance, in the example mentioned above one might use $F_\pi =F/\{1-x\ln (\Lambda _4^2/M^2)\}$ as a different functional form at NLO. Another way to establish such an estimate is through introducing by hand “analytical” higher-order terms (e.g. “analytical NNLO” as done, in the past, by MILC [89]). In principle it would be preferable to include all NNLO terms or none, such that the structure of the chiral expansion is preserved at any order (this is what ETM [36] and JLQCD/TWQCD [138] have done for *SU*(2) $\chi$PT and MILC for both *SU*(2) and *SU*(3) $\chi$PT [6, 13, 29]). There are different opinions in the field as to whether it is advisable to include terms to which the data are not sensitive. In the case one is willing to include external (typically: nonlattice) information, the use of priors is a theoretically well-founded option (e.g. priors for NNLO LECs if one is interested exclusively in LECs at LO/NLO).
3. Another issue concerns the *s*-quark mass dependence of the LECs $\bar{\ell }_i$ or $\Lambda _i$ of the *SU*(2) framework. As far as variations of $m_s$ around $m_s^\mathrm {phys}$ are concerned (say for $0<m_s<1.5m_s^\mathrm {phys}$ at best) the issue can be studied in *SU*(3) $\chi$PT, and this has been done in a series of papers [129, 324, 325]. However, the effect of sending $m_s$ to infinity, as is the case in $N_{ f}=2$ lattice studies of *SU*(2) LECs, cannot be addressed in this way. A way to analyse this difference is to compare the numerical values of LECs determined in $N_{ f}=2$ lattice simulations to those determined in $N_{ f}=2+1$ lattice simulations (see e.g. Ref. [326] for a discussion).
4. Last but not least let us recall that the determination of the LECs is affected by discretization effects, and it is important that these are removed by means of a continuum extrapolation. In this step invoking an extended version of the chiral Lagrangian [275, 327–331] may be useful^28^ in the case one aims for a global fit of lattice data involving several $M_\pi$ and *a* values and several chiral observables.

In the tables and figures we summarize the results of various lattice collaborations for the *SU*(2) LECs at LO (*F* or $F/F_\pi$, *B* or $\Sigma$) and at NLO ($\bar{\ell }_1-\bar{\ell }_2$, $\bar{\ell }_3$, $\bar{\ell }_4$, $\bar{\ell }_6$). Throughout we group the results into those which stem from $N_{ f}=2+1+1$ calculations, those which come from $N_{ f}=2+1$ calculations and those which stem from $N_{ f}=2$ calculations (since, as mentioned above, the LECs are logically distinct even if the current precision of the data is not sufficient to resolve the differences). Furthermore, we make a distinction whether the results are obtained from simulations in the *p*-regime or whether alternative methods ($\epsilon$-regime, spectral densities, topological susceptibility, etc.) have been used (this should not affect the result). For comparison we add, in each case, a few representative phenomenological determinations.

A generic comment applies to the issue of the scale setting. In the past none of the lattice studies with $N_{ f}\ge 2$ involved simulations in the *p*-regime at the physical value of $m_{ud}$. Accordingly, the setting of the scale $a^{-1}$ via an experimentally measurable quantity did necessarily involve a chiral extrapolation, and as a result of this dimensionful quantities used to be particularly sensitive to this extrapolation uncertainty, while in dimensionless ratios such as $F_\pi /F$, $F/F_0$, $B/B_0$, $\Sigma /\Sigma _0$ this particular problem is much reduced (and often finite lattice-to-continuum renormalization factors drop out). Now, there is a new generation of lattice studies with $N_{ f}=2$ [333], $N_{ f}=2+1$ [7, 8, 10, 23, 31, 34, 35, 94, 334, 335], and $N_{ f}=2+1+1$ [26, 336], which does involve simulations at physical-pion masses. In such studies the uncertainty that the scale setting has on dimensionful quantities is much mitigated.

It is worth repeating here that the standard colour-coding scheme of our tables is necessarily schematic and cannot do justice to every calculation. In particular there is some difficulty in coming up with a fair adjustment of the rating criteria to finite-volume regimes of QCD. For instance, in the $\epsilon$-regime^29^ we re-express the “chiral-extrapolation” criterion in terms of $\sqrt{2m_\mathrm {min}\Sigma }/F$, with the same threshold values (in MeV) between the three categories as in the *p*-regime. Also the “infinite-volume” assessment is adapted to the $\epsilon$-regime, since the $M_\pi L$ criterion does not make sense here; we assign a green star if at least 2 volumes with $L>2.5\,\mathrm{fm}$ are included, an open symbol if at least 1 volume with $L>2\,\mathrm{fm}$ is invoked and a red square if all boxes are smaller than $2\,\mathrm{fm}$. Similarly, in the calculation of form factors and charge radii the tables do not reflect whether an interpolation to the desired $q^2$ has been performed or whether the relevant $q^2$ has been engineered by means of “twisted boundary conditions” [339]. In spite of these limitations we feel that these tables give an adequate overview of the qualities of the various calculations.

#### Results for the LO *SU*(2) LECs

We begin with a discussion of the lattice results for the *SU*(2) LEC $\Sigma$. We present the results in Table 19 and Fig. 11. We add that results which include only a statistical error are listed in the table but omitted from the plot. Regarding the $N_{ f}=2$ computations there are six entries without a red tag. We form the average based on ETM 09C, ETM 13 (here we deviate from our “superseded” rule, since the two works use different methods), Brandt 13, and Engel 14. We add that the last one (with numbers identical to those given in Ref. [304]) is new compared to FLAG 13. Here and in the following we take into account that ETM 09C, ETM 13 share configurations, and the same statement holds true for Brandt 13 and Engel 14. Regarding the $N_{ f}=2+1$ computations there are four published or updated papers (MILC 10A, Borsanyi 12, BMW 13, and RBC/UKQCD 14B) which qualify for the $N_{ f}=2+1$ average. The last one is new compared to FLAG 13, and the last but one was not included in the FLAG 13 average, since at the time it was only a preprint.

In slight deviation from the general recipe outlined in Sect. 2.2 we use these values as a basis for our *estimates* (as opposed to *averages*) of the $N_{ f}=2$ and $N_{ f}=2+1$ condensates. In each case the central value is obtained from our standard averaging procedure, but the (symmetrical) error is just the median of the overall uncertainties of all contributing results (see the comment below for details). This leads to the values

94 $$\begin{aligned}&N_f=2 :&\Sigma ^{1/3}&= 266(10) \,\mathrm {MeV}&\,\mathrm {Refs.}~[33, 36{-}38],\nonumber \\&N_f=2+1:&\Sigma ^{1/3}&= 274( 3) \,\mathrm {MeV}&\,\mathrm {Refs.}~[10, 13, 34, 35], \end{aligned}$$

in the ${\overline{\text {MS}}}$ scheme at the renormalization scale $2\,\mathrm {GeV}$, where the errors include both statistical and systematic uncertainties. In accordance with our guidelines we ask the reader to cite the appropriate set of references as indicated in Eq. (94) when using these numbers. Finally, for $N_{ f}=2+1+1$ there is only one calculation available, the result of Ref. [33] as given in Table 19. According to the conventions of Sect. 2.2 this will be denoted as the “FLAG average” for $N_f=2+1+1$ in Fig. 11.

As a rationale for using *estimates* (as opposed to *averages*) for $N_{ f}=2$ and $N_{ f}=2+1$, we add that for $\Sigma ^{1/3}|_{N_{ f}=2}$ and $\Sigma ^{1/3}|_{N_{ f}=2+1}$ the standard averaging method would yield central values as quoted in Eq. (94), but with (overall) uncertainties of $4\,\mathrm {MeV}$ and $1\,\mathrm {MeV}$, respectively. It is not entirely clear to us that the scale is sufficiently well known in all contributing works to warrant a precision of up to 0.36% on our $\Sigma ^{1/3}$, and a similar statement can be made about the level of control over the convergence of the chiral expansion. The aforementioned uncertainties would suggest an $N_{ f}$-dependence of the *SU*(2) chiral condensate which (especially in view of similar issues with other LECs; see below) seems premature to us. Therefore we choose to form the central value of our estimate with the standard averaging procedure, but its uncertainty is taken as the median of the uncertainties of the participating results. We hope that future high-quality determinations with both $N_f=2$, $N_f=2+1$, and in particular with $N_f=2+1+1$, will help determine whether there is a noticeable $N_f$-dependence of the *SU*(2) chiral condensate or not.

The next quantity considered is *F*, i.e. the pion decay constant in the *SU*(2) chiral limit ($m_{ud}\rightarrow 0$, at fixed physical $m_s$ for $N_f > 2$ simulations). As argued on previous occasions we tend to give preference to $F_\pi /F$ (here the numerator is meant to refer to the physical-pion-mass point) wherever it is available, since often some of the systematic uncertainties are mitigated. We collect the results in Table 20 and Fig. 12. In those cases where the collaboration provides only *F*, the ratio is computed on the basis of the phenomenological value of $F_\pi$, and the respective entries in Table 20 are in slanted fonts. We encourage authors to provide both *F* and $F_\pi /F$ from their analysis, since the ratio is less dependent on the scale setting, and errors tend to partially cancel. Among the $N_{ f}=2$ determinations five (ETM 08, ETM 09C, QCDSF 13, Brandt 13 and Engel 14) are without red tags. Since the third one is without systematic error, only four of them enter the average. Compared to FLAG 13 the last work is the only one which is new. Among the $N_{ f}=2+1$ determinations five values (MILC 10 as an update of MILC 09, NPLQCD 11, Borsanyi 12, BMW 13, and RBC/UKQCD 14B) contribute to the average. Compared to FLAG 13 the last work is a new addition, and the last but one is included in the average for the first time. Here and in the following we take into account that MILC 10 and NPLQCD 11 share configurations. Finally, there is a single $N_{ f}=2+1+1$ determination (ETM 10) which forms the current best estimate in this category.

In analogy to the condensates discussed above, we use these values as a basis for our *estimates* (as opposed to *averages*) of the decay constant ratios

95 $$\begin{aligned}&N_f=2 :&{F_\pi }/{F}&=1.073(15)&\,\mathrm {Refs.}~ [36{-}38, 41] ,\nonumber \\&N_f=2+1:&{F_\pi }/{F}&=1.064( 7)&\,\mathrm {Refs.}~ [10, 29, 34, 35, 40], \end{aligned}$$

where the errors include both statistical and systematic uncertainties. These numbers are obtained through the well-defined procedure described subsequent to Eq. (94). We ask the reader to cite the appropriate set of references as indicated in Eq. (95) when using these numbers. Finally, for $N_{ f}=2+1+1$ the result of Ref. [39] is the only one available; see Table 20 for the numerical value.

For this observable the standard averaging method would yield the central values as quoted in Eq. (95), but with (overall) uncertainties of 6 and 1, respectively, on the last digit quoted. In this particular case the single $N_{ f}=2+1+1$ determination lies significantly higher than the $N_{ f}=2+1$ *average* (with the small error-bar), basically on par with the $N_{ f}=2$ *average* (with the small error-bar), and this makes such a standard *average* look even more suspicious to us. At the least, one should wait for one more qualifying $N_f=2+1+1$ determination before attempting any conclusions about the $N_f$ dependence of $F_\pi /F$. While we are not aware of any theorem which excludes a nonmonotonic behaviour in $N_f$ of a LEC, standard physics reasoning would suggest that quark-loop effects become smaller with increasing quark mass, hence a dynamical charm quark will influence LECs less significantly than a dynamical strange quark, and even the latter one seems to bring about rather small shifts. As a result, we feel that a nonmonotonic behaviour of $F_\pi /F$ with $N_{ f}$, once established, would represent a noteworthy finding. We hope this reasoning explains why we prefer to stay in Eq. (95) with *estimates* which obviously are on the conservative side.

#### Results for the NLO *SU*(2) LECs

We move on to a discussion of the lattice results for the NLO LECs $\bar{\ell }_3$ and $\bar{\ell }_4$. We remind the reader that on the lattice the former LEC is obtained as a result of the tiny deviation from linearity seen in $M_\pi ^2$ versus $Bm_{ud}$, whereas the latter LEC is extracted from the curvature in $F_\pi$ versus $Bm_{ud}$. The available determinations are presented in Table 21 and Fig. 13. Among the $N_{ f}=2$ determinations ETM 08, ETM 09C and Brandt 13 are published prior to the deadline, with a systematic uncertainty, and without red tags. Given that the former two use different approaches, all three determinations enter our average. The colour coding of the $N_{ f}=2+1$ results looks very promising; there is a significant number of lattice determinations without any red tag. Applying our superseding rule, MILC 10, NPLQCD 11, Borsanyi 12, BMW 13, and RBC/UKQCD 14B contribute to the average. Compared to the previous edition of our review, the last one is a new addition, and the last but one is included for the first time in the average. For $N_{ f}=2+1+1$ there is only the single work ETM 10.

In analogy to our processing of the LECs at LO, we use these determinations as the basis of our *estimate* (as opposed to *average*) of the NLO quantities

96 $$\begin{aligned}&N_f=2 :&\bar{\ell }_3&=3.41(82)&\,\mathrm {Refs.}~[36, 37, 41],\nonumber \\&N_f=2+1:&\bar{\ell }_3&=2.81(64)&\,\mathrm {Refs.}~[10, 29, 34, 35, 40], \end{aligned}$$

97 $$\begin{aligned}&N_f=2 :&\bar{\ell }_4&=4.51(26)&\,\mathrm {Refs.}~[36, 37, 41],\nonumber \\&N_f=2+1:&\bar{\ell }_4&=4.10(45)&\,\mathrm {Refs.}~[10, 29, 34, 35, 40], \end{aligned}$$

where the errors include both statistical and systematic uncertainties. These numbers are obtained through the well-defined procedure described next to Eq. (94). Again we ask the reader to cite the appropriate set of references as indicated in Eq. (96) or Eq. (97) when using these numbers. For $N_{ f}=2+1+1$ once again Ref. [39] is the single reference available; see Table 21 for the numerical values.

We remark that our preprocessing procedure^30^ symmetrizes the asymmetric error of ETM 09C with a slight adjustment of the central value. Regarding the difference between the *estimates* as given in Eqs. (96) and (97) and the result of the standard *averaging* procedure we add that the latter would yield the overall uncertainties 25 and 12 for $\bar{\ell }_3$, and the overall uncertainties 17 and 5 for $\bar{\ell }_4$. In all cases the central value would be unchanged. Especially for $\bar{\ell }_4$ such numbers would suggest a clear difference between the value with $N_{ f}=2$ dynamical flavours and the one at $N_{ f}=2+1$. Similarly to what happened with $F_\pi /F$, the single determination with $N_{ f}=2+1+1$ is more on the $N_{ f}=2$ side which, if confirmed, would suggest a nonmonotonicity of a $\chi$PT LEC with $N_{ f}$. Again we think that currently such a conclusion would be premature, and this is why we give preference to the *estimates* quoted in Eqs. (96) and (97).

From a more phenomenological point of view there is a notable difference between $\bar{\ell }_3$ and $\bar{\ell }_4$ in Fig. 13. For $\bar{\ell }_4$ the precision of the phenomenological determination achieved in Colangelo 01 [261] represents a significant improvement compared to Gasser 84 [131]. Picking any $N_{ f}$, the lattice estimate of $\bar{\ell }_4$ is consistent with both of the phenomenological values and comes with an error-bar which is roughly comparable to or somewhat larger than the one in Colangelo 01 [261]. By contrast, for $\bar{\ell }_3$ the error of an individual lattice computation is usually much smaller than the error of the estimate given in Gasser 84 [131], and even our conservative estimates (96) have uncertainties which represent a significant improvement on the error-bar of Gasser 84 [131]. Evidently, our hope is that future determinations of $\bar{\ell }_3,\bar{\ell }_4$, with $N_{ f}=2$, $N_{ f}=2+1$ and $N_{ f}=2+1+1$, will allow us to further shrink our error-bars in a future edition of FLAG.

We finish with a discussion of the lattice results for $\bar{\ell }_6$ and $\bar{\ell }_1{-}\bar{\ell }_2$. The LEC $\bar{\ell }_6$ determines the leading contribution in the chiral expansion of the pion vector charge radius; see Eq. (85). Hence from a lattice study of the vector form factor of the pion with several $M_\pi$ one may extract the radius $\langle r^2\rangle _V^\pi$, the curvature $c_V$ (both at the physical-pion-mass point) and the LEC $\bar{\ell }_6$ in one go. Similarly, the leading contribution in the chiral expansion of the scalar radius of the pion determines $\bar{\ell }_4$; see Eq. (85). This LEC is also present in the pion-mass dependence of $F_\pi$, as we have seen. The difference $\bar{\ell }_1{-}\bar{\ell }_2$, finally, may be obtained from the momentum dependence of the vector and scalar pion form factors, based on the two-loop formulae of Ref. [264]. The top part of Table 22 collects the results obtained from the vector form factor of the pion (charge radius, curvature and $\bar{\ell }_6$). Regarding this low-energy constant two $N_{ f}=2$ calculations are published works without a red tag; we thus arrive at the *average* (actually the first one in the LEC section)

98 $$\begin{aligned} N_f=2:\bar{\ell }_6=15.1(1.2) \quad \,\mathrm {Refs.}~[37, 41], \end{aligned}$$

which is represented as a grey band in the last panel of Fig. 13. Here we ask the reader to cite Refs. [37, 41] when using this number.

The experimental information concerning the charge radius is excellent and the curvature is also known very accurately, based on $e^+e^-$ data and dispersion theory. The vector form factor calculations thus present an excellent testing ground for the lattice methodology. The first data column of Table 22 shows that most of the available lattice results pass the test. There is, however, one worrisome point. For $\bar{\ell }_6$ the agreement seems less convincing than for the charge radius, even though the two quantities are closely related. In particular the $\bar{\ell }_6$ value of JLQCD 14 [360] seems inconsistent with the phenomenological determinations of Refs. [131, 264], even though its value for $\langle r^2\rangle _V^\pi$ is consistent. So far we have no explanation (other than observing that lattice computations which disagree with the phenomenological determination of $\bar{\ell }_6$ tend to have red tags), but we urge the groups to pay special attention to this point. Similarly, the bottom part of Table 22 collects the results obtained for the scalar form factor of the pion and the combination $\bar{\ell }_1{-}\bar{\ell }_2$ that is extracted from it. A new feature is that the (yet unpublished) paper of Ref. [336] gives both the (flavour) octet and the singlet parts in *SU*(3), finding $\langle r^2\rangle _{S,\mathrm {octet}}^\pi =0.431(38)(46)$ and $\langle r^2\rangle _{S,\mathrm {singlet}}^\pi =0.506(38)(53)$. For reasons of backward compatibility they also give $\langle r^2\rangle _{S,ud}^\pi$ defined with a $\bar{u}u+\bar{d}d$ density, and this number is shown in Table 22. Last but not least they find the ordering $\langle r^2\rangle _{S,\mathrm {conn}}^\pi< \langle r^2\rangle _{S,\mathrm {octet}}^\pi< \langle r^2\rangle _{S,ud}^\pi < \langle r^2\rangle _{S,\mathrm {singlet}}^\pi$ [336].

#### Epilogue

In this subsection there are several quantities for which only one qualifying (“all-green”) determination is available for a given *SU*(2) LEC. Obviously the phenomenologically oriented reader is encouraged to use such a value (as provided in our tables) and to cite the original work. We hope that the lattice community will come up with further computations, in particular for $N_{ f}=2+1+1$, such that a fair comparison of different works is possible at any $N_{ f}$, and eventually a statement can be made about the presence or absence of an $N_{ f}$-dependence of *SU*(2) LECs.

What can be learned about the convergence pattern of *SU*(2) $\chi$PT from varying the fit ranges (in $m_{ud}$) of the pion mass and decay constant (i.e. the quantities from which $\bar{\ell }_3,\bar{\ell }_4$ are derived) is discussed in Ref. [365], where also the usefulness of comparing results from the *x* and the $\xi$ expansion (with material taken from Ref. [35]) is emphasized.

Perhaps the most important physics result of this subsection is that the lattice simulations confirm the approximate validity of the Gell-Mann–Oakes–Renner formula and show that the square of the pion mass indeed grows in proportion to $m_{ud}$. The formula represents the leading term of the chiral series and necessarily receives corrections from higher orders. At first nonleading order, the correction is determined by the effective coupling constant $\bar{\ell }_3$. The results collected in Table 21 and in the top panel of Fig. 13 show that $\bar{\ell }_3$ is now known quite well. They corroborate the conclusion drawn already in Ref. [366]: the lattice confirms the estimate of $\bar{\ell }_3$ derived in Ref. [131]. In the graph of $M_\pi ^2$ versus $m_{ud}$, the values found on the lattice for $\bar{\ell }_3$ correspond to remarkably little curvature: the Gell-Mann–Oakes–Renner formula represents a reasonable first approximation out to values of $m_{ud}$ that exceed the physical value by an order of magnitude.

As emphasized by Stern et al. [367–369], the analysis in the framework of $\chi$PT is coherent only if (i) the leading term in the chiral expansion of $M_\pi ^2$ dominates over the remainder and (ii) the ratio $m_s/m_{ud}$ is close to the value 25.6, which follows from Weinberg’s leading-order formulae. In order to investigate the possibility that one or both of these conditions might fail, the authors proposed a more general framework, referred to as “generalized $\chi$PT”, which includes $\chi$PT as a special case. The results found on the lattice demonstrate that QCD does satisfy both of the above conditions – in the context of QCD, the proposed generalization of the effective theory does not appear to be needed. There is a modified version, however, referred to as “re-summed $\chi$PT” [370], which is motivated by the possibility that the Zweig-rule violating couplings $L_4$ and $L_6$ might be larger than expected. The available lattice data do not support this possibility, but they do not rule it out either (see Sect. 5.3 for details).

**Table 23:** Lattice results for the low-energy constants $F_0$, $B_0$ (in MeV) and $\Sigma _0\equiv F_0^2B_0$, which specify the effective *SU*(3) Lagrangian at leading order. The ratios $F/F_0$, $B/B_0$, $\Sigma /\Sigma _0$, which compare these with their *SU*(2) counterparts, indicate the strength of the Zweig-rule violations in these quantities (in the large-$N_c$ limit, they tend to unity). Numbers in slanted fonts are calculated by us, from the information given in the references

Collaboration	Refs.	$N_{ f}$	Publication status	Chiral extrapolation	Continuum extrapolation	Finite volume	$F_0$	$F/F_0$	$B/B_0$
JLQCD/TWQCD 10A	[338]	3	A				71(3)(8)		
MILC 10	[29]	$2+1$	C				80.3(2.5)(5.4)		
MILC 09A	[6]	$2+1$	C				78.3(1.4)(2.9)	*1.104(3)(41)*	1.21(4)$$\left( {\begin{array}{c}+5\\ -6\end{array}}\right) $$
MILC 09	[89]	$2+1$	A					1.15(5)$$\left( {\begin{array}{c}+13\\ -03\end{array}}\right) $$	1.15(16)$$\left( {\begin{array}{c}+39\\ -13\end{array}}\right) $$
PACS-CS 08	[93]	$2+1$	A				83.8(6.4)	1.078(44)	1.089(15)
RBC/UKQCD 08	[145]	$2+1$	A				66.1(5.2)	1.229(59)	1.03(05)

Collaboration	Refs.	$N_{ f}$	Publication status	Chiral extrapolation	Continuum extrapolation	Finite volume	Renormalization	$\Sigma _0^{1/3}$	$\Sigma /\Sigma _0$
JLQCD/TWQCD 10A	[338]	3	A					214(6)(24)	*1.31(13)(52)*
MILC 09A	[6]	$2+1$	C					245(5)(4)(4)	*1.48(9)(8)(10)*
MILC 09	[89]	$2+1$	A					242(9)$$\left( {\begin{array}{c}+05\\ -17\end{array}}\right) $$(4)	1.52(17)$$\left( {\begin{array}{c}+38\\ -15\end{array}}\right) $$
PACS-CS 08	[93]	$2+1$	A					290(15)	1.245(10)
RBC/UKQCD 08	[145]	$2+1$	A						1.55(21)

**Table 24:** Low-energy constants of the *SU*(3) Lagrangian at NLO with running scale $\mu =770~\,\mathrm {MeV}$ (the values in Refs. [6, 26, 29, 89, 129] are evolved accordingly). The MILC 10 entry for $L_6$ is obtained from their results for $2L_6-L_4$ and $L_4$ (similarly for other entries in slanted fonts). The JLQCD 08A result for $\ell _5(770~\,\mathrm {MeV})$ [despite the paper saying $L_{10}(770~\,\mathrm {MeV})$] was converted to $L_{10}$ with the GL one-loop formula, assuming that the difference between $\bar{\ell }_5(m_s=m_s^\mathrm {phys})$ [needed in the formula] and $\bar{\ell }_5(m_s=\infty )$ [computed by JLQCD] is small

Collaboration	Refs.	$N_{ f}$	Publication status	Chiral extrapolation	Continuum extrapolation	Finite volume	$10^3L_4$	$10^3L_6$	$10^3(2L_6-L_4)$
HPQCD 13A	[26]	$2+1+1$	A				0.09(34)	0.16(20)	0.22(17)
JLQCD/TWQCD 10A	[338]	3	A					0.03(7)(17)	
MILC 10	[29]	$2+1$	C				$-$0.08(22)$$\left( {\begin{array}{c}+57\\ -33\end{array}}\right) $$	$-$0.02(16)$$\left( {\begin{array}{c}+33\\ -21\end{array}}\right) $$	0.03(24)$$\left( {\begin{array}{c}+32\\ -27\end{array}}\right) $$
MILC 09A	[6]	$2+1$	C				0.04(13)(4)	0.07(10)(3)	0.10(12)(2)
MILC 09	[89]	$2+1$	A				0.1(3)$$\left( {\begin{array}{c}+3\\ -1\end{array}}\right) $$	0.2(2)$$\left( {\begin{array}{c}+2\\ -1\end{array}}\right) $$	0.3(1)$$\left( {\begin{array}{c}+2\\ -3\end{array}}\right) $$
PACS-CS 08	[93]	$2+1$	A				$-$0.06(10)(–)	*0.02(5)(–)*	0.10(2)(–)
RBC/UKQCD 08	[145]	$2+1$	A				0.14(8)(–)	0.07(6)(–)	0.00(4)(–)
Bijnens 11	[268]						0.75(75)	0.29(85)	$-$ *0.17(1.86)*
Gasser 85	[129]						$-$0.3(5)	$-$0.2(3)	$-$ *0.1(8)*

Collaboration	Refs.	$N_{ f}$					$10^3L_5$	$10^3L_8$	$10^3(2L_8-L_5)$
HPQCD 13A	[26]	$2+1+1$	A				1.19(25)	0.55(15)	$-$0.10(20)
MILC 10	[29]	$2+1$	C				0.98(16)$$\left( {\begin{array}{c}+28\\ -41\end{array}}\right) $$	0.42(10)$$\left( {\begin{array}{c}+27\\ -23\end{array}}\right) $$	$-$0.15(11)$$\left( {\begin{array}{c}+45\\ -19\end{array}}\right) $$
MILC 09A	[6]	$2+1$	C				0.84(12)(36)	0.36(5)(7)	$-$0.12(8)(21)
MILC 09	[89]	$2+1$	A				1.4(2)$$\left( {\begin{array}{c}+2\\ -1\end{array}}\right) $$	0.8(1)(1)	0.3(1)(1)
PACS-CS 08	[93]	$2+1$	A				1.45(7)(–)	*0.62(4)(–)*	$-$0.21(3)(–)
RBC/UKQCD 08	[145]	$2+1$	A				0.87(10)(–)	0.56(4)(–)	0.24(4)(–)
NPLQCD 06	[238]	$2+1$	A				1.42(2)$$\left( {\begin{array}{c}+18\\ -54\end{array}}\right) $$		
Bijnens 11	[268]						0.58(13)	0.18(18)	$-$ *0.22(38)*
Gasser 85	[129]						1.4(5)	0.9(3)	*0.4(8)*

Collaboration	Refs.	$N_{ f}$					$10^3L_9$	$10^3L_{10}$	
Boito 15	[371]	$2+1$	P					$-$3.50(17)	
JLQCD 15A	[359]	$2+1$	P				4.6(1.1)$$\left( {\begin{array}{c}+0.1\\ -0.5\end{array}}\right) $$(0.4)		
Boyle 14	[372]	$2+1$	A					$-$3.46(32)	
JLQCD 14	[360]	$2+1$	A				2.4(0.8)(1.0)		
RBC/UKQCD 09	[373]	$2+1$	A					$-$5.7(11)(07)	
RBC/UKQCD 08A	[339]	$2+1$	A				3.08(23)(51)		
JLQCD 08A	[374]	2	A					$-$5.2(2)$$\left( {\begin{array}{c}+5\\ -3\end{array}}\right) $$	
Bijnens 02	[375]						5.93(43)		
Davier 98	[376]							$-$5.13(19)	
Gasser 85	[129]						6.9(7)	$-$5.5(7)	

### Extraction of *SU*(3) low-energy constants

To date, there are three comprehensive *SU*(3) papers with results based on lattice QCD with $N_{ f}\!=\!2+1$ dynamical flavours [89, 93, 145], and one more with results based on $N_{ f}\!=\!2+1+1$ dynamical flavours [26]. It is an open issue whether the data collected at $m_s \simeq m_s^\mathrm {phys}$ allow for an unambiguous determination of *SU*(3) low-energy constants (cf. the discussion in Ref. [145]). To make definite statements one needs data at considerably smaller $m_s$, and so far only MILC has some [89]. We are aware of a few papers with a result on one *SU*(3) low-energy constant each which we list for completeness. Some particulars of the computations are listed in Table 23.

Results for the *SU*(3) low-energy constants of leading order are found in Table 23 and analogous results for some of the effective coupling constants that enter the chiral *SU*(3) Lagrangian at NLO are collected in Table 24. From PACS-CS [93] only those results are quoted which have been *corrected* for finite-size effects (misleadingly labelled “w/FSE” in their tables). For staggered data our colour-coding rule states that $M_\pi$ is to be understood as $M_\pi ^\mathrm {RMS}$. The rating of Refs. [29, 89] is based on the information regarding the RMS masses given in Ref. [6]. Finally, Refs. [371, 372] are “hybrids” in the sense that they combine lattice data and experimental information.

**Fig. 14:**
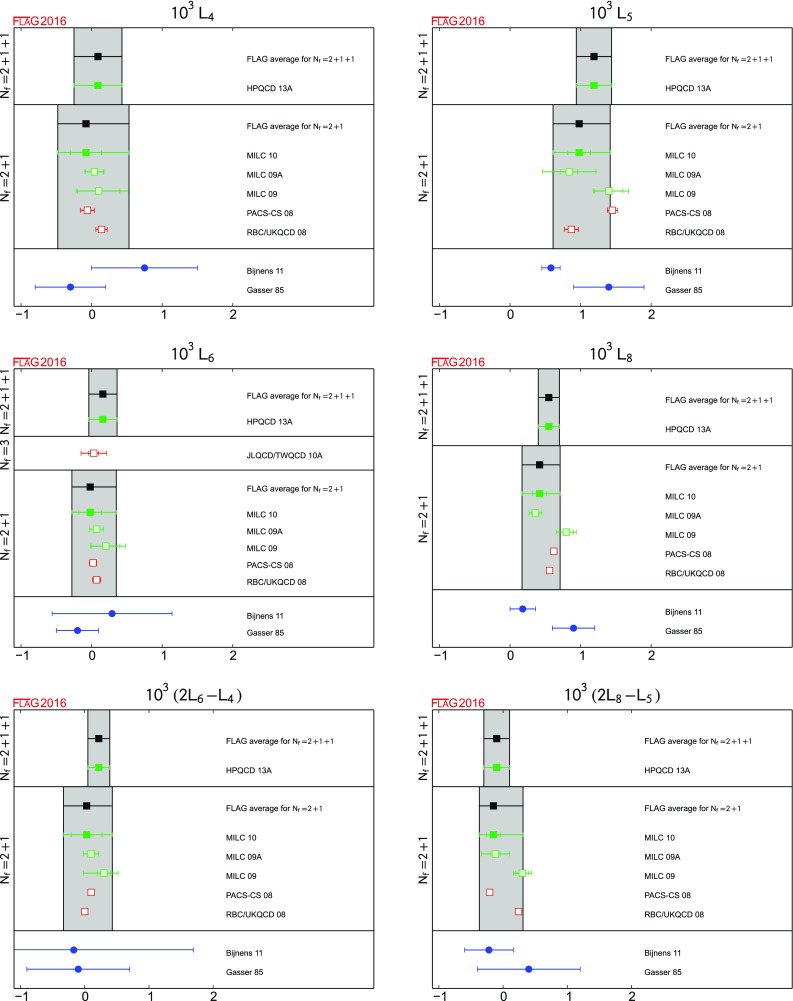
Low-energy constants that enter the effective *SU*(3) Lagrangian at NLO, with scale $\mu =770\,\mathrm {MeV}$. The *grey bands* labelled as “FLAG average” coincide with the results of MILC 10 [29] for $N_{ f}=2+1$ and with HPQCD 13A [26] for $N_{ f}=2+1+1$, respectively

A graphical summary of the lattice results for the coupling constants $L_4$, $L_5$, $L_6$ and $L_8$, which determine the masses and the decay constants of the pions and kaons at NLO of the chiral *SU*(3) expansion, is displayed in Fig. 14, along with the two phenomenological determinations quoted in the above tables. The overall consistency seems fairly convincing. In spite of this apparent consistency, there is a point which needs to be clarified as soon as possible. Some collaborations (RBC/UKQCD and PACS-CS) find that they are having difficulties in fitting their partially quenched data to the respective formulae for pion masses above $\simeq$400 MeV. Evidently, this indicates that the data are stretching the regime of validity of these formulae. To date it is, however, not clear which subset of the data causes the troubles, whether it is the unitary part extending to too large values of the quark masses or whether it is due to $m^\mathrm {val}/m^\mathrm {sea}$ differing too much from one. In fact, little is known, in the framework of partially quenched $\chi$PT, about the *shape* of the region of applicability in the $m^\mathrm {val}$ versus $m^\mathrm {sea}$ plane for fixed $N_{ f}$. This point has also been emphasized in Ref. [326].

To date only the computations MILC 10 [29] (as an obvious update of MILC 09 and MILC 09A) and HPQCD 13A [26] are free of red tags. Since they use different $N_{ f}$ (in the former case $N_{ f}=2+1$, in the latter case $N_{ f}=2+1+1$) we stay away from averaging them. Hence the situation remains unsatisfactory in the sense that for each $N_{ f}$ only a single determination of high standing is available. Accordingly, for the phenomenologically oriented reader there is no alternative to using the results of MILC 10 [29] for $N_{ f}=2+1$ and HPQCD 13A [26] for $N_{ f}=2+1+1$, as given in Table 24.

#### Epilogue

In this subsection we find ourselves again in the unpleasant situation that only one qualifying (“all-green”) determination is available (at a given $N_{ f}$) for several LECs in the *SU*(3) framework, both at LO and at NLO. Obviously the phenomenologically oriented reader is encouraged to use such a value (as provided in our tables) and to cite the original work. Again our hope is that further computations would become available in forthcoming years, such that a fair comparison of different works will become possible both at $N_{ f}=2+1$ and $N_{ f}=2+1+1$.

In the large-$N_c$ limit, the Zweig rule becomes exact, but the quarks have $N_c=3$. The work done on the lattice is ideally suited to confirm or disprove the approximate validity of this rule for QCD. Two of the coupling constants entering the effective *SU*(3) Lagrangian at NLO disappear when $N_c$ is sent to infinity: $L_4$ and $L_6$. The upper part of Table 24 and the left panels of Fig. 14 show that the lattice results for these quantities are in good agreement. At the scale $\mu =M_\rho$, $L_4$ and $L_6$ are consistent with zero, indicating that these constants do approximately obey the Zweig rule. As mentioned above, the ratios $F/F_0$, $B/B_0$ and $\Sigma /\Sigma _0$ also test the validity of this rule. Their expansion in powers of $m_s$ starts with unity and the contributions of first order in $m_s$ are determined by the constants $L_4$ and $L_6$, but they also contain terms of higher order. Apart from measuring the Zweig-rule violations, an accurate determination of these ratios will thus also allow us to determine the range of $m_s$ where the first few terms of the expansion represent an adequate approximation. Unfortunately, at present, the uncertainties in the lattice data on these ratios are too large to draw conclusions, both concerning the relative size of the subsequent terms in the chiral series and concerning the magnitude of the Zweig-rule violations. The data seem to confirm the *paramagnetic inequalities* [369], which require $F/F_0>1$, $\Sigma /\Sigma _0>1$, and it appears that the ratio $B/B_0$ is also larger than unity, but the numerical results need to be improved before further conclusions can be drawn.

The matching formulae in Ref. [129] can be used to calculate the *SU*(2) couplings $\bar{l}_i$ from the *SU*(3) couplings $L_j$. Results obtained in this way are included in Table 21, namely the entries explicitly labelled “*SU*(3)-fit” as well as MILC 10. Within the still rather large errors, the converted LECs from the *SU*(3) fits agree with those directly determined within *SU*(2) $\chi$PT. We plead with every collaboration performing $N_{ f}=2+1$ simulations to also *directly* analyse their data in the *SU*(2) framework. In practice, lattice simulations are performed at values of $m_s$ close to the physical value and the results are then corrected for the difference of $m_s$ from its physical value. If simulations with more than one value of $m_s$ have been performed, this can be done by interpolation. Alternatively one can use the technique of *reweighting* (for a review see e.g. Ref. [377]) to shift $m_s$ to its physical value.

## Kaon mixing

The mixing of neutral pseudoscalar mesons plays an important role in the understanding of the physics of CP violation. In this section we discuss $K^0{-}\bar{K}^0$ oscillations, which probe the physics of indirect CP violation. Extensive reviews on the subject can be found in Refs. [378–380]. For the most part we shall focus on kaon mixing in the SM. The case of Beyond-the-Standard-Model (BSM) contributions is discussed in Sect. 6.3.

### Indirect CP violation and $\epsilon _{K}$ in the SM

Indirect CP violation arises in $K_L \rightarrow \pi \pi$ transitions through the decay of the $\mathrm CP=+1$ component of $K_L$ into two pions (which are also in a $\mathrm CP=+1$ state). Its measure is defined as

99 $$\begin{aligned} \epsilon _{K}= \dfrac{\mathcal{A} [ K_L \rightarrow (\pi \pi )_{I=0}]}{\mathcal{A} [ K_S \rightarrow (\pi \pi )_{I=0}]}, \end{aligned}$$

with the final state having total isospin zero. The parameter $\epsilon _{K}$ may also be expressed in terms of $K^0{-}\bar{K}^0$ oscillations. In particular, to lowest order in the electroweak theory, the contribution to these oscillations arises from so-called box diagrams, in which two *W* bosons and two “up-type” quarks (i.e. up, charm, top) are exchanged between the constituent down and strange quarks of the *K* mesons. The loop integration of the box diagrams can be performed exactly. In the limit of vanishing external momenta and external quark masses, the result can be identified with an effective four-fermion interaction, expressed in terms of the “effective Hamiltonian”

100 $$\begin{aligned} \mathcal{H}_\mathrm{eff}^{\Delta S = 2}= \frac{G_F^2 M_\mathrm{{W}}^2}{16\pi ^2} \mathcal{F}^0 Q^{\Delta S=2}+\mathrm{h.c.} \end{aligned}$$

In this expression, $G_F$ is the Fermi coupling, $M_\mathrm{{W}}$ the *W*-boson mass, and

101 $$\begin{aligned} Q^{\Delta S=2}= & {} \left[ \bar{s}\gamma _\mu (1-\gamma _5)d\right] \left[ \bar{s}\gamma _\mu (1-\gamma _5)d\right] \nonumber \\\equiv & {} O_{\mathrm{VV}+\mathrm{AA}}-O_{\mathrm{VA}+\mathrm{AV}}, \end{aligned}$$

is a dimension-six, four-fermion operator. The function $\mathcal{F}^0$ is given by

102 $$\begin{aligned} \mathcal{F}^0 =\lambda _c^2 S_0(x_c) +\lambda _t^2 S_0(x_t) +2 \lambda _c \lambda _t S_0(x_c,x_t), \end{aligned}$$

where $\lambda _a = V^*_{as} V_{ad}$, and $a=c,t$ denotes a flavour index. The quantities $S_0(x_c),\,S_0(x_t)$ and $S_0(x_c,x_t)$ with $x_c=m_c^2/M_\mathrm{{W}}^2$, $x_t=m_t^2/M_\mathrm{{W}}^2$ are the Inami–Lim functions [381], which express the basic electroweak loop contributions without QCD corrections. The contribution of the up quark, which is taken to be massless in this approach, has been taken into account by imposing the unitarity constraint $\lambda _u + \lambda _c + \lambda _t = 0$.

When strong interactions are included, $\Delta {S}=2$ transitions can no longer be discussed at the quark level. Instead, the effective Hamiltonian must be considered between mesonic initial and final states. Since the strong coupling is large at typical hadronic scales, the resulting weak matrix element cannot be calculated in perturbation theory. The operator product expansion (OPE) does, however, factorize long- and short-distance effects. For energy scales below the charm threshold, the $K^0{-}\bar{K}^0$ transition amplitude of the effective Hamiltonian can be expressed as

103 $$\begin{aligned} \langle \bar{K}^0 \vert \mathcal{H}_\mathrm{eff}^{\Delta S = 2} \vert K^0 \rangle= & {} \frac{G_F^2 M_\mathrm{{W}}^2}{16 \pi ^2}[ \lambda _c^2 S_0(x_c) \eta _1 +\lambda _t^2 S_0(x_t) \eta _2\nonumber \\&+ \, 2 \lambda _c \lambda _t S_0(x_c,x_t) \eta _3] \times \left( \frac{\bar{g}(\mu )^2}{4\pi }\right) ^{-\gamma _0/(2\beta _0)}\nonumber \\&\times \exp \left\{ \int _0^{\bar{g}(\mu )} \, \mathrm{d}g \, \left( \frac{\gamma (g)}{\beta (g)}+\frac{\gamma _0}{\beta _0g} \right) \right\} \nonumber \\&\times \langle \bar{K}^0 \vert Q^{\Delta S=2}_\mathrm{R} (\mu ) \vert K^0 \rangle +\mathrm{h.c.}, \end{aligned}$$

where $\bar{g}(\mu )$ and $Q^{\Delta S=2}_\mathrm{R}(\mu )$ are the renormalized gauge coupling and four-fermion operator in some renormalization scheme. The factors $\eta _1, \eta _2$ and $\eta _3$ depend on the renormalized coupling $\bar{g}$, evaluated at the various flavour thresholds $m_t, m_b, m_c$ and $M_\mathrm{{W}}$, as required by the OPE and RG-running procedure that separate high- and low-energy contributions. Explicit expressions can be found in Ref. [379] and references therein, except that $\eta _1$ and $\eta _3$ have been recently calculated to NNLO in Refs. [382] and [383], respectively. We follow the same conventions for the RG equations as in Ref. [379]. Thus the Callan–Symanzik function and the anomalous dimension $\gamma (\bar{g})$ of $Q^{\Delta S=2}$ are defined by

104 $$\begin{aligned} \dfrac{\mathrm{d} \bar{g}}{\mathrm{d} \ln \mu } = \beta (\bar{g}),\quad \dfrac{\mathrm{d} Q^{\Delta S=2}_\mathrm{R}}{\mathrm{d} \ln \mu } = -\gamma (\bar{g})\,Q^{\Delta S=2}_\mathrm{R}, \end{aligned}$$

with perturbative expansions

105 $$\begin{aligned} \beta (g)= & {} -\beta _0 \dfrac{g^3}{(4\pi )^2} -\beta _1 \dfrac{g^5}{(4\pi )^4} -\cdots \\ \gamma (g)= & {} \gamma _0 \dfrac{g^2}{(4\pi )^2} +\gamma _1 \dfrac{g^4}{(4\pi )^4} + \cdots .\nonumber \end{aligned}$$

We stress that $\beta _0, \beta _1$ and $\gamma _0$ are universal, i.e. scheme independent. $K^0{-}\bar{K}^0$ mixing is usually considered in the naive dimensional regularization (NDR) scheme of ${\overline{\text {MS}}}$, and below we specify the perturbative coefficient $\gamma _1$ in that scheme:

106 $$\begin{aligned}&\beta _0 = \left\{ \frac{11}{3}N-\frac{2}{3}N_{ f}\right\} ,\nonumber \\&\beta _1 = \left\{ \frac{34}{3}N^2-N_{ f}\left( \frac{13}{3}N-\frac{1}{N} \right) \right\} , \\&\gamma _0 = \frac{6(N-1)}{N},\nonumber \\&\gamma _1 = \frac{N-1}{2N} \left\{ -21 + \frac{57}{N} - \frac{19}{3}N + \frac{4}{3}N_{ f}\right\} .\nonumber \end{aligned}$$

Note that for QCD the above expressions must be evaluated for $N=3$ colours, while $N_{ f}$ denotes the number of active quark flavours. As already stated, Eq. (103) is valid at scales below the charm threshold, after all heavier flavours have been integrated out, i.e. $N_{ f}= 3$.

In Eq. (103), the terms proportional to $\eta _1,\,\eta _2$ and $\eta _3$, multiplied by the contributions containing $\bar{g}(\mu )^2$, correspond to the Wilson coefficient of the OPE, computed in perturbation theory. Its dependence on the renormalization scheme and scale $\mu$ is canceled by that of the weak matrix element $\langle \bar{K}^0 \vert Q^{\Delta S=2}_\mathrm{R} (\mu ) \vert K^0 \rangle$. The latter corresponds to the long-distance effects of the effective Hamiltonian and must be computed nonperturbatively. For historical, as well as technical reasons, it is convenient to express it in terms of the *B* parameter $B_\mathrm{{K}}$, defined as

107 $$\begin{aligned} B_\mathrm{{K}}(\mu )= \frac{{\left\langle \bar{K}^0\left| Q^{\Delta S=2}_\mathrm{R}(\mu )\right| K^0\right\rangle } }{ {\frac{8}{3}f_\mathrm{K}^2m_\mathrm{K}^2}}. \end{aligned}$$

The four-quark operator $Q^{\Delta S=2}(\mu )$ is renormalized at scale $\mu$ in some regularization scheme, for instance, NDR-${\overline{\text {MS}}}$. Assuming that $B_\mathrm{{K}}(\mu )$ and the anomalous dimension $\gamma (g)$ are both known in that scheme, the renormalization group independent (RGI) *B* parameter $\hat{B}_\mathrm{K}$ is related to $B_\mathrm{{K}}(\mu )$ by the exact formula

108 $$\begin{aligned} \hat{B}_\mathrm{{K}}= & {} \left( \frac{\bar{g}(\mu )^2}{4\pi }\right) ^{-\gamma _0/(2\beta _0)}\nonumber \\&\times \exp \left\{ \int _0^{\bar{g}(\mu )} \, \mathrm{d}g \, \left( \frac{\gamma (g)}{\beta (g)}+\frac{\gamma _0}{\beta _0g} \right) \right\} \, B_\mathrm{{K}}(\mu ). \end{aligned}$$

At NLO in perturbation theory the above reduces to

109 $$\begin{aligned} \hat{B}_\mathrm{{K}}= & {} \left( \frac{\bar{g}(\mu )^2}{4\pi }\right) ^{- \gamma _0/(2\beta _0)}\nonumber \\&\times \left\{ 1+\dfrac{\bar{g}(\mu )^2}{(4\pi )^2}\left[ \frac{\beta _1\gamma _0-\beta _0\gamma _1}{2\beta _0^2} \right] \right\} \, B_\mathrm{{K}}(\mu ). \end{aligned}$$

To this order, this is the scale-independent product of all $\mu$-dependent quantities in Eq. (103).

Lattice QCD calculations provide results for $B_K(\mu )$. These results are, however, usually obtained in intermediate schemes other than the continuum ${\overline{\text {MS}}}$ scheme used to calculate the Wilson coefficients appearing in Eq. (103). Examples of intermediate schemes are the RI/MOM scheme [384] (also dubbed the “Rome–Southampton method”) and the Schrödinger functional (SF) scheme [153]. These schemes are used as they allow a nonperturbative renormalization of the four-fermion operator, using an auxiliary lattice simulation. This allows $B_K(\mu )$ to be calculated with percent-level accuracy, as described below.

In order to make contact with phenomenology, however, and in particular to use the results presented above, one must convert from the intermediate scheme to the ${\overline{\text {MS}}}$ scheme or to the RGI quantity $\hat{B}_\mathrm{K}$. This conversion relies on one or two-loop perturbative matching calculations, the truncation errors in which are, for many recent calculations, the dominant source of error in $\hat{B}_\mathrm{{K}}$ (see, for instance, Refs. [10, 31, 44, 45, 385]). While this scheme-conversion error is not, strictly speaking, an error of the lattice calculation itself, it must be included in results for the quantities of phenomenological interest, namely $B_K({\overline{\text {MS}}},2\,\mathrm{GeV})$ and $\hat{B}_\mathrm{K}$. We note that this error can be minimized by matching between the intermediate scheme and ${\overline{\text {MS}}}$ at as large a scale $\mu$ as possible (so that the coupling which determines the rate of convergence is minimized). Recent calculations have pushed the matching $\mu$ up to the range $3{-}3.5\,$GeV. This is possible because of the use of nonperturbative RG running determined on the lattice [10, 31, 43]. The Schrödinger functional offers the possibility to run nonperturbatively to scales $\mu \sim M_\mathrm{{W}}$ where the truncation error can be safely neglected. However, so far this has been applied only for two flavours of Wilson quarks [386].

Perturbative truncation errors in Eq. (103) also affect the Wilson coefficients $\eta _1$, $\eta _2$ and $\eta _3$. It turns out that the largest uncertainty comes from that in $\eta _1$ [382]. Although it is now calculated at NNLO, the series shows poor convergence. The net effect is that the uncertainty in $\eta _1$ is larger than that in present lattice calculations of $B_K$.

In the Standard Model, $\epsilon _{K}$ receives contributions from: (1) short-distance physics given by $\Delta S = 2$ “box diagrams” involving $W^\pm$ bosons and *u*, *c* and *t* quarks; (2) long distance physics from light hadrons contributing to the imaginary part of the dispersive amplitude $M_{12}$ used in the two component description of $K^0{-}\bar{K}^0$ mixing; (3) the imaginary part of the absorptive amplitude $\Gamma _{12}$ from $K^0{-}\bar{K}^0$ mixing; and (4) $\text {Im}(A_0)/\text {Re}(A_0)$. The terms in this decomposition can vary with phase conventions. It is common to represent contribution 1 by

110 $$\begin{aligned} \text {Im}(M_{12}^\text {SD}) \equiv \frac{1}{2m_K}\text {Im} [ \langle \bar{K}^0 | \mathcal{H}_\text {eff}^{\Delta S = 2} | K^0 \rangle ]^*\end{aligned}$$

and contribution 2 by $M_{12}^\text {LD}$. Contribution 3 can be related to $\text {Im}(A_0)/\text {Re}(A_0)$, yielding [380, 387–390]

111 $$\begin{aligned} \epsilon _K= & {} \exp (i \phi _{\epsilon }) \, \sin (\phi _{\epsilon }) \nonumber \\&\times \left[ \frac{\text {Im}(M_{12}^\text {SD})}{\Delta M_K} + \frac{\text {Im}(M_{12}^\text {LD})}{\Delta M_K} + \frac{\text {Im}(A_0)}{\text {Re}(A_0)} \right] \end{aligned}$$

for $\lambda _u$ real and positive; the phase of $\epsilon _{K}$ is given by

112 $$\begin{aligned} \phi _{\epsilon } =\arctan \frac{\Delta M_{K}}{\Delta \Gamma _{K}/2}. \end{aligned}$$

The quantities $\Delta M_K$ and $\Delta \Gamma _K$ are the mass and decay width differences between long- and short-lived neutral kaons, while $A_0$ is the amplitude of the kaon decay into an isospin-0 two pion state. Experimentally known values of the above quantities are [151]:

113 $$\begin{aligned}&\vert \epsilon _{K} \vert = 2.228(11) \times 10^{-3}, \nonumber \\&\phi _{\epsilon } = 43.52(5)^\circ , \\&\Delta M_{K} = 3.4839(59) \times 10^{-12}~\mathrm{MeV}, \nonumber \\&\Delta \Gamma _{K} = 7.3382(33) \times 10^{-15}~\mathrm{GeV}.\nonumber \end{aligned}$$

A recent analytical estimate of the contributions of $M_{12}^\text {LD}$ (Refs. [389, 390]) leads to

114 $$\begin{aligned} \epsilon _{K} =\exp (i \phi _{\epsilon }) \, \sin (\phi _{\epsilon }) \, \left[ \frac{\text {Im}(M_{12}^\mathrm{SD})}{\Delta M_K } +\rho \frac{\text {Im}(A_0)}{\text {Re}(A_0)}\right] . \end{aligned}$$

A phenomenological estimate for $\xi =\mathrm{Im}\,(A_0)/\mathrm{Re}\,(A_0)$ can be determined using the experimental value of $\epsilon ^\prime /\epsilon$ [390]

115 $$\begin{aligned} \xi = -6.0(1.5)\times 10^{-4}\sqrt{2}|\epsilon _K| = -1.9(5)\times 10^{-4}. \end{aligned}$$

A more precise result has been obtained from the ratio of amplitudes $\mathrm{Im}\,(A_2)/\mathrm{Re}\,(A_2)$ computed in lattice QCD [391] (where $A_2$ denotes the $\Delta {I}=3/2$ decay amplitude for $K\rightarrow \pi \pi$):

116 $$\begin{aligned} \xi = -1.6(2)\times 10^{-4}. \end{aligned}$$

The value of $\xi$ can then be combined with a ${\chi }\mathrm PT$-based estimate for the long-range contribution, i.e. $\rho =0.6(3)$ [390]. Overall, the combination $\rho \xi$ leads to a suppression of $|\epsilon _K|$ by $6(2)\%$ relative to the naive estimate (i.e. the first term in square brackets in Eq. (111)), regardless of whether the phenomenological or lattice estimate for $\xi$ is used. The uncertainty in the suppression factor is dominated by the error on $\rho$. Although this is a small correction, we note that its contribution to the error of $\epsilon _K$ is larger than that arising from the value of $B_\mathrm{K}$ reported below.

Efforts are under way to compute both the real and the imaginary long-distance contributions to the $K_L{-}K_S$ mass difference in lattice QCD [392–394]. However, the results are not yet precise enough to improve the accuracy in the determination of the parameter $\rho$.

### Lattice computation of $B_\mathrm{{K}}$

Lattice calculations of $B_\mathrm{{K}}$ are affected by the same systematic effects discussed in previous sections. However, the issue of renormalization merits special attention. The reason is that the multiplicative renormalizability of the relevant operator $Q^{\Delta S=2}$ is lost once the regularized QCD action ceases to be invariant under chiral transformations. For Wilson fermions, $Q^{\Delta S=2}$ mixes with four additional dimension-six operators, which belong to different representations of the chiral group, with mixing coefficients that are finite functions of the gauge coupling. This complicated renormalization pattern was identified as the main source of systematic error in earlier, mostly quenched calculations of $B_\mathrm{{K}}$ with Wilson quarks. It can be bypassed via the implementation of specifically designed methods, which are either based on Ward identities [395] or on a modification of the Wilson quark action, known as twisted-mass QCD [396, 397].

An advantage of staggered fermions is the presence of a remnant *U*(1) chiral symmetry. However, at nonvanishing lattice spacing, the symmetry among the extra unphysical degrees of freedom (tastes) is broken. As a result, mixing with other dimension-six operators cannot be avoided in the staggered formulation, which complicates the determination of the *B* parameter. The effects of the broken taste symmetry are usually treated via an effective field theory, such as staggered Chiral Perturbation Theory (S$\chi$PT).

**Table 25:** Results for the Kaon *B* parameter in QCD with $N_{ f}=2+1+1$ and $N_{ f}=2+1$ dynamical flavours, together with a summary of systematic errors. Any available information as regards nonperturbative running is indicated in the column “running”, with details given at the bottom of the table

Collaboration	Refs.	$N_{ f}$	Publication status	Continuum extrapolation	Chiral extrapolation	Finite volume	Renormalization	Running	$B_{{K}}(\overline{\mathrm{MS}},2~\mathrm{GeV})$	$\hat{B}_{{K}}$
ETM 15	[42]	$2+1+1$	A					$\,a$	0.524(13)(12)	0.717(18)(16)$^{*}$
SWME 15A	[45]	$2+1$	A				$^{\dagger }$	−	0.537(4)(26)	0.735(5)(36)¶
RBC/UKQCD 14B	[10]	$2+1$	A					$\,b$	0.5478(18)(110)$^{\S }$	0.7499(24)(150)
SWME 14	[385]	$2+1$	A				$^{\dagger }$	−	0.5388(34)(266)	0.7379(47)(365)
SWME 13A	[402]	$2+1$	A				$^{\dagger }$	−	0.537(7)(24)	0.735(10)(33)
SWME 13	[403]	$2+1$	C				$^{\dagger }$	−	0.539(3)(25)	0.738(5)(34)
RBC/UKQCD 12A	[31]	$2+1$	A					$\,b$	0.554(8)(14)$^{\S }$	0.758(11)(19)
Laiho 11	[44]	$2+1$	C					−	0.5572(28)(150)	0.7628(38)(205)¶
SWME 11A	[404]	$2+1$	A				$^{\dagger }$	−	0.531(3)(27)	0.727(4)(38)
BMW 11	[43]	$2+1$	A					$\,c$	0.5644(59)(58)	0.7727(81)(84)
RBC/UKQCD 10B	[405]	$2+1$	A					$\,d$	0.549(5)(26)	0.749(7)(26)
SWME 10	[278]	$2+1$	A					−	0.529(9)(32)	0.724(12)(43)
Aubin 09	[406]	$2+1$	A					−	0.527(6)(21)	0.724(8)(29)
RBC/UKQCD 07A, 08	[145, 407]	$2+1$	A					−	0.524(10)(28)	0.720(13)(37)
HPQCD/UKQCD 06	[408]	$2+1$	A		$^{\ddagger }$			−	0.618(18)(135)	0.83(18)

*a* $B_K$ is renormalized nonperturbatively at scales $1/a \sim 2.2{-}3.3~\mathrm{GeV}$ in the $N_{ f}= 4$ RI/MOM scheme using two different lattice momentum scale intervals, the first around 1 / *a* while the second around 3.5 GeV. The impact of the two ways to the final result is taken into account in the error budget. Conversion to ${\overline{\text {MS}}}$ is at one-loop at 3 GeV

*b* $B_K$ is renormalized nonperturbatively at a scale of 1.4 GeV in two RI/SMOM schemes for $N_{ f}= 3$, and then run to 3 GeV using a nonperturbatively determined step-scaling function. Conversion to ${\overline{\text {MS}}}$ is at one-loop order at 3 GeV

*c* $B_K$ is renormalized and run nonperturbatively to a scale of $3.4~\mathrm{GeV}$ in the RI/MOM scheme. nonperturbative and NLO perturbative running agrees down to scales of $1.8~\mathrm{GeV}$ within statistical uncertainties of about 2%

*d* $B_K$ is renormalized nonperturbatively at a scale of 2 GeV in two RI/SMOM schemes for $N_{ f}= 3$, and then run to 3 GeV using a nonperturbatively determined step-scaling function. Conversion to ${\overline{\text {MS}}}$ is at one-loop order at 3 GeV

$^{\dagger }$ The renormalization is performed using perturbation theory at one loop, with a conservative estimate of the uncertainty

$^{{\ddagger }}$ This result has been obtained with only two “light” sea-quark masses

$^{*}$ $B_{K}({\overline{\text {MS}}}, 2~\mathrm{GeV})$ and $\hat{B}_{{K}}$ are related using the conversion factor 1.369 i.e. the one obtained with $N_f=2+1$

¶ $\hat{B}_{{K}}$ is obtained from the estimate for $B_{K}({\overline{\text {MS}}}, 2~\mathrm{GeV})$ using the conversion factor 1.369

$^{\S }$ $B_{K}({\overline{\text {MS}}}, 2~\mathrm{GeV})$ is obtained from the estimate for $\hat{B}_{{K}}$ using the conversion factor 1.369

Fermionic lattice actions based on the Ginsparg–Wilson relation [398] are invariant under the chiral group, and hence four-quark operators such as $Q^{\Delta S=2}$ renormalize multiplicatively. However, depending on the particular formulation of Ginsparg–Wilson fermions, residual chiral symmetry-breaking effects may be present in actual calculations. For instance, in the case of domain-wall fermions, the finiteness of the extra 5th dimension implies that the decoupling of modes with different chirality is not exact, which produces a residual nonzero quark mass in the chiral limit. Whether or not a significant mixing with dimension-six operators is induced as well must be investigated on a case-by-case basis.

Recent lattice QCD calculations of $B_K$ have been performed with $N_{ f}=2+1+1$ dynamical quarks [42], and we want to mention a few conceptual issues that arise in this context. As described in Sect. 6.1, kaon mixing is expressed in terms of an effective four-quark interaction $Q^{{\Delta }S=2}$, considered below the charm threshold. When the matrix element of $Q^{{\Delta }S=2}$ is evaluated in a theory that contains a dynamical charm quark, the resulting estimate for $B_K$ must then be matched to the three-flavour theory which underlies the effective four-quark interaction.^31^ In general, the matching of $2+1$-flavour QCD with the theory containing $2+1+1$ flavours of sea quarks below the charm threshold can be accomplished by adjusting the coupling and quark masses of the $N_{ f}=2+1$ theory so that the two theories match at energies $E<m_c$. The corrections associated with this matching are of order $(E/m_c)^2$, since the subleading operators have dimension eight [399]. When the kaon mixing amplitude is considered, the matching also involves the relation between the relevant box graphs and the effective four-quark operator. In this case, corrections of order $(E/m_c)^2$ arise not only from the charm quarks in the sea, but also from the valence sector, since the charm quark propagates in the box diagrams. One expects that the sea-quark effects are subdominant, as they are suppressed by powers of $\alpha _s$. We note that the original derivation of the effective four-quark interaction is valid up to corrections of order $(E/m_c)^2$. While the kaon mixing amplitudes evaluated in the $N_{ f}=2+1$ and $2+1+1$ theories are thus subject to corrections of the same order in $E/m_c$ as the derivation of the conventional four-quark interaction, the general conceptual issue regarding the calculation of $B_K$ in QCD with $N_{ f}=2+1+1$ flavours should be addressed in detail in future calculations.

Another issue in this context is how the lattice scale and the physical values of the quark masses are determined in the $2+1$ and $2+1+1$ flavour theories. Here it is important to consider in which way the quantities used to fix the bare parameters are affected by a dynamical charm quark. Apart from a brief discussion in Ref. [42], these issues have not been fully worked out in the literature, but these kinds of mismatches were seen in simple lattice-QCD observables as quenched calculations gave way to $N_{ f}=2$ and then $2+1$ flavour results. Given the scale of the charm-quark mass relative to the scale of $B_K$, we expect these errors to be modest, but a more quantitative understanding is needed as statistical errors on $B_K$ are reduced. Within this review we will not discuss this issue further.

**Table 26:** Results for the Kaon *B* parameter in QCD with $N_{ f}=2$ dynamical flavours, together with a summary of systematic errors. Any available information as regards nonperturbative running is indicated in the column “running”, with details given at the bottom of the table

Collaboration	Refs.	$N_{ f}$	Publication status	Continuum extrapolation	Chiral extrapolation	Finite volume	Renormalization	Running	$B_{{K}}(\overline{\mathrm{MS}},2~\mathrm{GeV})$	$\hat{B}_{{K}}$
ETM 12D	[46]	2	A					$\,e$	0.531(16)(9)	0.727(22)(12)$^{{\ddagger }}$
ETM 10A	[401]	2	A					$\,f$	0.533(18)(12)$^{{\ddagger }}$	0.729(25)(17)
JLQCD 08	[409]	2	A					−	0.537(4)(40)	0.758(6)(71)
RBC 04	[400]	2	A			$^{{\dagger }}$		−	0.495(18)	0.678(25)$^{{\ddagger }}$
UKQCD 04	[410]	2	A			$^{{\dagger }}$		−	0.49(13)	0.68(18)

*e* $B_K$ is renormalized nonperturbatively at scales $1/a \sim 2{-}3.7~\mathrm{GeV}$ in the $N_{ f}= 2$ RI/MOM scheme. In this scheme, nonperturbative and NLO perturbative running are shown to agree from 4 GeV down to 2 GeV to better than 3% [142, 401]

*f* $B_K$ is renormalized nonperturbatively at scales $1/a \sim 2{-}3~\mathrm{GeV}$ in the $N_{ f}= 2$ RI/MOM scheme. In this scheme, nonperturbative and NLO perturbative running are shown to agree from 4 GeV down to 2 GeV to better than 3% [142, 401]

$^{{\dagger }}$ These results have been obtained at $(M_\pi L)_\mathrm{min} > 4$ in a lattice box with a spatial extension $L < 2$ fm

$^{{\ddagger }}$ $B_{K}({\overline{\text {MS}}}, 2~\mathrm{GeV})$ and $\hat{B}_{{K}}$ are related using the conversion factor 1.369 i.e. the one obtained with $N_f=2+1$

**Fig. 15:**
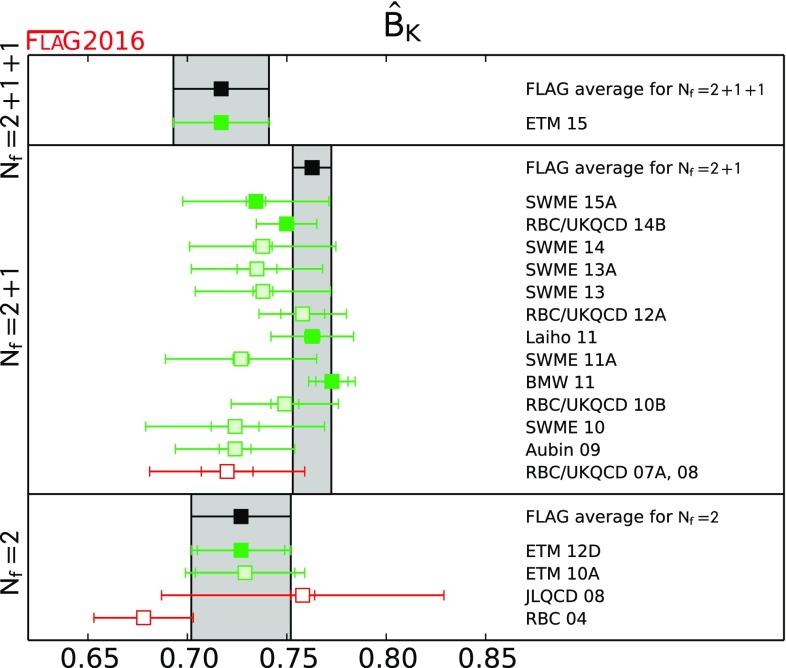
Recent unquenched lattice results for the RGI *B* parameter $\hat{B}_\mathrm{{K}}$. The *grey bands* indicate our global averages described in the text. For $N_{ f}=2+1+1$ and $N_{ f}=2$ the global estimate coincide with the results by ETM 12D and ETM 10A, respectively

Below we focus on recent results for $B_\mathrm{{K}}$, obtained for $N_{ f}=2, 2+1$ and $2+1+1$ flavours of dynamical quarks. A compilation of results is shown in Tables 25 and 26, as well as Fig. 15. An overview of the quality of systematic error studies is represented by the colour coded entries in Tables 25 and 26. In Appendix B.4 we gather the simulation details and results from different collaborations, the values of the most relevant lattice parameters, and comparative tables on the various estimates of systematic errors.

Some of the groups whose results are listed in Tables 25 and 26 do not quote results for both $B_\mathrm{{K}}(\overline{\mathrm{MS}},2\,\mathrm{GeV})$ – which we denote by the shorthand $B_\mathrm{{K}}$ from now on – and $\hat{B}_\mathrm{{K}}$. This concerns Refs. [46, 400, 401] for $N_{ f}=2$, Refs. [10, 31, 44, 45] for $2+1$ and Ref. [42] for $2+1+1$ flavours. In these cases we perform the conversion ourselves by evaluating the proportionality factor in Eq. (109) at $\mu =2\,\mathrm{GeV}$, using the following procedure: For $N_{ f}=2+1$ we use the value $\alpha _s(M_\mathrm{{Z}})=0.1185$ from the 2014 edition of the PDG [151] and run it across the quark thresholds at $m_b=4.18$ GeV and $m_c=1.275$ GeV, and then run up in the three-flavour theory to $\mu =2\,\mathrm{GeV}$. All running is done using the four-loop RG $\beta$-function. The resulting value of $\alpha _s^{{\overline{\text {MS}}}}(2\,\mathrm{GeV})=0.29672$ is then used to evaluate $\hat{B}_\mathrm{{K}}/B_\mathrm{{K}}$ in perturbation theory at NLO, which gives $\hat{B}_\mathrm{{K}}/B_\mathrm{{K}}=1.369$ in the three-flavour theory. This value of the conversion factor has also been applied to the result computed in QCD with $N_{ f}=2+1+1$ flavours of dynamical quarks [42].

In two-flavour QCD one can insert the updated nonperturbative estimate for the $\Lambda$ parameter by the ALPHA Collaboration [12], i.e. $\Lambda ^{(2)}=310(20)$ MeV, into the NLO expressions for $\alpha _s$. The resulting value of the perturbative conversion factor $\hat{B}_K/B_K$ for $N_{ f}=2$ is then equal to 1.386. However, since the running coupling in the ${\overline{\text {MS}}}$ scheme enters at several stages in the entire matching and running procedure, it is difficult to use this estimate of $\alpha _s$ consistently without a partial reanalysis of the data in Refs. [46, 400, 401]. We have therefore chosen to apply the conversion factor of 1.369 not only to results obtained for $N_{ f}=2+1$ flavours but also to the two-flavour theory (in the cases where only one of $\hat{B_K}$ and $B_K$ are quoted). We note that the difference between 1.386 and 1.369 will produce an ambiguity of the order of 1%, which is well below the overall uncertainties in Refs. [400, 401]. We have indicated explicitly in Table 26 in which way the conversion factor 1.369 has been applied to the results of Refs. [46, 400, 401].

Since the last edition of the FLAG review [2] several new or updated results have been reported. For QCD with $N_f=2+1+1$ there is now a published calculation from the ETM Collaboration [42]; updated results for $N_f=2+1$ have been reported by several collaborations, i.e. RBC/UKQCD 14B [10], SWME 13A [402], SWME 14 [385] and SWME 15A [45]. For $N_f=2$ we now include the result from ETMC, i.e. ETM 12D [46]. We briefly discuss the main features of the most recent calculations below.

The calculation by ETM 15 [42] employs Osterwalder–Seiler valence quarks on twisted-mass dynamical quark ensembles. Both valence and sea quarks are tuned to maximal twist. This mixed action setup guarantees that the four-fermion matrix elements are automatically $\mathcal {O}(a)$ improved and free of wrong chirality mixing effects. The calculation has been carried out at three values of the lattice spacing ($a \simeq 0.06{-}0.09$ fm). Light pseudoscalar mass values are in the range $210{-}450$ MeV. The spatial lattice sizes vary between 2.1 to 2.9 fm and correspond to $M_{\pi , \mathrm{min}} L \simeq 3.2{-}3.5$. Finite-volume effects are investigated at the coarsest lattice spacing by controlling the consistency of results obtained at two lattice volumes at 280 MeV for the light pseudoscalar mass. The determination of the bag parameter is performed using simultaneous chiral and continuum fits. The renormalization factors have been evaluated using the RI/MOM technique for $N_f=4$ degenerate Wilson twisted-mass dynamical quark gauge configurations generated for this purpose. In order to gain control over discretization effects the evaluation of the renormalization factors has been carried out following two different methods. The uncertainty from the RI computation is estimated at 2%. The conversion to $\overline{\mathrm{MS}}$ produces an additional 0.6% of systematic error. The overall uncertainty for the bag parameter is computed from a distribution of several results, each one of them corresponding to a variant of the analysis procedure.

The collection of results from the SWME Collaboration [45, 278, 385, 402–404] have all been obtained using a mixed action, i.e. HYP-smeared valence staggered quarks on the Asqtad improved, rooted staggered MILC ensembles. For the latest set of results, labelled SWME 14, 15A [45, 385] an extended set of ensembles, comprising finer lattice spacings and a smallest pion mass of 174 MeV has been added to the calculation. The final estimate for $B_K$ is obtained from a combined chiral and continuum extrapolation using the data computed for the three finest lattice spacings. The dominant systematic error of 4.4% is associated with the matching factor between the lattice and ${\overline{\text {MS}}}$ schemes. It has been computed in perturbation theory at one loop, and its error was estimated assuming a missing two-loop matching term of size $1\times \alpha (1/a)^2$, i.e. with no factors of $1/(4\pi )$ included. Different functional forms for the chiral fits contribute another 2% to the error budget. It should also be noted that Bayesian priors are used to constrain some of the coefficients in the chiral ansatz. The total systematic error amounts to about 5%. Compared to the earlier calculations of SWME one finds that “the overall error is only slightly reduced, but, more importantly, the methods of estimating errors have been improved” [385].

The RBC and UKQCD Collaborations have updated their value for $B_K$ using $N_{ f}=2+1$ flavours of domain-wall fermions [10]. Previous results came from ensembles at three different lattice spacings with unitary pion masses in the range of 170 to 430 MeV. The new work adds an ensemble with essentially physical light and strange quark masses at two of the lattice spacings, along with a third finer lattice with 370 MeV pion masses. This finer ensemble provides an additional constraint on continuum extrapolations. Lattice spacings and quark masses are determined via a combined continuum and chiral extrapolation to all ensembles. With lattice spacings at hand, nonperturbative renormalization and nonperturbative step scaling are used to find the renormalized value of $B_K$ at 3 GeV in the RI-SMOM($\gamma ^\mu ,\gamma ^\mu )$ and RI-SMOM() schemes for all of the ensembles. These $B_K$ values for each pion mass are determined for the physical strange quark mass through valence strange quark interpolations/extrapolations and dynamical strange quark mass reweighting. The light-quark mass dependence is then fit to *SU*(2) chiral perturbation theory. Because the new ensembles have quark masses within a few percent of their physical values, the systematic error related to the extrapolation to physical values is neglected. The new physical point ensembles have (5.5 fm)$^3$ volumes, and chiral perturbation theory fits with and without finite-volume corrections differ by 10–20% of the statistical errors, so no finite-volume error is quoted. The fits are dominated by the physical point ensembles, which have small errors. Fits with $B_K$ normalized in both RI-SMOM schemes are done, and the difference is used to estimate the systematic error due to nonperturbative renormalization.

The $N_{ f}=2$ calculation described in ETM 12D [46] uses a mixed action setup employing twisted-mass dynamical quarks and Osterwalder–Seiler quarks in the valence, both tuned to maximal twist. The work of ETM 12D is an update of the calculation of ETM 10A [401]. The main addition is the inclusion of a fourth (superfine) lattice spacing ($a \simeq 0.05$ fm). Thus, the computation is performed at four values of the lattice spacing ($a \simeq 0.05{-}0.1$ fm), and the lightest simulated value of the light pseudoscalar mass is about 280 MeV. Final results are obtained with combined chiral and continuum fits. Finite-volume effects are studied at one value of the lattice spacing ($a \simeq 0.08$ fm), and it is found that results obtained on two lattice volumes, namely for $L=2.2$ and 2.9 fm at $M_\pi \approx 300$ MeV are in good agreement within errors. The four- and two-fermion renormalization factors needed in the bag parameter evaluation are computed nonperturbatively using the Rome–Southampton method. The systematic error due to the matching of RI and $\overline{\mathrm{MS}}$ schemes is estimated to be 2.5%.

We now describe our procedure for obtaining global averages. The rules of Sect. 2.1 stipulate that results free of red tags and published in a refereed journal may enter an average. Papers that at the time of writing are still unpublished but are obvious updates of earlier published results can also be taken into account.

There is only one result for $N_f=2+1+1$, computed by the ETM Collaboration [42]. Since it is free of red tags, it qualifies as the currently best global estimate, i.e.

117 $$\begin{aligned}&N_{ f}=2+1+1:\quad \hat{B}_\mathrm{{K}} = 0.717(18)(16),\nonumber \\&B_\mathrm{{K}}^{\overline{\text {MS}}}(2\,\mathrm{GeV}) = 0.524(13)(12)\quad \,\mathrm {Ref.}~[42]. \end{aligned}$$

The bulk of results for the kaon *B* parameter has been obtained for $N_{ f}=2+1$. As in the previous edition of the FLAG review [2] we include the results from SWME [45, 385, 402], despite the fact that nonperturbative information on the renormalization factors is not available. Instead, the matching factor has been determined in perturbation theory at one loop, but with a sufficiently conservative error of 4.4%.

Thus, for $N_{ f}=2+1$ our global average is based on the results of BMW 11 [43], Laiho 11 [44], RBC/UKQCD 14B [10] and SWME 15A [45]. The last three are the latest updates from a series of calculations by the same collaborations. Our procedure is as follows: in a first step statistical and systematic errors of each individual result for the RGI *B* parameter, $\hat{B}_\mathrm{{K}}$, are combined in quadrature. Next, a weighted average is computed from the set of results. For the final error estimate we take correlations between different collaborations into account. To this end we note that we consider the statistical and finite-volume errors of SWME 15A and Laiho 11 to be correlated, since both groups use the Asqtad ensembles generated by the MILC Collaboration. Laiho 11 and RBC/UKQCD 14B both use domain-wall quarks in the valence sector and also employ similar procedures for the nonperturbative determination of matching factors. Hence, we treat the quoted renormalization and matching uncertainties by the two groups as correlated. After constructing the global covariance matrix according to Schmelling [91], we arrive at

118 $$\begin{aligned} N_{ f}=2+1:\quad \hat{B}_\mathrm{{K}} = 0.7625(97)\quad \,\mathrm {Refs.}~[10, 43{-}45],\nonumber \\ \end{aligned}$$

with $\chi ^2/\mathrm{d.o.f.}=0.675$. After applying the NLO conversion factor $\hat{B}_\mathrm{{K}}/B_\mathrm{{K}}^{\overline{\text {MS}}}(2\,\mathrm{GeV})=1.369$, this translates into

119 $$\begin{aligned} N_{ f}=2+1:\quad B_\mathrm{{K}}^{\overline{\text {MS}}}(2\,\mathrm{GeV})=0.5570(71)\,\mathrm {Refs.}~[10, 43{-}45].\nonumber \\ \end{aligned}$$

These values and their uncertainties are very close to the global estimates quoted in the previous edition of the FLAG review [2]. Note, however, that the statistical errors of each calculation entering the global average have now been reduced to a level that makes them statistically incompatible. It is only because of the relatively large systematic errors that the weighted average produces a value of $\mathcal {O}(1)$ for the reduced $\chi ^2$.

Passing over to describing the results computed for $N_{ f}=2$ flavours, we note that there is only the set of results published in ETM 12D [46] and ETM 10A [401] that allow for an extensive investigation of systematic uncertainties. We identify the result from ETM 12D [46], which is an update of ETM 10A, with the currently best global estimate for two-flavour QCD, i.e.

120 $$\begin{aligned}&N_{ f}=2:\quad \hat{B}_\mathrm{{K}} = 0.727(22)(12),\nonumber \\&\quad B_\mathrm{{K}}^{\overline{\text {MS}}}(2\,\mathrm{GeV}) = 0.531(16)(9)\quad \,\mathrm {Ref.}~[46].\nonumber \\ \end{aligned}$$

The result in the ${\overline{\text {MS}}}$ scheme has been obtained by applying the same conversion factor of 1.369 as in the three-flavour theory.

### Kaon BSM *B* parameters

We now report on lattice results concerning the matrix elements of operators that encode the effects of physics beyond the Standard Model (BSM) to the mixing of neutral kaons. In this theoretical framework both the SM and the BSM contributions add up to reproduce the experimentally observed value of $\epsilon _K$. Since BSM contributions involve heavy but unobserved particles, it is natural to assume that they are short-distance dominated. The effective Hamiltonian for generic ${\Delta }S=2$ processes including BSM contributions reads

121 $$\begin{aligned} \mathcal{H}_\mathrm{eff,BSM}^{\Delta S=2} = \sum _{i=1}^5 C_i(\mu )Q_i(\mu ), \end{aligned}$$

where $Q_1$ is the four-quark operator of Eq. (101) that gives rise to the SM contribution to $\epsilon _K$. In the so-called SUSY basis introduced by Gabbiani et al. [411] the (parity-even) operators $Q_2,\ldots ,Q_5$ read^32^

122 $$\begin{aligned}&Q_2 = \big (\bar{s}^a(1-\gamma _5)d^a\big ) \big (\bar{s}^b(1-\gamma _5)d^b\big ), \nonumber \\&Q_3 = \big (\bar{s}^a(1-\gamma _5)d^b\big ) \big (\bar{s}^b(1-\gamma _5)d^a\big ), \nonumber \\&Q_4 = \big (\bar{s}^a(1-\gamma _5)d^a\big ) \big (\bar{s}^b(1+\gamma _5)d^b\big ), \nonumber \\&Q_5 = \big (\bar{s}^a(1-\gamma _5)d^b\big ) \big (\bar{s}^b(1+\gamma _5)d^a\big ), \end{aligned}$$

where *a* and *b* denote colour indices. In analogy to the case of $B_\mathrm{{K}}$ one then defines the *B* parameters of $Q_2,\ldots ,Q_5$ according to

123 $$\begin{aligned} B_i(\mu ) = \frac{\left\langle \bar{K}^0\left| Q_i(\mu )\right| K^0 \right\rangle }{N_i\left\langle \bar{K}^0\left| \bar{s}\gamma _5 d\right| 0\right\rangle \left\langle 0\left| \bar{s}\gamma _5 d\right| K^0\right\rangle }, \quad i=2,\ldots ,5.\nonumber \\ \end{aligned}$$

The factors $\{N_2,\ldots ,N_5\}$ are given by $\{-5/3, 1/3, 2, 2/3\}$, and it is understood that $B_i(\mu )$ is specified in some renormalization scheme, such as ${\overline{\text {MS}}}$ or a variant of the regularization-independent momentum subtraction (RI-MOM) scheme.

The SUSY basis has been adopted in Refs. [42, 46, 412]. Alternatively, one can employ the chiral basis of Buras, Misiak and Urban [413]. The SWME Collaboration prefers the latter, since the anomalous dimension which enters the RG running has been calculated to two loops in perturbation theory [413]. Results obtained in the chiral basis can easily be converted to the SUSY basis via

124 $$\begin{aligned} B_3^\mathrm{SUSY}={\textstyle \frac{1}{2}}\left( 5B_2^\mathrm{chiral} - 3B_3^\mathrm{chiral} \right) . \end{aligned}$$

The remaining *B* parameters are the same in both bases. In the following we adopt the SUSY basis and drop the superscript.

Older quenched results for the BSM *B* parameters can be found in Refs. [414–416]. Recent estimates for $B_2,\ldots ,B_5$ have been reported for QCD with $N_{ f}=2$ (ETM 12D [46]), $N_{ f}=2+1$ (RBC/UKQCD 12E [412], SWME 13A [402], SWME 14C [417], SWME 15A [45]) and $N_{ f}=2+1+1$ (ETM 15 [42]) flavours of dynamical quarks. The main features of these calculations are identical to the case of $B_\mathrm{{K}}$ discussed above. We note, in particular, that SWME perform the matching between rooted staggered quarks and the ${\overline{\text {MS}}}$ scheme using perturbation theory at one loop, while RBC/UKQCD and ETMC employ nonperturbative renormalization for domain-wall and twisted-mass Wilson quarks, respectively. Control over systematic uncertainties (chiral and continuum extrapolations, finite-volume effects) in $B_2,\ldots ,B_5$ is expected to be at the same level as for $B_\mathrm{{K}}$, as far as the results by ETM 12D, ETM 15 and SWME 15A are concerned. The calculation by RBC/UKQCD 12E has been performed at a single value of the lattice spacing and a minimum pion mass of 290 MeV. Thus, the results do not benefit from the same improvements regarding control over the chiral and continuum extrapolations as in the case of $B_\mathrm{{K}}$ [10]. Preliminary results from RBC/UKQCD using two values of the lattice spacing have been reported in Refs. [418] and [419].

**Fig. 16:**
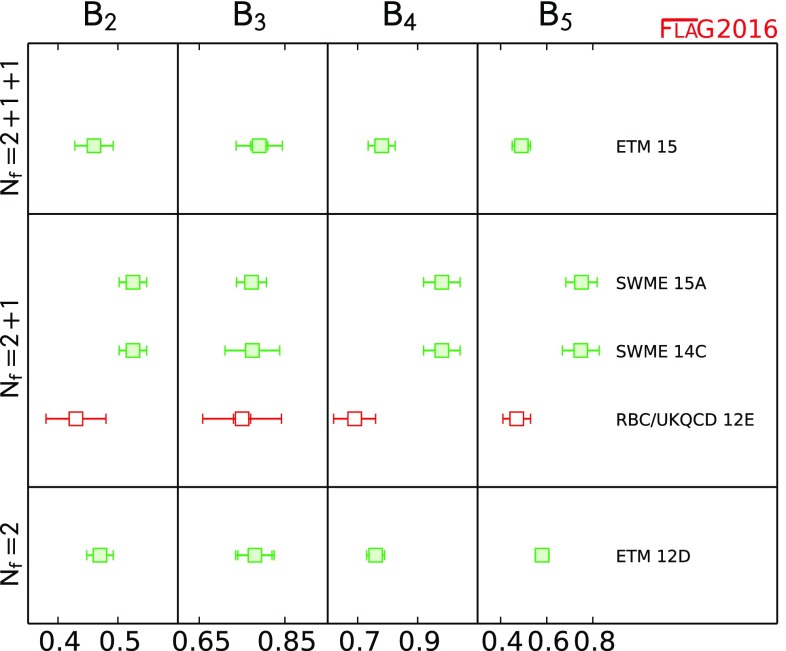
Lattice results for the BSM *B* parameters defined in the ${\overline{\text {MS}}}$ scheme at a reference scale of 3 GeV; see Table 27

Results for the *B* parameters $B_2,\ldots ,B_5$ computed with $N_{ f}=2, 2+1$ and $2+1+1$ dynamical quarks are listed and compared in Table 27 and Fig. 16. In general one finds that the BSM *B* parameters computed by different collaborations do not show the same level of consistency as the SM kaon mixing parameter $B_K$ discussed previously. In particular, the results for $B_2, B_4$ and $B_5$ from SWME [45, 402, 417], obtained using staggered quarks and employing perturbative matching differ significantly from those quoted by the ETM [42, 46] and RBC/UKQCD [412] Collaborations, which both determine the matching factors nonperturbatively. The preliminary results from the recent update of the RBC/UKQCD calculation described in Ref. [419] provides a hint that the nonperturbative determination of the matching factors depends strongly on the details in the implementation of the Rome–Southampton method. The use of nonexceptional momentum configurations in the calculation of the vertex functions produces a significant modification of the renormalization factors, which in turn brings the results from RBC/UKQCD – in particular the estimates for $B_4$ and $B_5$ – much closer to the estimates from SWME.

**Table 27:** Results for the BSM *B* parameters $B_2,\ldots ,B_5$ in the ${\overline{\text {MS}}}$ scheme at a reference scale of 3 GeV. Any available information on nonperturbative running is indicated in the column “running”, with details given at the bottom of the table

Collaboration	Refs.	$N_{ f}$	Publication status	Continuum extrapolation	Chiral extrapolation	Finite volume	Renormalization	Running	$B_2$	$B_3$	$B_4$	$B_5$
ETM 15	[42]	$2+1+1$	A					$\,a$	0.46(1)(3)	0.79(2)(5)	0.78(2)(4)	0.49(3)(3)
SWME 15A	[45]	$2+1$	A				$^{\dagger }$	−	0.525(1)(23)	0.773(6)(35)	0.981(3)(62)	0.751(7)(68)
SWME 14C	[417]	$2+1$	C				$^{\dagger }$	−	0.525(1)(23)	0.774(6)(64)	0.981(3)(61)	0.748(9)(79)
SWME 13A$^{\ddagger }$	[402]	$2+1$	A				$^{\dagger }$	−	0.549(3)(28)	0.790(30)	1.033(6)(46)	0.855(6)(43)
RBC/UKQCD 12E	[412]	$2+1$	A					$\,b$	0.43(1)(5)	0.75(2)(9)	0.69(1)(7)	0.47(1)(6)
ETM 12D	[46]	2	A					$\,c$	0.47(2)(1)	0.78(4)(2)	0.76(2)(2)	0.58(2)(2)

*a* $B_i$ are renormalized nonperturbatively at scales $1/a \sim 2.2{-}3.3\,\mathrm{GeV}$ in the $N_{ f}= 4$ RI/MOM scheme using two different lattice momentum scale intervals, with values around 1 / *a* for the first and around 3.5 GeV for the second one. The impact of these two ways to the final result is taken into account in the error budget. Conversion to ${\overline{\text {MS}}}$ is at one loop at 3 GeV

*b* The *B* parameters are renormalized nonperturbatively at a scale of 3 GeV

*c* $B_i$ are renormalized nonperturbatively at scales $1/a \sim 2{-}3.7\,\mathrm{GeV}$ in the $N_{ f}= 2$ RI/MOM scheme using two different lattice momentum scale intervals, with values around 1 / *a* for the first and around 3 GeV for the second one

$^{{\dagger }}$ The renormalization is performed using perturbation theory at one loop, with a conservative estimate of the uncertainty

$^{{\ddagger }}$ The computation of $B_4$ and $B_5$ has been revised in Refs. [45] and [417]

Therefore, insufficient control over the renormalization and matching procedure appears to be the most likely explanation for the observed deviations. In the absence of further investigations that corroborate this conjecture, it is difficult to quote global estimates for the BSM *B* parameters $B_2,\ldots ,B_5$. However, we observe that for each choice of $N_{ f}$ there is only one set of results that meets the required quality criteria, i.e. ETM 15 [42] for $N_{ f}=2+1+1$, SWME 15A [45] for $N_{ f}=2+1$, and ETM 12D [46] for two-flavour QCD.

## *D*-meson-decay constants and form factors

Leptonic and semileptonic decays of charmed *D* and $D_s$ mesons occur via charged *W*-boson exchange, and they are sensitive probes of $c \rightarrow d$ and $c \rightarrow s$ quark flavour-changing transitions. Given experimental measurements of the branching fractions combined with sufficiently precise theoretical calculations of the hadronic matrix elements, they enable the determination of the CKM matrix elements $|V_{cd}|$ and $|V_{cs}|$ (within the Standard Model) and a precise test of the unitarity of the second row of the CKM matrix. Here we summarize the status of lattice-QCD calculations of the charmed leptonic decay constants. Significant progress has been made in charm physics on the lattice in recent years, largely due to the availability of gauge configurations produced using highly improved lattice-fermion actions that enable treating the *c*-quark with the same action as for the *u*, *d*, and *s*-quarks.

This section updates the corresponding one in the last FLAG review [2] for results that appeared after November 30, 2013. As already done in Ref. [2], we limit our review to results based on modern simulations with reasonably light pion masses (below approximately 500 MeV). This excludes results obtained from the earliest unquenched simulations, which typically had two flavours in the sea, and which were limited to heavier pion masses because of the constraints imposed by the computational resources and methods available at that time. Recent lattice-QCD averages for $D_{(s)}$-meson-decay constants were also presented by the Particle Data Group in the review on “Leptonic Decays of Charged Pseudoscalar Mesons” [184]. The PDG three- and four-flavour averages for $f_D$, $f_{D_s}$, and their ratio are identical to those obtained here. This is because both reviews include the same sets of calculations in the averages, and make the same assumptions about the correlations between the calculations.

Following our review of lattice-QCD calculations of $D_{(s)}$-meson leptonic decay constants and semileptonic form factors, we then interpret our results within the context of the Standard Model. We combine our best-determined values of the hadronic matrix elements with the most recent experimentally measured branching fractions to obtain $|V_{cd(s)}|$ and test the unitarity of the second row of the CKM matrix.

### Leptonic decay constants $f_D$ and $f_{D_s}$

In the Standard Model the decay constant $f_{D_{(s)}}$ of a charged pseudoscalar *D* or $D_s$ meson is related to the branching ratio for leptonic decays mediated by a *W* boson through the formula

125 $$\begin{aligned} {\mathcal {B}}(D_{(s)} \rightarrow \ell \nu _\ell )= & {} {{G_F^2|V_{cq}|^2 \tau _{D_{(s)}}}\over {8 \pi }} f_{D_{(s)}}^2 m_\ell ^2 m_{D_{(s)}}\nonumber \\&\times \left( 1-{{m_\ell ^2}\over {m_{D_{(s)}}^2}}\right) ^2, \end{aligned}$$

where $V_{cd}$ ($V_{cs}$) is the appropriate CKM matrix element for a *D* ($D_s$) meson. The branching fractions have been experimentally measured by CLEO, Belle, Babar and BES with a precision around 4–5$\%$ for both the *D* and the $D_s$-meson decay modes [184]. When combined with lattice results for the decay constants, they allow for determinations of $|V_{cs}|$ and $|V_{cd}|$.

**Fig. 17:**
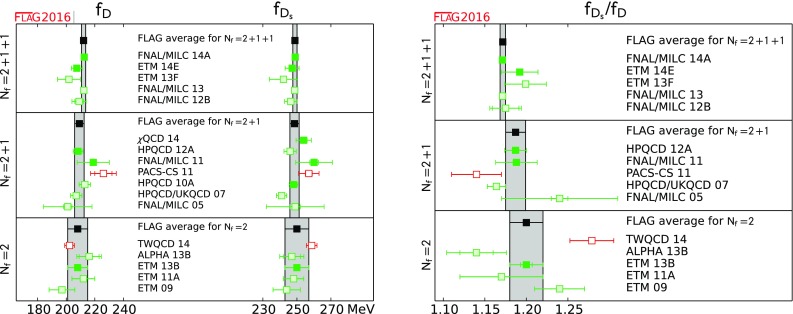
Decay constants of the *D* and $D_s$ mesons [values in Table 28] and Eqs. 127–129]. The *significance of the colours* is explained in Sect. 2. The *black squares* and *grey bands* indicate our averages

In lattice-QCD calculations the decay constants $f_{D_{(s)}}$ are extracted from Euclidean matrix elements of the axial current

126 $$\begin{aligned} \langle 0| A^{\mu }_{cq} | D_q(p) \rangle = i f_{D_q}\;p_{D_q}^\mu , \end{aligned}$$

with $q=d,s$ and $A^{\mu }_{cq} =\bar{c}\gamma _\mu \gamma _5 q$. Results for $N_f=2,\; 2+1$ and $2+1+1$ dynamical flavours are summarized in Table 28 and Fig. 17. Since the publication of the last FLAG review, a handful of results for $f_D$ and $f_{D_s}$ have appeared, which we are going to briefly describe here. We consider isospin-averaged quantities, although in a few cases results for $f_{D^+}$ are quoted (FNAL/MILC 11 and FNAL/MILC 14A, where the difference between $f_D$ and $f_{D^+}$ has been estimated to be at the 0.5 MeV level).

**Table 28:** Decay constants of the *D* and $D_{s}$ mesons (in MeV) and their ratio

Collaboration	Refs.	$N_{ f}$	Publication status	Continuum extrapolation	Chiral extrapolation	Finite volume	Renormalization/ matching	Heavy-quark treatment	$f_D$	$f_{D_s}$	$f_{D_s}/f_D$
FNAL/MILC 14A$^{\mathrm{f}}$	[14]	$2+1+1$	A						212.6(0.4) $+1.0 \atopwithdelims ()-1.2$	249.0(0.3)$+1.1 \atopwithdelims ()-1.5$	1.1712(10)$+29 \atopwithdelims ()-32$
ETM 14E$^{\mathrm{a}}$	[27]	$2+1+1$	A						207.4(3.8)	247.2(4.1)	1.192(22)
ETM 13F	[230]	$2+1+1$	C						202(8)	242(8)	1.199(25)
FNAL/MILC 13$^{\mathrm{b}}$	[420]	$2+1+1$	C						212.3(0.3)(1.0)	248.7(0.2)(1.0)	1.1714(10)(25)
FNAL/MILC 12B	[421]	$2+1+1$	C						209.2(3.0)(3.6)	246.4(0.5)(3.6)	1.175(16)(11)
$\chi$QCD 14	[17]	$2+1$	A							254(2)(4)	
HPQCD 12A	[47]	$2+1$	A						208.3(1.0)(3.3)	246.0(0.7)(3.5)	1.187(4)(12)
FNAL/MILC 11	[48]	$2+1$	A						218.9(11.3)	260.1(10.8)	1.188(25)
PACS-CS 11	[422]	$2+1$	A						226(6)(1)(5)	257(2)(1)(5)	1.14(3)
HPQCD 10A	[49]	$2+1$	A						213(4)$^{\mathrm{c}}$	248.0(2.5)	
HPQCD/UKQCD 07	[28]	$2+1$	A						207(4)	241 (3)	1.164(11)
FNAL/MILC 05	[423]	$2+1$	A						201(3)(17)	249(3)(16)	1.24(1)(7)
TWQCD 14$^{\mathrm{e}}$	[424]	2	A						202.3(2.2)(2.6)	258.7(1.1)(2.9)	1.2788(264)
ALPHA 13B	[177]	2	C						216(7)(5)	247(5)(5)	1.14(2)(3)
ETM 13B$^{\mathrm{d}}$	[20]	2	A						208(7)	250(7)	1.20(2)
ETM 11A	[182]	2	A						212(8)	248(6)	1.17(5)
ETM 09	[32]	2	A						197(9)	244(8)	1.24(3)

$^{\mathrm{a}}$ Update of ETM 13F

$^{\mathrm{b}}$ Update of FNAL/MILC 12B

$^{\mathrm{c}}$ This result is obtained by using the central value for $f_{D_s}/f_D$ from HPQCD/UKQCD 07 and increasing the error to account for the effects from the change in the physical value of $r_1$

$^{\mathrm{d}}$ Update of ETM 11A and ETM 09

$^{\mathrm{e}}$ One lattice spacing ${\simeq } 0.1$ fm only. $M_{\pi ,\mathrm{min}}L=1.93$

$^{\mathrm{f}}$ At $\beta = 5.8$, $M_{\pi , \mathrm{min}}L=3.2$ but this ensemble is primarily used for the systematic error estimate

Two new results have appeared for $N_f=2$. The averages, however, remain unchanged, as we will see in the following. In Ref. [177], the ALPHA Collaboration directly computed the matrix element in Eq. (126) (for $\mu =0$ and $q=d,s$) on two $N_f=2$ ensembles of nonperturbatively $\mathcal {O}(a)$ improved Wilson fermions at lattice spacings of 0.065 and 0.048 fm. Pion masses range between 440 and 190 MeV and the condition $Lm_\pi \ge 4$ is always met. Chiral/continuum extrapolations are performed adopting either a fit ansatz linear in $m_\pi ^2$ and $a^2$ or, for $f_D$, by using a fit form inspired by partially quenched Heavy Meson Chiral Perturbation Theory (HM$\chi$PT). Together with the scale setting, these extrapolations dominate the final systematic errors. As the scale is set through another decay constant ($f_K$), what is actually computed is $f_{D_{(s)}}/f_K$ and most of the uncertainty on the renormalization constant of the axial current drops out. Since the results only appeared as a proceeding contribution to the Lattice 2013 conference, they do not enter the final averages.

The TWQCD Collaboration reported in Ref. [424] about the first computation of the masses and decay constants of pseudoscalar $D_{(s)}$ mesons in two-flavour lattice QCD with domain-wall fermions. This is a calculation performed at one lattice spacing only ($a\approx 0.061$fm) and in a rather small volume ($24^3 \times 48$, with $M_{\pi ,\mathrm{min}}L \approx 1.9$). For these reasons the quoted values of the decay constants do not qualify for the averages and should be regarded as the result of a pilot study in view of a longer and on-going effort, in which the remaining systematics will be addressed through computations at different volumes as well as several lattice spacings.

The $N_f=2$ averages therefore coincide with those in the previous FLAG review and are given by the values in ETM 13B, namely127The situation is quite similar for the $N_f=2+1$ case, where only one new result, and for $f_{D_s}$ only, appeared in the last 2 years. The $\chi$QCD Collaboration used (valence) overlap fermions on a sea of $2+1$ flavours of domain-wall fermions (corresponding to the gauge configurations generated by RBC/UKQCD and described in Ref. [144]) to compute the charm- and the strange-quark masses as well as $f_{D_s}$. The decay constant is obtained by combining the determinations from either an exactly conserved PCAC Ward identity or from the matrix element of the local axial current. The latter needs to be renormalized and the corresponding renormalization constant has been determined nonperturbatively in Ref. [425]. The computation of $f_{D_s}$ has been performed at two lattice spacings ($a=0.113$ and $a=0.085$ fm) with the value of the bare charm-quark mass, in lattice units, ranging between 0.3 and 0.75. Pion masses reach down to about 300 MeV and $M_{\pi ,\mathrm{min}}L$ is always larger than 4. The chiral extrapolation and lattice artefacts are responsible for the largest systematic uncertainties, both being estimated to be around 1%, on top of a statistical error of about the same size. The lattice spacing dependence is estimated by changing the functional form in the chiral/continuum extrapolation by terms of $\mathcal {O}(a^4)$. As the authors point out, it will be possible to make a more accurate assessment of the discretization errors only once the planned ensembles at a finer lattice spacing are available.

The RBC/UKQCD Collaboration presented intermediate results for the *D* and $D_s$ decay constants with $2+1$ flavours of Möbius domain-wall fermions in Ref. [426]. Since the analysis has not been completed yet, no values for $f_{D_{(s)}}$ are quoted.

Summarizing the $N_{ f}=2+1$ case, the average for $f_D$ did not change with respect to the last review and it is obtained from the HPQCD 12A and the FNAL/MILC 11 determinations, whereas for $f_{D_s}$ the value changes in order to include the result from the $\chi$QCD Collaboration (together with the values in HPQCD 10A and in FNAL/MILC 11). The updated estimates then read128where the error on the $N_{ f}=2+1$ average of $f_{D_s}$ has been rescaled by the factor $\sqrt{\chi ^2/\hbox {d.o.f.}}=1.1$ (see Sect. 2). In addition, the statistical errors between the results of FNAL/MILC and HPQCD have been everywhere treated as 100% correlated since the two collaborations use overlapping sets of configurations. The same procedure had been used in the 2013 review.

Two new determinations appeared from simulations with $2+1+1$ dynamical flavours. These are FNAL/MILC 14A and ETM 14E. The FNAL/MILC 14A results in Ref. [14] are obtained using the HISQ ensembles with up, down, strange and charm dynamical quarks, generated by the MILC Collaboration [334] (see also Ref. [209] for the RMS pion masses) employing HISQ sea quarks and a one-loop tadpole improved Symanzik gauge action. The RHMC as well as the RHMD algorithms have been used in this case. The latter is an inexact algorithm, where the accept/reject step at the end of the molecular-dynamics trajectory is skipped. In Ref. [334] results for the plaquette, the bare fermion condensates and a few meson masses, using both algorithms, are compared and found to agree within statistical uncertainties. The relative scale is set through $F_{4ps}$, the decay constant of a fictitious meson with valence masses of $0.4 m_s$ and physical sea-quark masses. For the absolute scale $f_\pi$ is used. In FNAL/MILC 14A four different lattice spacings, ranging from 0.15 to 0.06 fm, have been considered with all quark masses close to their physical values. The analysis includes additional ensembles with light sea-quark masses that are heavier than in nature, and where in some cases the strange sea-quark masses are lighter than in nature. This allowed one to actually perform two different analyses; the “physical mass analysis” and the “chiral analysis”. The second analysis uses staggered chiral perturbation theory for all-staggered heavy–light mesons in order to include the unphysical-mass ensembles. This results in smaller statistical errors compared to the “physical mass analysis”. The latter is used for the central values and the former as a cross-check and as an ingredient in the systematic error analysis. Chiral and continuum extrapolation uncertainties are estimated by considering a total of 114 different fits. The quark-mass and lattice-spacing dependence of the decay constants are modelled in heavy-meson, rooted, all-staggered chiral perturbation theory (HMrAS$\chi$PT) including all NNLO and N$^3$LO mass-dependent, analytic, terms. Fits differ in the way some of the LEC’s are fixed, in the number of NNLO parameters related to discretization effects included, in the use of priors, in whether the $a=0.15$ fm ensembles are included or not and in the inputs used for the quark masses and the lattice spacings. The number of parameters ranges between 23 and 28 and the number of data points varies between 314 and 366. The maximum difference between these results and the central values is taken as an estimate of the chiral/continuum extrapolation errors. The central fit is chosen to give results that are close to the centres of the distributions, in order to symmetrize the errors. FNAL/MILC also provides in Ref. [14] an estimate of strong isospin-breaking effects by computing the *D*-meson-decay constant with the mass of the light quark in the valence set to the physical value of the down-quark mass. The result reads $f_{D^+} - f_D=0.47(1){+25\atopwithdelims ()-6}$ MeV. This effect is of the size of the quoted errors, and the number in Table 28 indeed corresponds to $f_{D^+}$. The final accuracy on the decay constants is at the level of half-a-percent. It is therefore necessary to consider the electroweak corrections to the decay rates when extracting $|V_{cd}|$ and $|V_{cs}|$ from leptonic transitions of $D_{(s)}$ mesons. The most difficult to quantify is due to electromagnetic effects that depend on the meson hadronic structure. In Ref. [14] this contribution to the decay rates is estimated to be between 1.1% and 2.8%, by considering the corresponding contribution for $\pi$ and *K* decays, as computed in $\chi$PT, and allowing for a factor 2 to 5. After correcting the PDG data for the decay rates in Ref. [151], by including the effects mentioned above with their corresponding uncertainty, the FNAL/MILC Collaboration uses the results for $f_D$ and $f_{D_s}$ to produce estimates for $|V_{cd}|$ and $|V_{cs}|$, as well as a unitarity test of the second row of the CKM matrix, which yields $1- |V_{cd}|^2 - |V_{cs}|^2 - |V_{cb}|^2 =-0.07(4)$, indicating a slight tension with CKM unitarity.^33^

The ETM Collaboration has also published results with $2+1+1$ dynamical flavours in Ref. [27] (ETM 14E), updating the values that appeared in the Lattice 2013 Conference proceedings [230] (ETM 13F). The configurations have been generated using the Iwasaki action in the gauge and the Wilson twisted-mass action for sea quarks. The charm and strange valence quarks are discretized as Osterwalder–Seiler fermions [427]. Three different lattice spacings in the range 0.09–0.06 fm have been considered with pion masses as low as 210 MeV in lattices of linear spatial extent of about 2 to 3 fm (see Ref. [4] for details of the simulations). In ETM 14E $f_{D_s}$ is obtained by extrapolating the ratio $f_{D_s}/m_{D_s}$, differently from ETM 13B, where $f_{D_s}r_0$ was extrapolated. The new choice is found to be affected by smaller discretization effects. For the chiral/continuum extrapolation terms linear and quadratic in $m_l$ and one term linear in $a^2$ are included in the parameterization. Systematic uncertainties are assessed by comparing to a linear fit in $m_l$ and by taking the difference with the result at the finest lattice resolution. The decay constant $f_D$ is determined by fitting the double ratio $(f_{D_s}/f_D)/(f_K/f_\pi )$ using continuum HM$\chi$PT, as discretization effects are not visible, within errors, for that quantity. An alternative fit without chiral logs is used to estimate the systematic uncertainty associated to the chiral extrapolation. The main systematic uncertainties are due to the continuum and chiral extrapolations and to the error on $f_K/f_\pi$, which is also determined in ETM 14E. Using the experimental averages of $f_D|V_{cd}|$ and $f_{D_s}|V_{cs}|$ available in 2014 from PDG [151], the ETM Collaboration also provides a unitarity test of the second row of the CKM matrix, obtaining $1- |V_{cd}|^2 - |V_{cs}|^2 - |V_{cb}|^2 =-0.08(5)$, which is consistent with the estimate from FNAL/MILC 14A and with the value in the latest PDG report [184], which quotes $-0.063(34)$ for the same combination of matrix elements. That indicates a slight tension with three-generation unitarity.

Finally, by combining in a weighted average the FNAL/MILC 14A and the ETM 14E results, we get the estimates129where the error on the average of $f_{D}$ has been rescaled by the factor $\sqrt{\chi ^2/\hbox {d.o.f.}}=1.3$. The PDG [151] produces *experimental* averages of the decay constants, by combining the measurements of $f_D|V_{cd}|$ and $f_{D_s}|V_{cs}|$ with values of $|V_{cd}|$ and $|V_{cs}|$ obtained by relating them to other CKM elements (i.e., by assuming unitarity). Given the choices detailed in Ref. [151], the values read

130 $$\begin{aligned} f_{D^+}^{\mathrm{exp}}=203.7 (4.8)~\mathrm{MeV}, \quad f_{D_s^+}^{\mathrm{exp}}= 257.8 (4.1)~\mathrm{MeV}, \end{aligned}$$

which disagree with the $N_{ f}=2+1+1$ lattice averages in Eq. (129) at the two-sigma level.

### Semileptonic form factors for $D\rightarrow \pi \ell \nu$ and $D\rightarrow K \ell \nu$

The form factors for semileptonic $D\rightarrow \pi \ell \nu$ and $D\rightarrow K \ell \nu$ decays, when combined with experimental measurements of the decay widths, enable determinations of the CKM matrix elements $|V_{cd}|$ and $|V_{cs}|$ via:

131 $$\begin{aligned}&\frac{\mathrm{d}\Gamma (D\rightarrow P\ell \nu )}{\mathrm{d}q^2} = \frac{G_F^2 |V_{cx}|^2}{24 \pi ^3} \,\frac{(q^2-m_\ell ^2)^2\sqrt{E_P^2-m_P^2}}{q^4m_{D}^2} \,\nonumber \\&\quad \times \left[ \left( 1+\frac{m_\ell ^2}{2q^2}\right) m_{D}^2(E_P^2-m_P^2)|f_+(q^2)|^2\right. \nonumber \\&\quad +\, \left. \frac{3m_\ell ^2}{8q^2}(m_{D}^2-m_P^2)^2|f_0(q^2)|^2 \right] , \end{aligned}$$

where $x = d, s$ is the daughter light quark, $P= \pi , K$ is the daughter light pseudoscalar meson, and $q = (p_D - p_P)$ is the momentum of the outgoing lepton pair. The vector and scalar form factors $f_+(q^2)$ and $f_0(q^2)$ parameterize the hadronic matrix element of the heavy-to-light quark flavour-changing vector current $V_\mu = \overline{x} \gamma _\mu c$:

132 $$\begin{aligned} \langle P| V_\mu | D \rangle= & {} f_+(q^2) \left( {p_D}_\mu + {p_P}_\mu - \frac{m_D^2 - m_P^2}{q^2}\,q_\mu \right) \nonumber \\&+\, f_0(q^2) \frac{m_D^2 - m_P^2}{q^2}\,q_\mu , \end{aligned}$$

and satisfy the kinematic constraint $f_+(0) = f_0(0)$. Because the contribution to the decay width from the scalar form factor is proportional to $m_\ell ^2$, it can be neglected for $\ell = e, \mu$, and Eq. (131) simplifies to

133 $$\begin{aligned} \frac{\mathrm{d}\Gamma \!\left( D \rightarrow P \ell \nu \right) }{\mathrm{d} q^2} = \frac{G_F^2}{24 \pi ^3} |\vec {p}_{P}|^3 {|V_{cx}|^2 |f_+^{DP} (q^2)|^2}. \end{aligned}$$

In practice, most lattice-QCD calculations of $D\rightarrow \pi \ell \nu$ and $D\rightarrow K \ell \nu$ focus on providing the value of the vector form factor at a single value of the momentum transfer, $f_+(q^2=0)$, which is sufficient to obtain $|V_{cd}|$ and $|V_{cs}|$. Because the decay rate cannot be measured directly at $q^2=0$, comparison of these lattice-QCD results with experiment requires a slight extrapolation of the experimental measurement. Some lattice-QCD calculations also provide determinations of the $D\rightarrow \pi \ell \nu$ and $D\rightarrow K \ell \nu$ form factors over the full kinematic range $0< q^2 < q^2_\mathrm{max} = (m_D - m_P)^2$, thereby allowing a comparison of the shapes of the lattice simulation and experimental data. This nontrivial test in the *D* system provides a strong check of lattice-QCD methods that are also used in the *B*-meson system.

Lattice-QCD calculations of the $D\rightarrow \pi \ell \nu$ and $D\rightarrow K \ell \nu$ form factors typically use the same light-quark and charm-quark actions as those of the leptonic decay constants $f_D$ and $f_{D_s}$. Therefore many of the same issues arise, e.g., chiral extrapolation of the light-quark mass(es) to the physical point, discretization errors from the charm quark, and matching the lattice weak operator to the continuum, as discussed in the previous section. Two strategies have been adopted to eliminate the need to renormalize the heavy–light vector current in recent calculations of $D\rightarrow \pi \ell \nu$ and $D\rightarrow K \ell \nu$, both of which can be applied to simulations in which the same relativistic action is used for the light (*u*, *d*, *s*) and charm quarks. The first method was proposed by Bećirević and Haas in Ref. [428], and introduces double-ratios of lattice 3-point correlation functions in which the vector-current renormalization cancels. Discretization errors in the double ratio are of $\mathcal {O}((am_h)^2)$ provided that the vector-current matrix elements are $\mathcal {O}(a)$ improved. The vector and scalar form factors $f_+(q^2)$ and $f_0(q^2)$ are obtained by taking suitable linear combinations of these double ratios. The second method was introduced by the HPQCD Collaboration in Ref. [51]. In this case, the quantity $(m_{c} - m_{x} ) \langle P | S | D \rangle$, where $m_{x}$ and $m_c$ are the bare lattice quark masses and $S = \bar{x}c$ is the lattice scalar current, does not get renormalized. The desired form factor at $q^2=0$ can be obtained by (i) using a Ward identity to relate the matrix element of the vector current to that of the scalar current, and (ii) taking advantage of the kinematic identity $f_+(0) = f_0(0)$, such that $f_+(q^2=0) = (m_{c} - m_{x} ) \langle P | S | D \rangle / (m^2_D - m^2_P)$.

Additional complications enter for semileptonic decay matrix elements due to the nonzero momentum of the outgoing pion or kaon. Both statistical errors and discretization errors increase at larger meson momenta, so results for the lattice form factors are most precise at $q^2_\mathrm{max}$. However, because lattice calculations are performed in a finite spatial volume, the pion or kaon three-momentum can only take discrete values in units of $2\pi /L$ when periodic boundary conditions are used. For typical box sizes in recent lattice *D*- and *B*-meson form-factor calculations, $L \sim 2.5{-}3$ fm; thus the smallest nonzero momentum in most of these analyses lies in the range $p_P \equiv |\vec {p}_P| \sim 400{-}500$ MeV. The largest momentum in lattice heavy–light form-factor calculations is typically restricted to $p_P \le 4\pi /L$. For $D \rightarrow \pi \ell \nu$ and $D \rightarrow K \ell \nu$, $q^2=0$ corresponds to $p_\pi \sim 940$ MeV and $p_K \sim 1$ GeV, respectively, and the full recoil-momentum region is within the range of accessible lattice momenta.^34^ Therefore the interpolation to $q^2=0$ is relatively insensitive to the fit function used to parameterize the momentum dependence, and the associated systematic uncertainty in $f_+(0)$ is small. In contrast, determinations of the form-factor shape can depend strongly on the parameterization of the momentum dependence, and the systematic uncertainty due to the choice of model function is often difficult to quantify. This is becoming relevant for $D \rightarrow \pi \ell \nu$ and $D \rightarrow K \ell \nu$ decays as more collaborations are beginning to present results for $f_+(q^2)$ and $f_0(q^2)$ over the full kinematic range. The parameterization of the form-factor shape is even more important for semileptonic *B* decays, for which the momentum range needed to connect to experiment is often far from $q^2_{\mathrm{max}}$.

A class of functions based on general field-theory properties, known as *z*-expansions, has been introduced to allow model-independent parameterizations of the $q^2$ dependence of semileptonic form factors over the entire kinematic range (see, e.g., Refs. [435, 436]). The use of such functions is now standard for the analysis of $B \rightarrow \pi \ell \nu$ transitions and the determination of $|V_{ub}|$ [437–440]; we therefore discuss approaches for parameterizing the $q^2$ dependence of semileptonic form factors, including *z*-expansions, in Sect. 8.3. Here we briefly summarize the aspects most relevant to calculations of $D \rightarrow \pi \ell \nu$ and $D \rightarrow K \ell \nu$. In general, all semileptonic form factors can be expressed as a series expansion in powers of *z* times an overall multiplicative function that accounts for any sub-threshold poles and branch cuts, where the new variable *z* is a nonlinear function of $q^2$. The series coefficients $a_n$ depend upon the physical process (as well as the choice of the prefactors), and can only be determined empirically by fits to lattice or experimental data. Unitarity establishes strict upper bounds on the size of the $a_n$’s, while guidance from heavy-quark power counting provides even tighter constraints. Some work now is using a variation of this approach, commonly referred to as “modified *z*-expansion,” which is used to simultaneously extrapolate their lattice simulation data to the physical light-quark masses and the continuum limit, and to interpolate/extrapolate their lattice data in $q^2$. More comments on this method are also provided in Sect. 8.3.

#### Results for $f_+(0)$

We now review the status of lattice calculations of the $D \rightarrow \pi \ell \nu$ and $D \rightarrow K \ell \nu$ form factors at $q^2=0$. As in the previous version of this review, although we also describe on-going calculations of the form-factor shapes, we do not rate these calculations, since all of them are still unpublished, except for conference proceedings that provide only partial results.^35^

The most advanced $N_f = 2$ lattice-QCD calculation of the $D \rightarrow \pi \ell \nu$ and $D \rightarrow K \ell \nu$ form factors is by the ETM Collaboration [431]. This still preliminary work uses the twisted-mass Wilson action for both the light and charm quarks, with three lattice spacings down to $a \approx 0.068$ fm and (charged) pion masses down to $m_\pi \approx 270$ MeV. The calculation employs the ratio method of Ref. [428] to avoid the need to renormalize the vector current, and extrapolates to the physical light-quark masses using *SU*(2) heavy–light meson $\chi$PT. ETM simulate with nonperiodic boundary conditions for the valence quarks to access arbitrary momentum values over the full physical $q^2$ range, and interpolate to $q^2=0$ using the Bećirević-Kaidalov ansatz [442]. The statistical errors in $f_+^{D\pi }(0)$ and $f_+^{DK}(0)$ are 9 and 7%, respectively, and lead to rather large systematic uncertainties in the fits to the light-quark mass and energy dependence (7 and 5%, respectively). Another significant source of uncertainty is from discretization errors (5 and 3%, respectively). On the finest lattice spacing used in this analysis $am_c \sim 0.17$, so $\mathcal {O}((am_c)^2)$ cutoff errors are expected to be about 5%. This can be reduced by including the existing $N_f = 2$ twisted-mass ensembles with $a \approx 0.051$ fm discussed in Ref. [36]. Work is in progress by the ETM Collaboration also to compute the form factors $f_+^{D\pi },~f_0^{D\pi }$ and $f_+^{DK},~f_0^{DK}$ for the whole kinematically available range on the $N_f = 2+1+1$ twisted-mass Wilson lattices [39]. This calculation will include dynamical charm-quark effects and use three lattice spacings down to $a\approx 0.06$ fm. A BCL *z*-parameterization is being used to describe the $q^2$ dependence. The latest progress report on this work, which provides values of the form factors at $q^2=0$ with statistical errors only, can be found in Ref. [443].

The first published $N_f = 2+1$ lattice-QCD calculation of the $D \rightarrow \pi \ell \nu$ and $D \rightarrow K \ell \nu$ form factors is by the Fermilab Lattice, MILC, and HPQCD Collaborations [441]. (Because only two of the authors of this work are in HPQCD, and to distinguish it from other more recent work on the same topic by HPQCD, we hereafter refer to this work as “FNAL/MILC.”) This work uses asqtad-improved staggered sea quarks and light (*u*, *d*, *s*) valence quarks and the Fermilab action for the charm quarks, with a single lattice spacing of $a \approx 0.12$ fm. At this lattice spacing, the staggered taste splittings are still fairly large, and the minimum RMS pion mass is ${\approx } 510$ MeV. This calculation renormalizes the vector current using a mostly nonperturbative approach, such that the perturbative truncation error is expected to be negligible compared to other systematics. The Fermilab Lattice and MILC Collaborations present results for the $D \rightarrow \pi \ell \nu$ and $D \rightarrow K \ell \nu$ semileptonic form factors over the full kinematic range, rather than just at $q^2=0$. In fact, the publication of this result predated the precise measurements of the $D\rightarrow K \ell \nu$ decay width by the FOCUS [444] and Belle experiments [445], and predicted the shape of $f_+^{DK}(q^2)$ quite accurately. This bolsters confidence in calculations of the *B*-meson semileptonic decay form factors using the same methodology. Work is in progress [446] to reduce both the statistical and systematic errors in $f_+^{D\pi }(q^2)$ and $f_+^{DK}(q^2)$ through increasing the number of configurations analysed, simulating with lighter pions, and adding lattice spacings as fine as $a \approx 0.045$ fm. In parallel, a much more ambitious computation of $D \rightarrow \pi \ell \nu$ and $D \rightarrow K \ell \nu$ by FNAL/MILC is now on-going, using $N_f=2+1+1$ MILC HISQ ensembles at four values of the lattice spacing down to $a=0.042~\mathrm{fm}$ and pion masses down to the physical point. The latest report on this computation, focusing on the form factors at $q^2=0$, but without explicit values of the latter yet, can be found in Ref. [447].

The most precise published calculations of the $D \rightarrow \pi \ell \nu$ [50] and $D \rightarrow K \ell \nu$ [51] form factors are by the HPQCD Collaboration. These analyses also use the $N_f = 2+1$ asqtad-improved staggered MILC configurations at two lattice spacings $a \approx 0.09$ and 0.12 fm, but use the HISQ action for the valence *u*, *d*, *s*, and *c* quarks. In these mixed-action calculations, the HISQ valence light-quark masses are tuned so that the ratio $m_l/m_s$ is approximately the same as for the sea quarks; the minimum RMS sea-pion mass is $\approx 390$ MeV. They calculate the form factors at $q^2=0$ by relating them to the matrix element of the scalar current, which is not renormalized. They use the “modified *z*-expansion” to simultaneously extrapolate to the physical light-quark masses and continuum and interpolate to $q^2 = 0$, and allow the coefficients of the series expansion to vary with the light- and charm-quark masses. The form of the light-quark dependence is inspired by $\chi$PT, and includes logarithms of the form $m_\pi ^2 \mathrm{log} (m_\pi ^2)$ as well as polynomials in the valence-, sea-, and charm-quark masses. Polynomials in $E_{\pi (K)}$ are also included to parameterize momentum-dependent discretization errors. (See Ref.  [50] for further technical details.) The number of terms is increased until the result for $f_+(0)$ stabilizes, such that the quoted fit error for $f_+(0)$ includes both statistical uncertainties and those due to most systematics. The largest uncertainties in these calculations are from statistics and charm-quark discretization errors.

The HPQCD Collaboration is now extending their work on *D*-meson semileptonic form factors to determining their shape over the full kinematic range [432], and recently obtained results for the $D \rightarrow K \ell \nu$ form factors $f_+(q^2)$ and $f_0(q^2)$ [433]. This analysis uses a subset of the ensembles included in their earlier work, with two sea-quark masses at $a \approx 0.12$ fm and one sea-quark mass at $a \approx 0.09$ fm, but with approximately three times more statistics on the coarser ensembles and ten times more statistics on the finer ensemble. As above, the scalar current is not renormalized. The spatial vector-current renormalization factor is obtained by requiring that $f_+(0)^{H\rightarrow H} = 1$ for $H = D, D_s, \eta _s$, and $\eta _c$. The renormalization factors for the flavour-diagonal currents agree for different momenta as well as for charm–charm and strange–strange external mesons within a few percent, and they are then used to renormalize the flavour-changing charm–strange and charm-light currents. The charm–strange temporal vector current is normalized by matching to the scalar current $f_0(q^2_\mathrm{max})$. Also as above, they simultaneously extrapolate to the physical light-quark masses and continuum and interpolate/extrapolate in $q^2$ using the modified *z*-expansion. In this case, however, they only allow for light-quark mass and lattice-spacing dependence in the series coefficients, but not for charm-quark mass or kaon energy dependence, and constrain the parameters with Bayesian priors. It is not clear, however, whether only three sea-quark ensembles at two lattice spacings are sufficient to resolve the quark-mass and lattice-spacing dependence, even within the context of constrained fitting. The quoted error in the zero-recoil form factor $f_+(0) = 0.745(11)$ is significantly smaller than in their 2010 work, but we are unable to understand the sources of this improvement with the limited information provided in Ref. [433]. The preprint does not provide an error budget, nor any information on how the systematic uncertainties are estimated. Thus we cannot rate this calculation, and do not include it in the summary table and plot.

**Table 29:** $D \rightarrow \pi \ell \nu$ and $D\rightarrow K\ell \nu$ semileptonic form factors at $q^2=0$

Collaboration	Refs.	$N_{ f}$	Publication status	Continuum extrapolation	Chiral extrapolation	Finite volume	Renormalization	Heavy-quark treatment	$f_+^{D\pi }(0)$	$f_+^{DK}(0)$
HPQCD 11	[50]	$2+1$	A						0.666(29)	
HPQCD 10B	[51]	$2+1$	A							0.747(19)
FNAL/MILC 04	[441]	$2+1$	A						0.64(3)(6)	0.73(3)(7)
ETM 11B	[431]	2	C						0.65(6)(6)	0.76(5)(5)

**Fig. 18:**
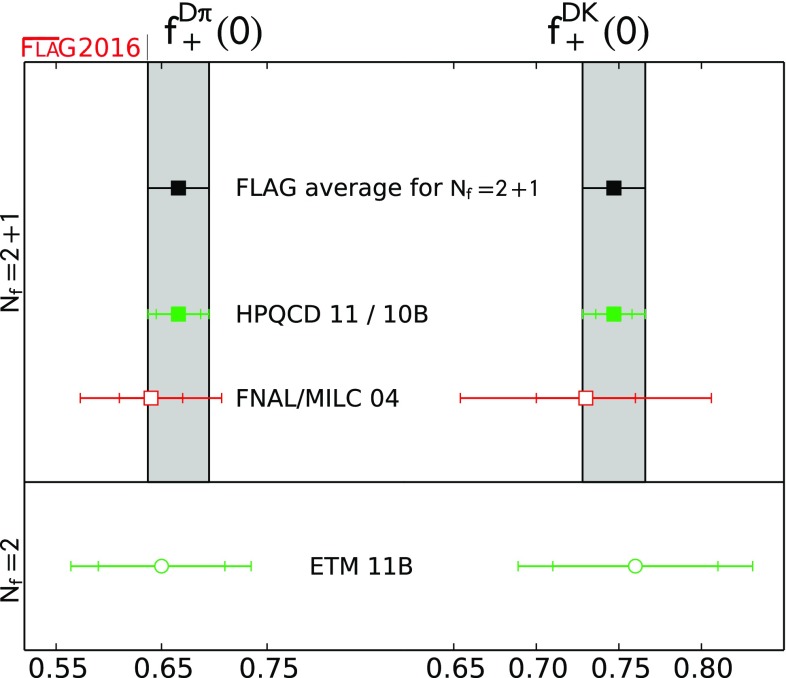
$D\rightarrow \pi \ell \nu$ and $D\rightarrow K\ell \nu$ semileptonic form factors at $q^2=0$. The HPQCD result for $f_+^{D\pi }(0)$ is from HPQCD 11, the one for $f_+^{DK}(0)$ represents HPQCD 10B (see Table 29)

Table 29 summarizes the existing $N_f =2$ and $N_f = 2+1$ calculations of the $D \rightarrow \pi \ell \nu$ and $D \rightarrow K \ell \nu$ semileptonic form factors. The quality of the systematic error studies is indicated by the symbols. Additional tables in Appendix B.5.2 provide further details of the simulation parameters and comparisons of the error estimates. Recall that only calculations without red tags that are published in a refereed journal are included in the FLAG average. Of the calculations described above, only those of HPQCD 10B,11 satisfy all of the quality criteria. Therefore our average of the $D \rightarrow \pi \ell \nu$ and $D \rightarrow K \ell \nu$ semileptonic form factors from $N_f = 2+1$ lattice QCD is134Figure 18 displays the existing $N_f =2$ and $N_f = 2+1$ results for $f_+^{D\pi }(0)$ and $f_+^{DK}(0)$; the grey bands show our average of these quantities. Section 7.3 discusses the implications of these results for determinations of the CKM matrix elements $|V_{cd}|$ and $|V_{cs}|$ and tests of unitarity of the second row of the CKM matrix.

### Determinations of $|V_{cd}|$ and $|V_{cs}|$ and test of second-row CKM unitarity

We now interpret the lattice-QCD results for the $D_{(s)}$ meson decays as determinations of the CKM matrix elements $|V_{cd}|$ and $|V_{cs}|$ in the Standard Model.

**Table 30:** Determinations of $|V_{cd}|$ (upper panel) and $|V_{cs}|$ (lower panel) obtained from lattice calculations of *D*-meson leptonic decay constants and semileptonic form factors. The errors shown are from the lattice calculation and experiment (plus nonlattice theory), respectively

Collaboration	Refs.	$N_{ f}$	From	$|V_{cd}|$ or $|V_{cs}|$
FNAL/MILC 14A	[14]	$$2+1+1$$	$$ f_D$$	0.2159(12)(49)
ETM 14E	[27]	$$2+1+1$$	$$ f_D$$	0.2214(41)(51)
HPQCD 12A	[47]	$2+1$	$f_{D}$	0.2204(36)(50)
HPQCD 11	[50]	$2+1$	$D \rightarrow \pi \ell \nu$	0.2140(93)(29)
FNAL/MILC 11	[48]	$2+1$	$f_{D}$	0.2097(108)(48)
ETM 13B	[20]	2	$f_{D}$	0.2207(74)(50)
FNAL/MILC 14A	[14]	$$2+1+1$$	$$ f_{D_s}$$	1.008(5)(16)
ETM 14E	[27]	$$2+1+1$$	$$ f_{D_s}$$	1.015(17)(16)
HPQCD 10A	[49]	$2+1$	$f_{D_s}$	1.012(10)(16)
FNAL/MILC 11	[48]	$2+1$	$f_{D_s}$	0.965(40)(16)
HPQCD 10B	[51]	$2+1$	$D \rightarrow K \ell \nu$	0.975(25)(7)
$\chi$QCD 14	[17]	$2+1$	$f_{D_s}$	0.988(17)(16)
ETM 13B	[20]	2	$f_{D_s}$	1.004(28)(16)

For the leptonic decays, we use the latest experimental averages from Rosner, Stone and Van de Water for the Particle Data Group [184]

135 $$\begin{aligned}&f_D |V_{cd}| = 45.91(1.05)~\mathrm{MeV},\nonumber \\&\quad f_{D_s} |V_{cs}| = 250.9(4.0)~\mathrm{MeV}. \end{aligned}$$

By combining these with the average values of $f_D$ and $f_{D_s}$ from the individual $N_f = 2$, $N_f = 2+1$ and $N_f=2+1+1$ lattice-QCD calculations that satisfy the FLAG criteria, we obtain the results for the CKM matrix elements $|V_{cd}|$ and $|V_{cs}|$ in Table 30. For our preferred values we use the averaged $N_f=2$ and $N_f = 2+1$ results for $f_D$ and $f_{D_s}$ in Eqs. (127), (128) and (129). We obtain

136 $$\begin{aligned}&\mathrm{leptonic~decays}, N_f=2+1+1:|V_{cd}| = 0.2164(14)(49),\nonumber \\&\quad |V_{cs}| = 1.008 (5)(16), \end{aligned}$$

137 $$\begin{aligned}&\mathrm{leptonic~decays}, N_f=2+1: |V_{cd}| = 0.2195(35)(50),\nonumber \\&\quad |V_{cs}| = 1.004 (9)(16), \end{aligned}$$

138 $$\begin{aligned}&\mathrm{leptonic~decays}, N_f=2: |V_{cd}| = 0.2207(74)(50),\nonumber \\&\quad |V_{cs}| = 1.004 (28)(16), \end{aligned}$$

**Table 31:** Comparison of determinations of $|V_{cd}|$ and $|V_{cs}|$ obtained from lattice methods with nonlattice determinations and the Standard Model prediction assuming CKM unitarity

	From	Refs.	$|V_{cd}|$	$|V_{cs}|$
$N_f = 2+1+1$	$f_D$ and $f_{D_s}$		0.2164(51)	1.008(17)
$N_f = 2+1$	$f_D$ and $f_{D_s}$		0.2195(61)	1.004(18)
$N_f = 2$	$f_D$ and $f_{D_s}$		0.2207(89)	1.004(32)
$N_f = 2+1$	$D \rightarrow \pi \ell \nu$ and $D\rightarrow K \ell \nu$		0.2140(97)	0.975(26)
PDG	Neutrino scattering	[151]	0.230(11)	
Rosner 15 (*for the* PDG)	CKM unitarity	[184]	0.2254(7)	0.9733(2)

where the errors shown are from the lattice calculation and experiment (plus nonlattice theory), respectively. For the $N_f = 2+1$ and the $N_f=2+1+1$ determinations, the uncertainties from the lattice-QCD calculations of the decay constants are smaller than the experimental uncertainties in the branching fractions. Although the results for $|V_{cs}|$ are slightly larger than one, they are consistent with unity within errors.

The leptonic determinations of these CKM matrix elements have uncertainties that are reaching the few-percent level. However, higher-order electroweak and hadronic corrections to the rate have not been computed for the case of $D_{(s)}$ mesons, whereas they have been estimated to be around 1–2% for pion and kaon decays [448]. It is therefore important that such theoretical calculations are tackled soon, perhaps directly on the lattice, as proposed in Ref. [449].

For the semileptonic decays, there is no update on the lattice side from the previous version of our review. As experimental input for the determination of $|V_{cb}|$ we use the latest experimental averages from the Heavy Flavour Averaging Group [197]:

139 $$\begin{aligned} f_+^{D\pi }(0) |V_{cd}| = 0.1425(19),\quad f_+^{DK}(0) |V_{cs}| = 0.728(5).\nonumber \\ \end{aligned}$$

For each of $f_+^{D\pi }(0)$ and $f_+^{DK}(0)$, there is only a single $N_f = 2+1$ lattice-QCD calculation that satisfies the FLAG criteria. Using these results, which are given in Eq. (134), we obtain our preferred values for $|V_{cd}|$ and $|V_{cs}|$:

140 $$\begin{aligned}&|V_{cd}| = 0.2140(93)(29),\quad |V_{cs}| = 0.975(25)(7),\nonumber \\&\quad (\mathrm{semileptonic~decays}, N_f=2+1) \end{aligned}$$

where the errors shown are from the lattice calculation and experiment (plus nonlattice theory), respectively. These values are compared with individual leptonic determinations in Table 30.

**Fig. 19:**
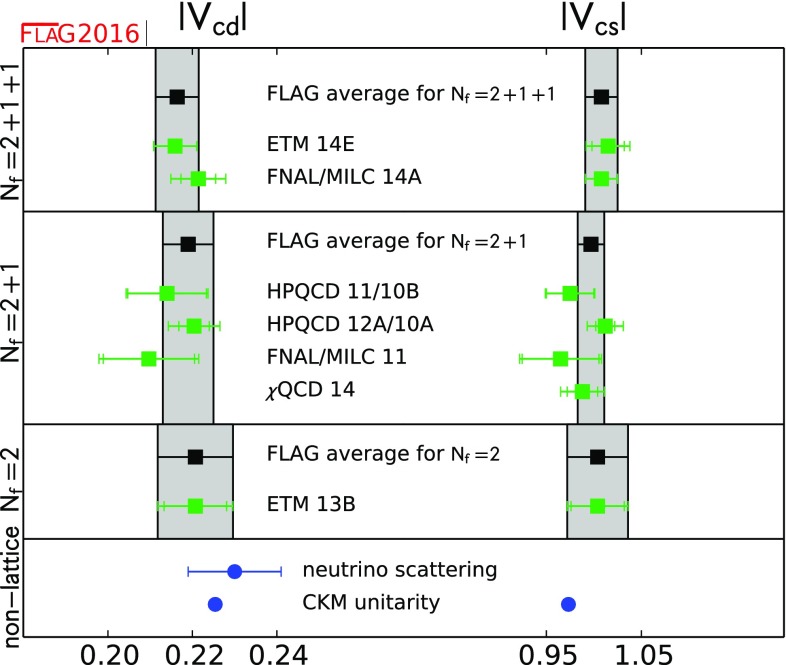
Comparison of determinations of $|V_{cd}|$ and $|V_{cs}|$ obtained from lattice methods with nonlattice determinations and the Standard Model prediction based on CKM unitarity. When two references are listed on a single row, the first corresponds to the lattice input for $|V_{cd}|$ and the second to that for $|V_{cs}|$. The results denoted by *squares* are from leptonic decays, while those denoted by *triangles* are from semileptonic decays

Table 31 summarizes the results for $|V_{cd}|$ and $|V_{cs}|$ from leptonic and semileptonic decays, and compares them to determinations from neutrino scattering (for $|V_{cd}|$ only) and CKM unitarity. These results are also plotted in Fig. 19. For both $|V_{cd}|$ and $|V_{cs}|$, the errors in the direct determinations from leptonic and semileptonic decays are approximately one order of magnitude larger than the indirect determination from CKM unitarity. Some tensions at the 2$\sigma$ level are present between the direct and the indirect estimates, namely in $|V_{cd}|$ using the $N_f=2+1+1$ lattice result and in $|V_{cs}|$ using both the $N_f=2+1$ and the $N_f=2+1+1$ values.

In order to provide final estimates, for $N_f=2$ and $N_f=2+1+1$ we take the only available results coming from leptonic decays, while for $N_f=2+1$ we average leptonic and semileptonic channels. For this purpose, we assume that the statistical errors are 100% correlated between the FNAL/MILC and HPQCD computations because they use the MILC asqtad gauge configurations. We also assume that the heavy-quark discretization errors are 100% correlated between the HPQCD calculations of leptonic and semileptonic decays because they use the same charm-quark action, and that the scale-setting uncertainties are 100% correlated between the HPQCD results as well. Finally, we include the 100% correlation between the experimental inputs for the two extractions of $|V_{cd(s)}|$ from leptonic decays. We finally quote

141 $$\begin{aligned}&\mathrm{our~average}, N_f=2+1+1:|V_{cd}| = 0.2164(51) ,\nonumber \\&\quad |V_{cs}| = 1.008(17) , \end{aligned}$$

142 $$\begin{aligned}&\mathrm{our~average}, N_f=2+1: |V_{cd}| = 0.2190(60) ,\nonumber \\&\quad |V_{cs}| = 0.997(14) , \end{aligned}$$

143 $$\begin{aligned}&\mathrm{our~average}, N_f=2: |V_{cd}| = 0.2207(89) ,\nonumber \\&\quad |V_{cs}| = 1.004(32), \end{aligned}$$

where the errors include both theoretical and experimental uncertainties.

Using the lattice determinations of $|V_{cd}|$ and $|V_{cs}|$ in Table 31, we can test the unitarity of the second row of the CKM matrix. We obtain

144 $$\begin{aligned}&N_f=2+1+1:&|V_{cd}|^2 + |V_{cs}|^2 + |V_{cb}|^2 - 1&= 0.06(3) \,,\end{aligned}$$

145 $$\begin{aligned}&N_f=2+1:&|V_{cd}|^2 + |V_{cs}|^2 + |V_{cb}|^2 - 1&= 0.04(3) \,, \end{aligned}$$

146 $$\begin{aligned}&N_f=2:&|V_{cd}|^2 + |V_{cs}|^2 + |V_{cb}|^2 - 1&= 0.06(7) \,. \end{aligned}$$

Again, tensions at the 2$\sigma$ level with CKM unitarity are visible, as also reported in the PDG review [184], where the value 0.063(34) is quoted for the quantity in the equations above. Given the current level of precision, this result does not depend on $|V_{cb}|$, which is of $\mathcal {O}(10^{-2})$.

## *B*-meson-decay constants, mixing parameters and form factors

The (semi)leptonic decay and mixing processes of $B_{(s)}$ mesons have been playing a crucial role in flavour physics. In particular, they contain important information for the investigation of the $b{-}d$ unitarity triangle in the Cabibbo–Kobayashi–Maskawa (CKM) matrix, and can be ideal probes to physics beyond the Standard Model. The charged-current decay channels $B^{+} \rightarrow l^{+} \nu _{l}$ and $B^{0} \rightarrow \pi ^{-} l^{+} \nu _{l}$, where $l^{+}$ is a charged lepton with $\nu _{l}$ being the corresponding neutrino, are essential in extracting the CKM matrix element $|V_{ub}|$. Similarly, the *B* to $D^{(*)}$ semileptonic transitions can be used to determine $|V_{cb}|$. The flavour changing neutral current (FCNC) processes, such as $B\rightarrow K^{(*)} \ell ^+ \ell ^-$ and $B_{d(s)} \rightarrow \ell ^+ \ell ^-$, occur only beyond the tree level in weak interactions and are suppressed in the Standard Model. Therefore, these processes can be sensitive to new physics, since heavy particles can contribute to the loop diagrams. They are also suitable channels for the extraction of the CKM matrix elements involving the top quark which can appear in the loop. For instance, the neutral $B_{d(s)}$-meson mixings are FCNC processes and are dominated by the one-loop “box” diagrams containing the top quark and the *W* bosons. Thus, using the experimentally measured neutral $B^0_{d(s)}$-meson oscillation frequencies, $\Delta M_{d(s)}$, and the theoretical calculations for the relevant hadronic mixing matrix elements, one can obtain $|V_{td}|$ and $|V_{ts}|$ in the Standard Model.^36^

Accommodating the light quarks and the *b* quark simultaneously in lattice-QCD computations is a challenging endeavour. To incorporate the pion and the *b* hadrons with their physical masses, the simulations have to be performed using the lattice size $\hat{L} = L/a \sim \mathcal {O}(10^{2})$, where *a* is the lattice spacing and *L* is the physical (dimensionful) box size. This is a few times larger than what one can practically afford in contemporary numerical projects. Therefore, in addition to employing Chiral Perturbation Theory for the extrapolations in the light-quark mass, current lattice calculations for quantities involving *b* hadrons often make use of effective theories that allow one to expand in inverse powers of $m_{b}$. In this regard, two general approaches are widely adopted. On the one hand, effective field theories such as Heavy-Quark Effective Theory (HQET) and Nonrelativistic QCD (NRQCD) can be directly implemented in numerical computations. On the other hand, a relativistic quark action can be improved *á la* Symanzik to suppress cutoff errors, and then re-interpreted in a manner that is suitable for heavy-quark physics calculations. This latter strategy is often referred to as the method of the Relativistic Heavy-Quark Action (RHQA). The utilization of such effective theories inevitably introduces systematic uncertainties that are not present in light-quark calculations. These uncertainties can arise from the truncation of the expansion in constructing the effective theories (as in HQET and NRQCD), or from more intricate cutoff effects (as in NRQCD and RQHA). They can also be introduced through more complicated renormalization procedures which often lead to significant systematic effects in matching the lattice operators to their continuum counterparts. For instance, due to the use of different actions for the heavy and the light quarks, it is more difficult to construct absolutely normalized bottom-light currents.

Complementary to the above “effective-theory approaches”, another popular method is to simulate the heavy and the light quarks using the same (normally improved) lattice action at several values of the heavy-quark mass, $m_{h}$, with $a m_{h} < 1$ and $m_{h} < m_{b}$. This enables one to employ HQET-inspired relations to extrapolate the computed quantities to the physical *b* mass. When combined with results obtained in the static heavy-quark limit, this approach can be rendered into an interpolation, instead of extrapolation, in $m_{h}$. The discretization errors are the main source of the systematic effects in this method, and very small lattice spacings are needed to keep such errors under control.

Because of the challenge described above, the efforts that have been made to obtain reliable, accurate lattice-QCD results for physics of the *b* quark have been enormous. These efforts include significant theoretical progress in formulating QCD with heavy quarks on the lattice. This aspect is briefly reviewed in Appendix A.1.3.

In this section, we summarize the results of the *B*-meson leptonic decay constants, the neutral *B*-mixing parameters, and the semileptonic form factors, from lattice QCD. To be focussed on the calculations which have strong phenomenological impact, we limit the review to results based on modern simulations containing dynamical fermions with reasonably light pion masses (below approximately 500 MeV). Compared to the progress in the light-quark sector, heavy-quark physics on the lattice is not as mature. Consequently, fewer collaborations have finished calculations for these quantities. In addition, the existing results are often obtained at coarser lattice spacings and heavier pions. Therefore, for some quantities, there is only a single lattice calculation that satisfies the criteria to be included in our average. Nevertheless, several collaborations are currently pursuing this line of research with various lattice *b*-quark actions, finer lattice spacings, and lighter pions. Thus many new results with controlled errors are expected to appear in the near future.

Following our review of the $B_{(s)}$-meson leptonic decay constants, the neutral *B*-meson mixing parameters, and semileptonic form factors, we then interpret our results within the context of the Standard Model. We combine our best-determined values of the hadronic matrix elements with the most recent experimentally measured branching fractions to obtain $|V_{(u)cb}|$ and compare these results to those obtained from inclusive semileptonic *B* decays.

Recent lattice-QCD averages for $B^+$- and $B_s$-meson decay constants were also presented by the Particle Data Group (PDG) in Ref. [184]. The PDG three- and four-flavour averages for these quantities differ from those quoted here because the PDG provides the charged-meson-decay constant, $f_{B^+}$, while we present the isospin-averaged meson-decay constant, $f_B$.

### Leptonic decay constants $f_B$ and $f_{B_s}$

The *B*- and $B_s$-meson-decay constants are crucial input for extracting information from leptonic *B* decays. Charged *B* mesons can decay to the lepton-neutrino final state through the charged-current weak interaction. On the other hand, neutral $B_{d(s)}$ mesons can decay to a charged-lepton pair via a flavour-changing neutral current (FCNC) process.

In the Standard Model the decay rate for $B^+ \rightarrow \ell ^+ \nu _{\ell }$ is described by a formula identical to Eq. (125), with $D_{(s)}$ replaced by *B*, and the relevant CKM matrix element, $V_{cq}$, substituted by $V_{ub}$,

147 $$\begin{aligned} \Gamma ( B \rightarrow \ell \nu _{\ell } ) = \frac{ m_B}{8 \pi } G_F^2 f_B^2 |V_{ub}|^2 m_{\ell }^2 \left( 1-\frac{ m_{\ell }^2}{m_B^2} \right) ^2. \end{aligned}$$

The only charged-current *B* meson decay that has been observed so far is $B^{+} \rightarrow \tau ^{+} \nu _{\tau }$, which has been measured by the Belle and Babar Collaborations [451, 452]. Both collaborations have reported results with errors around $20\%$. These measurements can be used to determine $|V_{ub}|$ when combined with lattice-QCD predictions of the corresponding decay constant.

Neutral $B_{d(s)}$-meson decays to a charged-lepton pair, $B_{d(s)} \rightarrow l^{+} l^{-}$ is a FCNC process, and can only occur at one-loop in the Standard Model. Hence these processes are expected to be rare, and they are sensitive to physics beyond the Standard Model. The corresponding expression for the branching fraction has the form

148 $$\begin{aligned}&B(B_q \rightarrow \ell ^+ \ell ^-)\nonumber \\&\quad = \frac{\tau _{B_q}}{1+y_q} \frac{G_F^2 \alpha ^2 }{ 16\pi ^3} m_{B_q} f_{B_q}^2 \vert V_{tb}^*V_{tq}\vert ^2 m_\ell ^2 C_{10}^\mathrm{SM} \sqrt{1 - \frac{4m_\ell ^2}{m_{B_q}^2}},\nonumber \\ \end{aligned}$$

where the light-quark $q=s$ or *d*, and the coefficient $C_{10}^\mathrm{SM}$ includes the NLO electroweak and NNLO QCD matching corrections [453]. The factor $1/(1+y_q)$, with $y_q=\Delta \Gamma _{B_q}/(2 \Gamma _{B_q})$, accounts for the fact that the measured branching fraction corresponds to a time-integrated rate of the oscillating $B_q$ system to $\ell ^+\ell ^-$ [454]. That correction is particularly important for the $B_s$ decays because of the relatively large $y_s=0.06(1)$ [197, 455]. Evidence for both $B_s \rightarrow \mu ^+ \mu ^-$ and $B_s \rightarrow \mu ^+ \mu ^-$ decays was recently observed by the CMS and the LHCb Collaborations. Combining the data from both collaborations, the branching fractions can be extracted to be [450],

149 $$\begin{aligned} B(B_d \rightarrow \mu ^+ \mu ^-)= & {} (3.9^{+1.6}_{-1.4}) \,10^{-10} , \nonumber \\ B(B_s \rightarrow \mu ^+ \mu ^-)= & {} (2.8^{+0.7}_{-0.6}) \,10^{-9} , \end{aligned}$$

which are compatible with the Standard Model predictions at the $2.2\sigma$ and $1.2\sigma$ level, respectively.

The decay constants $f_{B_q}$ (with $q=u,d,s$) parameterize the matrix elements of the corresponding axial-vector currents, $A^{\mu }_{bq} = \bar{b}\gamma ^{\mu }\gamma ^5q$, analogously to the definition of $f_{D_q}$ in Sect. 7.1:

150 $$\begin{aligned} \langle 0| A^{\mu } | B_q(p) \rangle = i p_B^{\mu } f_{B_q}. \end{aligned}$$

For heavy–light mesons, it is convenient to define and analyse the quantity

151 $$\begin{aligned} \Phi _{B_q} \equiv f_{B_q} \sqrt{m_{B_q}}, \end{aligned}$$

which approaches a constant (up to logarithmic corrections) in the $m_B \rightarrow \infty$ limit according to HQET. In the following discussion we denote lattice data for $\Phi$(*f*) obtained at a heavy-quark mass $m_h$ and light valence-quark mass $m_{\ell }$ as $\Phi _{h\ell }$($f_{hl}$), to differentiate them from the corresponding quantities at the physical *b* and light-quark masses.

The *SU*(3)-breaking ratio, $f_{B_s}/f_B$, is of interest. This is because in lattice-QCD calculations for this quantity, many systematic effects can be partially reduced. These include discretization errors, heavy-quark mass tuning effects, and renormalization/matching errors, amongst others. On the other hand, this *SU*(3)-breaking ratio is still sensitive to the chiral extrapolation. Given that the chiral extrapolation is under control, one can then adopt $f_{B_s}/f_B$ as input in extracting phenomenologically interesting quantities. For instance, this ratio can be used to determine $|V_{ts}/V_{td}|$. In addition, it often happens to be easier to obtain lattice results for $f_{B_{s}}$ with smaller errors. Therefore, one can combine the $B_{s}$-meson decay constant with the *SU*(3)-breaking ratio to calculate $f_{B}$. Such strategy can lead to better precision in the computation of the *B*-meson-decay constant, and has been adopted by the ETM [20] and the HPQCD Collaborations [55].

It is clear that the decay constants for charged and neutral *B* mesons play different roles in flavour physics phenomenology. As already mentioned above, the knowledge of the $B^{+}$-meson decay constant, $f_{B^{+}}$, is essential for extracting $|V_{ub}|$ from leptonic $B^{+}$ decays. The neutral *B*-meson-decay constants, $f_{B^{0}}$ and $f_{B_{s}}$, are inputs for obtaining $|V_{td}|$ using information from the *B*-meson mixing processes. In view of this, it is desirable to include isospin-breaking effects in lattice computations for these quantities, and have results for $f_{B^{+}}$ and $f_{B^{0}}$. Nevertheless, as will be discussed in detail in this section, such effects are small compared to the current errors of the decay constants calculated using lattice QCD. In this review, we will then concentrate on the isospin-averaged result, $f_{B}$, and the $B_{s}$-meson-decay constant, as well as the *SU*(3)-breaking ratio, $f_{B_{s}}/f_{B}$. For the world average for the lattice determination of $f_{B^{+}}$ and $f_{B_{s}}/f_{B^{+}}$, we refer the reader to the latest work from the Particle Data Group (PDG) [184]. Notice that the lattice results used in Ref. [184] and the current review are identical. We will discuss this in further detail at the end of this subsection.

**Table 32:** Decay constants of the *B*, $B^+$, $B^0$ and $B_{s}$ mesons (in MeV). Here $f_B$ stands for the mean value of $f_{B^+}$ and $f_{B^0}$, extrapolated (or interpolated) in the mass of the light valence-quark to the physical value of $m_{ud}$

Collaboration	Refs.	$N_{ f}$	Publication status	Continuum extrapolation	Chiral extrapolation	Finite volume	Renormalization/matching	Heavy-quark treatment	$f_{B^+}$	$f_{B^0}$	$f_{B}$	$f_{B_s}$
ETM 13E	[456]	$2+1+1$	C						−	−	196(9)	235(9)
HPQCD 13	[52]	$2+1+1$	A						184(4)	188(4)	186(4)	224(5)
RBC/UKQCD 14	[53]	$2+1$	A						195.6(14.9)	199.5(12.6)	−	235.4(12.2)
RBC/UKQCD 14A	[54]	$2+1$	A						−	−	219(31)	264(37)
RBC/UKQCD 13A	[457]	$2+1$	C						−	−	191(6)$_\mathrm{stat}{}^{\mathrm{a}}$	233(5)$_\mathrm{stat}{}^{\mathrm{a}}$
HPQCD 12	[55]	$2+1$	A						−	−	191(9)	228(10)
HPQCD 12	[55]	$2+1$	A						−	−	189(4)$^{\mathrm{b}}$	−
HPQCD 11A	[56]	$2+1$	A						−	−	−	225(4)$^{\mathrm{c}}$
FNAL/MILC 11	[48]	$2+1$	A						197(9)	−	−	242(10)
HPQCD 09	[59]	$2+1$	A						−	−	190(13)$^{\mathrm{d}}$	231(15)$^{\mathrm{d}}$
ALPHA 14	[57]	2	A						−	−	186(13)	224(14)
ALPHA 13	[458]	2	C						−	−	187(12)(2)	224(13)
ETM 13B, 13C$^{\mathrm{e}}$	[20, 58]	2	A						−	−	189(8)	228(8)
ALPHA 12A	[459]	2	C						−	−	193(9)(4)	219(12)
ETM 12B	[460]	2	C						−	−	197(10)	234(6)
ALPHA 11	[461]	2	C						−	−	174(11)(2)	−
ETM 11A	[182]	2	A						−	−	195(12)	232(10)
ETM 09D	[462]	2	A						−	−	194(16)	235(12)

$^{\mathrm{a}}$ Statistical errors only

$^{\mathrm{b}}$ Obtained by combining $f_{B_s}$ from HPQCD 11A with $f_{B_s}/f_B$ calculated in this work

$^{\mathrm{c}}$ This result uses one ensemble per lattice spacing with light to strange sea-quark mass ratio $m_{\ell }/m_s \approx 0.2$

$^{\mathrm{d}}$ This result uses an old determination of $r_1=0.321(5)$ fm from Ref. [463] that has since been superseded

$^{\mathrm{e}}$ Update of ETM 11A and 12B

**Fig. 20:**
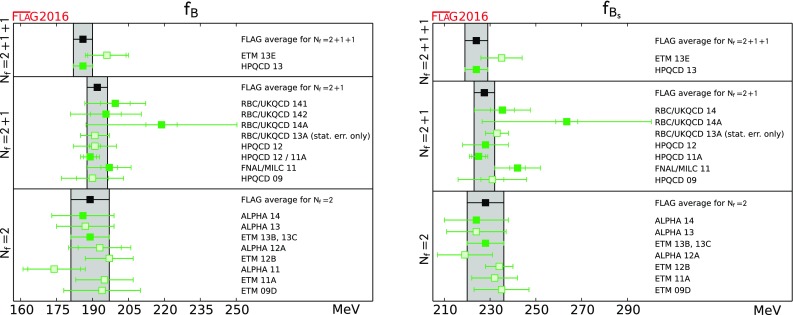
Decay constants of the *B* and $B_s$ mesons. The values are taken from Table 32 (the $f_B$ entry for FNAL/MILC 11 represents $f_{B^+}$). The *significance of the colours* is explained in Sect. 2. The *black squares* and *grey bands* indicate our averages in Eqs. (152), (153) and (154)

**Fig. 21:**
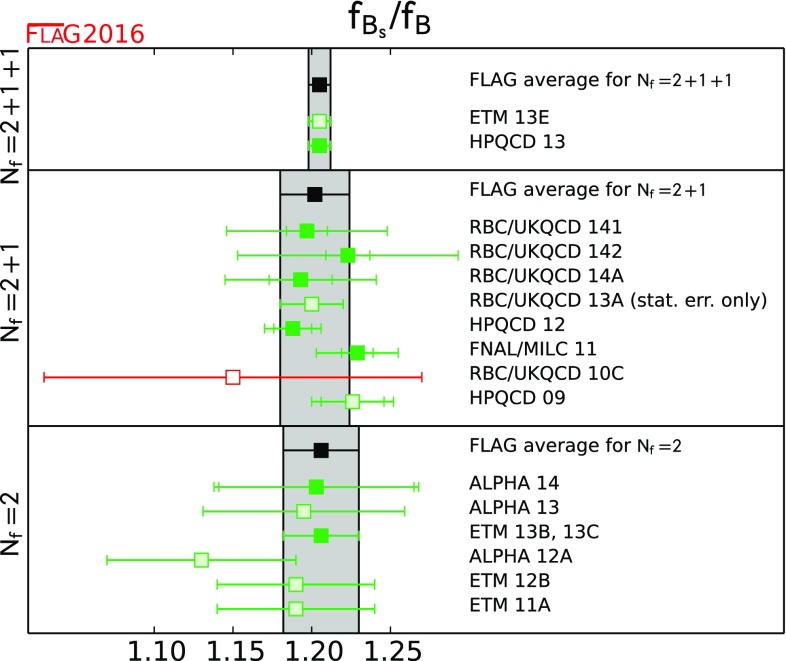
Ratio of the decay constants of the *B* and $B_s$ mesons. The values are taken from Table 33 (the $f_B$ entry for FNAL/MILC 11 represents $f_{B^+}$). The *significance of the colours* is explained in Sect. 2. The *black squares* and *grey bands* indicate our averages in Eqs. (152), (153) and (154)

The status of lattice-QCD computations for *B*-meson-decay constants and the *SU*(3)-breaking ratio, using gauge-field ensembles with light dynamical fermions, is summarized in Tables 32 and 33. Figures 20 and 21 contain the graphic presentation of the collected results and our averages. Many results in these tables and plots were already reviewed in detail in the previous FLAG report [2]. Below we will describe the new results that appeared after December 2013. In addition, we will comment on our updated strategies in performing the averaging.

**Table 33:** Ratios of decay constants of the *B* and $B_s$ mesons (for details see Table 32)

Collaboration	Refs.	$N_{ f}$	Publication status	Continuum extrapolation	Chiral extrapolation	Finite volume	Renormalization/matching	Heavy-quark treatment	$f_{B_s}/f_{B^+}$	$f_{B_s}/f_{B^0}$	$f_{B_s}/f_{B}$
ETM 13E	[456]	$2+1+1$	C						−	−	1.201(25)
HPQCD 13	[52]	$2+1+1$	A						1.217(8)	1.194(7)	1.205(7)
RBC/UKQCD 14	[53]	$2+1$	A						1.223(71)	1.197(50)	−
RBC/UKQCD 14A	[54]	$2+1$	A						−	−	1.193(48)
RBC/UKQCD 13A	[457]	$2+1$	C						−	−	1.20(2)$_\mathrm{stat}{}^{\mathrm{a}}$
HPQCD 12	[55]	$2+1$	A						−	−	1.188(18)
FNAL/MILC 11	[48]	$2+1$	A						1.229(26)	−	−
RBC/UKQCD 10C	[464]	$2+1$	A						−	−	1.15(12)
HPQCD 09	[59]	$2+1$	A						−	−	1.226(26)
ALPHA 14	[57]	2	A						−	−	1.203(65)
ALPHA 13	[458]	2	C						−	−	1.195(61)(20)
ETM 13B, 13C$^{\mathrm{b}}$	[20, 58]	2	A						−	−	1.206(24)
ALPHA 12A	[459]	2	C						−	−	1.13(6)
ETM 12B	[460]	2	C						−	−	1.19(5)
ETM 11A	[182]	2	A						−	−	1.19(5)

$^{\mathrm{a}}$ Statistical errors only

$^{\mathrm{b}}$ Update of ETM 11A and 12B

Only one new $N_{f}=2$ project for computing $f_{B}$, $f_{B_{s}}$ and $f_{B_{s}}/f_{B}$ was completed after the publication of the previous FLAG review. This was carried out by the ALPHA Collaboration [57] (ALPHA 14 in Tables 32 and 33), on the CLS (Coordinated Lattice Simulations) gauge-field ensembles which were generated using the Wilson plaquette action and $N_{f} = 2$ nonperturbatively $\mathcal {O}(a)$-improved Wilson fermions with the DD-HMC [465–467] or the MP-HMC [468] algorithm. There are three choices of lattice spacing, 0.048, 0.065 and 0.075 fm, in these ensembles. At each lattice spacing, three to four lattice sizes are adopted in the simulations. The hyper-cubic boxes are of the shape $L^{3}\times T$, with the temporal extent $T=2L$. The smallest box used in ALPHA 14 is $L \approx 2$ fm. On each of these lattice sizes, one sea-quark mass is employed in the computation, and the condition $M_{\pi } L > 4$ is always ensured. This leads to subpercentage-level finite-size effects [469]. The corresponding lightest pions composed of the sea quarks for these three values of the lattice spacing are 270, 190, and 280 MeV, respectively. In this work, the lattice-regularized HQET action and the axial current to the order of $1/m_{B}$, as tuned in Refs. [21, 470–473] with nonperturbative matching to QCD, are used to compute the heavy–light meson-decay constant. This matching procedure removes both the logarithmic and the power divergences in the effective theory regularized on the lattice. The valence light (up and down) quarks are implemented with the unitary setup, such that the valence and the sea pions have identical masses. On the other hand, the valence strange-quark mass is tuned on the CLS gauge-field ensembles employing the kaon decay constant [12]. The static-light axial current in this work is also $\mathcal {O}(a)$-improved to one-loop order. Using the lattice data, the ground-state contributions to the relevant correlators are obtained through the method of the generalized eigenvalue problem (GEVP), as detailed in Ref. [474]. With this GEVP approach in ALPHA 14, the systematic errors arising from the excited-state contamination are typically less than one third of the statistical errors in the extracted decay constants. Combined chiral-continuum extrapolations, adopting the NLO HM$\chi$PT predictions, are then performed to determine the decay constants in the limit of physical-pion mass and vanishing lattice spacing. The errors of the final results in ALPHA 14 include statistical uncertainties, the discrepancy to the static-limit results, the effects of the lattice spacing, the uncertainties from the HQET parameters in the matching procedure, and the systematic effects in the chiral extrapolations as estimated by comparing with fits to formulae without the chiral logarithms. Since the fits to the predictions of finite-volume HM$\chi$PT [469] have not been implemented, systematic effects resulting from the finite-lattice size are not included in the analysis. Nevertheless, given that the condition $M_{\pi } L > 4$ is always satisfied in ALPHA 14, these effects should be at the subpercentage level according to the one-loop formulae in Ref. [469].

The new result, ALPHA 14, satisfies all our criteria for being included in the averaging process. Therefore, in the current edition of the FLAG report, two $N_{f}=2$ calculations for the *B*-meson-decay constants and the *SU*(3)-breaking ratio contribute to our averages. The other determination of these quantities (ETM 13B, 13C in Tables 32, 33) was already reviewed in detail in the previous FLAG publication. These two projects are based on completely different lattice simulations, and there is no correlation between the errors quoted in them. This gives our estimate,152Two groups of authors (RBC/UKQCD 14 [53] and RBC/UKQCD 14A [54] in Tables 32, 33) presented their $N_{f} = 2+1$ results for $f_{B}$, $f_{B_{s}}$ and $f_{B_{s}}/f_{B}$ after the publication of the previous FLAG report in 2013. Both groups belong to the RBC/UKQCD Collaboration. They use the same gauge-field ensembles generated by this collaboration, with the Iwasaki gauge action and domain-wall dynamical quarks [144], adopting the “RHMC II” algorithm [145]. Two values of the lattice spacing, 0.11 and 0.086 fm, are used in the simulations, with the corresponding lattice sizes being $24^{3}\times 64$ and $32^{3}\times 64$, respectively. This fixes the spatial size $L \approx 2.7$ fm in all the datasets. For the coarse lattice, two choices of the sea-quark masses, with $M_{\pi } \approx 328$ and 420 MeV, are implemented in the simulations. On the other hand, three values of the sea-quark masses ($M_{\pi } \approx 289$, 344, 394 MeV) are used on the fine lattice. This makes certain that $M_{\pi } L > 4$ is always satisfied. At each value of the lattice spacing, only one sea strange-quark mass is implemented, which is about $10\%$ higher than its physical value.

In RBC/UKQCD 14, the heavy quark is described by the relativistic lattice action proposed in Ref. [475]. The three parameters of this relativistic heavy-quark (RHQ) action are tuned nonperturbatively in Ref.  [476] by requiring that the spin-averaged $B_{s}$-meson mass, $\overline{M}_{B_{s}} = (M_{B_s} + 3M_{B^{*}_{s}} )/4$, and the hyperfine splitting, $\Delta _{M_{B_s}} = M_{B_{s}^{*}} - M_{B_s}$ equal the PDG values, and that the lattice rest and kinetic meson masses are equal. Statistical uncertainties in the tuned parameters are propagated to the decay constants via jackknife resampling. Simulations with different values of the RHQ parameters are used to estimate the remaining uncertainties in the decay constants from the tuning procedure. Regarding valence light- and strange-quarks, the authors of RBC/UKQCD 14 adopt exactly the same domain-wall discretization as that in the sea-quark sector. For each lattice spacing, such valence domain-wall fermion propagators at six choices of the mass parameter are generated. These six values straddle between the lightest and strange sea-quark masses in the gauge-field ensembles, and several of them correspond to the unitary points. With the above lattice setting, the heavy-meson-decay constants are obtained, employing an axial current that is $\mathcal {O}(a)$-improved to one-loop level. The renormalization of the axial current is carried out with a mostly nonperturbative procedure proposed in Ref. [477]. Linear interpolations for the heavy-quark action parameters, as well as the valence strange-quark mass are then performed on these heavy-meson-decay constants. As for the chiral extrapolation for the light-quark mass, it is implemented together with the continuum extrapolation (linear in $a^{2}$) adopting *SU*(2)-HM$\chi$PT at NLO.^37^ The decay constants, $f_{B^{+}}$ and $f_{B^{0}}$, are determined by chirally extrapolating to the physical *u*- and *d*-quark masses, respectively, and their isospin-averaged counterpart, $f_{B}$, is not reported. Notice that only the unitary points in the light-quark mass are used in the central procedure for the chiral extrapolation. This extrapolation serves as the method to confirm that finite-size effects are at the subpercentage level by comparing with the prediction of finite-volume HM$\chi$PT [469]. Furthermore, since there is no observed sea-quark dependence in $f_{B_{s}}$, it is extrapolated to the continuum limit straight after the interpolation of the valence strange-quark mass. The authors of RBC/UKQCD 14 provided a comprehensive list of systematic errors in their work. The dominant effect is from the chiral-continuum extrapolation. This was investigated using several alternative procedures by varying the fit ansätze and omitting the data points at the heaviest pion mass. The error arising from the continuum extrapolation of $f_{B_{s}}$ is estimated by taking the result on the finer lattice as the alternative. One other important source of the systematic errors is the heavy-quark discretization effect, which is estimated using a power-counting argument in the improvement programme.

In the other newly completed *B*-meson-decay constants project, RBC/UKQCD 14A, the static heavy-quark action is implemented with the HYP smearing [478] that reduces the power divergences. As for the valence light- and strange-quarks, the same domain-wall discretization as adopted for the sea quarks is used. The masses of the valence light quarks are chosen to be at the unitary points. On the other hand, for each lattice spacing, two values of the valence strange-quark mass are utilized, with one of them identical to that of its sea-quark counterpart, and the other slightly smaller than the physical strange-quark mass. Employing the propagators of these valence quarks computed on the RBC/UKQCD gauge-field ensembles, the relevant matrix elements of the axial current are calculated to extract the decay constant. Notice that the source and sink smearings are applied on the valence light- and strange-quark propagators, in order to obtain better overlap with the ground state. The axial current is $\mathcal {O}(a)$-improved to one-loop order, and its renormalization/matching is performed in a two-step fashion. Namely, it is first matched from the lattice-regularized HQET to the same effective theory in the continuum at the inverse lattice spacing, $a^{-1}$, and then matched to QCD at the physical *b*-quark mass, $m_{b}$. At each of these two steps, the matching is carried out at one-loop level, and the two-loop running between $a^{-1}$ and $m_{b}$ is implemented accordingly. Regarding the extrapolation to the physical light-quark mass, it is achieved using *SU*(2)-HM$\chi$PT, after linearly interpolating the decay constants to the physical strange-quark mass in the valence sector. Unlike RBC/UKQCD 14, here the isospin-averaged $f_{B}$, instead of the individual $f_{B^{+}}$ and $f_{B^{0}}$, is reported in RBC/UKQCD 14A. This chiral fit is combined with the continuum extrapolation by including a term proportional to $a^{2}$ in the HM$\chi$PT formulae. In addition, finite-size effects are also estimated by replacing the one-loop integrals with sums in HM$\chi$PT [469]. The predominant systematic error in $f_{B_{s}}$ and $f_{B}$ is from the one-loop renormalization/matching procedure. This error is accounted for by employing a power-counting method, and is evaluated to be around $6\%$. Obviously, it is small for $f_{B_{s}}/f_{B}$. Another significant systematic effect (about $2{-}3\%$ in all relevant quantities) results from the chiral-continuum extrapolation. This effect is estimated by omitting the chiral logarithms in the fitting procedure. Finally, based upon a power-counting argument, the authors of RBC/UKQCD 14A include a $10\%$ error on $f_{B_{(s)}}$, and a $2.2\%$ error on $f_{B_{s}}/f_{B}$, to account for the use of the static heavy quarks in their work.

Both new computations from the RBC/UKQCD Collaboration satisfy the criteria for being considered in our averages of the relevant quantities. Since they are based on exactly the same gauge-field configurations, we treat the statistical errors in these two results as $100\%$ correlated. It also has to be pointed out that only $f_{B^{+}}$ and $f_{B^{0}}$ are reported in RBC/UKQCD 14, while we are concentrating on the isospin-averaged $f_{B}$ in our current work. For this purpose, we regard both $f_{B^{+}}$ and $f_{B^{0}}$ in RBC/UKQCD 14 as $f_{B}$, and completely correlate all the errors.

In addition to RBC/UKQCD 14 and RBC/UKQCD 14A, a few other results in Tables 32 and 33 are also in our averaging procedure. These include HPQCD 12, HPQCD 11A, and FNAL/MILC 11. Notice that there are two results of $f_{B}$ from HPQCD 12 in Table 32. Both of these were in the averaging procedure in the last edition of the FLAG report. However, for our current work, we only include the one with smaller error. This result is obtained by taking $f_{B_{s}}/f_{B}$ computed with the NRQCD description of the *b* quark in HPQCD 12, and multiplying it by $f_{B_{s}}$ calculated employing the HISQ discretization for the heavy quarks in HPQCD 11A. This strategy significantly reduces the systematic effect arising from the renormalization of the axial current in Eq. (150), as compared to the “direct” determination of $f_{B}$ using NRQCD heavy quarks in HPQCD 12. Since the calculations performed in FNAL/MILC 11, HPQCD 12 and HPQCD 11A all involve the gauge-field ensembles generated by the MILC Collaboration, we treat their statistical errors as $100\%$ correlated. Following the above discussion, our procedure leads to the averages,

153 $$\begin{aligned} f_B=192.0(4.3)\;\mathrm{MeV} \quad \,\mathrm {Refs.}~ [48, 53{-}56],\nonumber \\ N_f=2+1:\quad f_{B_s}=228.4(3.7)\,\;\mathrm{MeV}\quad \,\mathrm {Refs.}~ [48, 53{-}56], \nonumber \\ {{f_{B_s}}/{f_B}}=1.201(16)\,\quad \,\mathrm {Refs.}~ [48, 53{-}55].\nonumber \\ \end{aligned}$$

There have been no new $N_{f} = 2+1+1$ results for the *B*-meson decay constants and the *SU*(3)-breaking ratio since the release of the previous FLAG publication.^38^ Therefore, our averages remain the same as those in the previous FLAG report,154The PDG recently presented their averages for the $N_{f}=2+1$ and $N_{f}=2+1+1$ lattice-QCD determinations of $f_{B^{+}}$, $f_{B_{s}}$ and $f_{B_{s}}/f_{B^{+}}$ [184].^39^ The lattice-computation results used in Ref. [184] are identical to those included in our current work. Regarding our isospin-averaged $f_{B}$ as the representative for $f_{B^{+}}$, then the results from current FLAG and PDG estimations for these quantities are well compatible. In the PDG work, they “corrected” the isospin-averaged $f_{B}$, as reported by various lattice collaborations, using the $N_{f}=2+1+1$ strong isospin-breaking effect computed in HPQCD 13 [52] (see Table 32 in this subsection). This only accounts for the contribution from the valence-quark masses. However, since the isospin-breaking effects from the sea-quark masses appear in the form $(m^{(\mathrm{sea})}_{u} - m^{(\mathrm{sea})}_{d})^{2}$, the valence sector is the predominant source of strong isospin breaking [480].^40^

### Neutral *B*-meson mixing matrix elements

Neutral *B*-meson mixing is induced in the Standard Model through one-loop box diagrams to lowest order in the electroweak theory, similar to those for short-distance effects in neutral kaon mixing. The effective Hamiltonian is given by

155 $$\begin{aligned} \mathcal{H}_\mathrm{eff}^{\Delta B = 2, \mathrm{SM}}=\frac{G_F^2 M_\mathrm{{W}}^2}{16\pi ^2} (\mathcal{F}^0_d \mathcal{Q}^d_1 + \mathcal{F}^0_s \mathcal{Q}^s_1)+\mathrm{h.c.}, \end{aligned}$$

with

156 $$\begin{aligned} \mathcal{Q}^q_1 = \left[ \bar{b}\gamma _\mu (1-\gamma _5)q\right] \left[ \bar{b}\gamma _\mu (1-\gamma _5)q\right] , \end{aligned}$$

where $q=d$ or *s*. The short-distance function $\mathcal{F}^0_q$ in Eq. (155) is much simpler compared to the kaon mixing case due to the hierarchy in the CKM matrix elements. Here, only one term is relevant,

157 $$\begin{aligned} \mathcal{F}^0_q = \lambda _{tq}^2 S_0(x_t) \end{aligned}$$

where

158 $$\begin{aligned} \lambda _{tq} = V^*_{tq}V_{tb}, \end{aligned}$$

and where $S_0(x_t)$ is an Inami–Lim function with $x_t=m_t^2/M_W^2$, which describes the basic electroweak loop contributions without QCD [381]. The transition amplitude for $B_q^0$ with $q=d$ or *s* can be written as

159 $$\begin{aligned}&\langle \bar{B}^0_q \vert \mathcal{H}_\mathrm{eff}^{\Delta B = 2} \vert B^0_q\rangle =\frac{G_F^2 M_\mathrm{{W}}^2}{16 \pi ^2} [\lambda _{tq}^2 S_0(x_t) \eta _{2B}] \nonumber \\&\quad \times \left( \frac{\bar{g}(\mu )^2}{4\pi }\right) ^{-\gamma _0/(2\beta _0)} \exp \left\{ \int _0^{\bar{g}(\mu )} \, \mathrm{d}g \, \left( \frac{\gamma (g)}{\beta (g)}+ \frac{\gamma _0}{\beta _0g} \right) \right\} \nonumber \\&\quad \times \langle \bar{B}^0_q \vert Q^q_\mathrm{R} (\mu ) \vert B^0_q \rangle +\mathrm{h.c.}, \end{aligned}$$

where $Q^q_\mathrm{R} (\mu )$ is the renormalized four-fermion operator (usually in the NDR scheme of ${\overline{\text {MS}}}$). The running coupling ($\bar{g}$), the $\beta$-function ($\beta (g)$), and the anomalous dimension of the four-quark operator ($\gamma (g)$) are defined in Eqs. (104) and (105). The product of $\mu$ dependent terms on the second line of Eq. (159) is, of course, $\mu$-independent (up to truncation errors arising from the use of perturbation theory). The explicit expression for the short-distance QCD correction factor $\eta _{2B}$ (calculated to NLO) can be found in Ref. [379].

For historical reasons the *B*-meson mixing matrix elements are often parameterized in terms of bag parameters defined as

160 $$\begin{aligned} B_{B_q}(\mu )= \frac{{\left\langle \bar{B}^0_q\left| Q^q_\mathrm{R}(\mu )\right| B^0_q\right\rangle } }{ {\frac{8}{3}f_{B_q}^2m_\mathrm {B}^2}}. \end{aligned}$$

The RGI *B* parameter $\hat{B}$ is defined, as in the case of the kaon, and expressed to two-loop order as

161 $$\begin{aligned} \hat{B}_{B_q}= & {} \left( \frac{\bar{g}(\mu )^2}{4\pi }\right) ^{- \gamma _0/(2\beta _0)}\nonumber \\&\times \left\{ 1+\dfrac{\bar{g}(\mu )^2}{(4\pi )^2}\left[ \frac{\beta _1\gamma _0-\beta _0\gamma _1}{2\beta _0^2} \right] \right\} \, B_{B_q}(\mu ), \end{aligned}$$

with $\beta _0$, $\beta _1$, $\gamma _0$, and $\gamma _1$ defined in Eq. (106). Note, as Eq. (159) is evaluated above the bottom threshold ($m_b<\mu <m_t$), the active number of flavours here is $N_f=5$.

Nonzero transition amplitudes result in a mass difference between the *CP* eigenstates of the neutral *B*-meson system. Writing the mass difference for a $B_q^0$ meson as $\Delta m_q$, its Standard Model prediction is

162 $$\begin{aligned} \Delta m_q = \frac{G^2_Fm^2_W m_{B_q}}{6\pi ^2} \, |\lambda _{tq}|^2 S_0(x_t) \eta _{2B} f_{B_q}^2 \hat{B}_{B_q}. \end{aligned}$$

Experimentally the mass difference is measured as oscillation frequency of the *CP* eigenstates. The frequencies are measured precisely with an error of less than a percent. Many different experiments have measured $\Delta m_d$, but the current average [151] is based on measurements from the *B*-factory experiments Belle and Babar, and from the LHC experiment LHC*b*. For $\Delta m_s$ the experimental average is dominated by results from LHC*b* [151]. With these experimental results and lattice-QCD calculations of $f_{B_q}^2\hat{B}_{B_q}$ at hand, $\lambda _{tq}$ can be determined. In lattice-QCD calculations the flavour *SU*(3)-breaking ratio

163 $$\begin{aligned} \xi ^2 = \frac{f_{B_s}^2B_{B_s}}{f_{B_d}^2B_{B_d}} \end{aligned}$$

can be obtained more precisely than the individual $B_q$-mixing matrix elements because statistical and systematic errors cancel in part. With this the ratio $|V_{td}/V_{ts}|$ can be determined, which can be used to constrain the apex of the CKM triangle.

Neutral *B*-meson mixing, being loop-induced in the Standard Model is also a sensitive probe of new physics. The most general $\Delta B=2$ effective Hamiltonian that describes contributions to *B*-meson mixing in the Standard Model and beyond is given in terms of five local four-fermion operators:

164 $$\begin{aligned} \mathcal{H}_\mathrm{eff, BSM}^{\Delta B = 2} = \sum _{q=d,s}\sum _{i=1}^5 \mathcal{C}_i \mathcal{Q}^q_i, \end{aligned}$$

where $\mathcal{Q}_1$ is defined in Eq. (156) and where

165 $$\begin{aligned}&\mathcal{Q}^q_2 = \left[ \bar{b}(1-\gamma _5)q\right] \left[ \bar{b}(1-\gamma _5)q\right] ,\nonumber \\&\mathcal{Q}^q_3 = \left[ \bar{b}^{\alpha }(1-\gamma _5)q^{\beta }\right] \left[ \bar{b}^{\beta }(1-\gamma _5)q^{\alpha }\right] ,\nonumber \\&\mathcal{Q}^q_4 = \left[ \bar{b}(1-\gamma _5)q\right] \left[ \bar{b}(1+\gamma _5)q\right] , \nonumber \\&\mathcal{Q}^q_5 = \left[ \bar{b}^{\alpha }(1-\gamma _5)q^{\beta }\right] \left[ \bar{b}^{\beta }(1+\gamma _5)q^{\alpha }\right] , \end{aligned}$$

with the superscripts $\alpha ,\beta$ denoting colour indices, which are shown only when they are contracted across the two bilinears. There are three other basis operators in the $\Delta B=2$ effective Hamiltonian. When evaluated in QCD, however, they give identical matrix elements to the ones already listed due to parity invariance in QCD. The short-distance Wilson coefficients $\mathcal{C}_i$ depend on the underlying theory and can be calculated perturbatively. In the Standard Model only matrix elements of $\mathcal{Q}^q_1$ contribute to $\Delta m_q$, while all operators do for example for general SUSY extensions of the Standard Model [411]. The matrix elements or bag parameters for the non-SM operators are also useful to estimate the width difference in the Standard Model, where combinations of matrix elements of $\mathcal{Q}^q_1$, $\mathcal{Q}^q_2$, and $\mathcal{Q}^q_3$ contribute to $\Delta \Gamma _q$ at $\mathcal {O}(1/m_b)$ [481, 482].

**Table 34:** Neutral *B*- and $B_\mathrm{s}$-meson mixing matrix elements (in MeV) and bag parameters

Collaboration	Refs.	$N_{ f}$	Publication status	Continuum extrapolation	Chiral extrapolation	Finite volume	Renormalization/matching	Heavy-quark treatment	$f_\mathrm{B_d}\sqrt{\hat{B}_\mathrm{B_d}}$	$f_\mathrm{B_s}\sqrt{\hat{B}_\mathrm{B_s}}$	$\hat{B}_\mathrm{B_d}$	$\hat{B}_\mathrm{B_\mathrm{s}}$
RBC/UKQCD 14A	[54]	$2+1$	A						240(15)(33)	290(09)(40)	1.17(11)(24)	1.22(06)(19)
FNAL/MILC 11A	[483]	$2+1$	C						250(23)$^{\mathrm{a}}$	291(18)$^{\mathrm{a}}$	−	−
HPQCD 09	[59]	$2+1$	A		$^\mathrm{b}$				216(15)$^{\mathrm{c}}$	266(18)$^{\mathrm{c}}$	1.27(10)$^{\mathrm{c}}$	1.33(6)$^{\mathrm{c}}$
HPQCD 06A	[484]	$2+1$	A						−	281(21)	−	1.17(17)
ETM 13B	[20]	2	A						216(6)(8)	262(6)(8)	1.30(5)(3)	1.32(5)(2)
ETM 12A, 12B	[460, 485]	2	C						−	−	1.32(8)$^{\mathrm{d}}$	1.36(8)$^{\mathrm{d}}$

$^{\mathrm{a}}$ Reported $f_B^2B$ at $\mu =m_b$ is converted to RGI by multiplying the two-loop factor 1.517

$^{\mathrm{b}}$ Wrong-spin contributions are not included in the rS$\chi$PT fits

$^{\mathrm{c}}$ This result uses an old determination of $r_1=0.321(5)$ fm from Ref. [463] that has since been superseded

$^{\mathrm{d}}$ Reported *B* at $\mu =m_b=4.35$ GeV is converted to RGI by multiplying the two-loop factor 1.521

**Table 35:** Results for *SU*(3)-breaking ratios of neutral $B_{d}$- and $B_{s}$-meson mixing matrix elements and bag parameters

Collaboration	Refs.	$N_{ f}$	Publication status	Continuum extrapolation	Chiral extrapolation	Finite volume	Renormalization/matching	Heavy-quark treatment	$\xi$	$B_\mathrm{B_\mathrm{s}}/B_\mathrm{B_d}$
RBC/UKQCD 14A	[54]	$2+1$	A						1.208(41)(52)	1.028(60)(49)
FNAL/MILC 12	[60]	$2+1$	A						1.268(63)	1.06(11)
RBC/UKQCD 10C	[464]	$2+1$	A						1.13(12)	−
HPQCD 09	[59]	$2+1$	A		$^{\mathrm{a}}$				1.258(33)	1.05(7)
ETM 13B	[20]	2	A						1.225(16)(14)(22)	1.007(15)(14)
ETM 12A, 12B	[460, 485]	2	C						1.21(6)	1.03(2)

$^{\mathrm{a}}$ Wrong-spin contributions are not included in the rS$\chi$PT fits

**Fig. 22:**
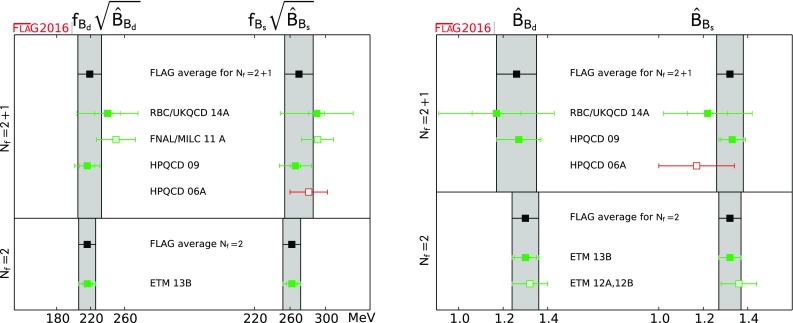
Neutral *B*- and $B_\mathrm{s}$-meson mixing matrix elements and bag parameters [values in Table 34 and Eqs. (166), (167), (169), (170)]

**Fig. 23:**
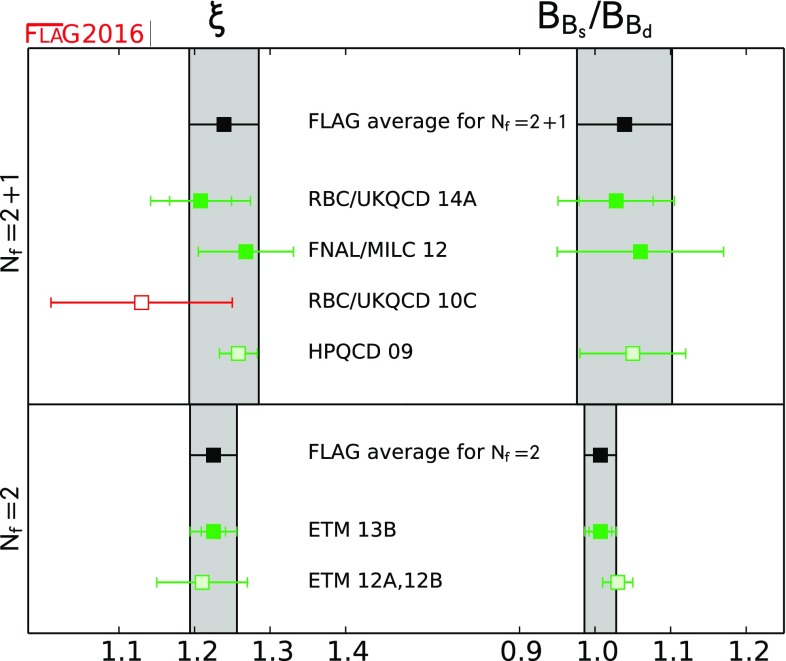
The *SU*(3)-breaking quantities $\xi$ and $B_{B_s}/B_{B_d}$ [values in Table 35 and Eqs. (168) and (171)]

In this section we report on results from lattice-QCD calculations for the neutral *B*-meson mixing parameters $\hat{B}_{B_d}$, $\hat{B}_{B_s}$, $f_{B_d}\sqrt{\hat{B}_{B_d}}$, $f_{B_s}\sqrt{\hat{B}_{B_s}}$ and the *SU*(3)-breaking ratios $B_{B_s}/B_{B_d}$ and $\xi$ defined in Eqs. (160), (161), and (163). The results are summarized in Tables 34 and 35 and in Figs. 22 and 23. Additional details about the underlying simulations and systematic error estimates are given in Appendix B.6.2. Some collaborations do not provide the RGI quantities $\hat{B}_{B_q}$ but quote instead $B_B(\mu )^{\overline{MS},NDR}$. In such cases we convert the results to the RGI quantities quoted in Table 34 using Eq. (161). More details of the conversion factors are provided below in the descriptions of the individual results. We do not provide the *B*-meson matrix elements of the other operators $\mathcal{Q}_{2-5}$ in this report. They have been calculated in Ref. [20] for the $N_f=2$ case and in Ref. [483], which is a conference proceedings article.

There are no new results for $N_f=2$ reported after the previous FLAG review. However, the paper by the ETM Collaboration (ETM 13B) [20], which was a preprint, has been published in a journal, thus, it is now eligible to enter the averages. Because this is the only result that passes the quality criteria for $N_f=2$, we quote their values as our averages in this version:

166 $$\begin{aligned}&f_{B_d}\sqrt{\hat{B}_{b_d}}= 216(10)~\mathrm{MeV},\quad f_{B_s}\sqrt{\hat{B}_{B_s}}= 262(10)~\mathrm{MeV}\nonumber \\&\quad \,\mathrm {Ref.}~[20], \end{aligned}$$

167 $$\begin{aligned}&N_f=2:\hat{B}_{B_d}= 1.30(6)\,, \hat{B}_{B_s}= 1.32(5)\nonumber \\&\quad \,\mathrm {Ref.}~[20], \end{aligned}$$

168 $$\begin{aligned}&\xi = 1.225(31)\,, B_{B_s}/B_{B_d} = 1.007(21)\nonumber \\&\quad \,\mathrm {Ref.}~[20]. \end{aligned}$$

For the $N_f=2+1$ case there is a new report (RBC/UKQCD 14A) [54] by the RBC/UKQCD Collaboration on the neutral *B*-meson mixing parameter, using domain-wall fermions for the light quarks and the static approximation for the *b* quark. Used gauge configuration ensembles are the $N_f=2+1$ domain-wall fermion and Iwasaki gauge actions with two lattice spacings ($a\approx 0.09, 0.11$ fm) and a minimum pion mass of about 290 MeV. Two different static-quark actions, smeared with HYP1 [478] and HYP2 [486] are used to further constrain the continuum limit. The operators used are one-loop $\mathcal {O}(a)$-improved with the tadpole improved perturbation theory. Two different types of chiral formulae are adopted for the combined continuum and chiral extrapolation: *SU*(2) NLO HM$\chi$PT and first order polynomial in quark masses with linear $\mathcal {O}(a^2)$ terms. The central values are determined as the average of the results with two different chiral formulae. The systematic error is estimated as half of the full difference of the two, with an exception for the quantity only involving $B_s^0$, where the NLO $\chi$PT is identical to the first order polynomial. In such cases, the fit excluding the heaviest *ud* mass point is used for the estimate of the systematic error. The systematic error due to the static approximation is estimated by the simple power counting: the size of $\Lambda _{QCD}/m_b$, where $\Lambda _{QCD}=0.5$ GeV and $m_b(\mu =m_b)^{\overline{\mathrm{MS}}}=4.18$ GeV (PDG) leads to 12%. This is the dominant systematic error for individual $f_B\sqrt{B_B}$ or $B_B$. Due to this large error, the effect of the inclusion in the FLAG averages of these quantities is small. The dominant systematic error for the *SU*(3)-breaking error, instead, comes from the combined continuum and chiral extrapolation, while the statistical uncertainty is a bit larger than that.

Due to the addition of this new result, the values for $N_f=2+1$ are now averages from multiple results by multiple collaborations, rather than being given by the values from a single computation, as it was done in the previous FLAG report. Our averages are:

169 $$\begin{aligned}&f_{B_d}\sqrt{\hat{B}_{B_d}} = 219(14)\, \mathrm{MeV}\, , f_{B_s}\sqrt{\hat{B}_{B_s}} = 270(16)\, \mathrm{MeV}\nonumber \\&\quad \,\mathrm {Refs.}~[54, 59], \end{aligned}$$

170 $$\begin{aligned}&N_f=2+1: \hat{B}_{B_d} = 1.26(9)\,, \hat{B}_{B_s} = 1.32(6)\nonumber \\&\quad \,\mathrm {Refs.}~[54, 59], \end{aligned}$$

171 $$\begin{aligned}&\xi = 1.239(46)\,, B_{B_s}/B_{B_d} = 1.039(63)\nonumber \\&\quad \,\mathrm {Refs.}~[54, 60]. \end{aligned}$$

Here Eqs. (169) and (170) are averages from HPQCD 09 [59] and RBC/UKQCD 14A [54], while Eq. (171) is from FNAL/MILC 12 [60] and RBC/UKQCD 14A [54].

Let us note that there has been a major update of these quantities from FNAL/MILC [487] with $N_f=2+1$ Asqtad MILC ensembles, extended towards physical *u*-*d* quark mass, continuum limit and increased statistics compared to the ones that entered this review (FNAL/MILC 12 [60], FNAL/MILC 11A [483]). This result could make significant improvements to the quantities for $N_f=2+1$. However, since the paper appeared after the closing date of this report, the results are not reviewed or taken into the average here due to the rule described in the Introduction and Sect. 2.2. The corresponding averages on our website http://itpwiki.unibe.ch/flag [3], will instead be updated soon in order to include the new FNAL/MILC results.

As discussed in detail in the previous FLAG review [2] HPQCD 09 does not include wrong-spin contributions, which are staggered fermion artefacts, to the chiral-extrapolation analysis. It is possible that the effect is significant for $\xi$ and $B_{B_s}/B_{B_d}$, since the chiral-extrapolation error is a dominant one for these *SU*(3) flavour breaking ratios. Indeed, a test done by FNAL/MILC 12 [60] indicates that the omission of the wrong-spin contribution in the chiral analysis may be a significant source of error. We therefore took the conservative choice to exclude $\xi$ and $B_{B_s}/B_{B_d}$ by HPQCD 09 from our average and we follow the same strategy in this report as well.

We note that the above results are all correlated with each other: the numbers in Eqs. (169) and (170) are dominated by those from HPQCD 09 [59], while those in Eq. (171) involve FNAL/MILC 12 [60] – the same Asqtad MILC ensembles are used in these simulations. The results are also correlated with the averages obtained in Sect. 8.1 and shown in Eq. (153), because the calculations of *B*-meson-decay constants and mixing quantities are performed on the same (or on similar) sets of ensembles, and results obtained by a given collaboration use the same actions and setups. These correlations must be considered when using our averages as inputs to UT fits. In the future, as more independent calculations enter the averages, correlations between the lattice-QCD inputs to the UT fit will become less significant.

### Semileptonic form factors for *B* decays to light flavours

The Standard Model differential rate for the decay $B_{(s)}\rightarrow P\ell \nu$ involving a quark-level $b\rightarrow u$ transition is given, at leading order in the weak interaction, by a formula identical to the one for *D* decays in Eq. (131) but with $D \rightarrow B_{(s)}$ and the relevant CKM matrix element $|V_{cq}| \rightarrow |V_{ub}|$:

172 $$\begin{aligned}&\frac{\mathrm{d}\Gamma (B_{(s)}\rightarrow P\ell \nu )}{\mathrm{d}q^2} = \frac{G_F^2 |V_{ub}|^2}{24 \pi ^3} \,\frac{(q^2-m_\ell ^2)^2\sqrt{E_P^2-m_P^2}}{q^4m_{B_{(s)}}^2}\nonumber \\&\quad \times \left[ \left( 1+\frac{m_\ell ^2}{2q^2}\right) m_{B_{(s)}}^2(E_P^2-m_P^2)|f_+(q^2)|^2\right. \nonumber \\&\quad +\left. \,\frac{3m_\ell ^2}{8q^2}(m_{B_{(s)}}^2-m_P^2)^2|f_0(q^2)|^2\right] . \end{aligned}$$

Again, for $\ell =e,\mu$ the contribution from the scalar form factor $f_0$ can be neglected, and one has a similar expression to Eq. (133), which in principle allows for a direct extraction of $|V_{ub}|$ by matching theoretical predictions to experimental data. However, while for *D* (or *K*) decays the entire physical range $0 \le q^2 \le q^2_\mathrm{max}$ can be covered with moderate momenta accessible to lattice simulations, in $B \rightarrow \pi \ell \nu$ decays one has $q^2_\mathrm{max} \sim 26~\mathrm{GeV}^2$ and only part of the full kinematic range is reachable. As a consequence, obtaining $|V_{ub}|$ from $B\rightarrow \pi \ell \nu$ is more complicated than obtaining $|V_{cd(s)}|$ from semileptonic *D*-meson decays.

In practice, lattice computations are restricted to small values of the momentum transfer (see Sect. 7.2) where statistical and momentum-dependent discretization errors can be controlled,^41^ which in existing calculations roughly cover the upper third of the kinematically allowed $q^2$ range. Since, on the other hand, the decay rate is suppressed by phase space at large $q^2$, most of the semileptonic $B\rightarrow \pi$ events are selected in experiment at lower values of $q^2$, leading to more accurate experimental results for the binned differential rate in that region.^42^ It is therefore a challenge to find a window of intermediate values of $q^2$ at which both the experimental and the lattice results can be reliably evaluated.

In current practice, the extraction of CKM matrix elements requires that both experimental and lattice data for the $q^2$ dependence be parameterized by fitting data to a specific ansatz. Before the generalization of the sophisticated ansätze that will be discussed below, the most common procedure to overcome this difficulty involved matching the theoretical prediction and the experimental result for the integrated decay rate over some finite interval in $q^2$,

173 $$\begin{aligned} \Delta \zeta = \frac{1}{|V_{ub}|^2} \int _{q^2_{1}}^{q^2_{2}} \left( \frac{\mathrm{d} \Gamma }{\mathrm{d} q^2} \right) \mathrm{d}q^2. \end{aligned}$$

In the most recent literature, it has become customary to perform a joint fit to lattice and experimental results, keeping the relative normalization $|V_{ub}|^2$ as a free parameter. In either case, good control of the systematic uncertainty induced by the choice of parameterization is crucial to obtain a precise determination of $|V_{ub}|$.

#### Parameterizations of semileptonic form factors

In this section, we discuss the description of the $q^2$ dependence of form factors, using the vector form factor $f_+$ of $B\rightarrow \pi \ell \nu$ decays as a benchmark case. Since in this channel the parameterization of the $q^2$ dependence is crucial for the extraction of $|V_{ub}|$ from the existing measurements (involving decays to light leptons), as explained above, it has been studied in great detail in the literature. Some comments about the generalization of the techniques involved will follow.

*The vector form factor for* $B\rightarrow \pi \ell \nu$ All form factors are analytic functions of $q^2$ outside physical poles and inelastic threshold branch points; in the case of $B\rightarrow \pi \ell \nu$, the only pole expected below the $B\pi$ production region, starting at $q^2 = t_+ = (m_B+m_\pi )^2$, is the $B^*$. A simple ansatz for the $q^2$ dependence of the $B\rightarrow \pi \ell \nu$ semileptonic form factors that incorporates vector-meson dominance is the Bećirević-Kaidalov (BK) parameterization [442], which for the vector form factor reads

174 $$\begin{aligned} f_+(q^2) = \frac{f(0)}{(1-q^2/m_{B^*}^2)(1-\alpha q^2/m_{B^*}^2)}. \end{aligned}$$

Because the BK ansatz has few free parameters, it has been used extensively to parameterize the shape of experimental branching-fraction measurements and theoretical form-factor calculations. A variant of this parameterization proposed by Ball and Zwicky (BZ) adds extra pole factors to the expressions in Eq. (174) in order to mimic the effect of multiparticle states [489]. A similar idea, extending the use of effective poles also to $D\rightarrow \pi \ell \nu$ decays, is explored in Ref. [490]. Finally, yet another variant (RH) has been proposed by Hill in Ref. [491]. Although all of these parameterizations capture some known properties of form factors, they do not manifestly satisfy others. For example, perturbative QCD scaling constrains the behaviour of $f_+$ in the deep Euclidean region [492–494], and angular momentum conservation constrains the asymptotic behaviour near thresholds – e.g., $\mathrm{Im}\,f_+(q^2) \sim (q^2-t_+)^{3/2}$ (see, e.g., Ref. [436]). Most importantly, these parameterizations do not allow for an easy quantification of systematic uncertainties.

A more systematic approach that improves upon the use of simple models for the $q^2$ behaviour exploits the positivity and analyticity properties of 2-point functions of vector currents to obtain optimal parameterizations of form factors [435, 494–498]. Any form factor *f* can be shown to admit a series expansion of the form

175 $$\begin{aligned} f(q^2) = \frac{1}{B(q^2)\phi (q^2,t_0)}\,\sum _{n=0}^\infty a_n(t_0)\,z(q^2,t_0)^n, \end{aligned}$$

where the squared momentum transfer is replaced by the variable

176 $$\begin{aligned} z(q^2,t_0) = \frac{\sqrt{t_+-q^2}-\sqrt{t_+-t_0}}{\sqrt{t_+-q^2}+\sqrt{t_+-t_0}}. \end{aligned}$$

This is a conformal transformation, depending on an arbitrary real parameter, $t_0<t_+$, that maps the $q^2$ plane cut for $q^2 \ge t_+$ onto the disk $|z(q^2,t_0)|<1$ in the *z* complex plane. The function $B(q^2)$ is called the *Blaschke factor*, and contains poles and cuts below $t_+$ – for instance, in the case of $B\rightarrow \pi$ decays,

177 $$\begin{aligned} B(q^2)=\frac{z(q^2,t_0)-z(m_{B^*}^2,t_0)}{1-z(q^2,t_0)z(m_{B^*}^2,t_0)}=z(q^2,m_{B^*}^2). \end{aligned}$$

Finally, the quantity $\phi (q^2,t_0)$, called the *outer function*, is some otherwise arbitrary function that does not introduce further poles or branch cuts. The crucial property of this series expansion is that the sum of the squares of the coefficients

178 $$\begin{aligned} \sum _{n=0}^\infty a_n^{2} = \frac{1}{2\pi i}\oint \frac{\mathrm{d}z}{z}\,|B(z)\phi (z)f(z)|^2, \end{aligned}$$

is a finite quantity. Therefore, by using this parameterization an absolute bound to the uncertainty induced by truncating the series can be obtained. The aim in choosing $\phi$ is to obtain a bound that is useful in practice, while (ideally) preserving the correct behaviour of the form factor at high $q^2$ and around thresholds.

The simplest form of the bound would correspond to $\sum _{n=0}^\infty a_n^{2}=1$. *Imposing* this bound yields the following “standard” choice for the outer function

179 $$\begin{aligned} \phi (q^2,t_0)= & {} \sqrt{\frac{1}{32\pi \chi _{1^-}(0)}}\, \left( \sqrt{t_+-q^2}+\sqrt{t_+-t_0}\right) \nonumber \\&\times \left( \sqrt{t_+-q^2}+\sqrt{t_+-t_-}\right) ^{3/2}\nonumber \\&\times \left( \sqrt{t_+-q^2}+\sqrt{t_+}\right) ^{-5} \,\frac{t_+-q^2}{(t_+-t_0)^{1/4}}, \end{aligned}$$

where $t_-=(m_B-m_\pi )^2$, and $\chi _{1^-}(0)$ is the derivative of the transverse component of the polarization function (i.e., the Fourier transform of the vector 2-point function) $\Pi _{\mu \nu }(q)$ at Euclidian momentum $Q^2=-q^2=0$. It is computed perturbatively, using operator product expansion techniques, by relating the $B\rightarrow \pi \ell \nu$ decay amplitude to $\ell \nu \rightarrow B\pi$ inelastic scattering via crossing symmetry and reproducing the correct value of the inclusive $\ell \nu \rightarrow X_b$ amplitude. We will refer to the series parameterization with the outer function in Eq. (179) as Boyd, Grinstein, and Lebed (BGL). The perturbative and OPE truncations imply that the bound is not strict, and one should take it as

180 $$\begin{aligned} \sum _{n=0}^N a_n^{2} \lesssim 1, \end{aligned}$$

where this holds for any choice of *N*. Since the values of |*z*| in the kinematical region of interest are well below 1 for judicious choices of $t_0$, this provides a very stringent bound on systematic uncertainties related to truncation for $N\ge 2$. On the other hand, the outer function in Eq. (179) is somewhat unwieldy and, more relevantly, spoils the correct large $q^2$ behaviour and induces an unphysical singularity at the $B\pi$ threshold.

A simpler choice of outer function has been proposed by Bourrely, Caprini and Lellouch (BCL) in Ref. [436], which leads to a parameterization of the form

181 $$\begin{aligned} f_+(q^2)=\frac{1}{1-q^2/m_{B^*}^2}\,\sum _{n=0}^N a_n^{+}(t_0) z(q^2,t_0)^n. \end{aligned}$$

This satisfies all the basic properties of the form factor, at the price of changing the expression for the bound to

182 $$\begin{aligned} \sum _{j,k=0}^N B_{jk}(t_0)a_j^{+}(t_0)a_k^{+}(t_0) \le 1. \end{aligned}$$

The constants $B_{jk}$ can be computed and shown to be $|B_{jk}|\lesssim \mathcal {O}(10^{-2})$ for judicious choices of $t_0$; therefore, one again finds that truncating at $N\ge 2$ provides sufficiently stringent bounds for the current level of experimental and theoretical precision. It is actually possible to optimize the properties of the expansion by taking

183 $$\begin{aligned} t_0 = t_\mathrm{opt} = (m_B+m_\pi )(\sqrt{m_B}-\sqrt{m_\pi })^2, \end{aligned}$$

which for physical values of the masses results in the semileptonic domain being mapped onto the symmetric interval $|z| \lesssim 0.279$ (where this range differs slightly for the $B^{\pm }$ and $B^0$ decay channels), minimizing the maximum truncation error. If one also imposes the requirement that the asymptotic behaviour $\mathrm{Im}\,f_+(q^2) \sim (q^2-t_+)^{3/2}$ near threshold is satisfied, then the highest-order coefficient is further constrained by

184 $$\begin{aligned} a_N^{+}=-\,\frac{(-1)^N}{N}\,\sum _{n=0}^{N-1}(-1)^n\,n\,a_n^{+}. \end{aligned}$$

Substituting the above constraint on $a_N^{+}$ into Eq. (181) leads to the constrained BCL parameterization

185 $$\begin{aligned} f_+(q^2)=\frac{1}{1-q^2/m_{B^*}^2}\,\sum _{n=0}^{N-1} a_n^{+}\left[ z^n-(-1)^{n-N}\,\frac{n}{N}\,z^N\right] ,\nonumber \\ \end{aligned}$$

which is the standard implementation of the BCL parameterization used in the literature.

Parameterizations of the BGL and BCL kind, to which we will refer collectively as “*z*-parameterizations”, have already been adopted by the BaBar and Belle Collaborations to report their results, and also by the Heavy Flavour Averaging Group (HFAG). Some lattice collaborations, such as FNAL/MILC and ALPHA, have already started to report their results for form factors in this way. The emerging trend is to use the BCL parameterization as a standard way of presenting results for the $q^2$ dependence of semileptonic form factors. Our policy will be to quote results for *z*-parameterizations when the latter are provided in the paper (including the covariance matrix of the fits); when this is not the case, but the published form factors include the full correlation matrix for values at different $q^2$, we will perform our own fit to the constrained BCL ansatz in Eq. (185); otherwise no fit will be quoted. We, however, stress the importance of providing, apart from parameterization coefficients, values for the form factors themselves (in the continuum limit and at physical quark masses) for a number of values of $q^2$, so that the results can be independently parameterized by the readers if so wished.

*The scalar form factor for* $B\rightarrow \pi \ell \nu$ The discussion of scalar $B\rightarrow \pi$ form factor is very similar. The main differences are the absence of a constraint analogue to Eq. (184) and the choice of the overall pole function. In our fits we adopt the simple expansion:

186 $$\begin{aligned} f_0 (q^2) = \sum _{n=0}^{N-1} a_n^0 \; z^n. \end{aligned}$$

We do impose the exact kinematical constraint $f_+ (0) = f_0 (0)$ by expressing the $a_{N-1}^0$ coefficient in terms of all remaining $a_n^+$ and $a_n^0$ coefficients. This constraint introduces important correlations between the $a_n^+$ and $a_n^0$ coefficients; thus only lattice calculations that present the correlations between the vector and scalar form factors can be used in an average that takes into account the constraint at $q^2 = 0$.

Finally we point out that we do not need to use the same number of parameters for the vector and scalar form factors. For instance, with $(N^+ = 3, N^0 = 3)$ we have $a_{0,1,2}^+$ and $a_{0,1}^0$, while with $(N^+ = 3, N^0 = 4)$ we have $a_{0,1,2}^+$ and $a_{0,1,2}^0$ as independent fit parameters. In our average we will choose the combination that optimizes uncertainties.

*Extension to other form factors* The discussion above largely extends to form factors for other semileptonic transitions (e.g., $B_s\rightarrow K$ and $B_{(s)} \rightarrow D^{(*)}_{(s)}$, and semileptonic *D* and *K* decays). As a matter of fact, after the publication of our previous review *z*-parameterizations have been applied in several such cases, as discussed in the relevant sections.

A general discussion of semileptonic meson decay in this context can be found, e.g., in Ref. [499]. Extending what has been discussed above for $B\rightarrow \pi$, the form factors for a generic $H \rightarrow L$ transition will display a cut starting at the production threshold $t_+$, and the optimal value of $t_0$ required in *z*-parameterizations is $t_0=t_+(1-\sqrt{1-t_-/t_+})$ (where $t_\pm =(m_H\pm m_L)^2$). For unitarity bounds to apply, the Blaschke factor has to include all sub-threshold poles with the quantum numbers of the hadronic current – i.e., vector (resp. scalar) resonances in $B\pi$ scattering for the vector (resp. scalar) form factors of $B\rightarrow \pi$, $B_s\rightarrow K$, or $\Lambda _b \rightarrow p$; and vector (resp. scalar) resonances in $B_c\pi$ scattering for the vector (resp. scalar) form factors of $B\rightarrow D$ or $\Lambda _b \rightarrow \Lambda _c$.^43^ Thus, as emphasized above, the control over systematic uncertainties brought in by using *z*-parameterizations strongly depends on implementation details. This has practical consequences, in particular, when the resonance spectrum in a given channel is not sufficiently well known. Caveats may also apply for channels where resonances with a nonnegligible width appear. A further issue is whether $t_+=(m_H+m_L)^2$ is the proper choice for the start of the cut in the cases such as $B_s\rightarrow K\ell \nu$ and $B\rightarrow D\ell \nu$, where there are lighter two-particle states that project on the current (*B*, $\pi$ and $B_c$, $\pi$ for the two processes, respectively).^44^ In any such situation, it is not clear a priori that a given *z*-parameterization will satisfy strict bounds, as has been seen, e.g., in determinations of the proton charge radius from electron-proton scattering [500–502].

The HPQCD Collaboration pioneered a variation on the *z*-parameterization approach, which they refer to as a “modified *z*-expansion,” that is used to simultaneously extrapolate their lattice simulation data to the physical light-quark masses and the continuum limit, and to interpolate/extrapolate their lattice data in $q^2$. This entails allowing the coefficients $a_n$ to depend on the light-quark masses, squared lattice spacing, and, in some cases the charm-quark mass and pion or kaon energy. Because the modified *z*-expansion is not derived from an underlying effective field theory, there are several potential concerns with this approach that have yet to be studied. The most significant is that there is no theoretical derivation relating the coefficients of the modified *z*-expansion to those of the physical coefficients measured in experiment; it therefore introduces an unquantified model dependence in the form-factor shape. As a result, the applicability of unitarity bounds has to be examined carefully. Related to this, *z*-parameterization coefficients implicitly depend on quark masses, and particular care should be taken in the event that some state can move across the inelastic threshold as quark masses are changed (which would in turn also affect the form of the Blaschke factor). Also, the lattice-spacing dependence of form factors provided by Symanzik effective-theory techniques may not extend trivially to *z*-parameterization coefficients. The modified *z*-expansion is now being utilized by collaborations other than HPQCD and for quantities other than $D \rightarrow \pi \ell \nu$ and $D \rightarrow K \ell \nu$, where it was originally employed. We advise treating results that utilize the modified *z*-expansion to obtain form-factor shapes and CKM matrix elements with caution, however, since the systematics of this approach warrant further study.

#### Form factors for $B\rightarrow \pi \ell \nu$

The semileptonic decay processes $B\rightarrow \pi \ell \nu$ enable determinations of the CKM matrixelement $|V_{ub}|$ within the Standard Model via Eq. (172). At the time of our previous review, the only available results for $B\rightarrow \pi \ell \nu$ form factors came from the HPQCD [503] and FNAL/MILC [437] Collaborations. Only HPQCD provided results for the scalar form factor $f_0$. The last two years, however, have witnessed significant progress: FNAL/MILC have significantly upgraded their $B\rightarrow \pi \ell \nu$ results [504],^45^ while a completely new computation has been provided by RBC/UKQCD [505]. All the above computations employ $N_f=2+1$ dynamical configurations, and provide values for both form factors $f_+$ and $f_0$. Finally, HPQCD have recently published the first $N_f=2+1+1$ results for the $B\rightarrow \pi \ell \nu$ scalar form factor, working at zero recoil and pion masses down to the physical value [506]; this adds to previous reports on on-going work to upgrade their 2006 computation [507, 508]. Since the latter result has no immediate impact on current $|V_{ub}|$ determinations, which come from the vector-form-factor-dominated decay channels into light leptons, we will from now on concentrate on the $N_f=2+1$ determinations of the $q^2$ dependence of $B\rightarrow \pi$ form factors.

Both the HPQCD and the FNAL/MILC computations of $B\rightarrow \pi \ell \nu$ amplitudes use ensembles of gauge configurations with $N_f=2+1$ flavours of rooted staggered quarks produced by the MILC Collaboration; however, the latest FNAL/MILC work makes a much more extensive use of the currently available ensembles, both in terms of lattice spacings and light-quark masses. HPQCD have results at two values of the lattice spacing ($a\sim 0.12,~0.09~\mathrm{fm}$), while FNAL/MILC employs four values ($a\sim 0.12,~0.09,~0.06,~0.045~\mathrm{fm}$). Lattice-discretization effects are estimated within HMrS$\chi$PT in the FNAL/MILC computation, while HPQCD quotes the results at $a\sim 0.12~\mathrm{fm}$ as central values and uses the $a\sim 0.09~\mathrm{fm}$ results to quote an uncertainty. The relative scale is fixed in both cases through $r_1/a$. HPQCD set the absolute scale through the $\Upsilon$ $2S{-}1S$ splitting, while FNAL/MILC uses a combination of $f_\pi$ and the same $\Upsilon$ splitting, as described in Ref. [48]. The spatial extent of the lattices employed by HPQCD is $L\simeq 2.4~\mathrm{fm}$, save for the lightest mass point (at $a\sim 0.09~\mathrm{fm}$) for which $L\simeq 2.9~\mathrm{fm}$. FNAL/MILC, on the other hand, uses extents up to $L \simeq 5.8~\mathrm{fm}$, in order to allow for light pion masses while keeping finite-volume effects under control. Indeed, while in the 2006 HPQCD work the lightest RMS pion mass is $400~\mathrm{MeV}$, the latest FNAL/MILC work includes pions as light as $165~\mathrm{MeV}$ – in both cases the bound $m_\pi L \gtrsim 3.8$ is kept. Other than the qualitatively different range of MILC ensembles used in the two computations, the main difference between HPQCD and FNAL/MILC lies in the treatment of heavy quarks. HPQCD uses the NRQCD formalism, with a one-loop matching of the relevant currents to the ones in the relativistic theory. FNAL/MILC employs the clover action with the Fermilab interpretation, with a mostly nonperturbative renormalization of the relevant currents, within which light-light and heavy-heavy currents are renormalized nonperturbatively and one-loop perturbation theory is used for the relative normalization. (See Table 36; full details about the computations are provided in tables in Appendix B.6.3.)

The RBC/UKQCD computation is based on $N_f=2+1$ DWF ensembles at two values of the lattice spacing ($a\sim 0.12,~0.09~\mathrm{fm}$), and pion masses in a narrow interval ranging from slightly above $400~\mathrm{MeV}$ to slightly below $300~\mathrm{MeV}$, keeping $m_\pi L \gtrsim 4$. The scale is set using the $\Omega ^-$ baryon mass. Discretization effects coming from the light sector are estimated in the $1\%$ ballpark using HM$\chi$PT supplemented with effective higher-order interactions to describe cutoff effects. The *b* quark is treated using the Columbia RHQ action, with a mostly nonperturbative renormalization of the relevant currents. Discretization effects coming from the heavy sector are estimated with power-counting arguments to be below $2\%$.

Given the large kinematical range available in the $B\rightarrow \pi$ transition, chiral extrapolations are an important source of systematic uncertainty: apart from the eventual need to reach physical-pion masses in the extrapolation, the applicability of $\chi$PT is not guaranteed for large values of the pion energy $E_\pi$. Indeed, in all computations $E_\pi$ reaches values in the $1~\mathrm{GeV}$ ballpark, and chiral-extrapolation systematics is the dominant source of errors. FNAL/MILC uses *SU*(2) NLO HMrS$\chi$PT for the continuum-chiral extrapolation, supplemented by NNLO analytic terms and hard-pion $\chi$PT terms [509];^46^ systematic uncertainties are estimated through an extensive study of the effects of varying the specific fit ansatz and/or data range. RBC/UKQCD uses *SU*(2) hard-pion HM$\chi$PT to perform its combined continuum-chiral extrapolation, and obtains sizeable estimates for systematic uncertainties by varying the ansätze and ranges used in fits. HPQCD performs chiral extrapolations using HMrS$\chi$PT formulae, and estimates systematic uncertainties by comparing the result with the ones from fits to a linear behaviour in the light-quark mass, continuum HM$\chi$PT, and partially quenched HMrS$\chi$PT formulae (including also data with different sea and valence light-quark masses).

**Table 36:** Results for the $B \rightarrow \pi \ell \nu$ semileptonic form factor. The quantity $\Delta \zeta$ is defined in Eq. (173); the quoted values correspond to $q_1=4$ GeV, $q_2=q_{\mathrm{max}}$, and they are given in $\hbox {ps}^{-1}$

Collaboration	Refs.	$N_{ f}$	Publication status	Continuum extrapolation	Chiral extrapolation	Finite volume	Renormalization	Heavy-quark treatment	*z*-Parameterization	$\Delta \zeta ^{B\pi }$
FNAL/MILC 15	[504]	$2+1$	A						BCL	n/a
RBC/UKQCD 15	[505]	$2+1$	A						BCL	1.77(34)
HPQCD 06	[503]	$2+1$	A						n/a	2.07(41)(39)

FNAL/MILC and RBC/UKQCD describe the $q^2$ dependence of $f_+$ and $f_0$ by applying a BCL parameterization to the form factors extrapolated to the continuum limit, within the range of values of $q^2$ covered by data. RBC/UKQCD generate synthetic data for the form factors at some values of $q^2$ (evenly spaced in *z*) from the continuous function of $q^2$ obtained from the joint chiral-continuum extrapolation, which are then used as input for the fits. After having checked that the kinematical constraint $f_+(0)=f_0(0)$ is satisfied within errors by the extrapolation to $q^2=0$ of the results of separate fits, this constraint is imposed to improve fit quality. In the case of FNAL/MILC, rather than producing synthetic data a functional method is used to extract the *z*-parameterization directly from the fit functions employed in the continuum-chiral extrapolation. The resulting preferred fits for both works are quoted in Table 36. In the case of HPQCD, the parameterization of the $q^2$ dependence of form factors is somewhat intertwined with chiral extrapolations: a set of fiducial values $\{E_\pi ^{(n)}\}$ is fixed for each value of the light-quark mass, and $f_{+,0}$ are interpolated to each of the $E_\pi ^{(n)}$; chiral extrapolations are then performed at fixed $E_\pi$ (i.e. $m_\pi$ and $q^2$ are varied subject to $E_\pi =\hbox {constant}).$ The interpolation is performed using a BZ ansatz. The $q^2$ dependence of the resulting form factors in the chiral limit is then described by means of a BZ ansatz, which is cross-checked against BK, RH, and BGL parameterizations. Unfortunately, the correlation matrix for the values of the form factors at different $q^2$ is not provided, which severely limits the possibilities of combining them with other computations into a global *z*-parameterization.

Based on the parameterized form factors, HPQCD and RBC/UKQCD provide values for integrated decay rates $\Delta \zeta ^{B\pi }$, as defined in Eq. (173); they are quoted in Table 36. The latest FNAL/MILC work, on the other hand, does not quote a value for the integrated ratio. Furthermore, as mentioned above, the field has recently moved forward to determine CKM matrix elements from direct joint fits of experimental results and theoretical form factors, rather than a matching through $\Delta \zeta ^{B\pi }$. Thus, we will not provide here a FLAG average for the integrated rate, and focus on averaging lattice results for the form factors themselves.

In our previous review, we averaged the results for $f_+(q^2)$ in HPQCD 06 and the superseded FNAL/MILC 2008 determination [437], fitting them jointly to our preferred BCL *z*-parameterization, Eq. (185). The new results do not, however, allow for an update of such a joint fit: RBC/UKQCD only provides synthetic values of $f_+$ and $f_0$ at a few values of $q^2$ as an illustration of their results, and FNAL/MILC does not quote synthetic values at all. In both cases, full results for BCL *z*-parameterizations defined by Eq. (185) are quoted. In the case of HPQCD 06, unfortunately, a fit to a BCL *z*-parameterization is not possible, as discussed above.

In order to combine these form factor calculations we start from sets of synthetic data for several $$q^2$$ values. HPQCD and RBC/UKQCD provide directly this information; FNAL/MILC presents only fits to a BCL *z*-parametrization from which we can easily generate an equivalent set of form factor values. It is important to note that in both the RBC/UKQCD synthetic data and the FNAL/MILC *z*-parametrization fits the kinematic constraint at $$q^2=0$$ is automatically included (in the FNAL/MILC case the constraint is manifest in an exact degeneracy of the $$(a_n^+ ,a_n^0)$$ covariance matrix). Due to these considerations, in our opinion the most accurate procedure is to perform a simultaneous fit to all synthetic data for the vector and scalar form factors. Unfortunately the absence of information on the correlation in the HPQCD result between the vector and scalar form factors even at a single $$q^2$$ point makes it impossible to include consistently this calculation in the overall fit. In fact, the HPQCD and FNAL/MILC statistical uncertainties are highly correlated (because they are based on overlapping subsets of MILC $$N_f=2+1$$ ensembles) and, without knowledge of the $$f_+ $$–$$ f_0$$ correlation we are unable to construct the HPQCD-FNAL/MILC off-diagonal entries of the overall covariance matrix.

In conclusion, we will present as our best result a combined vector and scalar form factor fit to the FNAL/MILC and RBC/UKQCD results that we treat as completely uncorrelated. For the sake of completeness we will also show the results of a vector form factor fit alone in which we include one HPQCD datum at $q^2=17.34~\,\mathrm {GeV}^2$ assuming conservatively a 100% correlation between the statistical error of this point and of all FNAL/MILC synthetic data. In spite of contributing just one point, the HPQCD datum has a significant weight in the fit due to its small overall uncertainty. We stress again that this procedure is slightly inconsistent because FNAL/MILC and RBC/UKQCD include information on the kinematic constraint at $q^2=0$ in their $f_+$ results.

**Fig. 24:**
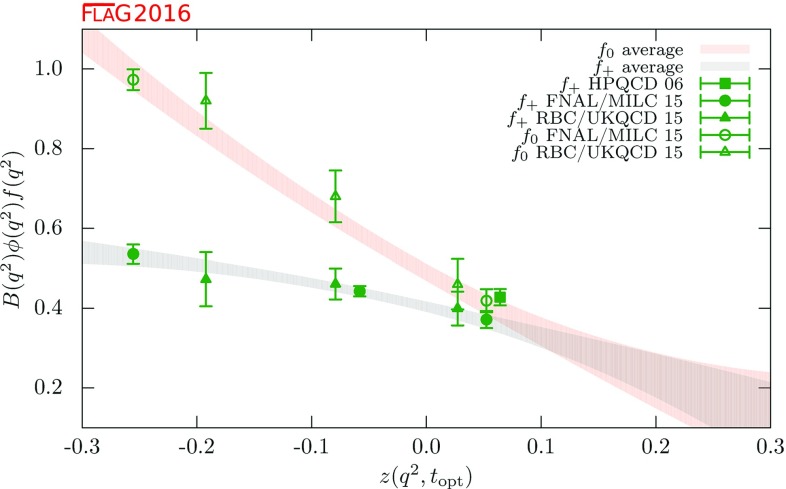
The form factors $(1 - q^2/m_{B^*}^2) f_+(q^2)$ and $f_0 (q^2)$ for $B \rightarrow \pi \ell \nu$ plotted versus *z*. (See text for a discussion of the dataset.) The *grey* and *orange bands* display our preferred $N^+=N^0=3$ BCL fit (five parameters) to the plotted data with errors

The resulting dataset is then fitted to the BCL parameterization in Eqs. (185) and (186). We assess the systematic uncertainty due to truncating the series expansion by considering fits to different orders in *z*. In the two panels of Fig. 24 we show the FNAL/MILC, RBC/UKQCD, and HPQCD data points for $(1-q^2/m_{B^*}^2) f_+(q^2)$ and $f_0 (q^2)$ versus *z*. The data is highly linear and we get a good $\chi ^2/\mathrm{d.o.f.}$ with $N^+ = N^0 = 3$. Note that this implies three independent parameters for $f_+$ corresponding to a polynomial through $\mathcal {O}(z^3)$ and two independent parameters for $f_0$ corresponding to a polynomial through $\mathcal {O}(z^2)$ (the coefficient $a_2^0$ is fixed using the $q^2=0$ kinematic constraint). We cannot constrain the coefficients of the *z*-expansion beyond this order; for instance, including a fourth parameter in $f_+$ yields 100% uncertainties on $a_2^+$ and $a_3^+$. The outcome of the five-parameter BCL fit to the FNAL/MILC and RBC/UKQCD calculations is:

**Table:** 

$$B\rightarrow \pi \; (N_f=2+1)$$						
	Central values	Correlation matrix				
$a_0^+$	0.404 (13)	1	0.404	0.118	0.327	0.344
$a_1^+$	$-$0.68 (13)	0.404	1	0.741	0.310	0.900
$a_2^+$	$-$0.86 (61)	0.118	0.741	1	0.363	0.886
$a_0^0$	0.490 (21)	0.327	0.310	0.363	1	0.233
$a_1^0$	$-$1.61 (16)	0.344	0.900	0.886	0.233	1

The uncertainties on $a_0^{+,0}$, $a_1^{+,0}$ and $a_2^+$ encompass the central values obtained from $N^+=2,4$ and $N^0=2,4,5$ fits and thus adequately reflect the systematic uncertainty on those series coefficients. This can be used as the averaged FLAG result for the lattice-computed form factor $f_+(q^2)$. The coefficient $a_3^+$ can be obtained from the values for $a_0^+$–$a_2^+$ using Eq. (184). The coefficient $a_3^0$ can be obtained from all other coefficients imposing the $f_+(q^2=0) = f_0(q^2=0)$ constraint. The fit is illustrated in Fig. 24.

It is worth stressing that, with respect to our average in the previous edition of the FLAG report, the relative error on $a_0^+$, which dominates the theory contribution to the determination of $|V_{ub}|$, has decreased from $7.3\%$ to $3.2\%$. The dominant factor in this remarkable improvement is the new FNAL/MILC determination of $f_+$. We emphasize that future lattice-QCD calculations of semileptonic form factors should publish their full statistical and systematic correlation matrices to enable others to use the data. It is also preferable to present a set of synthetic form factors data equivalent to the *z*-fit results, since this allows for an independent analysis that avoids further assumptions about the compatibility of the procedures to arrive at a given *z*-parameterization.^47^ It is also preferable to present covariance/correlation matrices with enough significant digits to calculate correctly all their eigenvalues.

For the sake of completeness, we present also a standalone *z*-fit to the vector form factor alone. In this fit we are able to include the single $f_+$ point at $q^2 = 17.34\; \mathrm{GeV}^2$ that we mentioned above. This fit uses the FNAL/MILC and RBC/UKQCD results that do make use of the kinematic constraint at $q^2=0$ but is otherwise unbiased. The results of the three-parameter BCL fit to the HPQCD, FNAL/MILC and RBC/UKQCD calculations of the vector form factor are:

187 $$\begin{aligned}&N_f=2+1: \quad a_0^+ = 0.421(13),\quad a_1^+ = -0.35(10),\nonumber \\&\quad a_2^+ = -0.41(64); \\&\mathrm{corr}(a_i,a_j)=\left( \begin{array}{l@{\quad }l@{\quad }l} 1.000 &{} 0.306 &{} 0.084 \\ 0.306 &{} 1.000 &{} 0.856 \\ 0.084 &{} 0.856 &{} 1.000 \end{array}\right) .\nonumber \end{aligned}$$

Note that the $a_0^+$ coefficient, which is the one most relevant for input to the extraction of $V_{ub}$ from semileptonic $B\rightarrow \pi \ell \nu _\ell (\ell =e,\mu )$ decays, shifts by about a standard deviation.

#### Form factors for $B_s\rightarrow K\ell \nu$

Similar to $B\rightarrow \pi \ell \nu$, measurements of $B_s\rightarrow K\ell \nu$ enable determinations of the CKM matrix element $|V_{ub}|$ within the Standard Model via Eq. (172). From the lattice point of view the two channels are very similar – as a matter of fact, $B_s\rightarrow K\ell \nu$ is actually somewhat simpler, in that the fact that the kaon mass region is easily accessed by all simulations makes the systematic uncertainties related to chiral extrapolation smaller. On the other hand, $B_s\rightarrow K\ell \nu$ channels have not been measured experimentally yet, and therefore lattice results provide SM predictions for the relevant rates.

At the time of our previous review, only preliminary results existed for $B_s \rightarrow K\ell \nu$ form factors. However, as with $B \rightarrow \pi \ell \nu$, great progress has been made during the last year, and first full results for $B_s\rightarrow K\ell \nu$ form factors have been provided by HPQCD [511] and RBC/UKQCD [504] for both form factors $f_+$ and $f_0$, in both cases using $N_f=2+1$ dynamical configurations. Finally, the ALPHA Collaboration determination of $B_s\rightarrow K\ell \nu$ form factors with $N_f=2$ is also well under way [512]; however, since the latter is so far described only in conference proceedings which do not provide quotable results, it will not be discussed here.

The RBC/UKQCD computation has been published together with the $B\rightarrow \pi \ell \nu$ computation discussed in Sect. 8.3.2, all technical details being practically identical. The main difference is that errors are significantly smaller, mostly due to the reduction of systematic uncertainties due to the chiral extrapolation; detailed information is provided in tables in Appendix B.6.3. The HPQCD computation uses ensembles of gauge configurations with $N_f=2+1$ flavours of rooted staggered quarks produced by the MILC Collaboration at two values of the lattice spacing ($a\sim 0.12,~0.09~\mathrm{fm}$), for three and two different sea-pion masses, respectively, down to a value of $260~\mathrm{MeV}$. The *b* quark is treated within the NRQCD formalism, with a one-loop matching of the relevant currents to the ones in the relativistic theory, omitting terms of $\mathcal {O}(\alpha _s\Lambda _\mathrm{QCD}/m_b)$. A HISQ action is used for the valence *s* quark. The continuum-chiral extrapolation is combined with the description of the $q^2$ dependence of the form factors into a modified *z*-expansion (cf. Sect. 8.3.1) that formally coincides in the continuum with the BCL ansatz. The dependence of form factors on the pion energy and quark masses is fitted to a one-loop ansatz inspired by hard-pion $\chi$PT [509], which factorizes out the chiral logarithms describing soft physics. See Table 37 and the tables in Appendix B.6.3 for full details.

**Table 37:** Results for the $B_s \rightarrow K\ell \nu$ semileptonic form factor

Collaboration	Refs.	$N_{ f}$	Publication status	Continuum extrapolation	Chiral extrapolation	Finite volume	Renormalization	Heavy-quark treatment	*z*-Parameterization
RBC/UKQCD 15	[505]	$2+1$	A						BCL
HPQCD 14	[511]	$2+1$	A						BCL$^{\mathrm{a}}$

$^{\mathrm{a}}$ Results from modified *z*-expansion

Both RBC/UKQCD and HPQCD quote values for integrated differential decay rates over the full kinematically available region. However, since the absence of experiment makes the relevant integration interval subject to change, we will not discuss them here, and focus on averages of form factors. In order to proceed to combine the results from the two collaborations, we will follow a similar approach to the one adopted above for $B\rightarrow \pi \ell \nu$: we will take as direct input the synthetic values of the form factors provided by RBC/UKQCD, use the preferred HPQCD parameterization to produce synthetic values, and perform a joint fit to the two datasets.

Note that the kinematic constraint at $q^2=0$ is included explicitly in the results presented by HPQCD (the coefficient $b_0^0$ is expressed analytically in terms of all others) and implicitly in the synthetic data provided by RBC/UKQCD. Therefore, following the procedure we adopted for the $B\rightarrow \pi$ case, we present a joint fit to the vector and scalar form factors and implement explicitly the $q^2=0$ constraint by expressing the coefficient $b^0_{N^0-1}$ in terms of all others.

For the fits we employ a BCL ansatz with $t_+=(M_{B_s}+M_{K^\pm })^2 \simeq 34.35~\,\mathrm {GeV}^2$ and $t_0=(M_{B_s}+M_{K^\pm })(\sqrt{M_{B_s}}-\sqrt{M_{K^\pm }})^2 \simeq 15.27~\,\mathrm {GeV}^2$. Our pole factors will contain a single pole in both the vector and scalar channels, for which we take the mass values $M_{B^*}=5.325~\,\mathrm {GeV}$ and $M_{B^*(0^+)}=5.65~\,\mathrm {GeV}$.^48^

We quote as our preferred result the outcome of the $N^+ = N^0 = 3$ BCL fit:

**Table:** 

$$B_s\rightarrow K \; (N_f=2+1)$$						
	Central values	Correlation matrix				
$a_0^+$	0.360(14)	1	0.098	$-$0.216	0.730	0.345
$a_1^+$	$-$0.828(83)	0.098	1	0.459	0.365	0.839
$a_2^+$	1.11(55)	$-$0.216	0.459	1	0.263	0.6526
$a_0^0$	0.233(10)	0.730	0.365	0.263	1	0.506
$a_1^0$	0.197(81)	0.345	0.839	0.652	0.506	1

where the uncertainties on $a_0$ and $a_1$ encompass the central values obtained from $\mathcal {O}(z^2)$ fits, and thus adequately reflect the systematic uncertainty on those series coefficients.^49^ These can be used as the averaged FLAG results for the lattice-computed form factors $f_+(q^2)$ and $f_0(q^2)$. The coefficient $a_3^+$ can be obtained from the values for $a_0^+$–$a_2^+$ using Eq. (184). The fit is illustrated in Fig. 25.

**Fig. 25:**
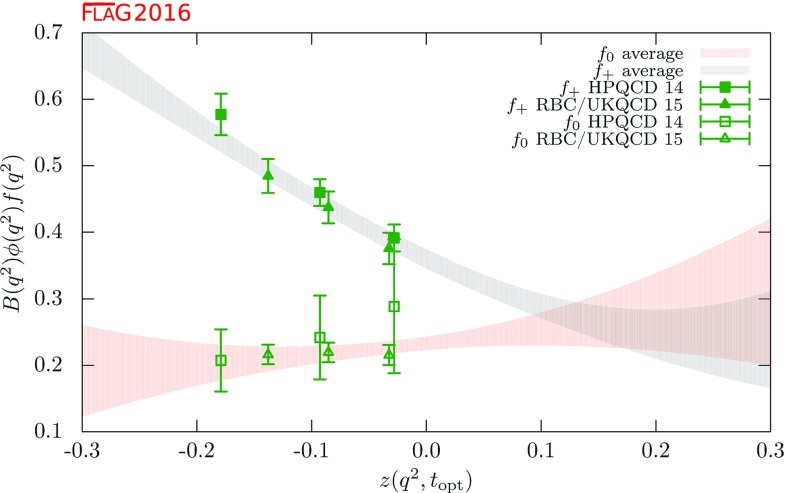
The form factors $(1 - q^2/m_{B^*}^2) f_+(q^2)$ and $(1 - q^2/m_{B^*(0+)}^2) f_0(q^2)$ for $B_s \rightarrow K\ell \nu$ plotted versus *z*. (See text for a discussion of the datasets.) The *grey* and *orange bands* display our preferred $N^+=N^0=3$ BCL fit (five parameters) to the plotted data with errors

#### Form factors for rare and radiative *B*-semileptonic decays to light flavours

Lattice-QCD input is also available for some exclusive semileptonic decay channels involving neutral-current $b\rightarrow q$ transitions at the quark level, where $q=d,s$. Being forbidden at tree level in the SM, these processes allow for stringent tests of potential new physics; simple examples are $B\rightarrow K^*\gamma$, $B\rightarrow K^{(*)}\ell ^+\ell ^-$, or $B\rightarrow \pi \ell ^+\ell ^-$ where the *B* meson (and therefore the light meson in the final state) can be either neutral or charged.

The corresponding SM effective weak Hamiltonian is considerably more complicated than the one for the tree-level processes discussed above: after neglecting top-quark effects, as many as ten dimension-six operators formed by the product of two hadronic currents or one hadronic and one leptonic current appear.^50^ Three of the latter, coming from penguin and box diagrams, dominate at short distances and have matrix elements that, up to small QED corrections, are given entirely in terms of $B\rightarrow (\pi ,K,K^*)$ form factors. The matrix elements of the remaining seven operators can be expressed, up to power corrections whose size is still unclear, in terms of form factors, decay constants and light-cone distribution amplitudes (for the $\pi$, *K*, $K^*$ and *B* mesons) by employing OPE arguments (at large di-lepton invariant mass) and results from Soft Collinear Effective Theory (at small di-lepton invariant mass). In conclusion, the most important contributions to all of these decays are expected to come from matrix elements of current operators (vector, tensor, and axial-vector) between one-hadron states, which in turn can be parameterized in terms of a number of form factors (see Ref. [514] for a complete description).

**Table 38:** Results for the $B \rightarrow K$ semileptonic form factors

Collaboration	Refs.	$N_{ f}$	Publication status	Continuum extrapolation	Chiral extrapolation	Finite volume	Renormalization	Heavy-quark treatment	*z*-Parameterization
HPQCD 13E	[515]	$2+1$	A						BCL
FNAL/MILC 15D	[516]	$2+1$	A						BCL

In channels with pseudoscalar mesons in the final state, the level of sophistication of lattice calculations is similar to the $B\rightarrow \pi$ case and there are results for the vector, scalar, and tensor form factors for $B\rightarrow K\ell ^+\ell ^-$ decays by HPQCD [515], and (very recent) results for both $B\rightarrow \pi \ell ^+\ell ^-$ [517] and $B\rightarrow K\ell ^+\ell ^-$ [516] from FNAL/MILC. Full details about these two calculations are provided in Table 38 and in the tables in Appendix B.6.4. Both computations employ MILC $N_f=2+1$ asqtad ensembles. HPQCD [518] and FNAL/MILC [519] have also companion papers in which they calculate the Standard Model predictions for the differential branching fractions and other observables and compare to experiment. The HPQCD computation employs NRQCD *b* quarks and HISQ valence light quarks, and parameterizes the form factors over the full kinematic range using a model-independent *z*-expansion as in Sect. 8.3.1, including the covariance matrix of the fit coefficients. In the case of the (separate) FNAL/MILC computations, both of them use Fermilab *b* quarks and asqtad light quarks, and a BCL *z*-parameterization of the form factors.

The averaging of the HPQCD and FNAL/MILC results is similar to our treatment of the $B\rightarrow \pi$ and $B_s\rightarrow K$ form factors. In this case, even though the statistical uncertainties are partially correlated because of some overlap between the adopted sets of MILC ensembles, we choose to treat the two calculations as independent. The reason is that, in $B\rightarrow K$, statistical uncertainties are subdominant and cannot be easily extracted from the results presented by HPQCD and FNAL/MILC. Both collaborations provide only the outcome of a simultaneous *z*-fit to the vector, scalar and tensor form factors, which we use to generate appropriate synthetic data. We then impose the kinematic constraint $f_+(q^2=0) = f_0(q^2=0)$ and fit to $(N^+ = N^0 = N^T = 3)$ BCL parametrization. The functional forms of the form factors that we use are identical to those adopted in Ref. [519].^51^ Our results are:

**Table:** 

$$B\rightarrow K \; (N_f=2+1)$$									
	Central values	Correlation matrix							
$a_0^+$	0.4696 (97)	1	0.467	0.058	0.755	0.553	0.609	0.253	0.102
$a_1^+$	$-$0.73 (11)	0.467	1	0.643	0.770	0.963	0.183	0.389	0.255
$a_2^+$	0.39 (50)	0.058	0.643	1	0.593	0.749	$-$0.145	0.023	0.176
$a_0^0$	0.3004 (73)	0.755	0.770	0.593	1	0.844	0.379	0.229	0.187
$a_1^0$	0.42 (11)	0.553	0.963	0.749	0.844	1	0.206	0.325	0.245
$a_0^T$	0.454 (15)	0.609	0.183	$-$0.145	0.379	0.206	1	0.707	0.602
$a_1^T$	$-$1.00 (23)	0.253	0.389	0.023	0.229	0.325	0.707	1	0.902
$a_2^T$	$-$0.89 (96)	0.102	0.255	0.176	0.187	0.245	0.602	0.902	1

**Fig. 26:**
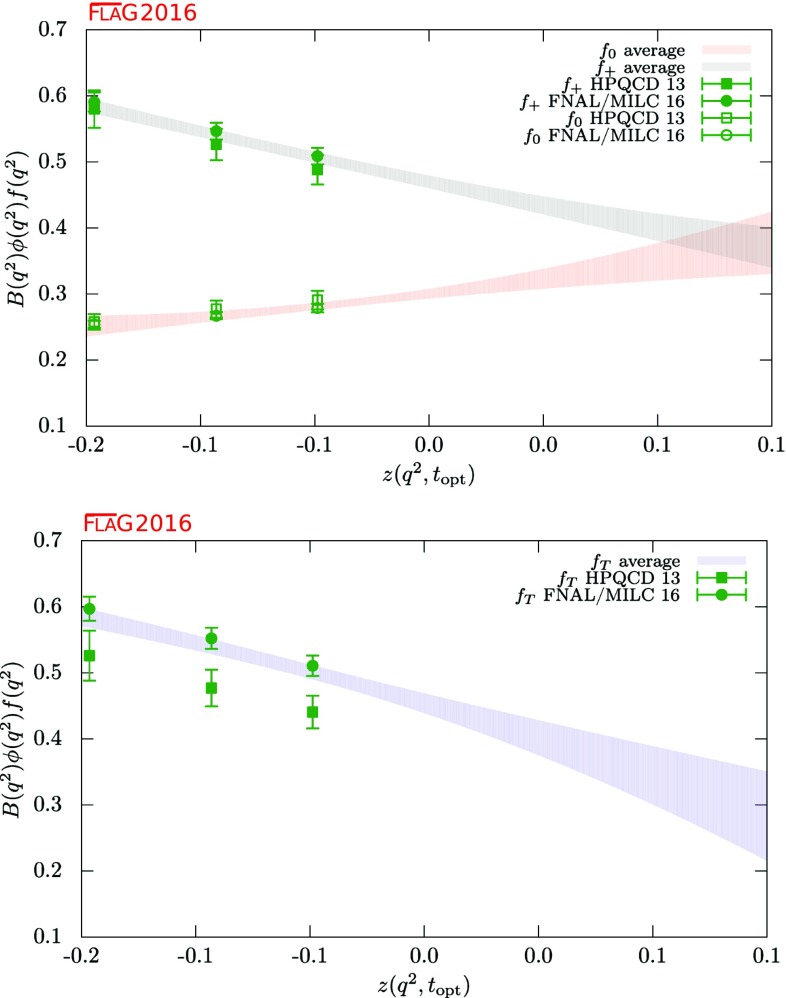
The $B\rightarrow K$ form factors $(1 - q^2/m_{B^*}^2) f_+(q^2)$, $(1 - q^2/m_{B^*(0+)}^2) f_0(q^2)$ and $(1 - q^2/m_{B^*}^2) f_T(q^2)$ plotted versus *z*. (See text for a discussion of the datasets.) The *grey*, *orange* and *blue bands* display our preferred $N^+=N^0=N^T=3$ BCL fit (eight parameters) to the plotted data with errors

The fit is illustrated in Fig. 26. Note that the average for the $f_T$ form factor appears to prefer the FNAL/MILC synthetic data. This happens because we perform a correlated fit of the three form factors simultaneously (both FNAL/MILC and HPQCD present covariance matrices that include correlations between all form factors). We checked that the average for the $f_T$ form factor, obtained neglecting correlations with $f_0$ and $f_+$, is a little lower and lies in between the two datasets.

Lattice computations of form factors in channels with a vector meson in the final state face extra challenges with respect to the case of a pseudoscalar meson: the state is unstable, and the extraction of the relevant matrix element from correlation functions is significantly more complicated; $\chi$PT cannot be used as a guide to extrapolate results at unphysically heavy pion masses to the chiral limit. While the field theory procedures to take resonance effects into account are available [521–529], they have not yet been implemented in the existing preliminary computations, which therefore suffer from uncontrolled systematic errors in calculations of weak decay form factors into unstable vector meson final states, such as the $K^*$ or $\rho$ mesons.^52^

As a consequence of the complexity of the problem, the level of maturity of these computations is significantly below the one present for pseudoscalar form factors. Therefore, we will only provide below a short guide to the existing results.

Concerning channels with vector mesons in the final state, Horgan et al. have obtained the seven form factors governing $B \rightarrow K^* \ell ^+ \ell ^-$ (as well as those for $B_s \rightarrow \phi \, \ell ^+ \ell ^-$) in Ref. [530] using NRQCD *b* quarks and asqtad staggered light quarks. In this work, they use a modified *z*-expansion to simultaneously extrapolate to the physical light-quark masses and continuum and extrapolate in $q^2$ to the full kinematic range. As discussed in Sect. 7.2, the modified *z*-expansion is not based on an underlying effective theory, and the associated uncertainties have yet to be fully studied. Horgan et al. use their form-factor results to calculate the differential branching fractions and angular distributions and discuss the implications for phenomenology in a companion paper [531]. Finally, on-going work on $B\rightarrow K^*\ell ^+\ell ^-$ and $B_s\rightarrow \phi \ell ^+\ell ^-$ by RBC/UKQCD, including first results, have recently been reported in Ref. [532].

### Semileptonic form factors for $B \rightarrow D \ell \nu$, $B \rightarrow D^* \ell \nu$, and $B \rightarrow D \tau \nu$

The semileptonic processes $B \rightarrow D \ell \nu$ and $B \rightarrow D^* \ell \nu$ have been studied extensively by experimentalists and theorists over the years. They allow for the determination of the CKM matrix element $|V_{cb}|$, an extremely important parameter of the Standard Model. $|V_{cb}|$ appears in many quantities that serve as inputs into CKM Unitarity Triangle analyses and reducing its uncertainties is of paramount importance. For example, when $\epsilon _K$, the measure of indirect *CP* violation in the neutral kaon system, is written in terms of the parameters $\rho$ and $\eta$ that specify the apex of the unitarity triangle, a factor of $|V_{cb}|^4$ multiplies the dominant term. As a result, the errors coming from $|V_{cb}|$ (and not those from $B_K$) are now the dominant uncertainty in the Standard Model (SM) prediction for this quantity.

The decay rates for $B \rightarrow D^{(*)}\ell \nu$ can be parameterized in terms of vector and scalar form factors in the same way as, e.g., $B\rightarrow \pi \ell \nu$; see Sect. 8.3. Traditionally, the light channels $\ell =e,~\mu$ have, however, been dealt with using a somewhat different notation, viz.

188 $$\begin{aligned} \frac{\mathrm{d}\Gamma _{B^-\rightarrow D^{0} \ell ^-\bar{\nu }}}{\mathrm{d}w}= & {} \frac{G^2_\mathrm{F} m^3_{D}}{48\pi ^3}(m_B+m_{D})^2(w^2-1)^{3/2}\nonumber \\&\times \, |\eta _{\mathrm {EW}}|^2|V_{cb}|^2 |{\mathcal {G}}(w)|^2, \end{aligned}$$

189 $$\begin{aligned} \frac{\mathrm{d}\Gamma _{B^-\rightarrow D^{0*}\ell ^-\bar{\nu }}}{\mathrm{d}w}= & {} \frac{G^2_\mathrm{F} m^3_{D^*}}{4\pi ^3}(m_B-m_{D^*})^2(w^2-1)^{1/2}\nonumber \\&\times \,|\eta _{\mathrm {EW}}|^2|V_{cb}|^2\chi (w)|{\mathcal {F}}(w)|^2, \end{aligned}$$

where $w \equiv v_B \cdot v_{D^{(*)}}$, $v_P=p_P/m_P$ are the four-velocities of the mesons, and $\eta _\mathrm {EW}=1.0066$ is the one-loop electroweak correction [533]. The function $\chi (w)$ in Eq. (189) depends upon the recoil *w* and the meson masses, and reduces to unity at zero recoil [513]. These formulae do not include terms that are proportional to the lepton mass squared, which can be neglected for $\ell = e, \mu$. Until recently, most unquenched lattice calculations for $B \rightarrow D^* \ell \nu$ and $B \rightarrow D \ell \nu$ decays focussed on the form factors at zero recoil $\mathcal{F}^{B \rightarrow D^*}(1)$ and $\mathcal{G}^{B \rightarrow D}(1)$; these can then be combined with experimental input to extract $|V_{cb}|$. The main reasons for concentrating on the zero-recoil point are that (i) the decay rate then depends on a single form factor, and (ii) for $B \rightarrow D^*\ell \nu$, there are no $\mathcal {O}(\Lambda _{QCD}/m_Q)$ contributions due to Luke’s theorem [534]. Further, the zero-recoil form factor can be computed via a double ratio in which most of the current renormalization cancels and heavy-quark discretization errors are suppressed by an additional power of $\Lambda _{QCD}/m_Q$. Recent work on $B \rightarrow D^{(*)}\ell \nu$ transitions has started to explore the dependence of the relevant form factors on the momentum transfer, using a similar methodology to the one employed in $B\rightarrow \pi \ell \nu$ transitions; we refer the reader to Sect. 8.3 for a detailed discussion.

At the time of the previous version of this review, there were no published complete computations of the form factors for $B \rightarrow D\ell \nu$ decays: $N_f=2+1$ results by FNAL/MILC for $\mathcal{G}^{B \rightarrow D}(1)$ had only appeared in proceedings form [535, 536], while the (now published) $N_f=2$ study by Atoui et al. [537], which in addition to providing $\mathcal{G}^{B \rightarrow D}(1)$ explores the $w>1$ region, was still in preprint form. The latter work also provided the first results for $B_s \rightarrow D_s\ell \nu$ amplitudes, again including information as regards the momentum transfer dependence; this will allow for an independent determination of $|V_{cb}|$ as soon as experimental data are available for these transitions. Meanwhile, the only fully published unquenched results for $\mathcal{F}^{B \rightarrow D^*}(1)$, obtained by FNAL/MILC, dated from 2008 [538]. In the last two years, however, significant progress has been attained in $N_f=2+1$ computations: the FNAL/MILC value for $\mathcal{F}^{B \rightarrow D^*}(1)$ has been updated in Ref. [539], and full results for $B \rightarrow D\ell \nu$ at $w \ge 1$ have been published by FNAL/MILC [540] and HPQCD [541]. These works also provide full results for the scalar form factor, allowing us to analyse the decay in the $\tau$ channel. In the discussion below, we will only refer to this latest generation of results, which supersedes previous $N_f=2+1$ determinations and allows for an extraction of $|V_{cb}|$ that incorporates information as regards the $q^2$ dependence of the decay rate (cf. Sect. 8.7).

**Table 39:** Lattice results for the $B \rightarrow D^* \ell \nu$, $B\rightarrow D\ell \nu$, and $B_s \rightarrow D_s \ell \nu$ semileptonic form factors and *R*(*D*)

Collaboration	Refs.	$N_{ f}$	Publication status	Continuum extrapolation	Chiral extrapolation	Finite volume	Renormalization	Heavy-quark treatment	$w=1$ form factor/ratio	
FNAL/MILC 14	[539]	$2+1$	A						${\mathcal F}^{B\rightarrow D^*}(1)$	0.906(4)(12)
HPQCD 15	[541]	$2+1$	A						${\mathcal G}^{B\rightarrow D}(1)$	1.035(40)
FNAL/MILC 15C	[540]	$2+1$	A						${\mathcal G}^{B\rightarrow D}(1)$	1.054(4)(8)
HPQCD 15	[541]	$2+1$	A						*R*(*D*)	0.300(8)
FNAL/MILC 15C	[540]	$2+1$	A						*R*(*D*)	0.299(11)
Atoui 13	[537]	2	A				–		${\mathcal G}^{B\rightarrow D}(1)$	1.033(95)
Atoui 13	[537]	2	A				–		${\mathcal G}^{B_s\rightarrow D_s}(1)$	1.052(46)

#### $B_{(s)} \rightarrow D_{(s)}$ decays

We will first discuss the $N_f=2+1$ computations of $B \rightarrow D \ell \nu$ by FNAL/MILC and HPQCD mentioned above, both based on MILC asqtad ensembles. Full details about all the computations are provided in Table 39 and in the tables in Appendix B.6.5.

The FNAL/MILC study [540] employs ensembles at four values of the lattice spacing ranging between approximately 0.045 and $0.12~\mathrm{fm}$, and several values of the light-quark mass corresponding to pions with RMS masses ranging between 260 and $670~\mathrm{MeV}$ (with just one ensemble with $M_\pi ^\mathrm{RMS} \simeq 330~\mathrm{MeV}$ at the finest lattice spacing). The *b* and *c* quarks are treated using the Fermilab approach. The quantities directly studied are the form factors $h_\pm$ defined by

190 $$\begin{aligned} \frac{\langle D(p_D)| i\bar{c} \gamma _\mu b| B(p_B)\rangle }{\sqrt{m_D m_B}}= & {} h_+(w)(v_B+v_D)_\mu \nonumber \\&+\,h_-(w)(v_B-v_D)_\mu , \end{aligned}$$

which are related to the standard vector and scalar form factors by

191 $$\begin{aligned} f_+(q^2)= & {} \frac{1}{2\sqrt{r}}\,\left[ (1+r)h_+(w)-(1-r)h_-(w)\right] ,\nonumber \\ f_0(q^2)= & {} \sqrt{r}\left[ \frac{1+w}{1+r}\,h_+(w)+\frac{1-w}{1-r}\,h_-(w)\right] , \end{aligned}$$

with $r=m_D/m_B$. (Recall that $q^2=(p_B-p_D)^2=m_B^2+m_D^2-2wm_Bm_D$.) The hadronic form factor relevant for experiment, ${\mathcal {G}}(w)$, is then obtained from the relation ${\mathcal {G}}(w)=4rf_+(q^2)/(1+r)$. The form factors are obtained from double ratios of 3-point functions in which the flavour-conserving current renormalization factors cancel. The remaining matching factor $\rho _{V^\mu _{cb}}$ is estimated with one-loop lattice perturbation theory. In order to obtain $h_\pm (w)$, a joint continuum-chiral fit is performed to an ansatz that contains the light-quark mass and lattice-spacing dependence predicted by next-to-leading order HMrS$\chi$PT, and the leading dependence on $m_c$ predicted by the heavy-quark expansion ($1/m_c^2$ for $h_+$ and $1/m_c$ for $h_-$). The *w*-dependence, which allows for an interpolation in *w*, is given by analytic terms up to $(1-w)^2$, as well as a contribution from the log proportional to $g^2_{D^*D\pi }$. The total resulting systematic error is $1.2\%$ for $f_+$ and $1.1\%$ for $f_0$. This dominates the final error budget for the form factors. After $f_+$ and $f_0$ have been determined as functions of *w* within the interval of values of $q^2$ covered by the computation, synthetic data points are generated to be subsequently fitted to a *z*-expansion of the BGL form, cf. Sect. 8.3, with pole factors set to unity. This in turn enables one to determine $|V_{cb}|$ from a joint fit of this *z*-expansion and experimental data. The value of the zero-recoil form factor resulting from the *z*-expansion is

192 $$\begin{aligned} \mathcal{G}^{B \rightarrow D}(1)= 1.054(4)_\mathrm{stat}(8)_\mathrm{sys}. \end{aligned}$$

The HPQCD computation [541] considers ensembles at two values of the lattice spacing, $a=0.09,~0.12~\mathrm{fm}$, and two and three values of light-quark masses, respectively. The *b* quark is treated using NRQCD, while for the *c* quark the HISQ action is used. The form factors studied, extracted from suitable 3-point functions, are

193 $$\begin{aligned}&\langle D(p_D)| V^0 | B\rangle = \sqrt{2M_B}f_\parallel ,\nonumber \\&\langle D(p_D)| V^k | B\rangle = \sqrt{2M_B}p^k_D f_\perp , \end{aligned}$$

where $V_\mu$ is the relevant vector current and the *B* rest frame is assumed. The standard vector and scalar form factors are retrieved as

194 $$\begin{aligned} f_+= & {} \frac{1}{\sqrt{2M_B}}f_\parallel + \frac{1}{\sqrt{2M_B}}(M_B-E_D)f_\perp ,\nonumber \\ f_0= & {} \frac{\sqrt{2M_B}}{M_B^2-M_D^2}\left[ (M_B-E_D)f_\parallel +(M_B^2-E_D^2)f_\perp \right] .\nonumber \\ \end{aligned}$$

The currents in the effective theory are matched at one-loop to their continuum counterparts. Results for the form factors are then fitted to a modified BCL *z*-expansion ansatz, which takes into account simultaneously the lattice spacing, light-quark masses, and $q^2$ dependence. For the mass dependence NLO chiral logs are included, in the form obtained in hard-pion $\chi$PT. As in the case of the FNAL/MILC computation, once $f_+$ and $f_0$ have been determined as functions of $q^2$, $|V_{cb}|$ can be determined from a joint fit of this *z*-expansion and experimental data. The work quotes for the zero-recoil vector form factor the result

195 $$\begin{aligned} \mathcal{G}^{B \rightarrow D}(1)=1.035(40). \end{aligned}$$

This value is 1.8$\sigma$ smaller than the FNAL/MILC result and significantly less precise. The dominant source of errors in the $|V_{cb}|$ determination by HPQCD are discretization effects and the systematic uncertainty associated with the perturbative matching.

In order to combine the form factors determinations of HPQCD and FNAL/MILC into a lattice average, we proceed in a similar way as with $B\rightarrow \pi \ell \nu$ and $B_s\rightarrow K\ell \nu$ above. FNAL/MILC quotes synthetic values for the form factors at three values of *w* (or, alternatively, $q^2$) with a full correlation matrix, which we take directly as input. In the case of HPQCD, we use their preferred modified *z*-expansion parameterization to produce synthetic values of the form factors at two different values of $q^2$. This leaves us with a total of five data points in the kinematical range $w\in [1.00,1.11]$. As in the case of $B\rightarrow \pi \ell \nu$, we conservatively assume a 100% correlation of statistical uncertainties between HPQCD and FNAL/MILC. We then fit this dataset to a BCL ansatz, using $t_+=(M_{B^0}+M_{D^\pm })^2 \simeq 51.12~\,\mathrm {GeV}^2$ and $t_0=(M_{B^0}+M_{D^\pm })(\sqrt{M_{B^0}}-\sqrt{M_{D^\pm }})^2 \simeq 6.19~\,\mathrm {GeV}^2$. In our fits, pole factors have been set to unity – i.e., we do not take into account the effect of sub-threshold poles, which is then implicitly absorbed into the series coefficients. The reason for this is our imperfect knowledge of the relevant resonance spectrum in this channel, which does not allow us to decide the precise number of poles needed.^53^ This in turn implies that unitarity bounds do not rigorously apply, which has to be taken into account when interpreting the results (cf. Sect. 8.3.1).

With a procedure similar to what we adopted for the $B\rightarrow \pi$ and $B_s\rightarrow K$ cases, we impose the kinematic constraint at $q^2=0$ by expressing the $a^0_{N^0-1}$ coefficient in the *z*-expansion of $f_0$ in terms of all other coefficients. As mentioned above FNAL/MILC provides synthetic data for $f_+$ and $f_0$ including correlations; HPQCD presents the result of simultaneous *z*-fits to the two form factors including all correlations and, thus enabling us to generate a complete set of synthetic data for $f_+$ and $f_0$. Since both calculations are based on MILC ensembles, we then reconstruct the off-diagonal HPQCD-FNAL/MILC entries of the covariance matrix by conservatively assuming that statistical uncertainties are 100% correlated. The Fermilab/MILC (HPQCD) statistical error is 58% (31%) for every $f_+$ value and 64% (49%) for every $f_0$ one. Using this information we can easily build the off-diagonal block of the overall covariance matrix (e.g., the covariance between $[f_+(q_1^2)]_\mathrm{FNAL}$ and $[f_0(q_2^2)]_\mathrm{HPQCD}$ is $(\delta [f_+(q_1^2)]_\mathrm{FNAL} \times 0.58)\; (\delta [f_0(q_2^2)]_\mathrm{HPQCD} \times 0.49)$, where $\delta f$ is the total error).

For our central value, we choose an $N^+ =N^0=3$ BCL fit:

**Table:** 

$$B\rightarrow D \; (N_f=2+1)$$						
$a_n^i$	Central values	Correlation matrix				
$a_0^+$	0.909 (14)	1	0.737	0.594	0.976	0.777
$a_1^+$	$-$7.11 (65)	0.737	1	0.940	0.797	0.992
$a_2^+$	66 (11)	0.594	0.940	1	0.666	0.938
$a_0^0$	0.794 (12)	0.976	0.797	0.666	1	0.818
$a_1^0$	$-$2.45 (65)	0.777	0.992	0.938	0.818	1

where the coefficient $a_3^+$ can be obtained from the values for $a_0^+$–$a_2^+$ using Eq. (184). The fit is illustrated in Fig. 27.

**Fig. 27:**
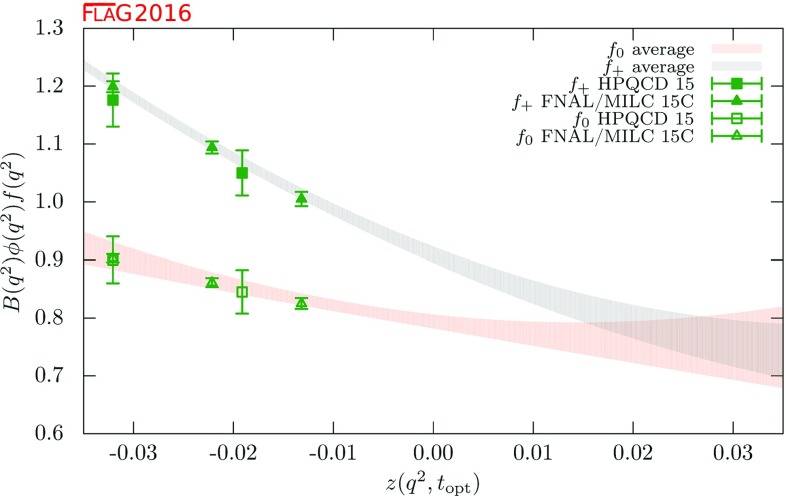
The form factors $f_+(q^2)$ and $f_0(q^2)$ for $B \rightarrow D\ell \nu$ plotted versus *z*. (See text for a discussion of the datasets.) The *grey* and *orange bands* display our preferred $N^+=N^0=3$ BCL fit (five parameters) to the plotted data with errors

Reference [537] is the only existing $N_f=2$ work on $B \rightarrow D\ell \nu$ transitions, which furthermore provides the only available results for $B_s \rightarrow D_s\ell \nu$. This computation uses the publicly available ETM configurations obtained with the twisted-mass QCD action at maximal twist. Four values of the lattice spacing, ranging between $0.054~\mathrm{fm}$ and $0.098~\mathrm{fm}$, are considered, with physical box lengths ranging between 1.7 and $2.7~\mathrm{fm}$. At two values of the lattice spacing two different physical volumes are available. Charged-pion masses range between ${\approx } 270~\mathrm{MeV}$ and ${\approx } 490~\mathrm{MeV}$, with two or three masses available per lattice spacing and volume, save for the $a \approx 0.054~\mathrm{fm}$ point at which only one light mass is available for each of the two volumes. The strange and heavy valence quarks are also treated with maximally twisted-mass QCD.

The quantities of interest are again the form factors $h_\pm$ defined above. In order to control discretization effects from the heavy quarks, a strategy similar to the one employed by the ETM Collaboration in their studies of *B*-meson-decay constants (cf. Sect. 8.1) is employed: the value of $\mathcal{G}(w)$ is computed at a fixed value of $m_c$ and several values of a heavier quark mass $m_h^{(k)}=\lambda ^k m_c$, where $\lambda$ is a fixed scaling parameter, and step-scaling functions are built as

196 $$\begin{aligned} \Sigma _k(w) = \frac{\mathcal{G}(w,\lambda ^{k+1} m_c,m_c,a^2)}{\mathcal{G}(w,\lambda ^k m_c,m_c,a^2)}. \end{aligned}$$

Each ratio is extrapolated to the continuum limit, $\sigma _k(w)=\lim _{a \rightarrow 0}\Sigma _k(w)$. One then exploits the fact that the $m_h \rightarrow \infty$ limit of the step scaling is fixed – in particular, it is easy to find from the heavy-quark expansion that $\lim _{m_h\rightarrow \infty }\sigma (1)=1$. In this way, the physical result at the *b*-quark mass can be reached by interpolating $\sigma (w)$ between the charm region (where the computation can be carried out with controlled systematics) and the known static-limit value.

In practice, the values of $m_c$ and $m_s$ are fixed at each value of the lattice spacing such that the experimental kaon and $D_s$ masses are reached at the physical point, as determined in Ref. [11]. For the scaling parameter, $\lambda =1.176$ is chosen, and eight scaling steps are performed, reaching $m_h/m_c=1.176^9\simeq 4.30$, approximately corresponding to the ratio of the physical *b*- and *c*-masses in the $\overline{\mathrm{MS}}$ scheme at $2~\mathrm{GeV}$. All observables are obtained from ratios that do not require (re)normalization. The ansatz for the continuum and chiral extrapolation of $\Sigma _k$ contains a constant and linear terms in $m_\mathrm{sea}$ and $a^2$. Twisted boundary conditions in space are used for valence-quark fields for better momentum resolution. Applying this strategy the form factors are finally obtained at four reference values of *w* between 1.004 and 1.062, and, after a slight extrapolation to $w=1$, the result is quoted

197 $$\begin{aligned} \mathcal{G}^{B_s \rightarrow D_s}(1) = 1.052(46). \end{aligned}$$

The authors also provide values for the form factor relevant for the meson states with light valence quarks, obtained from a similar analysis to the one described above for the $B_s\rightarrow D_s$ case. Values are quoted from fits with and without a linear $m_\mathrm{sea}/m_s$ term in the chiral extrapolation. The result in the former case, which safely covers systematic uncertainties, is

198 $$\begin{aligned} \mathcal{G}^{B \rightarrow D}(1)=1.033(95). \end{aligned}$$

Given the identical strategy, and the small sensitivity of the ratios used in their method to the light valence- and sea-quark masses, we assign this result the same ratings in Table 39 as those for their calculation of $\mathcal{G}^{B_s \rightarrow D_s}(1)$. Currently the precision of this calculation is not competitive with that of $N_f=2+1$ work, but this is due largely to the small number of configurations analysed by Atoui et al. The viability of their method has been clearly demonstrated, however, which leaves significant room for improvement on the errors of both the $B \rightarrow D$ and the $B_s \rightarrow D_s$ form factors with this approach by including either additional two-flavour data or analysing more recent ensembles with $N_f>2$.

Finally, Atoui et al. also study the scalar and tensor form factors, as well as the momentum transfer dependence of $f_{+,0}$. The value of the ratio $f_0(q^2)/f_+(q^2)$ is provided at a reference value of $q^2$ as a proxy for the slope of $\mathcal{G}(w)$ around the zero-recoil limit.

#### Ratios of $B\rightarrow D\ell \nu$ form factors

The availability of results for the scalar form factor $f_0$ in the latest generation of results for $B\rightarrow D\ell \nu$ amplitudes allows us to study interesting observables that involve the decay in the $\tau$ channel. One such quantity is the ratio

199 $$\begin{aligned} R(D) = \mathcal{B}(B \rightarrow D \tau \nu ) / \mathcal{B}(B \rightarrow D \ell \nu )\quad \hbox {with}\ \ell =e,\mu ,\nonumber \\ \end{aligned}$$

which is sensitive to $f_0$, and can be accurately determined by experiment.^54^ Indeed, the recent availability of experimental results for *R*(*D*) has made this quantity particularly relevant in the search for possible physics beyond the Standard Model. Both FNAL/MILC and HPQCD provide values for *R*(*D*) from their recent form factor computations, discussed above. In the FNAL/MILC case, this result supersedes their 2012 determination, which was discussed in the previous version of this review. The quoted values by FNAL/MILC and HPQCD are

200 $$\begin{aligned}&R(D) = 0.299(11)\,\,\mathrm {Ref.}~[539],\nonumber \\&\quad R(D) = 0.300(8)\,\,\mathrm {Ref.}~[540]. \end{aligned}$$

These results are in excellent agreement, and can be averaged (using the same considerations for the correlation between the two computations as we did in the averaging of form factors) into

201 $$\begin{aligned} R(D) = 0.300(8),\quad \hbox {our average.} \end{aligned}$$

This result is about $1.6\sigma$ lower than the current experimental average for this quantity. It has to be stressed that achieving this level of precision critically depends on the reliability with which the low-$q^2$ region is controlled by the parameterizations of the form factors.

Another area of immediate interest in searches for physics beyond the Standard Model is the measurement of $B_s \rightarrow \mu ^+ \mu ^-$ decays, recently achieved by LHCb.^55^ In addition to the $B_s$ decay constant (see Sect. 8.1), one of the hadronic inputs required by the LHCb analysis is the ratios of $B_q$ meson ($q = d,s$) fragmentation fractions, $f_s / f_d$. A dedicated $N_f=2+1$ study by FNAL/MILC^56^ Ref. [542] addresses the ratios of scalar form factors $f_0^{(q)}(q^2)$, and quotes:

202 $$\begin{aligned}&f_0^{(s)}(M_\pi ^2) / f_0^{(d)}(M_K^2) = 1.046(44)(15),\nonumber \\&f_0^{(s)}(M_\pi ^2) / f_0^{(d)}(M_\pi ^2) = 1.054(47)(17), \end{aligned}$$

where the first error is statistical and the second systematic. These results lead to fragmentation fraction ratios $f_s/f_d$ that are consistent with LHCb’s measurements via other methods [543].

#### $B \rightarrow D^*$ decays

The most precise computation of the zero-recoil form factors needed for the determination of $|V_{cb}|$ from exclusive *B* semileptonic decays comes from the $B \rightarrow D^* \ell \nu$ form factor at zero recoil, $\mathcal{F}^{B \rightarrow D^*}(1)$, calculated by the FNAL/MILC Collaboration. The original computation, published in Ref. [538], has now been updated [539] by employing a much more extensive set of gauge ensembles and increasing the statistics of the ensembles originally considered, while preserving the analysis strategy. There is currently no unquenched computation of the relevant form factors at nonzero recoil.

This work uses the MILC $N_f = 2 + 1$ ensembles. The bottom and charm quarks are simulated using the clover action with the Fermilab interpretation and light quarks are treated via the asqtad staggered fermion action. At zero recoil $\mathcal{F}^{B \rightarrow D^*}(1)$ reduces to a single form factor $h_{A_1}(1)$ coming from the axial-vector current

203 $$\begin{aligned} \langle D^*(v,\epsilon ^\prime )| \mathcal{A}_\mu | \overline{B}(v) \rangle = i \sqrt{2m_B 2 m_{D^*}} \; {\epsilon ^\prime _\mu }^*h_{A_1}(1), \end{aligned}$$

where $\epsilon ^\prime$ is the polarization of the $D^*$. The form factor is accessed through a ratio of 3-point correlators, viz.

204 $$\begin{aligned} \mathcal{R}_{A_1} = \frac{\langle D^*|\bar{c} \gamma _j \gamma _5 b | \overline{B} \rangle \; \langle \overline{B}| \bar{b} \gamma _j \gamma _5 c | D^* \rangle }{\langle D^*|\bar{c} \gamma _4 c | D^* \rangle \; \langle \overline{B}| \bar{b} \gamma _4 b | \overline{B} \rangle } = |h_{A_1}(1)|^2.\nonumber \\ \end{aligned}$$

Simulation data are obtained on MILC ensembles with five lattice spacings, ranging from $a \approx 0.15~\mathrm{fm}$ to $a \approx 0.045~\mathrm{fm}$, and as many as five values of the light-quark masses per ensemble (though just one at the finest lattice spacing). Results are then extrapolated to the physical, continuum/chiral, limit employing staggered $\chi$PT.

The $D^*$ meson is not a stable particle in QCD and decays predominantly into a *D* plus a pion. Nevertheless, heavy–light meson $\chi$PT can be applied to extrapolate lattice simulation results for the $B\rightarrow D^*\ell \nu$ form factor to the physical light-quark mass. The $D^*$ width is quite narrow, 0.096 MeV for the $D^{*\pm }(2010)$ and less than 2.1MeV for the $D^{*0}(2007)$, making this system much more stable and long lived than the $\rho$ or the $K^*$ systems. The fact that the $D^* - D$ mass difference is close to the pion mass leads to the well known “cusp” in $\mathcal{R}_{A_1}$ just above the physical-pion mass [544–546]. This cusp makes the chiral extrapolation sensitive to values used in the $\chi$PT formulae for the $D^*D \pi$ coupling $g_{D^*D\pi }$. The error budget in Ref. [539] includes a separate error of 0.3% coming from the uncertainty in $g_{D^*D \pi }$ in addition to general chiral-extrapolation errors in order to take this sensitivity into account.

The final updated value presented in Ref. [539], that we quote as our average for this quantity, is

205 $$\begin{aligned} \mathcal{F}^{B \rightarrow D^*}(1) = h_{A_1}(1) = 0.906(4)(12)\,, \end{aligned}$$

where the first error is statistical, and the second the sum of systematic errors added in quadrature, making up a total error of 1.4% (down from the original 2.6% of Ref. [538]). The largest systematic uncertainty comes from discretization errors followed by the effects of higher-order corrections in the chiral perturbation theory ansatz.

### Semileptonic form factors for $\Lambda _b\rightarrow p\ell \nu$ and $\Lambda _b\rightarrow \Lambda _c\ell \nu$

A recent new development in Lattice QCD computations for heavy-quark physics is the study of semileptonic decays of the $\Lambda _b$ baryon, with first unquenched results provided in a work by Detmold, Lehner and Meinel [547]. The importance of this result is that, together with a recent analysis by LHCb of the ratio of decay rates $\Gamma (\Lambda _b\rightarrow p\ell \nu )/\Gamma (\Lambda _b\rightarrow \Lambda _c\ell \nu )$ [548], it allows for an exclusive determination of the ratio $|V_{ub}|/|V_{cb}|$ largely independent from the outcome of different exclusive channels, thus contributing a very interesting piece of information to the existing tensions in the determination of third-column CKM matrix elements (cf. Sects. 8.6, 8.7). For that reason, we will discuss these results briefly, notwithstanding the fact that baryon physics is in general out of the scope of the present review.

The amplitudes of the decays $\Lambda _b\rightarrow p\ell \nu$ and $\Lambda _b\rightarrow \Lambda _c\ell \nu$ receive contributions from both the vector and the axial components of the current in the matrix elements $\langle p|\bar{q}\gamma ^\mu (\mathbf {1}-\gamma _5)b|\Lambda _b\rangle$ and $\langle \Lambda _c|\bar{q}\gamma ^\mu (\mathbf {1}-\gamma _5)b|\Lambda _b\rangle$, and can be parameterized in terms of six different form factors – see, e.g., Ref. [549] for a complete description. They split into three form factors $f_+$, $f_0$, $f_\perp$ in the parity-even sector, mediated by the vector component of the current, and another three form factors $g_+,g_0,g_\perp$ in the parity-odd sector, mediated by the axial component. All of them provide contributions that are parametrically comparable.

The computation of Detmold et al. uses RBC/UKQCD $N_f=2+1$ DWF ensembles, and treats the *b* and *c* quarks within the Columbia RHQ approach. Two values of the lattice spacing ($a\sim 0.112,~0.085~\mathrm{fm}$) are considered, with the absolute scale set from the $\Upsilon (2S)$–$\Upsilon (1S)$ splitting. Sea pion masses lie in a narrow interval ranging from slightly above $400~\mathrm{MeV}$ to slightly below $300~\mathrm{MeV}$, keeping $m_\pi L \gtrsim 4$; however, lighter pion masses are considered in the valence DWF action for the *u*, *d* quarks, leading to partial quenching effects in the chiral extrapolation. More importantly, this also leads to values of $M_{\pi ,\mathrm{min}}L$ close to 3.0 (cf. Appendix B.6.3 for details); compounded with the fact that there is only one lattice volume in the computation, an application of the FLAG criteria would lead to a  rating for finite-volume effects. It has to be stressed, however, that our criteria have been developed in the context of meson physics, and their application to the baryon sector is not straightforward; as a consequence, we will refrain from providing a conclusive rating of this computation for the time being.

Results for the form factors are obtained from suitable 3-point functions, and fitted to a modified *z*-expansion ansatz that combines the $q^2$ dependence with the chiral and continuum extrapolations. The main results of the paper are the predictions (errors are statistical and systematic, respectively)

206 $$\begin{aligned}&\frac{1}{|V_{ub}|^2}\int _{15~\mathrm{GeV}^2}^{q^2_\mathrm{max}} \frac{\mathrm{d}\Gamma (\Lambda _b\rightarrow p\mu ^-\bar{\nu }_\mu )}{\mathrm{d}q^2}\,\mathrm{d}q^2\nonumber \\&\quad = 12.32(93)(80)~\mathrm{ps}^{-1},\nonumber \\&\frac{1}{|V_{cb}|^2}\int _{15~\mathrm{GeV}^2}^{q^2_\mathrm{max}}\frac{\mathrm{d}\Gamma (\Lambda _b\rightarrow \Lambda _c\mu ^-\bar{\nu }_\mu )}{\mathrm{d}q^2}\,\mathrm{d}q^2\nonumber \\&\quad = 8.39(18)(32)~\mathrm{ps}^{-1}, \end{aligned}$$

which are the input for the LHCb analysis. Prediction for the total rates in all possible lepton channels, as well as for ratios similar to *R*(*D*) (cf. Sect. 8.4) between the $\tau$ and light lepton channels are also available.

### Determination of $|V_{ub}|$

We now use the lattice-determined Standard Model transition amplitudes for leptonic (Sect. 8.1) and semileptonic (Sect. 8.3) *B*-meson decays to obtain exclusive determinations of the CKM matrix element $|V_{ub}|$. In this section, we describe the aspect of our work that involves experimental input for the relevant charged-current exclusive decay processes. The relevant formulae are Eqs. (147) and (172). Among leptonic channels the only input comes from $B\rightarrow \tau \nu _\tau$, since the rates for decays to *e* and $\mu$ have not yet been measured. In the semileptonic case we only consider $B\rightarrow \pi \ell \nu$ transitions (experimentally measured for $\ell =e,\mu$). As discussed in Sects. 8.3 and 8.5, there are now lattice predictions for the rates of the decays $B_s\rightarrow K\ell \nu$ and $\Lambda _b\rightarrow p\ell \nu$; however, in the former case the process has not been experimentally measured yet, while in the latter case the only existing lattice computation does not meet FLAG requirements for controlled systematics.

We first investigate the determination of $|V_{ub}|$ through the $B\rightarrow \tau \nu _\tau$ transition. This is the only experimentally measured leptonic decay channel of the charged *B*-meson. After the publication of the previous FLAG report [2] in 2013, the experimental measurements of the branching fraction of this channel, $B(B^{-} \rightarrow \tau ^{-} \bar{\nu })$, were updated. While the results from the BaBar Collaboration remain the same as those reported before the end of 2013, the Belle Collaboration reanalysed the data and reported that the value of $B(B^{-} \rightarrow \tau ^{-} \bar{\nu })$ obtained with semileptonic tags changed from $1.54^{+0.38_0.29}_{-0.37-0.31} \times 10^{-4}$ to $1.25 \pm 0.28 \pm 0.27 \times 10^{-4}$ [452]. Table 40 summarizes the current status of experimental results for this branching fraction.

**Table 40:** Experimental measurements for $B(B^{-}\rightarrow \tau ^{-}\bar{\nu })$. The first error on each result is statistical, while the second error is systematic

Collaboration	Tagging method	$B(B^{-}\rightarrow \tau ^{-}\bar{\nu }) \times 10^{4}$
Belle [550]	Hadronic	$0.72^{+0.27}_{-0.25}\pm 0.11$
Belle [452]	Semileptonic	$1.25 \pm 0.28 \pm 0.27$
BaBar [451]	Hadronic	$1.83^{+0.53}_{-0.49}\pm 0.24$
BaBar [551]	Semileptonic	$1.7 \pm 0.8 \pm 0.2$

It is obvious that all the measurements listed in Table 40 have significance less than $5\sigma$, and the uncertainties are dominated by statistical errors. These measurements lead to the averages of experimental measurements for $B(B^{-}\rightarrow \tau \bar{\nu })$ [451, 452],

207 $$\begin{aligned} B(B^{-}\rightarrow \tau \bar{\nu } )= & {} 0.91 \pm 0.22\quad \hbox {from Belle},\nonumber \\= & {} 1.79 \pm 0.48\quad \hbox {from BaBar}. \end{aligned}$$

We notice that minor tension between results from the two collaborations can be observed, even in the presence of large errors. Despite this situation, in Ref. [184] the Particle Data Group performed a global average of $B(B^{-}\rightarrow \tau \bar{\nu } )$ employing all the information in Table 40. Here we choose to proceed with the strategy of quoting different values of $|V_{ub}|$ as determined using inputs from the Belle and the BaBar experiments shown in Eq. (207), respectively.

Combining the results in Eq. (207) with the experimental measurements of the mass of the $\tau$-lepton and the *B*-meson lifetime and mass, the Particle Data Group presented [184]

208 $$\begin{aligned} |V_{ub}| f_{B}= & {} 0.72 \pm 0.09~\mathrm{MeV}\quad \hbox {from Belle},\nonumber \\= & {} 1.01 \pm 0.14~\mathrm{MeV}\quad \hbox {from BaBar}, \end{aligned}$$

which can be used to extract $|V_{ub}|$.

209 $$\begin{aligned} \begin{array}{lll} N_f=2 &{}\quad \hbox {Belle}~B\rightarrow \tau \nu _\tau : &{}\quad |V_{ub}| = 3.83(48)(15) \times 10^{-3} ,\\ N_f=2+1 &{}\quad \hbox {Belle}~B\rightarrow \tau \nu _\tau : &{}\quad |V_{ub}| = 3.75(47)(9) \times 10^{-3} ,\\ N_f=2+1+1 &{}\quad \hbox {Belle}~B\rightarrow \tau \nu _\tau : &{}\quad |V_{ub}| = 3.87(48)(9) \times 10^{-3} ;\\ N_f=2 &{}\quad \hbox {Babar}~B\rightarrow \tau \nu _\tau : &{}\quad |V_{ub}| = 5.37(74)(21) \times 10^{-3} ,\\ N_f=2+1 &{}\quad \hbox {Babar}~B\rightarrow \tau \nu _\tau : &{}\quad |V_{ub}| = 5.26(73)(12) \times 10^{-3} ,\\ N_f=2+1+1 &{}\quad \hbox {Babar}~B\rightarrow \tau \nu _\tau : &{}\quad |V_{ub}| = 5.43(75)(12) \times 10^{-3}. \end{array}\nonumber \\ \end{aligned}$$

where the first error comes from experiment and the second comes from the uncertainty in $f_B$.

Let us now turn our attention to semileptonic decays. The experimental value of $|V_{ub}|f_+(q^2)$ can be extracted from the measured branching fractions for $B^0\rightarrow \pi ^\pm \ell \nu$ and/or $B^\pm \rightarrow \pi ^0\ell \nu$ applying Eq. (172);^57^ $|V_{ub}|$ can then be determined by performing fits to the constrained BCL *z* parameterization of the form factor $f_+(q^2)$ given in Eq. (185). This can be done in two ways: one option is to perform separate fits to lattice and experimental results, and extract the value of $|V_{ub}|$ from the ratio of the respective $a_0$ coefficients; a second option is to perform a simultaneous fit to lattice and experimental data, leaving their relative normalization $|V_{ub}|$ as a free parameter. We adopt the second strategy, because it combines the lattice and experimental input in a more efficient way, leading to a smaller uncertainty on $|V_{ub}|$.

The available state-of-the-art experimental input, as employed, e.g., by HFAG, consists of five datasets: three untagged measurements by BaBar (6-bin [552] and 12-bin [439]) and Belle [438], all of which assume isospin symmetry and provide combined $B^0\rightarrow \pi ^-$ and $B^+\rightarrow \pi ^0$ data; and the two tagged Belle measurements of $\bar{B}^0\rightarrow \pi ^+$ (13-bin) and $B^-\rightarrow \pi ^0$ (7-bin ) [553]. In the previous version of the FLAG review [2] we only used the 13-bin Belle and 12-bin BaBar datasets, and performed separate fits to them due to the lack of information on systematic correlations between them. Now, however, we will follow established practice and perform a combined fit to all the experimental data. This is based on the existence of new information as regards cross-correlations, which allows us to obtain a meaningful final error estimate.^58^ The lattice input dataset will be the same as discussed in Sect. 8.3.

We perform a constrained BCL fit of the vector and scalar form factors (this is necessary in order to take into account the $f_+(q^2=0) = f_0 (q^2)$ constraint) together with the combined experimental datasets. We find that the error on $V_{ub}$ stabilizes for $(N^+ = N^0 = 3)$. The result of the combined fit is

**Table:** 

$$B\rightarrow \pi \ell \nu \; (N_f=2+1)$$							
	Central values	Correlation matrix					
$V_{ub}^{} \times 10^3$	3.73 (14)	1	0.852	0.345	$-$0.374	0.211	0.247
$a_0^+$	0.414 (12)	0.852	1	0.154	$-$0.456	0.259	0.144
$a_1^+$	$-$0.494 (44)	0.345	0.154	1	$-$0.797	$-$0.0995	0.223
$a_2^+$	$-$0.31 (16)	$-$0.374	$-$0.456	$-$0.797	1	0.0160	$-$0.0994
$a_0^0$	0.499 (19)	0.211	0.259	$-$0.0995	0.0160	1	$-$0.467
$a_1^0$	$-$1.426 (46)	0.247	0.144	0.223	$-$0.0994	$-$0.467	1

**Fig. 28:**
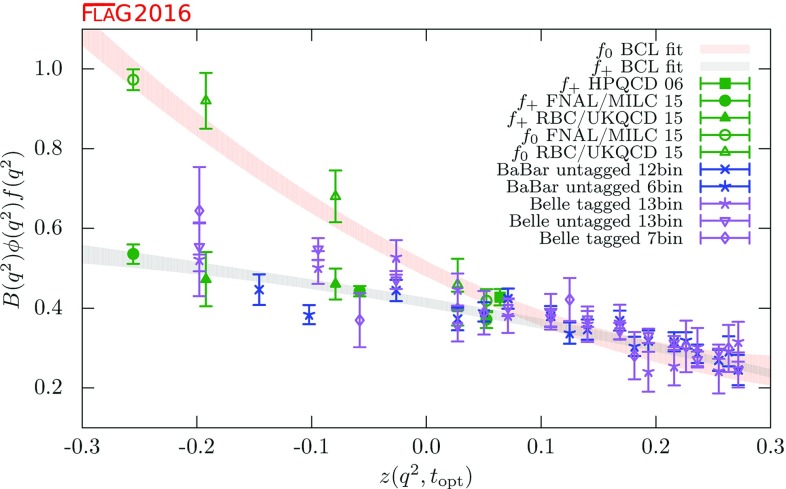
Lattice and experimental data for $(1-q^2/m_{B^*}^2)f_+^{B\rightarrow \pi }(q^2)$ and $f_0^{B\rightarrow \pi } (q^2)$ versus *z*. *Green symbols* denote lattice-QCD points included in the fit, while *blue* and *indigo points* show experimental data divided by the value of $|V_{ub}|$ obtained from the fit. The *grey* and *orange bands* display the preferred $N^+ = N^0 = 3$ BCL fit (six parameters) to the lattice-QCD and experimental data with errors

Figure 28 shows both the lattice and the experimental data for $(1-q^2/m_{B^*}^2)f_+(q^2)$ as a function of $z(q^2)$, together with our preferred fit; experimental data have been rescaled by the resulting value for $|V_{ub}|^2$. It is worth noting the good consistency between the form factor shapes from lattice and experimental data. This can be quantified, e.g., by computing the ratio of the two leading coefficients in the constrained BCL parameterization: the fit to lattice form factors yields $a_1^+/a_0^+=-1.67(12)$ (cf. the results presented in Sect. 8.3.2), while the above lattice+experiment fit yields $a_1^+/a_0^+=-1.193(16)$.

We plot the values of $|V_{ub}|$ we have obtained in Fig. 30, where the determination through inclusive decays by the Heavy Flavour Averaging Group (HFAG) [197], yielding $|V_{ub}| = 4.62 (20) (29) \times 10^{-3}$, is also shown for comparison. In this plot the tension between the BaBar and the Belle measurements of $B(B^{-} \rightarrow \tau ^{-} \bar{\nu })$ is manifest. As discussed above, it is for this reason that we do not extract $|V_{ub}|$ through the average of results for this branching fraction from these two collaborations. In fact this means that a reliable determination of $|V_{ub}|$ using information from leptonic *B*-meson decays is still absent; the situation will only clearly improve with the more precise experimental data expected from Belle II. The value for $|V_{ub}|$ obtained from semileptonic *B* decays for $N_f=2+1$, on the other hand, is significantly more precise than both the leptonic and the inclusive determinations, and exhibits the well-known ${\sim } 3\sigma$ tension with the latter.

### Determination of $|V_{cb}|$

We will now use the lattice QCD results for the $B \rightarrow D^{(*)}\ell \nu$ form factors in order to obtain determinations of the CKM matrix element $|V_{cb}|$ in the Standard Model. The relevant formulae are given in Eq. (189).

Let us summarize the lattice input that satisfies FLAG requirements for the control of systematic uncertainties, discussed in Sect. 8.4. In the (experimentally more precise) $B\rightarrow D^*\ell \nu$ channel, there is only one $N_f=2+1$ lattice computation of the relevant form factor ${\mathcal {F}}^{B\rightarrow D^*}$ at zero recoil. Concerning the $B \rightarrow D\ell \nu$ channel, for $N_f=2$ there is one determination of the relevant form factor ${\mathcal {G}}^{B\rightarrow D}$ at zero recoil;^59^ while for $N_f=2+1$ there are two determinations of the $B \rightarrow D$ form factor as a function of the recoil parameter in roughly the lowest third of the kinematically allowed region. In the latter case, it is possible to replicate the analysis carried out for $|V_{ub}|$ in Sect. 8.6, and perform a joint fit to lattice and experimental data; in the former, the value of $|V_{cb}|$ has to be extracted by matching to the experimental value for ${\mathcal {F}}^{B\rightarrow D^*}(1)\eta _\mathrm{EW}|V_{cb}|$ and ${\mathcal {G}}^{B\rightarrow D}(1)\eta _\mathrm{EW}|V_{cb}|$.

The latest experimental average by HFAG [197] for the $B\rightarrow D^*$ form factor at zero recoil is

210 $$\begin{aligned} {\mathcal {F}}^{B\rightarrow D^*}(1)\eta _\mathrm{EW}|V_{cb}| = 35.81(0.45)\times 10^{-3}. \end{aligned}$$

By using $\eta _\mathrm{EW}=1.00662$ 60 and the lattice value for ${\mathcal {F}}^{B\rightarrow D^*}(1)$ in Eq. (205), we thus extract our average

211 $$\begin{aligned}&N_f=2+1&B\rightarrow D^*\ell \nu :\quad |V_{cb}| \!=\! 39.27(56)(49) \times 10^{-3} , \end{aligned}$$

where the first uncertainty comes from the lattice computation and the second from the experimental input. For the zero-recoil $B \rightarrow D$ form factor, HFAG quotes

212 $$\begin{aligned} \hbox {HFAG:} \quad {\mathcal {G}}^{B\rightarrow D}(1)\eta _\mathrm{EW}|V_{cb}| = 42.65(1.53)\times 10^{-3}. \end{aligned}$$

This average is strongly dominated by the BaBar input. The set of experimental results for $B \rightarrow D\ell \nu$ has, however, been significantly improved by the recent publication of a new Belle measurement [554], which quotes

213 $$\begin{aligned} \hbox {Belle 2016:} \quad {\mathcal {G}}^{B\rightarrow D}(1)\eta _\mathrm{EW}|V_{cb}| = 42.29(1.37)\times 10^{-3}.\nonumber \\ \end{aligned}$$

Given the difficulties to include the latter number in a global average replicating the procedure followed by HFAG, and the fact that the final uncertainty will be completely dominated by the error of the lattice input in Eq. (198), we will conservatively use the value in Eq. (212) to provide an average for $N_f=2$, and quote

214 $$\begin{aligned} N_f=2\quad B\rightarrow D\ell \nu : |V_{cb}| = 41.0(3.8)(1.5) \times 10^{-3}.\nonumber \\ \end{aligned}$$

Finally, for $N_f=2+1$ we will perform, as discussed above, a joint fit to the available lattice data, discussed in Sect. 8.4, and state-of-the-art experimental determinations. In this case we will combine the aforementioned recent Belle measurement [554], which provides partial integrated decay rates in 10 bins in the recoil parameter *w*, with the 2010 BaBar dataset in Ref. [555], which quotes the value of ${\mathcal {G}}^{B\rightarrow D}(w)\eta _\mathrm{EW}|V_{cb}|$ for ten values of *w*.^61^ The fit is dominated by the more precise Belle data; given this, and the fact that only partial correlations among systematic uncertainties are to be expected, we will treat both datasets are uncorrelated.^62^

A constrained $(N^+ = N^0 = 3)$ BCL fit using the same ansatz as for lattice-only data in Sect. 8.4, yields our average

**Table:** 

$$B\rightarrow D\ell \nu \; (N_f=2+1)$$							
	Central values	Correlation matrix					
$|V_{cb}| \times 10^3$	40.1 (1.0)	1	-0.525	-0.431	-0.185	-0.526	-0.497
$a_0^+$	0.8944 (95)	-0.525	1	0.282	-0.162	0.953	0.450
$a_1^+$	-8.08 (22)	-0.431	0.282	1	0.613	0.350	0.934
$a_2^+$	49.0 (4.6)	-0.185	-0.162	0.613	1	-0.0931	0.603
$a_0^0$	0.7802 (75)	-0.526	0.953	0.350	-0.0931	1	0.446
$a_1^0$	-3.42 (22)	-0.497	0.450	0.934	0.603	0.446	1

**Fig. 29:**
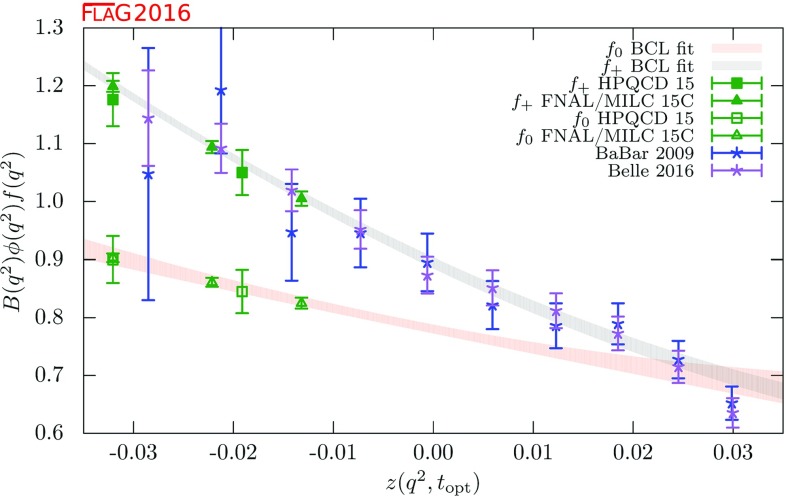
Lattice and experimental data for $f_+^{B\rightarrow D}(q^2)$ and $f_0^{B\rightarrow D}(q^2)$ versus *z*. *Green symbols* denote lattice-QCD points included in the fit, while *blue* and *indigo points* show experimental data divided by the value of $|V_{cb}|$ obtained from the fit. The *grey* and *orange bands* display the preferred $N^+=N^0=3$ BCL fit (six parameters) to the lattice-QCD and experimental data with errors

The fit is illustrated in Fig. 29. In passing, we note that, if correlations between the FNAL/MILC and HPQCD calculations are neglected, the $V_{cb}$ central value rises to $40.3 \times 10^{-3}$ in nice agreement with the results presented in Ref. [800].

**Fig. 30:**
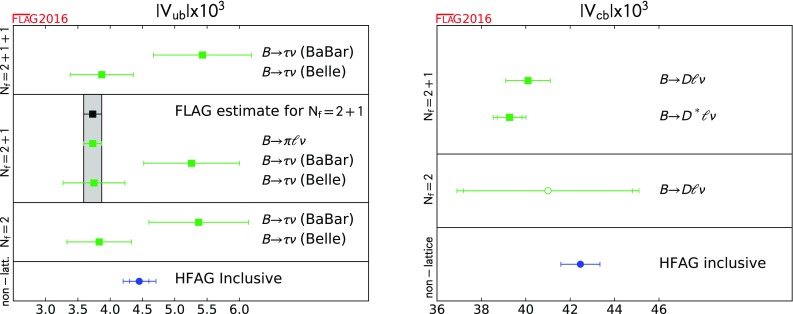
*Left* Summary of $|V_{ub}|$ determined using: (i) the *B*-meson leptonic decay branching fraction, $B(B^{-} \rightarrow \tau ^{-} \bar{\nu })$, measured at the Belle and BaBar experiments, and our averages for $f_{B}$ from lattice QCD; and (ii) the various measurements of the $B\rightarrow \pi \ell \nu$ decay rates by Belle and BaBar, and our averages for lattice determinations of the relevant vector form factor $f_+(q^2)$. *Right* Same for determinations of $|V_{cb}|$ using semileptonic decays. The HFAG inclusive results are from Ref. [197]

Our results are summarized in Table 41, which also shows the HFAG inclusive determination of $|V_{cb}|$ for comparison, and illustrated in Fig. 30. The $N_f=2+1$ results coming from $B\rightarrow D^*\ell \nu$ and $B\rightarrow D\ell \nu$ could in principle be averaged; we will, however, not do so, due to the difficulties of properly taking into account experimental correlations. We will thus leave them as separate exclusive estimates, which show good mutual consistence, and the well-known tension with the inclusive determination.

**Table 41:** Results for $|V_{cb}|$. When two errors are quoted in our averages, the first one comes from the lattice form factor, and the second from the experimental measurement. The HFAG inclusive average obtained in the kinetic scheme from Ref. [197] is shown for comparison

	From	$|V_{cb}| \times 10^3$
Our average for $N_f=2+1$	$B \rightarrow D^*\ell \nu$	39.27(56)(49)
Our average for $N_f=2+1$	$B \rightarrow D\ell \nu$	40.1(1.0)
Our average for $N_f=2$	$B \rightarrow D\ell \nu$	41.0(3.8)(1.5)
HFAG inclusive average	$B \rightarrow X_c\ell \nu$	42.46(88)

## The strong coupling $\alpha _\mathrm{s}$

### Introduction

The strong coupling $\bar{g}(\mu )$ defined at scale $\mu$, plays a key role in the understanding of QCD and in its application for collider physics. For example, the parametric uncertainty from $\alpha _s$ is one of the dominant sources of uncertainty in the Standard Model prediction for the $H \rightarrow b\bar{b}$ partial width, and the largest source of uncertainty for $H \rightarrow gg$. Thus higher precision determinations of $\alpha _s$ are needed to maximize the potential of experimental measurements at the LHC, and for high-precision Higgs studies at future colliders [556–558]. The value of $\alpha _s$ also yields one of the essential boundary conditions for completions of the standard model at high energies.

In order to determine the running coupling at scale $\mu$

215 $$\begin{aligned} \alpha _s(\mu ) = { \bar{g}^2(\mu ) \over 4\pi }, \end{aligned}$$

we should first “measure” a short-distance quantity ${\mathcal {Q}}$ at scale $\mu$ either experimentally or by lattice calculations and then match it with a perturbative expansion in terms of a running coupling, conventionally taken as $\alpha _{\overline{\mathrm{MS}}}(\mu )$,

216 $$\begin{aligned} {\mathcal {Q}}(\mu ) = c_1 \alpha _{\overline{\mathrm{MS}}}(\mu ) + c_2 \alpha _{\overline{\mathrm{MS}}}(\mu )^2 + \cdots . \end{aligned}$$

The essential difference between continuum determinations of $\alpha _s$ and lattice determinations is the origin of the values of $\mathcal {Q}$ in Eq. (216).

The basis of continuum determinations are experimentally measurable cross sections from which $\mathcal {Q}$ is defined. These cross sections have to be sufficiently inclusive and at sufficiently high scales such that perturbation theory can be applied. Often hadronization corrections have to be used to connect the observed hadronic cross sections to the perturbative ones. Experimental data at high $\mu$, where perturbation theory is progressively more precise, usually have increasing experimental errors, and it is not easy to find processes which allow one to follow the $\mu$ dependence of a single $\mathcal {Q}(\mu )$ over a range where $\alpha _s(\mu )$ changes significantly and precision is maintained.

In contrast, in lattice gauge theory, one can design $\mathcal {Q}(\mu )$ as Euclidean short-distance quantities which are not directly related to experimental observables. This allows us to follow the $\mu$ dependence until the perturbative regime is reached and nonperturbative “corrections” are negligible. The only experimental input for lattice computations of $\alpha _s$ is the hadron spectrum which fixes the overall energy scale of the theory and the quark masses. Therefore experimental errors are completely negligible and issues such as hadronization do not occur. We can construct many short-distance quantities that are easy to calculate nonperturbatively in lattice simulations with small statistical uncertainties. We can also simulate at parameter values that do not exist in nature (for example with unphysical quark masses between bottom and charm) to help control systematic uncertainties. These features mean that precise results for $\alpha _s$ can be achieved with lattice gauge theory computations. Further, as in the continuum, the different methods available to determine $\alpha _s$ in lattice calculations with different associated systematic uncertainties enable valuable cross-checks. Practical limitations are discussed in the next section, but a simple one is worth mentioning here. Experimental results (and therefore the continuum determinations) of course have all quarks present, while in lattice gauge theories only the light ones are included and one then is forced to use the matching at thresholds, as discussed in the following subsection.

It is important to keep in mind that the dominant source of uncertainty in most present day lattice-QCD calculations of $\alpha _s$ are from the truncation of continuum/lattice perturbation theory and from discretization errors. Perturbative truncation errors are of a different nature than most other lattice (or continuum) systematics, in that they often cannot easily be estimated from studying the data itself. Further, the size of higher-order coefficients in the perturbative series can sometimes turn out to be larger than naive expectations based on power counting from the behaviour of lower-order terms.

The various phenomenological approaches to determining the running coupling, $\alpha ^{(5)}_{\overline{\mathrm{MS}}}(M_Z)$ are summarized by the Particle Data Group [151]. The PDG review lists 4 categories of phenomenological results used to obtain the running coupling using hadronic $\tau$ decays, hadronic final states of $e^+e^-$ annihilation, deep inelastic lepton–nucleon scattering and electroweak precision data. Excluding lattice results, the PDG quotes a weighted average of

217 $$\begin{aligned} \alpha ^{(5)}_{\overline{\mathrm{MS}}}(M_Z) = 0.1175(17), \end{aligned}$$

compared to $\alpha ^{(5)}_{\overline{\mathrm{MS}}}(M_Z) = 0.1183(12)$ of the previous review [559]. For a general overview of the various phenomenological and lattice approaches see e.g. Ref. [560]. We note that perturbative truncation errors are also the dominant source of uncertainty in several of the phenomenological determinations of $\alpha _s$. In particular, the extraction of $\alpha _s$ from $\tau$ data, which is the most precise and has the largest impact on the nonlattice average in Eq. (217) is especially sensitive to the treatment of higher-order perturbative terms. This is important to keep in mind when comparing our chosen range for $\alpha ^{(5)}_{\overline{\mathrm{MS}}}(M_Z)$ from lattice determinations in Eq. (261) with the nonlattice average from the PDG.

#### Scheme and scale dependence of $\alpha _s$ and $\Lambda _\mathrm{QCD}$

Despite the fact that the notion of the QCD coupling is initially a perturbative concept, the associated $\Lambda$ parameter is nonperturbatively defined

218 $$\begin{aligned} \Lambda\equiv & {} \mu \,(b_0\bar{g}^2(\mu ))^{-b_1/(2b_0^2)} e^{-1/(2b_0\bar{g}^2(\mu ))}\nonumber \\&\times \exp \left[ -\int _0^{\bar{g}(\mu )}\mathrm{d}x \left( {1\over \beta (x)} + {1 \over b_0x^3} - {b_1 \over b_0^2x} \right) \right] , \end{aligned}$$

where $\beta$ is the full renormalization group function in the scheme which defines $\bar{g}$, and $b_0$ and $b_1$ are the first two scheme-independent coefficients of the perturbative expansion

219 $$\begin{aligned} \beta (x) \sim -b_0 x^3 -b_1 x^5 + \cdots , \end{aligned}$$

with

220 $$\begin{aligned} b_0 = {1\over (4\pi )^2} \left( 11 - {2\over 3}N_f \right) ,\quad b_1 = {1\over (4\pi )^4} \left( 102 - {38 \over 3} N_f \right) .\nonumber \\ \end{aligned}$$

Thus the $\Lambda$ parameter is renormalization-scheme-dependent but in an exactly computable way, and lattice gauge theory is an ideal method to relate it to the low-energy properties of QCD.

The change in the coupling from one scheme, *S*, to another (taken here to be the $\overline{\mathrm{MS}}$ scheme) is perturbative,

221 $$\begin{aligned} g_{\overline{\mathrm{MS}}}^2(\mu ) = g_\mathrm{S}^2(\mu ) (1 + c^{(1)}_g g_\mathrm{S}^2(\mu ) + \cdots ), \end{aligned}$$

where $c^{(i)}_g$ are the finite renormalization coefficients. The scale $\mu$ must be taken high enough for the error in keeping only the first few terms in the expansion to be small. On the other hand, the conversion to the $\Lambda$ parameter in the $\overline{\mathrm{MS}}$ scheme is given exactly by

222 $$\begin{aligned} \Lambda _{\overline{\mathrm{MS}}} = \Lambda _\mathrm{S} \exp [ c_g^{(1)}/(2b_0)]. \end{aligned}$$

By convention $\alpha _{\overline{\text {MS}}}$ is usually quoted at a scale $\mu =M_Z$ where the appropriate effective coupling is the one in the 5-flavour theory: $\alpha ^{(5)}_{\overline{\mathrm{MS}}}(M_Z)$. In order to obtain it from a result with fewer flavours, one connects effective theories with different number of flavours as discussed by Bernreuther and Wetzel [561]. For example one considers the ${\overline{\text {MS}}}$ scheme, matches the 3-flavour theory to the 4-flavour theory at a scale given by the charm-quark mass, runs with the 4-loop $\beta$-function of the 4-flavour theory to a scale given by the *b*-quark mass and there matches to the 5-flavour theory, after which one runs up to $\mu =M_Z$. For the matching relation at a given quark threshold we use the mass $m_\star$ which satisfies $m_\star = \overline{m}_{\overline{\text {MS}}}(m_\star )$, where $\overline{m}$ is the running mass (analogous to the running coupling). Then

223 $$\begin{aligned} \bar{g}^2_{N_f-1}(m_\star )= & {} \bar{g}^2_{N_f}(m_\star )\times [1+t_2\,\bar{g}^4_{N_f}(m_\star )\nonumber \\&+\,t_3\,\bar{g}^6_{N_f}(m_\star )+ \cdots ] \end{aligned}$$

with [562]

224 $$\begin{aligned} t_2= & {} {1 \over (4\pi ^2)^2} {11\over 72} \end{aligned}$$

225 $$\begin{aligned} t_3= & {} {1 \over (4\pi ^2)^3} \left[ - {82043\over 27648}\zeta _3 + {564731\over 124416}-{2633\over 31104}(N_f-1)\right] \nonumber \\ \end{aligned}$$

(where $\zeta _3$ is the Riemann zeta-function) provides the matching at the thresholds in the ${\overline{\text {MS}}}$ scheme. While $t_2$, $t_3$ are numerically small coefficients, the charm threshold scale is also relatively low and so there are nonperturbative uncertainties in the matching procedure, which are difficult to estimate but which we assume here to be negligible. Obviously there is no perturbative matching formula across the strange “threshold”; here matching is entirely nonperturbative. Model dependent extrapolations of $\bar{g}^2_{N_f}$ from $N_f=0,2$ to $N_f=3$ were done in the early days of lattice gauge theory. We will include these in our listings of results but not in our estimates, since such extrapolations are based on untestable assumptions.

#### Overview of the review of $\alpha _s$

We begin by explaining lattice-specific difficulties in Sect. 9.2 and the FLAG criteria designed to assess whether the associated systematic uncertainties can be controlled and estimated in a reasonable manner. We then discuss, in Sects. 9.3–9.8, the various lattice approaches. For completeness, we present results from calculations with $N_f = 0, 2, 3$, and 4 flavours. Finally, in Sect. 9.9, we present averages together with our best estimates for $\alpha _{\overline{\mathrm{MS}}}^{(5)}$. These are determined from 3- and 4-flavour QCD simulations. The earlier $N_f = 0, 2$ work obtained results for $N_f = 3$ by extrapolation in $N_f$. Because this is not a theoretically controlled procedure, we do not include these results in our averages. For the $\Lambda$ parameter, we also give results for other number of flavours, including $N_f=0$. Even though the latter numbers should not be used for phenomenology, they represent valuable nonperturbative information concerning field theories with variable numbers of quarks.

#### Differences compared to the FLAG 13 report

For the benefit of the readers who are familiar with our previous report, we list here where changes and additions can be found which go beyond slight improvements of the presentation.

Our criteria are unchanged as far as the explicit ratings on renormalization scale, perturbative behaviour and continuum extrapolation are concerned. However, where we discuss the criteria, we emphasize that it is also important whether finite-size effects and topology sampling are under control. In a few cases, this influences our decision on which computations enter our ranges and averages.

New computations which are reviewed here are

- Karbstein 14 [563] and Bazavov 14 [61] based on the static-quark potential (Sect. 9.4),
- FlowQCD 15 [564] based on a tadpole-improved bare coupling (Sect. 9.6),
- HPQCD 14A [5] based on heavy-quark current 2-point functions (Sect. 9.7).

They influence the final ranges marginally.

### Discussion of criteria for computations entering the averages

As in the PDG review, we only use calculations of $\alpha _s$ published in peer-reviewed journals, and that use NNLO or higher-order perturbative expansions, to obtain our final range in Sect. 9.9. We also, however, introduce further criteria designed to assess the ability to control important systematics which we describe here. Some of these criteria, e.g. that for the continuum extrapolation, are associated with lattice-specific systematics and have no continuum analogue. Other criteria, e.g. that for the renormalization scale, could in principle be applied to nonlattice determinations. Expecting that lattice calculations will continue to improve significantly in the near future, our goal in reviewing the state of the art here is to be conservative and avoid prematurely choosing an overly small range.

In lattice calculations, we generally take ${\mathcal {Q}}$ to be some combination of physical amplitudes or Euclidean correlation functions which are free from UV and IR divergences and have a well-defined continuum limit. Examples include the force between static quarks and 2-point functions of quark bilinear currents.

In comparison to values of observables ${\mathcal {Q}}$ determined experimentally, those from lattice calculations require two more steps. The first step concerns setting the scale $\mu$ in GeV, where one needs to use some experimentally measurable low-energy scale as input. Ideally one employs a hadron mass. Alternatively convenient intermediate scales such as $\sqrt{t_0}$, $w_0$, $r_0$, $r_1$, [136, 245, 246, 565] can be used if their relation to an experimental dimensionful observable is established. The low-energy scale needs to be computed at the same bare parameters where ${\mathcal {Q}}$ is determined, at least as long as one does not use the step-scaling method (see below). This induces a practical difficulty given present computing resources. In the determination of the low-energy reference scale the volume needs to be large enough to avoid finite-size effects. On the other hand, in order for the perturbative expansion of Eq. (216) to be reliable, one has to reach sufficiently high values of $\mu$, i.e. short enough distances. To avoid uncontrollable discretization effects the lattice spacing *a* has to be accordingly small. This means

226 $$\begin{aligned} L \gg \hbox {hadron size}\sim \Lambda _\mathrm{QCD}^{-1}\quad \hbox {and} \quad 1/a \gg \mu , \end{aligned}$$

(where *L* is the box size) and therefore

227 $$\begin{aligned} L/a \ggg \mu /\Lambda _\mathrm{QCD}. \end{aligned}$$

The currently available computer power, however, limits *L* / *a*, typically to $L/a = 20$–64. Unless one accepts compromises in controlling discretization errors or finite-size effects, this means one needs to set the scale $\mu$ according to

228 $$\begin{aligned} \mu \lll L/a \times \Lambda _\mathrm{QCD} \sim 5{-}20\, \hbox {GeV}. \end{aligned}$$

Therefore, $\mu$ can be 1–$3\, \hbox {GeV}$ at most. This raises the concern whether the asymptotic perturbative expansion truncated at 1-loop, 2-loop, or 3-loop in Eq. (216) is sufficiently accurate. There is a finite-size scaling method, usually called step-scaling method, which solves this problem by identifying $\mu =1/L$ in the definition of ${\mathcal {Q}}(\mu )$; see Sect. 9.3.

For the second step after setting the scale $\mu$ in physical units ($\hbox {GeV}$), one should compute ${\mathcal {Q}}$ on the lattice, ${\mathcal {Q}}_\mathrm{lat}(a,\mu )$ for several lattice spacings and take the continuum limit to obtain the left hand side of Eq. (216) as

229 $$\begin{aligned} {\mathcal {Q}}(\mu ) \equiv \lim _{a\rightarrow 0} {\mathcal {Q}}_\mathrm{lat}(a,\mu ) \quad \hbox {with }\mu \ \mathrm{fixed}. \end{aligned}$$

This is necessary to remove the discretization error.

Here it is assumed that the quantity ${\mathcal {Q}}$ has a continuum limit, which is regularization-independent up to discretization errors. The method discussed in Sect. 9.6, which is based on the perturbative expansion of a lattice-regulated, divergent short-distance quantity $W_\mathrm{lat}(a)$ differs in this respect and must be treated separately.

In summary, a controlled determination of $\alpha _s$ needs to satisfy the following:

1. The determination of $\alpha _s$ is based on a comparison of a short-distance quantity ${\mathcal {Q}}$ at scale $\mu$ with a well–defined continuum limit without UV and IR divergences to a perturbative expansion formula in Eq. (216).
2. The scale $\mu$ is large enough so that the perturbative expansion in Eq. (216) is precise to the order at which it is truncated, i.e. it has good *asymptotic* convergence.
3. If ${\mathcal {Q}}$ is defined by physical quantities in infinite volume, one needs to satisfy Eq. (227). Nonuniversal quantities need a separate discussion; see Sect. 9.6.

Conditions 2. and 3. give approximate lower and upper bounds for $\mu$ respectively. It is important to see whether there is a window to satisfy 2. and 3. at the same time. If it exists, it remains to examine whether a particular lattice calculation is done inside the window or not.

Obviously, an important issue for the reliability of a calculation is whether the scale $\mu$ that can be reached lies in a regime where perturbation theory can be applied with confidence. However, the value of $\mu$ does not provide an unambiguous criterion. For instance, the Schrödinger Functional, or SF-coupling (Sect. 9.3) is conventionally taken at the scale $\mu =1/L$, but one could also choose $\mu =2/L$. Instead of $\mu$ we therefore define an effective $\alpha _\mathrm{eff}$. For schemes such as SF (see Sect. 9.3) or *qq* (see Sect. 9.4) this is directly the coupling of the scheme. For other schemes such as the vacuum polarization we use the perturbative expansion Eq. (216) for the observable ${\mathcal {Q}}$ to define

230 $$\begin{aligned} \alpha _\mathrm{eff} = {\mathcal {Q}}/c_1. \end{aligned}$$

If there is an $\alpha _s$-independent term it should first be subtracted. Note that this is nothing but defining an effective, regularization-independent coupling, a physical renormalization scheme.

Let us now comment further on the use of the perturbative series. Since it is only an asymptotic expansion, the remainder $R_n({\mathcal {Q}})={\mathcal {Q}}-\sum _{i\le n}c_i \alpha _s^i$ of a truncated perturbative expression ${\mathcal {Q}}\sim \sum _{i\le n}c_i \alpha _s^i$ cannot just be estimated as a perturbative error $k\,\alpha _s^{n+1}$. The error is nonperturbative. Often one speaks of “nonperturbative contributions”, but nonperturbative and perturbative cannot be strictly separated due to the asymptotic nature of the series (see e.g. Ref. [566]).

Still, we do have some general ideas concerning the size of nonperturbative effects. The known ones such as instantons or renormalons decay for large $\mu$ like inverse powers of $\mu$ and are thus roughly of the form

231 $$\begin{aligned} \exp (-\gamma /\alpha _s), \end{aligned}$$

with some positive constant $\gamma$. Thus we have, loosely speaking,

232 $$\begin{aligned} {\mathcal {Q}}= & {} c_1 \alpha _s + c_2 \alpha _s^2 + \cdots + c_n\alpha _s^n + \mathcal {O}(\alpha _s^{n+1})\nonumber \\&+\, \mathcal {O}(\exp (-\gamma /\alpha _s)). \end{aligned}$$

For small $\alpha _s$, the $\exp (-\gamma /\alpha _s)$ is negligible. Similarly the perturbative estimate for the magnitude of relative errors in Eq. (232) is small; as an illustration for $n=3$ and $\alpha _s = 0.2$ the relative error is ${\sim } 0.8\%$ (assuming coefficients $|c_{n+1} /c_1 | \sim 1$).

For larger values of $\alpha _s$ nonperturbative effects can become significant in Eq. (232). An instructive example comes from the values obtained from $\tau$ decays, for which $\alpha _s\approx 0.3$. Here, different applications of perturbation theory (fixed order, FOPT, and contour improved, CIPT) each look reasonably asymptotically convergent but the difference does not seem to decrease much with the order (see, e.g., the contribution of Pich in Ref. [560]). In addition nonperturbative terms in the spectral function may be nonnegligible even after the integration up to $m_\tau$ (see, e.g., Ref. [567], Golterman in Ref. [560]). All of this is because $\alpha _s$ is not really small.

Since the size of the nonperturbative effects is very hard to estimate one should try to avoid such regions of the coupling. In a fully controlled computation one would like to verify the perturbative behaviour by changing $\alpha _s$ over a significant range instead of estimating the errors as ${\sim } \alpha _s^{n+1}$ . Some computations try to take nonperturbative power ‘corrections’ to the perturbative series into account by including such terms in a fit to the $\mu$ dependence. We note that this is a delicate procedure, both because the separation of nonperturbative and perturbative is theoretically not well defined and because in practice a term like, e.g., $\alpha _s(\mu )^3$ is hard to distinguish from a $1/\mu ^2$ term when the $\mu$-range is restricted and statistical and systematic errors are present. We consider it safer to restrict the fit range to the region where the power corrections are negligible compared to the estimated perturbative error.

The above considerations lead us to the following special criteria for the determination of $\alpha _s$.

- Renormalization scale

all points relevant in the analysis have $\alpha _\mathrm{eff} < 0.2$
all points have $\alpha _\mathrm{eff} < 0.4$ and at least one $\alpha _\mathrm{eff} \le 0.25$
otherwise

- Perturbative behaviour

verified over a range of a factor 4 change in $\alpha _\mathrm{eff}^{n_\mathrm {l}}$ without power corrections or alternatively $\alpha _\mathrm{eff}^{n_\mathrm {l}}=0.01$ is reached
agreement with perturbation theory over a range of a factor 2.25 in $\alpha _\mathrm{eff}^{n_\mathrm {l}}$ possibly fitting with power corrections or alternatively $\alpha _\mathrm{eff}^{n_\mathrm {l}}=0.02$ is reached
otherwise

- Here $n_\mathrm {l}$ is the loop order to which the connection of $\alpha _\mathrm{eff}$ to the ${\overline{\text {MS}}}$ scheme is known. The $\beta$-function of $\alpha _\mathrm{eff}$ is then known to $n_\mathrm {l}+1$ loop order.^63^

- Continuum extrapolation At a reference point of $\alpha _\mathrm{eff} = 0.3$ (or less) we require

three lattice spacings with $\mu a < 1/2$ and full $\mathcal {O}(a)$ improvement, or three lattice spacings with $\mu a \le 1/4$ and 2-loop $\mathcal {O}(a)$ improvement, or $\mu a \le 1/8$ and 1-loop $\mathcal {O}(a)$ improvement
three lattice spacings with $\mu a < 1.5$ reaching down to $\mu a =1$ and full $\mathcal {O}(a)$ improvement, or three lattice spacings with $\mu a \le 1/4$ and one-loop $\mathcal {O}(a)$ improvement
otherwise

- Finite-size effects
  These are a less serious issue for the determination of $\alpha _s$ since one looks at short-distance observables where such effects are expected to be suppressed. We therefore have no special criterion in our tables, but do check that volumes are not too small and in particular the scale is determined in large enough volume.^64^ Remarks are added in the text when appropriate.
- Topology sampling
  In principle a good way to improve the quality of determinations of $\alpha _s$ is to push to very small lattice spacings thus enabling large $\mu$. It is known that the sampling of field space becomes very difficult for the HMC algorithm when the lattice spacing is small and one has the standard periodic boundary conditions. In practice, for all known discretizations the topological charge slows down dramatically for $a\approx 0.05\,\mathrm{fm}$ and smaller [68, 71–75, 351]. Open boundary conditions solve the problem [76] but are rarely used. Since the effect of the freezing is generally not known, we also do need to pay attention to this issue. Remarks are added in the text when appropriate.

We assume that quark-mass effects of light quarks (including strange) are negligible in the effective coupling itself where large, perturbative, $\mu$ is considered.

We also need to specify what is meant by $\mu$. Here are our choices:233where *q* is the magnitude of the momentum and $\bar{m}_\mathrm {h}$ the heavy-quark mass. We note again that the above criteria cannot be applied when regularization dependent quantities $W_\mathrm {lat}(a)$ are used instead of ${\mathcal {O}}(\mu )$. These cases are specifically discussed in Sect. 9.6.

A popular scale choice is the intermediate $r_0$ scale, although one should also bear in mind that its determination from physical observables has also to be taken into account. The phenomenological value of $r_0$ was originally determined as $r_0 \approx 0.49\,\hbox {fm}$ through potential models describing quarkonia [136]. Recent determinations from 2-flavour QCD are $r_0 = 0.420(14)$–$0.450(14)\,\hbox {fm}$ by the ETM Collaboration [32, 36], using as input $f_\pi$ and $f_K$ and carrying out various continuum extrapolations. On the other hand, the ALPHA Collaboration [12] determined $r_0 = 0.503(10)\,\hbox {fm}$ with input from $f_K$, and the QCDSF Collaboration [568] cites $0.501(10)(11)\,\hbox {fm}$ from the mass of the nucleon (no continuum limit). Recent determinations from 3-flavour QCD are consistent with $r_1 = 0.313(3)\,\hbox {fm}$ and $r_0 = 0.472(5)\,\hbox {fm}$ [29, 250, 569]. Due to the uncertainty in these estimates, and as many results are based directly on $r_0$ to set the scale, we shall often give both the dimensionless number $r_0 \Lambda _{\overline{\mathrm{MS}}}$ and $\Lambda _{\overline{\mathrm{MS}}}$. In the cases where no physical $r_0$ scale is given in the original papers or we convert to the $r_0$ scale, we use the value $r_0=0.472\,\mathrm{fm}$. In the case $r_1 \Lambda _{\overline{\mathrm{MS}}}$ is given in the publications, we use $r_0 /r_1 = 1.508$ [569] to convert, neglecting the error on this ratio. In some, mostly early, computations the string tension, $\sqrt{\sigma }$ was used. We convert to $r_0$ using $r_0^2\sigma = 1.65-\pi /12$, which has been shown to be an excellent approximation in the relevant pure gauge theory [570, 571]. The new scales $t_0,w_0$ based on the Wilson flow are very attractive alternatives to $r_0$ but have not yet been used as much and their discretization errors are still under discussion [572–575]. We remain with $r_0$ as our main reference scale for now.

The attentive reader will have noticed that bounds such as $\mu a < 1.5$ or at least one value of $\alpha _{\mathrm {eff}}\le 0.25$ which we require for a  are not very stringent. There is a considerable difference between  and . We have chosen the above bounds, unchanged as compared to FLAG 13, since not too many computations would satisfy more stringent ones at present. Nevertheless, we believe that the  criteria already give reasonable bases for estimates of systematic errors. In the future, we expect that we will be able to tighten our criteria for inclusion in the average, and that many more computations will reach the present  rating in one or more categories.

In principle one should also account for electroweak radiative corrections. However, both in the determination of $\alpha _\mathrm {s}$ at intermediate scales $\mu$ and in the running to high scales, we expect electroweak effects to be much smaller than the presently reached precision. Such effects are therefore not further discussed.

### $\alpha _s$ from the Schrödinger functional

#### General considerations

The method of step-scaling functions avoids the scale problem, Eq. (226). It is in principle independent of the particular boundary conditions used and was first developed with periodic boundary conditions in a two-dimensional model [576]. However, at present most applications in QCD use Schrödinger functional boundary conditions [153, 577]. An important reason is that these boundary conditions avoid zero modes for the quark fields and quartic modes [578] in the perturbative expansion in the gauge fields. Furthermore the corresponding renormalization scheme is well studied in perturbation theory [579–581] with the 3-loop $\beta$-function and two-loop cutoff effects (for the standard Wilson regularization) known.

**Table 42:** Results for the $\Lambda$ parameter from computations using step scaling of the SF-coupling. Entries without values for $\Lambda$ computed the running and established perturbative behaviour at large $\mu$

Collaboration	Refs.	$N_{ f}$	Publication status	Renormalization scale	Perturbative behaviour	Continuum extrapolation	Scale	$\Lambda _{\overline{\text {MS}}}[\,\mathrm {MeV}]$	$r_0\Lambda _{\overline{\text {MS}}}$
ALPHA 10A	[586]	4	A				Only running of $\alpha _s$ in Fig. 4		
Perez 10	[587]	4	P				Only step-scaling function in Fig. 4		
PACS-CS 09A	[62]	$2+1$	A				$m_\rho$	$371(13)(8)(^{+0}_{-27})^{\mathrm{a}}$	$0.888(30)(18)(^{+0}_{-65})^{\mathrm{b}}$
			A				$m_\rho$	$345(59)^{\mathrm{c}}$	$0.824(141)^{\mathrm{b}}$
ALPHA 12$^{\mathrm{d}}$	[12]	2	A				$f_\mathrm{K}$	310(20)	0.789(52)
ALPHA 04	[588]	2	A				$r_0 = 0.5~\hbox {fm}^{\mathrm{e}}$	$245(16)(16)^{\mathrm{e}}$	$0.62(2)(2)^{\mathrm{e}}$
ALPHA 01A	[589]	2	A				Only running of $\alpha _s$ in Fig. 5		
CP-PACS 04$^{\mathrm{f}}$	[582]	0	A				Only tables of $g^2_\mathrm{SF}$		
ALPHA 98$^{\mathrm{g}}$	[590]	0	A				$r_0=0.5~\mathrm{fm}$	238(19)	0.602(48)
Lüscher 93	[579]	0	A				$r_0=0.5~\mathrm{fm}$	233(23)	0.590(60)$^{\mathrm{h}}$

$^{\mathrm{a}}$ Result with a constant (in *a*) continuum extrapolation of the combination $L_{\mathrm {max}}m_\rho$

$^{\mathrm{b}}$ In conversion to $r_0\Lambda _{\overline{\mathrm{MS}}}$, $r_0$ is taken to be $0.472~\hbox {fm}$

$^{\mathrm{c}}$ Result with a linear continuum extrapolation in *a* of the combination $L_{\mathrm {max}}m_\rho$

$$^{\mathrm{d}}$$ Supersedes ALPHA 04

$^{\mathrm{e}}$ The $N_f=2$ results were based on values for $r_0/a$ which have later been found to be too small by [12]. The effect will be of the order of 10–15%, presumably an increase in $\Lambda r_0$. We have taken this into account by a  in the renormalization scale

$^{\mathrm{f}}$ This investigation was a precursor for PACS-CS 09A and confirmed two step-scaling functions as well as the scale setting of ALPHA 98

$^{\mathrm{g}}$ Uses data of Lüscher 93 and therefore supersedes it

$$^{\mathrm{h}}$$ Converted from $$\alpha _{\overline{\text {MS}}}(37r_0^{-1})=0.1108(25)$$

Let us first briefly review the step-scaling strategy. The essential idea is to split the determination of the running coupling at large $\mu$ and of a hadronic scale into two lattice calculations and connect them by ‘step scaling’. In the former part, we determine the running coupling constant in a finite-volume scheme, in practice a ‘Schrödinger Functional (SF) scheme’ in which the renormalization scale is set by the inverse lattice size $\mu = 1/L$. In this calculation, one takes a high renormalization scale while keeping the lattice spacing sufficiently small as

234 $$\begin{aligned} \mu \equiv 1/L \sim 10\,\ldots \, 100\,\hbox {GeV},\quad a/L \ll 1. \end{aligned}$$

In the latter part, one chooses a certain $\bar{g}^2_{\mathrm {max}}=\bar{g}^2(1/L_{\mathrm {max}})$, typically such that $L_{\mathrm {max}}$ is around $0.5\,\mathrm{fm}$. With a common discretization, one then determines $L_{\mathrm {max}}/a$ and (in a large volume $L \ge 2$–$3\,\hbox {fm}$) a hadronic scale such as a hadron mass, $\sqrt{t_0}/a$ or $r_0/a$ at the same bare parameters. In this way one gets numbers for $L_{\mathrm {max}}/r_0$ and by changing the lattice spacing *a* carries out a continuum limit extrapolation of that ratio.

In order to connect $\bar{g}^2(1/L_{\mathrm {max}})$ to $\bar{g}^2(\mu )$ at high $\mu$, one determines the change of the coupling in the continuum limit when the scale changes from *L* to *L* / 2, starting from $L=L_\mathrm{max}$ and arriving at $\mu = 2^k /L_\mathrm{max}$. This part of the strategy is called step scaling. Combining these results yields $\bar{g}^2(\mu )$ at $\mu = 2^k {r_0 \over L_{\mathrm {max}}} r_0^{-1}$, where $r_0$ stands for the particular chosen hadronic scale.

In order to have a perturbatively well-defined scheme, the SF scheme uses Dirichlet boundary condition at time $t = 0$ and $t = T$. These break translation invariance and permit ${\mathcal {O}}(a)$ counter terms at the boundary through quantum corrections. Therefore, the leading discretization error is ${\mathcal {O}}(a)$. Improving the lattice action is achieved by adding counter terms at the boundaries whose coefficients are denoted as $c_t,\tilde{c}_t$. In practice, these coefficients are computed with 1-loop or 2-loop perturbative accuracy. A better precision in this step yields a better control over discretization errors, which is important, as can be seen, e.g., in Refs. [570, 582]. The finite $c^{(i)}_g$, Eq. (221), are known for $i=1,2$ [580, 581].

Also computations with Dirichlet boundary conditions do in principle suffer from the insufficient change of topology in the HMC algorithm at small lattice spacing. However, in a small volume the weight of nonzero charge sectors in the path integral is exponentially suppressed [583]^65^ and one practically should not sample any nontrivial topology. Considering the suppression quantitatively Ref. [584] finds a strong suppression below $L\approx 0.8\,\mathrm{fm}$. Therefore the lack of topology change of the HMC is not a real issue in the computations discussed here. A mix of Dirichlet and open boundary conditions is expected to remove this worry [585] and may be considered in the future.

#### Discussion of computations

In Table 42 we give results from various determinations of the $\Lambda$ parameter. For a clear assessment of the $N_f$ dependence, the last column also shows results that refer to a common hadronic scale, $r_0$. As discussed above, the renormalization scale can be chosen large enough such that $\alpha _s < 0.2$ and the perturbative behaviour can be verified. Consequently only  is present for these criteria except for early work where the $n_l=2$ loop connection to ${\overline{\text {MS}}}$ was not yet known. With dynamical fermions, results for the step-scaling functions are always available for at least $a/L = \mu a =1/4,1/6, 1/8$. All calculations have a nonperturbatively $\mathcal {O}(a)$ improved action in the bulk. For the discussed boundary $\mathcal {O}(a)$ terms this is not so. In most recent calculations two-loop $\mathcal {O}(a)$ improvement is employed together with at least three lattice spacings.^66^ This means a  for the continuum extrapolation. In the other contributions only one-loop $c_t$ was available and we arrive at . We note that the discretization errors in the step-scaling functions are usually found to be very small, at the percent level or below. However, the overall desired precision is very high as well, and the results in CP-PACS 04 [582] show that discretization errors at the below percent level cannot be taken for granted. In particular with staggered fermions (unimproved except for boundary terms) few percent effects are seen in Perez 10 [587].

In the work by PACS-CS 09A [62], the continuum extrapolation in the scale setting is performed using a constant function in *a* and with a linear function. Potentially the former leaves a considerable residual discretization error. We here use, as discussed with the collaboration, the continuum extrapolation linear in *a*, as given in the second line of PACS-CS 09A [62] results in Table 42.

A single computation, PACS-CS 09A [62], quotes also $\alpha _{\overline{\text {MS}}}(M_Z)$. We take the linear continuum extrapolation as discussed above:

235 $$\begin{aligned} \alpha _{\overline{\text {MS}}}^{(5)}(M_Z)=0.118(3), \end{aligned}$$

where the conversion from a 3-flavour result to 5-flavours was done perturbatively (see Sect. 9.2). Other results do not have a sufficient number of quark flavours (ALPHA 10A [586], Perez 10 [587]) or do not yet contain the conversion of the scale to physical units. Thus no value for $\alpha _{\overline{\text {MS}}}^{(5)}(M_Z)$ is quoted.

More results for $\alpha _{\overline{\text {MS}}}^{(5)}(M_Z)$ using step-scaling functions can be expected soon. Their precision is likely to be much better than what we were able to report on here. A major reason is the use of the gradient flow [245] in definitions of finite-volume schemes [591, 592].

### $\alpha _s$ from the potential at short distances

#### General considerations

The basic method was introduced in Ref. [593] and developed in Ref. [594]. The force or potential between an infinitely massive quark and antiquark pair defines an effective coupling constant via

236 $$\begin{aligned} F(r) = {\mathrm{d} V(r) \over \mathrm{d}r} = C_F {\alpha _\mathrm {qq}(r) \over r^2}. \end{aligned}$$

The coupling can be evaluated nonperturbatively from the potential through a numerical differentiation; see below. In perturbation theory one also defines couplings in different schemes $\alpha _{\bar{V}}$, $\alpha _V$ via

237 $$\begin{aligned} V(r) = - C_F {\alpha _{\bar{V}}(r) \over r},\quad \hbox {or}\quad \tilde{V}(Q) = - C_F {\alpha _V(Q) \over Q^2}, \end{aligned}$$

where one fixes the unphysical constant in the potential by $\lim _{r\rightarrow \infty }V(r)=0$ and $\tilde{V}(Q)$ is the Fourier transform of *V*(*r*). Nonperturbatively, the subtraction of a constant in the potential introduces an additional renormalization constant, the value of $V(r_{\mathrm {ref}})$ at some distance $r_{\mathrm {ref}}$. Perturbatively, it is believed to entail a renormalon ambiguity. In perturbation theory, these definitions are all simply related to each other, and their perturbative expansions are known including the $\alpha _s^4$ and $\alpha _s^5 \log \alpha _s$ terms [595–602].

The potential *V*(*r*) is determined from ratios of Wilson loops, *W*(*r*, *t*), which behave as

238 $$\begin{aligned} \langle W(r, t) \rangle = |c_0|^2 e^{-V(r)t} + \sum _{n\not = 0} |c_n|^2 e^{-V_n(r)t}, \end{aligned}$$

where *t* is taken as the temporal extension of the loop, *r* is the spatial one and $V_n$ are excited-state potentials. To improve the overlap with the ground state, and to suppress the effects of excited states, *t* is taken large. Also various additional techniques are used, such as a variational basis of operators (spatial paths) to help in projecting out the ground state. Furthermore some lattice-discretization effects can be reduced by averaging over Wilson loops related by rotational symmetry in the continuum.

In order to reduce discretization errors it is of advantage to define the numerical derivative giving the force as

239 $$\begin{aligned} F(r_\mathrm {I}) = { V(r) - V(r-a) \over a }, \end{aligned}$$

where $r_\mathrm {I}$ is chosen so that at tree level the force is the continuum force. $F(r_\mathrm {I})$ is then a ‘tree-level improved’ quantity and similarly the tree-level improved potential can be defined [603].

Lattice potential results are in position space, while perturbation theory is naturally computed in momentum space at large momentum. Usually, the Fourier transform is then taken of the perturbation expansion to match to the lattice data.

Finally, as was noted in Sect. 9.2, a determination of the force can also be used to determine the $r_0$ scale, by defining it from the static force by

240 $$\begin{aligned} r_0^2 F(r_0) = {1.65}, \end{aligned}$$

and with $r_1^2 F(r_1) = 1$ the $r_1$ scale.

#### Discussion of computations

In Table 43, we list results of determinations of $r_0\Lambda _{{\overline{\text {MS}}}}$ (together with $\Lambda _{{\overline{\text {MS}}}}$ using the scale determination of the authors). Since the last review, FLAG 13, there have been two new computations, Karbstein 14 [563] and Bazavov 14 [61].

**Table 43:** Short-distance potential results

Collaboration	Refs.	$N_f$	Publication status	Renormalization scale	Perturbative behaviour	Continuum extrapolation	Scale	$\Lambda _{\overline{\text {MS}}}[\,\mathrm {MeV}]$	$r_0\Lambda _{\overline{\text {MS}}}$
Bazavov 14	[61]	$2+1$	A				$r_1 = 0.3106(17)~\hbox {fm}^\mathrm{a}$	$315(^{+18}_{-12})^\mathrm{b}$	$0.746(^{+42}_{-27})$
Bazavov 12	[604]	$2+1$	A	$^{\mathrm{c}}$		$^{\mathrm{d}}$	$r_0 = 0.468~\hbox {fm}$	$295(30)^{\mathrm{e}}$	$0.70(7)^{\mathrm{f}}$
Karbstein 14	[563]	2	A				$r_0 = 0.42~\hbox {fm}$	331(21)	0.692(31)
ETM 11C	[605]	2	A				$r_0 = 0.42~\hbox {fm}$	$315(30)^{\mathrm{g}}$	0.658(55)
Brambilla 10	[606]	0	A			$^{\mathrm{h}}$		$266(13)^{\mathrm{i}}$	$0.637(^{+32}_{-30})^{\mathrm{h}}$
UKQCD 92	[594]	0	A		$^{\mathrm{j}}$		$\sqrt{\sigma }=0.44~\,\mathrm {GeV}$	256(20)	0.686(54)
Bali 92	[607]	0	A		$^{\mathrm{j}}$		$\sqrt{\sigma }=0.44~\,\mathrm {GeV}$	247(10)	0.661(27)

$^\mathrm{a}$ Determination on lattices with $m_\pi L=2.2$–2.6. About 10 changes of topological charge on the finest lattice [351]. Scale from $r_1$ [351] as determined from $f_\pi$ in Ref. [29]

$$^\mathrm{b}$$ $$\alpha ^{(3)}_{\overline{\mathrm{MS}}}(1.5~\hbox {GeV}) = 0.336(^{+12}_{-8})$$, $$\alpha ^{(5)}_{\overline{\mathrm{MS}}}(M_Z) = 0.1166(^{+12}_{-8})$$

$^{\mathrm{c}}$ Since values of $\alpha _{\mathrm {eff}}$ within our designated range are used, we assign a  despite values of $\alpha _{\mathrm {eff}}$ up to $\alpha _{\mathrm {eff}}=0.5$ being used

$^{\mathrm{d}}$ Since values of 2*a* / *r* within our designated range are used, we assign a  although only values of $2a/r\ge 1.14$ are used at $\alpha _{\mathrm {eff}}=0.3$

$^{\mathrm{e}}$ Using results from Ref. [569]

$$^{\mathrm{f}}$$ $$\alpha ^{(3)}_{\overline{\mathrm{MS}}}(1.5~\hbox {GeV}) = 0.326(19)$$, $$\alpha ^{(5)}_{\overline{\mathrm{MS}}}(M_Z) = 0.1156(^{+21}_{-22})$$

$^{\mathrm{g}}$ Both potential and $r_0/a$ are determined on a small ($L=3.2r_0$) lattice

$^{\mathrm{h}}$ Uses lattice results of Ref. [570], some of which have very small lattice spacings where according to more recent investigations a bias due to the freezing of topology may be present

$^{\mathrm{i}}$ Only $r_0\Lambda _{\overline{\mathrm{MS}}}$ is given, our conversion using $r_0 = 0.472~\hbox {fm}$

$^{\mathrm{j}}$ We give a  because only a NLO formula is used and the error bars are very large; our criterion does not apply well to these very early calculations

The first determinations in the three-colour Yang Mills theory are by UKQCD 92 [594] and Bali 92 [607] who used $\alpha _\mathrm {qq}$ as explained above, but not in the tree-level improved form. Rather a phenomenologically determined lattice-artefact correction was subtracted from the lattice potentials. The comparison with perturbation theory was on a more qualitative level on the basis of a two-loop $\beta$-function ($n_l=1$) and a continuum extrapolation could not be performed as yet. A much more precise computation of $\alpha _\mathrm {qq}$ with continuum extrapolation was performed in Refs. [570, 603]. Satisfactory agreement with perturbation theory was found [603] but the stability of the perturbative prediction was not considered sufficient to be able to extract a $\Lambda$ parameter.

In Brambilla 10 [606] the same quenched lattice results of Ref. [603] were used and a fit was performed to the continuum potential, instead of the force. Perturbation theory to $n_l=3$ loop was used including a resummation of terms $\alpha _s^3 (\alpha _s \ln \alpha _s)^n$ and $\alpha _s^4 (\alpha _s \ln \alpha _s)^n$. Close agreement with perturbation theory was found when a renormalon subtraction was performed. Note that the renormalon subtraction introduces a second scale into the perturbative formula which is absent when the force is considered.

Bazavov 14 [61] is an update of Bazavov 12 [604] and modify this procedure somewhat. They consider the well-defined perturbative expansion for the force, where renormalon problems disappear. They set $\mu = 1/r$ to eliminate logarithms and then integrate the force to obtain an expression for the potential. The resulting integration constant is fixed by requiring the perturbative potential to be equal to the nonperturbative one exactly at a reference distance $r_\mathrm{ref}$ and the two are then compared at other values of *r*. As a further check, the force is also used directly.

For the quenched calculation Brambilla 10 [606] very small lattice spacings were available, $a \sim 0.025\,\hbox {fm}$, [603]. For ETM 11C [605], Bazavov 12 [604], Karbstein 14 [563] and Bazavov 14 [61] using dynamical fermions such small lattice spacings are not yet realized (Bazavov 14 reaches down to $a \sim 0.041\,\hbox {fm}$). They all use the tree-level improved potential as described above. We note that the value of $\Lambda _{\overline{\text {MS}}}$ in physical units by ETM 11C [605] is based on a value of $r_0=0.42$ fm. This is at least 10% smaller than the large majority of other values of $r_0$. Also the value of $r_0/a$ or $r_1/a$ on the finest lattices in ETM 11C [605] and Bazavov 14 [61] come from rather small lattices with $m_\pi L \approx 2.4$, 2.2 respectively.

Instead of the procedure discussed previously, Karbstein 14 [563] reanalyses the data of ETM 11C [605] by first estimating the Fourier transform $\tilde{V}(p)$ of *V*(*r*) and then fits the perturbative expansion of $\tilde{V}(p)$ in terms of $\alpha _{\overline{\text {MS}}}(p)$. Of course, the Fourier transform cannot be computed without modelling the *r*-dependence of *V*(*r*) at short and at large distances. The authors fit a linearly rising potential at large distances together with string-like corrections of order $r^{-n}$ and define the potential at large distances by this fit.^67^ Recall that for observables in momentum space we take the renormalization scale entering our criteria as $\mu =p$, Eq. (233). The analysis (as in ETM 11C [605]) is dominated by the data at the smallest lattice spacing, where a controlled determination of the overall scale is difficult due to possible finite-size effects.

**Table 44:** Vacuum polarization results

Collaboration	Refs.	$N_{ f}$	Publication status	Renormalization scale	Perturbative behaviour	Continuum extrapolation	Scale	$\Lambda _{\overline{\text {MS}}}[\,\mathrm {MeV}]$	$r_0\Lambda _{\overline{\text {MS}}}$
JLQCD 10	[613]	$2+1$	A				$r_0 = 0.472~\hbox {fm}$	$247(5)^{\mathrm{a}}$	0.591(12)
JLQCD/TWQCD 08C	[614]	2	A				$r_0 = 0.49~\hbox {fm}$	$234(9)(^{+16}_{-0})$	$0.581(22)(^{+40}_{-0})$

$$^{\mathrm{a}}$$ $$\alpha _{\overline{\text {MS}}}^{(5)}(M_Z)=0.1118(3)(^{+16}_{-17})$$

One of the main issues for all these computations is whether the perturbative running of the coupling constant has been reached. While for quenched or $N_f=0$ fermions this seems to be the case at the smallest distances, for dynamical fermions at present there is no consensus. Brambilla 10 [606], Bazavov 12 [604] and Bazavov 14 [61] report good agreement with perturbation theory after the renormalon is subtracted or eliminated, but Ref. [608] uses the force directly, where no renormalon contributes, and finds that far shorter distances are needed than are presently accessible for dynamical fermion simulations in order to match to perturbation theory. Further work is needed to clarify this point.

A second issue is the coverage of configuration space in some of the simulations, which use very small lattice spacings with periodic boundary conditions. Affected are the smallest two lattice spacings of Bazavov 14 [61] where very few tunnellings of the topological charge occur [351]. With present knowledge, it also seems possible that the older data by Refs. [570, 603] used by Brambilla 10 [606] are partially done in (close to) frozen topology.

### $\alpha _s$ from the vacuum polarization at short distances

#### General considerations

The vacuum polarization function for the flavour nonsinglet currents $J^a_\mu$ ($a=1,2,3$) in the momentum representation is parameterized as

241 $$\begin{aligned} \langle J^a_\mu J^b_\nu \rangle= & {} \delta ^{ab} [(\delta _{\mu \nu }Q^2 - Q_\mu Q_\nu ) \Pi ^{(1)}(Q)\nonumber \\&-\, Q_\mu Q_\nu \Pi ^{(0)}(Q)], \end{aligned}$$

where $Q_\mu$ is a space like momentum and $J_\mu \equiv V_\mu$ for a vector current and $J_\mu \equiv A_\mu$ for an axial-vector current. Defining $\Pi _J(Q)\equiv \Pi _J^{(0)}(Q)+\Pi _J^{(1)}(Q)$, the operator product expansion (OPE) of the vacuum polarization function $\Pi _{V+A}(Q)=\Pi _V(Q)+\Pi _A(Q)$ is given by

242 $$\begin{aligned} {\Pi _{V+A}|_\mathrm{OPE}(Q^2,\alpha _s)}= & {} c + C_1(Q^2) + C_m^{V+A}(Q^2) \frac{\bar{m}^2(Q)}{Q^2}\nonumber \\&+\, \sum _{q=u,d,s}C_{\bar{q}q}^{V+A}(Q^2) \frac{\langle m_q\bar{q}q \rangle }{Q^4}\nonumber \\&+\, C_{GG}(Q^2) \frac{\langle \alpha _s GG\rangle }{Q^4}+{\mathcal {O}}(Q^{-6}),\nonumber \\ \end{aligned}$$

for large $Q^2$. $C_X^{V+A}(Q^2)=\sum _{i\ge 0}( C_X^{V+A})^{(i)}\alpha _s^i(Q^2)$ are the perturbative coefficient functions for the operators *X* ($X=1$, $\bar{q}q$, *GG*) and $\bar{m}$ is the running mass of the mass-degenerate up and down quarks. $C_1$ is known including $\alpha _s^4$ in a continuum renormalization scheme such as the $\overline{\mathrm{MS}}$ scheme [609–611]. Nonperturbatively, there are terms in $C_X$ which do not have a series expansion in $\alpha _s$. For an example for the unit operator see Ref. [612]. The term *c* is *Q*–independent and divergent in the limit of infinite ultraviolet cutoff. However the Adler function defined as

243 $$\begin{aligned} D(Q^2) \equiv - Q^2 { \mathrm{d}\Pi (Q^2) \over \mathrm{d}Q^2}, \end{aligned}$$

is a scheme-independent finite quantity. Therefore one can determine the running coupling constant in the $\overline{\mathrm{MS}}$ scheme from the vacuum polarization function computed by a lattice-QCD simulation. In more detail, the lattice data of the vacuum polarization is fitted with the perturbative formula Eq. (242) with fit parameter $\Lambda _{\overline{\mathrm{MS}}}$ parameterizing the running coupling $\alpha _{\overline{\mathrm{MS}}}(Q^2)$.

While there is no problem in discussing the OPE at the nonperturbative level, the ‘condensates’ such as ${\langle \alpha _s GG\rangle }$ are ambiguous, since they mix with lower-dimensional operators including the unity operator. Therefore one should work in the high-$Q^2$ regime where power corrections are negligible within the given accuracy. Thus setting the renormalization scale as $\mu \equiv \sqrt{Q^2}$, one should seek, as always, the window $\Lambda _\mathrm{QCD} \ll \mu \ll a^{-1}$.

#### Discussion of computations

Results using this method are, to date, only available using overlap fermions. These are collected in Table 44 for $N_f=2$, JLQCD/TWQCD 08C [614] and for $N_f = 2+1$, JLQCD 10 [613]. At present, only one lattice spacing $a \approx 0.11\,\hbox {fm}$ has been simulated.

The fit to Eq. (242) is done with the 4-loop relation between the running coupling and $\Lambda _{\overline{\text {MS}}}$. It is found that without introducing condensate contributions, the momentum scale where the perturbative formula gives good agreement with the lattice results is very narrow, $aQ \simeq 0.8$–1.0. When condensate contributions are included the perturbative formula gives good agreement with the lattice results for the extended range $aQ \simeq 0.6$–1.0. Since there is only a single lattice spacing there is a  for the continuum limit. The renormalization scale $\mu$ is in the range of $Q=1.6$–$2\,\hbox {GeV}$. Approximating $\alpha _\mathrm{eff}\approx \alpha _{\overline{\mathrm{MS}}}(Q)$, we estimate that $\alpha _\mathrm{eff}=0.25$–0.30 for $N_f=2$ and $\alpha _\mathrm{eff}=0.29$–0.33 for $N_f=2+1$. Thus we give a  and  for $N_{ f}=2$ and $N_{ f}=2+1$ respectively for the renormalization scale and a  for the perturbative behaviour.

We note that more investigations of this method are in progress [615].

### $\alpha _s$ from observables at the lattice-spacing scale

#### General considerations

The general method is to evaluate a short-distance quantity ${\mathcal {Q}}$ at the scale of the lattice spacing ${\sim } 1/a$ and then determine its relationship to $\alpha _{\overline{\mathrm{MS}}}$ via a power series expansion.

This is epitomized by the strategy of the HPQCD Collaboration [616, 617], discussed here for illustration, which computes and then fits to a variety of short-distance quantities, *Y*,

244 $$\begin{aligned} Y = \sum _{n=1}^{n_\mathrm{max}} c_n \alpha _\mathrm {V'}^n(q^*) \,. \end{aligned}$$

*Y* is taken as the logarithm of small Wilson loops (including some nonplanar ones), Creutz ratios, ‘tadpole-improved’ Wilson loops and the tadpole-improved or ‘boosted’ bare coupling ($\mathcal {O}(20)$ quantities in total). $c_n$ are perturbative coefficients (each depending on the choice of *Y*) known to $n = 3$ with additional coefficients up to $n_\mathrm{max}$ being numerically fitted. $\alpha _\mathrm {V'}$ is the running coupling constant related to $\alpha _\mathrm {V}$ from the static-quark potential (see Sect. 9.4).^68^

The coupling constant is fixed at a scale $q^* = d/a$. This is chosen as the mean value of $\ln q$ with the one gluon loop as measure [618, 619]. (Thus a different result for *d* is found for every short-distance quantity.) A rough estimate yields $d \approx \pi$, and in general the renormalization scale is always found to lie in this region.

For example for the Wilson loop $W_{mn} \equiv \langle W(ma,na) \rangle$ we have

245 $$\begin{aligned} \ln \left( \frac{W_{mn}}{u_0^{2(m+n)}}\right)= & {} c_1 \alpha _\mathrm {V'}(q^*) + c_2 \alpha _\mathrm {V'}^2(q^*) \nonumber \\&+\, c_3 \alpha _\mathrm {V'}^3(q^*) + \cdots , \end{aligned}$$

for the tadpole-improved version, where $c_1$, $c_2, \ldots$ are the appropriate perturbative coefficients and $u_0 = W_{11}^{1/4}$. Substituting the nonperturbative simulation value in the left hand side, we can determine $\alpha _\mathrm {V'}(q^*)$, at the scale $q^*$. Note that one finds empirically that perturbation theory for these tadpole-improved quantities have smaller $c_n$ coefficients and so the series has a faster apparent convergence.

Using the $\beta$-function in the $\mathrm V'$ scheme, results can be run to a reference value, chosen as $\alpha _0 \equiv \alpha _\mathrm {V'}(q_0)$, $q_0 = 7.5\,\hbox {GeV}$. This is then converted perturbatively to the continuum ${\overline{\text {MS}}}$ scheme

246 $$\begin{aligned} \alpha _{\overline{\mathrm{MS}}}(q_0) = \alpha _0 + d_1 \alpha _0^2 + d_2 \alpha _0^3 + \cdots , \end{aligned}$$

where $d_1, d_2$ are known one and two loop coefficients.

Other collaborations have focussed more on the bare ‘boosted’ coupling constant and directly determined its relationship to $\alpha _{\overline{\mathrm{MS}}}$. Specifically, the boosted coupling is defined by

247 $$\begin{aligned} \alpha _\mathrm {P}(1/a) = {1\over 4\pi } {g_0^2 \over u_0^4}, \end{aligned}$$

again determined at a scale ${\sim } 1/a$. As discussed previously since the plaquette expectation value in the boosted coupling contains the tadpole diagram contributions to all orders, which are dominant contributions in perturbation theory, there is an expectation that the perturbation theory using the boosted coupling has smaller perturbative coefficients [618], and hence smaller perturbative errors.

#### Continuum limit

Lattice results always come along with discretization errors, which one needs to remove by a continuum extrapolation. As mentioned previously, in this respect the present method differs in principle from those in which $\alpha _s$ is determined from physical observables. In the general case, the numerical results of the lattice simulations at a value of $\mu$ fixed in physical units can be extrapolated to the continuum limit, and the result can be analysed as to whether it shows perturbative running as a function of $\mu$ in the continuum. For observables at the cutoff-scale ($q^*=d/a$), discretization effects cannot easily be separated out from perturbation theory, as the scale for the coupling comes from the lattice spacing. Therefore the restriction $a\mu \ll 1$ (the ‘continuum extrapolation’ criterion) is not applicable here. Discretization errors of order $a^2$ are, however, present. Since $a\sim \exp (-1/(2b_0 g_0^2)) \sim \exp (-1/(8\pi b_0 \alpha (q^*))$, these errors now appear as power corrections to the perturbative running, and have to be taken into account in the study of the perturbative behaviour, which is to be verified by changing *a*. One thus usually fits with power corrections in this method.

In order to keep a symmetry with the ‘continuum extrapolation’ criterion for physical observables and to remember that discretization errors are, of course, relevant, we replace it here by one for the lattice spacings used:

- Lattice spacings
3 or more lattice spacings, at least 2 points below $a = 0.1\,\hbox {fm}$
two lattice spacings, at least 1 point below $a = 0.1\,\hbox {fm}$
otherwise

#### Discussion of computations

Note that due to $\mu \sim 1/a$ being relatively large the results easily have a  or  in the rating on renormalization scale.

The work of El-Khadra 92 [620] employs a one-loop formula to relate $\alpha ^{(0)}_{\overline{\mathrm{MS}}}(\pi /a)$ to the boosted coupling for three lattice spacings $a^{-1} = 1.15$, 1.78, $2.43\,\hbox {GeV}$. (The lattice spacing is determined from the charmonium 1S-1P splitting.) They obtain $\Lambda ^{(0)}_{\overline{\mathrm{MS}}}=234\,\hbox {MeV}$, corresponding to $\alpha _\mathrm{eff} = \alpha ^{(0)}_{\overline{\mathrm{MS}}}(\pi /a) \approx 0.15$–0.2. The work of Aoki 94 [621] calculates $\alpha ^{(2)}_V$ and $\alpha ^{(2)}_{\overline{\mathrm{MS}}}$ for a single lattice spacing $a^{-1}\sim 2\,\hbox {GeV}$ again determined from charmonium 1S-1P splitting in 2-flavour QCD. Using one-loop perturbation theory with boosted coupling, they obtain $\alpha ^{(2)}_V=0.169$ and $\alpha ^{(2)}_{\overline{\mathrm{MS}}}=0.142$. Davies 94 [622] gives a determination of $\alpha _\mathrm {V}$ from the expansion

248 $$\begin{aligned} -\ln W_{11}\equiv & {} \frac{4\pi }{3}\alpha _\mathrm {V}^{(N_f)}(3.41/a)\nonumber \\&\times [1 - (1.185+0.070N_f)\alpha _\mathrm {V}^{(N_f)}], \end{aligned}$$

neglecting higher-order terms. They compute the $\Upsilon$ spectrum in $N_f=0$, 2 QCD for single lattice spacings at $a^{-1} = 2.57$, $2.47\,\hbox {GeV}$ and obtain $\alpha _\mathrm {V}(3.41/a)\simeq 0.1$5, 0.18 respectively. Extrapolating the inverse coupling linearly in $N_f$, a value of $\alpha _\mathrm {V}^{(3)}(8.3\,\hbox {GeV}) = 0.196(3)$ is obtained. SESAM 99 [623] follows a similar strategy, again for a single lattice spacing. They linearly extrapolated results for $1/\alpha _\mathrm {V}^{(0)}$, $1/\alpha _\mathrm {V}^{(2)}$ at a fixed scale of $9\,\hbox {GeV}$ to give $\alpha _\mathrm {V}^{(3)}$, which is then perturbatively converted to $\alpha _{\overline{\mathrm{MS}}}^{(3)}$. This finally gave $\alpha _{\overline{\mathrm{MS}}}^{(5)}(M_Z) = 0.1118(17)$. Wingate 95 [624] also follow this method. With the scale determined from the charmonium 1S-1P splitting for single lattice spacings in $N_f = 0$, 2 giving $a^{-1}\simeq 1.80\,\hbox {GeV}$ for $N_f=0$ and $a^{-1}\simeq 1.66\,\hbox {GeV}$ for $N_f=2$ they obtain $\alpha _\mathrm {V}^{(0)}(3.41/a)\simeq 0.15$ and $\alpha _\mathrm {V}^{(2)}\simeq 0.18$ respectively. Extrapolating the coupling linearly in $N_f$, they obtain $\alpha _\mathrm {V}^{(3)}(6.48\,\hbox {GeV})=0.194(17)$.

The QCDSF/UKQCD Collaborations, QCDSF/UKQCD 05 [625–628], use the two-loop relation (re-written here in terms of $\alpha$)

249 $$\begin{aligned} {1 \over \alpha _{\overline{\mathrm{MS}}}(\mu )}= & {} {1 \over \alpha _\mathrm {P}(1/a)} + 4\pi (2b_0\ln a\mu - t_1^P) \nonumber \\&+\, (4\pi )^2(2b_1\ln a\mu - t_2^P)\alpha _\mathrm {P}(1/a), \end{aligned}$$

where $t_1^P$ and $t_2^P$ are known. (A two-loop relation corresponds to a 3-loop lattice $\beta$-function.) This was used to directly compute $\alpha _\mathrm{\overline{\mathrm{MS}}}$, and the scale was chosen so that the $\mathcal {O}(\alpha _\mathrm {P}^0)$ term vanishes, i.e.

250 $$\begin{aligned} \mu ^* = {1 \over a} \exp {[t_1^P/(2b_0)] } \approx \left\{ \begin{array}{cc} 2.63/a &{} N_f = 0 \\ 1.4/a &{} N_f = 2 \\ \end{array} \right. . \end{aligned}$$

The method is to first compute $\alpha _\mathrm {P}(1/a)$ and from this using Eq. (249) to find $\alpha _{\overline{\mathrm{MS}}}(\mu ^*)$. The RG equation, Eq. (218), then determines $\mu ^*/\Lambda _{\overline{\mathrm{MS}}}$ and hence using Eq. (250) leads to the result for $r_0\Lambda _{\overline{\mathrm{MS}}}$. This avoids giving the scale in $\hbox {MeV}$ until the end. In the $N_{ f}=0$ case 7 lattice spacings were used [570], giving a range $\mu ^*/\Lambda _{\overline{\mathrm{MS}}} \approx 24$–72 (or $a^{-1} \approx 2$–$7\,\hbox {GeV}$) and $\alpha _\mathrm{eff} = \alpha _{\overline{\mathrm{MS}}}(\mu ^*) \approx 0.15$–0.10. Neglecting higher-order perturbative terms (see discussion after Eq. (251) below) in Eq. (249) this is sufficient to allow a continuum extrapolation of $r_0\Lambda _{\overline{\mathrm{MS}}}$. A similar computation for $N_f = 2$ by QCDSF/UKQCD 05 [625] gave $\mu ^*/\Lambda _{\overline{\mathrm{MS}}} \approx 12$–17 (or roughly $a^{-1} \approx 2$–$3\,\hbox {GeV}$) and $\alpha _\mathrm{eff} = \alpha _{\overline{\mathrm{MS}}}(\mu ^*) \approx 0.20$–0.18. The $N_f=2$ results of QCDSF/UKQCD 05 [625] are affected by an uncertainty which was not known at the time of publication: It has been realized that the values of $r_0/a$ of Ref. [625] were significantly too low [12]. As this effect is expected to depend on *a*, it influences the perturbative behaviour leading us to assign a  for that criterion.

Since FLAG 13, there has been one new result for $N_f = 0$ by FlowQCD 15 [564]. They also use the techniques as described in Eqs. (249), (250), but together with the gradient flow scale $w_0$ (rather than the $r_0$ scale). The continuum limit is estimated by extrapolating the data at 9 lattice spacings linearly in $a^2$. The data range used is $\mu ^*/\Lambda _{\overline{\mathrm{MS}}} \approx 40$–120 (or $a^{-1} \approx 3$–$11\,\hbox {GeV}$) and $\alpha _{\overline{\mathrm{MS}}}(\mu ^*) \approx 0.12$–0.09. Since a very small value of $\alpha _{\overline{\text {MS}}}$ is reached, there is a  in the perturbative behaviour. Note that our conversion to the common $r_0$ scale leads to a significant increase of the error of the $\Lambda$ parameter compared to^69^ $w_{0.4} \Lambda _{\overline{\text {MS}}}=0.2388(5)(13)$.

The work of HPQCD 05A [616] (which supersedes the original work [629]) uses three lattice spacings $a^{-1} \approx 1.2$, 1.6, $2.3\,\hbox {GeV}$ for $2+1$ flavour QCD. Typically the renormalization scale $q \approx \pi /a \approx 3.50$–$7.10\,\hbox {GeV}$, corresponding to $\alpha _\mathrm {V'} \approx 0.22$–0.28.

In the later update HPQCD 08A [617] 12 datasets (with six lattice spacings) are now used reaching up to $a^{-1} \approx 4.4\,\hbox {GeV}$ corresponding to $\alpha _\mathrm {V'}\approx 0.18$. The values used for the scale $r_1$ were further updated in HPQCD 10 [9]. Maltman 08 [63] uses most of the same lattice ensembles as HPQCD 08A [617], but considers a much smaller set of quantities (three versus 22) that are less sensitive to condensates. They also use different strategies for evaluating the condensates and for the perturbative expansion, and a slightly different value for the scale $r_1$. The central values of the final results from Maltman 08 [63] and HPQCD 08A [617] differ by 0.0009 (which would be decreased to 0.0007 taking into account a reduction of 0.0002 in the value of the $r_1$ scale used by Maltman 08 [63]).

As mentioned before, the perturbative coefficients are computed through 3-loop order [630], while the higher-order perturbative coefficients $c_n$ with $n_\mathrm{max} \ge n > 3$ (with $n_\mathrm{max} = 10$) are numerically fitted using the lattice-simulation data for the lattice spacings with the help of Bayesian methods. It turns out that corrections in Eq. (245) are of order $|c_i/c_1|\alpha ^i=$ 5–15% and 3–10% for $i=2,3$, respectively. The inclusion of a fourth-order term is necessary to obtain a good fit to the data, and leads to a shift of the result by 1–2 sigma. For all but one of the 22 quantities, central values of $|c_4/c_1|\approx 2$–4 were found, with errors from the fits of ${\approx } 2$.

An important source of uncertainty is the truncation of perturbation theory. In HPQCD 08A [617], 10 [9] it is estimated to be about 0.4% of $\alpha _{\overline{\text {MS}}}(M_Z)$. In FLAG 13 we included a rather detailed discussion of the issue with the result that we prefer for the time being a more conservative error based on the above estimate $|c_4/c_1| = 2$. From Eq. (244) this gives an estimate of the uncertainty in $\alpha _\mathrm{eff}$ of

251 $$\begin{aligned} \Delta \alpha _\mathrm{eff}(\mu _1) = \left| {c_4 \over c_1}\right| \alpha _\mathrm{eff}^4(\mu _1), \end{aligned}$$

**Table 45:** Wilson loop results

Collaboration	Refs.	$N_f$	Publication status	Renormalization scale	Perturbative behaviour	Lattice spacings	Scale	$\Lambda _{\overline{\text {MS}}}[\,\mathrm {MeV}]$	$r_0\Lambda _{\overline{\text {MS}}}$
HPQCD 10$^{\mathrm{a,j}}$	[9]	$2+1$	A				$r_1 = 0.3133(23)~\hbox {fm}$	340(9)	0.812(22)
HPQCD 08A$^\mathrm{a}$	[617]	$2+1$	A				$r_1 = 0.321(5)~\hbox {fm}^{\mathrm{m}}$	338(12)$^{\mathrm{l}}$	0.809(29)
Maltman 08$^\mathrm{a}$	[63]	$2+1$	A				$r_1 = 0.318~\hbox {fm}$	352(17)$^{\mathrm{k}}$	0.841(40)
HPQCD 05A$^\mathrm{a}$	[616]	$2+1$	A				$r_1^{\,\,\,\mathrm{m}}$	319(17)$^{\mathrm{n}}$	0.763(42)
QCDSF/UKQCD 05	[625]	2	A				$r_0 = 0.467(33)~\hbox {fm}$	261(17)(26)	0.617(40)(21)$^\mathrm{b}$
SESAM 99$^\mathrm{c}$	[623]	2	A				$c\bar{c}$(1S-1P)		
Wingate 95$^\mathrm{d}$	[624]	2	A				$c\bar{c}$(1S-1P)		
Davies 94$^\mathrm{e}$	[622]	2	A				$\Upsilon$		
Aoki 94$^\mathrm{f}$	[621]	2	A				$c\bar{c}$(1S-1P)		
FlowQCD 15	[564]	0	P				$w_{0.4} = 0.193(3)~\hbox {fm}^\mathrm{i}$	$258(6)^\mathrm{i}$	0.618(11)$^\mathrm{i}$
QCDSF/UKQCD 05	[625]	0	A				$r_0 = 0.467(33)~\hbox {fm}$	259(1)(20)	0.614(2)(5)$^\mathrm{b}$
SESAM 99$^\mathrm{c}$	[623]	0	A				$c\bar{c}$(1S-1P)		
Wingate 95$^\mathrm{d}$	[624]	0	A				$c\bar{c}$(1S-1P)		
Davies 94$^\mathrm{e}$	[622]	0	A				$\Upsilon$		
El-Khadra 92$^\mathrm{g}$	[620]	0	A				$c\bar{c}$(1S-1P)	234(10)	0.560(24)$^\mathrm{h}$

$^\mathrm{a}$ The numbers for $\Lambda$ have been converted from the values for $\alpha _s^{(5)}(M_Z)$

$^\mathrm{b}$ This supersedes Refs. [626–628]. $\alpha ^{(5)}_{\overline{\mathrm{MS}}}(M_Z)=0.112(1)(2)$. The $N_f=2$ results were based on values for $r_0 /a$ which have later been found to be too small [12]. The effect will be of the order of 10–15%, presumably an increase in $\Lambda r_0$

$$^\mathrm{c}$$ $$\alpha ^{(5)}_{\overline{\mathrm{MS}}}(M_Z)=0.1118(17)$$

$$^\mathrm{d}$$ $$\alpha _V^{(3)}(6.48~\hbox {GeV})=0.194(7)$$ extrapolated from $$N_{ f}=0,2$$. $$\alpha ^{(5)}_{\overline{\mathrm{MS}}}(M_Z)=0.107(5)$$

$$^\mathrm{e}$$ $$\alpha _P^{(3)}(8.2~\hbox {GeV})=0.1959(34)$$ extrapolated from $$N_f=0,2$$. $$\alpha ^{(5)}_{\overline{\mathrm{MS}}}(M_Z)=0.115(2)$$

$$^\mathrm{f}$$ Estimated $$\alpha ^{(5)}_{\overline{\mathrm{MS}}}(M_Z)=0.108(5)(4)$$

$^\mathrm{g}$ This early computation violates our requirement that scheme conversions are done at the two-loop level. $\Lambda _{\overline{\mathrm{MS}}}^{(4)}=160(^{+47}_{-37})~\hbox {MeV}$, $\alpha ^{(4)}_{\overline{\mathrm{MS}}}(5~\hbox {GeV})=0.174(12)$. We converted this number to give $\alpha ^{(5)}_{\overline{\mathrm{MS}}}(M_Z)=0.106(4)$

$^\mathrm{h}$ We used $r_0=0.472~\hbox {fm}$ to convert to $r_0 \Lambda _{\overline{\text {MS}}}$

$^\mathrm{i}$ Reference scale $w_{0.4}$ where $w_x$ is defined by $\left. t\partial _t[t^2 \langle E(t)\rangle ]\right| _{t=w_x^2}=x$ in terms of the action density *E*(*t*) at positive flow time *t* [564]. Our conversion to $r_0$ scale using [564] $r_0/w_{0.4}=2.587(45)$ and $r_0=0.472~\hbox {fm}$

$^{\mathrm{j}}$ $\alpha _{\overline{\mathrm{MS}}}^{(3)}(5\ \hbox {GeV})=0.2034(21)$, $\alpha ^{(5)}_{\overline{\mathrm{MS}}}(M_Z)=0.1184(6)$, only update of intermediate scale and *c*-, *b*-quark masses, supersedes HPQCD 08A

$$^{\mathrm{k}}$$ $$\alpha ^{(5)}_{\overline{\mathrm{MS}}}(M_Z)=0.1192(11)$$

$$^{\mathrm{l}}$$ $$\alpha _V^{(3)}(7.5~\hbox {GeV})=0.2120(28)$$, $$\alpha ^{(5)}_{\overline{\mathrm{MS}}}(M_Z)=0.1183(8)$$, supersedes HPQCD 05

$^{\mathrm{m}}$ Scale is originally determined from $\Upsilon$ mass splitting. $r_1$ is used as an intermediate scale. In conversion to $r_0\Lambda _{\overline{\mathrm{MS}}}$, $r_0$ is taken to be $0.472~\hbox {fm}$

$$^{\mathrm{n}}$$ $$\alpha _V^{(3)}(7.5~\hbox {GeV})=0.2082(40)$$, $$\alpha ^{(5)}_{\overline{\mathrm{MS}}}(M_Z)=0.1170(12)$$

at the scale $\mu _1$ where $\alpha _\mathrm{eff}$ is computed from the Wilson loops. This can be used with a variation in $\Lambda$ at lowest order of perturbation theory and also applied to $\alpha _s$ evolved to a different scale $\mu _2$,^70^

252 $$\begin{aligned} {\Delta \Lambda \over \Lambda } = {1\over 8\pi b_0 \alpha _s} {\Delta \alpha _s \over \alpha _s},\quad {\Delta \alpha _s(\mu _2) \over \Delta \alpha _s(\mu _1)} = {\alpha _s^2(\mu _2) \over \alpha _s^2(\mu _1)}. \end{aligned}$$

We shall later use this with $\mu _2 = M_Z$ and $\alpha _s(\mu _1)=0.2$ as a typical value extracted from Wilson loops in HPQCD 10 [9], HPQCD 08A [617].

Again we note that the results of QCDSF/UKQCD 05 [625] ($N_f = 0$) and FlowQCD 15 [564] may be affected by frozen topology as they have lattice spacings significantly below $a = 0.05\,\hbox {fm}$. The associated additional systematic error is presently unknown.

Table 45 summarizes the results.

### $\alpha _s$ from current 2-point functions

#### General considerations

The method has been introduced in Ref. [152] and updated in Ref. [9]; see also Ref. [631]. Since FLAG 13 a new application, HPQCD 14A [5], with $2+1+1$ flavours has appeared. There the definition for larger-*n* moments is somewhat simplified and we describe it here. The previously used one can be found in FLAG 13.

The basic observable is constructed from a current

253 $$\begin{aligned} J(x) = i m_{0h}\overline{\psi }_h(x)\gamma _5\psi _{h'}(x) \end{aligned}$$

of two mass-degenerate heavy-valence quarks, *h*, $h^\prime$. The pre-factor $m_{0h}$ denotes the bare mass of the quark. With a residual chiral symmetry, *J*(*x*) is a renormalization group invariant local field, i.e. it requires no renormalization. Staggered fermions and twisted mass fermions have such a residual chiral symmetry. The (Euclidean) time-slice correlation function

254 $$\begin{aligned} G(x_0) = a^3 \sum _{\vec {x}} \langle J^\dagger (x) J(0) \rangle , \end{aligned}$$

($J^\dagger (x) = im_{0h}\overline{\psi }_{h'}(x)\gamma _5\psi _{h}(x)$) has a $\sim x_0^{-3}$ singularity at short distances and moments

255 $$\begin{aligned} G_n = a \sum _{t=-(T/2-a)}^{T/2-a} t^n \,G(t), \end{aligned}$$

are nonvanishing for even *n* and furthermore finite for $n \ge 4$. Here *T* is the time extent of the lattice. The moments are dominated by contributions at *t* of order $1/m_{0h}$. For large mass $m_{0h}$ these are short distances and the moments become increasingly perturbative for decreasing *n*. Denoting the lowest-order perturbation theory moments by $G_n^{(0)}$, one defines the normalized moments

256 $$\begin{aligned} \tilde{R}_n = \left\{ \begin{array}{ll} G_4/G_4^{(0)} &{} \quad \hbox {for } n=4 , \\ { G_n^{1/(n-4)} \over m_{0c}\left( G_n^{(0)} \right) ^{1/(n-4)} } &{}\quad \hbox {for } n \ge 6, \\ \end{array} \right. \end{aligned}$$

of even order *n*. Note that Eq. (253) contains the variable (bare) heavy-quark mass $m_{0h}$, while Eq. (256) is defined with the charm-quark mass, tuned to its physical value. The normalization $m_{0c}( G_n^{(0)})^{1/(n-4)}$ in Eq. (256) ensures that $\tilde{R}_n$ remains renormalization group invariant, but introduces a mass scale. In the continuum limit the normalized moments can then be parameterized in terms of functions257with $\bar{m}_c(\mu )$ being the renormalized charm-quark mass. The reduced moments $r_n$ have a perturbative expansion

258 $$\begin{aligned} r_n = 1 + r_{n,1}\alpha _s + r_{n,2}\alpha _s^2 + r_{n,3}\alpha _s^3 + \ldots \,, \end{aligned}$$

where the written terms $r_{n,i}(\mu /\bar{m}_h(\mu ))$, $i \le 3$ are known for low *n* from Refs. [632–636]. In practice, the expansion is performed in the $\overline{\mathrm{MS}}$ scheme. Matching nonperturbative lattice results for the moments to the perturbative expansion, one determines an approximation to $\alpha _{\overline{\mathrm{MS}}}(\mu )$ as well as $\bar{m}_c(\mu )$. With the lattice spacing (scale) determined from some extra physical input, this calibrates $\mu$. As usual suitable pseudoscalar masses determine the bare quark masses, here in particular the charm mass, and then through Eq. (257) the renormalized charm-quark mass.

**Fig. 31:**
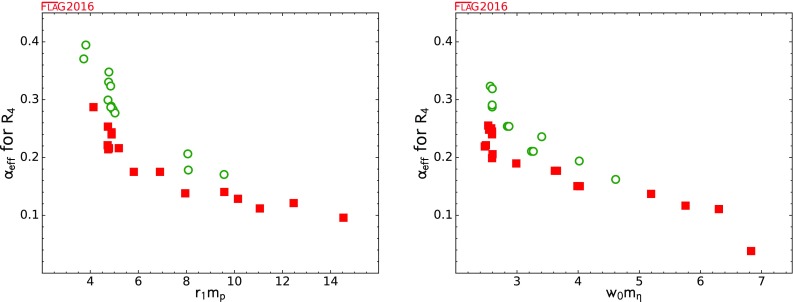
$\alpha _\mathrm{eff}$ for $R_4$ from HPQCD 10 data (*left*) and from HPQCD 14A (*right*). A similar graph for $R_6/R_8$ is shown in FLAG 13. *Symbols* correspond to  for data with $1\le a\mu \le 1.5$ and  for $a\mu >1.5$, while  ($a\mu < 1/2$) is not present. This corresponds exactly to the $a\mu$ part of our continuum limit criterion, but does not consider how many lattice spacings are present. Note that mistunings in the quark masses have not been accounted for, but, estimated as in HPQCD 14A [5], they are smaller than the size of the symbols in the graphs

A difficulty with this approach is that large masses are needed to enter the perturbative domain. Lattice artefacts can then be sizeable and have a complicated form. The ratios in Eq. (256) use the tree-level lattice results in the usual way for normalization. This results in unity as the leading term in Eq. (258), suppressing some of the kinematical lattice artefacts. We note that in contrast to e.g. the definition of $\alpha _\mathrm {qq}$, here the cutoff effects are of order $a^k\alpha _s$, while there the tree-level term defines $\alpha _s$ and therefore the cutoff effects after tree-level improvement are of order $a^k\alpha _s^2$.

Finite-size effects (FSE) due to the omission of $|t| > T /2$ in Eq. (255) grow with *n* as $(m_{\mathrm {p}}T/2)^n\, \exp {(-m_{\mathrm {p}} T/2)}$. In practice, however, since the (lower) moments are short-distance dominated, the FSE are expected to be irrelevant at the present level of precision.

Moments of correlation functions of the quark’s electromagnetic current can also be obtained from experimental data for $e^+e^-$ annihilation [637, 638]. This enables a nonlattice determination of $\alpha _s$ using a similar analysis method. In particular, the same continuum perturbation theory computation enters both the lattice and the phenomenological determinations.

**Table 46:** Current 2-point function results

Collaboration	Refs.	$N_f$	Publication status	Renormalization scale	Perturbative behaviour	Continuum extrapolation	Scale	$\Lambda _{\overline{\text {MS}}}[\,\mathrm {MeV}]$	$r_0\Lambda _{\overline{\text {MS}}}$
HPQCD 14A	[5]	$2+1+1$	A				$w_0=0.1715(9)~\hbox {fm}^\mathrm{a}$	294(11)$^{\mathrm{b},\mathrm{c}}$	0.703(26)
HPQCD 10	[9]	$2+1$	A				$r_1 = 0.3133(23)~\hbox {fm}^{\mathrm{d}}$	338(10)$^{\mathrm{e}}$	0.809(25)
HPQCD 08B	[152]	$2+1$	A				$r_1 = 0.321(5)~\hbox {fm}^{\mathrm{d}}$	325(18)$^{\mathrm{f}}$	0.777(42)

$^\mathrm{a}$ Scale determined in [26] using $f_\pi$

$$^\mathrm{b}$$ $$\alpha ^{(4)}_{\overline{\mathrm{MS}}}(5~\hbox {GeV}) = 0.2128(25)$$, $$\alpha ^{(5)}_{\overline{\mathrm{MS}}}(M_Z) = 0.11822(74)$$

$^\mathrm{c}$ Our conversion for $\Lambda _{\overline{\mathrm{MS}}}$ for $N_f = 4$. We also used $r_0 = 0.472~\hbox {fm}$

$^{\mathrm{d}}$ Scale is determined from $\Upsilon$ mass splitting

$$^{\mathrm{e}}$$ $$\alpha ^{(3)}_{\overline{\mathrm{MS}}}(5\,\hbox {GeV}) = 0.2034(21)$$, $$\alpha ^{(5)}_{\overline{\mathrm{MS}}}(M_Z) = 0.1183(7)$$

$$^{\mathrm{f}}$$ $$\alpha ^{(4)}_{\overline{\mathrm{MS}}}(3\,\hbox {GeV}) = 0.251(6)$$, $$\alpha ^{(5)}_{\overline{\mathrm{MS}}}(M_Z) = 0.1174(12)$$

#### Discussion of computations

The method has originally been applied in HPQCD 08B [152] and in HPQCD 10 [9], based on the MILC ensembles with $2 + 1$ flavours of Asqtad staggered quarks and HISQ valence quarks. The scale was set using $r_1 = 0.321(5)\,\hbox {fm}$ in HPQCD 08B [152] and the updated value $r_1 = 0.3133(23)\,\hbox {fm}$ in HPQCD 10 [9]. The effective range of couplings used is here given for $n = 4$, which is the moment most dominated by short (perturbative) distances and important in the determination of $\alpha _s$. The range is similar for other ratios. With $r_{4,1} = 0.7427$ and $R_4 = 1.28$ determined in the continuum limit at the charm mass in Ref. [152], we have $\alpha _\mathrm{eff} = 0.38$ at the charm-quark mass, which is the mass value where HPQCD 08B [152] carries out the analysis. In HPQCD 10 [9] a set of masses is used, with $R_4 \in [1.09, 1.29]$ which corresponds to $\alpha _\mathrm{eff} \in [0.12, 0.40]$.

The available data of HPQCD 10 [9] is summarized in the left panel of Fig. 31 where we plot $\alpha _{\mathrm {eff}}$ against $m_{\mathrm {p}} r_1$. For the continuum limit criterion, we choose the scale $\mu = 2\bar{m}_h \approx m_{\mathrm {p}}/1.1$, where we have taken $\bar{m}_h$ in the ${\overline{\text {MS}}}$ scheme at scale $\bar{m}_h$ and the numerical value 1.1 was determined in HPQCD 10B [51].

The data in Fig. 31 are grouped according to the range of $a\mu$ that they cover. The vertical spread of the results for $\alpha _\mathrm{eff}$ at fixed $r_1m_{\mathrm {p}}$ in the figure measures the discretization errors seen: in the continuum we would expect all the points to lie on one universal curve. The plots illustrate the selection applied by our criterion for the continuum limit with our choices for $\mu$. Figure 31 gives reason for concern, since it shows that the discretization errors that need to be removed in the continuum extrapolation are not small.

With our choices for $\mu$, the continuum limit criterion is satisfied for three lattice spacings when $\alpha _{\mathrm {eff}} \le 0.3$ and $n=4$. Larger-*n* moments are more influenced by nonperturbative effects. For the *n* values considered, adding a gluon condensate term only changed error bars slightly in HPQCD’s analysis. We note that HPQCD in their papers perform a global fit to all data using a joint expansion in powers of $\alpha _s^n$, $( \Lambda /(m_{\mathrm {p}}/2) )^j$ to parameterize the heavy-quark mass dependence, and $( am_{\mathrm {p}}/2)^{2i}$ to parameterize the lattice-spacing dependence. To obtain a good fit, they must exclude data with $am_{\mathrm {p}} > 1.95$ and include lattice-spacing terms $a^{2i}$ with *i* greater than 10. Because these fits include many more fit parameters than data points, HPQCD uses their expectations for the sizes of coefficients as Bayesian priors. The fits include data with masses as large as $am_{\text {p}}/2 \sim 0.86$, so there is only minimal suppression of the many high-order contributions for the heavier masses. It is not clear, however, how sensitive the final results are to the larger $am_{\text {p}}/2$ values in the data. The continuum limit of the fit is in agreement with a perturbative scale dependence (a 5-loop running $\alpha _{\overline{\mathrm{MS}}}$ with a fitted 5-loop coefficient in the $\beta$-function is used). Indeed, Fig. 2 of Ref. [9] suggests that HPQCD’s fit describes the data well.

The new computation, HPQCD 14A [5], is based on MILC’s $2+1+1$ HISQ staggered ensembles. Compared to HPQCD 10 [9] valence- and sea-quarks now use the same discretization and the scale is set through the gradient flow scale $w_0$, determined to $w_0=0.1715(9)\,\mathrm{fm}$ in Ref. [639].

We again show the values of $\alpha _{\mathrm {eff}}$ as a function of the physical scale. Discretization errors are noticeable. A number of data points, satisfy our continuum limit criterion $a\mu < 1.5$, at two different lattice spacings. This does not by itself lead to a  but the next-larger lattice spacing does not miss the criterion by much; see Table 165. We therefore assign a  in that criterion.

The other details of the analysis by HPQCD 10 [9] are very similar to the ones described above, with one noteworthy exception. The new definition of the moments does not involve the pseudoscalar $h \bar{h}$ mass anymore. Therefore its relation to the quark mass does not need to be modeled in the fit. Since it is now replaced by the renormalized charm-quark mass, the analysis produces a result for $\alpha _s$ and the charm-quark mass at the same time. Here we only discuss the result for $\alpha _s$.

In Table 46 we list the current 2-point function results. Thus far, only one group has used this approach, which models complicated and potentially large cutoff effects together with a perturbative coefficient. We therefore are waiting to see confirmation by other collaborations of the small systematic errors obtained (cf. discussion in Sect. 9.9.2). (We note that more investigations of this method are in progress [174].) We do, however, include the values of $\alpha _{\overline{\mathrm{MS}}}(M_Z)$ and $\Lambda _{\overline{\mathrm{MS}}}$ of HPQCD 10 [9] and HPQCD 14A [5] in our final range.

### $\alpha _s$ from QCD vertices

#### General considerations

The most intuitive and in principle direct way to determine the coupling constant in QCD is to compute the appropriate three- or 4-point gluon vertices or alternatively the quark–quark–gluon vertex or ghost–ghost–gluon vertex (i.e. $q\overline{q}A$ or $c\overline{c}A$ vertex respectively). A suitable combination of renormalization constants then leads to the relation between the bare (lattice) and renormalized coupling constant. This procedure requires the implementation of a nonperturbative renormalization condition and the fixing of the gauge. For the study of nonperturbative gauge fixing and the associated Gribov ambiguity, we refer to Refs. [640–642] and references therein. In practice the Landau gauge is used and the renormalization constants are defined by requiring that the vertex is equal to the tree level value at a certain momentum configuration. The resulting renormalization schemes are called ‘MOM’ scheme (symmetric momentum configuration) or ‘$\widetilde{\mathrm{MOM}}$’ (one momentum vanishes), which are then converted perturbatively to the $\overline{\mathrm{MS}}$ scheme.

A pioneering work to determine the three-gluon vertex in the $N_f = 0$ theory is Alles 96 [643] (which was followed by Ref. [644] for two flavour QCD); a more recent $N_f = 0$ computation was Ref. [645] in which the three-gluon vertex as well as the ghost–ghost–gluon vertex was considered. (This requires in general a computation of the propagator of the Faddeev–Popov ghost on the lattice.) The latter paper concluded that the resulting $\Lambda _{\overline{\mathrm{MS}}}$ depended strongly on the scheme used, the order of perturbation theory used in the matching and also on nonperturbative corrections [646].

Subsequently in Refs. [647, 648] a specific $\widetilde{\mathrm{MOM}}$ scheme with zero ghost momentum for the ghost–ghost–gluon vertex was used. In this scheme, dubbed the ‘MM’ (Minimal MOM) or ‘Taylor’ (T) scheme, the vertex is not renormalized, and so the renormalized coupling reduces to

259 $$\begin{aligned} \alpha _\mathrm{T}(\mu ) = D^\mathrm{gluon}_\mathrm{lat}(\mu , a) D^\mathrm{ghost}_\mathrm{lat}(\mu , a)^2 \, {g_0^2(a) \over 4\pi }, \end{aligned}$$

where $D^\mathrm{ghost}_\mathrm{lat}$ and $D^\mathrm{gluon}_\mathrm{lat}$ are the (bare lattice) dressed ghost and gluon ‘form factors’ of these propagator functions in the Landau gauge,

260 $$\begin{aligned}&D^{ab}(p) = - \delta ^{ab}\, {D^\mathrm{ghost}(p) \over p^2},\nonumber \\&\quad D_{\mu \nu }^{ab}(p) = \delta ^{ab} \left( \delta _{\mu \nu } - {p_\mu p_\nu \over p^2} \right) {D^\mathrm{gluon}(p) \over p^2}, \end{aligned}$$

and we have written the formula in the continuum with $D^\mathrm{ghost/gluon}(p)=D^\mathrm{ghost/gluon}_\mathrm{lat}(p, 0)$. Thus there is now no need to compute the ghost–ghost–gluon vertex, just the ghost and gluon propagators.

#### Discussion of computations

**Table 47:** Results for the gluon–ghost vertex

Collaboration	Refs.	$N_{ f}$	Publication status	Renormalization scale	Perturbative behaviour	Continuum extrapolation	Scale	$\Lambda _{\overline{\text {MS}}}[\,\mathrm {MeV}]$	$r_0\Lambda _{\overline{\text {MS}}}$
ETM 13D	[649]	$2+1+1$	A				$f_\pi$	$314(7)(14)(10)^\mathrm{a}$	$0.752(18)(34)(81)^{\mathrm{b}}$
ETM 12C	[650]	$2+1+1$	A				$f_\pi$	$324(17)^\mathrm{a}$	$0.775(41)^{\mathrm{b}}$
ETM 11D	[651]	$2+1+1$	A				$f_\pi$	$316(13)(8)(^{+0}_{-9})^{\mathrm{c}}$	$0.756(31)(19)(^{+0}_{-22})^{\mathrm{b}}$
Sternbeck 12	[652]	$2+1$	C				Only running of $\alpha _s$ in Fig. 4		
Sternbeck 12	[652]	2	C				Agreement with $r_0\Lambda _{\overline{\text {MS}}}$ value of [12]		
Sternbeck 10	[653]	2	C					$251(15)^{\mathrm{d}}$	0.60(3)(2)
ETM 10F	[654]	2	A				$f_\pi$	$330(23)(22)(^{+0}_{-33})$	$0.72(5)^{\mathrm{e}}$
Boucaud 01B	[644]	2	A				$K^{*}-K$	$264(27)^{\mathrm{f}}$	0.669(69)
Sternbeck 12	[652]	0	C				Agreement with $r_0\Lambda _{\overline{\text {MS}}}$ value of [606]		
Sternbeck 10	[653]	0	C					$259(4)^{\mathrm{d}}$	0.62(1)
Ilgenfritz 10	[655]	0	A				Only running of $\alpha _s$ in Fig. 13		
Boucaud 08	[648]	0	A				$\sqrt{\sigma } = 445~\hbox {MeV}$	$224(3)(^{+8}_{-5})$	$0.59(1)(^{+2}_{-1})$
Boucaud 05	[645]	0	A				$\sqrt{\sigma } = 445~\hbox {MeV}$	320(32)	0.85(9)
Soto 01	[656]	0	A				$\sqrt{\sigma } = 445~\hbox {MeV}$	260(18)	0.69(5)
Boucaud 01A	[657]	0	A				$\sqrt{\sigma } = 445~\hbox {MeV}$	233(28) MeV	0.62(7)
Boucaud 00B	[658]	0	A					Only running of $\alpha _s$	
Boucaud 00A	[659]	0	A				$\sqrt{\sigma } = 445~\hbox {MeV}$	$237(3)(^{+~0}_{-10})$	$0.63(1)(^{+0}_{-3})$
Becirevic 99B	[660]	0	A				$\sqrt{\sigma } = 445~\hbox {MeV}$	$319(14)(^{+10}_{-20})$	$0.84(4)(^{+3}_{-5})$
Becirevic 99A	[661]	0	A				$\sqrt{\sigma } = 445~\hbox {MeV}$	${\lesssim } 353(2)(^{+25}_{-15})$	${\lesssim } 0.93 (^{+7}_{-4})$
Boucaud 98B	[662]	0	A				$\sqrt{\sigma } = 445~\hbox {MeV}$	295(5)(15)	0.78(4)
Boucaud 98A	[663]	0	A				$\sqrt{\sigma } = 445~\hbox {MeV}$	300(5)	0.79(1)
Alles 96	[643]	0	A				$\sqrt{\sigma } = 440~\hbox {MeV}^{\mathrm{g}}$	340(50)	0.91(13)

$$^\mathrm{a}$$ $$\alpha _{\overline{\mathrm{MS}}}^{(5)}(M_Z)=0.1200(14)$$

$^{\mathrm{b}}$ We use the $2+1$ value $r_0=0.472$ fm

$^{\mathrm{c}}$ First error is statistical; second is due to the lattice spacing and third is due to the chiral extrapolation. $\alpha _{\overline{\mathrm{MS}}}^{(5)}(M_Z)=0.1198(9)(5)(^{+0}_{-5})$

$^{\mathrm{d}}$ In the paper only $r_0\Lambda _{\overline{\mathrm{MS}}}$ is given, we converted to $\,\mathrm {MeV}$ with $r_0=0.472$ fm

$^{\mathrm{e}}$ The determination of $r_0$ from the $f_\pi$ scale is found in Ref. [36]

$$^{\mathrm{f}}$$ $$\alpha _{\overline{\mathrm{MS}}}^{(5)}(M_Z)=0.113(3)(4)$$

$^{\mathrm{g}}$ The scale is taken from the string tension computation of Ref. [607]

For the calculations considered here, to match to perturbative scaling, it was first necessary to reduce lattice artefacts by an *H*(4) extrapolation procedure (addressing *O*(4) rotational invariance), e.g. ETM 10F [654] or by lattice perturbation theory, e.g. Sternbeck 12 [652]. To match to perturbation theory, collaborations vary in their approach. In ETM 10F [654] it was necessary to include the operator $A^2$ in the OPE of the ghost and gluon propagators, while in Sternbeck 12 [652] very large momenta are used and $a^2p^2$ and $a^4p^4$ terms are included in their fit to the momentum dependence. A further later refinement was the introduction of higher nonperturbative OPE power corrections in ETM 11D [651] and ETM 12C [650]. Although the expected leading power correction, $1/p^4$, was tried, ETM finds good agreement with their data only when they fit with the next-to-leading-order term, $1/p^6$. The update ETM 13D [649] investigates this point in more detail, using better data with reduced statistical errors. They find that after again including the $1/p^6$ term they can describe their data over a large momentum range from about 1.75 to 7 GeV.

In all calculations except for Sternbeck 10 [653], Sternbeck 12 [652] , the matching with the perturbative formula is performed including power corrections in the form of condensates, in particular $\langle A^2 \rangle$. Three lattice spacings are present in almost all calculations with $N_f=0$, 2, but the scales *ap* are rather large. This mostly results in a  on the continuum extrapolation (Sternbeck 10 [653], Boucaud 01B [644] for $N_f=2$. Ilgenfritz 10 [655], Boucaud 08 [648], Boucaud 05 [645], Becirevic 99B [660], Becirevic 99A [661], Boucaud 98B [662], Boucaud 98A [663], Alles 96 [643] for $N_f=0$). A  is reached in the $N_{ f}=0$ computations Boucaud 00A [659], 00B [658], 01A [657], Soto 01 [656] due to a rather small lattice spacing, but this is done on a lattice of a small physical size. The $N_f=2+1+1$ calculation, fitting with condensates, is carried out for two lattice spacings and with $ap>1.5$, giving  for the continuum extrapolation as well. In ETM 10F [654] we have $0.25< \alpha _\mathrm{eff} < 0.4$, while in ETM 11D [651], ETM 12C [650] (and ETM 13 [33]) we find $0.24< \alpha _\mathrm{eff} < 0.38$ which gives a green circle in these cases for the renormalization scale. In ETM 10F [654] the values of *ap* violate our criterion for a continuum limit only slightly, and we give a .

In Sternbeck 10 [653], the coupling ranges over $0.07 \le \alpha _\mathrm{eff} \le 0.32$ for $N_f=0$ and $0.19 \le \alpha _\mathrm{eff} \le 0.38$ for $N_f=2$ giving  and  for the renormalization scale respectively. The fit with the perturbative formula is carried out without condensates, giving a satisfactory description of the data. In Boucaud 01A [657], depending on *a*, a large range of $\alpha _\mathrm{eff}$ is used which goes down to 0.2 giving a  for the renormalization scale and perturbative behaviour, and several lattice spacings are used leading to  in the continuum extrapolation. The $N_{ f}=2$ computation Boucaud 01B [657], fails the continuum limit criterion because both $a\mu$ is too large and an unimproved Wilson fermion action is used. Finally in the conference proceedings Sternbeck 12 [652], the $N_f=0,2,3$ coupling $\alpha _\mathrm {T}$ is studied. Subtracting one-loop lattice artefacts and subsequently fitting with $a^2p^2$ and $a^4p^4$ additional lattice artefacts, agreement with the perturbative running is found for large momenta ($r_0^2p^2 > 600$) without the need for power corrections. In these comparisons, the values of $r_0\Lambda _{\overline{\text {MS}}}$ from other collaborations are used. As no numbers are given, we have not introduced ratings for this study.

In Table 47 we summarize the results. Presently there are no $N_f \ge 3$ calculations of $\alpha _s$ from QCD vertices that satisfy the FLAG criteria to be included in the range.

### Summary

#### The present situation

**Fig. 32:**
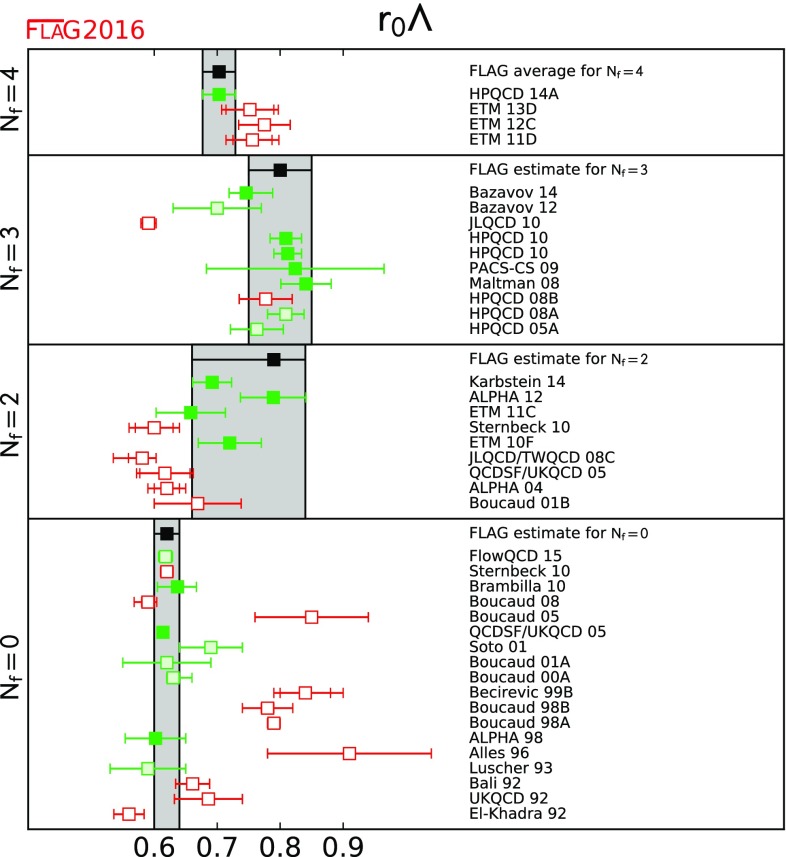
$r_0\Lambda _{\overline{\mathrm{MS}}}$ estimates for $N_f = 0$, 2, 3, 4 flavours. *Full green squares* are used in our final ranges, *pale green squares* also indicate that there are no *red squares* in the colour coding but the computations were superseded by later more complete ones or not published, while *red open squares* mean that there is at least one red square in the colour coding

We first summarize the status of lattice-QCD calculations of the QCD scale $\Lambda _{\overline{\text {MS}}}$. Figure 32 shows all results for $r_0\Lambda _{\overline{\mathrm{MS}}}$ discussed in the previous sections.

Many of the numbers are the ones given directly in the papers. However, when only $\Lambda _{\overline{\mathrm{MS}}}$ in physical units ($\hbox {MeV}$) is available, we have converted them by multiplying with the value of $r_0$ in physical units. The notation used is full green squares for results used in our final average, while a lightly shaded green square indicates that there are no red squares in the previous colour coding but the computation does not enter the ranges because either it has been superseded by an update or it is not published. Red open squares mean that there is at least one red square in the colour coding.

For $N_f=0$ there is relatively little spread in the more recent numbers, even in those which do not satisfy our criteria.

When two flavours of quarks are included, the numbers extracted by the various groups show a considerable spread, as in particular older computations did not yet control the systematics sufficiently. This illustrates the difficulty of the problem and emphasizes the need for strict criteria. The agreement among the more modern calculations with three or more flavours, however, is quite good.

**Fig. 33:**
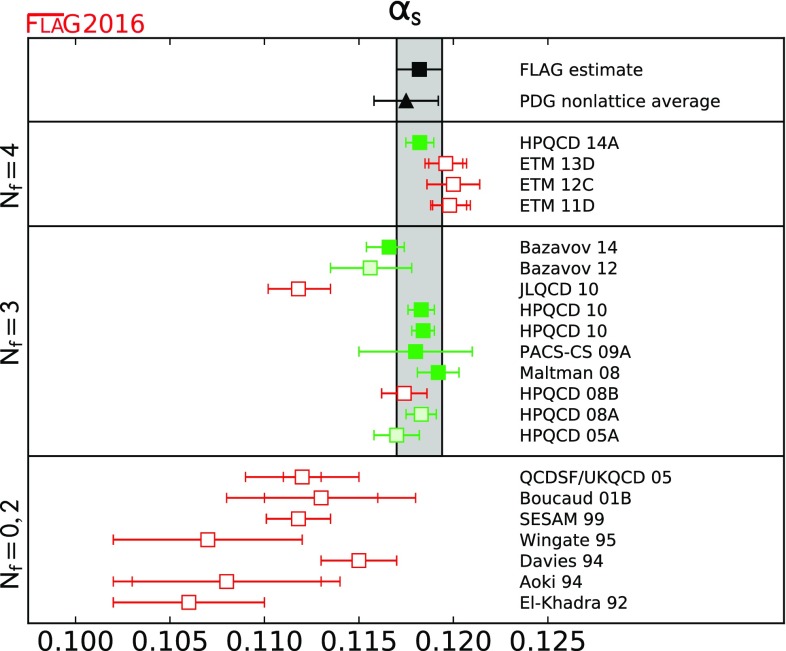
$\alpha _{\overline{\mathrm{MS}}}^{(5)}(M_Z)$, the coupling constant in the $\overline{\mathrm{MS}}$ scheme at the *Z* mass. The results labeled $N_f=0,2$ use estimates for $N_f=3$ obtained by first extrapolating in $N_f$ from $N_f=0,2$ results. Since this is not a theoretically justified procedure, these are not included in our final estimate and are thus given a *red symbol*. However, they are shown to indicate the progress made since these early calculations. The PDG entry indicates the outcome of their analysis excluding lattice results (see Sect. 9.9.4)

We now turn to the status of the essential result for phenomenology, $\alpha _{\overline{\mathrm{MS}}}^{(5)}(M_Z)$. In Table 48 and Fig. 33 we show all the results for $\alpha _{\overline{\mathrm{MS}}}^{(5)}(M_Z)$ (i.e. $\alpha _{\overline{\mathrm{MS}}}$ at the *Z* mass) obtained from $N_f=2+1$ and $N_f = 2+1+1$ simulations. For comparison, we also include results from $N_f = 0$, 2 simulations, which are not relevant for phenomenology. For the $N_f \ge 3$ simulations, the conversion from $N_{ f}= 3$ or $N_{ f}= 4$ to $N_{ f}= 5$ is made by matching the coupling constant at the charm and bottom quark thresholds and using the scale as determined or used by the authors. For $N_f = 0$, 2 the results for $\alpha _{\overline{\text {MS}}}$ in the summary table come from evaluations of $\alpha _{\overline{\text {MS}}}$ at a relatively low scale and are extrapolated in $N_{ f}$ to $N_{ f}= 3$.

**Table 48:** Results for $\alpha _{\overline{\text {MS}}}(M_\mathrm {Z})$. $N_f = 3$ results are matched at the charm and bottom thresholds and scaled to $M_Z$ to obtain the $N_f =5$ result. The arrows in the $N_f$ column indicates which $N_f$ ($N_f = 0$, 2 or a combination of both) were used to first extrapolate to $N_f = 3$ or estimate the $N_f = 3$ value through a model/assumption. The exact procedures used vary and are given in the various papers

Collaboration	Refs.	$N_f$	Publication status	Renormalization scale	Perturbative behaviour	Continuum extrapolation	$\alpha _{\overline{\text {MS}}}(M_\mathrm {Z})$	Method	Tables
HPQCD 14A	[5]	$2+1+1$	A				0.11822(74)	Current two points	46
ETM 13D	[649]	$2+1+1$	A				0.1196(4)(8)(16)	Gluon-ghost vertex	47
ETM 12C	[650]	$2+1+1$	A				0.1200(14)	Gluon-ghost vertex	47
ETM 11D	[651]	$2+1+1$	A				$0.1198(9)(5)(^{+0}_{-5})$	Gluon-ghost vertex	47
Bazavov 14	[61]	$2+1$	A				$0.1166(^{+12}_{-8})$	*Q*-$\bar{Q}$ potential	43
Bazavov 12	[604]	$2+1$	A				$0.1156(^{+21}_{-22})$	*Q*-$\bar{Q}$ potential	43
HPQCD 10	[9]	$2+1$	A				0.1183(7)	Current two points	46
HPQCD 10	[9]	$2+1$	A				0.1184(6)	Wilson loops	45
JLQCD 10	[613]	$2+1$	A				$0.1118(3)(^{+16}_{-17})$	Vacuum polarization	44
PACS-CS 09A	[62]	$2+1$	A				$0.118(3)^{\mathrm{a}}$	Schrödinger functional	42
Maltman 08	[63]	$2+1$	A				0.1192(11)	Wilson loops	45
HPQCD 08B	[152]	$2+1$	A				0.1174(12)	Current two points	46
HPQCD 08A	[617]	$2+1$	A				0.1183(8)	Wilson loops	45
HPQCD 05A	[616]	$2+1$	A				0.1170(12)	Wilson loops	45
QCDSF/UKQCD 05	[625]	$0,2 \rightarrow 3$	A				0.112(1)(2)	Wilson loops	45
Boucaud 01B	[644]	$2\rightarrow 3$	A				0.113(3)(4)	Gluon-ghost vertex	47
SESAM 99	[623]	$0,2\rightarrow 3$	A				0.1118(17)	Wilson loops	45
Wingate 95	[624]	$0,2\rightarrow 3$	A				0.107(5)	Wilson loops	45
Davies 94	[622]	$0,2\rightarrow 3$	A				0.115(2)	Wilson loops	45
Aoki 94	[621]	$2\rightarrow 3$	A				0.108(5)(4)	Wilson loops	45
El-Khadra 92	[620]	$0\rightarrow 3$	A				0.106(4)	Wilson loops	45

$^{\mathrm{a}}$ Result with a linear continuum extrapolation in *a*

As can be seen from the tables and figures, at present there are several computations satisfying the criteria to be included in the FLAG average. Since FLAG 13 two new computations of $\alpha _{\overline{\mathrm{MS}}}^{(5)}(M_Z)$, Bazavov 14 [61] and HPQCD 14A [5], pass all our criteria with a . We note that none of those calculations of $\alpha _{\overline{\mathrm{MS}}}^{(5)}(M_Z)$ satisfy all of our more stringent criteria: a  for the renormalization scale, perturbative behaviour and continuum extrapolation. The results, however, are obtained from four different methods that have different associated systematics, and agree quite well within the stated uncertainties.

#### Our range for $\alpha _{\overline{\mathrm{MS}}}^{(5)}$

We now explain the determination of our range. We only include those results without a red tag and that are published in a refereed journal. We also do not include any numbers which were obtained by extrapolating from theories with less than three flavours. There is no real basis for such extrapolations; rather they use ad hoc assumptions on the low-energy behaviour of the theories. One also notices from the published results that the estimated numbers are quite significantly below those with at least $2+1$ flavours.

A general issue with most recent determinations of $\alpha _{\overline{\text {MS}}}$, both lattice and nonlattice, is that they are dominated by perturbative truncation errors, which are difficult to estimate. Further, all results discussed here except for those of Sects. 9.3 and 9.6 are based on extractions of $\alpha _{\overline{\text {MS}}}$ that are largely influenced by data with $\alpha _{\mathrm {eff}}\ge 0.3$. At smaller $\alpha _s$ the momentum scale $\mu$ quickly is at or above $a^{-1}$. We have included computations using $a\mu$ up to 1.5 and $\alpha _{\mathrm {eff}}$ up to 0.4, but one would ideally like to be significantly below that. Accordingly we choose at this stage to estimate the error ranges in a conservative manner, and not simply perform weighted averages with the individual errors estimated by each group.

Many of the methods have thus far only been applied by a single collaboration, and with simulation parameters that could still be improved. We therefore think that the following aspects of the individual calculations are important to keep in mind, and look forward to additional clarification and/or corroboration in the future.

$\bullet \,$ The potential computations Brambilla 10 [606], ETM 11C [605] and Bazavov 12 [604] give evidence that they have reached distances where perturbation theory can be used. However, in addition to $\Lambda$, a scale is introduced into the perturbative prediction by the process of subtracting the renormalon contribution. This subtraction is avoided in Bazavov 14 [61] by using the force and again agreement with perturbative running is reported. The extractions of $\Lambda$ are dominated by data with $\alpha _{\mathrm {eff}}\ge 0.3$. In contrast, Ref. [608], which studies the force instead of the potential and therefore does not need a renormalon subtraction, finds that significantly smaller lattice spacings would be needed in order for perturbation theory to be reliable in a region of $\mu =1/r$ where discretization errors are controlled. Further study is still needed to clarify the situation.

$\bullet \,$ In the determination of $\alpha _s$ from observables at the lattice-spacing scale, there is an interplay of higher-order perturbative terms and lattice artefacts. In HPQCD 05A [616], HPQCD 08A [617] and Maltman 08 [63] both lattice artefacts (which are power corrections in this approach) and higher-order perturbative terms are fitted. We note that Maltman 08 [63] and HPQCD 08A [617] analyse largely the same dataset but use different versions of the perturbative expansion and treatments of nonperturbative terms. After adjusting for the slightly different lattice scales used, the values of $\alpha _{\overline{\text {MS}}}(M_Z)$ differ by 0.0004 to 0.0008 for the three quantities considered. In fact the largest of these differences (0.0008) comes from a tadpole-improved loop, which is expected to be best behaved perturbatively.

$\bullet \,$ Other computations with very small errors are HPQCD 10 [9] and HPQCD 14A [5], where correlation functions of heavy quarks are used to construct short-distance quantities. Due to the large quark masses needed to reach the region of small coupling, considerable discretization errors are present; see Fig. 31. These are treated by fits to the perturbative running (a 5-loop running $\alpha _{\overline{\mathrm{MS}}}$ with a fitted 5-loop coefficient in the $\beta$-function is used) with high-order terms in a double expansion in $a^2\Lambda ^2$ and $a^2 m_\mathrm {h}^2$ supplemented by priors which limit the size of the coefficients. The priors play an especially important role in these fits given the much larger number of fit parameters than data points. We note, however, that the size of the coefficients does not prevent high-order terms from contributing significantly, since the data includes values of $am_{\text {p}}/2$ that are rather close to 1.

As previously mentioned $\alpha _{\overline{\mathrm{MS}}}^{(5)}(M_Z)$ is summarized in Table 48 and Fig. 33. A number of calculations that include at least the effect of the strange quark make up our final estimate. These are Bazavov 14 [61], HPQCD 14A [5], HPQCD 10 [9] (Wilson loops and current 2-point correlators), PACS-CS 09A [62], Maltman 08 [63] while HPQCD 08A/05A [616, 617] and Bazavov 12 [604] have been superseded by more recent calculations. We obtain the central value for our range,

261 $$\begin{aligned} \alpha _{\overline{\mathrm{MS}}}^{(5)}(M_Z) = 0.1182(12), \end{aligned}$$

from the weighted average of the six results.^71^ Of the results that enter our range, those from Wilson loops (HPQCD 10 [9], and Maltman 08 [63]) and current 2-point correlators (HPQCD 10 [9]) presently have the smallest quoted errors. We have just listed reasons to be careful in estimating the present overall uncertainty. We therefore take a larger range for $\alpha _{\overline{\mathrm{MS}}}^{(5)}(M_Z)$ than one would obtain from the weighted average, or even from the most precise individual calculation. We arrive at its value as follows. We make a conservative estimate of the perturbative uncertainty in the calculation of $\alpha _s$ from small Wilson loops. One approach for making such an estimate would be to take the largest of the differences between the calculations of Maltman 08 [63] and HPQCD 08A [617], 0.0008, which comes from the quantity computed by both groups that is expected to be best behaved perturbatively. This is somewhat larger than some of the estimates in the individual papers. Our choice is instead to take an estimate of the perturbative truncation error as the overall uncertainty. As explained in Sect. 9.6 the first unknown coefficient in the perturbative series was estimated in the fits to be $|c_4/c_1|\approx 2$. Using it in Eqs. (251) and (252)^72^ yields $\Delta \alpha ^{(5)}_{\overline{\mathrm{MS}}}(M_Z) = 0.0012$. This is larger than the estimate of 0.0008 above and is what we adopt as the uncertainty of the Wilson loop results. The second number with small errors entering the average comes from the analysis of moments of heavy-quark correlators. Here an independent estimate of the uncertainty due to the fit to the *a*-dependence (see Fig. 31) is much more difficult to make; as discussed above, and in the absence of confirmation by other groups, we are not yet ready to use the result of HPQCD 10 [9] from the analysis of moments to reduce the size of our range. Thus the overall size of the range is determined by our estimate of the uncertainty of $\alpha _{\overline{\mathrm{MS}}}^{(5)}(M_Z)$ from Wilson loops. It is further reassuring to see that almost all central values that qualify for averaging are within the so-determined range.

The range for $\alpha _{\overline{\mathrm{MS}}}^{(5)}(M_Z)$ presented here is based on results with rather different systematics (apart from the matching across the charm threshold). We therefore believe that the true value is quite likely to lie within this range.

We emphasize once more that all computations which enter this range rely on a perturbative inclusion of the charm and beauty quarks. While perturbation theory for the matching of $\bar{g}^2_{N_f}$ and $\bar{g}^2_{N_f-1}$ looks very well behaved even at the mass of the charm, this scale is rather low and we have no accurate information about the precision of perturbation theory. Nonperturbative studies are not yet precise enough [90]. However, it seems unlikely that the associated uncertainty is comparable with the present errors. With future improved precision, this will become a relevant issue. Note that this uncertainty is also present in some of the phenomenological determinations, in particular from $\tau$ decays.

#### Ranges for $[r_0 \Lambda ]^{(N_{ f})}$ and $\Lambda _{\overline{\text {MS}}}$

In the present situation, we give ranges for $[r_0 \Lambda ]^{(N_{ f})}$ and $\Lambda _{\overline{\text {MS}}}$, discussing their determination case by case. We include results with $N_{ f}<3$ because it is interesting to see the $N_{ f}$-dependence of the connection of low- and high-energy QCD. This aids our understanding of the field theory and helps in finding possible ways to tackle it beyond the lattice approach. It is also of interest in providing an impression on the size of the vacuum polarization effects of quarks, in particular with an eye on the still difficult-to-treat heavier charm and beauty quarks. Even if this information is rather qualitative, it may be valuable, given that it is of a completely nonperturbative nature. We emphasize that results for $[r_0 \Lambda ]^{(0)}$ and $[r_0 \Lambda ]^{(2)}$ are *not* meant to be used in phenomenology.

For $N_{ f}=2+1+1$, we presently do not quote a range as there is a single result: HPQCD 14A [5] found $[r_0 \Lambda ]^{(4)} = 0.70(3)$.

For $N_{ f}=2+1$, we take as a central value the weighted average of Bazavov 14 [61], HPQCD 10 [9] (Wilson loops and current 2-point correlators), PACS-CS 09A [62] and Maltman 08 [63]. Since the uncertainty in $r_0$ is small compared to that of $\Lambda$, we can directly propagate the error from Eq. (261) and arrive at

262 $$\begin{aligned}{}[r_0 \Lambda _{\overline{\text {MS}}}]^{(3)} = 0.80(5). \end{aligned}$$

It is in good agreement with all $2+1$ results without red tags. In physical units, using $r_0=0.472$ fm and neglecting its error, this means

263 $$\begin{aligned} \Lambda _{\overline{\text {MS}}}^{(3)} = 336(19)\,\hbox {MeV}. \end{aligned}$$

For $N_f=2$, at present there is one computation with a  rating for all criteria, ALPHA 12 [12]. We adopt it as our central value and enlarge the error to cover the central values of the other three results with filled green boxes. This results in an asymmetric error. Our range is unchanged as compared to FLAG 13,

264 $$\begin{aligned}{}[r_0 \Lambda _{\overline{\text {MS}}}]^{(2)} = 0.79(^{+~5}_{-{13}}), \end{aligned}$$

and in physical units, using $r_0=0.472$ fm,

265 $$\begin{aligned} \Lambda _{\overline{\text {MS}}}^{(2)} = 330(^{+21}_{-{54}})~\hbox {MeV}. \end{aligned}$$

A weighted average of the four eligible numbers would yield $[r_0 \Lambda _{\overline{\text {MS}}}]^{(2)} = 0.709(22)$, not covering the best result and in particular leading to a smaller error than we feel is justified, given the issues discussed previously in Sect. 9.4.2 (Karbstein 14 [563], ETM 11C [605]) and Sect. 9.8.2 (ETM 10F [654]). Thus we believe that our estimate is a conservative choice; the low value of ETM 11C [605] leads to a large downward error. We hope that future work will improve the situation.

For $N_f=0$ we take into account ALPHA 98 [590], QCDSF/UKQCD 05 [625], and Brambilla 10 [606] for forming a range. We exclude the older estimates shown in the graph which have a limited control of the systematic errors due to power law corrections and discretization errors.^73^ None of the computations have a full set of  and has P for publication status. Taking a weighted average of the three numbers, we obtain $[r_0 \Lambda _{\overline{\text {MS}}}]^{(0)} = 0.615(5)$, dominated by the QCDSF/UKQCD 05 [625] result.

Since we are not yet convinced that such a small uncertainty has been reached, we prefer to presently take a range which encompasses all four central values and whose uncertainty comes close to our estimate of the perturbative error in QCDSF/UKQCD 05 [625]: based on $|c_4/c_1| \approx 2$ as before, we find $\Delta [r_0 \Lambda _{\overline{\text {MS}}}]^{(0)} = 0.018$. We then have

266 $$\begin{aligned}{}[r_0 \Lambda _{\overline{\text {MS}}}]^{(0)} = 0.62(2). \end{aligned}$$

Converting to physical units, again using $r_0=0.472\,\hbox {fm}$ yields

267 $$\begin{aligned} \Lambda _{\overline{\text {MS}}}^{(0)} = 260(7)~\hbox {MeV}. \end{aligned}$$

While the conversion of the $\Lambda$ parameter to physical units is quite unambiguous for $N_{ f}=2+1$, our choice of $r_0=0.472$ fm also for smaller numbers of flavour amounts to a convention, in particular for $N_{ f}=0$. Indeed, in the Tables 42, 43, 44, 45, 46 and 47 somewhat different numbers in MeV are found.

How sure are we about our ranges for $[r_0 \Lambda _{\overline{\text {MS}}}]^{(N_f)}$? In one case we have a result, Eq. (264) which easily passes our criteria, in another one (Eq. (266)) we have three compatible results which are close to that quality and agree. For $N_{ f}=2+1$ the range (Eq. (262)) takes account of results with rather different systematics. We therefore find it difficult to imagine that the ranges could be violated by much.

#### Conclusions

With the present results our range for the strong coupling is (repeating Eq. (261))

$$\begin{aligned} \alpha _{\overline{\mathrm{MS}}}^{(5)}(M_Z) = 0.1182(12)\quad \,\mathrm {Refs.}~ [5, 9, 61{-}63], \end{aligned}$$

and the associated $\Lambda$ parameter

268 $$\begin{aligned} \Lambda _{\overline{\mathrm{MS}}}^{(5)} = 211(14)\,\,\mathrm {MeV}\quad \,\mathrm {Refs.}~ [5, 9, 61{-}63]. \end{aligned}$$

These have changed little compared to the previous FLAG review. As can be seen from Fig. 33, when surveying the green data points, the individual lattice results agree within their quoted errors. Furthermore those points are based on different methods for determining $\alpha _s$, each with its own difficulties and limitations. Thus the overall consistency of the lattice $\alpha _s$ results engenders confidence in our range.

It is interesting to compare to the new Particle Data Group world average, which appeared in February 2016 [151]. The PDG performs their averages, both of lattice determinations and of different categories of phenomenological determinations of $\alpha _s$, in a way differing significantly from how we determine our range. They perform an unweighted average of the mean values. As its error they use the average of the quoted errors of the different determinations that went into the average. This procedure leads to larger final uncertainties than the one used in the previous edition [559]. When one applies this method to the numbers entering Eq. (261), i.e. the ones satisfying our criteria, one obtains $\alpha _{\overline{\mathrm{MS}}}^{(5)}(M_Z) = 0.1181(12)\,.$ This number is close to our result Eq. (261). It differs a little from the value quoted by the PDG since in a couple of cases we used updated results and because not all determinations entering the PDG average satisfy our criteria. For comparison, the PDG number for lattice results is 0.1187(12), and their average of all phenomenological results is 0.1175(17).

Our range for the lattice determination of $\alpha _{\overline{\mathrm{MS}}}(M_Z)$ in Eq. (261) is in excellent agreement with the PDG nonlattice average Eq. (217). This is an excellent check for the subtle interplay of theory, phenomenology and experiments in the nonlattice determinations. The work done on the lattice provides an entirely independent determination, with negligible experimental uncertainty, which reaches a better precision even with our conservative estimate of its uncertainty.

We finish by commenting on perspectives for the future. In the next few years we anticipate that a growing number of lattice calculations of $\alpha _s$ from different quantities and by different collaborations will enable increasingly precise determinations, coupled with stringent cross-checks. The determination of $\alpha _s$ from observables at the lattice-spacing scale may improve due to a further reduction of the lattice spacing. This reduces $\alpha _{\mathrm {eff}}$ and thus the dominating error in $\alpha _{\overline{\text {MS}}}$ as long as perturbative results for the simulated action are available to high order. Schrödinger functional methods for $N_f=2+1$ will certainly reach the precision of the present $N_f=2$ results soon, as this just requires an application of the presently known techniques. Furthermore, we may expect a significant reduction of errors due to new definitions of running couplings [591, 592] using the Yang Mills gradient flow [245]. Factors of two and more in precision are certainly possible. At this point it will then also be necessary to include the charm quark in the computations such that the perturbative matching of $N_f=2+1$ and $2+1+1$ theories at the charm-quark threshold is avoided. First generation $N_f=2+1+1$ simulations are presently being carried out.

## Appendix A: Glossary

### A.1 Lattice actions

In this appendix we give brief descriptions of the lattice actions used in the simulations and summarize their main features.

#### A.1.1 Gauge actions

The simplest and most widely used discretization of the Yang–Mills part of the QCD action is the Wilson plaquette action [664]:

269 $$\begin{aligned} S_\mathrm{G} = \beta \sum _{x} \sum _{\mu <\nu }\left( 1-\frac{1}{3}\mathrm{Re\,\mathrm{Tr}\,}\,W_{\mu \nu }^{1\times 1}(x)\right) , \end{aligned}$$

where $\beta \equiv 6/g_0^2$ (with $g_0$ the bare gauge coupling) and the plaquette $W_{\mu \nu }^{1\times 1}(x)$ is the product of link variables around an elementary square of the lattice, i.e.

270 $$\begin{aligned} W_{\mu \nu }^{1\times 1}(x) \equiv U_\mu (x)U_\nu (x+a\hat{\mu }) U_\mu (x+a\hat{\nu })^{-1} U_\nu (x)^{-1}.\nonumber \\ \end{aligned}$$

This expression reproduces the Euclidean Yang–Mills action in the continuum up to corrections of order $a^2$. There is a general formalism, known as the “Symanzik improvement programme” [64, 65], which is designed to cancel the leading lattice artefacts, such that observables have an accelerated rate of convergence to the continuum limit. The improvement programme is implemented by adding higher-dimensional operators, whose coefficients must be tuned appropriately in order to cancel the leading lattice artefacts. The effectiveness of this procedure depends largely on the method with which the coefficients are determined. The most widely applied methods (in ascending order of effectiveness) include perturbation theory, tadpole-improved (partially resummed) perturbation theory, renormalization group methods, and the nonperturbative evaluation of improvement conditions.

In the case of Yang–Mills theory, the simplest version of an improved lattice action is obtained by adding rectangular $1\times 2$ loops to the plaquette action, i.e.

271 $$\begin{aligned} S_\mathrm{G}^\mathrm{imp}= & {} \beta \sum _{x}\left\{ c_0\sum _{\mu <\nu }\left( 1-\frac{1}{3}\mathrm{Re\,\mathrm{Tr}\,}\,W_{\mu \nu }^{1\times 1}(x)\right) \right. \nonumber \\&\left. +\, c_1\sum _{\mu ,\nu } \left( 1-\frac{1}{3}\mathrm{Re\,\mathrm{Tr}\,}\,W_{\mu \nu }^{1\times 2}(x)\right) \right\} , \end{aligned}$$

where the coefficients $c_0, c_1$ satisfy the normalization condition $c_0+8c_1=1$. The *Symanzik-improved* [665], *Iwasaki* [666], and *DBW2* [667, 668] actions are all defined through Eq. (271) via particular choices for $c_0, c_1$. Details are listed in Table 49 together with the abbreviations used in the summary tables. Another widely used variant is the *tadpole Symanzik-improved* [618, 669] action which is obtained by adding additional 6-link parallelogram loops $W_{\mu \nu \sigma }^{1\times 1\times 1}(x)$ to the action in Eq. (271), i.e.

272 $$\begin{aligned} S_\mathrm{G}^\mathrm{tadSym} = S_\mathrm{G}^\mathrm{imp} + \beta \sum _x c_2 \sum _{\mu<\nu <\sigma }\left( 1-\frac{1}{3} \mathrm{Re\,\mathrm{Tr}\,}\,W_{\mu \nu \sigma }^{1\times 1\times 1}(x)\right) ,\!\!\!\nonumber \\ \end{aligned}$$

where

273 $$\begin{aligned} W_{\mu \nu \sigma }^{1\times 1\times 1}(x)\equiv & {} U_\mu (x)U_\nu (x+a\hat{\mu })U_\sigma (x+a\hat{\mu }+a\hat{\nu })\nonumber \\&\times \, U_\mu (x+a\hat{\sigma }+a\hat{\nu })^{-1} U_\nu (x+a\hat{\sigma })^{-1} U_\sigma (x)^{-1}\nonumber \\ \end{aligned}$$

allows for one-loop improvement [665].

**Table 49:** Summary of lattice gauge actions. The leading lattice artefacts are $\mathcal {O}(a^2)$ or better for all discretizations

Abbrev.	$c_1$	Description
Wilson	0	Wilson plaquette action
tlSym	$-1/12$	Tree-level Symanzik-improved gauge action
tadSym	Variable	Tadpole Symanzik-improved gauge action
Iwasaki	$-0.331$	Renormalization group improved (“Iwasaki”) action
DBW2	$-1.4088$	Renormalization group improved (“DBW2”) action

#### A.1.2 Light-quark actions

If one attempts to discretize the quark action, one is faced with the fermion doubling problem: the naive lattice transcription produces a 16-fold degeneracy of the fermion spectrum.

*Wilson fermions:*

Wilson’s solution to the fermion doubling problem is based on adding a dimension-5 (irrelevant) operator to the lattice action. The Wilson–Dirac operator for the massless case reads [664, 670]

274 $$\begin{aligned} D_\mathrm{w} = \textstyle {1\over 2}\gamma _\mu (\nabla _\mu +\nabla _\mu ^*) +a\nabla _\mu ^*\nabla _\mu , \end{aligned}$$

where $\nabla _\mu ,\,\nabla _\mu ^*$ denote the covariant forward and backward lattice derivatives, respectively. The addition of the Wilson term $a\nabla _\mu ^*\nabla _\mu$, results in fermion doublers acquiring a mass proportional to the inverse lattice spacing; close to the continuum limit these extra degrees of freedom are removed from the low-energy spectrum. However, the Wilson term also results in an explicit breaking of chiral symmetry even at zero bare quark mass. Consequently, it also generates divergences proportional to the UV cutoff (inverse lattice spacing), besides the usual logarithmic ones. Therefore the chiral limit of the regularized theory is not defined simply by the vanishing of the bare quark mass but must be appropriately tuned. As a consequence quark-mass renormalization requires a power subtraction on top of the standard multiplicative logarithmic renormalization. The breaking of chiral symmetry also implies that the nonrenormalization theorem has to be applied with care [671, 672], resulting in a normalization factor for the axial current which is a regular function of the bare coupling. On the other hand, vector symmetry is unaffected by the Wilson term and thus a lattice (point split) vector current is conserved and obeys the usual nonrenormalization theorem with a trivial (unity) normalization factor. Thus, compared to lattice fermion actions which preserve chiral symmetry, or a subgroup of it, the Wilson regularization typically results in more complicated renormalization patterns.

Furthermore, the leading-order lattice artefacts are of order *a*. With the help of the Symanzik improvement programme, the leading artefacts can be cancelled in the action by adding the so-called “Clover” or Sheikholeslami–Wohlert (SW) term [673]. The resulting expression in the massless case reads

275 $$\begin{aligned} D_\mathrm{sw} = D_\mathrm{w} +\frac{ia}{4}\,c_\mathrm{sw}\sigma _{\mu \nu }\widehat{F}_{\mu \nu }, \end{aligned}$$

where $\sigma _{\mu \nu }=\frac{i}{2}[\gamma _\mu ,\gamma _\nu ]$, and $\widehat{F}_{\mu \nu }$ is a lattice transcription of the gluon field strength tensor $F_{\mu \nu }$. The coefficient $c_\mathrm{sw}$ can be determined perturbatively at tree-level ($c_\mathrm{sw}= 1$; tree-level improvement or tlSW for short), via a mean-field approach [618] (mean-field improvement or mfSW) or via a nonperturbative approach [674] (nonperturbatively improved or npSW). Hadron masses, computed using $D_\mathrm{sw}$, with the coefficient $c_\mathrm{sw}$ determined nonperturbatively, will approach the continuum limit with a rate proportional to $a^2$; with tlSW for $c_\mathrm{sw}$ the rate is proportional to $g_0^2 a$.

Other observables require additional improvement coefficients [673]. A common example consists in the computation of the matrix element $\langle \alpha \vert Q \vert \beta \rangle$ of a composite field *Q* of dimension-*d* with external states $\vert \alpha \rangle$ and $\vert \beta \rangle$. In the simplest cases, the above bare matrix element diverges logarithmically and a single renormalization parameter $Z_Q$ is adequate to render it finite. It then approaches the continuum limit with a rate proportional to the lattice spacing *a*, even when the lattice action contains the Clover term. In order to reduce discretization errors to ${\mathcal {O}}(a^2)$, the lattice definition of the composite operator *Q* must be modified (or “improved”), by the addition of all dimension-$(d+1)$ operators with the same lattice symmetries as *Q*. Each of these terms is accompanied by a coefficient which must be tuned in a way analogous to that of $c_\mathrm{sw}$. Once these coefficients are determined nonperturbatively, the renormalized matrix element of the improved operator, computed with a npSW action, converges to the continuum limit with a rate proportional to $a^2$. A tlSW improvement of these coefficients and $c_\mathrm{sw}$ will result in a rate proportional to $g_0^2 a$.

It is important to stress that the improvement procedure does not affect the chiral properties of Wilson fermions; chiral symmetry remains broken.

Finally, we mention “twisted-mass QCD” as a method which was originally designed to address another problem of Wilson’s discretization: the Wilson–Dirac operator is not protected against the occurrence of unphysical zero modes, which manifest themselves as “exceptional” configurations. They occur with a certain frequency in numerical simulations with Wilson quarks and can lead to strong statistical fluctuations. The problem can be cured by introducing a so-called “chirally twisted” mass term. The most common formulation applies to a flavour doublet $\bar{\psi }= ( u \quad d)$ of mass-degenerate quarks, with the fermionic part of the QCD action in the continuum assuming the form [396]

276 $$\begin{aligned} S_\mathrm{F}^\mathrm{tm;cont} = \int \mathrm{d}^4{x}\, \bar{\psi }(x)(\gamma _\mu D_\mu + m + i\mu _\mathrm{q}\gamma _5\tau ^3)\psi (x). \end{aligned}$$

Here, $\mu _\mathrm{q}$ is the twisted-mass parameter, and $\tau ^3$ is a Pauli matrix in flavour space. The standard action in the continuum can be recovered via a global chiral field rotation. The physical quark mass is obtained as a function of the two mass parameters *m* and $\mu _\mathrm{q}$. The corresponding lattice regularization of twisted-mass QCD (tmWil) for $N_{ f}=2$ flavours is defined through the fermion matrix

277 $$\begin{aligned} D_\mathrm{w}+m_0+i\mu _\mathrm{q}\gamma _5\tau ^3. \end{aligned}$$

Although this formulation breaks physical parity and flavour symmetries, resulting in nondegenerate neutral and charged pions, is has a number of advantages over standard Wilson fermions. First of all, the presence of the twisted-mass parameter $\mu _\mathrm{q}$ protects the discretized theory against unphysical zero modes. A second attractive feature of twisted-mass lattice QCD is the fact that, once the bare mass parameter $m_0$ is tuned to its “critical value” (corresponding to massless pions in the standard Wilson formulation), the leading lattice artefacts are of order $a^2$ without the need to add the Sheikholeslami–Wohlert term in the action, or other improving coefficients [675]. A third important advantage is that, although the problem of explicit chiral symmetry breaking remains, quantities computed with twisted fermions with a suitable tuning of the mass parameter $\mu _\mathrm{q}$, are subject to renormalization patterns which are simpler than the ones with standard Wilson fermions. Well known examples are the pseudoscalar decay constant and $B_\mathrm{K}$.

*Staggered fermions:*

An alternative procedure to deal with the doubling problem is based on so-called “staggered” or Kogut–Susskind fermions [676–679]. Here the degeneracy is only lifted partially, from 16 down to 4. It has become customary to refer to these residual doublers as “tastes” in order to distinguish them from physical flavours. Taste changing interactions can occur via the exchange of gluons with one or more components of momentum near the cutoff $\pi /a$. This leads to the breaking of the *SU*(4) vector symmetry among tastes, thereby generating order $a^2$ lattice artefacts.

The residual doubling of staggered quarks (four tastes per flavour) is removed by taking a fractional power of the fermion determinant [680] – the “fourth-root procedure,” or, sometimes, the “fourth-root trick.” This procedure would be unproblematic if the action had full *SU*(4) taste symmetry, which would give a Dirac operator that was block-diagonal in taste space. However, the breaking of taste symmetry at nonzero lattice spacing leads to a variety of problems. In fact, the fourth root of the determinant is not equivalent to the determinant of any local lattice Dirac operator [681]. This in turn leads to violations of unitarity on the lattice [682–685].

According to standard renormalization group lore, the taste violations, which are associated with lattice operators of dimension greater than four, might be expected to go away in the continuum limit, resulting in the restoration of locality and unitarity. However, there is a problem with applying the standard lore to this nonstandard situation: the usual renormalization group reasoning assumes that the lattice action is local. Nevertheless, Shamir [686, 687] shows that one may apply the renormalization group to a “nearby” local theory, and thereby gives a strong argument that the desired local, unitary theory of QCD is reproduced by the rooted staggered lattice theory in the continuum limit.

A version of chiral perturbation that includes the lattice artefacts due to taste violations and rooting (“rooted staggered chiral perturbation theory”) can also be worked out [329, 688, 689] and shown to correctly describe the unitarity-violating lattice artefacts in the pion sector [683, 690]. This provides additional evidence that the desired continuum limit can be obtained. Further, it gives a practical method for removing the lattice artefacts from simulation results. Versions of rooted staggered chiral perturbation theory exist for heavy–light mesons with staggered light quarks but nonstaggered heavy quarks [691], heavy–light mesons with staggered light and heavy quarks [692, 693], staggered baryons [694], and mixed actions with a staggered sea [276, 278], as well as the pion-only version referenced above.

There is also considerable numerical evidence that the rooting procedure works as desired. This includes investigations in the Schwinger model [695–697], studies of the eigenvalues of the Dirac operator in QCD [698–701], and evidence for taste restoration in the pion spectrum as $a\rightarrow 0$ [89, 107].

Issues with the rooting procedure have led Creutz [702–708] to argue that the continuum limit of the rooted staggered theory cannot be QCD. These objections have, however, been answered in Refs. [86–88, 701, 709–712]. In particular, a claim that the continuum ’t Hooft vertex [713, 714] could not be properly reproduced by the rooted theory has been refuted [701, 710].

Overall, despite the lack of rigorous proof of the correctness of the rooting procedure, we think the evidence is strong enough to consider staggered QCD simulations on a par with simulations using other actions. See the following reviews for further evidence and discussion: Refs. [85–89].

*Improved staggered fermions:*

An improvement program can be used to suppress taste-changing interactions, leading to “improved staggered fermions,” with the so-called “Asqtad” [715], “HISQ” [716], “Stout-smeared” [717], and “HYP” [478] actions as the most common versions. All these actions smear the gauge links in order to reduce the coupling of high-momentum gluons to the quarks, with the main goal of decreasing taste-violating interactions. In the Asqtad case, this is accomplished by replacing the gluon links in the derivatives by averages over 1-, 3-, 5-, and 7-link paths. The other actions reduce taste changing even further by smearing more. In addition to the smearing, the Asqtad and HISQ actions include a three-hop term in the action (the “Naik term” [718]) to remove order $a^2$ errors in the dispersion relation, as well as a “Lepage term” [719] to cancel other order $a^2$ artefacts introduced by the smearing. In both the Asqtad and HISQ actions, the leading taste violations are of order $\alpha _S^2 a^2$, and “generic” lattices artefacts (those associated with discretization errors other than taste violations) are of order $\alpha _S a^2$. The overall coefficients of these errors are, however, significantly smaller with HISQ than with Asqtad. With the stout-smeared and HYP actions, the errors are formally larger (order $\alpha _S a^2$ for taste violations and order $a^2$ for generic lattices artefacts). Nevertheless, the smearing seems to be very efficient, and the actual size of errors at accessible lattice spacings appears to be at least as small as with HISQ.

Although logically distinct from the light-quark improvement program for these actions, it is customary with the HISQ action to include an additional correction designed to reduce discretization errors for heavy quarks (in practice, usually charm quarks) [716]. The Naik term is adjusted to remove leading $(am_c)^4$ and $\alpha _S(am_c)^2$ errors, where $m_c$ is the charm-quark mass and “leading” in this context means leading in powers of the heavy-quark velocity *v* ($v/c\sim 1/3$ for $D_s$). With these improvements, the claim is that one can use the staggered action for charm quarks, although it must be emphasized that it is not obvious a priori how large a value of $am_c$ may be tolerated for a given desired accuracy, and this must be studied in the simulations.

*Ginsparg–Wilson fermions:*

Fermionic lattice actions, which do not suffer from the doubling problem whilst preserving chiral symmetry go under the name of “Ginsparg–Wilson fermions”. In the continuum the massless Dirac operator (*D*) anticommutes with $\gamma _5$. At nonzero lattice spacing a chiral symmetry can be realized if this condition is relaxed to [720–722]

278 $$\begin{aligned} \left\{ D,\gamma _5\right\} = aD\gamma _5 D, \end{aligned}$$

which is now known as the Ginsparg–Wilson relation [398]. The Nielsen–Ninomiya theorem [723], which states that any lattice formulation for which *D* anticommutes with $\gamma _5$ necessarily has doubler fermions, is circumvented since $\{D,\gamma _5\}\ne 0$.

A lattice Dirac operator which satisfies Eq. (278) can be constructed in several ways. The so-called “overlap” or Neuberger–Dirac operator [724] acts in four space-time dimensions and is, in its simplest form, defined by

279 $$\begin{aligned}&D_\mathrm{N} = \frac{1}{\overline{a}} \left( 1-\epsilon (A) \right) ,\quad \hbox {where }\epsilon (A)\equiv A (A^\dagger A)^{-1/2},\nonumber \\&\quad A=1+s-aD_\mathrm{w},\quad \overline{a}=\frac{a}{1+s}, \end{aligned}$$

$D_\mathrm{w}$ is the massless Wilson–Dirac operator and $|s|<1$ is a tunable parameter. The overlap operator $D_\mathrm{N}$ removes all doublers from the spectrum, and can readily be shown to satisfy the Ginsparg–Wilson relation. The occurrence of the sign function $\epsilon (A)$ in $D_\mathrm{N}$ renders the application of $D_\mathrm{N}$ in a computer program potentially very costly, since it must be implemented using, for instance, a polynomial approximation.

The most widely used approach to satisfying the Ginsparg–Wilson relation Eq. (278) in large-scale numerical simulations is provided by *Domain Wall Fermions* (DWF) [725–727] and we therefore describe this in some more detail. Following early exploratory studies [728]. this approach has been developed into a practical formulation of lattice QCD with good chiral and flavour symmetries leading to results which contribute significantly to this review. In this formulation, the fermion fields $\psi (x,s)$ depend on a discrete fifth coordinate $s=1,\ldots ,N$ as well as the physical four-dimensional space-time coordinates $x_\mu ,\,\mu =1\cdots 4$ (the gluon fields do not depend on *s*). The lattice on which the simulations are performed, is therefore a five-dimensional one of size $L^3\times T\times N$, where $L,\,T$ and *N* represent the number of points in the spatial, temporal and fifth dimensions respectively. The remarkable feature of DWF is that for each flavour there exists a physical light mode corresponding to the field *q*(*x*):

280 $$\begin{aligned} q(x)= & {} \frac{1+\gamma ^5}{2}\psi (x,1)+\frac{1-\gamma ^5}{2}\psi (x,N) \end{aligned}$$

281 $$\begin{aligned} \bar{q}(x)= & {} \overline{\psi }(x,N)\frac{1+\gamma ^5}{2} + \overline{\psi }(x,1)\frac{1-\gamma ^5}{2}. \end{aligned}$$

The left and right-handed modes of the physical field are located on opposite boundaries in the five-dimensional space which, for $N\rightarrow \infty$, allows for independent transformations of the left and right components of the quark fields, that is, for chiral transformations. Unlike Wilson fermions, where for each flavour the quark-mass parameter in the action is fine-tuned requiring a subtraction of contributions of $\mathcal {O}(1/a)$ where *a* is the lattice spacing, with DWF no such subtraction is necessary for the physical modes, whereas the unphysical modes have masses of $\mathcal {O}(1/a)$ and decouple.

In actual simulations *N* is finite and there are small violations of chiral symmetry which must be accounted for. The theoretical framework for the study of the residual breaking of chiral symmetry has been a subject of intensive investigation (for a review and references to the original literature see e.g. [729]). The breaking requires one or more *crossings* of the fifth dimension to couple the left and right-handed modes; the more crossings that are required the smaller the effect. For many physical quantities the leading effects of chiral symmetry breaking due to finite *N* are parameterized by a *residual* mass, $m_{\mathrm {res}}$. For example, the PCAC relation (for degenerate quarks of mass *m*) $\partial _\mu A_\mu (x) = 2m P(x)$, where $A_\mu$ and *P* represent the axial current and pseudoscalar density respectively, is satisfied with $m=m^\mathrm {DWF}+m_\mathrm {res}$, where $m^\mathrm {DWF}$ is the bare mass in the DWF action. The mixing of operators which transform under different representations of chiral symmetry is found to be negligibly small in current simulations. The important thing to note is that the chiral symmetry-breaking effects are small and that there are techniques to mitigate their consequences.

The main price which has to be paid for the good chiral symmetry is that the simulations are performed in 5 dimensions, requiring approximately a factor of N in computing resources and resulting in practice in ensembles at fewer values of the lattice spacing and quark masses than is possible with other formulations. The current generation of DWF simulations is being performed at physical quark masses so that ensembles with good chiral and flavour symmetries are being generated and analysed [31]. For a discussion of the equivalence of DWF and overlap fermions see Refs. [730, 731].

A third example of an operator which satisfies the Ginsparg–Wilson relation is the so-called fixed-point action [732–734]. This construction proceeds via a renormalization group approach. A related formalism are the so-called “chirally improved” fermions [735].

*Smearing:*

A simple modification which can help improve the action as well as the computational performance is the use of smeared gauge fields in the covariant derivatives of the fermionic action. Any smearing procedure is acceptable as long as it consists of only adding irrelevant (local) operators. Moreover, it can be combined with any discretization of the quark action. The “Asqtad” staggered quark action mentioned above [715] is an example which makes use of so-called “Asqtad” smeared (or “fat”) links. Another example is the use of n-HYP-smeared [478, 736], stout-smeared [737, 738] or HEX (hypercubic stout) smeared [739] gauge links in the tree-level clover improved discretization of the quark action, denoted by “n-HYP tlSW”, “stout tlSW” and “HEX tlSW” in the following.

In Table 50 we summarize the most widely used discretizations of the quark action and their main properties together with the abbreviations used in the summary tables. Note that in order to maintain the leading lattice artefacts of the actions as given in the table in nonspectral observables (like operator matrix elements) the corresponding nonspectral operators need to be improved as well.

**Table 50:** The most widely used discretizations of the quark action and some of their properties. Note that in order to maintain the leading lattice artefacts of the action in nonspectral observables (like operator matrix elements) the corresponding nonspectral operators need to be improved as well

Abbrev.	Discretization	Leading lattice artefacts	Chiral symmetry	Remarks
Wilson	Wilson	$\mathcal {O}(a)$	Broken	
tmWil	Twisted-mass Wilson	$\mathcal {O}(a^2)$ at maximal twist	Broken	Flavour-symmetry breaking: $(M_\text {PS}^{0})^2-(M_\text {PS}^\pm )^2\sim \mathcal {O}(a^2)$
tlSW	Sheikholeslami–Wohlert	$\mathcal {O}(g^2 a)$	Broken	Tree-level impr., $c_\mathrm{sw}=1$
n-HYP tlSW	Sheikholeslami–Wohlert	$\mathcal {O}(g^2 a)$	Broken	Tree-level impr., $c_\mathrm{sw}=1$, n-HYP-smeared gauge links
Stout tlSW	Sheikholeslami–Wohlert	$\mathcal {O}(g^2 a)$	Broken	Tree-level impr., $c_\mathrm{sw}=1$, stout-smeared gauge links
HEX tlSW	Sheikholeslami–Wohlert	$\mathcal {O}(g^2 a)$	Broken	Tree-level impr., $c_\mathrm{sw}=1$, HEX smeared gauge links
mfSW	Sheikholeslami–Wohlert	$\mathcal {O}(g^2 a)$	Broken	Mean-field impr.
npSW	Sheikholeslami–Wohlert	$\mathcal {O}(a^2)$	Broken	Nonperturbatively impr.
KS	Staggered	$\mathcal {O}(a^2)$	$\mathrm U(1)\times U(1)$ subgr. unbroken	Rooting for $N_{ f}<4$
Asqtad	Staggered	$\mathcal {O}(a^2)$	$\mathrm U(1)\times U(1)$ subgr. unbroken	Asqtad smeared gauge links, rooting for $N_{ f}<4$
HISQ	Staggered	$\mathcal {O}(a^2)$	$\mathrm U(1)\times U(1)$ subgr. unbroken	HISQ smeared gauge links, rooting for $N_{ f}<4$
DW	Domain Wall	Asymptotically $\mathcal {O}(a^2)$	Remnant breaking exponentially suppr.	Exact chiral symmetry and $\mathcal {O}(a)$ impr. only in the limit $N \rightarrow \infty$
oDW	Optimal Domain Wall	Asymptotically $\mathcal {O}(a^2)$	Remnant breaking exponentially suppr.	Exact chiral symmetry and $\mathcal {O}(a)$ impr. only in the limit $N \rightarrow \infty$
M-DW	Moebius Domain Wall	Asymptotically $\mathcal {O}(a^2)$	Remnant breaking exponentially suppr.	Exact chiral symmetry and $\mathcal {O}(a)$ impr. only in the limit $N \rightarrow \infty$
Overlap	Neuberger	$\mathcal {O}(a^2)$	Exact	

#### A.1.3 Heavy-quark actions

Charm and bottom quarks are often simulated with different lattice-quark actions than up, down, and strange quarks because their masses are large relative to typical lattice spacings in current simulations; for example, $a m_c \sim 0.4$ and $am_b \sim 1.3$ at $a=0.06$ fm. Therefore, for the actions described in the previous section, using a sufficiently small lattice spacing to control generic $(am_h)^n$ discretization errors is computationally costly, and in fact prohibitive at the physical *b*-quark mass.

One approach for lattice heavy quarks is direct application of effective theory. In this case the lattice heavy-quark action only correctly describes phenomena in a specific kinematic regime, such as Heavy-Quark Effective Theory (HQET) [740–742] or Nonrelativistic QCD (NRQCD) [743, 744]. One can discretize the effective Lagrangian to obtain, for example, Lattice HQET [470] or Lattice NRQCD [745, 746], and then simulate the effective theory numerically. The coefficients of the operators in the lattice-HQET and lattice-NRQCD actions are free parameters that must be determined by matching to the underlying theory (QCD) through the chosen order in $1/m_h$ or $v_h^2$, where $m_h$ is the heavy-quark mass and $v_h$ is the heavy-quark velocity in the heavy–light meson rest frame.

Another approach is to interpret a relativistic quark action such as those described in the previous section in a manner suitable for heavy quarks. One can extend the standard Symanzik improvement program, which allows one to systematically remove lattice cutoff effects by adding higher-dimension operators to the action, by allowing the coefficients of the dimension 4 and higher operators to depend explicitly upon the heavy-quark mass. Different prescriptions for tuning the parameters correspond to different implementations: those in common use are often called the Fermilab action [747], the relativistic heavy-quark action (RHQ) [475], and the Tsukuba formulation [748]. In the Fermilab approach, HQET is used to match the lattice theory to continuum QCD at the desired order in $1/m_h$.

More generally, effective theory can be used to estimate the size of cutoff errors from the various lattice heavy-quark actions. The power counting for the sizes of operators with heavy quarks depends on the typical momenta of the heavy quarks in the system. Bound-state dynamics differ considerably between heavy-heavy and heavy–light systems. In heavy–light systems, the heavy quark provides an approximately static source for the attractive binding force, like the proton in a hydrogen atom. The typical heavy-quark momentum in the bound-state rest frame is $|\vec {p}_h| \sim \Lambda _\mathrm{QCD}$, and heavy–light operators scale as powers of $(\Lambda _\mathrm{QCD}/m_h)^n$. This is often called “HQET power counting”, although it applies to heavy–light operators in HQET, NRQCD, and even relativistic heavy-quark actions described below. Heavy-heavy systems are similar to positronium or the deuteron, with the typical heavy-quark momentum $|\vec {p}_h| \sim \alpha _S m_h$. Therefore motion of the heavy quarks in the bound state rest frame cannot be neglected. Heavy-heavy operators have complicated power-counting rules in terms of $v_h^2$ [746]; this is often called “NRQCD power counting.”

Alternatively, one can simulate bottom or charm quarks with the same action as up, down, and strange quarks provided that (1) the action is sufficiently improved, and (2) the lattice spacing is sufficiently fine. These qualitative criteria do not specify precisely how large a numerical value of $am_h$ can be allowed while obtaining a given precision for physical quantities; this must be established empirically in numerical simulations. At present, both the HISQ and twisted-mass Wilson actions discussed previously are being used to simulate charm quarks. Simulations with HISQ quarks have employed heavier-quark masses than those with twisted-mass Wilson quarks because the action is more highly improved, but neither action can be used to simulate at the physical $am_b$ for current lattice spacings. Therefore calculations of heavy–light decay constants with these actions still rely on effective theory to reach the *b*-quark mass: the ETM Collaboration interpolates between twisted-mass Wilson data generated near $am_c$ and the static point [182], while the HPQCD Collaboration extrapolates HISQ data generated below $am_b$ up to the physical point using an HQET-inspired series expansion in $(1/m_h)^n$ [56].

*Heavy-quark effective theory:*

HQET was introduced by Eichten and Hill in Ref. [741]. It provides the correct asymptotic description of QCD correlation functions in the static limit $m_{h}/|\vec {p}_h| \!\rightarrow \! \infty$. Subleading effects are described by higher-dimensional operators whose coupling constants are formally of ${\mathcal {O}}((1/m_{h})^n)$. The HQET expansion works well for heavy–light systems in which the heavy-quark momentum is small compared to the mass.

The HQET Lagrangian density at the leading (static) order in the rest frame of the heavy quark is given by

282 $$\begin{aligned} {\mathcal L}^\mathrm{stat}(x) = \overline{\psi }_{h}(x) \,D_0\, \psi _{h}(x), \end{aligned}$$

with

283 $$\begin{aligned} P_+ \psi _{h} = \psi _{h},\quad \overline{\psi }_{h} P_+=\overline{\psi }_{h},\quad P_+={{1+\gamma _0}\over {2}}. \end{aligned}$$

A bare quark mass $m_\mathrm{bare}^\mathrm{stat}$ has to be added to the energy levels $E^\mathrm{stat}$ computed with this Lagrangian to obtain the physical ones. For example, the mass of the *B* meson in the static approximation is given by

284 $$\begin{aligned} m_{B} = E^\mathrm{stat} + m_\mathrm{bare}^\mathrm{stat}. \end{aligned}$$

At tree-level $m_\mathrm{bare}^\mathrm{stat}$ is simply the (static approximation of the) *b*-quark mass, but in the quantized lattice formulation it has to further compensate a divergence linear in the inverse lattice spacing. Weak composite fields are also rewritten in terms of the static fields, e.g.

285 $$\begin{aligned} A_0(x)^\mathrm{stat}=Z_\mathrm{A}^\mathrm{stat} (\overline{\psi }(x) \gamma _0\gamma _5\psi _h(x)), \end{aligned}$$

where the renormalization factor of the axial current in the static theory $Z_\mathrm{A}^\mathrm{stat}$ is scale-dependent. Recent lattice-QCD calculations using static *b* quarks and dynamical light quarks [182, 464] perform the operator matching at one-loop in mean-field improved lattice perturbation theory [749, 750]. Therefore the heavy-quark discretization, truncation, and matching errors in these results are of ${\mathcal {O}}(a^2 \Lambda _\mathrm{QCD}^2)$, ${\mathcal {O}} (\Lambda _\mathrm{QCD}/m_h)$, and ${\mathcal {O}}(\alpha _s^2, \alpha _s^2 a \Lambda _\mathrm{QCD})$.

In order to reduce heavy-quark truncation errors in *B*-meson masses and matrix elements to the few-percent level, state-of-the-art lattice-HQET computations now include corrections of ${\mathcal {O}}(1/m_h)$. Adding the $1/m_{h}$ terms, the HQET Lagrangian reads

286 $$\begin{aligned}&{\mathcal L}^\mathrm{HQET}(x)={\mathcal L}^\mathrm{stat}(x) - \omega _\mathrm {kin}{\mathcal {O}}_\mathrm{kin}(x) - \omega _\mathrm {spin}{\mathcal {O}}_\mathrm{spin}(x), \nonumber \\\end{aligned}$$

287 $$\begin{aligned}&\mathcal {O}_\mathrm{kin}(x)=\overline{\psi }_{h}(x)\mathbf{D}^2\psi _{h}(x),\quad \mathcal {O}_\mathrm{spin}(x) = \overline{\psi }_{h}(x){\varvec{\sigma }}\!\cdot \!\mathbf{B}\psi _{h}(x).\nonumber \\ \end{aligned}$$

At this order, two other parameters appear in the Lagrangian, $\omega _\mathrm {kin}$ and $\omega _\mathrm {spin}$. The normalization is such that the tree-level values of the coefficients are $\omega _\mathrm {kin}=\omega _\mathrm {spin}=1/(2m_{h})$. Similarly the operators are formally expanded in inverse powers of the heavy-quark mass. The time component of the axial current, relevant for the computation of mesonic decay constants is given by

288 $$\begin{aligned}&A_0^\mathrm{HQET}(x)=Z_\mathrm{A}^\mathrm{HQET}\left( A_0^\mathrm{stat}(x) +\sum _{i=1}^2 c_\mathrm{A}^{(i)} A_0^{(i)}(x)\right) , \end{aligned}$$

289 $$\begin{aligned}&A_0^{(1)}(x)=\overline{\psi }\frac{1}{2}\gamma _5 \gamma _k (\nabla _k-\overleftarrow{\nabla }_k)\psi _h(x), \quad k=1,2,3 \end{aligned}$$

290 $$\begin{aligned}&A_0^{(2)}=-\partial _kA_k^\mathrm{stat}(x), \quad A_k^\mathrm{stat}=\overline{\psi }(x) \gamma _k\gamma _5\psi _h(x), \end{aligned}$$

and depends on two additional parameters $c_\mathrm{A}^{(1)}$ and $c_\mathrm{A}^{(2)}$.

A framework for nonperturbative HQET on the lattice has been introduced in Refs. [470, 472]. As pointed out in Refs. [751, 752], since $\alpha _s(m_h)$ decreases logarithmically with $m_h$, whereas corrections in the effective theory are power-like in $\Lambda /m_h$, it is possible that the leading errors in a calculation will be due to the perturbative matching of the action and the currents at a given order $(\Lambda /m_h)^l$ rather than to the missing ${\mathcal {O}}((\Lambda /m_h)^{l+1})$ terms. Thus, in order to keep matching errors below the uncertainty due to truncating the HQET expansion, the matching is performed nonperturbatively beyond leading order in $1/m_{h}$. The asymptotic convergence of HQET in the limit $m_h \rightarrow \infty$ indeed holds only in that case.

The higher-dimensional interaction terms in the effective Lagrangian are treated as space-time volume insertions into static correlation functions. For correlators of some multi-local fields ${\mathcal {Q}}$ and up to the $1/m_h$ corrections to the operator, this means

291 $$\begin{aligned} \langle {\mathcal {Q}} \rangle= & {} \langle {\mathcal {Q}} \rangle _\mathrm{stat} +\omega _\mathrm {kin}a^4 \sum _x \langle {\mathcal {Q}\mathcal {O}}_\mathrm{kin}(x) \rangle _\mathrm{stat}\nonumber \\&+\, \omega _\mathrm {spin}a^4 \sum _x \langle {\mathcal {Q}\mathcal {O}}_\mathrm{spin}(x) \rangle _\mathrm{stat}, \end{aligned}$$

where $\langle {\mathcal {Q}} \rangle _\mathrm{stat}$ denotes the static expectation value with ${\mathcal {L}}^\mathrm{stat}(x) +{\mathcal {L}}^\mathrm{light}(x)$. Nonperturbative renormalization of these correlators guarantees the existence of a well-defined continuum limit to any order in $1/m_h$. The parameters of the effective action and operators are then determined by matching a suitable number of observables calculated in HQET (to a given order in $1/m_{h}$) and in QCD in a small volume (typically with $L\simeq 0.5$ fm), where the full relativistic dynamics of the *b*-quark can be simulated and the parameters can be computed with good accuracy. In Refs. [472, 473] the Schrödinger Functional (SF) setup has been adopted to define a set of quantities, given by the small volume equivalent of decay constants, pseudoscalar-vector splittings, effective masses and ratio of correlation functions for different kinematics, which can be used to implement the matching conditions. The kinematical conditions are usually modified by changing the periodicity in space of the fermions, i.e. by directly exploiting a finite-volume effect. The new scale *L*, which is introduced in this way, is chosen such that higher orders in $1/m_hL$ and in $\Lambda _\mathrm{QCD}/m_h$ are of about the same size. At the end of the matching step the parameters are known at lattice spacings which are of the order of 0.01 fm, significantly smaller than the resolutions used for large volume, phenomenological, applications. For this reason a set of SF-step-scaling functions is introduced in the effective theory to evolve the parameters to larger lattice spacings. The whole procedure yields the nonperturbative parameters with an accuracy which allows to compute phenomenological quantities with a precision of a few percent (see Refs. [459, 753] for the case of the $B_{(s)}$ decay constants). Such an accuracy cannot be achieved by performing the nonperturbative matching in large volume against experimental measurements, which in addition would reduce the predictivity of the theory. For the lattice-HQET action matched nonperturbatively through ${\mathcal {O}}(1/m_h)$, discretization and truncation errors are of ${\mathcal {O}}(a \Lambda ^2_\mathrm{QCD}/m_h, a^2 \Lambda ^2_\mathrm{QCD})$ and ${\mathcal {O}}((\Lambda _\mathrm{QCD}/m_h )^2)$.

The noise-to-signal ratio of static-light correlation functions grows exponentially in Euclidean time, $\propto e^{\mu x_0}$ . The rate $\mu$ is nonuniversal but diverges as 1 / *a* as one approaches the continuum limit. By changing the discretization of the covariant derivative in the static action one may achieve an exponential reduction of the noise to signal ratio. Such a strategy led to the introduction of the $S^\mathrm{stat}_\mathrm{HYP1,2}$ actions [486], where the thin links in $D_0$ are replaced by HYP-smeared links [478]. These actions are now used in all lattice applications of HQET.

*Nonrelativistic QCD:*

Nonrelativistic QCD (NRQCD) [745, 746] is an effective theory that can be matched to full QCD order by order in the heavy-quark velocity $v_h^2$ (for heavy-heavy systems) or in $\Lambda _\mathrm{QCD}/m_h$ (for heavy–light systems) and in powers of $\alpha _s$. Relativistic corrections appear as higher-dimensional operators in the Hamiltonian.

As an effective field theory, NRQCD is only useful with an ultraviolet cutoff of order $m_h$ or less. On the lattice this means that it can be used only for $am_h>1$, which means that $\mathcal {O}(a^n)$ errors cannot be removed by taking $a\rightarrow 0$ at fixed $m_h$. Instead heavy-quark discretization errors are systematically removed by adding additional operators to the lattice Hamiltonian. Thus, while strictly speaking no continuum limit exists at fixed $m_h$, continuum physics can be obtained at finite-lattice spacing to arbitrarily high precision provided enough terms are included, and provided that the coefficients of these terms are calculated with sufficient accuracy. Residual discretization errors can be parameterized as corrections to the coefficients in the nonrelativistic expansion, as shown in Eq. (294). Typically they are of the form $(a|\vec {p}_h|)^n$ multiplied by a function of $am_h$ that is smooth over the limited range of heavy-quark masses (with $am_h > 1$) used in simulations, and can therefore can be represented by a low-order polynomial in $am_h$ by Taylor’s theorem (see Ref. [754] for further discussion). Power-counting estimates of these effects can be compared to the observed lattice-spacing dependence in simulations. Provided that these effects are small, such comparisons can be used to estimate and correct the residual discretization effects.

An important feature of the NRQCD approach is that the same action can be applied to both heavy-heavy and heavy–light systems. This allows, for instance, the bare *b*-quark mass to be fixed via experimental input from $\Upsilon$ so that simulations carried out in the *B* or $B_s$ systems have no adjustable parameters left. Precision calculations of the $B_s$-meson mass (or of the mass splitting $M_{B_s} - M_\Upsilon /2$) can then be used to test the reliability of the method before turning to quantities one is trying to predict, such as decay constants $f_B$ and $f_{B_s}$, semileptonic form factors or neutral *B* mixing parameters.

Given the same lattice-NRQCD heavy-quark action, simulation results will not be as accurate for charm quarks as for bottom ($1/m_b < 1/m_c$, and $v_b < v_c$ in heavy-heavy systems). For charm, however, a more serious concern is the restriction that $am_h$ must be greater than one. This limits lattice-NRQCD simulations at the physical $am_c$ to relatively coarse lattice spacings for which light-quark and gluon discretization errors could be large. Thus recent lattice-NRQCD simulations have focussed on bottom quarks because $am_b > 1$ in the range of typical lattice spacings between $\approx$ 0.06 and 0.15 fm.

In most simulations with NRQCD *b*-quarks during the past decade one has worked with an NRQCD action that includes tree-level relativistic corrections through ${\mathcal {O}}(v_h^4)$ and discretization corrections through ${\mathcal {O}}(a^2)$,

292 $$\begin{aligned}&S_\mathrm{NRQCD} = a^4 \sum _x \left\{ {\Psi }^\dagger _t \Psi _t - {\Psi }^\dagger _t \left( 1 -\frac{a \delta H}{2}\right) _t \left( 1-\frac{aH_0}{2n}\right) ^{n}_t \right. \nonumber \\&\times \left. U^\dagger _t(t-a) \left( 1-\frac{aH_0}{2n}\right) ^{n}_{t-a} \left( 1-\frac{a\delta H}{2}\right) _{t-a} \Psi _{t-a} \right\} ,\nonumber \\ \end{aligned}$$

where the subscripts “*t*” and “$t-a$” denote that the heavy-quark, gauge, $\mathbf {E}$, and $\mathbf {B}$-fields are on time slices *t* or $t-a$, respectively. $H_0$ is the nonrelativistic kinetic energy operator,

293 $$\begin{aligned} H_0 = - {\Delta ^{(2)}\over 2m_h}, \end{aligned}$$

and $\delta H$ includes relativistic and finite-lattice-spacing corrections,

294 $$\begin{aligned} \delta H= & {} - c_1\,\frac{(\Delta ^{(2)})^2}{8m_h^3} + c_2\,\frac{i g}{8m_h^2}\left( \mathbf{\nabla }\cdot \tilde{\mathbf{E}}- \tilde{\mathbf{E}}\cdot \mathbf{\nabla }\right) \nonumber \\&-\,c_3\,\frac{g}{8m_h^2} \varvec{\sigma }\cdot (\tilde{\mathbf{\nabla }}\times \tilde{\mathbf{E}}- \tilde{\mathbf{E}}\times \tilde{\mathbf{\nabla }})\nonumber \\&-\, c_4\,\frac{g}{2m_h}\,\varvec{\sigma }\cdot \tilde{\mathbf{B}}+ c_5\,\frac{a^2{\Delta ^{(4)}}}{24m_h} - c_6\,\frac{a(\Delta ^{(2)})^2}{16nm_h^2}. \end{aligned}$$

$m_h$ is the bare heavy-quark mass, $\Delta ^{(2)}$ the lattice Laplacian, $\mathbf{\nabla }$ the symmetric lattice derivative and ${\Delta ^{(4)}}$ the lattice discretization of the continuum $\sum _i D^4_i$. $\tilde{\mathbf{\nabla }}$ is the improved symmetric lattice derivative and the $\tilde{\mathbf{E}}$ and $\tilde{\mathbf{B}}$ fields have been improved beyond the usual clover leaf construction. The stability parameter *n* is discussed in Ref. [746]. In most cases the $c_i$’s have been set equal to their tree-level values $c_i = 1$. With this implementation of the NRQCD action, errors in heavy–light-meson masses and splittings are of ${\mathcal {O}}(\alpha _S \Lambda _\mathrm{QCD}/m_h )$, ${\mathcal {O}}(\alpha _S (\Lambda _\mathrm{QCD}/m_h)^2 )$, ${\mathcal {O}}((\Lambda _\mathrm{QCD}/m_h )^3)$, and ${\mathcal {O}}(\alpha _s a^2 \Lambda _\mathrm{QCD}^2)$, with coefficients that are functions of $am_h$. One-loop corrections to many of the coefficients in Eq. (294) have now been calculated, and they are starting to be included in simulations [755–757].

Most of the operator matchings involving heavy–light currents or four-fermion operators with NRQCD *b*-quarks and AsqTad or HISQ light quarks have been carried out at one-loop order in lattice perturbation theory. In calculations published to date of electroweak matrix elements, heavy–light currents with massless light quarks have been matched through ${\mathcal {O}}(\alpha _s, \Lambda _\mathrm{QCD}/m_h, \alpha _s/(a m_h), \alpha _s \Lambda _\mathrm{QCD}/m_h)$, and four-fermion operators through ${\mathcal {O}}(\alpha _s, \Lambda _\mathrm{QCD}/m_h, \alpha _s/(a m_h))$. NRQCD/HISQ currents with massive HISQ quarks are also of interest, e.g. for the bottom-charm currents in $B \rightarrow D^{(*)}, l \nu$ semileptonic decays and the relevant matching calculations have been performed at one-loop order in Ref. [758]. Taking all the above into account, the most significant systematic error in electroweak matrix elements published to date with NRQCD *b*-quarks is the ${\mathcal {O}}(\alpha _s^2)$ perturbative matching uncertainty. Work is therefore under way to use current-current correlator methods combined with very high order continuum perturbation theory to do current matchings nonperturbatively [759].

*Relativistic heavy quarks:*

An approach for relativistic heavy-quark lattice formulations was first introduced by El-Khadra, Kronfeld, and Mackenzie in Ref. [747]. Here they showed that, for a general lattice action with massive quarks and non-Abelian gauge fields, discretization errors can be factorized into the form $f(m_h a)(a|\vec {p}_h|)^n$, and that the function $f(m_h a)$ is bounded to be of ${\mathcal {O}}(1)$ or less for all values of the quark mass $m_h$. Therefore cutoff effects are of ${\mathcal {O}}(a \Lambda _\mathrm{QCD})^n$ and ${\mathcal {O}}((a|\vec {p}_h|)^n)$, even for $am_h \gtrsim 1$, and can be controlled using a Symanzik-like procedure. As in the standard Symanzik improvement program, cutoff effects are systematically removed by introducing higher-dimension operators to the lattice action and suitably tuning their coefficients. In the relativistic heavy-quark approach, however, the operator coefficients are allowed to depend explicitly on the quark mass. By including lattice operators through dimension *n* and adjusting their coefficients $c_{n,i}(m_h a)$ correctly, one enforces that matrix elements in the lattice theory are equal to the analogous matrix elements in continuum QCD through $(a|\vec {p}_h|)^n$, such that residual heavy-quark discretization errors are of ${\mathcal {O}}(a|\vec {p}_h|)^{n+1}$.

The relativistic heavy-quark approach can be used to compute the matrix elements of states containing heavy quarks for which the heavy-quark spatial momentum $|\vec {p}_h|$ is small compared to the lattice spacing. Thus it is suitable to describe bottom and charm quarks in both heavy–light and heavy-heavy systems. Calculations of bottomonium and charmonium spectra serve as nontrivial tests of the method and its accuracy.

At fixed lattice spacing, relativistic heavy-quark formulations recover the massless limit when $(am_h) \ll 1$, recover the static limit when $(am_h) \gg 1$, and smoothly interpolate between the two; thus they can be used for any value of the quark mass, and, in particular, for both charm and bottom. Discretization errors for relativistic heavy-quark formulations are generically of the form $\alpha _s^k f(am_h)(a |\vec {p}_h|)^n$, where *k* reflects the order of the perturbative matching for operators of ${\mathcal {O}}((a |\vec {p}_h|)^n)$. For each *n*, such errors are removed completely if the operator matching is nonperturbative. When $(am_h) \sim 1$, this gives rise to nontrivial lattice-spacing dependence in physical quantities, and it is prudent to compare estimates based on power counting with a direct study of scaling behaviour using a range of lattice spacings. At fixed quark mass, relativistic heavy-quark actions possess a smooth continuum limit without power divergences. Of course, as $m_h \rightarrow \infty$ at fixed lattice spacing, the power divergences of the static limit are recovered (see, e.g. Ref. [760]).

The relativistic heavy-quark formulations in use all begin with the anisotropic Sheikholeslami–Wohlert (“clover”) action [761]:

295 $$\begin{aligned} S_{\text {lat}}= & {} a^4 \sum _{x,x'} \bar{\psi }(x') \left( m_0 + \gamma _0 D_0 + \zeta \vec {\gamma } \cdot \vec {D} - \frac{a}{2} (D^0)^2 \right. \nonumber \\&\left. -\, \frac{a}{2} \zeta (\vec {D})^2+ \sum _{\mu ,\nu } \frac{ia}{4} c_\mathrm{SW} \sigma _{\mu \nu } F_{\mu \nu } \right) _{x' x} \psi (x), \end{aligned}$$

where $D_\mu$ is the lattice covariant derivative and $F_{\mu \nu }$ is the lattice field-strength tensor. Here we show the form of the action given in Ref. [475]. The introduction of a space-time anisotropy, parameterized by $\zeta$ in Eq. (295), is convenient for heavy-quark systems because the characteristic heavy-quark four-momenta do not respect space-time axis exchange ($\vec {p}_h < m_h$ in the bound-state rest frame). Further, the Sheikoleslami–Wohlert action respects the continuum heavy-quark spin and flavour symmetries, so HQET can be used to interpret and estimate lattice discretization effects [760, 762, 763]. We discuss three different prescriptions for tuning the parameters of the action in common use below. In particular, we focus on aspects of the action and operator improvement and matching relevant for evaluating the quality of the calculations discussed in the main text.

The meson energy-momentum dispersion relation plays an important role in relativistic heavy-quark formulations:

296 $$\begin{aligned} E(\vec {p}) = M_1 + \frac{\vec {p}^2}{2M_2} + {\mathcal {O}}(\vec {p}^4), \end{aligned}$$

where $M_1$ and $M_2$ are known as the rest and kinetic masses, respectively. Because the lattice breaks Lorentz invariance, there are corrections proportional to powers of the momentum. Further, the lattice rest masses and kinetic masses are not equal ($M_1 \ne M_2$), and only become equal in the continuum limit.

The Fermilab interpretation [747] is suitable for calculations of mass splittings and matrix elements of systems with heavy quarks. The Fermilab action is based on the hopping-parameter form of the Wilson action, in which $\kappa _h$ parameterizes the heavy-quark mass. In practice, $\kappa _h$ is tuned such that the kinetic meson mass equals the experimentally measured heavy-strange meson mass ($m_{B_s}$ for bottom and $m_{D_s}$ for charm). In principle, one could also tune the anisotropy parameter such that $M_1 = M_2$. This is not necessary, however, to obtain mass splittings and matrix elements, which are not affected by $M_1$ [762]. Therefore in the Fermilab action the anisotropy parameter is set equal to unity. The clover coefficient in the Fermilab action is fixed to the value $c_\mathrm{SW} = 1/u_0^3$ from mean-field improved lattice perturbation theory [618]. With this prescription, discretization effects are of ${\mathcal {O}}(\alpha _sa|\vec {p}_h|, (a|\vec {p}_h|)^2)$. Calculations of electroweak matrix elements also require improving the lattice current and four-fermion operators to the same order, and matching them to the continuum. Calculations with the Fermilab action remove tree-level ${\mathcal {O}}(a)$ errors in electroweak operators by rotating the heavy-quark field used in the matrix element and setting the rotation coefficient to its tadpole-improved tree-level value (see e.g. Eqs. (7.8) and (7.10) of Ref. [747]). Finally, electroweak operators are typically renormalized using a mostly nonperturbative approach in which the flavour-conserving light-light and heavy-heavy current renormalization factors $Z_V^{ll}$ and $Z_V^{hh}$ are computed nonperturbatively [477]. The flavour-conserving factors account for most of the heavy–light current renormalization. The remaining correction is expected to be close to unity due to the cancellation of most of the radiative corrections including tadpole graphs [760]; therefore it can be reliably computed at one-loop in mean-field improved lattice perturbation theory with truncation errors at the percent to few-percent level.

The relativistic heavy-quark (RHQ) formulation developed by Li, Lin, and Christ builds upon the Fermilab approach, but tunes all the parameters of the action in Eq. (295) nonperturbatively [475]. In practice, the three parameters $\{m_0a, c_\mathrm{SW}, \zeta \}$ are fixed to reproduce the experimentally measured $B_s$ meson mass and hyperfine splitting ($m_{B_s^*}-m_{B_s}$), and to make the kinetic and rest masses of the lattice $B_s$ meson equal [476]. This is done by computing the heavy-strange meson mass, hyperfine splitting, and ratio $M_1/M_2$ for several sets of bare parameters $\{m_0a, c_\mathrm{SW}, \zeta \}$ and interpolating linearly to the physical $B_s$ point. By fixing the $B_s$-meson hyperfine splitting, one loses a potential experimental prediction with respect to the Fermilab formulation. However, by requiring that $M_1 = M_2$, one gains the ability to use the meson rest masses, which are generally more precise than the kinetic masses, in the RHQ approach. The nonperturbative parameter-tuning procedure eliminates ${\mathcal {O}}(a)$ errors from the RHQ action, such that discretization errors are of ${\mathcal {O}}((a|\vec {p}_h|)^2)$. Calculations of *B*-meson decay constants and semileptonic form factors with the RHQ action are in progress [764, 765], as is the corresponding one-loop mean-field improved lattice perturbation theory [766]. For these works, cutoff effects in the electroweak vector and axial-vector currents will be removed through ${\mathcal {O}}(\alpha _s a)$, such that the remaining discretization errors are of ${\mathcal {O}}(\alpha _s^2a|\vec {p}_h|, (a|\vec {p}_h|)^2)$. Matching the lattice operators to the continuum will be done following the mostly nonperturbative approach described above.

The Tsukuba heavy-quark action is also based on the Sheikholeslami–Wohlert action in Eq. (295), but allows for further anisotropies and hence has additional parameters: specifically the clover coefficients in the spatial $(c_B)$ and temporal $(c_E)$ directions differ, as do the anisotropy coefficients of the $\vec {D}$ and $\vec {D}^2$ operators [748]. In practice, the contribution to the clover coefficient in the massless limit is computed nonperturbatively [767], while the mass-dependent contributions, which differ for $c_B$ and $c_E$, are calculated at one-loop in mean-field improved lattice perturbation theory [768]. The hopping parameter is fixed nonperturbatively to reproduce the experimentally measured spin-averaged 1*S* charmonium mass [422]. One of the anisotropy parameters ($r_t$ in Ref. [422]) is also set to its one-loop perturbative value, while the other ($\nu$ in Ref. [422]) is fixed nonperturbatively to obtain the continuum dispersion relation for the spin-averaged charmonium 1*S* states (such that $M_1 = M_2$). For the renormalization and improvement coefficients of weak current operators, the contributions in the chiral limit are obtained nonperturbatively [95, 769], while the mass-dependent contributions are estimated using one-loop lattice perturbation theory [770]. With these choices, lattice cutoff effects from the action and operators are of ${\mathcal {O}}(\alpha _s^2 a|\vec {p}|, (a|\vec {p}_h|)^2)$.

**Table 51:** Discretizations of the quark action most widely used for heavy *c* and *b* quarks and some of their properties

Abbrev.	Discretization	Leading lattice artefacts and truncation errors for heavy–light mesons	Remarks
tmWil	Twisted-mass Wilson	${\mathcal {O}}\big ((am_h)^2\big )$	PCAC relation for axial-vector current
HISQ	Staggered	${\mathcal {O}}\big (\alpha _S (am_h)^2 (v/c), (am_h)^4 (v/c)^2 \big )$	PCAC relation for axial-vector current; Ward identity for vector current
Static	Static effective action	${\mathcal {O}}\big ( a^2 \Lambda _\mathrm{QCD}^2, \Lambda _\mathrm{QCD}/m_h, \alpha _s^2, \alpha _s^2 a \Lambda _\mathrm{QCD} \big )$	Implementations use APE, HYP1, and HYP2 smearing
HQET	Heavy-Quark Effective Theory	${\mathcal {O}}\big ( a \Lambda ^2_\mathrm{QCD}/m_h, a^2 \Lambda ^2_\mathrm{QCD}, (\Lambda _\mathrm{QCD}/m_h)^2 \big )$	Nonperturbative matching through ${\mathcal {O}}(1/m_h)$
NRQCD	Nonrelativistic QCD	${\mathcal {O}}\big (\alpha _S \Lambda _\mathrm{QCD}/m_h, \alpha _S (\Lambda _\mathrm{QCD}/m_h)^2 , (\Lambda _\mathrm{QCD}/m_h )^3, \alpha _s a^2 \Lambda _\mathrm{QCD}^2 \big )$	Tree-level relativistic corrections through ${\mathcal {O}}(v_h^4)$ and discretization corrections through ${\mathcal {O}}(a^2)$
Fermilab	Sheikholeslami–Wohlert	${\mathcal {O}}\big (\alpha _sa\Lambda _\mathrm{QCD}, (a\Lambda _\mathrm{QCD})^2\big )$	Hopping parameter tuned nonperturbatively; clover coefficient computed at tree-level in mean-field-improved lattice perturbation theory
RHQ	Sheikholeslami–Wohlert	${\mathcal {O}}\big ( \alpha _s^2 a\Lambda _\mathrm{QCD}, (a\Lambda _\mathrm{QCD})^2 \big )$	Hopping parameter, anisotropy and clover coefficient tuned nonperturbatively by fixing the $B_s$-meson hyperfine splitting
Tsukuba	Sheikholeslami–Wohlert	${\mathcal {O}}\big ( \alpha _s^2 a\Lambda _\mathrm{QCD}, (a\Lambda _\mathrm{QCD})^2 \big )$	NP clover coefficient at $ma=0$ plus mass-dependent corrections calculated at one-loop in lattice perturbation theory; $\nu$ calculated NP from dispersion relation; $r_s$ calculated at one-loop in lattice perturbation theory

*Light-quark actions combined with HQET:*

The heavy-quark formulations discussed in the previous sections use effective field theory to avoid the occurrence of discretization errors of the form $(am_h)^n$. In this section we describe methods that use improved actions that were originally designed for light-quark systems for *B* physics calculations. Such actions unavoidably contain discretization errors that grow as a power of the heavy-quark mass. In order to use them for heavy-quark physics, they must be improved to at least ${\mathcal {O}}(am_h)^2$. However, since $am_b > 1$ at the smallest lattice spacings available in current simulations, these methods also require input from HQET to guide the simulation results to the physical *b*-quark mass.

The ETM Collaboration has developed two methods, the “ratio method” [462] and the “interpolation method” [771, 772]. They use these methods together with simulations with twisted-mass Wilson fermions, which have discretization errors of $\mathcal {O}(am_h)^2$. In the interpolation method $\Phi _{hs}$ and $\Phi _{h\ell }$ (or $\Phi _{hs}/\Phi _{h\ell }$) are calculated for a range of heavy-quark masses in the charm region and above, while roughly keeping $am_h \mathop {\sim }\limits ^{\textstyle {<}}0.5$. The relativistic results are combined with a separate calculation of the decay constants in the static limit, and then interpolated to the physical *b* quark mass. In ETM’s implementation of this method, the heavy Wilson decay constants are matched to HQET using NLO in continuum perturbation theory. The static-limit result is renormalized using one-loop mean-field improved lattice perturbation theory, while for the relativistic data PCAC is used to calculate absolutely normalized matrix elements. Both, the relativistic and static-limit data are then run to the common reference scale $\mu _b = 4.5 \,\mathrm {GeV}$ at NLO in continuum perturbation theory. In the ratio method, one constructs physical quantities $P(m_h)$ from the relativistic data that have a well-defined static limit ($P(m_h) \rightarrow$ const. for $m_h \rightarrow \infty$) and evaluates them at the heavy-quark masses used in the simulations. Ratios of these quantities are then formed at a fixed ratio of heavy-quark masses, $z = P(m_h) / P(m_h/\lambda )$ (where $1< \lambda \,\mathop {\sim }\limits ^{{<}}\,1.3$), which ensures that *z* is equal to unity in the static limit. Hence, a separate static-limit calculation is not needed with this method. In ETM’s implementation of the ratio method for the *B*-meson decay constant, $P(m_h)$ is constructed from the decay constants and the heavy-quark pole mass as $P(m_h) = f_{h\ell }(m_h) \cdot (m^\mathrm{pole}_h)^{1/2}$. The corresponding *z*-ratio therefore also includes ratios of perturbative matching factors for the pole mass to ${\overline{\text {MS}}}$ conversion. For the interpolation to the physical *b*-quark mass, ratios of perturbative matching factors converting the data from QCD to HQET are also included. The QCD-to-HQET matching factors improve the approach to the static limit by removing the leading logarithmic corrections. In ETM’s implementation of this method (ETM 11 and 12) both conversion factors are evaluated at NLO in continuum perturbation theory. The ratios are then simply fit to a polynomial in $1/m_h$ and interpolated to the physical *b*-quark mass. The ratios constructed from $f_{h\ell }$ ($f_{hs}$) are called *z* ($z_s$). In order to obtain the *B*-meson-decay constants, the ratios are combined with relativistic decay constant data evaluated at the smallest reference mass.

The HPQCD Collaboration has introduced a method in Ref. [56] which we shall refer to as the “heavy HISQ” method. The first key ingredient is the use of the HISQ action for the heavy and light valence quarks, which has leading discretization errors of ${\mathcal {O}} \left( \alpha _s (v/c) (am_h)^2, (v/c)^2 (am_h)^4\right)$. With the same action for the heavy and light valence quarks it is possible to use PCAC to avoid renormalization uncertainties. Another key ingredient is the availability of gauge ensembles over a large range of lattice spacings, in this case in the form of the library of $N_f = 2+1$ asqtad ensembles made public by the MILC Collaboration which includes lattice spacings as small as $a \approx 0.045$ fm. Since the HISQ action is so highly improved and with lattice spacings as small as 0.045 fm, HPQCD is able to use a large range of heavy-quark masses, from below the charm region to almost up to the physical *b* quark mass with $am_h \mathop {\sim }\limits ^{\textstyle {<}}0.85$. They then fit their data in a combined continuum and HQET fit (i.e. using a fit function that is motivated by HQET) to a polynomial in $1/m_H$ (the heavy pseudo scalar meson mass of a meson containing a heavy (*h*) quark).

In Table 51 we list the discretizations of the quark action most widely used for heavy *c* and *b* quarks together with the abbreviations used in the summary tables. We also summarize the main properties of these actions and the leading lattice discretization errors for calculations of heavy–light meson matrix quantities with them. Note that in order to maintain the leading lattice artefacts of the actions as given in the table in nonspectral observables (like operator matrix elements) the corresponding nonspectral operators need to be improved as well.

### A.2 Setting the scale

In simulations of lattice QCD quantities such as hadron masses and decay constants are obtained in “lattice units” i.e. as dimensionless numbers. In order to convert them into physical units they must be expressed in terms of some experimentally known, dimensionful reference quantity *Q*. This procedure is called “setting the scale”. It amounts to computing the nonperturbative relation between the bare gauge coupling $g_0$ (which is an input parameter in any lattice simulation) and the lattice spacing *a* expressed in physical units. To this end one chooses a value for $g_0$ and computes the value of the reference quantity in a simulation: This yields the dimensionless combination, $(aQ)|_{g_0}$, at the chosen value of $g_0$. The calibration of the lattice spacing is then achieved via

297 $$\begin{aligned} a^{-1}\,[\mathrm{MeV}] = \frac{Q|_\mathrm{{exp}}\,[\mathrm{MeV}]}{(aQ)|_{g_0}}, \end{aligned}$$

where $Q|_\mathrm{{exp}}$ denotes the experimentally known value of the reference quantity. Common choices for *Q* are the mass of the nucleon, the $\Omega$ baryon or the decay constants of the pion and the kaon. Vector mesons, such as the $\rho$ or $K^*$-meson, are unstable and therefore their masses are not very well suited for setting the scale, despite the fact that they have been used over many years for that purpose.

Another widely used quantity to set the scale is the hadronic radius $r_0$, which can be determined from the force between static quarks via the relation [136]

298 $$\begin{aligned} F(r_0)r_0^2 = 1.65. \end{aligned}$$

If the force is derived from potential models describing heavy quarkonia, the above relation determines the value of $r_0$ as $r_0\approx 0.5$ fm. A variant of this procedure is obtained [565] by using the definition $F(r_1)r_1^2=1.00$, which yields $r_1\approx 0.32$ fm. It is important to realize that both $r_0$ and $r_1$ are not directly accessible in experiment, so that their values derived from phenomenological potentials are necessarily model-dependent. Inspite of the inherent ambiguity whenever hadronic radii are used to calibrate the lattice spacing, they are very useful quantities for performing scaling tests and continuum extrapolations of lattice data. Furthermore, they can be easily computed with good statistical accuracy in lattice simulations.

### A.3 Matching and running

The lattice formulation of QCD amounts to introducing a particular regularization scheme. Thus, in order to be useful for phenomenology, hadronic matrix elements computed in lattice simulations must be related to some continuum reference scheme, such as the ${\overline{\text {MS}}}$-scheme of dimensional regularization. The matching to the continuum scheme usually involves running to some reference scale using the renormalization group.

In principle, the matching factors which relate lattice matrix elements to the ${\overline{\text {MS}}}$-scheme, can be computed in perturbation theory formulated in terms of the bare coupling. It has been known for a long time, though, that the perturbative expansion is not under good control. Several techniques have been developed which allow for a nonperturbative matching between lattice regularization and continuum schemes, and they are briefly introduced here.

*Regularization-independent Momentum Subtraction:*

In the *Regularization-independent Momentum Subtraction* (“RI/MOM” or “RI”) scheme [384] a nonperturbative renormalization condition is formulated in terms of Green functions involving quark states in a fixed gauge (usually Landau gauge) at nonzero virtuality. In this way one relates operators in lattice regularization nonperturbatively to the RI scheme. In a second step one matches the operator in the RI scheme to its counterpart in the ${\overline{\text {MS}}}$-scheme. The advantage of this procedure is that the latter relation involves perturbation theory formulated in the continuum theory. The uncontrolled use of lattice perturbation theory can thus be avoided. A technical complication is associated with the accessible momentum scales (i.e. virtualities), which must be large enough (typically several $\mathrm{GeV}$) in order for the perturbative relation to ${\overline{\text {MS}}}$ to be reliable. The momentum scales in simulations must stay well below the cutoff scale (i.e. $2\pi$ over the lattice spacing), since otherwise large lattice artefacts are incurred. Thus, the applicability of the RI scheme traditionally relies on the existence of a “window” of momentum scales, which satisfy

299 $$\begin{aligned} \Lambda _\mathrm{QCD} \lesssim p \lesssim 2\pi a^{-1}. \end{aligned}$$

However, solutions for mitigating this limitation, which involve continuum limit, nonperturbative running to higher scales in the RI/MOM scheme, have recently been proposed and implemented [7, 8, 405, 773].

*Schrödinger functional:*

Another example of a nonperturbative matching procedure is provided by the Schrödinger functional (SF) scheme [153]. It is based on the formulation of QCD in a finite volume. If all quark masses are set to zero the box length remains the only scale in the theory, such that observables like the coupling constant run with the box size *L*. The great advantage is that the RG running of scale-dependent quantities can be computed nonperturbatively using recursive finite-size scaling techniques. It is thus possible to run nonperturbatively up to scales of, say, $100\,\mathrm{GeV}$, where one is sure that the perturbative relation between the SF and ${\overline{\text {MS}}}$-schemes is controlled.

*Perturbation theory:*

The third matching procedure is based on perturbation theory in which higher order are effectively resummed [618]. Although this procedure is easier to implement, it is hard to estimate the uncertainty associated with it.

*Mostly nonperturbative renormalization:*

Some calculations of heavy–light and heavy-heavy matrix elements adopt a mostly nonperturbative matching approach. Let us consider a weak decay process mediated by a current with quark flavours *h* and *q*, where *h* is the initial heavy quark (either bottom or charm) and *q* can be a light ($\ell = u,d$), strange, or charm quark. The matrix elements of lattice current $J_{hq}$ are matched to the corresponding continuum matrix elements with continuum current $\mathcal{J}_{hq}$ by calculating the renormalization factor $Z_{J_{hq}}$. The mostly nonperturbative renormalization method takes advantage of rewriting the current renormalization factor as the following product:

300 $$\begin{aligned} Z_{J_{hq}} = \rho _{J_{hq}} \sqrt{Z_{V^4_{hh}}Z_{V^4_{qq}}} \, \end{aligned}$$

The flavour-conserving renormalization factors $Z_{V^4_{hh}}$ and $Z_{V^4_{qq}}$ can be obtained nonperturbatively from standard heavy–light and light-light meson charge normalization conditions. $Z_{V^4_{hh}}$ and $Z_{V^4_{qq}}$ account for the bulk of the renormalization. The remaining correction $\rho _{J_{hq}}$ is expected to be close to unity because most of the radiative corrections, including self-energy corrections and contributions from tadpole graphs, cancel in the ratio [760, 763]. The one-loop coefficients of $\rho _{J_{hq}}$ have been calculated for heavy–light and heavy-heavy currents for Fermilab heavy and both (improved) Wilson light [760, 763] and asqtad light [774] quarks. In all cases the one-loop coefficients are found to be very small, yielding sub-percent to few percent level corrections.

In Table 52 we list the abbreviations used in the compilation of results together with a short description.

**Table 52:** The most widely used matching and running techniques

Abbrev.	Description
RI	Regularization-independent momentum subtraction scheme
SF	Schrödinger functional scheme
PT1$\ell$	Matching/running computed in perturbation theory at one loop
PT2$\ell$	Matching/running computed in perturbation theory at two loops
mNPR	Mostly nonperturbative renormalization

### A.4 Chiral extrapolation

As mentioned in the introduction, Symanzik’s framework can be combined with Chiral Perturbation Theory. The well-known terms occurring in the chiral effective Lagrangian are then supplemented by contributions proportional to powers of the lattice spacing *a*. The additional terms are constrained by the symmetries of the lattice action and therefore depend on the specific choice of the discretization. The resulting effective theory can be used to analyse the *a*-dependence of the various quantities of interest – provided the quark masses and the momenta considered are in the range where the truncated chiral perturbation series yields an adequate approximation. Understanding the dependence on the lattice spacing is of central importance for a controlled extrapolation to the continuum limit.

For staggered fermions, this program has first been carried out for a single staggered flavour (a single staggered field) [688] at $\mathcal {O}(a^2)$. In the following, this effective theory is denoted by S$\chi$PT. It was later generalized to an arbitrary number of flavours [329, 330], and to next-to-leading order [689]. The corresponding theory is commonly called Rooted Staggered chiral perturbation theory and is denoted by RS$\chi$PT.

For Wilson fermions, the effective theory has been developed in [327, 328, 775] and is called W$\chi$PT, while the theory for Wilson twisted-mass fermions [84, 776, 777] is termed tmW$\chi$PT.

Another important approach is to consider theories in which the valence- and sea-quark masses are chosen to be different. These theories are called *partially quenched*. The acronym for the corresponding chiral effective theory is PQ$\chi$PT [778–781].

Finally, one can also consider theories where the fermion discretizations used for the sea and the valence quarks are different. The effective chiral theories for these “mixed action” theories are referred to as MA$\chi$PT [274–277, 782–784].

*Finite-Volume Regimes of QCD:*

Once QCD with $N_{ f}$ nondegenerate flavours is regulated both in the UV and in the IR, there are $3+N_{ f}$ scales in play: The scale $\Lambda _\mathrm {QCD}$ that reflects “dimensional transmutation” (alternatively, one could use the pion decay constant or the nucleon mass, in the chiral limit), the inverse lattice spacing 1 / *a*, the inverse box size 1 / *L*, as well as $N_{ f}$ meson masses (or functions of meson masses) that are sensitive to the $N_{ f}$ quark masses, e.g. $M_\pi ^2$, $2M_K^2-M_\pi ^2$ and the spin-averaged masses of ${}^1S$ states of quarkonia.

**Table 53:** Summary of simulated lattice actions with $N_{ f}=2$ quark flavours

Collaboration	Refs.	$N_{ f}$	Gauge action	Quark action
ALPHA 01A, 04, 05, 12, 13A	[12, 135, 239, 588, 589]	2	Wilson	npSW
Aoki 94	[621]	2	Wilson	KS
Bernardoni 10	[345]	2	Wilson	npSW ${}^{\mathrm{a}}$
Bernardoni 11	[343]	2	Wilson	npSW
Brandt 13	[37]	2	Wilson	npSW
Boucaud 01B	[644]	2	Wilson	Wilson
CERN-TOV 06	[358]	2	Wilson	Wilson/npSW
CERN 08	[302]	2	Wilson	npSW
CP-PACS 01	[134]	2	Iwasaki	mfSW
Davies 94	[622]	2	Wilson	KS
Dürr 11	[132]	2	Wilson	npSW
Engel 14	[38]	2	Wilson	npSW
ETM 07, 07A, 08, 09, 09A-D, 10B, 10D, 10F, 11C, 12, 13, 13A	[11, 25, 32, 33, 36, 41, 83, 133, 215, 332, 342, 346, 462, 605, 654]	2	tlSym	tmWil
ETM 10A, 12D	[46, 401]	2	tlSym	tmWil$^\mathrm{b}$
ETMC 14D, 15A	[160, 333]	2	Iwasaki	tmWil with npSW
Gülpers 13, 15	[355, 356]	2	Wilson	npSW
Hasenfratz 08	[347]	2	tadSym	n-HYP tlSW
JLQCD 08	[409]	2	Iwasaki	overlap
JLQCD 02, 05	[141, 218]	2	Wilson	npSW
JLQCD/TWQCD 07, 08A, 10	[138, 338, 348]	2	Iwasaki	overlap
QCDSF 07, 13	[216, 353]	2	Wilson	npSW
QCDSF/UKQCD 04, 06, 06A, 07	[137, 139, 241, 363]	2	Wilson	npSW
RBC 04, 06, 07	[105, 217, 400]	2	DBW2	DW
RBC/UKQCD 07	[214]	2	Wilson	npSW
RM123 11, 13	[16, 167]	2	tlSym	tmWil
Sesam 99	[623]	2	Wilson	Wilson
Sternbeck 10, 12	[652, 653]	2	Wilson	npSW
SPQcdR 05	[140]	2	Wilson	Wilson
TWQCD 11, 11A	[249, 344]	2	Wilson	optimal DW
UKQCD 04	[214, 410]	2	Wilson	npSW
Wingate 95	[624]	2	Wilson	KS

${}^{\mathrm{a}}$ The calculation uses overlap fermions in the valence quark sector

${}^\mathrm{b}$ The calculation uses Osterwalder–Seiler fermions [427] in the valence quark sector

Ultimately, we are interested in results with the two regulators removed, i.e. physical quantities for which the limits $a \rightarrow 0$ and $L \rightarrow \infty$ have been carried out. In both cases there is an effective field theory (EFT) which guides the extrapolation. For the $a \rightarrow 0$ limit, this is a version of the Symanzik EFT which depends, in its details, on the lattice action that is used, as outlined in Sect. A.1. The finite-volume effects are dominated by the lightest particles, the pions. Therefore, a chiral EFT, also known as $\chi$PT, is appropriate to parameterize the finite-volume effects, i.e. the deviation of masses and other observables, such as matrix elements, in a finite volume from their infinite-volume, physical values. Most simulations of phenomenological interest are carried out in boxes of size $L \gg 1/M_\pi$, that is, in boxes whose diameter is large compared to the Compton wavelength that the pion would have, at the given quark mass, in infinite volume. In this situation the finite-volume corrections are small, and in many cases the ratio $M_\mathrm {had}(L) / M_\mathrm {had}$ or *f*(*L*) / *f*, where *f* denotes some generic matrix element, can be calculated in $\chi$PT, such that the leading finite-volume effects can be taken out analytically. In the terminology of $\chi$PT this setting is referred to as the *p*-regime, as the typical contributing momenta $p \sim M_\pi \gg 1/L$. A peculiar situation occurs if the condition $L \gg 1/M_\pi$ is violated (while $L\Lambda _\mathrm {QCD}\gg 1$ still holds), in other words if the quark mass is taken so light that the Compton wavelength that the pion would have (at the given $m_q$) in infinite volume, is as large or even larger than the actual box size. Then the pion zero-momentum mode dominates and needs to be treated separately. While this setup is unlikely to be useful for standard phenomenological computations, the low-energy constants of $\chi$PT can still be calculated, by matching to a re-ordered version of the chiral series, and following the details of the reordering such an extreme regime is called the $\epsilon$- or $\delta$-regime, respectively. Accordingly, further particulars of these regimes are discussed in Sect. 5.1 of this report.

### A.5 Summary of simulated lattice actions

In the following tables 53, 54, 55, 56 and 57 we summarize the gauge and quark actions used in the various calculations with $N_f=2, 2+1$ and $2+1+1$ quark flavours. The calculations with $N_f=0$ quark flavours mentioned in Sect. 9 all used the Wilson gauge action and are not listed. Abbreviations are explained in Sects. A.1.1, A.1.2 and A.1.3, and summarized in Tables 49, 50 and 51.

**Table 54:** Summary of simulated lattice actions with $N_{ f}=2+1$ or $N_{ f}=3$ quark flavours

Collaboration	Refs.	$N_{ f}$	Gauge action	Quark action
Aubin 08, 09	[236, 406]	$2+1$	tadSym	Asqtad${}^{\mathrm{a}}$
Blum 10	[103]	$2+1$	Iwasaki	DW
BMW 10A-C, 11, 13	[7, 8, 35, 43, 115]	$2+1$	tlSym	2-level HEX tlSW
BMW 10	[30]	$2+1$	tlSym	6-level stout tlSW
Boyle 14	[372]	$2+1$	Iwasaki, Iwasaki $+$ DSDR	DW
CP-PACS/JLQCD 07	[146]	$2+1$	Iwasaki	npSW
FNAL/MILC 12, 12I	[23, 60]	$2+1$	tadSym	Asqtad
HPQCD 05, 05A, 08A, 13A	[26, 147, 616, 617]	$2+1$	tadSym	Asqtad
HPQCD 10	[9]	$2+1$	tadSym	Asqtad${}^\mathrm{b}$
HPQCD/UKQCD 06	[408]	$2+1$	tadSym	Asqtad
HPQCD/UKQCD 07	[28]	$2+1$	tadSym	Asqtad${}^\mathrm{b}$
HPQCD/MILC/UKQCD 04	[148]	$2+1$	tadSym	Asqtad
JLQCD 09, 10	[337, 613]	$2+1$	Iwasaki	Overlap
JLQCD 11, 12, 14, 15A	[211, 212, 359, 360]	$2+1$	Iwasaki (fixed topology)	Overlap
JLQCD 15B	[174]	$2+1$	Iwasaki	M-DW
JLQCD/TWQCD 08B, 09A	[235, 341]	$2+1$	Iwasaki	Overlap
JLQCD/TWQCD 10	[338]	$2+1, 3$	Iwasaki	Overlap
Laiho 11	[44]	$2+1$	tadSym	Asqtad${}^{\mathrm{a}}$
LHP 04	[362]	$2+1$	tadSym	Asqtad$^{\mathrm{a}}$
Maltman 08	[63]	$2+1$	tadSym	Asqtad
MILC 04, 07, 09, 09A, 10, 10A	[13, 29, 89, 107, 148, 785]	$2+1$	tadSym	Asqtad
NPLQCD 06	[238]	$2+1$	tadSym	Asqtad$^{\mathrm{a}}$
PACS-CS 08, 08A, 09, 09A, 10, 11A, 12	[62, 93–95, 237, 361]	$2+1$	Iwasaki	npSW
QCDSF/UKQCD 15	[166]	$2+1$	tlSym	npSW
RBC/UKQCD 07, 08, 08A, 10, 10A-B, 11, 12, 13	[31, 144, 145, 210, 213, 339, 405, 407, 786]	$2+1$	Iwasaki, Iwasaki $+$ DSDR	DW
RBC/UKQCD 12E	[412]	$2+1$	Iwasaki	DW
RBC/UKQCD 14B, 15A, 15E	[10, 24, 335]	$2+1$	Iwasaki, Iwasaki $+$ DSDR	DW, M-DW
Sternbeck 12	[652]	$2+1$	tlSym	npSW
SWME 10, 11, 11A, 13, 13A, 14A, 14C, 15A	[45, 278, 385, 402–404, 417, 787]	$2+1$	tadSym	Asqtad${}^\mathrm{c}$
TWQCD 08	[340]	$2+1$	Iwasaki	DW

${}^{\mathrm{a}}$ The calculation uses domain-wall fermions in the valence-quark sector

${}^\mathrm{b}$ The calculation uses HISQ staggered fermions in the valence-quark sector

${}^\mathrm{c}$ The calculation uses HYP-smeared improved staggered fermions in the valence-quark sector

**Table 55:** Summary of simulated lattice actions with $N_{ f}=4$ or $N_{ f}=2+1+1$ quark flavours

Collaboration	Refs.	$N_{ f}$	Gauge action	Quark action
ALPHA 10A	[586]	4	Wilson	npSW
Bazavov 12	[604]	$2+1+1$	tlSym	HISQ
ETM 10, 10E, 11, 11D, 12C, 13, 13A, 13D	[33, 39, 233, 332, 352, 649–651]	$2+1+1$	Iwasaki	tmWil
ETM 14A, 14B, 15, 15C	[42, 176, 180, 208]	$2+1+1$	Iwasaki	tmWil${}^{\mathrm{a}}$
FNAL/MILC 12B, 13, 13C, 13E, 14A	[14, 22, 209, 420, 421]	$2+1+1$	tadSym	HISQ
HPQCD 14A, 15B	[5, 336]	$2+1+1$	tadSym	HISQ
MILC 13A	[231]	$2+1+1$	tadSym	HISQ
Perez 10	[587]	4	Wilson	npSW

${}^{\mathrm{a}}$ The calculation uses Osterwalder–Seiler fermions [427] in the valence-quark sector

**Table 56:** Summary of lattice simulations $N_f=2$ sea-quark flavours and with *b* and *c* valence quarks

Collaboration	Refs.	$N_{ f}$	Gauge action	Quark actions		
				Sea	Light valence	Heavy
ALPHA 11, 12A, 13, 14, 14B	[57, 458, 459, 461, 512]	2	Plaquette	npSW	npSW	HQET
ALPHA 13C	[177]	2	Plaquette	npSW	npSW	npSW
Atoui 13	[537]	2	tlSym	tmWil	tmWil	tmWil
ETM 09, 09D, 11B, 12A, 12B, 13B, 13C	[20, 32, 58, 431, 460, 462, 485]	2	tlSym	tmWil	tmWil	tmWil
ETM 11A	[182]	2	tlSym	tmWil	tmWil	tmWil, static
TWQCD 14	[424]	2	Plaquette	oDW	oDW	oDW

**Table 57:** Summary of lattice simulations with $N_f=2+1$ or $N_f=2+1+1$ sea-quark flavours and *b* and *c* valence quarks

Collaboration	Refs.	$N_{ f}$	Gauge action	Quark actions		
				Sea	Light valence	Heavy
$\chi$QCD 14	[17]	$2+1$	Iwasaki	DW	Overlap	Overlap
FNAL/MILC 04, 04A, 05, 08, 08A, 10, 11, 11A, 12, 13B	[48, 60, 423, 437, 441, 483, 535, 536, 538, 788]	$2+1$	tadSym	Asqtad	Asqtad	Fermilab
FNAL/MILC 14, 15C	[539, 540]	$2+1$	tadSym	Asqtad	Asqtad${}^{\mathrm{a}}$	Fermilab${}^{\mathrm{a}}$
FNAL/MILC 15	[504]	$2+1$	tadSym	Asqtad	Asqtad	Fermilab
HPQCD 06, 06A, 08B, 09, 13B	[59, 152, 181, 484, 503]	$2+1$	tadSym	Asqtad	Asqtad	NRQCD
HPQCD 12	[55]	$2+1$	tadSym	Asqtad	HISQ	NRQCD
HPQCD 15	[541]	$2+1$	tadSym	Asqtad	HISQ${}^{\mathrm{b}}$	NRQCD${}^{\mathrm{b}}$
HPQCD/UKQCD 07, HPQCD 10A, 10B, 11, 11A, 12A, 13C	[28, 47, 49–51, 56, 434]	$2+1$	tadSym	Asqtad	HISQ	HISQ
PACS-CS 11	[422]	$2+1$	Iwasaki	npSW	npSW	Tsukuba
RBC/UKQCD 10C, 14A	[54, 464]	$2+1$	Iwasaki	DW	DW	Static
RBC/UKQCD 13A, 14, 15	[53, 457, 505]	$2+1$	Iwasaki	DW	DW	RHQ
ETM 13E, 13F, 14E	[27, 230, 456]	$2+1+1$	Iwasaki	tmWil	tmWil	tmWil
FNAL/MILC 12B, 13, 14A	[14, 420, 421]	$2+1+1$	tadSym	HISQ	HISQ	HISQ
HPQCD 13	[52]	$2+1+1$	tadSym	HISQ	HISQ	NRQCD

$^{\mathrm{a}}$ Asqtad for *u*, *d* and *s* quark; Fermilab for *b* and *c* quark

$^{\mathrm{b}}$ HISQ for *u*, *d*, *s* and *c* quark; NRQCD for *b* quark

**Table 58:** Continuum extrapolations/estimation of lattice artefacts in determinations of $m_{ud}$, $m_s$ and, in some cases $m_u$ and $m_d$, with $N_f=2+1+1$ quark flavours

Collaboration	Refs.	$N_{ f}$	*a* [fm]	Description
HPQCD 14A	[5]	$2+1+1$	0.15, 0.12, 0.09, 0.06	Scale set through the Wilson flow parameter $w_0$
FNAL/MILC 14A	[14]	$2+1+1$	0.06, 0.09, 0.12, 0.15	HISQ action for both valence and sea quarks. Absolute scale though $f_\pi$
ETM 14	[4]	$2+1+1$	0.062, 0.082, 0.089	Scale set through $f_\pi$. Automatic $\mathcal {O}(a)$ improvement, flavour symmetry breaking: $(M_{PS}^{0})^2-(M_{PS}^\pm )^2\sim \mathcal {O}(a^2)$. Discretization and volume effects due to the $\pi ^0$–$\pi ^\pm$ mass splitting are taken into account through $\chi$PT for twisted-mass fermions

## Appendix B: Notes

### B.1 Notes to Sect. 3 on quark masses

See Tables 58, 59, 60, 61, 62, 63, 64, 65, 66, 67, 68, 69, 70, 71, 72, 73, 74, 75, 76, 77.

**Table 59:** Continuum extrapolations/estimation of lattice artefacts in determinations of $m_{ud}$, $m_s$ and, in some cases $m_u$ and $m_d$, with $N_f=2+1$ quark flavours

Collaboration	Refs.	$N_{ f}$	*a* [fm]	Description
QCDSF/UKQCD 15	[166]	$2+1$	0.07	Scale set through the gradient flow parameter $w_0$
RBC/UKQCD 14B	[10]	$2+1$	0.063, 0.084, 0.114, 0.144	Scale set through $M_\Omega$
RBC/UKQCD 12	[31]	$2+1$	0.085, 0.113, 0.144	Scale set through $M_\Omega$
PACS-CS 12	[143]	$1+1+1$	0.09	Reweighting of PACS-CS 08 $N_f=2+1$ QCD configurations with e.m. and $m_u\ne m_d$
Laiho 11	[44]	$2+1$	0.06, 0.09, 0.15	MILC staggered ensembles [13], scale set using $r_1$ determined by HPQCD with $\Upsilon$ splittings, pseudoscalar decay constants, through $r_1$ [250]
PACS-CS 10	[95]	$2+1$	0.09	cf. PACS-CS 08
MILC 10A	[13]	$2+1$		cf. MILC 09, 09A
BMW 10A, 10B	[7, 8]	$2+1$	0.054, 0.065, 0.077, 0.093, 0.116	Scale set via $M_\pi , M_K, M_\Omega$
RBC/UKQCD 10A	[144]	$2+1$	0.114, 0.087	Scale set through $M_\Omega$
Blum 10	[103]	$2+1$	0.11	Relies on RBC/UKQCD 08 scale setting
PACS-CS 09	[94]	$2+1$	0.09	Scale setting via $M_\Omega$
HPQCD 09A, 10	[9, 18]	$2+1$	0.045, 0.06, 0.09, 0.12, 0.15	Scale set through $r_1$ and $\Upsilon$ and continuum extrapolation based on RS$\chi$PT. See MILC 09 for details
MILC 09A, 09	[6, 89]	$2+1$	0.045, 0.06, 0.09	Scale set through $r_1$ and $\Upsilon$ and continuum extrapolation based on RS$\chi$PT
PACS-CS 08	[93]	$2+1$	0.09	Scale set through $M_\Omega$. Nonperturbatively $\mathcal {O}(a)$-improved
RBC/UKQCD 08	[145]	$2+1$	0.11	Scale set through $M_\Omega$. Automatic $\mathcal {O}(a)$-improvement due to approximate chiral symmetry. $(\Lambda _\mathrm{QCD}a)^2\approx 4\%$ systematic error due to lattice artefacts added
CP-PACS/JLQCD 07	[146]	$2+1$	0.07, 0.10, 0.12	Scale set through $M_K$ or $M_\phi$. Nonperturbatively $\mathcal {O}(a)$-improved
HPQCD 05	[147]	$2+1$	0.09, 0.12	Scale set through the $\Upsilon -\Upsilon '$ mass difference
HPQCD/MILC/UKQCD 04, MILC 04	[107, 148]	$2+1$	0.09, 0.12	Scale set through $r_1$ and $\Upsilon$ and continuum extrapolation based on RS$\chi$PT

**Table 60:** Continuum extrapolations/estimation of lattice artefacts in determinations of $m_{ud}$, $m_s$ and, in some cases $m_u$ and $m_d$, with $N_f=2$ quark flavours

Collaboration	Refs.	$N_{ f}$	*a* [fm]	Description
ETM 14D	[160]	2	0.094	Scale set through $F_\pi$, $r_0$, $t_0$ and $w_0$. Twisted Wilson fermions plus clover term. Automatic $\mathcal {O}(a)$ improvement
RM123 13	[16]	2	0.098, 0.085, 0.067, 0.054	cf. ETM 10B
ALPHA 12	[12]	2	0.076, 0.066, 0.049	Scale set through $F_K$
RM123 11	[167]	2	0.098, 0.085, 0.067, 0.054	cf. ETM 10B
Dürr 11	[132]	2	0.076, 0.072, 0.060	Scale for light-quark masses set through $m_c$
ETM 10B	[11]	2	0.098, 0.085, 0.067, 0.054	Scale set through $F_\pi$
JLQCD/TWQCD 08A	[138]	2	0.12	Scale set through $r_0$
RBC 07	[105]	2	0.12	Scale set through $M_\rho$
ETM 07	[133]	2	0.09	Scale set through $F_\pi$
QCDSF/UKQCD 06	[139]	2	0.065–0.09	Scale set through $r_0$
SPQcdR 05	[140]	2	0.06, 0.08	Scale set through $M_{K^*}$
ALPHA 05	[135]	2	0.07–0.12	Scale set through $r_0$
QCDSF/UKQCD 04	[137]	2	0.07–0.12	Scale set through $r_0$
JLQCD 02	[141]	2	0.09	Scale set through $M_\rho$
CP-PACS 01	[134]	2	0.11, 0.16, 0.22	Scale set through $M_\rho$

**Table 61:** Chiral-extrapolation/minimum pion mass in determinations of $m_{ud}$, $m_s$ and, in some cases, $m_u$ and $m_d$, with $N_f=2+1+1$ quark flavours

Collaboration	Refs.	$N_{ f}$	$M_{\pi ,{\mathrm{min}}}$ [MeV]	Description
HPQCD 14A	[5]	$2+1+1$	$128_{\pi , 5}$ ($173_\mathrm{RMS}$)	Sea quark masses linearly extrapolated/interpolated to physical values. $m_s$ determined from physical $m_s/m_c$ and $m_c$
FNAL/MILC 14A	[14]	$2+1+1$	$128_{\pi ,5}$ ($143_\mathrm{RMS}$)	Linear interpolation to physical point. The lightest RMS mass is from the $a=0.06$ fm ensemble and the lightest Nambu–Goldstone mass is from the $a=0.09$ fm ensemble
ETM 14	[4]	$2+1+1$	$180_\mathrm{\pi ^0}(220_{\pi ^\pm })$	Chiral extrapolation performed through *SU*(2) $\chi$PT or polynomial fit

**Table 62:** Chiral-extrapolation/minimum pion mass in determinations of $m_{ud}$, $m_s$ and, in some cases $m_u$ and $m_d$, with $N_f=2+1$ quark flavours

Collaboration	Refs.	$N_{ f}$	$M_{\pi ,{\mathrm{min}}}$ [MeV]	Description
QCDSF/UKQCD 15	[166]	$2+1$	205 (val.)	Expansion around the symmetric point $m_u = m_d = m_s$
RBC/UKQCD 14B	[10]	$2+1$	139	NLO PQ *SU*(2) $\chi$PT as well as analytic ansätze
RBC/UKQCD 12	[31]	$2+1$	170	Combined fit to Iwasaki and Iwasaki $+$ DSDR gauge action ensembles
PACS-CS 12	[143]	$1+1+1$		cf. PACS-CS 08
Laiho 11	[44]	$2+1$	210 (val.) 280 (sea-RMS)	NLO *SU*(3), mixed-action $\chi$PT [276], with N$^2$LO-N$^4$LO analytic terms
PACS-CS 10	[95]	$2+1$		cf. PACS-CS 08
MILC 10A	[13]	$2+1$		NLO *SU*(2) S$\chi$PT. cf. also MILC 09A, 09
BMW 10A, 10B	[7, 8]	$2+1$	135	Interpolation to the physical point
RBC/UKQCD 10A	[144]	$2+1$	290	NLO PQ *SU*(2) $\chi$PT as well as analytic ansätze
Blum 10	[103, 145]	$2+1$	242 (valence), 330 (sea)	Extrapolation done on the basis of PQ$\chi$PT formulae with virtual photons
PACS-CS 09	[94]	$2+1$	135	Physical point reached by reweighting technique, no chiral extrapolation needed
HPQCD 09A, 10	[9, 18]	$2+1$		cf. MILC 09
MILC 09A, 09	[6, 89]	$2+1$	177, 224	NLO *SU*(3) RS$\chi$PT, continuum $\chi$PT at NNLO and NNNLO and NNNNLO analytic terms. The lightest Nambu–Goldstone mass is 177 MeV (09A) and 224 MeV (09) (at $a=0.09$ fm) and the lightest RMS mass is 258 MeV (at $a=0.06$ fm)
PACS-CS 08	[93]	$2+1$	156	NLO *SU*(2) $\chi$PT and *SU*(3) (Wilson)$\chi$PT
RBC/UKQCD 08	[145]	$2+1$	242 (valence), 330 (sea)	*SU*(3) PQ$\chi$PT and heavy kaon NLO *SU*(2) PQ$\chi$PT fits
CP-PACS/JLQCD 07	[146]	$2+1$	620	NLO Wilson $\chi$PT fits to meson masses
HPQCD 05	[147]	$2+1$	240	PQ RS$\chi$PT fits
HPQCD/MILC/UKQCD 04, MILC 04	[107, 148]	$2+1$	240	PQ RS$\chi$PT fits

**Table 63:** Chiral-extrapolation/minimum pion mass in determinations of $m_{ud}$, $m_s$ and, in some cases $m_u$ and $m_d$, with $N_f=2$ quark flavours

Collaboration	Refs.	$N_{ f}$	$M_{\pi ,{\mathrm{min}}}$ [MeV]	Description
ETM 14D	[160]	2	140	Charged/neutral pion-mass breaking, $M_{\pi ^pm}^2-M_{\pi ^0}^2 \sim \mathcal {O}(a^2)$, estimated to be ${\simeq } 20$ MeV
RM123 13	[16]	2	270	Fits based on NLO $\chi$PT and Symanzik expansion up to $\mathcal {O}(a^2)$. $\mathcal {O}(\alpha )$ e.m. effects included
ALPHA 12	[12]	2	270	NLO *SU*(2) and *SU*(3) $\chi$PT and $\mathcal {O}(a^2)$ on LO LEC
RM123 11	[167]	2	270	Fits based on NLO $\chi$PT and Symanzik expansion up to $\mathcal {O}(a^2)$
Dürr 11	[132]	2	285	$m_c/m_s$ determined by quadratic or cubic extrapolation in $M_\pi$
ETM 10B	[11]	2	270	Fits based on NLO $\chi$PT and Symanzik expansion up to $\mathcal {O}(a^2)$
JLQCD/TWQCD 08A	[138]	2	290	NLO $\chi$PT fits
RBC 07	[105]	2	440	NLO fit including $\mathcal {O}(\alpha )$ effects
ETM 07	[133]	2	300	Polynomial and PQ$\chi$PT fits
QCDSF/UKQCD 06	[139]	2	520 (valence), 620 (sea)	NLO (PQ)$\chi$PT fits
SPQcdR 05	[140]	2	600	Polynomial fit
ALPHA 05	[135]	2	560	LO $\chi$PT fit
QCDSF/UKQCD 04	[137]	2	520 (valence), 620 (sea)	NLO (PQ)$\chi$PT fits
JLQCD 02	[141]	2	560	Polynomial and $\chi$PT fits
CP-PACS 01	[134]	2	430	Polynomial fits

**Table 64:** Finite-volume effects in determinations of $m_{ud}$, $m_s$ and, in some cases $m_u$ and $m_d$, with $N_f=2+1+1$ quark flavours

Collaboration	Refs.	$N_{ f}$	*L* [fm]	$M_{\pi ,\text {min}} L$	Description
HPQCD 14A	[5]	$2+1+1$	2.5–5.8	3.7	
FNAL/MILC 14A	[14]	$2+1+1$	2.8–5.8	$3.9_\mathrm{RMS}(3.7_{\pi ,5})$	Includes error estimate from NNLO S$\chi$PT
ETM 14	[4]	$2+1+1$	2.0–3.0	$2.7_{\pi ^0}$ ($3.3_{\pi ^\pm }$)	FV effect for the pion is corrected through resummed NNLO $\chi$PT for twisted-mass fermions, which takes into account the effects due to the $\pi ^0$–$\pi ^\pm$ mass splitting

**Table 65:** Finite-volume effects in determinations of $m_{ud}$, $m_s$ and, in some cases $m_u$ and $m_d$, with $N_f=2+1$ quark flavours

Collaboration	Refs.	$N_{ f}$	*L* [fm]	$M_{\pi ,{\mathrm{min}}} L$	Description
QCDSF/UKQCD 15	[166]	$2+1$	1.7, 2.2, 3.4		Effective field theory used to extrapolate to infinite volume
RBC/UKQCD 14B	[10]	$2+1$	2.0, 2.7, 4.6, 5.4	3.8	Uses FV chiral perturbation theory to estimate the error, which is deemed negligible and omitted
RBC/UKQCD 12	[31]	$2+1$	2.7, 4.6	${\gtrsim } 4.0$	Uses FV chiral perturbation theory to estimate the error
PACS-CS 12	[143]	$1+1+1$			cf. PACS-CS 08
Laiho 11	[44]	$2+1$	2.5, 2.9, 3.0, 3.6, 3.8, 4.8	4.1 (val.) 4.1 (sea)	Data corrected using NLO *SU*(3) $\chi$PT finite-V formulae
PACS-CS 10	[95]	$2+1$			cf. PACS-CS 08
MILC 10A	[13]	$2+1$			cf. MILC 09A, 09
BMW 10A, 10B	[7, 8]	$2+1$	${\gtrsim } 5.0$	${\gtrsim } 4.0$	FV corrections below 5 per mil on the largest lattices
RBC/UKQCD 10A	[144]	$2+1$	2.7	${\gtrsim } 4.0$	
Blum 10	[103]	$2+1$	1.8, 2.7	–	Simulations done with quenched photons; large finite-volume effects analytically corrected for, but not related to $M_\pi L$
PACS-CS 09	[94]	$2+1$	2.9	2.0	Only one volume
HPQCD 09A, 10	[9, 18]	$2+1$			cf. MILC 09
MILC 09A, 09	[6, 89]	$2+1$	2.5, 2.9, 3.4, 3.6, 3.8, 5.8	4.1, 3.8	
PACS-CS 08	[93]	$2+1$	2.9	2.3	Correction for FV from $\chi$PT using [82]
RBC/UKQCD 08	[145]	$2+1$	1.8, 2.7	4.6	Various volumes for comparison and correction for FV from $\chi$PT [82, 257, 258]
CP-PACS/JLQCD 07	[146]	$2+1$	2.0	6.0	Estimate based on the comparison to a $L=1.6$ fm volume assuming powerlike dependence on *L*
HPQCD 05	[147]	$2+1$	2.4, 2.9	3.5	
HPQCD/MILC/UKQCD 04, MILC 04	[107, 148]	$2+1$	2.4, 2.9	3.5	NLO S$\chi$PT

**Table 66:** Finite-volume effects in determinations of $m_{ud}$, $m_s$ and, in some cases $m_u$ and $m_d$, with $N_f=2$ quark flavours

Collaboration	Refs.	$N_{ f}$	*L* [fm]	$M_{\pi ,{\mathrm{min}}} L$	Description
ETM 14D	[160]	2	2.2, 4.5	3.2	
RM123 13	[16]	2	${\gtrsim } 2.0$	3.5	One volume $L=1.7$ fm at $m_\pi =495, \,a=0.054$ fm
ALPHA 12	[12]	2	2.1–3.2	4.2	Roughly 2 distinct volumes; no analysis of FV effects
RM123 11	[167]	2	${\gtrsim } 2.0$	3.5	One volume $L=1.7$ fm at $m_\pi =495, \,a=0.054$ fm
Dürr 11	[132]	2	1.22–2.30	2.8	A number of volumes in determination of $m_c/m_s$, but all but one have $L<2~\mathrm{fm}$
ETM 10B	[11]	2	${\gtrsim } 2.0$	3.5	One volume $L=1.7$ fm at $m_\pi =495, \,a=0.054$ fm
JLQCD/TWQCD 08A	[138]	2	1.9	2.8	Corrections for FV based on NLO $\chi$PT
RBC 07	[105]	2	1.9	4.3	Estimate of FV effect based on a model
ETM 07	[133]	2	2.1	3.2	NLO PQ$\chi$PT
QCDSF/UKQCD 06	[139]	2	1.4–1.9	4.7	
SPQcdR 05	[140]	2	1.0–1.5	4.3	Comparison between 1.0 and 1.5 fm
ALPHA 05	[135]	2	2.6	7.4	
QCDSF/UKQCD 04	[137]	2	1.7–2.0	4.7	
JLQCD 02	[141]	2	1.8	5.1	Numerical study with three volumes
CP-PACS 01	[134]	2	2.0–2.6	5.7	

**Table 67:** Renormalization in determinations of $m_{ud}$, $m_s$ and, in some cases $m_u$ and $m_d$, with $N_f=2+1+1$ quark flavours

Collaboration	Refs.	$N_{ f}$	Description
HPQCD 14A	[5]	$2+1+1$	Renormalization not required through the use of the ratio $m_c/m_s$
FNAL/MILC 14A	[14]	$2+1+1$	Renormalization not required for $m_s/m_{ud}$
ETM 14	[4]	$2+1+1$	Nonperturbative renormalization (RI/MOM)

**Table 68:** Renormalization in determinations of $m_{ud}$, $m_s$ and, in some cases $m_u$ and $m_d$, with $N_f=2+1$ quark flavours

Collaboration	Refs.	$N_{ f}$	Description
QCDSF/UKQCD 15	[166]	$2+1$	Nonperturbative renormalization (RI/MOM)
RBC/UKQCD 14B	[10]	$2+1$	Nonperturbative renormalization (RI/SMOM)
RBC/UKQCD 12	[31]	$2+1$	Nonperturbative renormalization (RI/SMOM)
PACS-CS 12	[143]	$1+1+1$	cf. PACS-CS 10
Laiho 11	[44]	$2+1$	$Z_A$ from AWI and $Z_A/Z_S-1$ from one-loop, tadpole-improved, perturbation theory
PACS-CS 10	[95]	$2+1$	Nonperturbative renormalization and running; Schrödinger functional method
MILC 10A	[13]	$2+1$	cf. MILC 09A, 09
BMW 10A, 10B	[7, 8]	$2+1$	Nonperturbative renormalization (tree-level improved RI-MOM), nonperturbative running
RBC/UKQCD 10A	[144]	$2+1$	Nonperturbative renormalization (RI/SMOM)
Blum 10	[103]	$2+1$	Relies on nonperturbative renormalization factors calculated by RBC/UKQCD 08; no QED renormalization
PACS-CS 09	[94]	$2+1$	Nonperturbative renormalization; Schrödinger functional method
HPQCD 09A, 10	[9, 18]	$2+1$	Lattice calculation of $m_s/m_c$: $m_s$ derived from a perturbative determination of $m_c$
MILC 09A, 09	[6, 89]	$2+1$	Two-loop perturbative renormalization
PACS-CS 08	[93]	$2+1$	One-loop perturbative renormalization
RBC/UKQCD 08	[145]	$2+1$	Nonperturbative renormalization, 3-loop perturbative matching
CP-PACS/JLQCD 07	[146]	$2+1$	One-loop perturbative renormalization, tadpole improved
HPQCD 05	[147]	$2+1$	Two-loop perturbative renormalization
HPQCD/MILC/UKQCD 04, MILC 04	[107, 148]	$2+1$	One-loop perturbative renormalization

**Table 69:** Renormalization in determinations of $m_{ud}$, $m_s$ and, in some cases $m_u$ and $m_d$, with $N_f=2$ quark flavours

Collaboration	Refs.	$N_{ f}$	Description
ETM 14D	[160]	2	Renormalization not required for $m_s/m_{ud}$
RM123 13	[16]	2	Nonperturbative renormalization
ALPHA 12	[12]	2	Nonperturbative renormalization
RM123 11	[167]	2	Nonperturbative renormalization
Dürr 11	[132]	2	Lattice calculation of $m_s/m_c$: $m_s$ derived from a perturbative determination of $m_c$
ETM 10B	[11]	2	Nonperturbative renormalization
JLQCD/TWQCD 08A	[138]	2	Nonperturbative renormalization
RBC 07	[105]	2	Nonperturbative renormalization
ETM 07	[133]	2	Nonperturbative renormalization
QCDSF/UKQCD 06	[139]	2	Nonperturbative renormalization
SPQcdR 05	[140]	2	Nonperturbative renormalization
ALPHA 05	[135]	2	Nonperturbative renormalization
QCDSF/UKQCD 04	[137]	2	Nonperturbative renormalization
JLQCD 02	[141]	2	One-loop perturbative renormalization
CP-PACS 01	[134]	2	One-loop perturbative renormalization

**Table 70:** Continuum extrapolations/estimation of lattice artefacts in the determinations of $m_c$

Collaboration	Refs.	$N_{ f}$	*a* [fm]	Description
HPQCD 14A	[5]	$2+1+1$	0.06, 0.09, 0.12, 0.15	Scale set through the Wilson flow parameter $w_0$
ETM 14	[4]	$2+1+1$	0.062, 0.082, 0.089	Scale set through $F_\pi$
ETM 14A	[176]	$2+1+1$	0.062, 0.082, 0.089	Scale set through the nucleon mass $M_N$
JLQCD 15B	[174]	$2+1$	0.044, 0.055, 0.083	Möbius domain wall fermions
$\chi$QCD 14	[17]	$2+1$	0.087, 0.11	Overlap valence fermions on domain-wall sea quarks from [144]. The lattice scale is set together with the strange- and charm-quark masses using the experimental values of the $D_s$, $D_s^*$ and $J/\psi$ meson masses
HPQCD 10	[9]	$2+1$	0.044, 0.059, 0.085, 0.12, 0.15	Scale set through the static-quark potential parameter $r_1$
HPQCD 08B	[152]	$2+1$	0.06, 0.09, 0.12, 0.15	Scale set through the static-quark potential parameter $r_1$
ALPHA 13B	[177]	2	0.048, 0.065	Scale set through $F_K$
ETM 11F	[175]	2		cf. ETM 10B
ETM 10B	[11]	2	0.054, 0.067, 0.085, 0.098	Scale set through $F_\pi$

**Table 71:** Chiral-extrapolation/minimum pion mass in the determinations of $m_c$

Collaboration	Refs.	$N_{ f}$	$M_{\pi ,{\mathrm{min}}}$ [MeV]	Description
HPQCD 14A	[5]	$2+1+1$	$128_{\pi , 5}$ ($173_{RMS}$)	
ETM 14	[4]	$2+1+1$	$180_{\pi ^0}$ ($220_{\pi ^\pm }$)	
ETM 14A	[176]	$2+1+1$	210	cf. ETM 14
JLQCD 15B	[174]	$2+1$		
$\chi$QCD 14	[17]	$2+1$	290	
HPQCD 10	[9]	$2+1$	260	
HPQCD 08B	[152]	$2+1$		
ALPHA 13B	[177]	2	190	
ETM 11F	[175]	2		cf. ETM 10B
ETM 10B	[11]	2	270	

**Table 72:** Finite-volume effects in the determinations of $m_c$

Collaboration	Refs.	$N_{ f}$	*L* [fm]	$M_{\pi ,{\mathrm{min}}} L$	Description
HPQCD 14A	[5]	$2+1+1$	2.5–5.8	3.7	
ETM 14	[4]	$2+1+1$	2.0–3.0	$2.7_{\pi ^0}$ ($3.3_{\pi ^\pm }$)	
ETM 14A	[176]	$2+1+1$	2.0–3.0	$2.7_{\pi ^0}$ ($3.3_{\pi ^\pm }$)	
JLQCD 15B	[174]	$2+1$	2.7		
$\chi$QCD 14	[17]	$2+1$	2.8	4.1	
HPQCD 10	[9]	$2+1$	2.3–3.4	3.8	
HPQCD 08B	[152]	$2+1$			
ALPHA 13B	[177]	2	4.2	4.0	
ETM 11F	[175]	2			cf. ETM 10B
ETM 10B	[11]	2	${\gtrsim } 2.0$	3.5	

**Table 73:** Renormalization in the determinations of $m_c$

Collaboration	Refs.	$N_{ f}$	Description
HPQCD 14A	[5]	$2+1+1$	Renormalization not required
ETM 14	[4]	$2+1+1$	Nonperturbative renormalization (RI/MOM)
ETM 14A	[176]	$2+1+1$	Nonperturbative renormalization (RI/MOM)
JLQCD 15B	[174]	$2+1$	Renormalization not required
$\chi$QCD 14	[17]	$2+1$	Nonperturbative renormalization (RI/MOM)
HPQCD 10	[9]	$2+1$	Renormalization not required
HPQCD 08B	[152]	$2+1$	Renormalization not required
ALPHA 13B	[177]	2	Nonperturbative renormalization (RI/MOM) plus one-loop PT estimate for the improvement b-coefficients
ETM 11F	[175]	2	Renormalization not required
ETM 10B	[11]	2	Nonperturbative renormalization (RI/MOM)

**Table 74:** Continuum extrapolations/estimation of lattice artefacts in the determinations of $m_b$

Collaboration	Refs.	$N_{ f}$	*a* [fm]	Description
HPQCD 14B	[19]	$2+1+1$	0.09, 0.12, 0.15	Scale set through the $\Upsilon ^\prime -\Upsilon$ mass splitting
ETM 14B	[180]	$2+1+1$	0.062, 0.082, 0.089	Scale set through $F_\pi$
HPQCD 14A	[5]	$2+1+1$	0.06, 0.09, 0.12, 0.15	Scale set through the Wilson flow parameter $w_0$
HPQCD 13B	[181]	$2+1$	0.084, 0.12	Scale set through the static-quark potential parameter $r_1$
HPQCD 10	[9]	$2+1$	0.044, 0.059, 0.084, 0.12, 0.15	Scale set through the static-quark potential parameter $r_1$
ETM 13B	[20]	2	0.054, 0.067, 0.085, 0.098	Scale set through the static-quark potential parameter $r_0$
ALPHA 13C	[21]	2	0.048, 0.065, 0.075	Scale set through $F_K$
ETM 11A	[182]	2	0.054, 0.067, 0.085, 0.098	Scale set through $F_\pi$

**Table 75:** Chiral-extrapolation/minimum pion mass in the determinations of $m_b$

Collaboration	Refs.	$N_{ f}$	$M_{\pi ,{\mathrm{min}}}$ [MeV]
HPQCD 14B	[19]	$2+1+1$	306, 128
ETM 14B	[180]	$2+1+1$	210
HPQCD 14A	[5]	$2+1+1$	$128_{\pi , 5}$ ($173_{RMS}$)
HPQCD 13B	[181]	$2+1$	345
HPQCD 10	[9]	$2+1$	260
ETM 13B	[20]	2	280
ALPHA 13C	[21]	2	190
ETM 11A	[182]	2	280

**Table 76:** Finite-volume effects in the determinations of $m_b$

Collaboration	Refs.	$N_{ f}$	*L* [fm]	$M_{\pi ,{\mathrm{min}}} L$
HPQCD 14B	[19]	$2+1+1$	2.4–7.8	3.0–3.8
ETM 14B	[180]	$2+1+1$	1.9–2.8	3.0–5.8
HPQCD 14A	[5]	$2+1+1$	2.5–5.8	3.7
HPQCD 13B	[181]	$2+1$	2.4, 3.4	4.1
HPQCD 10	[9]	$2+1$	2.3–3.4	3.8
ETM 13B	[20]	2	${\gtrsim } 2.0$	3.5
ALPHA 13C	[21]	2	2.3–3.6	4.1
ETM 11A	[182]	2	${\gtrsim } 2.0$	3.5

### B.2 Notes to Sect. 4 on $|V_{ud}|$ and $|V_{us}|$

See Tables 78, 79, 80, 81, 82, 83, 84, 85, 86, 87 and 88.

**Table 77:** Lattice renormalization in the determinations of $m_b$

Collaboration	Refs.	$N_{ f}$	Description
HPQCD 14B	[19]	$2+1+1$	Renormalization not required
ETM 14B	[180]	$2+1+1$	Nonperturbative renormalization (RI/MOM)
HPQCD 14A	[5]	$2+1+1$	Renormalization not required
HPQCD 13B	[181]	$2+1$	Renormalization not required
HPQCD 10	[9]	$2+1$	Renormalization not required
ETM 13B	[20]	2	Nonperturbative renormalization (RI/MOM)
ALPHA 13C	[21]	2	Nonperturbatively matched and renormalized HQET
ETM 11A	[182]	2	Renormalization not required

**Table 78:** Continuum extrapolations/estimation of lattice artefacts in the determinations of $f_+(0)$

Collaboration	Refs.	$N_{ f}$	*a* [fm]	Description
ETM 15C	[208]	$2+1+1$	0.062, 0.082, 0.089	Scale set through $f_\pi$. Automatic $\mathcal {O}(a)$ improvement
FNAL/MILC 13E	[22]	$2+1+1$	0.06, 0.09, 0.12, 0.15	HISQ action for both sea and valence quarks. Relative scale through $r_1$, physical scale from pseudoscalar decay constants calculated with Asqtad fermions. The ensemble with $a \simeq 0.06$ fm is used only for cross-checking discretization effects
FNAL/MILC 13C	[209]	$2+1+1$	0.09, 0.12, 0.15	Relative scale through $r_1$, physical scale from $f_\pi$ calculated by MILC 09A at $N_f = 2 + 1$
RBC/UKQCD 15A	[24]	$2+1$	0.08, 0.11	Scale set through $\Omega$ mass
FNAL/MILC 12I	[23]	$2+1$	0.09, 0.12	Relative scale $r_1$, physical scale determined from a mixture of $f_\pi$, $f_K$, radial excitation of $\Upsilon$ and $m_{D_s}-\frac{1}{2} m_{ {\eta }_c}$
RBC/UKQCD 13	[210]	$2+1$	0.09, 0.11, 0.14	Scale set through $\Omega$ mass
JLQCD 12	[211]	$2+1$	0.112	Scale set through $\Omega$ mass
JLQCD 11	[212]	$2+1$	0.112	Scale set through $\Omega$ mass
RBC/UKQCD 07,10	[213, 214]	$2+1$	0.114(2)	Scale fixed through $\Omega$ baryon mass. Add $(\Lambda _\mathrm{QCD}a)^2\approx 4$% systematic error for lattice artefacts. Fifth dimension with extension $L_s=16$, therefore small residual chiral symmetry breaking and approximate $\mathcal {O}(a)$-improvement
ETM 10D	[215]	2	0.05, 0.07, 0.09, 0.10	Scale set through $f_\pi$. Automatic $\mathcal {O}(a)$ impr., flavour symmetry breaking: $(M_{PS}^{0})^2 - (M_{PS}^\pm )^2\sim \mathcal {O}(a^2)$
ETM 09A	[25]	2	0.07, 0.09, 0.10	Scale set through $f_\pi$. Automatic $\mathcal {O}(a)$ impr., flavour symmetry breaking: $(M_{PS}^{0})^2 - (M_{PS}^\pm )^2\sim \mathcal {O}(a^2)$. Three lattice spacings only for pion mass 470 MeV
QCDSF 07	[216]	2	0.075	Scale set with $r_0$. Nonperturbatively $\mathcal {O}(a)$-improved Wilson fermions, not clear whether currents improved
RBC 06	[217]	2	0.12	Scale set through $M_\rho$. Automatic $\mathcal {O}(a)$-improvement due to approximate chiral symmetry of the action
JLQCD 05	[218]	2	0.0887	Scale set through $M_\rho$. Nonperturbatively $\mathcal {O}(a)$-improved Wilson fermions

**Table 79:** Chiral-extrapolation/minimum pion mass in determinations of $f_+(0)$. The subscripts RMS and $\pi ,5$ in the case of staggered fermions indicate the root-mean-square mass and the Nambu–Goldstone boson mass, respectively. In the case of twisted-mass fermions $\pi ^0$ and $\pi ^\pm$ indicate the neutral and charged pion mass where applicable

Collaboration	Refs.	$N_{ f}$	$M_{\pi ,{\mathrm{min}}}$ [MeV]	Description
ETM 15C	[208]	$2+1+1$	$180_\mathrm{\pi ^0}(220_{\pi ^\pm })$	Chiral extrapolation performed through *SU*(2) or *SU*(3) $\chi$PT
FNAL/MILC 13E	[22]	$2+1+1$	$173_\mathrm{RMS}(128_\mathrm{\pi ,5})$	NLO *SU*(3) PQ staggered $\chi$PT with continuum $\chi$PT at NNLO. Lightest Nambu–Goldstone mass is 128 MeV and lightest RMS mass is 173 MeV for the same gauge ensemble with $a \simeq 0.09$ fm
FNAL/MILC 13C	[209]	$2+1+1$	$173_\mathrm{RMS}(128_\mathrm{\pi ,5})$	NLO *SU*(3) PQ staggered $\chi$PT with continuum $\chi$PT at NNLO. Lightest Nambu–Goldstone mass is 128 MeV and lightest RMS mass is 173 MeV for the same gauge ensemble with $a \simeq 0.09$ fm
RBC/UKQCD 15A	[24]	$2+1$	140	NLO *SU*(3) $\chi$PT with phenomenological ansatz for higher orders or polynomial models
FNAL/MILC 12I	[23]	$2+1$	$378_\mathrm{RMS}(263_\mathrm{\pi ,5})$	NLO *SU*(3) PQ staggered $\chi$PT with either phenomenological NNLO ansatz or NNLO $\chi$PT. Lightest Nambu–Goldstone mass is 263 MeV with $a=0.12$ fm and lightest RMS mass is 378 MeV with $a=0.09$ fm
RBC/UKQCD 13	[210]	$2+1$	170	NLO *SU*(3) $\chi$PT with phenomenological ansatz for higher orders
JLQCD 12	[211]	$2+1$	290	NLO *SU*(3) $\chi$PT with phenomenological ansatz for higher orders
JLQCD 11	[212]	$2+1$	290	NLO *SU*(3) $\chi$PT with phenomenological ansatz for higher orders
RBC/UKQCD 07,10	[213, 214]	$2+1$	330	NLO *SU*(3) $\chi$PT with phenomenological ansatz for higher orders
ETM 10D	[215]	2	$210_{\pi ^0}(260_{\pi ^\pm })$	NLO heavy kaon *SU*(2) $\chi$PT and NLO *SU*(3) $\chi$PT and phenomenological ansatz for higher orders. Average of $f_+(0)$-fit and joint $f_+(0)$-$f_K/f_\pi$-fit
ETM 09A	[25]	2	$210_{\pi ^0}(260_{\pi ^\pm })$	NLO heavy kaon *SU*(2) $\chi$PT and NLO *SU*(3) $\chi$PT and phenomenological ansatz for higher orders
QCDSF 07	[216]	2	591	Only one value for the pion mass
RBC 06	[217]	2	490	NLO *SU*(3) $\chi$PT and phenomenological ansatz for higher orders
JLQCD 05	[218]	2	550	NLO *SU*(3) $\chi$PT and phenomenological ansatz for higher orders

**Table 80:** Finite-volume effects in determinations of $f_+(0)$. The subscripts RMS and $\pi ,5$ in the case of staggered fermions indicate the root-mean-square mass and the Nambu–Goldstone boson mass, respectively. In the case of twisted-mass fermions $\pi ^0$ and $\pi ^\pm$ indicate the neutral and charged pion mass where applicable

Collaboration	Refs.	$N_{ f}$	*L* [fm]	$M_{\pi ,{\mathrm{min}}}L$	Description
ETM 15C	[208]	$2+1+1$	2.0–3.0	$2.7_{\pi ^0}(3.3_{\pi ^\pm })$	FSE observed only in the slopes of the vector and scalar form factors
FNAL/MILC 13E	[22]	$2+1+1$	2.9–5.8	$4.9_\mathrm{RMS}(3.6_{\pi ,5})$	The values correspond to $M_{\pi ,\mathrm RMS}=173$ MeV and $M_{\pi ,5}=128$ MeV, respectively
FNAL/MILC 13C	[209]	$2+1+1$	2.9–5.8	$4.9_\mathrm{RMS}(3.6_{\pi ,5})$	The values correspond to $M_{\pi ,\mathrm RMS}=173$ MeV and $M_{\pi ,5}=128$ MeV, respectively
RBC/UKQCD 15A	[24]	$2+1$	2.6, 5.2	3.9	
FNAL/MILC 12I	[23]	$2+1$	2.4–3.4	$6.2_\mathrm{RMS}(3.8_{\pi ,5})$	The values correspond to $M_{\pi ,\mathrm RMS}=378$ MeV and $M_{\pi ,5}=263$ MeV, respectively
RBC/UKQCD 13	[210]	$2+1$	2.7, 4.6	3.9	
JLQCD 12	[211]	$2+1$	1.8, 2.7	4.1	
JLQCD 11	[212]	$2+1$	1.8, 2.7	4.1	
RBC/UKQCD 07,10	[213, 214]	$2+1$	1.8, 2.7	4.7	Two volumes for all but the lightest pion mass
ETM 10D	[215]	2	2.1–2.8	$3.0_{\pi ^0}(3.7_{\pi ^\pm })$	
ETM 09A	[25]	2	2.1, 2.8	$3.0_{\pi ^0}(3.7_{\pi ^\pm })$	Two volumes at $M_\pi =300$ MeV and $\chi$PT-motivated estimate of the error due to FSE
QCDSF 07	[216]	2	1.9	5.4	
RBC 06	[217]	2	1.9	4.7	
JLQCD 05	[218]	2	1.8	4.9	

**Table 81:** Continuum extrapolations/estimation of lattice artefacts in determinations of $f_K/f_\pi$ for $N_f=2+1+1$ simulations

Collaboration	Refs.	$N_{ f}$	*a* [fm]	Description
ETM 14E	[27]	$2+1+1$	0.062, 0.082, 0.089	Scale set through $f_\pi$. Automatic $\mathcal {O}(a)$ improvement, flavour symmetry breaking: $(M_{PS}^{0})^2-(M_{PS}^\pm )^2\sim \mathcal {O}(a^2)$. Discretization and volume effects due to the $\pi ^0$–$\pi ^\pm$ mass splitting are taken into account through $\chi$PT for twisted-mass fermions
FNAL/MILC 14A	[14]	$2+1+1$	0.06, 0.09, 0.12, 0.15	HISQ action for both valence and sea quarks. Absolute scale though $f_\pi$
HPQCD 13A	[26]	$2+1+1$	0.09, 0.12, 0.15	Relative scale through Wilson flow and absolute scale through $f_\pi$
MILC 13A	[231]	$2+1+1$	0.06, 0.09, 0.12, 0.15	Absolute scale though $f_\pi$
ETM 13F	[230]	$2+1+1$	0.062, 0.082, 0.089	Scale set through $f_\pi$. Automatic $\mathcal {O}(a)$ improvement, flavour symmetry breaking: $(M_{PS}^{0})^2-(M_{PS}^\pm )^2\sim \mathcal {O}(a^2)$. Discretization and volume effects due to the $\pi ^0$–$\pi ^\pm$ mass splitting are taken into account through $\chi$PT for twisted-mass fermions
ETM 10E	[233]	$2+1+1$	0.061, 0.078	Scale set through $f_\pi /m_\pi$. Two lattice spacings but *a*-dependence ignored in all fits. Finer lattice spacing from [352]
MILC 11	[232]	$2+1+1$	0.09, 0.12	Relative scale through $f_\mathrm{PS}/m_\mathrm{PS}=\hbox {fixed}$, absolute scale though $f_\pi$

**Table 82:** Continuum extrapolations/estimation of lattice artefacts in determinations of $f_K/f_\pi$ for $N_f=2+1$ simulations

Collaboration	Refs.	$N_{ f}$	*a* [fm]	Description
RBC/UKQCD 14B	[10]	$2+1$	0.063, 0.085, 0.114	Scale set through $m_\Omega$
RBC/UKQCD 12	[31]	$2+1$	0.09, 0.11, 0.14	Scale set through $m_\Omega$
Laiho 11	[44]	$2+1$	0.06, 0.09, 0.125	Scale set through $r_1$ and $\Upsilon$ and continuum extrapolation based on MA$\chi$PT
JLQCD/TWQCD 10	[234]	$2+1$	0.112	Scale set through $M_\Omega$
RBC/UKQCD 10A	[144]	$2+1$	0.087, 0.114	Scale set through $M_\Omega$
MILC 10	[29]	$2+1$	0.045, 0.06, 0.09	3 lattice spacings, continuum extrapolation by means of RS$\chi$PT
BMW 10	[30]	$2+1$	0.07, 0.08, 0.12	Scale set through $M_{\Omega ,\Xi }$. Perturbative $\mathcal {O}(a)$-improvement
JLQCD/TWQCD 09A	[138]	$2+1$	0.1184(3)(21)	Scale set through $F_\pi$. Automatic $\mathcal {O}(a)$-improvement due to chiral symmetry of action
PACS-CS 09	[94]	$2+1$	0.0900(4)	Scale set through $M_\Omega$
MILC 09A	[6]	$2+1$	0.045, 0.06, 0.09	Scale set through $r_1$ and $\Upsilon$ and continuum extrapolation based on RS$\chi$PT
MILC 09	[89]	$2+1$	0.045, 0.06, 0.09, 0.12	Scale set through $r_1$ and $\Upsilon$ and continuum extrapolation based on RS$\chi$PT
Aubin 08	[236]	$2+1$	0.09, 0.12	Scale set through $r_1$ and $\Upsilon$ and continuum extrapolation based on MA$\chi$PT
PACS-CS 08, 08A	[93, 237]	$2+1$	0.0907(13)	Scale set through $M_\Omega$. Nonperturbatively $\mathcal {O}(a)$-improved
HPQCD/UKQCD 07	[28]	$2+1$	0.09, 0.12, 0.15	Scale set through $r_1$ and $\Upsilon$ and continuum extrapolation on continuum-$\chi$PT motivated ansatz. Taste breaking of sea quarks ignored
RBC/UKQCD 08	[145]	$2+1$	0.114(2)	Scale set through $M_\Omega$. Automatic $\mathcal {O}(a)$-improvement due to approximate chiral symmetry. $(\Lambda _\mathrm{QCD}a)^2\approx 4\%$ systematic error due to lattice artefacts added
NPLQCD 06	[238]	$2+1$	0.125	Scale set through $r_0$ and $F_\pi$. Taste breaking of sea quarks ignored
MILC 04	[107]	$2+1$	0.09, 0.12	Scale set through $r_1$ and $\Upsilon$ and continuum extrapolation based on RS$\chi$PT

**Table 83:** Continuum extrapolations/estimation of lattice artefacts in determinations of $f_K/f_\pi$ for $N_f=2$ simulations

Collaboration	Refs.	$N_{ f}$	*a* [fm]	Description
ETM 14D	[160]	2	0.094	Scale set through $F_\pi$, $r_0$, $t_0$ and $w_0$. Twisted Wilson fermions plus clover term. Automatic $\mathcal {O}(a)$ improvement
ALPHA 13A	[239]	2	0.05, 0.065, 0.075	Scale set through $F_\pi$. $\mathcal {O}(a)$-improved Wilson action
BGR 11	[240]	2	0.135	Scale set through $r_0 = 0.48$ fm. Chirally improved Dirac operator
ETM 10D	[215]	2	0.05, 0.07, 0.09, 0.10	Scale set through $F_\pi$. Automatic $\mathcal {O}(a)$ impr., flavour symmetry breaking: $(M_{PS}^{0})^2-(M_{PS}^\pm )^2\sim \mathcal {O}(a^2)$
ETM 09	[32]	2	0.07, 0.09, 0.10	Scale set through $F_\pi$. Automatic $\mathcal {O}(a)$ impr., flavour symmetry breaking: $(M_{PS}^{0})^2-(M_{PS}^\pm )^2\sim \mathcal {O}(a^2)$
QCDSF/UKQCD 07	[241]	2	0.06, 0.07	Scale set through $F_\pi$. Nonperturbative $\mathcal {O}(a)$-improvement

**Table 84:** Chiral-extrapolation/minimum pion mass in determinations of $f_K / f_\pi$ for $N_f=2+1+1$ simulations. The subscripts RMS and $\pi ,5$ in the case of staggered fermions indicate the root-mean-square mass and the Nambu–Goldstone boson mass. In the case of twisted-mass fermions $\pi ^0$ and $\pi ^\pm$ indicate the neutral and charged pion mass and, where applicable, “val” and “sea” indicate valence- and sea-pion masses

Collaboration	Refs.	$N_{ f}$	$M_{\pi ,{\mathrm{min}}}$ [MeV]	Description
ETM 14E	[27]	$2+1+1$	$180_\mathrm{\pi ^0}(220_{\pi ^\pm })$	Chiral extrapolation performed through *SU*(2) $\chi$PT or polynomial fit
FNAL/MILC 14A	[14]	$2+1+1$	$143_\mathrm{RMS}(128_{\pi ,5})$	Linear interpolation to physical point. The lightest RMS mass is from the $a=0.06$ fm ensemble and the lightest Nambu–Goldstone mass is from the $a=0.09$ fm ensemble
HPQCD 13A	[26]	$2+1+1$	$173_\mathrm{RMS}(128_{\pi ,5})$	NLO $\chi$PT supplemented by model for NNLO. Both the lightest RMS and the lightest Nambu–Goldstone mass are from the $a=0.09$ fm ensemble
MILC 13A	[231]	$2+1+1$	$143_\mathrm{RMS}(128_{\pi ,5})$	Linear interpolation to physical point. The lightest RMS mass is from the $a=0.06$ fm ensemble and the lightest Nambu–Goldstone mass is from the $a=0.09$ fm ensemble
ETM 13F	[230]	$2+1+1$	$180_\mathrm{\pi ^0}(220_{\pi ^\pm })$	Chiral extrapolation performed through *SU*(2) $\chi$PT or polynomial fit
ETM 10E	[233]	$2+1+1$	$215_\mathrm{\pi ^0}(265_{\pi ^\pm })$	
MILC 11	[232]	$2+1+1$	$173_\mathrm{RMS}(128_{\pi ,5})$	Quoted result from polynomial interpolation to the physical point. The lightest RMS mass is from the $a=0.06$ fm ensemble and lightest the Nambu–Goldstone mass is from the $a=0.09$ fm ensemble

**Table 85:** Chiral-extrapolation/minimum pion mass in determinations of $f_K/f_\pi$ for $N_f=2+1$ simulations. The subscripts RMS and $\pi ,5$ in the case of staggered fermions indicate the root-mean-square mass and the Nambu–Goldstone boson mass. In the case of twisted-mass fermions $\pi ^0$ and $\pi ^\pm$ indicate the neutral and charged pion mass and where applicable, “val” and “sea” indicate valence- and sea-pion masses

Collaboration	Refs.	$N_{ f}$	$M_{\pi ,{\mathrm{min}}}$ [MeV]	Description
RBC/UKQCD 14B	[10]	$2+1$	139	NLO PQ *SU*(2) $\chi$PT as well as analytic ansätze
RBC/UKQCD 12	[31]	$2+1$	171$_\mathrm{sea}$, 143$_\mathrm{val}$	NLO PQ *SU*(2) $\chi$PT as well as analytic ansätze
Laiho 11	[44]	$2+1$	250$_\mathrm{RMS}(220_{\pi ,5})$	NLO MA$\chi$PT
JLQCD/TWQCD 10	[234]	$2+1$	290	NNLO $\chi$PT
RBC/UKQCD 10A	[144]	$2+1$	290	Results are based on heavy kaon NLO *SU*(2) PQ$\chi$PT
MILC 10	[29]	$2+1$	$258_\mathrm{RMS}(177_{\pi ,5})$	Lightest Nambu–Goldstone mass is 177 MeV (at 0.09 fm) and lightest RMS mass is 258 MeV (at 0.06 fm). NLO rS$\chi$PT and NNLO $\chi$PT
BMW 10	[30]	$2+1$	190	Comparison of various fit-ansätze: *SU*(3) $\chi$PT, heavy kaon *SU*(2) $\chi$PT, polynomial
JLQCD/TWQCD 09A	[138]	$2+1$	290	NNLO *SU*(3) $\chi$PT
PACS-CS 09	[94]	$2+1$	156	NNLO $\chi$PT
MILC 09A	[6]	$2+1$	$258_\mathrm{RMS}(177_{\pi ,5})$	NLO *SU*(3) RS$\chi$PT, continuum $\chi$PT at NNLO and up to NNNNLO analytic terms. Heavy kaon *SU*(2) RS$\chi$PT with NNLO continuum chiral logs on a sub-set of the lattices. The lightest Nambu–Goldstone mass is 177 MeV (at $a=0.09$ fm) and the lightest RMS mass is 258 MeV (at $a=0.06$ fm)
MILC 09	[89]	$2+1$	$258_\mathrm{RMS}(224_{\pi ,5})$	NLO *SU*(3) RS$\chi$PT with continuum $\chi$PT NNLO and NNNLO analytic terms added. According to [6] the lightest sea Nambu–Goldstone mass is 224 MeV and the lightest RMS mass is 258 MeV (at $a=0.06$ fm)
Aubin 08	[236]	$2+1$	$329_\mathrm{RMS}(246_{\pi ,5})$	NLO MA$\chi$PT. According to [6] the lightest sea Nambu–Goldstone mass is 246 MeV (at $a=0.09$ fm) and the lightest RMS mass is 329 MeV (at $a=0.09$ fm)
PACS-CS 08, 08A	[93, 237]	$2+1$	156	NLO *SU*(2) $\chi$PT and *SU*(3) (Wilson)$\chi$PT
HPQCD/UKQCD 07	[28]	$2+1$	$375_\mathrm{RMS}(263_{\pi ,5})$	NLO *SU*(3) chiral perturbation theory with NNLO and NNNLO analytic terms. The lightest RMS mass is from the $a=0.09$ fm ensemble and the lightest Nambu–Goldstone mass is from the $a=0.12$ fm ensemble
RBC/UKQCD 08	[145]	$2+1$	$330_\mathrm{sea}$, $242_\mathrm{val}$	While *SU*(3) PQ$\chi$PT fits were studied, final results are based on heavy kaon NLO *SU*(2) PQ$\chi$PT
NPLQCD 06	[238]	$2+1$	300	NLO *SU*(3) $\chi$PT and some NNLO terms. The sea RMS mass for the employed lattices is heavier
MILC 04	[107]	$2+1$	$400_\mathrm{RMS}(260_{\pi ,5})$	PQ RS$\chi$PT fits. The lightest sea Nambu–Goldstone mass is 260 MeV (at $a=0.12$ fm) and the lightest RMS mass is 400 MeV (at $a=0.09$ fm)

**Table 86:** Chiral-extrapolation/minimum pion mass in determinations of $f_K/f_\pi$ for $N_f=2$ simulations. The subscripts RMS and $\pi ,5$ in the case of staggered fermions indicate the root-mean-square mass and the Nambu–Goldstone boson mass. In the case of twisted-mass fermions $\pi ^0$ and $\pi ^\pm$ indicate the neutral and charged pion mass and where applicable, “val” and “sea” indicate valence- and sea-pion masses

Collaboration	Refs.	$N_{ f}$	$M_{\pi ,{\mathrm{min}}}$ [MeV]	Description
ETM 14D	[160]	2	140	Charged/neutral pion-mass breaking, $M_{\pi ^\pm }^2-M_{\pi ^0}^2 \sim \mathcal {O}(a^2)$, estimated to be ${\simeq }20$ MeV
ALPHA 13A	[239]	2	190	NLO *SU*(3) $\chi$PT and phenomenological ansatz for higher orders
BGR 11	[240]	2	250	NLO *SU*(2) $\chi$PT. Strange quark mass fixed by reproducing the $\Omega$ mass
ETM 10D	[215]	2	$210_{\pi ^0}(260_{\pi ^\pm })$	NLO *SU*(3) $\chi$PT and phenomenological ansatz for higher orders. Joint $f_+(0)$-$f_K/f_\pi$-fit
ETM 09	[32]	2	$210_{\pi ^0}(260_{pi^\pm })$	NLO heavy meson *SU*(2) $\chi$PT and NLO *SU*(3) $\chi$PT
QCDSF/UKQCD 07	[241]	2	300	Linear extrapolation of lattice data

**Table 87:** Finite-volume effects in determinations of $f_K/f_\pi$ for $N_f=2+1+1$. The subscripts RMS and $\pi ,5$ in the case of staggered fermions indicate the root-mean-square mass and the Nambu–Goldstone boson mass. In the case of twisted-mass fermions $\pi ^0$ and $\pi ^\pm$ indicate the neutral and charged pion mass and where applicable, “val” and “sea” indicate valence- and sea-pion masses

Collaboration	Refs.	$N_{ f}$	*L* [fm]	$M_{\pi ,{\mathrm{min}}}L$	Description
ETM 14E	[27]	$2+1+1$	2.0–3.0	$2.7_{\pi ^0}(3.3_{\pi ^\pm })$	FSE for the pion is corrected through resummed NNLO $\chi$PT for twisted-mass fermions, which takes into account the effects due to the $\pi ^0$–$\pi ^\pm$ mass splitting
FNAL/MILC 14A	[14]	$2+1+1$	2.8–5.8	$3.9_\mathrm{RMS}(3.7_{\pi ,5})$	
HPQCD 13A	[26]	$2+1+1$	2.5–5.8	$4.9_\mathrm{RMS}(3.7_{\pi ,5})$	
MILC 13A	[231]	$2+1+1$	2.8–5.8	$3.9_\mathrm{RMS}(3.7_{\pi ,5})$	
ETM 13F	[230]	$2+1+1$	2.0–3.0	$2.7_{\pi ^0}(3.3_{\pi ^\pm })$	FSE for the pion is corrected through resummed NNLO $\chi$PT for twisted-mass fermions, which takes into account the effects due to the $\pi ^0$–$\pi ^\pm$ mass splitting
ETM 10E	[233]	$2+1+1$	1.9–2.9	$3.1_{\pi ^0}(3.9_{\pi ^\pm })$	Simulation parameters from [352, 789]
MILC 11	[232]	$2+1+1$	5.6, 5.7	$4.9_\mathrm{RMS}(3.7_{\pi ,5})$	

**Table 88:** Finite-volume effects in determinations of $f_K/f_\pi$ for $N_f=2+1$ and $N_f=2$. The subscripts RMS and $\pi ,5$ in the case of staggered fermions indicate the root-mean-square mass and the Nambu–Goldstone boson mass. In the case of twisted-mass fermions $\pi ^0$ and $\pi ^\pm$ indicate the neutral and charged pion mass and where applicable, “val” and “sea” indicate valence- and sea-pion masses

Collaboration	Refs.	$N_{ f}$	*L* [fm]	$M_{\pi ,{\mathrm{min}}}L$	Description
RBC/UKQCD 14B	[10]	$2+1$	2.0, 2.7, 4.6, 5.4	3.8	
RBC/UKQCD 12	[31]	$2+1$	2.7, 4.6	3.3	For partially quenched $M_\pi =143$ MeV, $M_\pi L=3.3$ and for unitary $M_\pi =171$ MeV, $M_\pi L=4.0$
Laiho 11	[44]	$2+1$	2.5–4.0	$4.9_\mathrm{RMS}(4.3_{\pi ,5})$	
JLQCD/TWQCD 10	[234]	$2+1$	1.8, 2.7	4.0	
RBC/UKQCD 10A	[144]	$2+1$	2.7	4.0	$M_\pi L=4.0$ for lightest sea-quark mass and $M_\pi L=3.1$ for lightest partially quenched quark mass
MILC 10	[29]	$2+1$	2.5–3.8	$7.0_\mathrm{RMS}(4.0_{\pi ,5})$	$L\ge 2.9~\mathrm{fm}$ for the lighter masses
BMW 10	[30]	$2+1$	2.0–5.3	4.0	Various volumes for comparison and correction for FSE from $\chi$PT using [82]
JLQCD/TWQCD 09A	[138]	$2+1$	1.9	2.8	Estimate of FSE using $\chi$PT [82, 790]
PACS-CS 09	[94]	$2+1$	2.9	2.28	After reweighting to the physical point $M_{\pi ,\mathrm{min}}L=1.97$
MILC 09A	[6]	$2+1$	2.5–5.8	$7.0_\mathrm{RMS}(4.1_{\pi ,5})$	
MILC 09	[89]	$2+1$	2.4–5.8	$7.0_\mathrm{RMS}(4.8_{\pi ,5})$	Various volumes for comparison and correction for FSEs from (RS)$\chi$PT [82]
Aubin 08	[236]	$2+1$	2.4–3.6	4.0	Correction for FSE from MA$\chi$PT
PACS-CS 08, 08A	[93, 237]	$2+1$	2.9	2.3	Correction for FSE from $\chi$PT using [82]
HPQCD/UKQCD 07	[28]	$2+1$	2.4–2.9	$4.1_\mathrm{RMS}(3.8_{\pi ,5})$	Correction for FSE from $\chi$PT using [82]
RBC/UKQCD 08	[145]	$2+1$	1.8, 2.7	$4.6_\mathrm{sea}$, $3.4_{rval}$	Various volumes for comparison and correction for FSE from $\chi$PT [82, 257, 258]
NPLQCD 06	[238]	$2+1$	2.5	3.8	Correction for FSE from S$\chi$PT [329, 330]
MILC 04	[107]	$2+1$	2.4, 3.0	$4.8_\mathrm{RMS}(3.8_{\pi ,5})$	NLO S$\chi$PT
ETM 14D	[160]	2	2.2, 4.5	3.2	
ALPHA 13A	[239]	2	2.1, 2.4, 3.1	4.0	
BGR 11	[240]	2	2.1, 2.2	2.7	
ETM 10D	[215]	2	2.1–2.8	$3.0_{\pi ^0}(3.7_{\pi ^\pm })$	
ETM 09	[32]	2	2.0–2.7	$3.0_{\pi ^0}(3.7_{\pi ^\pm })$	Correction for FSE from $\chi$PT [82, 257, 258]
QCDSF/UKQCD 07	[241]	2	$1.4,\ldots ,2.6$	4.2	Correction for FSE from $\chi$PT

### B.3 Notes to Sect. 5 on low-energy constants

See Tables 89, 90, 91, 92, 93, 94, 95 and 96.

**Table 89:** Continuum extrapolations/estimation of lattice artefacts in $N_{ f}=2+1+1$ and $2+1$ determinations of the Low-Energy Constants

Collaboration	Refs.	$N_{ f}$	*a* [fm]	Description
HPQCD 13A, 15B	[26, 336]	$2+1+1$	0.09–0.15	Configurations are shared with MILC
ETM 11, 13	[33, 352]	$2+1+1$	0.0607–0.0863	Three lattice spacings fixed through $F_\pi /M_\pi$
ETM 10	[39]	$2+1+1$	0.078, 0.086	Fixed through $F_\pi /M_\pi$
JLQCD 15A	[359]	$2+1$	0.112	Fixed through $\Omega$ baryon mass
RBC/UKQCD 14B, 15E	[10, 335]	$2+1$	$a^{-1}=1.730{-}3.148$	Fixed through $m_\pi$, $m_K$, and $m_{\Omega }$
Boyle 14	[372]	$2+1$	$a^{-1}=1.37, 2.31$	Shared with RBC/UKQCD 12
BMW 13	[35]	$2+1$	0.054–0.093	Scale set through $\Omega$ baryon mass
RBC/UKQCD 12	[31]	$2+1$	0.086, 0.114 and 0.144 for $M_\pi ^{m_{{\mathrm{R}}}{min}}$	Scale set through $m_\Omega$
Borsanyi 12	[34]	$2+1$	0.097–0.284	Scale fixed through $F_\pi /M_\pi$
NPLQCD 11	[40]	$2+1$	0.09, 0.125	Configurations are shared with MILC 09 [89]
MILC 09, 09A, 10, 10A	[6, 13, 29, 89]	$2+1$	0.045–0.18	Three lattice spacings, continuum extrapolation by means of RS$\chi$PT
JLQCD(/TWQCD) 08B, 09, 10A, 14	[337, 338, 341, 360]	$2+1$, 3	0.11	One lattice spacing, fixed through $m_\Omega$
RBC/UKQCD 09, 10A	[144, 373]	$2+1$	0.1106(27), 0.0888(12)	Two lattice spacings. Data combined in global chiral-continuum fits
TWQCD 08	[340]	$2+1$	0.122(3)	Scale fixed through $m_\rho$, $r_0$
PACS-CS 08, 11A	[93, 361]	$2+1$	0.0907	One lattice spacing
RBC/UKQCD 08A, 08	[145, 339]	$2+1$	0.114	One lattice spacing, attempt to estimate cutoff effects via formal argument
NPLQCD 06	[238]	$2+1$	0.125	One lattice spacing, continuum $\chi$PT used
LHP 04	[362]	$2+1$	${\simeq } 0.12$	Only one lattice spacing, mixed discretization approach

**Table 90:** Continuum extrapolations/estimation of lattice artefacts in $N_{ f}=2$ determinations of the low-energy constants

Collaboration	Refs.	$N_{ f}$	*a* [fm]	Description
ETMC 15A	[333]	2	0.0914(3)(15)	Weighted average using $m_\pi$, $f_\pi$, $f_K$, $m_N$
Gülpers 15	[355]	2	0.050, 0.063, 0.079	Scale fixed through $m_{\Omega }$
Engel 14	[38]	2	0.0483(4), 0.0652(6), 0.0749(8)	Scale fixed through $F_K$
Gülpers 13	[356]	2	0.063	Scale fixed through $m_{\Omega }$
Brandt 13	[37]	2	0.05–0.08	Configurations are shared with CLS
QCDSF 13	[353]	2	0.06–0.076	Scale fixed through $r_0 = 0.50(1)$ fm
Bernardoni 11	[343]	2	0.0649(10)	Configurations are shared with CLS
TWQCD 11A, 11	[249, 344]	2	0.1034(1)(2)	Scale fixed through $r_0$
Bernardoni 10	[345]	2	0.0784(10)	Scale fixed through $M_K$. Nonperturbative $\mathcal {O}(a)$ improvement. No estimate of systematic error
ETM 09B	[346]	2	0.063, 0.073	Automatic $\mathcal {O}(a)$ impr. $r_0=0.49$ fm used
ETM 09C, 12, 13	[33, 36, 342]	2	0.051–0.1	Automatic $\mathcal {O}(a)$ impr. Scale fixed through $F_\pi$. four lattice spacings, continuum extrapolation
ETM 08	[41]	2	0.07–0.09	Automatic $\mathcal {O}(a)$ impr. Two lattice spacings. Scale fixed through $F_\pi$
JLQCD/TWQCD 07, 07A, 08A, 09, 10A JLQCD 08A	[138, 338, 348, 349, 357, 374]	2	0.1184(3)(21)	Automatic $\mathcal {O}(a)$ impr., exact chiral symmetry. Scale fixed through $r_0$
CERN 08	[302]	2	0.0784(10)	Scale fixed through $M_K$. Nonperturbative $\mathcal {O}(a)$ improvement
Hasenfratz 08	[347]	2	0.1153(5)	Tree level $\mathcal {O}(a)$ improvement. Scale fixed through $r_0$. Estimate of lattice artefacts via W$\chi$PT [791]
CERN-TOV 06	[358]	2	0.0717(15), 0.0521(7), 0.0784(10)	Scale fixed through $M_K$. The lattice with $a=0.0784(10)$ is obtained with nonperturbative $\mathcal {O}(a)$ improvement
QCDSF/UKQCD 06A	[363]	2	0.07–0.115	Five lattice spacings. Nonperturbative $\mathcal {O}(a)$ improvement. Scale fixed through $r_0$

**Table 91:** Chiral-extrapolation/minimum pion mass in $N_{ f}=2+1+1$ determinations of the low-energy constants

Collaboration	Refs.	$N_{ f}$	$M_{\pi ,{\mathrm{min}}}$ [MeV]	Description
HPQCD 15B	[336]	$2+1+1$	175	Simulated at physical point
HPQCD 13A	[26]	$2+1+1$	175	NLO chiral fit
ETM 13	[33]	$2+1+1$	270	Linear fit in the quark mass
ETM 11	[352]	$2+1+1$	270	NLO *SU*(2) chiral fit
ETM 10	[39]	$2+1+1$	270	*SU*(2) NLO and NNLO fits

**Table 92:** Chiral-extrapolation/minimum pion mass in $2+1$ determinations of the low-energy constants

Collaboration	Refs.	$N_{ f}$	$M_{\pi ,{\mathrm{min}}}$ [MeV]	Description
RBC/UKQCD 15E	[335]	$2+1$	117.3(4.4)	GMOR for $\Sigma$, NNLO PQ *SU*(2) $\chi$PT
JLQCD 15A	[359]	$2+1$	290	Dynamical overlap, NNLO *SU*(3)
RBC/UKQCD 14B	[10]	$2+1$	139.2	GMOR for $\Sigma$, global cont./chiral fit
JLQCD 14	[360]	$2+1$	99	$\epsilon$ Expansion
Boyle 14	[372]	$2+1$	171	Combines latt/pheno
BMW 13	[35]	$2+1$	120	NLO and NNLO *SU*(2) fits tested with x and $\xi$ expansion
RBC/UKQCD 12	[31]	$2+1$	293 plus run at 171, 246	NLO *SU*(2) $\chi$PT incl. finite-V and some discr. effects
Borsanyi 12	[34]	$2+1$	135	NNLO *SU*(2) chiral fit
NPLQCD 11	[40]	$2+1$	235	NNLO *SU*(2) mixed action $\chi$PT
PACS-CS 11A	[361]	$2+1$	296	Additional test runs at physical point
JLQCD/TWQCD 09, 10A	[338]	$2+1,3$	100($\epsilon$-reg.), 290(*p*-reg.)	$N_f=2+1$ runs both in $\epsilon$- and *p*-regime; $N_f=3$ runs only in *p*-regime. NLO $\chi$PT fit of the spectral density interpolating the two regimes
RBC/UKQCD 09, 10A	[144, 373]	$2+1$	290–420	Valence pions mass is 225–420 MeV. NLO *SU*(2) $\chi$PT fit
MILC 09, 09A, 10, 10A	[6, 13, 29, 89]	$2+1$	258	Lightest Nambu–Goldstone mass is 224 MeV and lightest RMS mass is 258 MeV (at 0.06 fm)

Collaboration	Refs.	$N_{ f}$	$M_{\pi ,{\mathrm{min}}}$ [MeV]	Description
TWQCD 08	[340]	$2+1$	$m_{ud}=m_s/4$, $m_s\sim$ phys	Quark condensate extracted from topological susceptibility, LO chiral fit
PACS-CS 08	[93]	$2+1$	156	Simulation at physical point
RBC/UKQCD 08	[145]	$2+1$	330	Lightest velence pion mass is $242~\,\mathrm {MeV}$
RBC/UKQCD 08A	[339]	$2+1$	330	Computed at one pion mass
NPLQCD 06	[238]	$2+1$	460	Value refers to lightest RMS mass at $a=0.125$ fm as quoted in [6]
LHP 04	[362]	$2+1$	318	Vector meson dominance fit

**Table 93:** Chiral-extrapolation/minimum pion mass in $N_{ f}=2$ determinations of the Low-Energy Constants

Collaboration	Refs.	$N_{ f}$	$M_{\pi ,{\mathrm{min}}}$ [MeV]	Description
ETMC 15A	[333]	2	134	Simulation at physical point
Gülpers 15	[355]	2	193	NLO *SU*(2) fit
Engel 14	[38]	2	193	NLO *SU*(2) fit, Dirac op. and GMOR for $\Sigma$
Gülpers 13	[356]	2	280	NLO $\chi$PT fit
Brandt 13	[37]	2	280	Configurations are shared with CLS
QCDSF 13	[353]	2	130	Fit with $\chi$PT $+$ analytic
ETM 12, 13	[33, 342]	2	260	Confs shared with ETM 09C
Bernardoni 11	[343]	2	312	Overlap valence $+$ $\mathcal {O}(a)$ improved Wilson sea, mixed regime $\chi$PT
TWQCD 11	[249]	2	230	NLO *SU*(2) $\chi$PT fit
TWQCD 11A	[344]	2	220	NLO $\chi$PT
Bernardoni 10	[345]	2	297, 377, 426	NLO *SU*(2) fit of $\chi _\mathrm{top}$
JLQCD/TWQCD 10A	[338]	2	$\sqrt{2m_\mathrm{min}\Sigma }/F=120$ ($\epsilon$-reg.), 290 (*p*-reg.)	Data both in the *p* and $\epsilon$-regime. NLO chiral fit of the spectral density interpolating the two regimes
JLQCD/TWQCD 09	[357]	2	290	LECs extracted from NNLO chiral fit of vector and scalar radii $\langle r^2\rangle ^\pi _{V,S}$
ETM 09B	[346]	2	$\sqrt{2m_\mathrm{min}\Sigma }/F=85$	NLO *SU*(2) $\epsilon$-regime fit
ETM 09C	[36]	2	280	NNLO *SU*(2) fit
ETM 08	[41]	2	260	From pion form factor using NNLO $\chi$PT and exp. value of $\langle r^2\rangle ^\pi _{S}$
JLQCD/TWQCD 08A	[138]	2	290	NNLO *SU*(2) fit
JLQCD 08A	[374]			
CERN 08	[302]	2	$m_{q,{\mathrm{min}}}=13$ MeV	NLO *SU*(2) fit for the mode number
Hasenfratz 08	[347]	2	$\sqrt{2m_\mathrm{min}\Sigma }/F=220$	NLO *SU*(2) $\epsilon$-regime fit
JLQCD/TWQCD 07	[348]	2	$\sqrt{2m_\mathrm{min}\Sigma }/F=120$	NLO *SU*(2) $\epsilon$-regime fit
JLQCD/TWQCD 07A	[349]	2	$m_{ud}= m_s/6-m_s$	$\Sigma$ from $\chi _{t}$, LO chiral fit
CERN-TOV 06	[358]	2	403, 381, 377	NLO *SU*(2) fit
QCDSF/UKQCD 06A	[363]	2	400	Several fit functions to extrapolate the pion form factor

**Table 94:** Finite-volume effects in $N_{ f}=2+1+1$ and $2+1$ determinations of the low-energy constants

Collaboration	Refs.	$N_{ f}$	*L* [fm]	$M_{\pi ,{\mathrm{min}}}L$	Description
HPQCD 15B	[336]	$2+1+1$		4.8	
HPQCD 13A	[26]	$2+1+1$	4.8–5.5	3.3	3 Volumes are compared
ETM 13	[33]	$2+1+1$	1.9–2.8	3.0	4 Volumes compared
ETM 10, 11	[39, 352]	$2+1+1$	1.9–2.8	3.0	FSE estimate using [82]. $M_{\pi ^+} L\gtrsim 4$, but $M_{\pi ^0} L \sim 2$
RBC/UKQCD 15E	[335]	$2+1$		3.78	1 Volume
JLQCD 15A	[359]	$2+1$		3.88	1 Volume
RBC/UKQCD 14B	[10]	$2+1$		5.476	1 Volume
JLQCD 14	[360]	$2+1$		1.8	$\epsilon$-Regime
Boyle 14	[372]	$2+1$		4.6	1 Volume
BMW 13	[35]	$2+1$	2.1	3.0	3 Volumes are compared
RBC/UKQCD 12	[31]	$2+1$	2.7–4.6	${>}4$	FSE seem to be very small
Borsanyi 12	[34]	$2+1$	3.9	3.3	Expected to be less than 1%
NPLQCD 11	[40]	$2+1$	2.5–3.5	3.6	Expected to be less than 1%
MILC 09, 09A, 10, 10A	[6, 13, 29, 89]	$2+1$	2.52	3.5–4.11	$L\ge 2.9~\mathrm{fm}$ for lighter masses
JLQCD/TWQCD 09, 10A	[338]	$2+1$, 3	1.9, 2.7		2 Volumes are compared for a fixed quark mass
RBC/UKQCD 09, 10A	[144, 373]	$2+1$	2.7	$\simeq$4	FSE estimated using $\chi$PT
TWQCD 08	[340]	$2+1$	1.95	–	No estimate of FSE
PACS-CS 08, 11A	[93, 361]	$2+1$	2.9	2.3	FSE is the main concern of the authors. Additional test runs on $64^4$
RBC/UKQCD 08	[145]	$2+1$	2.74	4.6	FSE by means of $\chi$PT
RBC/UKQCD 08A	[339]	$2+1$	2.74	4.6	FSE estimated to be ${<} 1 \%$
NPLQCD 06	[238]	$2+1$	2.5	3.7	Value refers to lightest valence pion mass
LHP 04	[362]	$2+1$	${\simeq } 2.4$	3.97	Value refers to domain-wall valence pion mass

**Table 95:** Finite-volume effects in $N_{ f}=2$ determinations of the low-energy constants

Collaboration	Refs.	$N_{ f}$	*L* [fm]	$M_{\pi ,{\mathrm{min}}}L$	Description
ETMC 15A	[333]	2		4.39	2 Volumes
Gülpers 15	[355]	2		4.09	3 Volumes, CLS confs
Engel 14	[38]	2		4.2	3 Volumes, CLS confs
Gülpers 13	[356]	2	4–6	4.3	Configs. shared with CLS
Brandt 13	[37]	2	${\sim } 5$	4	Configs. shared with CLS
QCDSF 13	[353]	2	1.8–2.4	2.7	NLO $\chi$PT is used for FSE
Bernardoni 11	[343]	2	1.56	2.5	Mixed regime $\chi$PT for FSE used
TWQCD 11	[249]	2	1.65	1.92	*SU*(2) $\chi$PT is used for FSE
TWQCD 11A	[344]	2	1.65	1.8	No estimate of FSE
Bernardoni 10	[345]	2	1.88	2.8	FSE included in the NLO chiral fit
JLQCD/TWQCD 10A	[338]	2	1.8–1.9		FSE estimated from different topological sectors
JLQCD/TWQCD 09	[357]	2	1.89	2.9	FSE by NLO $\chi$PT, additional FSE for fixing topology [792]
ETM 09B	[346]	2	1.3, 1.5	$\epsilon$-Regime	Topology: not fixed. 2 volumes
ETM 09C, 12, 13	[33, 36, 342]	2	2.0–2.5	3.2–4.4	Several volumes. Finite-volume effects estimated through [82]
ETM 08	[41]	2	2.1, 2.8	3.4, 3.7	Only data with $M_\pi L\gtrsim 4$ are considered
JLQCD/TWQCD 08A	[138]	2	1.89	2.9	FSE estimates through [82]. Additional FSE for fixing topology [792]
JLQCD 08A	[374]				
CERN 08	[302]	2	1.88, 2.51	–	Two volumes compared
Hasenfratz 08	[347]	2	1.84, 2.77	$\epsilon$-Regime	Topology: not fixed, 2 volumes
JLQCD/TWQCD 07	[348]	2	1.78	$\epsilon$-Regime	Topology: fixed to $\nu =0$
JLQCD/TWQCD 07A	[349]	2	1.92	–	Topology fixed to $\nu =0$ [792]
CERN-TOV 06	[358]	2	1.72, 1.67, 1.88	3.5, 3.2, 3.6	No estimate for FSE
QCDSF/UKQCD 06A	[363]	2	1.4–2.0	3.8	NLO $\chi$PT estimate for FSE [793]

**Table 96:** Renormalization in determinations of the low-energy constants

Collaboration	Refs.	$N_{ f}$	Description
HPQCD 15B	[336]	$2+1+1$	–
HPQCD 13A	[26]	$2+1+1$	–
ETM 10, 11, 13	[33, 39, 352]	$2+1+1$	Nonperturbative
RBC/UKQCD 15E	[335]	$2+1$	RI-SMOM
JLQCD 15A	[359]	$2+1$	RI-MOM
RBC/UKQCD 14B	[10]	$2+1$	RI-SMOM
JLQCD 14	[360]	$2+1$	–
Boyle 14	[372]	$2+1$	–
BMW 13	[35]	$2+1$	Nonperturbative
RBC/UKQCD 12	[31]	$2+1$	Nonperturbative (RI/SMOM)
Borsanyi 12	[34]	$2+1$	Indirectly nonperturbative through [7] for $\Sigma$; no renormalization needed for *F*, since only $F_\pi /F$ computed and scale set through $F_\pi$
NPLQCD 11	[40]	$2+1$	Not needed (no result for $\Sigma$)
JLQCD/TWQCD 10A	[338]	$2+1$, 3	Nonperturbative
MILC 09, 09A, 10, 10A	[6, 13, 29, 89]	$2+1$	2 Loop
RBC/UKQCD 10A	[144]	$2+1$	Nonperturbative
JLQCD 09	[337]	$2+1$	Nonperturbative
TWQCD 08	[340]	$2+1$	Nonperturbative
PACS-CS 08	[93]	$2+1$	1 Loop
RBC/UKQCD 08, 08A	[145, 339]	$2+1$	Nonperturbative
NPLQCD 06	[238]	$2+1$	–
LHP 04	[362]	$2+1$	–
All collaborations		2	Nonperturbative

### B.4 Notes to Sect. 6 on Kaon mixing

#### B.4.1 Kaon *B*-parameter $B_K$

See Tables 97, 98, 99, 100 and 101.

**Table 97:** Continuum extrapolations/estimation of lattice artefacts in determinations of $B_K$

Collaboration	Refs.	$N_{ f}$	*a* [fm]	Description
ETM 15	[42]	$2+1+1$	0.09, 0.08, 0.06	Combined chiral and continuum extrapolation. Systematic error of 2.0% is obtained from the distribution of results over analyses which differ by $\mathcal {O}(a^2)$ effects
SWME 15A	[45]	$2+1$	0.12, 0.09, 0.06, 0.045	The three finest lattice spacings are used for the combined chiral and continuum extrapolation. Residual combined discretization, sea-quark extrapolation and $\alpha _s$ matching error of 4.4% from difference between linear fit in $a^2$, $m_\mathrm{sea}$ and a fit where $\alpha _s$ dependence is added
RBC/UKQCD 14B	[10]	$2+1$	0.111, 0.083, 0.063, 0.114, 0.084	The three first lattice spacings use different action from the last two ones. Combined continuum and chiral fits
SWME 14	[385]	$2+1$	0.082, 0.059, 0.044	Residual combined discretization and sea-quark extrapolation error of 0.9% from difference between linear fit in $a^2$, $m_\mathrm{sea}$ and a constrained nine-parameter extrapolation
SWME 13A	[402]	$2+1$	0.09, 0.06, 0.045	Residual combined discretization, sea-quark extrapolation and $\alpha _s$ matching error of 4.4% from difference between linear fit in $a^2$, $m_\mathrm{sea}$ and a fit where $\alpha _s$ dependence is added
SWME 13	[403]	$2+1$	0.12, 0.09, 0.06, 0.045	Continuum extrapolation with the coarsest lattice spacing omitted; residual combined discretization and sea-quark extrapolation error of 1.1% from difference between linear fit in $a^2$, $m_\mathrm{sea}$ and a constrained nine-parameter extrapolation
RBC/UKQCD 12A	[31]	$2+1$	0.146, 0.114, 0.087	Coarsest lattice spacing uses different action. Combined continuum and chiral fits
Laiho 11	[44]	$2+1$	0.12, 0.09, 0.06	Combined continuum and chiral extrapolation based on *SU*(3) mixed-action partially quenched $\chi$PT
SWME 11, 11A	[404, 787]	$2+1$	0.12, 0.09, 0.06, 0.045	Continuum extrapolation with the coarsest lattice spacing omitted; residual discretization error of 1.9% from difference between fit to a constant and a constrained five-parameter extrapolation
BMW 11	[43]	$2+1$	0.093, 0.077, 0.065, 0.054	Combined continuum and chiral extrapolation; discretization error of 0.1% from comparison of $\mathcal {O}({\alpha _s}a)$ and $\mathcal {O}(a^2)$ extrapolations
RBC/UKQCD 10B	[405]	$2+1$	0.114, 0.087	Two lattice spacings. Combined chiral and continuum fits
SWME 10	[278]	$2+1$	0.12, 0.09, 0.06	Continuum extrapolation of results obtained at four lattice spacings; residual discretization error of 0.21% from difference to result at smallest lattice spacing
Aubin 09	[406]	$2+1$	0.12, 0.09	Two lattice spacings; quote 0.3% discretization error, estimated from various $a^2$-terms in fit function
RBC/UKQCD 07A, 08	[145, 407]	$2+1$	0.114(2)	Single lattice spacing; quote 4% discretization error, estimated from the difference between computed and experimental values of $f_\pi$
HPQCD/UKQCD 06	[408]	$2+1$	0.12	Single lattice spacing; 3% discretization error quoted without providing details
ETM 12D	[46]	2	0.1, 0.09, 0.07, 0.05	Four lattice spacings; systematic quoted obtained from the difference between the finest lattice spacing and the continuum limit and comparing results using two evaluations of the RCs that differ by $\mathcal {O}(a^2)$ effects
ETM 10A	[401]	2	0.1, 0.09, 0.07	Three lattice spacings; 1.2% error quoted
JLQCD 08	[409]	2	0.118(1)	Single lattice spacing; no error quoted
RBC 04	[400]	2	0.117(4)	Single lattice spacing; no error quoted
UKQCD 04	[410]	2	0.10	Single lattice spacing; no error quoted

**Table 98:** Chiral-extrapolation/minimum pion mass in determinations of $B_K$

Collaboration	Refs.	$N_{ f}$	$M_{\pi ,\mathrm{min}}\,[\mathrm{MeV}]$	Description
ETM 15	[42]	$2+1+1$	245, 239, 211	Each $M_{\pi ,\mathrm min}$ entry corresponds to a different lattice spacing. Simultaneous chiral and continuum extrapolations, based on polynomial and $\chi$PT at NLO, are carried out leads to systematic error of 0.8%
SWME 15A	[45]	$2+1$	222/372, 206/174, 195/222, 206/316	Valence/sea RMS $M_{\pi ,\mathrm{min}}$ entries correspond to the four lattice spacings (the last three are used for the chiral-continuum extrapolation). Chiral extrapolations based on *SU*(2) staggered $\chi$PT at NNLO (with some coefficients fixed by Bayesian priors), and also including one analytic NNNLO term. Residual error of 0.05% from changing the Bayesian priors and fit method
RBC/UKQCD 14B	[10]	$2+1$	337, 302, 371, 139, 139	$M_{\pi ,\mathrm{min}}$ entries correspond to the five lattice spacings. Combined chiral and continuum extrapolation, using $M_\pi <260$ MeV and $M_\pi <370$ MeV
SWME 14	[385]	$2+1$	206/174, 195/222, 207/316	Valence/sea RMS $M_{\pi ,\mathrm{min}}$ entries correspond to the three lattice spacings. Chiral extrapolations based on *SU*(2) staggered $\chi$PT at NNLO (with some coefficients fixed by Bayesian priors), and also including one analytic NNNLO term. Residual error of 0.1% error from doubling the widths of Bayesian priors
SWME 13A	[402]	$2+1$	207/243, 196/262, 207/316	Valence/sea RMS $M_{\pi ,\mathrm{min}}$ entries correspond to the three lattice spacings. Chiral extrapolations based on *SU*(2) staggered $\chi$PT at NNLO (with some coefficients fixed by Bayesian priors), and also including one analytic NNNLO term. Residual error of 0.1% from doubling the widths of Bayesian priors
SWME 13	[403]	$2+1$	442/445, 299/273, 237/256, 222/334	Valence/sea RMS $M_{\pi ,\mathrm{min}}$ entries correspond to the four lattice spacings. Chiral extrapolations based on *SU*(2) staggered $\chi$PT at NNLO (with some coefficients fixed by Bayesian priors), and also including one analytic NNNLO term. Residual error of 0.33% error from doubling the widths of Bayesian priors
RBC/UKQCD 12A	[31]	$2+1$	140/170, 240/330, 220/290	Valence/sea $M_{\pi ,\mathrm{min}}$ entries correspond to the three lattice spacings. Combined chiral and continuum extrapolation, using $M_\pi <350$ MeV
Laiho 11	[44]	$2+1$	210/280	$M_{\pi ,\mathrm{min}}$ entries correspond to the smallest valence/sea-quark masses. Chiral and continuum fits based on NLO mixed action $\chi$PT, including a subset of NNLO terms. Systematic error estimated from spread arising from variations in the fit function
SWME 11, 11A	[404, 787]	$2+1$	442/445, 299/325, 237/340, 222/334	Valence/sea RMS $M_{\pi ,\mathrm{min}}$ entries correspond to the four lattice spacings. Chiral extrapolations based on *SU*(2) staggered $\chi$PT at NNLO (with some coefficients fixed by Bayesian priors), and also including one analytic NNNLO term. Residual error of 0.33% error from doubling the widths of Bayesian priors
BMW 11	[43]	$2+1$	219, 182, 120, 131	$M_{\pi ,\mathrm{min}}$ entries correspond to the four lattice spacings used in the final result. Combined fit to the chiral and continuum behaviour. Systematics investigated by applying cuts to the maximum pion mass used in fits. Uncertainty of 0.1% assigned to chiral fit
RBC/UKQCD 10B	[405]	$2+1$	240/330, 220/290	Valence/sea $M_{\pi ,\mathrm{min}}$ entries correspond to the two lattice spacings. Combined chiral and continuum extrapolations
SWME 10	[278]	$2+1$	442/445, 299/325, 237/340	Valence/sea $M_{\pi ,\mathrm{min}}$ entries correspond to the three lattice spacings. Chiral extrapolations based on *SU*(2) staggered $\chi$PT at NLO, including some analytic NNLO terms. *SU*(3) staggered $\chi$PT as cross-check. Combined 1.1% error from various different variations in the fit procedure
Aubin 09	[406]	$2+1$	240/370	$M_{\pi ,\mathrm{min}}$ entries correspond to the smallest valence/sea-quark masses. Chiral and continuum fits based on NLO mixed action $\chi$PT at NLO, including a subset of NNLO terms. Systematic error estimated from spread arising from variations in the fit function
RBC/UKQCD 07A, 08	[145, 407]	$2+1$	330	Fits based on *SU*(2) PQ$\chi$PT at NLO. Effect of neglecting higher orders estimated at 6% via difference between fits based on LO and NLO expressions
HPQCD/UKQCD 06	[408]	$2+1$	360	3% Uncertainty from chiral extrapolation quoted, without giving further details
ETM 12D	[46]	2	400, 280, 300, 280	Each $M_{\pi ,\mathrm{min}}$ entry corresponds to a different lattice spacing. Simultaneous chiral and continuum extrapolations, based on polynomial and $\chi$PT at NLO, are carried out. Systematic error from several sources, including lattice calibration, quark mass calibration, chiral and continuum extrapolation etc., estimated at 3.0%
ETM 10A	[401]	2	400, 280, 300	Each $M_{\pi ,\mathrm{min}}$ entry corresponds to a different lattice spacing. Simultaneous chiral and continuum extrapolations, based on $\chi$PT at NLO, are carried out. Systematic error from several sources, including lattice calibration, quark mass calibration, chiral and continuum extrapolation etc., estimated at 3.1%
JLQCD 08	[409]	2	290	Fits based on NLO PQ$\chi$PT. Range of validity investigated. Fit error included in statistical uncertainty
RBC 04	[400]	2	490	Fits based on NLO PQ$\chi$PT. Fit error included in statistical uncertainty
UKQCD 04	[410]	2	780	Fits to continuum chiral behaviour at fixed sea-quark mass. Separate extrapolation in sea quark mass. Fit error included in overall uncertainty

**Table 99:** Finite-volume effects in determinations of $B_K$. If partially quenched fits are used, the quoted $M_{\pi ,\mathrm{min}}L$ is for lightest valence (RMS) pion

ETM 15	[42]	$2+1+1$	2.1–2.8, 2.6, 3.0	3.5, 3.2, 3.2	Each *L* entry corresponds to a different lattice spacing, with two volumes at the coarsest lattice spacing; results from these two volumes at $M_{\pi } \sim 280$ MeV are compatible
SWME 15A	[45]	$2+1$	2.4–3.4, 2.5–5.8, 2.9–3.9, 2.9	${\gtrsim } 3.8$	*L* entries correspond to the four lattice spacings, with several volumes in most cases. Finite-volume effects estimated using NLO *SU*(2) S$\chi$PT
RBC/UKQCD 14B	[10]	$2+1$	2.7, 2.7, 2.0, 5.5, 5.3	${\gtrsim } 3.8$	*L* entries correspond to the five lattice spacings. Finite-volume effects estimated using NLO $\chi$PT; negligible with comparison to the statistical error
SWME 14	[385]	$2+1$	2.8–5.4, 2.8–3.8, 2.8	5.6, 3.7, 2.9	*L* entries correspond to the three lattice spacings, with several volumes in most cases. Finite-volume effects estimated using NLO $\chi$PT
SWME 13A	[402]	$2+1$	2.4–3.4, 2.8–3.8, 2.8	3.5, 3.3, 2.9	*L* entries correspond to the three lattice spacings, with several volumes in most cases. Finite-volume effects estimated using NLO $\chi$PT
SWME 13	[403]	$2+1$	2.4–3.3, 2.4–5.5, 2.8–3.8, 2.8	${\gtrsim } 3.2$	*L* entries correspond to the four lattice spacings, with several volumes in most cases. Finite-volume effects estimated using NLO $\chi$PT
RBC/UKQCD 12A	[31]	$2+1$	4.6, 2.7, 2.8	${\gtrsim } 3.2$	*L* entries correspond to the three lattice spacings. Finite-volume effects estimated using NLO $\chi$PT
Laiho 11	[44]	$2+1$	2.4, 3.4, 3.8	${\gtrsim } 3.5$	*L* entries correspond to the three lattice spacings. Finite-volume effects estimated using NLO $\chi$PT
SWME 11, 11A	[404, 787]	$2+1$	2.4/3.3, 2.4, 2.8, 2.8	${\gtrsim } 3.2$	*L* entries correspond to the four lattice spacings, with two volumes at the coarsest lattice. Finite-volume effects estimated using NLO $\chi$PT
BMW 11	[43]	$2+1$	6.0, 4.9, 4.2, 3.5	${\gtrsim } 3.8, 3.0$	*L* entries correspond to the four lattice spacings, and are the largest of several volumes at each *a*. ${M_{\pi ,\mathrm min}}L\approx 3.0$ for the ensemble at $a\approx 0.08~\mathrm{fm}$. Finite-volume effects estimated in $\chi$PT and by combined fit to multiple volumes
RBC/UKQCD 10B	[405]	$2+1$	2.7, 2.8	${\gtrsim } 3.1$	*L* entries correspond to the three lattice spacings. Finite-volume effects estimated using NLO $\chi$PT
SWME 10	[278]	$2+1$	2.4/3.3, 2.4, 2.8	${\gtrsim } 3.4$	*L* entries correspond to the three lattice spacings, with two volumes for the coarsest spacing. Finite-volume error of 0.9% estimated from difference obtained these two volumes
Aubin 09	[406]	$2+1$	2.4, 3.4	3.5	*L* entries correspond to the two lattice spacings. Keep $m_{\pi }L\;\mathop {\sim }\limits ^{{>}}\;3.5$; no comparison of results from different volumes; 0.6% error estimated from mixed action $\chi$PT correction
RBC/UKQCD 07A, 08	[145, 407]	$2+1$	1.83/2.74	4.60	Each *L* entry corresponds to a different volume at the same lattice spacing; 1% error from difference in results on two volumes
HPQCD/UKQCD 06	[408]	$2+1$	2.46	4.49	Single volume; no error quoted
ETM 12D	[46]	2	2.1 2.2/2.9, 2.2, 2.6	5, 3.3/4.3, 3.3, 3.5	Each *L* entry corresponds to a different lattice spacing, with two volumes at the second less coarse lattice spacing. Results from these two volumes at $M_{\pi } \sim 300$ MeV are compatible
ETM 10A	[401]	2	2.1, 2.2/2.9, 2.2	5, 3.3/4.3, 3.3	Each *L* entry corresponds to a different lattice spacing, with two volumes at the intermediate lattice spacing. Results from these two volumes at $M_{\pi } \sim 300$ MeV are compatible
JLQCD 08	[409]	2	1.89	2.75	Single volume; data points with $m_\mathrm{val} < m_\mathrm{sea}$ excluded; 5% error quoted as upper bound of PQ$\chi$PT estimate of the effect
RBC 04	[400]	2	1.87	4.64	Single volume; no error quoted
UKQCD 04	[410]	2	1.6	6.51	Single volume; no error quoted

**Table 100:** Running and matching in determinations of $B_K$ for $N_f=2+1+1$ and $N_f=2+1$

Collaboration	Refs.	$N_{ f}$	Ren.	Running match.	Description
ETM 15	[42]	$2+1+1$	RI	PT1$\ell$	Uncertainty from RI renormalization estimated at 2%. Additional error of 0.6% for the conversion to ${\overline{\text {MS}}}$
SWME 15A	[45]	$2+1$	PT1$\ell$	PT1$\ell$	Uncertainty from neglecting higher orders estimated at 4.4% by identifying the unknown two-loop coefficient with result at the smallest lattice spacing
RBC/UKQCD 14B	[10]	$2+1$	RI	PT1$\ell$	Two different RI-SMOM schemes used to estimate 2% systematic error in conversion to ${\overline{\text {MS}}}$
SWME 14	[385]	$2+1$	PT1$\ell$	PT1$\ell$	Uncertainty from neglecting higher orders estimated at 4.4% by identifying the unknown two-loop coefficient with result at the smallest lattice spacing
SWME 13A	[402]	$2+1$	PT1$\ell$	PT1$\ell$	Uncertainty from neglecting higher orders estimated at 4.4% (in combination with systematic uncertainty from CL and chiral extrapolation fit) by identifying the unknown two-loop coefficient with result at the smallest lattice spacing
SWME 13	[403]	$2+1$	PT1$\ell$	PT1$\ell$	Uncertainty from neglecting higher orders estimated at 4.4% by identifying the unknown two-loop coefficient with result at the smallest lattice spacing
RBC/UKQCD 12A	[31]	$2+1$	RI	PT1$\ell$	Two different RI-SMOM schemes used to estimate 2% systematic error in conversion to ${\overline{\text {MS}}}$
Laiho 11	[44]	$2+1$	RI	PT1$\ell$	Total uncertainty in matching and running of 3%. Perturbative truncation error in the conversion to ${\overline{\text {MS}}}$, RGI schemes is dominant uncertainty
SWME 11, 11A	[404, 787]	$2+1$	PT1$\ell$	PT1$\ell$	Uncertainty from neglecting higher orders estimated at 4.4% by identifying the unknown two-loop coefficient with result at the smallest lattice spacing
BMW 11	[43]	$2+1$	RI	PT1$\ell$	Uncertainty of 0.05% in the determination of the renormalization factor included. 1% error estimated due to truncation of perturbative matching to ${\overline{\text {MS}}}$ and RGI schemes at NLO
RBC/UKQCD 10B	[405]	$2+1$	RI	PT1$\ell$	Variety of different RI-MOM schemes including non-exceptional momenta. Residual uncertainty of 2% uncertainty in running and matching
SWME 10	[278]	$2+1$	PT1$\ell$	PT1$\ell$	Uncertainty from neglecting higher orders estimated at 5.5% by identifying the unknown two-loop coefficient with result at the smallest lattice spacing
Aubin 09	[406]	$2+1$	RI	PT1$\ell$	Total uncertainty in matching and running of 3.3%, estimated from a number of sources, including a chiral-extrapolation fit ansatz for n.p. determination, strange sea-quark mass dependence, residual chiral symmetry breaking, perturbative matching and running
RBC/UKQCD 07A, 08	[145, 407]	$2+1$	RI	PT1$\ell$	Uncertainty from n.p. determination of ren. factor included in statistical error; 2% systematic error from perturbative matching to ${\overline{\text {MS}}}$ estimated via size of correction itself
HPQCD/UKQCD 06	[408]	$2+1$	PT1$\ell$	PT1$\ell$	Uncertainty due to neglecting two-loop order in perturbative matching and running estimated by multiplying result by $\alpha ^2$

**Table 101:** Running and matching in determinations of $B_K$ for $N_f=2$

Collaboration	Refs.	$N_{ f}$	Ren.	Running match.	Description
ETM 12D	[46]	2	RI	PT1$\ell$	Uncertainty from RI renormalization estimated at 2.5%
ETM 10A	[401]	2	RI	PT1$\ell$	Uncertainty from RI renormalization estimated at 2.5%
JLQCD 08	[409]	2	RI	PT1$\ell$	Uncertainty from n.p. determination of ren. factor included in statistical error; 2.3% systematic error from perturbative matching to ${\overline{\text {MS}}}$ estimated via size of correction itself
RBC 04	[400]	2	RI	PT1$\ell$	Uncertainty from n.p. determination of ren. factor included
UKQCD 04	[410]	2	PT1$\ell$	PT1$\ell$	No error quoted

#### B.4.2 Kaon BSM *B*-parameters

See Tables 102, 103, 104 and 105.

**Table 102:** Continuum extrapolations/estimation of lattice artefacts in determinations of the BSM $B_i$ parameters

Collaboration	Refs.	$N_{ f}$	*a* [fm]	Description
ETM 15	[42]	$2+1+1$	0.09, 0.08, 0.06	Combined chiral and continuum extrapolation. Systematic errors to $B_{i}$ from about 4 to 6% are obtained from the distribution of results over analyses which differ by $\mathcal {O}(a^2)$ effects
SWME 15A	[45]	$2+1$	0.12, 0.09, 0.06, 0.045	The three finest lattice spacings are used for the combined chiral and continuum extrapolation. Residual combined discretization, sea-quark extrapolation and $\alpha _s$ matching error from about 4.4 to 9.6% is reported for $B_{i}$ and is obtained from the difference between linear fit in $a^2$, $m_\mathrm{sea}$ and a fit where $\alpha _s$ dependence is added
SWME 14C	[417]	$2+1$	0.082, 0.059, 0.044	Residual combined discretization and sea-quark extrapolation error of 1–8% from difference between linear fit in $a^2$, $m_\mathrm{sea}$ and a constrained 19-parameter extrapolation
SWME 13A	[402]	$2+1$	0.09, 0.06, 0.045	Residual combined discretization, sea-quark extrapolation and $\alpha _s$ matching error for $B_i$ varies from 4.5 to −5.7%, from difference between linear fit in $a^2$, $m_\mathrm{sea}$ and a fit where $\alpha _s$ dependence is added
RBC/UKQCD 12E	[412]	$2+1$	0.087	Computation at only one value of the lattice spacing. Estimate for the systematic discretization error of about 1.5% based on the corresponding estimate from the $B_K$ computation
ETM 12D	[46]	2	0.1, 0.09, 0.07, 0.05	Four lattice spacings; estimates of systematic uncertainties obtained from the half difference of the distance between the finest lattice spacing and the continuum limit

**Table 103:** Chiral-extrapolation/minimum pion mass in determinations of the BSM $B_i$ parameters

Collaboration	Refs.	$N_{ f}$	$M_{\pi ,\mathrm{min}}\,[\mathrm{MeV}]$	Description
ETM 15	[42]	$2+1+1$	245, 239, 211	Each $M_{\pi ,\mathrm{min}}$ entry corresponds to a different lattice spacing. Simultaneous chiral and continuum extrapolations, based on polynomial and $\chi$PT at NLO, are carried out leads to systematic errors of $1.1{-}2.6$% depending on the bag parameter
SWME 15A	[45]	$2+1$	222/372, 206/174, 195/222, 206/316	Valence/sea RMS $M_{\pi ,\mathrm{min}}$ entries correspond to the four lattice spacings (the last three are used for the chiral-continuum extrapolation). Chiral extrapolations based on *SU*(2) staggered $\chi$PT at NNLO (with some coefficients fixed by Bayesian priors), and also including one analytic NNNLO term. Residual error of 0.4–1.2% depending on the bag parameter from changing the Bayesian priors and fit method
SWME 14C	[417]	$2+1$	206/174, 195/222, 207/316	Valence/sea RMS $M_{\pi ,\mathrm{min}}$ entries correspond to the three lattice spacings. Chiral extrapolations performed via $B_i$-ratios that do not show *SU*(2) NLO $\chi$PT contribution and assuming various terms up to NNLO (with some coefficients fixed by Bayesian priors)
SWME 13A	[402]	$2+1$	207/243, 196/262, 207/316	Valence/sea RMS $M_{\pi ,\mathrm{min}}$ entries correspond to the three lattice spacings. Chiral extrapolations performed via $B_i$-ratios that do not show *SU*(2) NLO $\chi$PT contribution and assuming various terms up to NNLO (with some coefficients fixed by Bayesian priors). Residual error in the valence of about 0.1% from doubling the widths of Bayesian priors. In the sea a combined error with the matching procedure of 4.4–5.6% is reported
RBC/UKQCD 12E	[412]	$2+1$	290/290	Chiral extrapolations based on polynomial and $\chi$PT fits at NLO are carried out. Central values are obtained from polynomial fits. Mild dependence on the quark mass. Systematic uncertainties are estimated to about 4% for all $B_i$’s
ETM 12D	[46]	2	400, 270, 300, 270	Each $M_{\pi ,\mathrm{min}}$ entry corresponds to a different lattice spacing. Simultaneous chiral and continuum extrapolations, based on polynomial and $\chi$PT at NLO, are carried out

**Table 104:** Finite-volume effects in determinations of the BSM $B_i$ parameters. If partially quenched fits are used, the quoted $M_{\pi ,\mathrm min}L$ is for lightest valence (RMS) pion

Collaboration	Refs.	$N_{ f}$	*L* [fm]	${M_{\pi ,\mathrm{min}}}L$	Description
ETM 15	[42]	$2+1+1$	2.1–2.8, 2.6, 3.0	3.5, 3.2, 3.2	Each *L* entry corresponds to a different lattice spacing, with two volumes at the coarsest lattice spacing; results from these two volumes at $M_{\pi } \sim 280$ MeV are compatible
SWME 15A	[45]	$2+1$	2.4–3.4, 2.5–5.8, 2.9–3.9, 2.9	${\gtrsim } 3.8$	*L* entries correspond to the four lattice spacings, with several volumes in most cases. Finite-volume effects estimated using NLO *SU*(2) S$\chi$PT
SWME 14C	[417]	$2+1$	2.8–5.4, 2.8–3.8, 2.8	5.6, 3.7, 2.9	*L* entries correspond to the three lattice spacings, with several volumes in most cases. Finite-volume effects estimated using NLO $\chi$PT
SWME 13A	[402]	$2+1$	2.4–3.4, 2.8–3.3, 2.8	3.5, 3.3, 2.9	*L* entries correspond to the three lattice spacings, with several volumes in most cases. Finite-volume effects estimated using NLO $\chi$PT
RBC/UKQCD 12E	[412]	$2+1$	2.8	${\gtrsim } 4.0$	The *L* value corresponds to the unique lattice spacing. Finite-volume effects, estimated using NLO $\chi$PT are small, as it has also been found in the $B_K$ computation, and they have thus been neglected in the final error budget analysis
ETM 12D	[46]	2	2.1, 2.2/2.9, 2.2, 2.6	5, 3.3/4.3, 3.3, 3.5	Each *L* entry corresponds to a different lattice spacing, with two volumes at the second less coarse lattice spacing. Results from these two volumes at $M_{\pi } \sim 300$ MeV are compatible

**Table 105:** Running and matching in determinations of the BSM $B_i$ parameters

Collaboration	Refs.	$N_{ f}$	Ren.	Running match.	Description
ETM 15	[42]	$2+1+1$	RI	PT1$\ell$	Uncertainty from RI renormalization combined with discretization effects estimates are reported to be from about 4 to 6%. Additional error from 1.8 to 3.9% (depending on the bag parameter) for the conversion to ${\overline{\text {MS}}}$ at the scale of 3 GeV
SWME 15A	[45]	$2+1$	PT1$\ell$	PT1$\ell$	Uncertainty from neglecting higher orders estimated from about 4.4 to 9.6% (depending on the bag parameter) by identifying the unknown two-loop coefficient with result at the smallest lattice spacing
SWME 14C	[417]	$2+1$	PT1$\ell$	PT1$\ell$	Uncertainty from neglecting higher orders estimated at 4.4% by identifying the unknown two-loop coefficient with result at the smallest lattice spacing
SWME 13A	[402]	$2+1$	PT1$\ell$	PT1$\ell$	Uncertainty from neglecting higher orders estimated at 4.4% (in combination with systematic uncertainty from CL and chiral-extrapolation fit) by identifying the unknown two-loop coefficient with result at the smallest lattice spacing
RBC/UKQCD 12E	[412]	$2+1$	RI	PT1$\ell$	Computation in RI-MOM scheme. Systematic error from the conversion to ${\overline{\text {MS}}}$ is estimated by taking the half of the difference between the LO and the NLO result
ETM 12D	[46]	2	RI	PT1$\ell$	Uncertainty from RI renormalization estimated at 2.5%

### B.5 Notes to Sect. 7 on *D*-meson-decay constants and form factors

In the following, we summarize the characteristics (lattice actions, pion masses, lattice spacings, etc.) of the recent $N_{ f}=2+1+1$, $N_{ f}= 2+1$ and $N_{ f}= 2$ runs. We also provide brief descriptions of how systematic errors are estimated by the various authors. We focus on calculations with either preliminary or published quantitative results.

#### B.5.1 $D_{(s)}$-meson-decay constants

See Tables 106, 107, 108, 109, 110, 111, 112, 113, 114, 115, 116, 117 and 118.

**Table 106:** Chiral-extrapolation/minimum pion mass in $N_f=2+1+1$ determinations of the *D* and $D_s$-meson-decay constants. For actions with multiple species of pions, masses quoted are the RMS pion masses (where available). The different $M_{\pi ,\mathrm min}$ entries correspond to the different lattice spacings

Collaboration	Refs.	$N_{ f}$	$M_{\pi ,\mathrm{min}}\,[\mathrm{MeV}]$	Description
FNAL/MILC 14A	[14]	$2+1+1$	311, 241, 173, 143	The lightest pions (not RMS) are around 130 MeV. Analyses are performed either by interpolating to the physical point or by using HMrAS$\chi$PT formulae to include heavier masses and non-unitary points. The latter procedure gives more accurate, and final, results
ETM 13F, ETM 14E	[27, 230]	$2+1+1$	245, 239, 211	$f_{D_s}\sqrt{m_{D_s}}$ in ETM 13F and $f_{D_s}/m_{D_s}$ in ETM 14E are extrapolated using both a quadratic and a linear fit in $m_l$ plus $\mathcal {O}(a^2)$ terms. Then the double ratio $(f_{D_s}/f_D)/(f_K/f_\pi )$ is fitted in continuum HM$\chi$PT, as no lattice-spacing dependence is visible within statistical errors
FNAL/MILC 12B, FNAL/MILC 13	[420, 421]	$2+1+1$	310, 245, 179, 145	Chiral and continuum extrapolations are performed simultaneously. Central values are produced using a fit function quadratic in $a^2$ and linear in the sea-quark mass. In FNAL/MILC 13 terms of $\mathcal {O}(a^4)$ are included

**Table 107:** Chiral-extrapolation/minimum pion mass in $N_f=2+1$ determinations of the *D*- and $D_s$-meson-decay constants. For actions with multiple species of pions, masses quoted are the RMS pion masses (where available). The different $M_{\pi ,\mathrm min}$ entries correspond to the different lattice spacings

Collaboration	Refs.	$N_{ f}$	$M_{\pi ,\mathrm{min}}\,[\mathrm{MeV}]$	Description
$\chi$QCD 14	[17]	$2+1$	334, 296	Chiral and continuum extrapolations are performed simultaneously using linear fits in $m_l$ (and quadratic or including partially quenched chiral logs, in order to assess the systematic error) plus terms up to $\mathcal {O}(a^2)$ and $\mathcal {O}(a^4m_c^4)$
HPQCD 12A	[47]	$2+1$	460, 329	Chiral and continuum extrapolations are performed simultaneously using PQHM$\chi$PT augmented by *a* dependent terms: $c_0(am_c)^2 + c_1(am_c)^4$
FNAL/MILC 11	[48]	$2+1$	570, 440, 320	Chiral and continuum extrapolations are performed simultaneously using HM$\chi$PT for rooted staggered quarks. Effects of hyperfine and flavour splittings are also included
PACS-CS 11	[422]	$2+1$	152	Simulations are reweighted in the light- and strange-quark masses to the physical point
HPQCD 10A	[49]	$2+1$	542, 460, 329, 258, 334	Chiral and continuum extrapolations are performed simultaneously. Polynomials up to $\left( \frac{m_{q,\mathrm{sea}}-m_{q,\mathrm{phys}}}{m_{q,\mathrm{phys}}}\right) ^2$ for $q=s,l$ and up to $(am_{c})^8$ are kept
HPQCD/UKQCD 07	[28]	$2+1$	542, 460, 329	Combined chiral and continuum extrapolations using HM$\chi$PT at NLO augmented by second and third-order polynomial terms in $m_q$ and terms up to $a^4$
FNAL/MILC 05	[423]	$2+1$	${>}440, 440, 400$	Chiral extrapolations are first performed at each lattice spacing using NLO HM$\chi$PT for rooted staggered quarks. Lattice artefacts are then extrapolated linearly in $a^2$

**Table 108:** Chiral-extrapolation/minimum pion mass in $N_f=2$ determinations of the *D*- and $D_s$-meson-decay constants. For actions with multiple species of pions, masses quoted are the RMS pion masses (where available). The different $M_{\pi ,\mathrm min}$ entries correspond to the different lattice spacings

Collaboration	Refs.	$N_{ f}$	$M_{\pi ,\mathrm{min}}\,[\mathrm{MeV}]$	Description
TWQCD 14	[424]	2	260	Comparison of NLO HM$\chi$PT fits for $f_{D_{( \mathrm s)}}$ and for $f_{D_{( \mathrm s)}}\sqrt{m_{D_{( \mathrm s)}}}$ in order to asses systematic error
ALPHA 13B	[177]	2	190, 270	Linear fits (in $m_\pi ^2$ and in $a^2$) and partially quenched HM$\chi$PT functional forms, including terms linear in $a^2$, are used in the combined chiral/continuum extrapolation
ETM 09 ETM 11A ETM 13B	[20, 32, 182]	2	410, 270, 310, 270	$M_{\pi ,\mathrm{min}}$ refers to the charged pions. NLO *SU*(2) HM$\chi$PT supplemented by terms linear in $a^2$ and in $m_D a^2$ is used in the combined chiral/continuum extrapolation. To estimate the systematic due to chiral extrapolation, once $f_{D_s}\sqrt{m_{D_s}}$ and $f_{D_s}\sqrt{m_{D_s}}/(f_{D}\sqrt{m_{D}})$ and once $f_{D_s}\sqrt{m_{D_s}}/f_K$ and $f_{D_s}\sqrt{m_{D_s}}/f_K \; \times \; f_\pi /(f_{D}\sqrt{m_{D}})$ are fitted. In ETM 13 the double ratio $(f_{D_s}/f_D)/(f_K/f_\pi )$ is fitted in HM$\chi$PT

**Table 109:** Finite-volume effects in $N_f=2+1+1$ determinations of the *D*- and $D_s$-meson-decay constants. Each *L*-entry corresponds to a different lattice spacing, with multiple spatial volumes at some lattice spacings. For actions with multiple species of pions, the RMS masses are used (where available)

Collaboration	Refs.	$N_{ f}$	*L* [fm]	${M_{\pi ,\mathrm{min}}}L$	Description
FNAL/MILC 14A	[14]	$2+1+1$	2.38–4.83, 2.90–5.82, 2.95–5.62, 2.94–5.44	7.6, 7, 4.9, 3.9	3 values of *L* (2.9, 3.9 and 4.9 fm) at $m_\pi =220$ MeV and $a=0.12$ fm
ETM 13F ETM 14E	[27, 230]	$2+1+1$	2.13/2.84, 1.96/2.61, 2.97	3.5, 3.2, 3.2	The comparison of two different volumes at the two largest lattice spacings indicates that FV effects are below the statistical errors
FNAL/MILC 12B FNAL/MILC 13	[420, 421]	$2+1+1$	2.4/4.8, 2.88/5.76, 2.88/5.76, 2.88/5.76	7.6, 7, 4.9, 3.9	FV errors estimated in $\chi$PT at NLO and, in FNAL/MILC 12B, by analyzing otherwise identical ensembles with three different spatial sizes at $a=0.12$ fm and $m_l/m_s=0.1$

**Table 110:** Finite-volume effects in $N_f=2+1$ determinations of the *D*- and $D_s$-meson-decay constants. Each *L*-entry corresponds to a different lattice spacing, with multiple spatial volumes at some lattice spacings. For actions with multiple species of pions, the RMS masses are used (where available)

Collaboration	Refs.	$N_{ f}$	*L* [fm]	${M_{\pi ,\mathrm{min}}}L$	Description
$\chi$QCD 14	[17]	$2+1$	2.7, 2.7	4.6, 4.1	No explicit discussion of FV effects
HPQCD 12A	[47]	$2+1$	2.4/2.8, 2.4/3.4	6.7, 4.2	FV errors estimated by comparing finite- and infinite-volume $\chi$PT
FNAL/MILC 11	[48]	$2+1$	2.4, 2.4/2.88, 2.52/3.6	6.9, 6.4, 5.8	FV errors estimated using finite-volume $\chi$PT
PACS-CS 11	[422]	$2+1$	2.88	2.2 (before reweighting)	No discussion of FV effects
HPQCD 10A	[49]	$2+1$	2.4, 2.4/2.88/3.36, 2.52, 2.88, 2.82	6.6, 6.7, 4.2, 3.8, 4.8	FV errors estimated using finite- vs. infinite-volume $\chi$PT
HPQCD/UKQCD 07	[28]	$2+1$	2.4, 2.4/2.88, 2.52	6.6, 6.7, 4.2	FV errors estimated using finite- vs infinite-volume $\chi$PT
FNAL/MILC 05	[423]	$2+1$	2.8, 2.9, 2.5	${>}6, 6.4,$ 5	FV errors estimated to be $1.5\%$ or less from $\chi$PT

**Table 111:** Finite-volume effects in $N_f=2$ determinations of the *D*- and $D_s$-meson-decay constants. Each *L*-entry corresponds to a different lattice spacing, with multiple spatial volumes at some lattice spacings. For actions with multiple species of pions, the RMS masses are used (where available)

Collaboration	Refs.	$N_{ f}$	*L* [fm]	${M_{\pi ,\mathrm{min}}}L$	Description
TWQCD 14	[424]	2	1.5	1.92	No explicit discussion of FV effects
ALPHA 13B	[177]	2	2.1/3.1/4.2, 2.3/3.1	4, 4.2	No explicit discussion of FV effects, but $m_\pi L>4$ always
ETM 09 ETM 11A ETM 13B	[20, 32, 182]	2	2.4, 2.0/2.7, 2.1, 2.6	5, 3.7, 3.3, 3.5	FV errors are found to be negligible by comparing results at $m_\pi L = 3.3$ and $m_\pi L=4.3$ for $m_\pi \simeq 310$ MeV

**Table 112:** Lattice spacings and description of actions used in $N_f=2+1+1$ determinations of the *D*- and $D_s$-meson-decay constants

Collaboration	Refs.	$N_{ f}$	*a* [fm]	Continuum extrapolation	Scale setting
FNAL/MILC 14A	[14]	$2+1+1$	0.15, 0.12, 0.09, 0.06	Interpolations around the physical light masses used to fix the ratio of quark masses. Subsequent chiral and continuum extrapolations for the charm decay constants performed simultaneously using different NLO HMrAS$\chi$PT fits	Relative scale through $F_{4ps}$, the decay constant at valence masses $=$ 0.4 $m_s$ and physical sea-quark masses. Absolute scale set through $f_\pi$; the uncertainty is propagated into the final error
ETM 13F ETM 14E	[27, 230]	$2+1+1$	0.09, 0.08, 0.06	Chiral and continuum extrapolations performed simultaneously by adding an $\mathcal {O}(a^2)$ term to the chiral fits	Relative scale set through $M_{c's'}$, the mass of a fictitious meson made of valence quarks of mass $r_0m_{s'}\!=0.22$ and $r_0m_{c'}=2.4$. Absolute scale through $f_\pi$
FNAL/MILC 12B FNAL/MILC 13	[420, 421]	$2+1+1$	0.15, 0.12, 0.09, 0.06	Chiral and continuum extrapolations performed simultaneously. Central values produced using a fit function quadratic in $a^2$ and linear in the sea-quark mass. In FNAL/MILC 13 terms of $\mathcal {O}(a^4)$ are included	Absolute scale set through $f_\pi$; the uncertainty is propagated into the final error

**Table 113:** Lattice spacings and description of actions used in $N_f=2+1$ determinations of the *D*- and $D_s$-meson-decay constants

Collaboration	Refs.	$N_{ f}$	*a* [fm]	Continuum extrapolation	Scale setting
$\chi$QCD 14	[17]	$2+1$	0.113, 0.085	Chiral and continuum extrapolations performed in global fits including linear terms in $m_l$ and terms up to $\mathcal {O}(a^2)$ and $\mathcal {O}(a^4m_c^4)$	Relative scale set through $r_0$, fixed together with the charm- and strange-quark masses using $m_{D_s}$, $m_{D_s^*}$ and $m_{J/\psi }$ as inputs
HPQCD 12A	[47]	$2+1$	0.12, 0.09	Chiral and continuum extrapolations performed simultaneously using PQHM$\chi$PT augmented by *a* dependent terms: $c_0(am_c)^2 + c_1(am_c)^4$	Relative scale set through $r_1$; absolute scale from $f_\pi$, $f_\mathrm{K}$ and the $\Upsilon$ splitting. Uncertainties from both $r_1$ and $r_1/a$ propagated
FNAL/MILC 11	[48]	$2+1$	0.15, 0.12, 0.09	Chiral and continuum extrapolations performed simultaneously using one-loop HM$\chi$PT for rooted staggered quarks. Effects of hyperfine and flavour splittings are also included	Relative scale set through $r_1=0.3117(22)$. The error in $r_1$ comes from the spread of different absolute scale determinations using $f_\pi$, $f_\mathrm{K}$ and the $\Upsilon$ splitting
PACS-CS 11	[422]	$2+1$	0.09	Cutoff effects from the heavy-quark action estimated by naive power counting to be at the percent level	Scale set through $m_\Omega$
HPQCD 10A	[49]	$2+1$	0.15, 0.12, 0.09, 0.06, 0.044	Chiral and continuum extrapolations performed simultaneously. Polynomials up to $am_{c}^8$ are kept (even powers only)	See the discussion for HPQCD 12A
HPQCD/UKQCD 07	[28]	$2+1$	0.15, 0.12, 0.09	Combined chiral and continuum extrapolations using HM$\chi$PT at NLO augmented by second and third-order polynomial terms in $m_q$ and terms up to $a^4$	Scale set through $r_1$ obtained from the $\Upsilon$ spectrum using the non-relativistic QCD action for *b* quarks. Uncertainty propagated among the systematics
FNAL/MILC 05	[423]	$2+1$	0.175, 0.121, 0.086	Most light-quark cutoff effects are removed through NLO HM$\chi$PT for rooted staggered quarks. Continuum values are then obtained by averaging the $a \approx 0.12$ and $a \approx 0.09$ fm results	Scale set through $r_1$ obtained from the $\Upsilon$ spectrum using the non-relativistic QCD action for *b* quarks

**Table 114:** Lattice spacings and description of actions used in $N_f=2$ determinations of the *D*- and $D_s$-meson-decay constants

Collaboration	Refs.	$N_{ f}$	*a* [fm]	Continuum extrapolation	Scale setting
TWQCD 14	[424]	2	0.061	Uncertainties associated to scale setting and discretization effects estimated by performing the chiral fits once in physical and once in lattice units (${\approx } 2$ MeV on $f_{ D_\mathrm{s}}$)	Scale set through the Wilson flow and $r_0$ set to 0.49 fm
ALPHA 13B	[177]	2	0.065, 0.048	Linear fits (in $m_\pi ^2$ and in $a^2$) and partially quenched HM$\chi$PT functional forms, including terms linear in $a^2$, are used in the combined chiral/continuum extrapolation	Scale set through $f_K$
ETM 09 ETM 11A ETM 13B	[20, 32, 182]	2	0.10, 0.085, 0.065, 0.054	NLO *SU*(2) HM$\chi$PT supplemented by terms linear in $a^2$ and in $m_D a^2$ is used in the combined chiral/continuum extrapolation	Scale set through $f_\pi$

**Table 115:** Operator renormalization in determinations of the *D*- and $D_s$-meson-decay constants

Collaboration	Refs.	$N_{ f}$	Ren.	Description
FNAL/MILC 14A	[14]	$2+1+1$	−	The axial current is absolutely normalized
ETM 13F, 14E	[27, 230]	$2+1+1$	−	The axial current is absolutely normalized
FNAL/MILC 12B, 13	[420, 421]	$2+1+1$	−	The axial current is absolutely normalized
$\chi$QCD 14	[17]	$2+1$	RI	The decay constant is extracted from an exact lattice Ward identity and from the NP renormalized axial current
HPQCD 12A	[47]	$2+1$	−	The axial current is absolutely normalized
FNAL/MILC 11	[48]	$2+1$	mNPR	Two-loop and higher-order perturbative truncation errors estimated to be the full size of the one-loop term
PACS-CS 11	[422]	$2+1$	PT1$\ell$ $+$ NP	Mass dependent part of the renormalization constant of the axial current computed at one-loop; the NP contribution is added in the chiral limit
HPQCD 10A	[49]	$2+1$	−	The axial current is absolutely normalized
HPQCD/UKQCD 07	[28]	$2+1$	−	The axial current is absolutely normalized
FNAL/MILC 05	[423]	$2+1$	mNPR	Errors due to higher-order corrections in the perturbative part are estimated to be $1.3\%$
TWQCD 14	[424]	2	−	The decay constant is extracted from an exact lattice Ward identity
ALPHA 13B	[177]	2	SF	NP renormalization and improvement of the axial current (*am* terms included at one-loop)
ETM 09, 11A, 13B	[20, 32, 182]	2	−	The axial current is absolutely normalized

**Table 116:** Heavy-quark treatment in $N_f=2+1+1$ determinations of the *D*- and $D_s$-meson-decay constants

Collaboration	Refs.	$N_{ f}$	Action	Description
FNAL/MILC 14A	[14]	$2+1+1$	HISQ (on HISQ)	$0.22<am_c<0.84$. Discretization errors estimated to be $\approx$1 MeV using the spread of 108 different chiral/continuum fits (for example by including or not some NNLO discretization effects in HMrAS$\chi$PT)
ETM 13F, 14E	[27, 230]	$2+1+1$	tmWil	$0.15 \,\mathop {\sim }\limits ^{{<}}\,am_c \,\mathop {\sim }\limits ^{{<}}\,0.20$
FNAL/MILC 12B FNAL/MILC 13	[420, 421]	$2+1+1$	HISQ (on HISQ)	$0.29<am_c<0.7$. Discretization errors estimated using different fit ansätze to be ${\approx } 1.5\%$ for $f_{D_{(s)}}$

**Table 117:** Heavy-quark treatment in $N_f=2+1$ determinations of the *D*- and $D_s$-meson-decay constants

Collaboration	Refs.	$N_{ f}$	Action	Description
$$\chi $$QCD 14	[17]	$$2+1$$	Overlap on DW	$$0.29<am_c<0.75$$. Heavy-quark discretization errors estimated by including $$(am_c)^2$$ and $$am_c)^4$$ terms in the chiral/continuum extrapolation
HPQCD 12A	[47]	$2+1$	HISQ	$0.41<am_c<0.62$. Heavy-quark discretization errors estimated using different fit ansätze to be ${\approx }1.2\%$
FNAL/MILC 11	[48]	$2+1$	Fermilab	Discretization errors from charm quark estimated through a combination of Heavy Quark and Symanzik Effective Theories to be around 3% for $f_{D_{(s)}}$ and negligible for the ratio
PACS-CS 11	[422]	$2+1$	Tsukuba	$am_c\approx 0.57$. Heavy-quark discretization errors estimated to be at the percent level by power counting
HPQCD 10A	[49]	$2+1$	HISQ	$0.193< am_c<0.825$. Heavy-quark discretization errors estimated by changing the fit-inputs to be ${\approx }0.4\%$
HPQCD/UKQCD 07	[28]	$2+1$	HISQ	$0.43<am_c<0.85$. Heavy-quark discretization errors estimated from the chiral/continuum fits to be ${\approx }0.5\%$
FNAL/MILC 05	[423]	$2+1$	Fermilab	Discretization errors from charm quark estimated via heavy-quark power counting at 4.2% for $f_{D_{(s)}}$ and 0.5% for the ratio

**Table 118:** Heavy-quark treatment in $N_f=2$ determinations of the *D*- and $D_s$-meson-decay constants

Collaboration	Refs.	$N_{ f}$	Action	Description
TWQCD 14	[424]	2	DW	$am_c\le 0.55$. Optimal Domain Wall fermions [794] preserving chiral symmetry
ALPHA 13B	[177]	2	npSW	$am_c\le 0.28$. Axial current nonperturbatively improved ($\mathcal {O}(am)$ at one-loop)
ETM 09, 11A, 13B	[20, 32, 182]	2	tmWil	$0.16<am_c<0.23$. $D(a_\mathrm{min})\approx 5\%$ in ETM 09

#### B.5.2 $D\rightarrow \pi \ell \nu$ and $D\rightarrow K\ell \nu$ form factors

See Tables 119, 120, 121, 122 and 123.

**Table 119:** Continuum extrapolations/estimation of lattice artefacts in determinations of the $D\rightarrow \pi \ell \nu$ and $D\rightarrow K \ell \nu$ form factors

Collaboration	Refs.	$N_{ f}$	*a* [fm]	Continuum extrapolation	Scale setting
HPQCD 10B, 11	[50, 51]	$2+1$	0.09, 0.12	Modified *z*-expansion fit combining the continuum and chiral extrapolations and the momentum transfer dependence. Leading discretization errors from $(am_c)^n$ charm-mass effects (see Table 123). Subleading $(aE)^n$ discretization corrections estimated to be 1.0% for both $D\rightarrow \pi$ and $D\rightarrow K$	Relative scale $r_1/a$ set from the static-quark potential. Absolute scale $r_1$ set from several quantities including $f_\pi$, $f_K$, and $\Upsilon$ 2*S*–1*S* splitting cf. HPQCD 09B [250]. Scale uncertainty estimated to be 0.7% in $D\rightarrow \pi$ and 0.2% in $D\rightarrow K$
FNAL/MILC 04	[441]	$2+1$	0.12	Discretization effects from light-quark sector estimated to be 4% by power counting. Discretization effects from final-state pion and kaon energies estimated to be 5%	Scale set through $\Upsilon$ 2*S*–1*S* splitting cf. HPQCD 03 [795]. Error in $a^{-1}$ estimated to be 1.2%, but scale error in dimensionless form factor negligible compared to other uncertainties
ETM 11B	[431]	2	0.068, 0.086, 0.102	Discretization errors estimated to be 5% for $D\rightarrow \pi$ and 3% for $D\rightarrow K$ from comparison of results in the continuum limit to those at the finest lattice spacing	Scale set through $f_\pi$ cf. ETM 07A [83] and ETM 09C [36]

**Table 120:** Chiral-extrapolation/minimum pion mass in determinations of the $D\rightarrow \pi \ell \nu$ and $D\rightarrow K \ell \nu$ form factors. For actions with multiple species of pions, masses quoted are the RMS pion masses. The different $M_{\pi ,\mathrm min}$ entries correspond to the different lattice spacings

Collaboration	Refs.	$N_{ f}$	$M_{\pi ,\mathrm{min}}\,[\mathrm{MeV}]$	Description
HPQCD 10B, 11	[50, 51]	$2+1$	390, 390	Modified *z*-expansion fit combining the continuum and chiral extrapolations and the momentum transfer dependence. Contributions to error budget from light valence and sea-quark mass dependence estimated to be 2.0% for $D \rightarrow \pi$ and 1.0% for $D \rightarrow K$
FNAL/MILC 04	[441]	$2+1$	510	Fit to S$\chi$PT, combined with the Becirevic–Kaidalov ansatz for the momentum transfer dependence of form factors. Error estimated to be 3% for $D \rightarrow \pi$ and 2% for $D \rightarrow K$ by comparing fits with and without one extra analytic term
ETM 11B	[431]	2	270	*SU*(2) tmHM$\chi$PT plus Becirevic–Kaidalov ansatz for fits to the momentum transfer dependence of form factors. Fit uncertainty estimated to be 7% for $D \rightarrow \pi$ and 5% for $D \rightarrow K$ by considering fits with and without NNLO corrections of order $\mathcal {O}(m_\pi ^4)$ and/or higher-order terms through $E^5$, and by excluding data with $E \gtrsim 1$ GeV

**Table 121:** Finite-volume effects in determinations of the $D\rightarrow \pi \ell \nu$ and $D\rightarrow K\ell \nu$ form factors. Each *L*-entry corresponds to a different lattice spacing, with multiple spatial volumes at some lattice spacings. For actions with multiple species of pions, the lightest pion masses are quoted

Collaboration	Refs.	$N_{ f}$	*L* [fm]	${M_{\pi ,\mathrm{min}}}L$	Description
HPQCD 10B, 11	[50, 51]	$2+1$	2.4, 2.4/2.9	${\gtrsim } 3.8$	Finite-volume effects estimated to be 0.04% for $D\rightarrow \pi$ and 0.01% for $D\rightarrow K$ by comparing the “$m_\pi ^2 \mathrm{log} (m_\pi ^2)$” term in infinite and finite volume
FNAL/MILC 04	[441]	$2+1$	2.4/2.9	${\gtrsim } 3.8$	No explicit estimate of FV error, but expected to be small for simulation masses and volumes
ETM 11B	[431]	2	2.2, 2.1/2.8, 2.4	${\gtrsim } 3.7$	Finite-volume uncertainty estimated to be at most 2% by considering fits with and without the lightest pion-mass point at $m_\pi L \approx 3.7$

**Table 122:** Operator renormalization in determinations of the $D\rightarrow \pi \ell \nu$ and $D\rightarrow K \ell \nu$ form factors

Collaboration	Refs.	$N_{ f}$	Ren.	Description
HPQCD 10B, 11	[50, 51]	$2+1$	–	Form factor extracted from absolutely normalized scalar-current matrix element then using kinematic constraint at zero momentum-transfer $f_+(0) = f_0(0)$
FNAL/MILC 04	[441]	$2+1$	mNPR	Size of two-loop correction to current renormalization factor assumed to be negligible
ETM 11B	[431]	2	–	Form factors extracted from double ratios insensitive to current normalization

**Table 123:** Heavy-quark treatment in determinations of the $D\rightarrow \pi \ell \nu$ and $D\rightarrow K \ell \nu$ form factors

Collaboration	Refs.	$N_{ f}$	Action	Description
HPQCD 10B, 11	[50, 51]	$2+1$	HISQ	Bare charm-quark mass $am_c \sim 0.41$–0.63. Errors of $(am_c)^n$ estimated within modified *z*-expansion to be 1.4% for $D\rightarrow K$ and 2.0% for $D \rightarrow \pi$. Consistent with expected size of dominant one-loop cutoff effects on the finest lattice spacing, $\mathcal {O}(\alpha _S (am_c)^2 (v/c) )\sim 1.6\%$
FNAL/MILC 04	[441]	$2+1$	Fermilab	Discretization errors from charm quark estimated via heavy-quark power counting to be 7%
ETM 11B	[431]	2	tmWil	Bare charm-quark mass $am_c \sim 0.17$–0.30. Expected size of $\mathcal {O}((am_c)^2)$ cutoff effects on the finest lattice spacing consistent with quoted 5% continuum-extrapolation uncertainty

### B.6 Notes to Sect. 8 on *B*-meson-decay constants and mixing parameters

In the following, we summarize the characteristics (lattice actions, pion masses, lattice spacings, etc.) of the recent $N_{ f}= 2+1+1$, $N_{ f}=2+1$ and $N_{ f}= 2$ runs. We also provide brief descriptions of how systematic errors are estimated by the various authors. We focus on calculations with either preliminary or published quantitative results.

#### B.6.1 $B_{(s)}$-meson-decay constants

See Tables 124, 125, 126, 127, 128, 129, 130, 131, 132, 133, 134 and 135.

**Table 124:** Chiral-extrapolation/minimum pion mass in determinations of the *B*- and $B_s$-meson-decay constants for $N_f=2+1+1$ simulations. For actions with multiple species of pions, masses quoted are the RMS pion masses (where available). The different $M_{\pi ,\mathrm{min}}$ entries correspond to the different lattice spacings

Collaboration	Refs.	$N_{ f}$	$M_{\pi ,\mathrm{min}}\,[\mathrm{MeV}]$	Description
ETM 13E	[456]	$2+1+1$	245, 239, 211	$M_{\pi ,\mathrm{min}}$ refers to the charged pions. Linear and NLO (full QCD) HM$\chi$PT supplemented by an $a^2$ term is used for the *SU*(3) breaking ratios. The chiral fit error is estimated from the difference between the NLO HM$\chi$PT and linear fits with half the difference used as estimate of the systematic error. The ratio $z_s$ is fit using just linear HM$\chi$PT supplemented by an $a^2$ term
HPQCD 13	[52]	$2+1+1$	310, 294, 173	Two or three pion masses at each lattice spacing, one each with a physical mass GB pion. NLO (full QCD) HM$\chi$PT supplemented by generic $a^2$ and $a^4$ terms is used to interpolate to the physical-pion mass

**Table 125:** Chiral-extrapolation/minimum pion mass in determinations of the *B*- and $B_s$-meson-decay constants for $N_f=2+1$ simulations. For actions with multiple species of pions, masses quoted are the RMS pion masses (where available). The different $M_{\pi ,\mathrm{min}}$ entries correspond to the different lattice spacings

Collaboration	Refs.	$N_{ f}$	$M_{\pi ,\mathrm{min}}\,[\mathrm{MeV}]$	Description
RBC/UKQCD 14RBC/UKQCD 13A	[53][457]	$2+1$	329, 289	Two or three light-quark masses per lattice spacing. In RBC/UKQCD 14, three to four light valence-quark masses that are heavier than the sea-quark masses are also employed to have partially quenched points. NLO *SU*(2) HM$\chi$PT is used. In RBC/UKQCD 14, the fit with only the unitary points is the central analysis procedure, and the systematic errors in the combined chiral-continuum extrapolation are estimated to be from 3.1 to $5.9\%$ in the decay constants and the *SU*(3) breaking ratios
RBC/UKQCD 14A	[54]	$2+1$	327, 289	Two or three light-quark masses per lattice spacing. NLO *SU*(2) HM$\chi$PT is used in the combined chiral-continuum extrapolation. The systematic errors in this extrapolation are estimated to be $3.54\%$ for $f_{B}$, $1.98\%$ for $f_{B_{s}}$, and $2.66\%$ for $f_{B_{s}}/f_{B}$
HPQCD 12	[55]	$2+1$	390, 390	Two or three pion masses at each lattice spacing. NLO (full QCD) HM$\chi$PT supplemented by NNLO analytic terms and generic $a^2$ and $a^4$ terms is used. The systematic error is estimated by varying the fit Ansatz, in particular for the NNLO analytic terms and the $a^{2n}$ terms
HPQCD 11A	[56]	$2+1$	570, 450, 390, 330, 330	One light sea-quark mass only at each lattice spacing. The sea-quark mass dependence is assumed to be negligible, based on the calculation of $f_{D_s}$ in Ref. [49], where the sea-quark extrapolation error is estimated as $0.34\%$
FNAL/MILC 11	[48]	$2+1$	570, 440, 320	Three to five sea-quark masses per lattice spacing, and 9–12 valence light-quark masses per ensemble. NLO partially quenched HMrS$\chi$PT including 1 / *m* terms and supplemented by NNLO analytic and $\alpha _s^2a^2$ terms is used. The systematic error is estimated by varying the fit Ansatz, in particular the NNLO analytic terms and the chiral scale
RBC/UKQCD 10C	[464]	$2+1$	430	Three light-quark masses at one lattice spacing. NLO *SU*(2) $\chi$PT is used. The systematic error is estimated from the difference between NLO $\chi$PT and linear fits as ${\sim } 7\%$
HPQCD 09	[59]	$2+1$	440, 400	Four or two pion masses per lattice spacing. NLO (full QCD) HMrS$\chi$PT supplemented by NNLO analytic terms and $\alpha _s a^2, a^4$ terms is used. The chiral fit error is estimated by varying the fit Ansatz, in particular, by adding or removing NNLO and discretization terms

**Table 126:** Chiral-extrapolation/minimum pion mass in determinations of the *B*- and $B_s$-meson-decay constants for $N_f=2$ simulations. For actions with multiple species of pions, masses quoted are the RMS pion masses (where available). The different $M_{\pi ,\mathrm{min}}$ entries correspond to the different lattice spacings

Collaboration	Refs.	$N_{ f}$	$M_{\pi ,\mathrm{min}}\,[\mathrm{MeV}]$	Description
ALPHA 14 ALPHA 13ALPHA 12A	[57][458][459]	2	280, 190, 270	LO and NLO HM$\chi$PT supplemented by a term linear in $a^2$ are used. In ALPHA 13 and ALPHA 12A, the final result is an average between LO and NLO with half the difference used as estimate of the systematic error. In ALPHA 14, the NLO fit is used as the central analysis procedure, and the LO results are used to estimate the systematic errors ($0.9\%$ MeV for $f_{B_{s}}$, $1.1\%$ for $f_{B}$, and $1.6\%$ for $f_{B_{s}}/f_{B}$)
ETM 13B, 13CETM 12BETM 11A	[20, 58][460][182]	2	410, 275, 300, 270	$M_{\pi ,\mathrm{min}}$ refers to the charged pions. Linear and NLO (full QCD) HM$\chi$PT supplemented by an $a^2$ term is used.The chiral fit error is estimated from the difference between the NLO HM$\chi$PT and linear fits with half the difference used as estimate of the systematic error. For the static-limit calculation in ETM 11A, $\Phi _s^\mathrm{stat}$ is extrapolated assuming a constant in light-quark mass. The ratio $\Phi _s^\mathrm{stat}/\Phi _{\ell }^\mathrm{stat}$ is fit using three different chiral fit forms (NLO HM$\chi$PT, linear, and quadratic) to estimate the chiral fir error
ALPHA 11	[461]	2	331, 268, 267	Linear and NLO (full QCD) HM$\chi$PT supplemented by a term linear in $a^2$ are used. The final result is an average between linear and NLO fits with half the difference used as estimate of the systematic error
ETM 09D	[462]	2	410, 275, 300	$M_{\pi ,\mathrm{min}}$ refers to the charged pions. Linear and NLO (full QCD) HM$\chi$PT is used. The final result given by the average of NLO HMChiPT and linear *Ansätze* ± half the difference)

**Table 127:** Finite-volume effects in determinations of the *B*- and $B_s$-meson-decay constants. Each *L*-entry corresponds to a different lattice spacing, with multiple spatial volumes at some lattice spacings

Collaboration	Refs.	$N_{ f}$	*L* [fm]	${M_{\pi ,\mathrm{min}}}L$	Description
ETM 13E	[456]	$2+1+1$	2.84/2.13, 2.61/1.96, 2.97	3.5, 3.2, 3.2	FV error estimated how?
HPQCD 13	[52]	$2+1+1$	2.4/3.5/4.7, 2.9/3.8/5.8, 2.8/5.6	7.4, 8.6, 4.9	The analysis uses finite-volume $\chi$PT
RBC/UKQCD 14RBC/UKQCD 13A	[53][457]	$2+1$	2.64, 2.75	4.4, 4.0	In RBC/UKQCD 14, finite-volume effects are estimated to be negligible for $f_{B_{s}}$, $0.4\%$ for $f_{B^{0}}$, $0.5\%$ for $f_{B^{+}}$ and the *SU*(3) breaking ratios
RBC/UKQCD 14A	[54]	$2+1$	2.74, 2.76	4.5, 4.0	Finite-volume effects are estimated to be negligible for $f_{B_{s}}$, $0.82\%$ for $f_{B}$, and $1\%$ for $f_{B_{s}}/f_{B}$
HPQCD 12	[55]	$2+1$	2.4/2.9, 2.5/3.6	5.7, 7.1	FV error is taken from Ref. [28] for HPQCD’s *D* meson analysis, where it was estimated using finite-volume $\chi$PT
HPQCD 11A	[56]	$2+1$	2.4, 2.4, 2.5, 2.9, 2.9	6.9, 5.5, 4.9, 4.8, 4.8	FV error is assumed to negligible
FNAL/MILC 11	[48]	$2+1$	2.4, 2.4/2.9, 2.5/3.6	6.9, 6.4, 5.8	FV error is estimated using finite-volume $\chi$PT
RBC/UKQCD 10C	[464]	$2+1$	1.8	3.9	FV error estimated using finite-volume $\chi$PT to be $1\%$ for *SU*(3) breaking ratios
HPQCD 09	[59]	$2+1$	2.4/2.9, 2.5	6.5, 5.1	FV error is assumed to negligible
ALPHA 14 ALPHA 13 ALPHA 12A ALPHA 11	[57] [458] [459] [461]	2	2.4/3.6, 2.1/3.1/4.2, 2.3/3.1	5.2, 4.1, 4.2	No explicit estimate of FV errors, but expected to be much smaller than other uncertainties
ETM 13B, 13CETM 12BETM 11A	[20, 58] [460] [182]	2	2.4, 2.0/2.7, 2.1, 1.7/2.6	5.0, 3.7, 3.3, 3.5	FV errors are found to be negligible by comparing results at $m_\pi L = 3.3$ and $m_\pi L=4.3$ for $m_\pi \simeq 310$ MeV

**Table 128:** Continuum extrapolations/estimation of lattice artefacts in determinations of the *B* and $B_s$ meson decay constants for $N_f=2+1+1$ simulations

Collaboration	Refs.	$N_{ f}$	*a* [fm]	Continuum extrapolation	Scale setting
ETM 13E	[456]	$2+1+1$	0.89, 0.82, 0.62	Combined continuum and chiral extrapolation, linear in $a^2$	Scale set from $f_\pi$. Scale setting uncertainty included in combined statistical and systematic error
HPQCD 13	[52]	$2+1+1$	0.15, 0.12, 0.09	Combined continuum and chiral extrapolation. Continuum extrapolation errors estimated to be $0.7\%$	Scale set from $\Upsilon$(2S-1S) splitting; see Ref. [757]. Scale uncertainty included in statistical error

**Table 129:** Continuum extrapolations/estimation of lattice artefacts in determinations of the *B* and $B_s$ meson decay constants for $N_f=2+1$ simulations

Collaboration	Refs.	$N_{ f}$	*a* [fm]	Continuum extrapolation	Scale setting
RBC/UKQCD 14 RBC/UKQCD 13A	[53] [457]	$2+1$	0.11, 0.086	Combined continuum and chiral extrapolation with linear in $a^2$ term. In RBC/UKQCD 14, the systematic errors from this procedure are estimated to be from 3.1 to $5.9\%$ in the decay constants and the *SU*(3)-breaking ratios	Scale set by the $\Omega$ baryon mass. In RBC/UKQCD 14, scale uncertainty estimated to be $1.5\%$ in the decay constants, and $0.1\%$ in the *SU*(3)-breaking ratios
RBC/UKQCD 14A	[54]	2+1	0.11, 0.086	Chiral-continuum extrapolation with linear in $a^2$ term is employed, with the systematic errors estimated to be from 1.98 to $3.54\%$ in the decay constants and $f_{B_{s}}/f_{B}$. Discretization errors at $\mathcal {O}(\alpha _{s} a)$ in the static-light system are estimated to be $1\%$ in the decay constants, and $0.2\%$ in $f_{B_{s}}/f_{B}$	Scale set by the $\Omega$ baryon mass
HPQCD 12	[55]	$2+1$	0.12, 0.09	Combined continuum and chiral extrapolation. Continuum extrapolation errors estimated to be $0.9\%$	Relative scale $r_1/a$ from the static-quark potential. Absolute scale $r_1$ from $f_\pi$, $f_K$, and $\Upsilon$(2S-1S) splitting. Scale uncertainty estimated to be $1.1\%$
HPQCD 11A	[56]	$2+1$	0.15, 0.12, 0.09, 0.06, 0.045	$am_Q \approx 0.2$–0.85. Combined continuum and HQET fit. Continuum extrapolation error estimated by varying the fit ansatz and the included data points to be $0.63\%$. Discretization errors appear to decrease with increasing heavy-meson mass	Relative scale $r_1/a$ from the static-quark potential. Absolute scale $r_1$ from $f_\pi$, $f_K$, and $\Upsilon$(2S-1S) splitting. Scale uncertainty estimated to be $0.74\%$
FNAL/MILC 11	[48]	$2+1$	0.15, 0.12, 0.09	Combined continuum and chiral extrapolation. Continuum extrapolation errors estimated to be 1.3%	Relative scale $r_1/a$ from the static-quark potential. Absolute scale $r_1$ from $f_\pi$, $f_K$, and $\Upsilon$(2S-1S) splitting. Scale uncertainty estimated to be 1 MeV
RBC/UKQCD 10C	[464]	$2+1$	0.11	One lattice spacing with discretization errors estimated by power counting as 3%	Scale set by the $\Omega$ baryon mass. Combined scale and mass tuning uncertainties on $f_{B_s}/f_B$ estimated as 1%
HPQCD 09	[59]	$2+1$	0.12, 0.09	Combined continuum and chiral extrapolation. Continuum extrapolation errors estimated to be 3%	Relative scale $r_1/a$ from the static-quark potential. Absolute scale $r_1$ from the $\Upsilon$(2S-1S) splitting. Scale uncertainty estimated to be $2.3\%$

**Table 130:** Continuum extrapolations/estimation of lattice artefacts in determinations of the *B*- and $B_s$-meson-decay constants for $N_f=2$ simulations

Collaboration	Refs.	$N_{ f}$	*a* [fm]	Continuum extrapolation	Scale setting
ALPHA 14 ALPHA 13 ALPHA 12A ALPHA 11	[57] [458] [459] [461]	2	0.075, 0.065, 0.048	Combined continuum and chiral extrapolation with linear in $a^2$ term. Continuum extrapolation errors estimated to be 5 MeV in ALPHA 11. The continuum extrapolation with a term linear in *a* also investigated in ALPHA 14, and within the statistical error no discernable difference was observed	Relative scale set from $r_0$. Absolute scale set from $f_K$. Scale setting uncertainty included in combined statistical and extrapolation error
ETM 13B, 13C, ETM 12B ETM 11A	[20, 58] [460] [182]	2	0.098, 0.085, 0.067, 0.054	Combined continuum and chiral extrapolation, with a term linear in $a^2$. ETM 12 and 13 include a heavier masses than ETM 11A. Discretization error included in combined statistical and systematic error, estimated by dropping the data at the coarsest lattice spacing as ${\sim } 0.5$–$1\%$	Scale set from $f_\pi$. Scale setting uncertainty included in combined statistical and systematic error
ETM 09D	[462]	2	0.098, 0.085, 0.067	Combined continuum and chiral extrapolation with a term linear in $a^2$	Scale set from $f_\pi$. Scale setting uncertainty included in combined statistical and systematic error

**Table 131:** Description of the renormalization/matching procedure adopted in the determinations of the *B*- and $B_s$-meson-decay constants for $N_{f} = 2 +1+1$ simulations

Collaboration	Refs.	$N_{ f}$	Ren.	Description
ETM 13E	[456]	$2+1+1$	–, PT1$\ell$	The current used for the relativistic decay constants is absolutely normalized. The ratio is constructed from the relativistic decay constant data and the heavy-quark pole masses. Ratios of pole-to-${\overline{\text {MS}}}$ mass conversion factors are included at NLO in continuum perturbation theory
HPQCD 13	[52]	$2+1+1$	PT1$\ell$	The NRQD effective current is matched through $\mathcal {O}(1/m)$ and renormalized using one-loop PT. Included are all terms though $\mathcal {O}(\alpha _s)$, $\mathcal {O}(\alpha _s \, a)$, $\mathcal {O}(\Lambda _\mathrm{QCD}/M)$, $\mathcal {O}(\alpha _s/a M)$, $\mathcal {O}(\alpha _s \, \Lambda _\mathrm{QCD}/M)$. The dominant error is due unknown $\mathcal {O}(\alpha _s^2)$ contributions to the current renormalization. The perturbation theory used in this work is the same as in HPQCD 09 and 12, but is rearranged to match the mNPR method. Using the fact that the heavy-heavy temporal vector current is normalized, and that the light-light HISQ vector current receives a small one-loop correction, the error is estimated as ${\sim }1.4$%

**Table 132:** Description of the renormalization/matching procedure adopted in the determinations of the *B*- and $B_s$-meson-decay constants for $N_{f} = 2+1$ simulations

Collaboration	Refs.	$N_{ f}$	Ren.	Description
RBC/UKQCD 14RBC/UKQCD 13A	[53] [457]	$2+1$	mNPR	In RBC/UKQCD 14, the error is dominated by the perturbative aspect, and is estimated to be $1.7\%$ for the decay constants by taking the full size of the one-loop correction for the fine lattice
RBC/UKQCD 14A	[54]	$2+1$	PT1$\ell$	A two-step matching procedure is employed, first from QCD to HQET in the continuum at $m_{b}$, then to HQET on the lattice at $a^{-1}$ with $\mathcal {O}(pa)$ and $\mathcal {O}(m_{q} a)$ errors included. Both matching steps are accurate to one-loop, and the running between $m_{b}$ and $a^{-1}$ is performed at two-loop accordingly. The error is estimated using a power-counting argument to be $6\%$ for the decay constants
HPQCD 12/09	[55, 59]	$2+1$	PT1$\ell$	The NRQD effective current is matched through $\mathcal {O}(1/m$) and renormalized using one-loop PT. Included are all terms though $\mathcal {O}(\alpha _s)$, $\mathcal {O}(\alpha _s \, a)$, $\mathcal {O}(\Lambda _\mathrm{QCD}/M)$, $\mathcal {O}(\alpha _s/a M)$, $\mathcal {O}(\alpha _s \, \Lambda _\mathrm{QCD}/M)$. The dominant error is due unknown $\mathcal {O}(\alpha _s^2)$ contributions to the current renormalization. The authors take the perturbative error as $\sim 2 \rho _0 \, \alpha _s^2$, where $\rho _0$ is the coefficient of the one-loop correction to the leading term, which yields an error of ${\sim }4$%
HPQCD 11A	[56]	$2+1$	–	This work uses PCAC together with an absolutely normalized current
FNAL/MILC 11	[48]	$2+1$	mNPR	The authors’ estimate of the perturbative errors is comparable in size to the actual one-loop corrections
RBC/UKQCD 10C	[464]	$2+1$	PT1$\ell$	The static-light current is matched through $\mathcal {O}(\alpha _s a, \alpha _s)$ and renormalized using one-loop tadpole improved PT. For massless light quarks, the renormalization factors cancel in the ratio of decay constants

**Table 133:** Description of the renormalization/matching procedure adopted in the determinations of the *B*- and $B_s$-meson-decay constants for $N_{f} = 2$ simulations

Collaboration	Refs.	$N_{ f}$	Ren.	Description
ALPHA 14	[57]	2	NPR	The authors use the Schrödinger functional for the NP matching
ALPHA 13	[458]			
ALPHA 12A	[459]			
ALPHA 11	[461]			
ETM 13B, 13CETM 12BETM 11A	[20, 58] [460][182]	2	–, PT1$\ell$	The current used for the relativistic decay constants is absolutely normalized. **Interpolation method:** The static-limit current renormalization is calculated in one-loop mean-field-improved perturbation theory, there half the correction is used to estimate the error. **Ratio method:** The ratio is constructed from the relativistic decay constant data and the heavy-quark pole masses. Ratios of pole-to-${\overline{\text {MS}}}$ mass conversion factors are included at NLO in continuum perturbation theory

**Table 134:** Heavy-quark treatment in $N_f=2+1+1$ determinations of the *B*- and $B_s$-meson-decay constants

Collaboration	Refs.	$N_{ f}$	Action	Description
ETM 13E	[456]	$2+1+1$	tmWil	The estimate of the discretization effects is described in the continuum table. The relativistic data are matched to HQET using NLO continuum PT in an intermediate step, and converted back to QCD at the end. The error due to HQET matching (estimated by replacing the NLO expressions with LO) is a very small contribution to the systematic error due to the heavy-quark mass dependence
HPQCD 13	[52]	$2+1+1$	NRQCD	The leading HQ truncation effects are of $\mathcal {O}( \Lambda ^{2}_\mathrm{QCD}/m^{2}_{h})$ and $\mathcal {O}(\alpha ^{2}_{s} \Lambda _\mathrm{QCD}/m_h)$, and the errors are at the subpercentage level

**Table 135:** Heavy-quark treatment in $N_f=2+1$ and $N_f=2$ determinations of the *B*- and $B_s$-meson-decay constants

Collaboration	Refs.	$N_{ f}$	Action	Description
RBC/UKQCD 14RBC/UKQCD 13A	[53][457]	$2+1$	RHQ	In RBC/UKQCD 14, the heavy-quark discretization errors are estimated to be $1.7\%$ in the decay constants, and $0.3\%$ in the *SU*(3) breaking ratios
RBC/UKQCD 14A	[54]	$2+1$	Static	Static-limit computation, with $\mathcal {O}(\Lambda _{\mathrm{QCD}}/m_{h})$ errors estimated to be $10\%$ for the decay constants, and $2.2\%$ for $f_{B_{s}}/f_{B}$
HPQCD 12	[55]	$2+1$	NRQCD	HQ truncation effects estimated as in HPQCD 09 to be $1.0\%$
HPQCD 11A	[56]	$2+1$	HISQ	The analysis uses a combined continuum and 1 / *m* extrapolation
FNAL/MILC 11	[48]	$2+1$	Fermilab	HQ discretization effects are included in the combined chiral and continuum fits, and they are estimated by varying the fit Ansatz and excluding the data at the coarsest lattice spacing to be ${\sim } 2\%$, consistent with simple power-counting estimates but larger than the residual discretization errors observed in the data
RBC/UKQCD 10C	[464]	$2+1$	Static	Truncation effects of $\mathcal {O}(1/m_h)$ on the *SU*(3) breaking ratios are estimated by power counting to be 2%
HPQCD 09	[59]	$2+1$	NRQCD	The leading HQ truncation effects are of $\mathcal {O}(\alpha _s \Lambda _\mathrm{QCD}/m_h)$ due to the tree-level coefficient of the ${\varvec{\sigma }}\cdot \mathrm{\mathbf B}$ term. The error is estimated by calculating the $B^*-B$ hyperfine splitting and comparing with experiment as $1\%$
ALPHA 14ALPHA 13ALPHA 12AALPHA 11	[57][458][459][461]	2	HQET	NP improved through $\mathcal {O}(1/m_h)$. Truncation errors of $\mathcal {O}\left[ (\Lambda _\mathrm{QCD}/m_h)^2 \right]$ are not included
ETM 13B, 13CETM 12BETM 11A	[20, 58] [460][182]	2	tmWil	The estimate of the discretization effects is described in the continuum table. In both methods the relativistic data are matched to HQET using NLO continuum PT in an intermediate step, and converted back to QCD at the end. The error due to HQET matching (estimated by replacing the NLO expressions with LO) is a very small contribution to the systematic error due to the heavy-quark mass dependence. The variation observed from adding heavier masses to their data and/or including $1/m_h^3$ terms is 0.4–1.3%

#### B.6.2 $B_{(s)}$-meson mixing matrix elements

See Tables 136, 137, 138, 139, 140 and 141.

**Table 136:** Continuum extrapolations/estimation of lattice artefacts in determinations of the neutral *B*-meson mixing matrix elements for $N_f=2+1$ simulations

Collaboration	Refs.	$N_{ f}$	*a* [fm]	Continuum extrapolation	Scale setting
RBC/UKQCD 14A	[54]	$2+1$	0.11, 0.086	Combined continuum and chiral extrapolation with *SU*(2) NLO HM$\chi$PT and linear in quark mass both with $\mathcal {O}(a^2)$ terms. The combined continuum and chiral extrapolation uncertainty is estimated as 2.55, 2.13 and 3.08% for $f_B\sqrt{B_B}$, $f_{B_s}\sqrt{B_{B_s}}$ and $\xi$ respectively	Scale is set using the $\Omega ^-$ mass as input [144]. The scale uncertainty is estimated as 1.84, 1.86 and 0.05% for $f_B\sqrt{B_B}$, $f_{B_s}\sqrt{B_{B_s}}$ and $\xi$ respectively
FNAL/MILC 12	[60]	$2+1$	0.12, 0.09	Combined continuum and chiral extrapolation with NLO rHMS$\chi$PT, NNLO analytic and generic $\mathcal {O}(\alpha _s^2 a^2, a^4)$ terms. Combined statistical, chiral and light-quark discretization error is estimated, by examining the variation with different fit Ansätze to be 3.7% on $\xi$	Relative scale $r_1/a$ is set via static-quark potential. Absolute scale $r_1=0.3117(22)$ fm is determined [48] through averaging the $f_\pi$ input and the estimate of HPQCD Collaboration [250]. The scale uncertainty on $\xi$ is estimated as 0.2%
FNAL/MILC 11A	[483]	$2+1$	0.12, 0.09, 0.06	Combined continuum and chiral extrapolation with NLO rHMS$\chi$PT, NNLO analytic and generic $\mathcal {O}(\alpha _s^2 a^2, a^4)$ terms	See above. The error in $r_1$ yields a 3% uncertainty on $f^2_B B_B$
RBC/UKQCD 10C	[464]	$2+1$	0.11	Only one lattice spacing is used. Discretization error is estimated to be 4% on $\xi$ by power counting	Scale is set using the $\Omega ^-$ mass as input [145]. The error on $\xi$ due to the combined scale and light-quark mass uncertainties is estimated as 1%
HPQCD 09	[59]	$2+1$	0.12, 0.09	Combined continuum and chiral extrapolation with NLO rHMS$\chi$PT and NNLO analytic terms. Light-quark discretization error is estimated as 3, 2 and 0.3% for $f_B\sqrt{B_B}$, $f_{B_s}\sqrt{B_{B_s}}$ and $\xi$ respectively	Relative scale $r_1/a$ is set via static-quark potential. Absolute scale $r_1=0.321(5)$ fm is determined through $\Upsilon$ mass [463]. The error on $f_B\sqrt{B_B}$ due to the scale uncertainty is estimated as 2.3%
HPQCD 06A	[484]	$2+1$	0.12	Only one lattice spacing is used. Light-quark discretization error on $f_{B_s}^2B_{B_s}$ is estimated as 4% by power counting	Scale is set using the $\Upsilon$ $2S-1S$ splitting as input [463]. The error on $f^2_B B_B$ due to the scale uncertainty is estimated as 5%

**Table 137:** Continuum extrapolations/estimation of lattice artefacts in determinations of the neutral *B*-meson mixing matrix elements for $N_f=2$ simulations

Collaboration	Refs.	$N_{ f}$	*a* [fm]	Continuum extrapolation	Scale setting
ETM 13B	[20]	2	0.098, 0.085, 0.067, 0.054	Combined chiral and continuum extrapolation, with a term linear in $a^2$. Discretization error is estimated by omitting the coarsest lattice as 0.5, 1.7, 1.3 and 1.0 % for $B_{B_s}$, $B_B$, $B_{B_s}/B_B$ and $\xi$ respectively. The heavy-quark masses vary in the range $0.13 \lesssim a m_h \lesssim 0.85$	See below
ETM 12A, 12B	[460, 485]	2	0.098, 0.085, 0.067	Combined chiral and continuum extrapolation, with a term linear in $a^2$. Discretization error included in combined statistical, chiral and continuum extrapolation error and estimated as 4.5%. The heavy-quark masses vary in the range $0.25 \lesssim a m_h \lesssim 0.6$	Relative scale $r_0/a$ set from the static-quark potential. Absolute scale set from $f_\pi$. Scale setting uncertainty included in combined statistical and systematic error

**Table 138:** Chiral-extrapolation/minimum pion mass in determinations of the neutral *B*-meson mixing matrix elements. For actions with multiple species of pions, masses quoted are the RMS pion masses (where available). The different $M_{\pi ,\mathrm min}$ entries correspond to the different lattice spacings

Collaboration	Refs.	$N_{ f}$	$M_{\pi ,\mathrm{min}}\,[\mathrm{MeV}]$	Description
RBC/UKQCD 14A	[54]	$2+1$	327, 289	Combined continuum and chiral extrapolation with *SU*(2) NLO HM$\chi$PT and linear in quark mass both with $\mathcal {O}(a^2)$ terms. The chiral fit error is estimated from difference between the NLO HM$\chi$PT and linear fits, and further from eliminating the heaviest *ud* quark mass point
FNAL/MILC 12	[60]	$2+1$	440, 320	Combined continuum and chiral extrapolation with NLO rHMS$\chi$PT and NNLO analytic terms. See the entry in Table 136. The omission of wrong-spin contributions [796] in the HMrS$\chi$PT is treated as a systematic error and estimated to be 3.2% for $\xi$
FNAL/MILC 11A	[483]	$2+1$	440, 320, 250	Combined continuum and chiral extrapolation with NLO rHMS$\chi$PT and NNLO analytic terms
RBC/UKQCD 10C	[464]	$2+1$	430	Linear fit matched with *SU*(2) NLO HM$\chi$PT at the lightest *ud* mass point is used as the preferred fit. Many different fit Ansätze are considered. The systematic error is estimated from the difference between the *SU*(2) HM$\chi$PT fit described above and a linear fit
HPQCD 09	[59]	$2+1$	440, 400	Combined continuum and chiral extrapolation with NLO rHMS$\chi$PT and NNLO analytic terms
HPQCD 06A	[484]	$2+1$	510	Two sea *ud* quark masses $m_{ud}/m_s=0.25$ and 0.5 are used to calculate the matrix element for $B_s$ meson at the predetermined value of the strange quark mass. No significant sea-quark mass dependence is observed and the value at the lighter sea *ud* mass is taken as the result
ETM 13B ETM 12A,12B	[20][460, 485]	2	410, 275, 300, 270	$M_{\pi ,\mathrm{min}}$ refers to the charged pions, where 270 MeV on the finest lattice only included in ETM 13B. Linear and NLO (full QCD) HM$\chi$PT supplemented by an $a^2$ term is used. The chiral fit error is estimated from the difference between the NLO HM$\chi$PT and linear fits

**Table 139:** Finite-volume effects in determinations of the neutral *B*-meson mixing matrix elements. Each *L*-entry corresponds to a different lattice spacing, with multiple spatial volumes at some lattice spacings. For actions with multiple species of pions, masses quoted are the RMS pion masses (where available)

Collaboration	Refs.	$N_{ f}$	*L* [fm]	${M_{\pi ,\mathrm{min}}}L$	Description
RBC/UKQCD 14A	[54]	$2+1$	2.74, 2.76	4.5, 4.0	FV error is estimated from *SU*(2) $\chi$PT to be 0.76, 0, 1.07% for $f_B\sqrt{B_B}$, $f_{B_s}\sqrt{B_{B_s}}$ and $\xi$ respectively
FNAL/MILC 12	[60]	$2+1$	2.4/2.9, 2.5	6.4, 5.1	FV error is estimated to be less than $0.1\%$ for *SU*(3) breaking ratios from FV HMrS$\chi$PT
FNAL/MILC 11A	[483]	$2+1$	2.4/2.9, 2.5/2.9/3.6, 3.8	6.4, 5.8, 4.9	FV error on $f_B\sqrt{B_B}$ is estimated to be less than $1\%$, which is inferred from the study of the *B*-meson decay constant using FV HM$\chi$PT [48]
RBC/UKQCD 10C	[464]	$2+1$	1.8	3.9	FV error estimated through FV HM$\chi$PT as $1\%$ for *SU*(3) breaking ratios
HPQCD 09	[59]	$2+1$	2.4/2.9, 2.5	6.4, 5.1	No explicit estimate of FV error, but expected to be much smaller than other uncertainties
HPQCD 06A	[484]	$2+1$	2.4	6.2	No explicit estimate of FV error, but expected to be much smaller than other uncertainties
ETM 13BETM 12A,12B	[20][460, 485]	2	2.4, 2.0/2.7, 2.1, 1.7/2.6	5.0, 3.7, 3.3, 3.5	$L=1.7/2.6\, \mathrm{fm}$ only included in ETM 13B. FV error is assumed to be negligible based on the study of *D*-meson decay constants in Ref. [32]

**Table 140:** Operator renormalization in determinations of the neutral *B*-meson mixing matrix elements

Collaboration	Refs.	$N_{ f}$	Ren.	Description
RBC/UKQCD 14A	[54]	$2+1$	PT1*l*	Static-light four-quark operators are renormalized with one-loop mean-field-improved PT. The errors due to neglected higher-order effects are estimated for purely $\mathcal {O}\alpha _s^2$ to be 6% on the matrix elements or 1.2% on $\xi$ and for $\mathcal {O}\alpha _s^2 a^2$ to be 1% or 0.2% respectively
FNAL/MILC 12	[60]	$2+1$	PT1*l*	One-loop mean-field improved PT is used to renormalize the four-quark operators with heavy quarks rotated to eliminate tree-level $\mathcal {O}(a)$ errors. The error from neglecting higher-order corrections is estimated to be 0.5% on $\xi$
FNAL/MILC 11A	[483]	$2+1$	PT1*l*	One-loop mean-field improved PT is used to renormalize the four-quark operators with heavy quarks rotated to eliminate tree-level $\mathcal {O}(a)$ errors. The error from neglected higher-order corrections is estimated to be 4% on $f_B\sqrt{B_B}$
RBC/UKQCD 10C	[464]	$2+1$	PT1*l*	Static-light four-quark operators are renormalized with one-loop mean-field-improved PT. The error due to neglected higher-order effects is estimated to be 2.2% on $\xi$
HPQCD 09	[59]	$2+1$	PT1*l*	Four-quark operators in lattice NRQCD are matched to QCD through order $\alpha _s$, $\Lambda _\mathrm{QCD}/M$ and $\alpha _s/(aM)$ [797] using one-loop PT. The error due to neglected higher-order effects is estimated to be 4% on $f_B\sqrt{B_B}$ and 0.7% on $\xi$
HPQCD 06A	[484]	$2+1$	PT1*l*	Four-quark operators in lattice NRQCD are matched to full QCD through order $\alpha _s$, $\Lambda _\mathrm{QCD}/M$ and $\alpha _s/(aM)$ [797]. The error is estimated as $\sim 1\cdot \alpha _s^2$ to be 9% on $f_{B_s}^2 B_{B_s}$
ETM 13B, 12A, 12B	[20, 460, 485]	2	NPR	The bag parameters are nonperturbatively renormalized in the RI’-MOM scheme. They are calculated as functions of the (${\overline{\text {MS}}}$) heavy-quark mass (renormalized nonperturbatively in RI/MOM)

**Table 141:** Heavy-quark treatment in determinations of the neutral *B*-meson mixing matrix elements

Collaboration	Refs.	$N_{ f}$	Action	Description
RBC/UKQCD 14A	[54]	$2+1$	Static	Two different static-quark actions with HYP1 and HYP2 smearings are used and the continuum extrapolation is constrained so the two values converges in the limit. The error due to the missing $1/m_b$ corrections is estimated to be 12% for individual matrix elements or 2.2% on $\xi$ using power counting
FNAL/MILC 12	[60]	$2+1$	Fermilab	The heavy-quark discretization error on $\xi$ is estimated to be 0.3%. The error on $\xi$ due to the uncertainty in the *b*-quark mass is are estimated to be 0.4%
FNAL/MILC 11A	[483]	$2+1$	Fermilab	The heavy-quark discretization error on $f_B\sqrt{B_B}$ is estimated as 4% using power counting
RBC/UKQCD 10C	[464]	$2+1$	Static	Two different static-quark actions with Ape and HYP smearings are used. The discretization error on $\xi$ is estimated as ${\sim } 4$% and the error due to the missing $1/m_b$ corrections as ${\sim }2$%, both using power counting
HPQCD 09	[59]	$2+1$	NRQCD	Heavy-quark truncation errors due to relativistic corrections are estimated to be 2.5, 2.5 and 0.4% for $f_B\sqrt{B_B}$, $f_{B_s}\sqrt{B_{B_s}}$ and $\xi$ respectively
HPQCD 06A	[484]	$2+1$	NRQCD	Heavy-quark truncation errors due to relativistic corrections are estimated to be 3% for $f_{B_s}^2{B_{B_s}}$
ETM 13BETM 12A,12B	[20][460, 485]	2	tmWil	The ratio method is used to perform an interpolation to the physical *b* quark mass from the simulated heavy mass and the known static limit. In an intermediate step, the ratios include HQET matching factors calculated to tree-level, leading-log, and next-to-leading-log (ETM 13B only) in continuum PT. The interpolation uses a polynomial up to quadratic in the inverse quark mass. The systematic errors added together with those of the chiral fit are estimated as 1.3–1.6% for bag parameters for ETM 13B, while they are estimated from changing the interpolating polynomial as 2% and from changing the order of HQET matching factors as 3% for ETM 12A and 12B

#### B.6.3 Form factors entering determinations of $|V_{ub}|$ ($B \rightarrow \pi l\nu$, $B_s \rightarrow K l\nu$, $\Lambda _b \rightarrow pl\nu$)

See Tables 142, 143, 144, 145 and 146.

**Table 142:** Continuum extrapolations/estimation of lattice artefacts in determinations of $B\rightarrow \pi \ell \nu$, $B_s\rightarrow K\ell \nu$, and $\Lambda _b\rightarrow p\ell \nu$ form factors

Collaboration	Refs.	$N_{ f}$	*a* [fm]	Continuum extrapolation	Scale setting
FNAL/MILC 15	[504]	$2+1$	0.045, 0.06, 0.09, 0.12	Fit to HMrS$\chi$PT to remove light-quark discretization errors. Residual heavy-quark discretization errors estimated with power counting. Total (stat $+$ chiral extrap $+$ HQ discretization $+$ $g_{B^*B\pi }$) error estimated to be 3.1% for $f_+$ and 3.8% for $f_0$ at $q^2=20~\mathrm{GeV}^2$	Relative scale $r_1/a$ set from the static-quark potential. Absolute scale $r_1$, including related uncertainty estimates, taken from [48]
Detmold 15 $\Lambda _b \rightarrow p$	[547]	$2+1$	0.0849(12), 0.1119(17)	Joint chiral-continuum extrapolation, combined with fit to $q^2$ dependence of form factors in a “modified” *z*-expansion. Systematics estimated by varying fit form and $\mathcal {O}(a)$ improvement parameter values	Set from $\Upsilon (2S)$–$\Upsilon (1S)$ splitting, cf. [798]
RBC/UKQCD 15	[505]	$2+1$	0.086,0.11	Joint chiral-continuum extrapolation using *SU*(2) hard-pion HM$\chi$PT. Systematic uncertainty estimated by varying fit ansatz and form of coefficients, as well as implementing different cuts on data; ranges from 5.0 to 10.9% for $B\rightarrow \pi$ form factors, and 2.5 to 5.1% for $B_s\rightarrow K$. Light-quark and gluon discretization errors estimated at 1.1 and 1.3%, respectively	Scale implicitly set in the light-quark sector using the $\Omega ^-$ mass, cf. [144]
HPQCD 14	[511]	$2+1$	0.09,0.12	Combined chiral-continuum extrapolation using hard-pion rHMS$\chi$PT. (No explicit estimate of discretization effects)	Relative scale $r_1/a$ set from the static-quark potential. Absolute scale $r_1$ set to 0.3133(23) fm
HPQCD 06	[503]	$2+1$	0.09, 0.12	Central values obtained from data at $a=0.12$ fm. Discretization errors observed to be within the statistical error by comparison with data at $a=0.09$ fm	Relative scale $r_1/a$ set from the static-quark potential. Absolute scale $r_1$ set through $\Upsilon$ 2*S*–1*S* splitting cf. HPQCD 05B [463]

**Table 143:** Chiral-extrapolation/minimum pion mass in determinations of $B\rightarrow \pi \ell \nu$, $B_s\rightarrow K\ell \nu$, and $\Lambda _b\rightarrow p\ell \nu$ form factors. For actions with multiple species of pions, masses quoted are the RMS pion masses. The different $M_{\pi ,\mathrm min}$ entries correspond to the different lattice spacings

Collaboration	Refs.	$N_{ f}$	$M_{\pi ,\mathrm{min}}\,[\mathrm{MeV}]$	Description
FNAL/MILC 15	[504]	$2+1$	330, 260, 280, 470	Simultaneous chiral-continuum extrapolation and $q^2$ interpolation using NNLO *SU*(2) hard-pion HMrS$\chi$PT. Systematic error estimated by adding higher-order analytic terms and varying the $B^*$-*B*-$\pi$ coupling
Detmold 15 $\Lambda _b \rightarrow p$	[547]	$2+1$	227, 245 (valence pions)	Joint chiral-continuum extrapolation, combined with fit to $q^2$ dependence of form factors in a “modified” *z*-expansion. Only analytic NLO terms $\propto (m_\pi ^2-m_{\pi ,\mathrm{phys}}^2)$ included in light mass dependence. Systematic uncertainty estimated by repeating fit with added higher-order terms
RBC/UKQCD 15	[505]	$2+1$	289, 329	Joint chiral-continuum extrapolation using *SU*(2) hard-pion HM$\chi$PT. Systematic uncertainty estimated by varying fit ansatz and form of coefficients, as well as implementing different cuts on data; ranges from 5.0 to 10.9% for $B\rightarrow \pi$ form factors, and 2.5 to 5.1% for $B_s\rightarrow K$
HPQCD 14	[511]	$2+1$	295, 260	Combined chiral-continuum extrapolation using hard-pion rHMS$\chi$PT. (No explicit estimate of extrapolation systematics)
HPQCD 06	[503]	$2+1$	400, 440	First interpolate data at fixed quark mass to fiducial values of $E_\pi$ using the Becirevic–Kaidalov and Ball–Zwicky ansätze, then extrapolate data at fixed $E_\pi$ to physical quark masses using *SU*(3) rHMS$\chi$PT. Systematic error estimated by varying interpolation and extrapolation fit functions

**Table 144:** Finite-volume effects in determinations of $B\rightarrow \pi \ell \nu$, $B_s\rightarrow K\ell \nu$, and $\Lambda _b\rightarrow p\ell \nu$ form factors. Each *L*-entry corresponds to a different lattice spacing, with multiple spatial volumes at some lattice spacings. For actions with multiple species of pions, the lightest masses are quoted

Collaboration	Refs.	$N_{ f}$	*L* [fm]	${M_{\pi ,\mathrm{min}}}L$	Description
FNAL/MILC 15	[504]	$2+1$	2.9, 2.9/3.4/3.8, 2.5/2.9/3.6/5.8, 2.4/2.9	${\gtrsim } 3.8$	FV effects estimated by replacing infinite-volume chiral logs with sums over discrete momenta, found to be negligible
Detmold 15 $\Lambda _b \rightarrow p$	[547]	$2+1$	2.7, 2.7	${\gtrsim } 3.1$ (valence sector)	FV effect estimated at 3% from experience on $\chi$PT estimates of FV effects for heavy-baryon axial couplings
RBC/UKQCD 15	[505]	$2+1$	2.8, 2.6	4.0, 4.4	FV effects estimated by correction to chiral logs due to sums over discrete momenta; quoted 0.3–0.5% for $f_+$ and 0.4–0.7% for $f_0$ for $B\rightarrow \pi$, and 0.2% for $f_+$ and 0.1–0.2% for $f_0$ for $B_s\rightarrow K$
HPQCD 14	[511]	$2+1$	2.5, 2.4/2.9	${\gtrsim } 3.8$	FV effects estimated by shift of pion log, found to be negligible
HPQCD 06	[503]	$2+1$	2.4/2.9	${\gtrsim } 3.8$	No explicit estimate of FV error, but expected to be much smaller than other uncertainties

**Table 145:** Operator renormalization in determinations of $B\rightarrow \pi \ell \nu$, $B_s\rightarrow K\ell \nu$, and $\Lambda _b\rightarrow p\ell \nu$ form factors

Collaboration	Refs.	$N_{ f}$	Ren.	Description
FNAL/MILC 15	[504]	$2+1$	mNPR	Perturbative truncation error estimated at 1% with size of one-loop correction on next-to-finer ensemble
Detmold 15 $\Lambda _b \rightarrow p$	[547]	$2+1$	mNPR	Perturbative truncation error estimated at 1% with size of one-loop correction on next-to-finer ensemble
RBC/UKQCD 15	[505]	$2+1$	mNPR	Perturbative truncation error estimated as largest of power counting, effect from value of $\alpha _s$ used, numerical integration. Nonperturbative normalization of flavour-diagonal currents computed by fixing values of ratios of meson 2-point functions to 3-point functions with an extra current inversion, cf. [53]
HPQCD 14	[511]	$2+1$	mNPR	Currents matched using one-loop HISQ lattice perturbation theory, omitting $\mathcal {O}(\alpha _s\Lambda _\mathrm{QCD}/m_b$. Systematic uncertainty resulting from one-loop matching and neglecting $\mathcal {O}(\Lambda _\mathrm{QCD}^2/m_b^2$ terms estimated at 4% from power counting
HPQCD 06	[503]	$2+1$	PT1$\ell$	Currents included through $\mathcal {O}(\alpha _S \Lambda _\mathrm{QCD}/M$, $\alpha _S/(aM)$, $\alpha _S\, a \Lambda _\mathrm{QCD})$. Perturbative truncation error estimated from power counting

**Table 146:** Heavy-quark treatment in determinations of $B\rightarrow \pi \ell \nu$, $B_s\rightarrow K\ell \nu$, and $\Lambda _b\rightarrow p\ell \nu$ form factors

Collaboration	Refs.	$N_{ f}$	Action	Description
FNAL/MILC 15	[504]	$2+1$	Fermilab	Total statistical $+$ chiral extrapolation $+$ heavy-quark discretization $+$ $g_{B^*B\pi }$ error estimated to be 3.1% for $f_+$ and 3.8% for $f_0$ at $q^2=20~\mathrm{GeV}^2$
Detmold 15 $\Lambda _b \rightarrow p$	[547]	$2+1$	Columbia RHQ	Discretization errors discussed as part of combined chiral-continuum-$q^2$ fit, stemming from $a^2|{\mathbf {p}}|^2$ terms
RBC/UKQCD 15	[505]	$2+1$	Columbia RHQ	Discretization errors estimated by power counting to be 1.8% for $f_+$ and 1.7% for $f_0$
HPQCD 14	[511]	$2+1$	NRQCD	Currents matched using one-loop HISQ lattice perturbation theory, omitting $\mathcal {O}(\alpha _s\Lambda _\mathrm{QCD}/m_b$. Systematic uncertainty resulting from one-loop matching and neglecting $\mathcal {O}(\Lambda _\mathrm{QCD}^2/m_b^2$ terms estimated at 4% from power counting
HPQCD 06	[503]	$2+1$	NRQCD	Discretization errors in $f_+(q^2)$ estimated to be $\mathcal {O}(\alpha _s (a \Lambda _\mathrm{QCD})^2) \sim 3\%$. Relativistic errors estimated to be $\mathcal {O}((\Lambda _\mathrm{QCD}/M)^2) \sim 1\%$

#### B.6.4 Form factors for $B \rightarrow K\ell ^+\ell ^-$

See Tables 147, 148, 149, 150 and 151.

**Table 147:** Continuum extrapolations/estimation of lattice artefacts in determinations of form factors for $B\rightarrow K\ell ^+\ell ^-$

Collaboration	Refs.	$N_{ f}$	*a* [fm]	Continuum extrapolation	Scale setting
FNAL/MILC 15D	[516]	$2+1$	0.045, 0.06, 0.09, 0.12	Fit to $\mathrm{SU}(2)$ HMrS$\chi$PT for the combined chiral-continuum limit extrapolation. Combined stat $+$ chiral extrap $+$ HQ discretization $+$ $g_{B^*B\pi }$ error provided as a function of $q^2$ for each form factor, ranging between ${\sim } 1.4$% and ${\sim } 2.8$%	Relative scale $r_1/a$ set from the static-quark potential. Absolute scale $r_1$, including related uncertainty estimates, taken from [48]
HPQCD 13E	[518]	$2+1$	0.09, 0.12	Combined chiral-continuum extrapolation using rHMS$\chi$PT. Errors provided as a function of $q^2$, combined total ranging from ${\sim } 3$% to ${\sim } 5$% in data region	Relative scale $r_1/a$ set from the static-quark potential. Absolute scale $r_1$ set to 0.3133(23) fm

**Table 148:** Chiral-extrapolation/minimum pion mass in determinations of form factors for $B\rightarrow K\ell ^+\ell ^-$. For actions with multiple species of pions, masses quoted are the RMS pion masses. The different $M_{\pi ,\mathrm{min}}$ entries correspond to the different lattice spacings

Collaboration	Refs.	$N_{ f}$	$M_{\pi ,\mathrm{min}}\,[\mathrm{MeV}]$	Description
FNAL/MILC 15D	[516]	$2+1$	330, 260, 280, 470	Simultaneous chiral-continuum extrapolation and $q^2$ interpolation using $\mathrm{SU}(2)$ HMrS$\chi$PT, with a hard-kaon $\chi$PT treatment of high-energy kaons. Combined stat $+$ chiral extrap $+$ HQ discretization $+$ $g_{B^*B\pi }$ error provided as a function of $q^2$ for each form factor, ranging between ${\sim } 1.4$% and ${\sim } 2.8$%
HPQCD 13E	[518]	$2+1$	295, 260	Combined chiral-continuum extrapolation using rHMS$\chi$PT. Errors provided as a function of $q^2$, combined total ranging from ${\sim } 3$% to ${\sim } 5$% in data region

**Table 149:** Finite-volume effects in determinations of form factors for $B\rightarrow K\ell ^+\ell ^-$. Each *L*-entry corresponds to a different lattice spacing, with multiple spatial volumes at some lattice spacings. For actions with multiple species of pions, the lightest masses are quoted

Collaboration	Refs.	$N_{ f}$	*L* [fm]	${M_{\pi ,\mathrm{min}}}L$	Description
FNAL/MILC 15D	[516]	$2+1$	2.9, 2.9/3.8, 2.5/2.9/3.6/5.8, 2.4/2.9	${\gtrsim } 3.8$	FV effects estimated by replacing infinite-volume chiral logs with sums over discrete momenta, found to be negligible
HPQCD 13E	[518]	$2+1$	2.5, 2.4/2.9	${\gtrsim } 3.8$	FV effects included in combined chiral-continuum extrapolation

**Table 150:** Operator renormalization in determinations of form factors for $B\rightarrow K\ell ^+\ell ^-$

Collaboration	Refs.	$N_{ f}$	Ren.	Description
FNAL/MILC 15D	[516]	$2+1$	mNPR	Perturbative truncation error estimated at 1% for $f_+$ and $f_0$ and 2% for $f_T$, using size of one-loop correction on next-to-finer ensemble
HPQCD 13E	[518]	$2+1$	mNPR	Currents matched using one-loop massless-HISQ lattice perturbation theory. Associated systematic uncertainty dominates quoted 4% uncertainty from matching, charm quenching, and electromagnetic and isospin-breaking effects

**Table 151:** Heavy-quark treatment in determinations of form factors for $B\rightarrow K\ell ^+\ell ^-$

Collaboration	Refs.	$N_{ f}$	Action	Description
FNAL/MILC 15D	[516]	$2+1$	Fermilab	Combined stat $+$ chiral extrap $+$ HQ discretization $+$ $g_{B^*B\pi }$ error provided as a function of $q^2$ for each form factor, ranging between ${\sim } 1.4$% and ${\sim } 2.8$%
HPQCD 13E	[518]	$2+1$	NRQCD	Currents matched using one-loop massless-HISQ lattice perturbation theory. Associated systematic uncertainty dominates quoted 4% uncertainty from matching, charm quenching, and electromagnetic and isospin-breaking effects

#### B.6.5 Form factors entering determinations of $|V_{cb}|$ ($B \rightarrow D^* l\nu$, $B \rightarrow D l\nu$, $B_s \rightarrow D_s l\nu$, $\Lambda _b \rightarrow \Lambda _c l\nu$) and *R*(*D*))

See Tables 152, 153, 154, 155 and 156.

**Table 152:** Continuum extrapolations/estimation of lattice artefacts in determinations of $B\rightarrow D \ell \nu$, $B\rightarrow D^* \ell \nu$, $B_s\rightarrow D_s \ell \nu$, and $\Lambda _b\rightarrow \Lambda _c\ell \nu$ form factors, and of *R*(*D*)

Collaboration	Refs.	$N_{ f}$	*a* [fm]	Continuum extrapolation	Scale setting
HPQCD 15	[541]	$2+1$	0.09, 0.12	Combined chiral-continuum extrapolation as part of modified *z*-expansion of form factors, which also includes uncertainty related to matching of NRQCD and relativistic currents	Implicitly set from $r_1$
FNAL/MILC 15C	[540]	$2+1$	0.045, 0.06, 0.09, 0.12	Combined chiral-continuum extrapolation using HMrS$\chi$PT. Form factors fitted to NLO $\chi$PT, with chiral logs taken from staggered version of the Chow-Wise result, modified to include taste-breaking terms. $\mathcal {O}(a^2)$ terms introduced based on power-counting arguments. Total uncertainty estimated at 0.6% for $f_+$ and 0.5% for $f_0$ for the largest recoil	Relative scale $r_1/a$ set from the static-quark potential. Absolute scale $r_1$, including related uncertainty estimates, taken from [48]. Uncertainty related to scale setting estimated at 0.2%
Detmold 15 $\Lambda _b \rightarrow \Lambda _c$	[547]	$2+1$	0.0849(12), 0.1119(17)	Joint chiral-continuum extrapolation, combined with fit to $q^2$ dependence of form factors in a “modified” *z*-expansion. Systematics estimated by varying fit form and $\mathcal {O}(a)$ improvement parameter values	Set from $\Upsilon (2S)$–$\Upsilon (1S)$ splitting, cf. [798]
FNAL/MILC 14	[539]	$2+1$	0.045, 0.06, 0.09, 0.12, 0.15	Combined chiral-continuum extrapolation using HMrS$\chi$PT. Total uncertainty quoted at 0.5%	Relative scale $r_1/a$ set from the static-quark potential. Absolute scale $r_1$, including related uncertainty estimates, taken from [48]. Uncertainty related to scale setting estimated at 0.1%
Atoui 13	[537]	2	0.054, 0.067, 0.085, 0.098	Combined continuum and chiral extrapolation, with linear terms in $a^2$ and $m_\mathrm{sea}$. No dependence on *a* or $m_\mathrm{sea}$ observed within errors. Stability of results vs. fits with no $m_\mathrm{sea}$ dependence checked	Scale set through $F_\pi$

**Table 153:** Chiral-extrapolation/minimum pion mass in determinations of $B\rightarrow D \ell \nu$, $B\rightarrow D^* \ell \nu$, $B_s\rightarrow D_s \ell \nu$, and $\Lambda _b\rightarrow \Lambda _c\ell \nu$ form factors, and of *R*(*D*). For actions with multiple species of pions, masses quoted are the RMS pion masses. The different $M_{\pi ,\mathrm{min}}$ entries correspond to the different lattice spacings

Collaboration	Refs.	$N_{ f}$	$M_{\pi ,\mathrm{min}}\,[\mathrm{MeV}]$	Description
HPQCD 15	[541]	$2+1$	295, 260	Combined chiral-continuum extrapolation as part of modified *z*-expansion of form factors. Hard-pion $\chi$PT for light mass dependence used to estimate systematic uncertainty to be 1.14%
FNAL/MILC 15C	[540]	$2+1$	330, 260, 280, 470	Combined chiral-continuum extrapolation using HMrS$\chi$PT. Form factors fitted to NLO $\chi$PT, with chiral logs taken from staggered version of the Chow-Wise result, modified to include taste-breaking terms. $\mathcal {O}(a^2)$ terms introduced based on power-counting arguments. Total uncertainty estimated at 0.6% for $f_+$ and 0.5% for $f_0$ for the largest recoil
Detmold 15 $\Lambda _b \rightarrow \Lambda _c$	[547]	$2+1$	227, 245 (valence pions)	Joint chiral-continuum extrapolation, combined with fit to $q^2$ dependence of form factors in a “modified” *z*-expansion. Only analytic *NLO* terms $\propto (m_\pi ^2-m_{\pi ,\mathrm{phys}}^2)$ included in light mass dependence. Systematic uncertainty estimated by repeating fit with added higher-order terms
FNAL/MILC 14	[539]	$2+1$	330, 260, 280, 470, 590	Combined chiral-continuum extrapolation using HMrS$\chi$PT. Systematic errors estimated by adding higher-order analytic terms and varying the $D^*$-*D*-$\pi$ coupling. Total uncertainty quoted at 0.5%
Atoui 13	[537]	2	270, 300, 270, 410	Combined continuum and chiral extrapolation, with linear terms in $a^2$ and $m_\mathrm{sea}$. No dependence on *a* or $m_\mathrm{sea}$ observed within errors. Stability of results vs. fits with no $m_\mathrm{sea}$ dependence checked

**Table 154:** Finite-volume effects in determinations of $B\rightarrow D \ell \nu$, $B\rightarrow D^* \ell \nu$, $B_s\rightarrow D_s \ell \nu$, and $\Lambda _b\rightarrow \Lambda _c\ell \nu$ form factors, and of *R*(*D*). Each *L*-entry corresponds to a different lattice spacing, with multiple spatial volumes at some lattice spacings. For actions with multiple species of pions, the lightest pion masses are quoted

Collaboration	Refs.	$N_{ f}$	*L* [fm]	${M_{\pi ,\mathrm{min}}}L$	Description
HPQCD 15	[541]	$2+1$	2.5, 2.4/2.9	${\gtrsim } 3.8$	FV effects estimated to be negligible
FNAL/MILC 15C	[540]	$2+1$	2.9, 2.9–3.8, 2.5–5.8, 2.4/2.9	${\gtrsim } 3.8$	FV error estimated to be negligible in [542]
Detmold 15 $\Lambda _b \rightarrow \Lambda _c$	[547]	$2+1$	2.7, 2.7	${\gtrsim } 3.1$ (valence sector)	FV effect estimated at 1.5% from experience on $\chi$PT estimates of FV effects for heavy-baryon axial couplings
FNAL/MILC 14	[539]	$2+1$	2.9, 2.9–3.8, 2.4–5.5, 2.4/2.9, 2.4	${\gtrsim } 3.8$	FV error estimated to be negligible
Atoui 13	[537]	2	1.7/2.6, 2.1, 2.0/2.7, 2.4	${\gtrsim } 3.6$	No volume dependence observed within errors

**Table 155:** Operator renormalization in determinations of $B\rightarrow D \ell \nu$, $B\rightarrow D^* \ell \nu$, $B_s\rightarrow D_s \ell \nu$, and $\Lambda _b\rightarrow \Lambda _c\ell \nu$ form factors, and of *R*(*D*)

Collaboration	Refs.	$N_{ f}$	Ren.	Description
HPQCD 15	[541]	$2+1$	One loop.	One-loop matching of currents taken from [758]
FNAL/MILC 15C	[540]	$2+1$	mNPR	Form factors extracted from ratios of correlators that renormalize with ratios of current normalizations, computed at one-loop in perturbation theory. Dependence of renormalization factor on recoil parameter *w* neglected. Systematic uncertainty due to perturbative truncation and *w*-dependence estimated by power counting to 0.7%
Detmold 15 $\Lambda _b \rightarrow \Lambda _c$	[547]	$2+1$	mNPR	Perturbative truncation error estimated at 1% with size of one-loop correction on next-to-finer ensemble
FNAL/MILC 14	[539]	$2+1$	mNPR	Majority of current renormalization factor cancels in double ratio of lattice correlation functions. Remaining correction calculated with one-loop tadpole-improved lattice perturbation theory. Systematic uncertainty estimated at 0.4%
Atoui 13	[537]	2	–	Observables obtained from ratios that do not require renormalization. Checks performed by comparing with results coming from currents that are renormalized separately with nonperturbative $Z_\mathrm{V}$

**Table 156:** Heavy-quark treatment in determinations of $B\rightarrow D \ell \nu$, $B\rightarrow D^* \ell \nu$, $B_s\rightarrow D_s \ell \nu$, and $\Lambda _b\rightarrow \Lambda _c\ell \nu$ form factors, and of *R*(*D*)

Collaboration	Refs.	$N_{ f}$	Action	Description
HPQCD 15	[541]	$2+1$	NRQCD for b quark, HISQ for c quark	Discretization errors estimated via power counting to be 2.59%
FNAL/MILC 15C	[540]	$2+1$	Fermilab	Discretization errors of form factors estimated via power counting to be 0.4%
Detmold 15 $\Lambda _b \rightarrow \Lambda _c$	[547]	$2+1$	Columbia RHQ	Discretization errors discussed as part of combined chiral-continuum-$q^2$ fit, stemming from $a^2|\mathbf {p}|^2$ terms
FNAL/MILC 14	[539]	$2+1$	Fermilab	Discretization errors estimated via power counting to be 1%
Atoui 13	[537]	2	tmWil	Results obtained from step scaling in heavy-quark mass via the ratio method. Separate continuum limit extrapolations with mild $a^2$ dependence carried out for each mass point separately. Result at physical value of $m_b$ obtained by interpolation between data region and known exact HQET limit

### B.7 Notes to Sect. 9 on the strong coupling $\alpha _\mathrm{s}$

#### B.7.1 Renormalization scale and perturbative behaviour

See Tables 157, 158, 159 and 160.

**Table 157:** Renormalization scale and perturbative behaviour of $\alpha _s$ determinations for $N_f=0$

Collaboration	Refs.	$N_{ f}$	$\alpha _\mathrm{eff}$	$n_l$	Description
FlowQCD 15	[564]	0	0.09–0.12	2	$\alpha _{\overline{\text {MS}}}(2.63/a)$ computed from the boosted coupling. The physical volume ranges from $2.4 \sim 3.8\,\hbox {fm}$
Sternbeck 12	[652]	0	0.11–0.18	3	$$\alpha _{\mathrm {T}}(p)$$ for $$p = 5 $$–40 GeV. Fitted to PT without power corrections. ($$\beta = 6.0, 6.4, 6.7, 6.92$$)
Ilgenfritz 10	[655]	0	0.07–0.9	3	$\alpha _\mathrm{T}(p)$ for $p = 1$–240 GeV. ($\beta = 5.8$, 6.0, 6.2, 6.4, 9.0)
Sternbeck 10	[653]	0	0.07–0.32	3	$\alpha _{\mathrm {T}}$ for $p = 2.5$–140 GeV, fitted to PT partially on very small lattices
Brambilla 10	[606]	0	0.22–0.47	3	$$\alpha _{\mathrm {qq}}(1/r)$$ for the range $$r/r_0 = 0.15 $$–0.5. Fit of *V*(*r*) to PT with renormalon subtraction and resummation reproduces the static potential for $$r/r_0 = 0.15$$–0.45 well
Boucaud 08	[648]	0	0.18–0.35	3	$\alpha _{\mathrm {T}}(p)$ with $p = 3$–6 GeV. Fitted to PT with $1/p^2$ correction
Boucaud 05	[645]	0	0.22–0.55	3	$\Lambda _{\widetilde{\mathrm{MOM}}_{g,c}}$ using gluon and ghost propagators with $2 \le \mu \le 6$ GeV. Fit to perturbation theory
QCDSF-UKQCD 05	[625]	0	0.10–0.15	2	$\alpha _{{\overline{\text {MS}}}}(2.63/a)$ computed from the boosted coupling
CP-PACS 04	[582]	0	0.08–0.28	2	$\alpha _\mathrm{SF}(1/L)$ step-scaling functions at $\alpha _\mathrm{eff} = 0.08, 0.19$, study of continuum limit. Agreement of continuum limit with ALPHA 98
Boucaud 01A	[657]	0	0.18–0.45	2	$$\alpha _\mathrm{MOM}$$ with $$p = 2.5 $$–10 GeV. Consistency check of $$n_l=2$$ loop perturbation formula with gluon condensate. $$\langle A^2 \rangle $$ from $$\alpha _\mathrm{MOM}$$ and gluon propagator are consistent
Soto 01	[656]	0	0.25–0.36, 0.3–0.36, 0.19–0.24	2	$\alpha _{\widetilde{\mathrm{MOM}}}$ for $p = 3$–10 GeV. Fit with $n_l= 2$ loop formula with gluon condensate. (Without condensate does not fit the lattice data.) ($\beta = 6.0,$ 6.2, 6.8)
Boucaud 00A	[659]	0	0.35–0.55, 0.25–0.45, 0.22–0.28, 0.18–0.22	2	$$\alpha _{\widetilde{\mathrm{MOM}}}$$ with $$p = 2 $$–10 GeV. Fitted to $$n_l = 2$$ loop perturbation theory with power correction. ($$\beta = 6.0,$$ 6.2, 6.4, 6.8)
Boucaud 00B	[658]	0	0.35–0.55, 0.25–0.45, 0.22–0.28, 0.18–0.22	2	$\alpha _\mathrm{MOM}$ with $2 \le \mu \le 10$ GeV. Consistency check of $n_l = 2$ loop perturbation formula with gluon condensate. $\beta _2^{MOM}=1.5\times \beta _2^{\widetilde{MOM}}$ is needed. ($\beta = 6.0,$ 6.2, 6.4, 6.8)
Becirevic 99A	[661]	0	0.25–0.4	2	$\alpha _{\widetilde{\mathrm{MOM}}}$ with $p = 2.5$–5.5 GeV
Becirevic 99B	[660]	0	0.18–0.25	2	$\alpha _{\widetilde{\mathrm{MOM}}}$ from a single lattice spacing with $p = 5.6$–9.5 GeV
SESAM 99	[623]	0	0.15	1	$\alpha _{\mathrm {V}}(3.41/a)$ computed from the boosted coupling
ALPHA 98	[590]	0	0.07–0.28	2	$\alpha _\mathrm{SF}(1/L)$ step scaling, agreement with perturbative running ($n_l=2$) for $\alpha _\mathrm{eff} < 0.15$
Boucaud 98A	[663]	0	0.35–0.5	1, 2	$\alpha _{\mathrm {MOM}}$, with $2.1 \le \mu \le 3.9$ GeV. $n_l=1$ for $\alpha _\mathrm{MOM}$, $n_l=2$ for $\alpha _{\widetilde{\mathrm{MOM}}}$
Boucaud 98B	[662]	0	0.27–0.50	2	$\alpha _{\widetilde{\mathrm{MOM}}}$ with $\mu = 2.2$–4.5 GeV
Alles 96	[643]	0	0.35–0.71	1	$\alpha _{\widetilde{\mathrm{MOM}}}(p)$ with $p=1.8$–3.0 GeV
Wingate 95	[624]	0	0.15	1	$\alpha _{V}(3.41/a)$ computed from the boosted coupling
Davies 94	[622]	0	0.15	1	$\alpha _{V}(3.41/a)$ computed from the boosted coupling
Lüscher 93	[579]	0	0.09–0.28	1	$\alpha _\mathrm{SF}(1/L)$ step scaling, agreement with perturbative running ($n_l=1$) for $\alpha _\mathrm{eff} < 0.17$
UKQCD 92	[594]	0	0.17–0.40	1	$\alpha _{\mathrm {qq}}(1/r)$ for a single lattice spacing. Fit of $\alpha _{\mathrm {qq}}(1/r)$ to a NLO formula
Bali 92	[607]	0	0.15–0.35	1	$\alpha _{\mathrm {qq}}(1/r)$ for the lattice spacing used in the analysis. Box size $L\approx 1.05$ fm. Fit of $\alpha _{\mathrm {qq}}(1/r)$ to a NLO formula. $\Lambda _{{\overline{\text {MS}}}}$ is found to depend on the fit range
El-Khadra 92	[620]	0	0.12–0.15	1	$\alpha _{{\overline{\text {MS}}}}(\pi /a)$ from one-loop boosted perturbation theory

**Table 158:** Renormalization scale and perturbative behaviour of $\alpha _s$ determinations for $N_f=2$

Collaboration	Refs.	$N_{ f}$	$\alpha _\mathrm{eff}$	$n_l$	Description
Karbstein 14	[563]	2	0.28–0.41	3	$\alpha _V(p)$ for momentum $1.5< p < 3.0\,\hbox {GeV}$. Values computed from the quoted $\Lambda$ parameter with the two-loop $\beta$ function; larger values (0.32–0.62) are obtained with 3-loop running. As with ETM 11C central values are taken from $a = 0.042\,\hbox {fm}$ lattice with $L = 1.3\,\hbox {fm}$ and $m_\pi = 350\,\hbox {MeV}$
ALPHA 12	[12]	2	See ALPHA 04	2	Determination of $\Lambda _{\overline{\text {MS}}}/ f_K$ using ALPHA 04
Sternbeck 12	[652]	2	0.17–0.23	3	$\alpha _{\mathrm {T}}$ for $(r_0 p)^2=200$–2000. Fit to PT without condensate. Deviation at higher energy is observed
ETM 11C	[605]	2	0.26–0.96	3	$\alpha _\mathrm{qq}(1/r)$ as computed by us from $\Lambda _{\overline{\text {MS}}}=315$ MeV. Fit of *V*(*r*) to PT with renormalon subtraction and resummation reproduces the static potential for $r/r_0=0.2$–0.6 well. One fit range, using $r/a=2$–4 at the smallest lattice spacing corresponds to $\alpha _\mathrm{eff}=0.26$–0.40. In the ${\overline{\text {MS}}}$ scheme one has $\alpha _{\overline{\text {MS}}}(1/r)=0.24$–0.63 and for the restricted fit $\alpha _{\overline{\text {MS}}}(1/r)=0.24$–0.36. Central values taken from $a=0.042$ fm lattice with $L=1.3$ fm and $m_\pi =350$ MeV
ETM 10F	[654]	2	0.24–0.45	3	$\alpha _T$ for momentum up to 2.6–5.6 GeV. Fitted to PT with gluon condensate correction term
Sternbeck 10	[653]	2	0.19–0.38	3	$\alpha _{\mathrm {T}}$ for $1\le (ap)^2\le 10$. Fitted with $n_l = 3$ loop formula
JLQCD 08	[614]	2	0.25–0.30	1	$\alpha _{\overline{\mathrm{MS}}}(Q)$ for $0.65<(aQ)^2<1.32$. Fit with the perturbative formula with power corrections
QCDSF-UKQCD 05	[625]	2	0.18–0.20	2	$\alpha _{\overline{\text {MS}}}(1.4/a)$ computed from the boosted coupling
ALPHA 04	[588]	2	0.078–0.44	2	$\alpha _\mathrm{SF}(1/L)$ step scaling, agreement with $n_l = 2$ looprunning for $\alpha _s < 0.2$
ALPHA 01	[589]	2	0.078–0.44	2	$\alpha _\mathrm{SF}(1/L)$ step scaling, agreement with $n_l=2$ loop running for $\alpha _s < 0.2$
Boucaud 01B	[644]	2	0.25–0.5	3	$\alpha _\mathrm{\widetilde{MOM}}$ for momentum up to 7 GeV. Fitted with $n_l = 3$ loop formula with and without power correction, leading to different results for $\Lambda _{\overline{\text {MS}}}^{(2)}$. Extrapolation of $\alpha _s(1.3\,\,\mathrm {GeV})$ in $N_f$ from $N_f=0, 2$ to $N_f=3$ is made
SESAM 99	[623]	2	0.17	1	The boosted coupling $\alpha _\mathrm{P}(3.41/a)$
Wingate 95	[624]	2	0.18	1	$\alpha _\mathrm{V}(3.41/a)$ computed from the boosted coupling
Aoki 94	[621]	2	0.14	1	$\alpha _{\overline{\text {MS}}}(\pi /a)$ computed from the boosted coupling
Davies 94	[622]	2	0.18	1	$\alpha _\mathrm{V}(3.41/a)$ computed from $\ln W_{11}$

**Table 159:** Renormalization scale and perturbative behaviour of $\alpha _s$ determinations for $N_f=3$

Collaboration	Refs.	$N_{ f}$	$\alpha _\mathrm{eff}$	$n_l$	Description
Bazavov 14	[61]	$2+1$	0.19–0.41	3	Update of Bazavov 12 including finer lattices down to $a = 0.041\,\hbox {fm}$. Fit range $r/r_1 = 0.12$–0.50 ($r/r_0 =$ 0.08–0.33). Perturbative expansion of the force *F*(*r*) integrated to determine potential
Bazavov 12	[604]	$2+1$	0.23–0.57	3	$\alpha _\mathrm{qq}$ computed by us from $\Lambda _{\overline{\text {MS}}}r_0=0.70$. Fit of *V*(*r*) to PT with renormalon subtraction and resummation reproduces the static potential for $r/r_0=0.135$–0.5 well
Sternbeck 12	[652]	$2+1$	0.19–0.25	3	$\alpha _{\mathrm {T}}$ for $(p r_0)^2=200$–2000. Comparison with 4-loop formula
JLQCD 10	[613]	$2+1$	0.29–0.35	2	$\alpha _{\overline{\mathrm{MS}}}(Q)$ for $0.4<(aQ)^2<1.0$. Fit with the perturbative formula with power corrections
HPQCD 10	[9]	$2+1$		2	Uses method of Sect. 9.6. Update of $r_1$ and $r_1/a$ in HPQCD 08A
HPQCD 10	[9]	$2+1$	0.12–0.42	2	Uses method of Sect. 9.7. $\alpha _{\mathrm {eff}}$ from $R_4$ and $R_6/R_8$. Fit of $R_n, \;n=4\ldots 10$ to PT including $(a m)^{2i}$ terms with $i\le 10$; coefficients constrained by priors
PACS-CS 09A	[62]	$2+1$	0.08–0.27	2	$\alpha _\mathrm{SF}(1/L)$ step scaling, agreement with 3-loop running for $\alpha _s \le 0.27$
HPQCD 08B	[152]	$2+1$	0.38	2	Fit of the ratios to PT at the charm mass including $(a m)^{2i}$ terms with $i\le 2\ldots 4$; coefficients constrained by priors
HPQCD 08A	[617]	$2+1$	0.15–0.4	2	$\alpha _\mathrm {V}(q^*)$ for a variety of short-distance quantities, using same method as in HPQCD 05A
Maltman 08	[63]	$2+1$		2	Re-analysis of HPQCD 05A for a restricted set of short-distance quantities with similar results
HPQCD 05A	[616]	$2+1$	0.2–0.4	2	$\alpha _\mathrm {V}(q^*)$ for a variety of short-distance quantities

**Table 160:** Renormalization scale and perturbative behaviour of $\alpha _s$ determinations for $N_f=4$

Collaboration	Refs.	$N_{ f}$	$\alpha _\mathrm{eff}$	$n_l$	Description
HPQCD 14A	[5]	$2+1+1$	0.11–0.33	2	Range given for $\alpha _{\mathrm {eff}}$ from $\tilde{R}_4$. Fit of ratios $R_n\;n=4\ldots 10$ to perturbation theory including $(a m)^{2i}$ terms with $i\le 10$–20 and higher-order perturbative terms; coefficients constrained by priors
ETM 13D	[649]	$2+1+1$	0.26–0.7	3	$\alpha _{\mathrm {T}}(p)$ for $p=1.6$–6.5 GeV. Update of [650] with improved power law determination
ETM 12C	[650]	$2+1+1$	0.24–0.38	3	$\alpha _{\mathrm {T}}(p)$ for $p=1.7$–6.8 GeV. Fit to PT with gluon condensate correction or higher power
ALPHA 10A	[586]	4	0.07–0.28	2	$\alpha _{\mathrm {SF}}(1/L)$. Comparison to PT with 2-, 3-loop $\beta$-function
ETM 11D	[651]	$2+1+1$	0.24–0.4	3	$\alpha _{\mathrm {T}}(p)$ for $p=3.8$–7.1 GeV with H(4)-procedure. Fit to PT with gluon condensate correction
Perez 10	[587]	4	0.06–0.28	2	$\alpha _{\mathrm {SF}}(1/L)$. Comparison with 1-, 2-, 3-loop $\beta$-function

#### B.7.2 Continuum limit

See Tables 161, 162, 163, 164 and 165.

**Table 161:** Continuum limit for $\alpha _s$ determinations with $N_f=0$

Collaboration	Refs.	$N_{ f}$	$a\,\mu$	Description
FlowQCD 15	[564]	0	9 Lattice spacings with $a = 0.06$–$0.02\,\hbox {fm}$	$w_{0.4}/a$, together with $r_0 = 0.5\,\hbox {fm}$ and conversion factor $r_0/w_{0.4} = 2.587(45)$
Sternbeck 12	[652]	0	4 Lattice spacings $a\le 0.1$ fm	At $\alpha _s = 0.18,$ $ap = 2.7, 1.5$ for $\beta = 6.0, 6.4$
Brambilla 10	[606]	0	At least three lattice spacings with $0.2 \le 2a/r \le 1.1$	Extrapolation of potential differences $V(r)-V(0.51r_0)$ linear in $a^2$ performed in [570] with several lattice spacings
Ilgenfritz 10	[655]	0	$a = 0.136,$ 0.093, 0.068, 0.051 fm ($\beta = 5.8, 6.0, 6.2,$ 6.4), while no value of *a* is given for $\beta = 9.0$	At $\alpha _s = 0.3$, $ap = 2.0,$ 1.4, 1.0, 0.8 ($\beta = 5.8, 6.0,$ 6.2, 6.4). For $\beta = 9.0$ at $ap = 1.4$, $\alpha _s = 0.082$
Sternbeck 10	[653]	0	8 Lattice spacings $a = 0.004{-} 0.087$ fm ($r_0 = 0.467$ fm)	$\sqrt{3}< ap < \sqrt{12}$
Boucaud 08	[648]	0	$a = 0.1, 0.07, 0.05$ fm	At $\alpha _s = 0.3$ the data have $ap = 2.6, 1.9, 1.5$
QCDSF/UKQCD 05	[625]	0	7 Lattice spacings with $a = 0.10$–$0.028\,\hbox {fm}$	$r_0/a$, together with $r_0 = 0.467\,\hbox {fm}$
Boucaud 05	[645]	0	$a = 0.1, 0.07, 0.05$ fm	At $\alpha _s \le 0.3$ $ap = 1.9, 1.4, 1.0$
CP-PACS 04	[582]	0	4 Spacings, $a/L = 1/12 - 1/4$.	Iwasaki and Lüscher Weisz tree-level improved bulk actions; boundary improvement at tree-level, one-loop and with two different choices of implementation
Soto 01	[656]	0	$a = 0.07, 0.05, 0.03$ fm	At $\alpha _s \le 0.3$, the data have $ap = 1.4, 1.0, 0.6$
Boucaud 01A	[657]	0	$a = 0.1, 0.07, 0.05, 0.03$ fm	At $\alpha _s \le 0.3$ $ap = 1.9, 1.4, 1.0, 0.6$
Boucaud 00A	[659]	0	$a = 0.1, 0.07, 0.05, 0.03$ fm	At $\alpha _s \le 0.3$ $ap = 1.9, 1.4, 1.0, 0.6$
Boucaud 00B	[658]	0	$a = 0.1, 0.07, 0.05, 0.03$ fm	At $\alpha _s \le 0.3$ $ap = 1.9, 1.4, 1.0, 0.6$

**Table 162:** Continuum limit for $\alpha _s$ determinations with $N_f=0$ continued

Collaboration	Refs.	$N_{ f}$	$a\,\mu$	Description
SESAM 99	[623]	0	One lattice spacing with $a = 0.086\,\hbox {fm}$	$\Upsilon$ spectrum splitting
Becirevic 99A	[661]	0	$a = 0.07, 0.05$ fm	At $\alpha _s \le 0.3$ $ap = 1.4, 1.0$
Becirevic 99B	[660]	0	$a = 0.1, 0.07, 0.03$ fm	Only $a = 0.03$fm used to extract $\alpha _s$. At $\alpha _s \le 0.3$, $ap = 0.6$–1.5
ALPHA 98	[590]	0	4 to 6 spacings, $a/L = 1/12$–1 / 5 in step-scaling functions (SSF)	One-loop $\mathcal {O}(a)$ boundary improvement, linear extrapolation in *a* / *L*. $a/L = 1/8$–1 / 5 for $\alpha _s\le 0.11$ SSF, $a/L = 1/12$–1 / 5 for $0.12 \le \alpha _s\le 0.20$ SSF. $L_\mathrm{max}/r_0$ from [799], where several lattice spacings were used
Boucaud 98A	[663]	0	$a = 0.1, 0.07, 0.05$ fm	At $\alpha _s \le 0.3$, $ap = 1.9, 1.4, 1.0$
Boucaud 98B	[662]	0	$a = 0.1, 0.07, 0.05$ fm	At $\alpha _s \le 0.3$, $ap = 1.9, 1.4, 1.0$
Alles 96	[643]	0	$a \le 0.1$ fm	At $\alpha _s = 0.35$, $ap = 1.5$
Wingate 95	[624]	0	One lattice spacing with $a = 0.11\,\hbox {fm}$	Charmonium 1*S*–1*P* splitting
Davies 94	[622]	0	One lattice spacing with $a = 0.077\,\hbox {fm}$	$\Upsilon$ spectrum splitting
Lüscher 93	[579]	0	Four or five lattice spacings, $$a/L = 1/12 $$–1 / 5 in step-scaling functions	One-loop $$\mathcal {O}(a)$$ boundary improvement, linear extrapolation in *a* / *L*. $$a/L = 1/8$$–1 / 5 for $$\alpha _s\le 0.11$$ SSF, $$a/L = 1/10$$–1 / 5 for $$0.11 \le \alpha _s\le 0.22$$ SSF, $$a/L = 1/12$$–1 / 6 for $$0.22 \le \alpha _s\le 0.28$$ SSF, $$a/L = 1/8.5$$–1 / 4.5 for continuum extrapolation of $$L_\mathrm{max}/ \sqrt{K}$$
UKQCD 92	[594]	0	One lattice spacing with $0.44 \le 2a/r \le 1.6$	No continuum limit
Bali 92	[607]	0	One lattice spacing with $0.4 \le 2a/r \le 1.6$	No continuum limit
El-Khadra 92	[620]	0	Three lattice spacings with $a = 0.17,\ 0.11,\ 0.08\,\hbox {fm}$	Charmonium 1*S*–1*P* splitting

**Table 163:** Continuum limit for $\alpha _s$ determinations with $N_f=2$

Collaboration	Refs.	$N_{ f}$	$a\,\mu$	Description
Karbstein 14	[563]	2	0.32–1.19	At $p=1.5$ GeV
			0.63–1.19	At $p=3$ GeV, roughly coincides with $\alpha _s=0.3$
ALPHA 12	[12]	2	$a=0.049, 0.066, 0.076$ fm from $f_K$	Two-loop $\mathcal {O}(a)$ boundary improvement, linear extrapolation of $L_{\mathrm {max}} f_{\mathrm {K}}$ in $a^2$
Sternbeck 12	[652]	2	$a=0.073,0.07,0.06$ fm	At $\alpha _s = 0.23$, $ap = 2.1, 2.0, 1.7$
ETM 11C	[605]	2	$0.30 \le 2a/r \le 1.0$ $0.67 \le 2a/r \le 1.26$ when $\alpha _s = 0.3$	Four lattice spacings; continuum limit studied with a particular range in *r*; central result from the smallest lattice spacing, $a = 0.042$ fm
ETM 10F	[654]	2	$a=0.05,0.07,0.08$ fm. Different lattice spacings are patched together	At $\alpha _s=0.3$, $ap=1.6, 1.3, 1.1$
Sternbeck 10	[653]	2	$a=0.068,0.076,0.082$ fm	At $\alpha _s \le 0.3$, $ap \ge 1.7$
JLQCD 08	[614]	2	$a=0.12$ fm from $r_0=0.49$ fm	Single lattice spacing, $0.64< (aQ)^2 < 1.32$. At $\alpha _s = 0.3$, $ap = 0.81$
QCDSF-UKQCD 05	[625]	2	Four lattice spacings with $a = 0.10$–$0.066\,\hbox {fm}$	$r_0$, together with $r_0 = 0.467\,\hbox {fm}$
ALPHA 04	[588]	2	$a/L=1/8,1/6,1/5,1/4$	One-loop (at weak coupling) and two-loop $\mathcal {O}(a)$ boundary improvement, linear extrapolation of SSF in $(a/L)^2$
ALPHA 01A	[589]	2	$a/L = 1/6, 1/5, 1/4$	One-loop (at weak coupling) and two-loop $\mathcal {O}(a)$ boundary improvement, weighted average of SSF with $a/L=1/5,1/6$
Boucaud 01B	[644]	2	$a = 0.05, 0.07, 0.09$ fm. Data at different lattice spacings are patched together	At $\alpha _s = 0.3$, $ap = 1.6, 1.3, 0.9$; plain Wilson action with ${\mathcal {O}}(a)$ errors
SESAM 99	[623]	2	One lattice spacing with $a = 0.079\,\hbox {fm}$	$\Upsilon$ spectrum splitting
Wingate 95	[624]	2	One lattice spacing with $a = 0.11\,\hbox {fm}$	Charmonium 1*S*–1*P* splitting
Aoki 94	[621]	2	One lattice spacing with $a = 0.10\,\hbox {fm}$	Charmonium 1*P*–1*S* splitting
Davies 94	[622]	2	One lattice spacing with $a = 0.08\,\hbox {fm}$	$\Upsilon$ spectrum splitting

**Table 164:** Continuum limit for $\alpha _s$ determinations with $N_f=3$

Collaboration	Refs.	$N_{ f}$	$a\,\mu$	Description
Bazavov 14	[61]	$$2+1$$	$$2a/r = 0.52 $$–3.2	Five lattice spacings; three used for determination. At $$\alpha _\mathrm{eff}=0.3$$, then $$0.86< a\mu =2a/r < 1.3$$. $$m_\pi L =2.4,2.6,2.2$$ at smallest three lattice spacings of $$a=0.060,0.049,0.041$$ fm respectively [351]; adequate coverage of topological sectors is not clear
Bazavov 12	[604]	$2+1$	$2a/r=0.6$–2.0	7 lattice spacings; four lattice spacings with $1.14 \le 2a/r \le 1.5$ when $\alpha _s(1/r) = 0.3$. $2a/r = 2$ when $\alpha _s(1/r) = 0.23$ (on the finest lattice)
Sternbeck 12	[652]	$2+1$	$a=0.07$ fm	At $\alpha _s = 0.23$, $ap = 2.1$
HPQCD 10	[9]	$2+1$	$a\mu =2 a\bar{m}_h = 0.61$–1.75	Five lattice spacings; three lattice spacings with $1.0 \le a\mu \le 1.5$ when $\alpha _{R_4}(\mu ) \le 0.3$; three lattice spacings with $1.0 \le a\mu \le 1.5$ when $\alpha _{R_6/R_8}(\mu ) \le 0.33$
JLQCD 10	[613]	$2+1$	$a = 0.11$ fm from $r_0 = 0.49$ fm	Single lattice spacing, $0.4< (aQ)^2 < 1.0$ for the momentum fit range. At $\alpha _s = 0.3$, $ap = 0.89$
HPQCD 10	[9]	$2+1$		Update of $r_1$ and $r_1/a$ in HPQCD 08A
PACS-CS 09A	[62]	$2+1$	$a/L=1/8,1/6,1/4$	Tree-level $\mathcal {O}(a)$ boundary improvement, which has been seen to behave better than one-loop in simulations [582]; weighted average of $a/L=1/8,1/6$ for step-scaling function which agrees with a linear extrapolation in *a* / *L* of all data points of the SSF. Linear extrapolation in *a* / *L* of $L_{\mathrm {max}}m_\rho$ with $a/L_{\mathrm {max}} = 1/8, 1/6, 1/4$
HPQCD 08B	[152]	$2+1$	$a\mu =2 a\bar{m}_h = 0.8,$ 1.2, 1.7, 2.1	Four lattice spacings with heavy-quark mass approximately the charm mass, where $\alpha _{R_4}(\mu ) = 0.38$
HPQCD 08A	[617]	$2+1$	Six lattice spacings with $a = 0.18$–$0.045\,\hbox {fm}$	$r_1$ using $\Upsilon$ spectrum splitting
Maltman 08	[63]	$2+1$	Five lattice spacings with $a = 0.18$–$0.06\,\hbox {fm}$	Re-analysis of HPQCD 05A with additional lattice spacings $a = 0.06$, $0.15\,\hbox {fm}$
HPQCD 05A	[616]	$2+1$	Three lattice spacings with $a = 0.18$–$0.09\,\hbox {fm}$	$r_1$ using $\Upsilon$ spectrum splitting

**Table 165:** Continuum limit for $\alpha _s$ determinations with $N_f=4$

Collaboration	Refs.	$N_{ f}$	$a\,\mu$	Description
HPQCD 14A	[5]	$2+1+1$	$a\mu =2 a\bar{m}_h = 0.78$–2.09	Four lattice spacings; two lattice spacings with $a\mu \le 1.5$ and one more lattice spacing with $a\mu \lesssim 1.6$ when $\alpha _{R_4}(\mu ) \le 0.3$
ETM 13D	[649]	$2+1+1$	$a=0.060, 0.068$ fm from $f_\pi$	For $\alpha _s \le 0.3$, $ap=1.5,1.7$. Update of [650]
ETM 12C	[650]	$2+1+1$	$a=0.061, 0.078$ fm from $f_\pi$	Global fit with $(ap)^2$ discretization effects. For $\alpha _s \le 0.3$, $ap = 1.5, 2.2$
ETM 11D	[651]	$2+1+1$	$a = 0.061, 0,078$ fm	For $\alpha _s \le 0.3$, $ap = 1.5, 2.0$
ALPHA 10A	[586]	4	$a/L = 1/4, 1/6, 1/8$	Constant or global linear fit in $(a/L)^2$
Perez 10	[587]	4	$a/L=1/4, 1/6, 1/8$	Linear extrapolation in $(a/L)^2$. one-loop improvement at the boundary

## Footnotes

1 Peter Boyle had participated actively in the early stages of the current FLAG effort. Unfortunately, due to other commitments, it was impossible for him to contribute until the end, and he decided to withdraw from the collaboration.

2 The WG on semileptonic *D* and *B* decays has currently four members, but only three of them belong to lattice collaborations.

3 We also use terms like “quality criteria”, “rating”, “colour coding” etc. when referring to the classification of results, as described in Sect. 2.

4 We note, however, that the data-driven criteria can be used by individual working groups in order to rate the reliability of the analyses for specific quantities.

5 We refer to Sect. 5.1 and Appendix A.4 in the Glossary for an explanation of the various regimes of chiral perturbation theory.

6 This is a change from Ref. [2], where we used the charged pion mass when evaluating both chiral-extrapolation and finite-volume effects.

7 We refer the interested reader to a number of good reviews on the subject [85–89].

8 In Ref. [2], the RMS value was used in the chiral-extrapolation criteria throughout the paper. For the finite-volume rating, however, $M_{\pi ,\mathrm {min}}$ was identified with the RMS value only in Sects. 4 and 6, while in Sects. 3, 5, 7 and 8 it was identified with the mass of the lightest pseudoscalar state.

9 For example, for quark mass results we distinguish between perturbative and nonperturbative renormalization, for low-energy constants we distinguish between the *p*- and $\epsilon$-regimes, and for heavy flavour results we distinguish between those from leptonic and semi-leptonic decays.

10 Whenever figures turn out to be overcrowded, older, superseded results are omitted. However, all the most recent results from each collaboration are displayed.

11 Note that a different convention is used in the analysis of the precision measurements carried out in low-energy pion physics (e.g. Ref. [97]). When comparing lattice results with experiment, it is important to fix the QCD parameters in accordance with the convention used in the analysis of the experimental data (for a more detailed discussion, see Refs. [98–101]).

12 Sometimes, e.g. in Ref. [103], the violation of Dashen’s theorem is given in terms of a different quantity, $\bar{\epsilon }\equiv (\Delta _{K^+}^\gamma -\Delta _{K^0}^\gamma )/(\Delta _{\pi ^+}^\gamma -\Delta _{\pi ^0}^\gamma )-1$. This parameter is related to $\epsilon$ used here through $\epsilon =(1-\epsilon _m)\bar{\epsilon }$. Given the value of $\epsilon _m$ (see Eq. (13)), these two quantities differ by 4% only.

13 To obtain this number, we have used the conversion from $\mu =3$ GeV to $m_c$ given in Ref. [152].

14 Note that in Sect. 9.7.2 different coding criteria are adopted and the HPQCD 14A paper is tagged differently for the continuum extrapolation.

15 The pion decay constant represents a QCD matrix element – in the full Standard Model, the one-pion state is not a meaningful notion: the correlation function of the charged axial current does not have a pole at $p^2=M_{\pi ^+}^2$, but a branch cut extending from $M_{\pi ^+}^2$ to $\infty$. The analytic properties of the correlation function and the problems encountered in the determination of $f_\pi$ are thoroughly discussed in Ref. [185]. The “experimental” value of $f_\pi$ depends on the convention used when splitting the sum $\mathcal{L}_{\mathrm{QCD}}+\mathcal{L}_{\mathrm{QED}}$ into two parts (compare Sect. 3.1.1). The lattice determinations of $f_\pi$ do not yet reach the accuracy where this is of significance, but at the precision claimed by the Particle Data Group [151, 184], the numerical value does depend on the convention used [98–100, 185].

16 It is not a trivial matter to perform the data analysis at this precision. In particular, isospin-breaking effects need to be properly accounted for [187–192]. For a review of recent work on this issue, we refer to Ref. [186].

17 Fortran programs for the numerical evaluation of the form factor representation in Ref. [227] are available on request from Johan Bijnens.

18 Here we have symmetrized the asymmetric systematic error and shifted the central value by half the difference as will be done throughout this section.

19 Note that the ellipses shown in Fig. 5 of both Ref. [1] and Ref. [2] correspond instead to the 39% likelihood contours. Note also that in Ref. [2] the likelihood was erroneously stated to be $68 \%$ rather than $39 \%$.

20 More precisely, they are independent of the 2 or 3 light-quark masses which are explicitly considered in the respective framework. However, all low-energy constants depend on the masses of the remaining quarks *s*, *c*, *b*, *t* or *c*, *b*, *t* in the *SU*(2) and *SU*(3) framework, respectively, although the dependence on the masses of the *c*, *b*, *t* quarks is expected to be small.

21 Here and in the following, we stick to the notation used in the papers where the $\chi$PT formulae were established, i.e. we work with $F_\pi \equiv f_\pi /\sqrt{2}=92.2(1)\,\mathrm {MeV}$ and $F_K\equiv f_K/\sqrt{2}$. The occurrence of different normalization conventions is not convenient, but avoiding it by reformulating the formulae in terms of $f_\pi$, $f_K$ is not a good way out. Since we are using different symbols, confusion cannot arise.

22 Notice that one could analyse the quark-mass dependence entirely in terms of the parameter $M^2$ defined in Eq. (74) and determine equally well all other LECs. Using the determination of the quark masses described in Sect. 3 one can then extract *B* or $\Sigma$. No matter the strategy of extraction, determination of *B* or $\Sigma$ requires knowledge of the scale and scheme dependent quark mass renormalization factor $Z_m(\mu )$.

23 By introducing an imaginary isospin chemical potential, the framework can be extended such that the low-lying spectrum of the Dirac operator is also sensitive to the pseudoscalar decay constant *F* at LO [315].

24 Higher-order systematic effects in the matching with RMT have been investigated in Refs. [316, 317].

25 The results for $\Sigma$ and *F* lie in the same range as the determinations reported in Tables 19 and 20.

26 Some of the $\mathcal {O}(p^6)$ formulae presented below have been derived in an unpublished note by three of us (GC, SD and HL) and Jürg Gasser. We thank him for allowing us to publish them here.

27 There are theoretical arguments suggesting that the $\xi$ expansion is preferable to the *x* expansion, based on the observation that the coefficients in front of the squared logs in Eq. (73) are somewhat larger than in Eq. (78). This can be traced to the fact that a part of every formula in the *x* expansion is concerned with locating the position of the pion pole (at the previous order) while in the $\xi$ expansion the knowledge of this position is built in exactly. Numerical evidence supporting this view is presented in Ref. [138].

28 This means that for any given lattice formulation one needs to determine additional lattice-artefact low-energy constants. For certain formulations, e.g. the twisted-mass approach, first steps in this direction have already been taken [332], while with staggered fermions MILC routinely does so; see e.g. Refs. [89, 107].

29 Also in the case of Refs. [337, 338] the colour-coding criteria for the $\epsilon$-regime have been applied.

30 There are two naive procedures to symmetrize an asymmetric systematic error: (i) keep the central value untouched and enlarge the smaller error, (ii) shift the central value by half of the difference between the two original errors and enlarge/shrink both errors by the same amount. Our procedure (iii) is to average the results of (i) and (ii). In other words a result $$c(s)\left( {\begin{array}{c}+u\\ -\ell \end{array}}\right) $$ with $\ell >u$ is changed into $c+(u-\ell )/4$ with statistical error *s* and a symmetric systematic error $(u+3\ell )/4$. The case $\ell <u$ is handled accordingly.

31 We thank Martin Lüscher for an interesting discussion of this issue.

32 Thanks to QCD parity invariance we can ignore three more dimension-six operators whose parity conserving parts coincide with the corresponding parity conserving contributions of the operators $Q_1, Q_2$ and $Q_3$.

33 Notice that the contribution of $|V_{cb}|^2$ to the unitarity relation is more than one order of magnitude below the quoted error, and it can therefore be neglected.

34 This situation differs from that of calculations of the $K\rightarrow \pi \ell \nu$ form factor, where the physical-pion recoil momenta are smaller than $2 \pi /L$. For $K\rightarrow \pi \ell \nu$ it is now standard to use nonperiodic (“twisted”) boundary conditions [429, 430] to simulate directly at $q^2=0$; see Sect. 4.3. Some collaborations have also begun to use twisted boundary conditions for *D* decays [431–434].

35 In Ref. [441], to be discussed below, form factors are indeed computed for several values of $q^2$, and fitted to a Bećirević–Kaidalov parameterization (cf. Sect. 8.3.1) to extract their values at $q^2=0$. However, while results for fit parameters are provided, the values of the form factors at $q^2 \ne 0$ are not provided, which prevents us from performing an independent analysis of their shape using model-independent parameterizations.

36 The neutral *B*-meson leptonic decays, $B_{d,s} \rightarrow \mu ^{+} \mu ^{-}$, were recently observed at the LHC experiments, and the corresponding branching fractions can be obtained by combining the data from the CMS and the LHCb Collaborations [450]. Nevertheless, the errors of these experimental results are currently too large to enable a precise determination of $|V_{td}|$ and $|V_{ts}|$.

37 The authors of RBC/UKQCD 14 claim that using the NLO *SU*(3)-HM$\chi$PT extrapolation formulae, acceptable fits for the decay constants can be found. On the other hand, no reasonable fit for the ratio, $f_{B_{s}}/f_{B}$, can result from this procedure, because this ratio has smaller statistical errors. The NLO *SU*(3)-HM$\chi$PT predictions are then used as a means to estimate the systematic effects arising from the chiral-continuum extrapolation.

38 At the Lattice 2015 conference, the Fermilab Lattice and MILC Collaborations reported their on-going project for computing the *B*-meson-decay constants in $N_{f}=2+1+1$ QCD [479]. However, no result has been shown yet.

39 We thank Ruth Van de Water for communication and discussion regarding the comparison of the averaging strategies.

40 We thank Ruth Van de Water and Andre Walker-Loud for helpful discussion of this point.

41 The variance of hadron correlation functions at nonzero three-momentum is dominated at large Euclidean times by zero-momentum multiparticle states [488]; therefore the noise-to-signal grows more rapidly than for the vanishing three-momentum case.

42 Upcoming data from Belle II are expected to significantly improve the precision of experimental results, in particular, for larger values of $q^2$.

43 A more complicated analytic structure may arise in other cases, such as channels with vector mesons in the final state. We will, however, not discuss form-factor parameterizations for any such process.

44 We are grateful to G. Herdoíza, R.J. Hill, A. Kronfeld and A. Szczepaniak for illuminating discussions on this issue.

45 Since the new FNAL/MILC results supersede Ref. [437], we will not discuss the latter work in the present version of the review.

46 Note that issues are known to exist with hard-pion $\chi$PT, cf. Ref. [510].

47 Note that generating synthetic data is a trivial task but less so the number of required points and the $q^2$ values that lead to an optimal description of the form factors.

48 The values of the scalar resonance mass in $B\pi$ scattering taken by HPQCD and RBC/UKQCD are $M_{B^*(0+)}=5.6794(10)~\,\mathrm {GeV}$ and $M_{B^*(0+)}=5.63~\,\mathrm {GeV}$, respectively. We use an average of the two values, and have checked that changing it by ${\sim } 1\%$ has a negligible impact on the fit results.

49 In this case, $\mathcal {O}(z^4)$ fits with just two degrees of freedom, are significantly less stable. Still, the results for $a_0^+$ and $a_1^+$ are always compatible with the ones at $\mathcal {O}(z^2)$ and $\mathcal {O}(z^3)$ within one standard deviation.

50 See, e.g., Ref. [513] and references therein.

51 Note in particular that not much is known about the sub-threshold poles for the scalar form factor. FNAL/MILC includes one pole at the $B_{s0}^*$ mass as taken from the calculation in Ref. [520].

52 In the cases such as $B\rightarrow D^*$ transitions, which will be discussed below, this is much less of a practical problem due to the very narrow nature of the resonance.

53 As noted above, this is the same approach adopted by FNAL/MILC in their fits to a BGL ansatz. HPQCD, meanwhile, uses one single pole in the pole factors that enter their modified *z*-expansion, using their spectral studies to fix the value of the relevant resonance masses.

54 A similar ratio $R(D^*)$ can be considered for $B \rightarrow D^*$ transitions – as a matter of fact, the experimental value of $R(D^*)$ is significantly more accurate than the one of *R*(*D*). However, the absence of lattice results for the $B\rightarrow D^*$ scalar form factor, and indeed of results at nonzero recoil (see below), takes $R(D^*)$ out of our current scope.

55 See Ref. [450] for the latest results, obtained from a joint analysis of CMS and LHCb data.

56 This work also provided a value for *R*(*D*), now superseded by Ref. [540].

57 Since $\ell =e,\mu$ the contribution from the scalar form factor in Eq. (172) is negligible.

58 See, e.g., Sect. V.D of  [504] for a detailed discussion.

59 The same work provides ${\mathcal {G}}^{B_s\rightarrow D_s}$, for which there are, however, no experimental data.

60 Note that this determination does not include the electromagnetic Coulomb correction roughly estimated in Ref. [539]. Currently the numerical impact of this correction is negligible.

61 We thank Marcello Rotondo for providing the ten bins result of the BaBar analysis.

62 We have checked that results using just one experimental dataset are compatible within $1\sigma$. In the case of BaBar, we have taken into account the introduction of some EW corrections in the data.

63 Once one is in the perturbative region with $\alpha _\mathrm{eff}$, the error in extracting the $\Lambda$ parameter due to the truncation of perturbation theory scales like $\alpha _\mathrm{eff}^{n_\mathrm {l}}$, as seen e.g. in Eq. (218). In order to well detect/control such corrections, one needs to change the correction term significantly; we require a factor of four for a  and a factor 2.25 for a . In comparison to FLAG 13, where $n_l=2$ was taken as the default, we have made the $n_l$ dependence explicit and list it in Tables 157, 158, 159 and 160. An exception to the above is the situation where the correction terms are small anyway, i.e. $\alpha _\mathrm{eff}^{n_\mathrm {l}} \approx 0.02$ is reached.

64 Note also that the determination of the scale does not need to be very precise, since using the lowest-order $\beta$-function shows that a 3% error in the scale determination corresponds to a ${\sim } 0.5\%$ error in $\alpha _s(M_Z)$. So as long as systematic errors from chiral-extrapolation and finite-volume effects are below 3% we do not need to be concerned about those. This covers most cases.

65 We simplify here and assume that the classical solution associated with the used boundary conditions has charge zero. In practice this is the case.

66 With two-loop $\mathcal {O}(a)$ improvement we here mean $c_\mathrm {t}$ including the $g_0^4$ term and $\tilde{c}_\mathrm {t}$ with the $g_0^2$ term. For gluonic observables such as the running coupling this is sufficient for cutoff effects being suppressed to $\mathcal {O}(g^6 a)$.

67 Note that at large distances, where string breaking is known to occur, this is not any more the ground state potential defined by Eq. (238).

68
$\alpha _\mathrm {V'}$ is defined by $\Lambda _\mathrm {V'}=\Lambda _\mathrm {V}$ and $b_i^\mathrm {V'}=b_i^\mathrm {V}$ for $i=0,1,2$ but $b_i^\mathrm {V'}=0$ for $i\ge 3$.

69 The scale $w_{0.4}$ used in FlowQCD 15 [564] is a modified $w_0$ Wilson flow scale. With this notation $w_0 \equiv w_{0.3}$.

70 From Eq. (223) we see that $\alpha _s$ is continuous and differentiable across the mass thresholds (at the same scale). Therefore to leading order $\alpha _s$ and $\Delta \alpha _s$ are independent of $N_f$.

71 We have symmetrized the asymmetric error bars of Bazavov 14 [61] to 0.1166(10) in taking the average.

72 More precisely, we use $\alpha _{\overline{\mathrm{MS}}}^{(3)}(5\,\mathrm {GeV})=0.203$ corresponding to Eq. (263) and $\alpha _{\overline{\mathrm{MS}}}^{(5)}(M_\mathrm {Z})=0.1182$ in Eqs. (251) and (252).

73 We have assigned a  for the continuum limit, in Boucaud 00A [659], 00B [658], 01A [657], Soto 01 [656] but these results are from lattices of a very small physical size with finite-size effects that are not easily quantified.
